# Robust SNP genotyping by multiplex PCR and arrayed primer extension

**DOI:** 10.1186/1755-8794-1-5

**Published:** 2008-01-31

**Authors:** Mohua Podder, Jian Ruan, Ben W Tripp, Zane E Chu, Scott J Tebbutt

**Affiliations:** 1 The James Hogg iCAPTURE Centre for Cardiovascular and Pulmonary Research, St. Paul's Hospital, University of British Columbia, Vancouver, BC, V6Z 1Y6, Canada; 2 Department of Statistics, University of British Columbia, Vancouver, BC, V6T 1Z2, Canada; 3 Department of Medicine, Division of Respiratory Medicine, University of British Columbia, Vancouver, BC, V6Z 1Y6, Canada; 4 Division of Engineering Science, University of Toronto, Toronto, ON, M5S 2E4, Canada

## Abstract

**Background:**

Arrayed primer extension (APEX) is a microarray-based rapid minisequencing methodology that may have utility in 'personalized medicine' applications that involve genetic diagnostics of single nucleotide polymorphisms (SNPs). However, to date there have been few reports that objectively evaluate the assay completion rate, call rate and accuracy of APEX. We have further developed robust assay design, chemistry and analysis methodologies, and have sought to determine how effective APEX is in comparison to leading 'gold-standard' genotyping platforms. Our methods have been tested against industry-leading technologies in two blinded experiments based on Coriell DNA samples and SNP genotype data from the International HapMap Project.

**Results:**

In the first experiment, we genotyped 50 SNPs across the entire 270 HapMap Coriell DNA sample set. For each Coriell sample, DNA template was amplified in a total of 7 multiplex PCRs prior to genotyping. We obtained good results for 41 of the SNPs, with 99.8% genotype concordance with HapMap data, at an automated call rate of 94.9% (not including the 9 failed SNPs). In the second experiment, involving modifications to the initial DNA amplification so that a single 50-plex PCR could be achieved, genotyping of the same 50 SNPs across each of 49 randomly chosen Coriell DNA samples allowed extremely robust 50-plex genotyping from as little as 5 ng of DNA, with 100% assay completion rate, 100% call rate and >99.9% accuracy.

**Conclusion:**

We have shown our methods to be effective for robust multiplex SNP genotyping using APEX, with 100% call rate and >99.9% accuracy. We believe that such methodology may be useful in future point-of-care clinical diagnostic applications where accuracy and call rate are both paramount.

## Background

If 'personalized medicine', using genomic knowledge, is to become a reality, then the ability to determine the most appropriate clinical intervention for a patient will require the genotyping of several tens to hundreds of single nucleotide polymorphisms (SNPs) across many genes and their regulatory sequences for that individual patient [1,2], rapidly and at the point-of-care. Of many genotyping methods, those based on microarrays offer the greatest potential for economic, patient-specific application [3-7], due to their ability to simultaneously interrogate multiple SNPs. Arrayed primer extension (APEX [8,9]) is a minisequencing microarray assay based on a two-dimensional array of oligonucleotide probes that are immobilized, via their 5' ends, on a glass surface. The probes (25-mers) are designed so that they are complementary to the gene up to, but not including, the base where the SNP exists. The Sanger-based sequencing chemistry of APEX allows genotyping of hundreds of SNPs, with the array chemistry taking only fifteen to twenty minutes to complete. APEX achieves this clinically relevant speed because it uses the catalytic ability of a DNA polymerase to carry out a single nucleotide base extension (SBE) at the 3' end of the arrayed probes, specific to the SNP sites of interest in amplified patient DNA that is temporarily hybridized to these probes. The dideoxynucleotide (ddNTP) 'terminator' bases are labelled with tags containing distinct fluorescent chromophores, specific for each of the four bases of DNA (A,C,G,T). Hence, the fluorescent 'colour' at each of the probe sites (array spots) will give SNP-specific genotypic information. As a discovery research tool, APEX has been used to detect β-thalassemia [10], p53 [11], and BRCA1 mutations [12]. Importantly, APEX has also been shown to be efficient at simultaneously genotyping SNP markers that are widely dispersed across the human genome [13,14]; such capability is essential for future 'individualized' genomic diagnostic analysis across multiple genes and pathways that are relevant to disease. In a recent quality assessment survey of SNP genotyping laboratories [15], in which up to 18 SNPs were genotyped across 47 DNA samples, APEX performed well against other methods, and the authors concluded that a "conservative approach for calling the genotypes should be used to achieve a high accuracy at the cost of a lower genotyping success rate." Whilst such a conservative approach may be applicable for research studies, it may not be appropriate for clinical diagnostics, in which life-saving medical decisions might require extremely accurate genotyping across all SNPs of interest.

Given the potential utility of APEX for rapid clinical diagnostics, we have developed robust assay design, chemistry and analysis methodologies, and have sought to determine just how effective APEX is in comparison to leading 'gold-standard' genotyping platforms, including Perlegen and Illumina. Our objective was to achieve 100% assay completion rate, call rate and genotyping accuracy rate, for multiple SNPs across multiple samples. Previous studies from our laboratory have reported APEX genotyping accuracies ranging from 98% to 99.8% [14,16-18], though the call rates in these studies have always been significantly lower than 100%, and usually do not include a proportion of the originally selected SNPs that fail the assay. Similarly, other laboratories that use APEX and equivalent technology have reported genotyping accuracies ranging from 98% to >99%, with call rates varying from 84.4% to 96.8% [10,11,13,15,19-21].

## Results and Discussion

**Figure 1:**
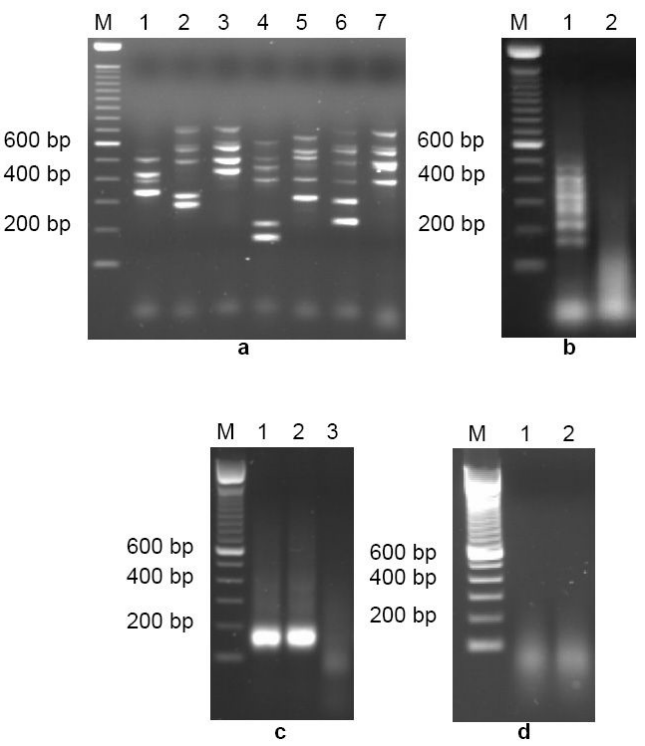
Multiplexing PCR and subsequent amplicon fragmentation results, prior to APEX reaction on HapMap Chip. (**a**) Standard multiplex PCR from a single Coriell DNA sample using optimally-designed primers [Additional files 1 &2] within seven unique multiplex groups (lanes 1–7; lane M shows 100 bp DNA ladder markers), showing wide range of amplicon sizes across the 50 SNP loci. (**b**) Purification, concentration and fragmentation of standard PCR amplicons. Lane 1 represents an aliquot of concentrated mixture of all seven multiplex products shown in **Fig. 1a**. Lane 2 shows the fragmentation result, generating single-stranded nucleic acid of 30–100 base length. (**c**) Multiplex PCR amplification of all 50 SNP loci in a single reaction tube using new PCR primer set [Additional file 6], showing 50-plex PCR products (individual SNP loci amplicons are unresolvable by agarose gel electrophoresis) from two Coriell DNA samples (lanes 1 & 2), plus a negative PCR control (lane 3). (**d**) Fragmentation of 50-plex PCR amplicons from aliquots of lane 1 & lane 2 samples shown in **Fig. 1c**.

We selected 50 SNPs from the HapMap database that had been previously genotyped and analyzed as part of the third quality control exercise on Illumina and Perlegen platforms, arguably the most accurate and best validated high-throughput methodologies for SNP genotyping to date. The randomly selected SNPs were located across multiple chromosomes and are listed in Additional file 1 online, along with details of the APEX probe sequences and PCR primer sequences. The genotyping arrays that are currently being developed and tested in our laboratory incorporate multiple redundant measures consisting of sense and antisense DNA-strand APEX probes plus allele-specific oligonucleotide (ASO) APEX probes for a total of six different probes per SNP [14], with each replicated five times on the array grid, which allows for more robust statistical averaging. Optimal PCR primer pairs were designed for each of the 50 SNP loci [Additional file 1] and seven multiplex PCR groups were set up that, together, would amplify all 50 loci [Additional file 2]. We obtained a set of 287 DNA samples from McGill University and Génome Québec Innovation Centre (one of the HapMap Project's genotyping centers). This set comprised 270 DNA samples from the Coriell Institute for Medical Research [22] plus hidden duplicates and negative controls, all of which our laboratory was blinded to. PCR [Fig. 1a and Fig. 1b] and APEX assays were performed on each of the samples, plus a 10% repeat set which was randomly selected by us to allow internal quality control and an initial assessment of genotyping concordance.

Microarray image data were imported into SNP Chart [23] and analyzed using previously described image analysis algorithms [24,25]. Genotypes were called using two previously published methods: 1. MACGT software [17], which is a multi-dimensional clustering tool; 2. simple linear discriminant analysis (LDA) using dynamic variable selection [18], which is a classification algorithm. Results are shown in Table 1 and Additional file 3 online. Briefly, a training set was established using SNP Chart, followed by auto-calling in MACGT. Nine SNPs did not pass quality control due to assay failure or inconsistent PCR amplification. For all remaining SNPs that were auto-called by MACGT, any genotypes that had a 'fit' score of less than 0.001 (approximately 9%) were checked by manual scoring in SNP Chart and either validated, or changed to a different genotype or to a non-call (NN). The final results using MACGT showed highly accurate genotyping (99.94% concordance with HapMap) with good call rates (90% auto-called plus 9% manual scoring). Importantly, of the 1,013 genotypes called manually, the accuracy was 99.87%, even in cases where the array spot signal intensities were up to an order of magnitude lower than for higher quality genotype data, and only slightly higher than background signals [Additional file 4 and Additional file 5]. Using the same training set, we then analyzed the data set with simple linear discriminant analysis (LDA) using dynamic variable selection [18]. Results [Table 1 and Additional file 3] also showed accurate genotyping (99.91% HapMap concordance), and with higher automated call-rates (94.91% – using a confidence score threshold of 0.75). We also calculated the homozygous and heterozygous performance for the set of 270 HapMap samples with the previously selected 41 SNPs out of 50 SNPs [See Table 1]. For a threshold of 0.75, we were able to call 6883 cases out of 7214 homozygous cases (95.41% call rate) with 6880 correct calls (99.96% HapMap concordance). Whereas, with the same threshold, out of 3873 heterozygous cases, we were able to call 3640 cases (93.98% call rate) with 3634 correct calls (99.84% HapMap concordance). Therefore, in common with other genotyping platforms, our methodology has a slight bias that favours the calling of homozygous genotypes.

**Table 1:** Results summary for 287 HapMap samples and 41 SNPs

**Method**	**Call rate**	**Concordance with HapMap**
MACGT (0.001 cut-off) + manual calls	98.90% (9% manual calls)	99.94%
LDA (0.75 threshold) Total cases	94.91% (10,523 cases vs. 11,087)	99.91% (10,514 vs. 10,523)
LDA (0.75 threshold) Homozygous cases	95.41% (6,883 vs. 7,214)	99.96% (6,880 vs. 6,883)
LDA (0.75 threshold) Heterozygous cases	93.98% (3,640 vs. 3,873)	99.84% (3,634 vs. 3,640)

These results, although promising and at least as accurate as any previously reported for APEX-based methodologies, did not deliver on our objective of 100% call rate and 100% accuracy, and several of the 50 SNPs failed quality control. However, two important lessons were learnt from the study: 1. our on-chip assay chemistry is extremely robust and specific, allowing accurate genotype calls (at least by manual inspection of the array spot data within SNP Chart) even at very low sensitivities (*i.e*., when the sequence-specific spot intensities are only slightly higher than background signals); 2. non-calls (NNs) generally resulted from sporadic PCR failure for certain amplicons, especially those of a length greater than 650–700 base pairs (bp). Taken together, our results suggested that even if specific SNPs give high NN rates across multiple samples, the genotypes for the remaining samples for these SNPs (for which APEX assay data can be obtained) are still very accurate, despite low signal to noise. We believe that this is due to the redundancy in the genotyping probe design: two classical APEX probes (one probe per DNA strand), plus four allele-specific (ASO) APEX probes (two probes per strand), each replicated five times, for each SNP site. When this redundant data is displayed in a SNP Chart, it is relatively straightforward to interpret the genotype manually [Additional file 4 and Additional file 5]. From these conclusions we reasoned that the PCR design itself needed to be addressed, so that sporadic failures (despite good primer design algorithms) could be consistently minimized or even eliminated.

For SNP genotyping, only the immediate sequence around the SNP site is of interest. Therefore, keeping the PCR amplicon size to a minimum ensures short extension times and minimal use of reagents. However, sequence-context issues, especially in multiplex PCR, necessitate the design of unique primers that have balanced annealing temperatures. This requirement can result in individual amplicon sizes in a multiplex mix ranging from 100 to >700 bp [14]. Large amplicons are optimal neither for fast PCR nor for the subsequent APEX assay, which requires amplicons to be fragmented to ~50–100 base lengths [Fig. 1b]. In addition, the degree of multiplexing is usually limited to between four and ten amplicons per individual multiplex PCR: *e.g*., for our original HapMap chip, the 50 SNP loci are amplified in a total of seven separate multiplex reactions [Fig. 1a and Additional file 2]. We initially tested multiplex PCR using all original PCR amplicon primer pairs in a single reaction. As expected, several experimental attempts all failed to amplify even a modest proportion of the 50 amplicons (typically, less than 20 amplicons would be successful; data not shown). Thus, our new objectives were to increase the degree of multiplexing and shorten the amplicon lengths to less than 200 bp, so that all 50 SNP loci could be simultaneously and robustly amplified in a single reaction vessel. New PCR primers were designed for the 50 HapMap SNP loci, with amplicon sizes restricted to between 100 and 200 bp [Additional file 6]. Because of this limitation, we were not able to optimally design the primers based on a balanced melting temperature (Tm). To try to compensate for this potential problem, each new PCR primer had a common linker sequence designed at its 5' end (5' TACGACTCACTTAGGGAG 3' for each of the left hand PCR primers/5' CGATGTAGGTGACACTAG 3' for each of the right hand PCR primers). These linkers have two properties: a balanced and reasonably high GC content to increase the melting temperature of the primer and a unique sequence not found in the human DNA template [26]. After the first few cycles of PCR, the linker sequence becomes incorporated into the amplicon sequence and is amplified along with the template sequence. This approach helps reduce primer-dimer formation during the PCR [27]. Because the primers have balanced GC content, primer annealing in later cycles of PCR should become much more sensitive and robust [28]. We randomly selected 50 of the HapMap Coriell DNA samples from our initial study, for 50-plex PCR using the pool of linker-modified primers. Specific PCR cycling conditions were adopted from a previously published study by Wang *et al*. [28]. We also attempted 50-plex PCR using the redesigned PCR primers, but without the common 5' linker sequences: we managed to amplify only a modest number of the 50 SNPs, and this multiplex PCR was not robust and we could never amplify all 50 SNPs (data not shown).

**Figure 2:**
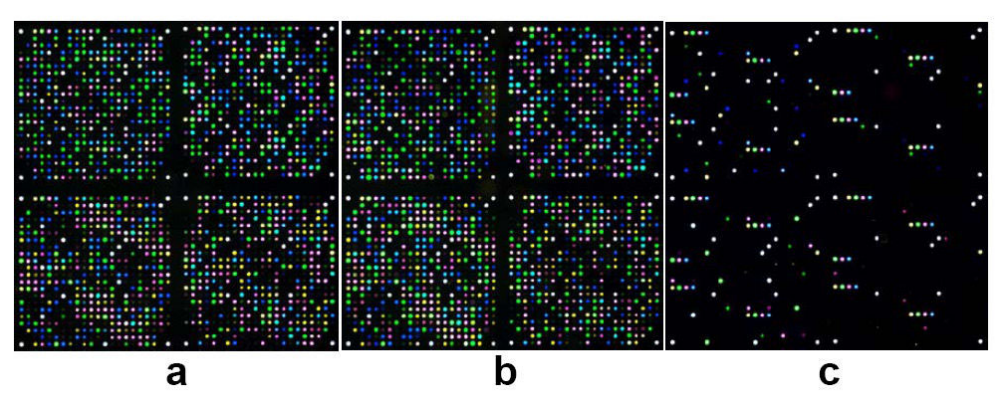
HapMap Chip four colour microarray images showing successful de-multiplexing of 50-plex PCR from two Coriell DNA samples (**a**, **b**), plus a negative control sample (**c**), prior to image analysis and automated genotyping. The spots on the negative control image represent positive control probes [8, 14].

PCR [Fig. 1c and Fig. 1d] and APEX assays [Fig. 2] were performed on each of the samples, including negative controls. Microarray image data were imported into SNP Chart and analyzed as described previously. Genotype calling was performed using three independent methods: 1. manual calling in SNP Chart; 2. auto-calling with MACGT; and 3. auto-calling by LDA using dynamic variable selection. Genotypes were compared to HapMap data for concordance. One SNP (rs7693776) was monomorphic (TT) across all samples genotyped. Results are presented in Table 2 and [Additional files 7, 8, 9, 10]. Manual genotype calling, although time-consuming and vulnerable to user-subjectivity issues [14,23], is nevertheless an accurate and validated way to interpret APEX data, especially at low spot intensity levels (see above). In addition, manual calling does not require the use of a training set. Of the 49 Coriell DNA samples (one sample out of the random set was a blinded negative control sample) assayed across 50 SNPs, manual calls were made for all possible 2,450 genotypes (100% assay completion and 100% call rate). Of these, 2,448 were concordant with HapMap data (99.92%). The two discrepant genotypes were for two different samples each at different SNP loci. Interestingly, the SNP Charts for these two genotypes showed high quality data, and the same samples/genotypes had previously been concordant with HapMap in the initial data set [Additional file 3, and discussed further below].

**Table 2:** Results summary for 49 HapMap samples and 50 SNPs

**Method**	**Call rate**	**Concordance with HapMap**
Manual calling only	100%	99.92%^1^
MACGT (no cut-off)	100%	99.84%^2^
LDA (0 threshold) Total cases	100% (1,941 cases vs. 1,941)	99.89%^3 ^(1,939 vs. 1,941)
LDA (0 threshold) Homozygous cases	100% (1,289 vs. 1,289)	100%^4 ^(1,289 vs. 1,289)
LDA (0 threshold) Heterozygous cases	100% (652 vs. 652)	99.7%^5 ^(650 vs. 652)
MACGT (0.001 cut-off)	94.04%	99.94%^6^
LDA (0.65 threshold) Total cases	99.18% (1,925 vs. 1,941)	99.90%^3 ^(1,923 vs. 1,925)
LDA (0.65 threshold) Homozygous cases	98.91%^7 ^(1,275 vs. 1,289)	100% (1,275 vs. 1,275)
LDA (0.65 threshold) Heterozygous cases	99.7% (650 vs. 652)	99.7% (648 vs. 650)

^1 ^Two discrepancies amongst 2,450 genotype cases.

^2 ^Three discrepancies amongst 1,926 genotype cases (524 cases used in training set).

^3 ^Two discrepancies amongst 1,941 genotype cases (509 cases used in training set).

^4 ^No discrepancy amongst 1289 cases (327 cases used in training set)

^5 ^Two discrepancies amongst 652 cases (182 cases used in training set)

^6 ^One discrepancy amongst 1,926 genotype cases (524 cases used in training set).

^7 ^Eleven predictions (all TT and correct) with confidence score less than 0.65 for a single SNP (rs1891403).

Auto-calling was independently undertaken. Initially, MACGT cluster plots and quality control using SNP Chart were used to allow manual selection of a limited training set of samples from the data set [17]. Using this training set, MACGT auto-calling of the test set with a 0.001 fit threshold resulted in a call rate of 94.04% and a concordance rate of 99.94%. When the fit threshold was relaxed to achieve a 100% call rate, three genotypes were discordant with HapMap data. Two of these genotypes (both with high fit values – good confidence scores) were the same as the two that had been identified as part of the manual calling data. The third discrepancy had a relatively poor fit confidence score. LDA with dynamic variable selection, using a slightly reduced sized training set, yielded identical genotyping results to manual calling, at a 100% call rate across all 50 SNPs (16 NNs at a 0.65 confidence score threshold). Again, the two discrepant genotypes, both of which were incorrectly called as homozygous, had high confidence scores, consistent with high quality APEX assay data. Separate analysis of homozygous and heterozygous cases showed that for a 0.0 threshold, homozygous cases (1289 in total) achieved a call rate of 100% with 100% HapMap concordance, whereas heterozygous cases (652 in total) achieved a call rate of 100% with 99.7% HapMap concordance (two heterozygous errors with high confidence scores). Surprisingly, with a 0.65 threshold, among 16 non-calls 14 were homozygous with 11 cases (all TT genotypes) from a single SNP rs1891403, which gives a homozygous call rate of 98.9% and a heterozygous call rate of 99.7%. Interestingly, the LDA-called genotype that had the lowest score (but nevertheless was still called correctly) was the same genotype as the third MACGT-called discordant genotype [see above and Additional file 7]. Subsequent inspection of the SNP Chart for this genotype (heterozygous CT) showed that the ASO-APEX probe intensity signals for the C allele were somewhat lower than the T allele signals. Again, this same sample/genotype had previously been concordant with HapMap in the initial data set, using the original PCR primer pairs. (See below for further discussion of this genotype and the other two discrepant genotypes.)

In summary, we have shown that a combination of multiplex PCR, redundant and robust APEX design and assay, and statistically-robust auto-calling (simple LDA using dynamic variable selection) can achieve 100% completion and call rate with >99.9% accuracy, for multiple SNPs and multiple samples. We believe that this is a significant improvement over other published APEX methodologies. The strength of our methodology is not based on the quality of a single measurement but on the redundancy obtained from measuring the allele intensities by using multiple chemistries. To take advantage of this inherent robustness of the assay we use robust statistical methods that automatically select the most reliable measurements for each SNP to make the genotype call, sample by sample [18]. Redundancy in genotyping arrays is associated with higher costs per SNP, concomitant with lower numbers of SNPs able to be interrogated in a given area of the microarray. For research studies, a trade-off may need to be taken into consideration, given the ever-increasing need to genotype as many SNPs as possible, at minimal cost per SNP, and a recent article by Smemo and Borevitz [29] cogently argues for a reduction in the approximately 40-fold probe redundancy currently featured on Affymetrix GeneChips, which only use hybridization for allelic signal generation. For clinical diagnostics however, we believe that genotyping accuracy, call rate and completion rate are paramount.

**Figure 3:**
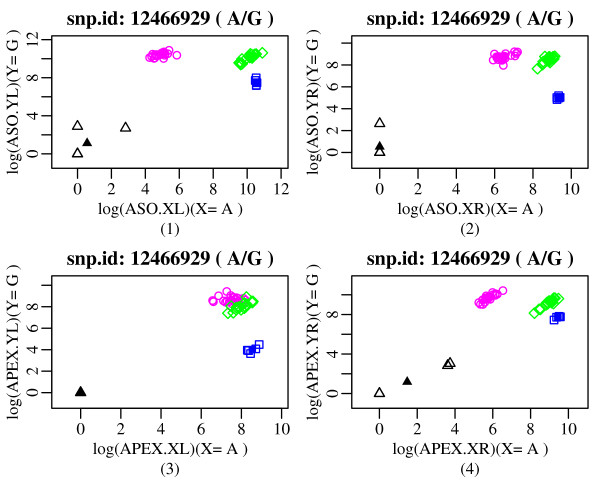
Simple scatter plots for SNP rs12466929 (A/G) from 50-plex data set (this SNP is representative of the entire set of 50 HapMap SNPs). For each plot the x-axis represents signal values for X allele (A for this SNP) and the y-axis represents signal values for Y allele (G for this SNP). All values are in log scale. Magenta, green, blue and black coloured symbols denote the classes YY (GG), YX (AG), XX (AA) and NN (negative control samples), respectively. Plot (**1**) combines the two ASO-APEX Left probes (one for each allele); plot (**2**) combines the two ASO-APEX Right probes (one for each allele); plot (**3**) is for the APEX Left probe; plot (**4**) is for the APEX Right probe. All the classifiers except APEX Left (plot **3**) give well separated genotype clusters for this SNP. Dynamic variable selection is able to automatically weight these LDA classifiers in such a way that the homozygous AA cluster in plot (**3**) (blue) is able to contribute to the final call for such genotypes, even though AG (green) and GG (magenta) genotype clusters overlap somewhat for this Left APEX probe. Additional file 11 shows four-panel scatter plots for all 50 SNPs from the 50-plex data set.

To further determine the effect of probe redundancy in our APEX methodology, we used LDA to reanalyze both data sets (original and 50-plex) but using non-redundant and partially-redundant probe-specific data [Additional files 8, 9 and 10]. Fig. 3 and Additional file 11 show simple four-panel scatter plots of the probe data for the 50-plex experiment. In particular, Fig. 3 represents the four separate scatter plots for the SNP rs12466929 corresponding to the four different probe chemistries: ASO.LEFT, ASO.RIGHT, APEX.LEFT and APEX.RIGHT. For each scatter plot, the three possible genotype clusters (previously known from the HapMap data set) are presented with three different colours: blue for allele 1 homozygous; magenta for allele 2 homozygous; and green for allele 1 and allele 2 heterozygous. For the SNP rs12466929, allele 1 is A and allele 2 is G, and the scatter plots are representative of the entire set of 50 HapMap SNPs. The four scatter plots indicate that three out of the four probe chemistries work perfectly well and produce well separable (informative) clusters corresponding to the three genotype classes (AA, AG and GG), whereas one probe chemistry, namely APEX.LEFT, fails to work properly and gives overlapping clusters for AG and GG genotype classes [plot (**3**) in Fig. 3]. Nevertheless, this probe chemistry gives a well separable cluster for the AA genotype class. This phenomenon conveys the point of considering each probe chemistry separately during the building of the genotype classification model, and in the next stage of the genotype calling algorithm, combining the four genotype models with proper weights adjusted dynamically with the quality of each of the four classifiers (four probe chemistries) specific to each SNP and sample. If all four probes failed to produce informative clusters, then our LDA-based genotype calling algorithm would flag that SNP as a failed SNP, which clearly is not the case for the SNP rs12466929. This is how the redundancy amongst our APEX based genotyping platform is captured through the proposed LDA-based genotype calling algorithm with dynamic variable selection. Viewing the four-panel scatter plots, we would also like to emphasize the point that for most of the SNPs the homozygous clusters show some significant signal intensities corresponding to the other allele, due to spectral overlap within the APEX fluorescent ddNTP chemistry, thus inducing background to the homozygous clusters. Particularly for this reason, we do not often see a homozygous cluster close to either of X- or Y-axes. Here, the aim is to compare the allele 1 and allele 2 signal intensities for the three possible genotype classes, and then assign a test sample to the appropriate class based on the prior knowledge of the available training set. We would also like to mention that the initial signal intensities corresponding to each allele for all four probe chemistries are converted into the log-scale in order to reduce the variability between several microarray slides.

Performance analyses for the different data sets are described below, addressing the redundant probe chemistry. The extreme left hand column of each table indicates the combination of four classifiers used to build the LDA model [Additional files 8, 9 and 10]. For example, in the first row, all four classifiers were used to give the final genotype call, and in the fourth row, only the left classifiers were used. In the last four rows, only one classifier was used at a time to give independent genotype calls using the simple LDA model (with no dynamic variable selection). For the complete set of 287 HapMap samples and the set of 41 SNPs, the training data had in total 807 genotype cases (among which 519 genotypes were from HapMap Coriell samples and 288 genotypes were from other Coriell samples) and the test data had in total 11,248 genotype cases (among which 163 had no validated genotypes from HapMap for comparison).

For the set of 270 HapMap DNA samples, applying a 0.65 threshold improved the concordance rate (0.31% miss-classification rate) with a reduced call rate of 97.30%. We further checked the performance of the same data set applying a stringent threshold of 0.75, which gave 99.91% concordance (0.06% miss-classification rate) for a reduced call rate of 94.91%. Applying different level of thresholds, we can control the call rates and, given the validated genotype set, we can also check the performance level by calculating the miss-classification rates. The underlying supposition is that, with reduced call rate, accuracy should increase successively until it reaches its maximum limit. For the improved 50-plex PCR chemistry, we were able to achieve a high concordance rate (99.89% using all four classifiers) with 100% call rate [Table 2]. If we apply a 0.65 threshold to the set of 50-plex PCR HapMap samples, then the automated call rate reduced to 99.18%, leaving only 16 non calls (below threshold value) to be verified manually using SNP Chart (all of which were correct).

Therefore, we have determined that reliance on any single probe type alone [*i.e*.: APEX Left probe; APEX Right probe; 2 × ASO-APEX Left probes (one for each allele); 2 × ASO-APEX Right probes (one for each allele)] resulted neither in as high an accuracy of genotyping nor in as high a call rate, compared to the dynamic use of multiple probes.

We were interested in further study of the two discrepant genotype cases, since both had previously been concordant with HapMap in the 7-reaction-multiplex PCR data set, and both showed high quality, unambiguous SNP Charts in the 50-plex PCR data set. A third genotype case (concordant with HapMap by manual calling and simple LDA, but with a low quality score of 0.4876) was also discrepant when called by MACGT. We re-amplified these three individual SNP loci from their respective Coriell DNA samples, using the original PCR primers [Additional file 1], and sequenced each amplicon from both ends. The two discrepant genotypes were: 1. DNA sample 192 (NA18502) at SNP rs3776720 – 50-plex genotype GG/HapMap & 7-reaction-multiplex genotype GA; 2. DNA sample 101 (NA18621) at SNP rs12472674 – 50-plex genotype CC/HapMap & 7-reaction-multiplex genotype CT. The third genotype case (concordant with HapMap by manual calling and simple LDA, but with a low quality score of 0.4876) was also discrepant when called by MACGT (DNA sample 228 (NA19210) at SNP rs4739199 – 50-plex genotype (MACGT) TT/HapMap, 7-reaction-multiplex, and 50-plex (manual call & LDA) genotype CT).

As expected, we identified additional polymorphic sites that coincided with the positions delimited by the PCR primer sequences used for the 50-plex reaction. One of the sites was identified as an existing SNP (rs6871885). To our knowledge, the other two sites represent genetic variants not previously reported. For each of these cases, it appears that the sequence variation within the PCR primer site has caused allelic drop-out, resulting in homozygous genotype calls for the two discrepant cases, and a poor quality heterozygous genotype call for the third case (partial allelic drop-out). Specifically, for discrepant genotype case 1 (Coriell NA18502 at SNP rs3776720), we found a neighbouring SNP (T/A) which is located at the 3' end of the anti-sense PCR primer site (5' CGA TGT AGG TGA CAC TAG TAT TGC AGG CAG ACG TG**A**3' – [Additional file 6]) – this polymorphic site (30 bp downstream of rs3776720) is reported in dbSNP as rs6871885, with the A base (sense strand) being described as a rare allele (0.083) in sub-Saharan African populations only (Coriell NA18502 is indeed a sub-Saharan African, Yoruba, and is heterozygote for this SNP).

For discrepant genotype case 2 (Coriell NA18621 at SNP rs12472674), we found a sequence variant (G/A) 52 bp downstream of SNP rs12472674, located within the anti-sense PCR primer site (5' CGA TGT AGG TGA CAC TAG CTC AAT ATG TTA **C**CA CAA 3' – [Additional file 6]) – this variant (heterozygous in Coriell NA18621 – Asian, Han Chinese) has not been previously reported in dbSNP and may represent a novel polymorphism. For the low quality genotype case 3 (Coriell NA19210 at SNP rs4739199), we found a sequence variant (G/A) 45 bp downstream of SNP rs4739199, located within the anti-sense PCR primer site (5' CGA TGT AGG TGA CAC TAG TC**C**ACT TCA TTA GGT GAA 3' – [Additional file 6]) – this variant (heterozygous in Coriell NA19210 – sub-Saharan African, Yoruba) has also not been previously reported in dbSNP and may represent a novel polymorphism.

Whilst more stringent due-diligence at the 50-plex PCR primer design stage would have alerted us to one of these SNPs (rs6871885), the evidence that we have identified two hitherto unreported SNPs provides a cautionary tale [30]. Elimination of such 'sporadic' genotyping errors due to novel or unaccounted-for SNPs, as well as due to structural variation in the genome (*e.g*., copy number variants – CNVs) [31], will need to be addressed in future clinical diagnostic genotyping technologies, and possibly even in research discovery studies where any sporadic errors due to hidden SNPs will not cause significant departure from Hardy-Weinberg equilibrium [15]. In preliminary studies we have been able to correct all three discrepancies previously described, using a redundant 50-plex PCR assay that includes two primer pairs for each SNP loci (data not shown).

Finally, due to the low amount (5 ng) of genomic DNA required for the 50-plex PCR (compared to 25 ng for each of the 7-reaction-multiplex PCRs), we have attempted APEX genotyping using our improved methodology on DNA derived from plasma samples. A pilot project was performed on five plasma samples (stored for up to ten years). Comparing the plasma-derived genotyping data with data obtained from high quality genomic DNA for the same five individuals, the call rate was >99% (100% for high quality DNA) and the concordance was >99%, which opens up the possibility of robust and accurate genotyping of clinical plasma samples without any need for prior whole genome amplification.

## Conclusion

We report significant improvements to arrayed primer extension (APEX) genotyping methodology that may show utility in future point-of-care genetic diagnostic applications. Our methods have been validated against industry-leading technologies in a blinded experiment based on Coriell DNA samples and SNP genotype data from the International HapMap Project. Modifications to PCR amplification design have allowed robust 50-plex genotyping from as little as 5 ng of DNA, with 100% call rate and >99.9% accuracy.

## Methods

### DNA Samples and Validated Genotypes

A set of 287 DNA samples were obtained from McGill University and Génome Québec Innovation Centre (one of the HapMap Project's genotyping centers). This set comprised 270 DNA samples from the Coriell Institute for Medical Research [22] plus hidden duplicates and negative controls, all of which our laboratory was blinded to. We were given access to the validated HapMap genotyping data for these samples only after we had finished the main genotyping experiment (287 samples/50 SNPs), and after we had sent a file of our genotyping results to McGill University.

### HapMap APEX Chip – Probe Design and Printing

Six oligonucleotide probes (25 mers) for each SNP were designed using Biodata algorithms (Biodata Ltd., Tartu, Estonia [32]) [Additional file 1]: two classical APEX probes (one probe per DNA strand), plus four allele-specific (ASO) APEX probes (two probes per strand) which include the actual SNP site at the 3' end of the probe. Allele-specific single base extension of these ASO-APEX probes during the reaction is contingent on the presence of the actual complementary base at the SNP site in the sample template DNA [6,10]. Probes were synthesized at a 25 nmol scale and aliquotted into 96-well plates by Integrated DNA Technologies (Coralville, IA, USA). We diluted each probe at 200 pmol/μL as stock concentration in pure water (resistivity of 18.2 MΩ-cm and total organic content of less than five parts per billion) using a Biomek FX robot (Beckman Coulter, Fullerton, CA, USA).

Arrays were generously printed for us at the Microarray Facility of The Prostate Centre at Vancouver General Hospital [33] (University of British Columbia, Vancouver, BC, Canada). Briefly, the APEX and ASO-APEX probe oligonucleotides (50 pmol/μL in 150 mM sodium phosphate printing buffer, pH 8.5) were printed to specific grid positions on CodeLink™ Activated Microarray Slides (Amersham Biosciences/GE Healthcare, Piscataway, NJ, USA) following the manufacturer's recommended protocols. The 5' end of each oligonucleotide probe was amino-modified during synthesis, allowing its covalent attachment to the slide's pre-applied surface chemistry. Each grid consisted of five spot replicates of each of the six probes per SNP, as well as multiple buffer-only spots and positive control normalization spots. The latter comprised an oligonucleotide probe based on a plant-specific gene sequence that will extend by a single N base due to the presence of an exogenous complementary template oligonucleotide in the APEX reaction mixture (Npg1) [14]. Each Npg1 positive control probe was spotted 40 times onto the grid, at regular physical intervals. Each one of the six probes for each SNP was printed at a reasonably wide distance apart from any other probe for the same SNP within the grid (as were their replicate spots). This enabled a useful degree of robustness in the system, especially helpful in cases of high local background and hybridization problems [14]. Each spot was approximately 110 μm in diameter. Three replicated grids were printed on each slide, enabling three samples to be genotyped per slide. Following the printing of the arrays, the slides were incubated overnight at room temperature at 75% relative humidity (saturated NaCl chamber) to drive the covalent coupling reaction between the probes' 5' amino group and the CodeLink™ slide chemistry to completion. Blocking of the arrays was in 50 mM ethanolamine, 0.1 M Tris, pH 9.0, 0.1% SDS, at 50°C for 20 min, according to the manufacturer's protocol.

### PCR Amplification and Fragmentation

For the first experiment, PCR primers were designed to amplify the regions across the 50 SNPs, based on a melting temperature (T_m_) of 62°C ± 3°C (at 20 mM monovalent salt concentration in PCR buffer [Additional file 1]). All primers were computationally tested against the human genome and found to amplify single product (Biodata Ltd., Tartu, Estonia [32]). Multiplex PCR amplifications were performed on the Coriell genomic DNA samples (plus several negative PCR control samples that contained no genomic DNA). The multiplex PCR group had a unique combination of the primer pairs among 7 reactions [Additional file 2]. Each PCR was performed in a total volume of 15 μL, containing 1.5 μL 10× PCR buffer [Tris-Cl, (NH_4_)_2_SO_4_, 15 mM MgCl_2_, pH 8.7], 1.5 mM MgCl_2_, 200 μM dNTPs without dTTP, 160 μM dTTP, 40 μM dUTP, 0.75 U HotStar *Taq *DNA polymerase (5 U/μL; Qiagen, Valencia, CA, USA), 1 μL 10 μM primer mixtures (each primer), and 25 ng genomic DNA. Incorporation of the dUTP allowed for the amplified DNA to be enzymatically sheared by uracil N-glycosylase (UNG, InterScience, Troy, NY, USA) to produce a DNA size of approximately 50–100 bases, optimal for hybridization to the oligonucleotides on the microarray (see below). Genomic DNA and PCR master mixture were transferred into ABI 384-well reaction plates (Applied Biosystems, Foster City, CA, USA) using a Biomek FX robot (Beckman Coulter, USA). PCR reactions were performed in a GeneAmp PCR System 9700 ThermoCycler (Applied Biosystems, USA). PCRs were initiated by a 15 min polymerase activation step at 95°C and completed by a final 10 min extension step at 72°C. The PCR cycles were as follows: 35 cycles of 30 s denaturation at 95°C, 30 s annealing at 58°C, and 50 s extension at 72°C.

For the second experiment, in order to increase the efficiency of PCR, we designed 50× 5' linker PCR primer pairs [Additional file 6] based on a T_m _of 65°C ± 7°C and performed 50-plex PCR in one single reaction per sample. Each new PCR primer had a common linker sequence designed at its 5' end (5' TACGACTCACTTAGGGAG-3' for each of the left hand PCR primers/5' CGATGTAGGTGACACTAG-3' for each of the right hand PCR primers). The 3' ends of the primers were chosen to have non-complementary bases with respect to each other (*i.e*., all primers ended with one or two A bases), in order to reduce the probability of primer interactions and primer-dimer formation. All primers were computationally tested against the human genome and found to amplify single product. The new amplicon sequences were located within the amplicon sequences from the original primer pairs. The multiplex PCR was carried out in a 25 μL reaction containing 20 nM (final) of each primer plus 20 nM of left and right linker-only primers (left linker: 5' TACGACTCACTTAGGGAG 3'/right linker: 5' CGATGTAGGTGACACTAG 3'), 200 μM dNTPs without dTTP, 160 μM dTTP, 40 μM dUTP, 6 units of HotStar *Taq *DNA polymerase (5 U/μL; Qiagen, USA), 1.5 mM MgCl_2 _in 1× PCR reaction buffer [100 mM Tris-HCl, 50 mM KCl, 100 μg/mL Gelatin, pH 8.3] with 5 ng of genomic DNA. PCR was performed using a MJR PTC 200 ThermoCycler (MJ Research, Waltham, MA, USA). PCR was initiated by a 15 min polymerase activation step at 95°C and completed by a final 3 min extension step at 72°C. The reaction procedure consisted of 40 cycles of denaturation at 95°C for 40 s, primer annealing at 55°C for 2 min and one ramping-up step from 55°C to 70°C for 2.5 min (0.1°C/s) [28].

Aliquots of PCR products were visualized with Gel Red fluorescent nucleic acid dye (Biotium, Hayward, CA, USA) staining under ultraviolet (UV) illumination on a 2% agarose gel, following electrophoresis in 0.5× Tris-borate EDTA (TBE) buffer. The 7 subgroup multiplex PCR products were pooled for each individual Coriell sample and precipitated by adding 2.5 volumes of ice-cold 100% ethanol and 0.25 volumes of 10 M ammonium acetate solution. After precipitation at -20°C overnight, the mixture was centrifuged at 20,800 × *g *at 4°C for 20 min. The supernatant was carefully removed, and the DNA pellet was washed with 400 μL of ice-cold 70% ethanol. The DNA pellet was then dissolved in 15 μL pure water. 10 μL of this DNA (or 10 μL of unpurified 50-plex PCR products; amplified to a concentration of approximately 300 – 400 ng/μL.) were then fragmented by 1 U uracil-N-glycosylase (UNG; Inter Science Inc., Troy, NY, USA) and unincorporated dNTPs were simultaneously inactivated by digestion with 1 U shrimp alkaline phosphatase (SAP; Amersham Biosciences/GE Healthcare, USA) for 15 min at 37°C, in a 20 μL reaction mixture containing 2 μL 10× digestion buffer [0.5 M Tris-HCl, 0.2 M (HN_4_)_2_SO_4_, pH9.0], followed by enzyme inactivation for 10 min at 95°C.

### Microarray-based Minisequencing: Arrayed Primer Extension (APEX)

The APEX reaction was performed in a total volume of 40 μL by the addition of 17 μL fragmented DNA template, 1 μL of 2 pmol/μL Npg1-positive control template oligonucleotide, 1.25 μM of each fluorescently labeled dideoxynucleotide triphosphate (Texas Red-ddATP, Cy3-ddCTP, Cy5-ddGTP, R110-ddUTP; Perkin Elmer Life Sciences, Boston, MA, USA), 5 U Thermo Sequenase™ DNA polymerase (Amersham Biosciences/GE Healthcare, USA) diluted in its dilution buffer, 2× Thermo Sequenase reaction buffer [10×, 260 mM Tris-HCl, 65 mM MgCl_2_, pH 9.5]. The reaction mixture was applied to the grid of APEX and ASO-APEX probes previously printed on the CodeLink slide that had been washed two times in 95°C pure water and placed on a Thermo Hybaid HyPro20 incubation plate (Thermo Electron, Waltham, MA, USA) set at 58°C. The reaction mixture was covered with a small piece of Parafilm™, and the APEX reaction allowed to proceed at 58°C with agitation (setting 1) for 20 min. Following the incubation period, slides were washed with 95°C water to remove the template DNA, enzyme, and excess ddNTPs. Further washing in 0.3% Alconox (Alconox Inc., White Plains, NY, USA) and 95°C pure water ensured low background on the array images.

### DNA Sequencing

As described in the main paper, we directly sequenced three SNP loci in three independent samples: 1. sample 192 (NA18502) at SNP rs3776720; 2. sample 101 (NA18621) at SNP rs12472674; 3. sample 228 (NA19210) at SNP rs4739199. We performed three single-plex PCR reactions using primer pairs from the first experimental design and methods [Additional file 1] to obtain the DNA fragments including the SNP sites on these three Coriell DNA samples. PCR primers pairs used were: 1. rs3776720 sense 5' GGC CAA GGA AAA GAA ATG AAT CTG CT 3', anti-sense 5' AAC TTT AGT GCA GGA TTT GCC ATC CA 3' – PCR amplicon size of 389 bp; 2. rs12472674 sense 5' TAA AAT CCA ATC AGG CCA ACT GTT CA 3', anti-sense 5' TCA ATG CCA TTA TAT GTG CCA GCC A 3' – PCR amplicon size of 388 bp; 3. rs4739199 sense >5' TCC AGC CAG CAA AAG ATC CTC AAA 3', anti-sense 5' TCA AGC ACA TGT TAC CAG TTT CCC AA 3' – PCR amplicon size of 587 bp. PCR products were purified using a QIAquick PCR Purification Kit (Qiagen, Valencia, CA, USA) according to the manufacture's instructions. DNA sequencing reactions were performed by the Nucleic Acid Protein Service Unit [34] at the University of British Columbia (Vancouver, BC, Canada). For each amplicon, sense and anti-sense PCR primers were used as sequencing primers.

### Microarray Imaging and Spot Intensity Calculation

Slide microarrays were imaged using an *arrayWoRx*^*e *^*Auto *Biochip Reader (Applied Precision, LLC, Issaquah, WA, USA), fitted with the following filter sets: 1. A488 – Ex. 480/15× – Em. 530/40 (R110 dye); 2. Cy3 (narrowband) – Ex. 546/11 – Em. HQ570/10 m (Cy3); 3. Texas Red – Ex. 602/13 – Em. 631/23 (Texas Red); 4. Cy5 – Ex. 635/20 – Em. 685/40 (Cy5) (Chroma Technology, Rockingham, VT, USA). Exposure times for each dye were set up to give approximately 60–70% pixel saturation for selected Npg1 positive control probe spots. Resolution of the imager was set to 10 μm. Four 16-bit TIFF files for each array were obtained (one from each channel) and these were imported into SNP Chart [35], a data management and visualization tool for array-based genotyping by primer extension from multiple probes [23]. This software generates visual patterns of spot intensity values, from multiple channels across a multiple probe set specific for a given SNP, allowing easy calling of the genotype. All the images were gridded in SNP Chart by manually selecting four pre-defined spots that, combined with knowledge of the layout of the grid, allows SNP Chart to locate every spot [23]. Spot segmentation and background subtraction were based on hybrid segmentation algorithms previously published by our laboratory [24,25]. Spot intensity values were normalized by setting the 40 Npg1 positive control spots, widely distributed across each array grid, to an average value of 20,000 units per channel, with the exported normalized intensity value calculated from the scale factor × median signal) [16].

Genotyping – Manual Calling

Manual genotype calling within SNP Chart was carried out as previously described [14,16,23].

### Genotyping – Automated Calling Using MACGT

The training set for MACGT (multi-dimensional automated clustering genotyping tool) [17] was selected by manually inspecting SNP Charts for each of the SNPs across some of the 287 samples. For the 50 SNPs, up to ten high-quality charts were chosen as 'prototypes' [23] for each genotype. All prototype data were exported from SNP Chart into a format readable by MACGT. MACGT was run on just the training data, and the clusters for each SNP were manually inspected to ensure there where no errors in the training set. Genotyping was performed by MACGT using the parameters NORMALIZE_GROUP_OF_4 = 1, GROUP_OF_4_MEAN_CUTOFF = 10, PATCH_GROUPS_OF_4 = 1, DROP_NNS = 1 [17]. A 'fit' statistical cut-off of 0.001 was used to identify poor quality genotypes as non-calls (NNs) [17]. Any SNP or sample with a high rate of NNs was subject to further inspection. We identified nine SNPs that the PCR assay performed poorly on and which MACGT could not confidently score, although manual inspection of SNP Charts did show that the assays were somewhat successful, albeit non-reproducibly. The final training set for the 41 SNPs was made up of 519 genotypes [Additional file 3]. All NNs were inspected within SNP Chart and manually called if possible. The final genotypes from MACGT and from those manually called were combined, and compared to the validated genotypes from HapMap using a Microsoft Excel macro [Additional file 3].

### Genotyping – Automated Calling Using Simple LDA with Dynamic Variable Selection

Detailed descriptions of the algorithms used in simple linear discriminant analysis (LDA) with dynamic variable selection have previously been published by our laboratory [18]. A brief descriptive example follows, using the data structure for SNP rs12466929 and DNA sample 101 (Coriell NA18621 – genotype AA [Additional file 12]).

Ideally, for variable construction, each genotype call could be based on just one of the four sets of probes: (1) APEX_LEFT; (2) APEX_RIGHT; (3) ASO_1LEFT and ASO_2LEFT; and (4) ASO_1RIGHT and ASO_2RIGHT [Additional file 12]. Considering the underlying chemistry, we have developed four sets of classifiers, named: APEX.L, APEX.R, ASO.L and ASO.R. Each of these classifiers consists of a pair of explanatory variables, generically denoted by X and Y, corresponding to two candidate alleles in the SNP position [Additional file 13]. In Additional file 12, for example, X and Y correspond to the A and G alleles, respectively. Since there are five realizations (replicates) for each of the two entries in each classifier, we summarized the information for each allele, by taking a robust average: median of the relevant signals from five spots, for each of the classifiers. From the example data in Additional file 12, the values of the variables for the classifier APEX.L are

APEX.XL = median (1394, 1148, 597, 1106, 1504) = 1148, and

APEX.YL = median (29, 27, 43, 27, 32) = 29, and so on, as summarized in Additional file 13. In our subsequent analyses, we have considered different combinations of the above mentioned classifiers.

Our automated genotype calling algorithm is based on the simple linear discriminant analysis (LDA), using dynamic variable selection as special criteria for various classifiers related to multiple probes. LDA is a supervised learning technique which requires a valid training set in order to build the classification (genotyping) model for each SNP. For the complete set of 287 HapMap samples, our dynamic variable LDA-based genotype calling algorithm used the same training set as used by MACGT above (*i.e*., 519 genotypes across the 41 SNPs [Additional file 3]) and predicted the genotypes for the remainder of the samples.

For LDA analysis of the 50-plex PCR chemistry, performed on a subset of 50 HapMap samples which were chosen randomly out of the original 287 samples, we selected prototypes to build a new training set using MACGT clusters, verifying the chosen cases with SNP Chart. We considered two different training sets, one with a small number of prototypes (at most 3 to 4 prototypes in each class) and the other with a minimal number of prototypes (at most 2 prototypes in each class) for each SNP. The two different training sets yielded different performances for the respective test data sets.

For automated genotype calling, we started our analysis by fitting the simple LDA-based genotype model using each classifier separately, and then comparing the predicted genotypes with the validated genotypes. Subsequently, we applied our dynamic-variable LDA-based genotyping model on different combinations of the four classifiers. When combining four classifiers together, for each SNP we apply LDA to each pair of variables in Additional file 13. For generic alleles X and Y, the possible classes are XX, XY, YY and NN (NN class corresponding to negative controls: generally low signal intensities for all channels throughout all probes). Bayesian posterior probabilities for the possible classes from each of the four possible classifiers are given in Table 3. The posterior probabilities for the four classifiers are combined using an entropy-based weighting scheme. For example, for the ASO.L classifier, define

**Table 3:** Bayesian posterior probabilities for the possible classes from each of the four possible classifiers

*LDA/Classes*	XX	XY	YY	NN
ASO.L	P_xx _^(*ASO.L*)^	P_xy _^(*ASO.L*)^	P_yy _^(*ASO.L*)^	P_NN _^(*ASO.L*)^
ASO.R	P_xx _^(*ASO.R*)^	P_xy _^(*ASO.R*)^	P_yy _^(*ASO.R*)^	P_NN _^(*ASO.R*)^
APEX.L	P_xx _^(*APEX.L*)^	P_xy _^(*APEX.L*)^	P_yy _^(*APEX.L*)^	P_NN _^(*APEX.L*)^
APEX.R	P_xx _^(*APEX.R*)^	P_xy _^(*APEX.R*)^	P_yy _^(*APEX.R*)^	P_NN _^(*APEX.R*)^

$${\text{E}}_{\text{ASO}\text{.L}}=-\text{[log(1/4)}+\text{(}-\sum_{\text{i}\in \text{C}}{\text{P}}_{\text{i}}^{\text{(ASO}\text{.L)}}{\text{log(P}}_{\text{i}}^{\text{(ASO}\text{.L)}}\text{)}\text{)]}$$

Analogous quantities are computed for other classifiers

E_ASO.R_, E_APEX.L _and E_APEX.R_

Proper weights are obtained by normalizing them, *e.g*.,

$${\text{W}}_{\text{ASO}\text{.L}}=\frac{{\text{E}}_{\text{ASO}\text{.L}}}{{\text{E}}_{\text{ASO}\text{.L}}+{\text{E}}_{\text{ASO}\text{.R}}+{\text{E}}_{\text{APEX}\text{.L}}+{\text{E}}_{\text{APEX}\text{.R}}}$$

The weights are applied to the posterior probabilities of the respective class to give the final class posterior probabilities. For example, the final posterior probability for XX class is

$${\text{P}}_{\text{XX}}={\text{W}}_{\text{ASO}\text{.L}}{\text{P}}_{\text{XX}}^{\text{ASO}\text{.L}}+{\text{W}}_{\text{ASO}\text{.R}}{\text{P}}_{\text{XX}}^{\text{ASO}\text{.R}}+{\text{W}}_{\text{APEX}\text{.L}}{\text{P}}_{\text{XX}}^{\text{APEX}\text{.L}}+{\text{W}}_{\text{APEX}\text{.R}}{\text{P}}_{\text{XX}}^{\text{APEX}\text{.R}}$$

After obtaining P_XY_, P_YY _and P_NN _in a similar manner, the final genotype call is obtained with highest weighted probability. In the last stage, call rate can be adjusted by applying varying thresholds to the 'final weighted probability' (confidence score), and the concordance with the validated genotype set will vary accordingly. The calls were checked for concordance with the validated genotypes from HapMap.

Additional file 7 contains all 50-plex HapMap genotyping data, for both MACGT and LDA.

## Competing interests

The author(s) declare that they have no competing interests.

## Authors' contributions

M.P. performed the linear discriminant analyses (LDA) using dynamic variable selection. J.R. performed the wet-lab experiments described in this study, and assisted in the design of the initial multiplex PCR. B.W.T. performed the image analysis and MACGT auto-calling and analysis steps, and assisted S.J.T. in the manual genotype calling. Z.E.C. helped design the 50-plex PCR primers, and undertook initial experimental evaluation of these primers. M.P., J.R., B.W.T. and S.J.T. discussed the results and contributed to the preparation of this manuscript. S.J.T. designed and supervised the experiments and analyses, and wrote the paper.

## Pre-publication history

The pre-publication history for this paper can be accessed here:



## Supplementary Material

Additional file 1 List of SNPs, probes and PCR primers. Table that details the rs numbers of the 50 SNPs investigated, as well as the APEX and allele-specific APEX probe sequences, and the PCR primer sequences for the initial experiment. Click here for file

Additional file 2 PCR multiplex groups. Table that details the 7 groups of multiplex PCRs. Click here for file

Additional file 3 Genotyping results from first experiment. Table that lists the complete genotyping results for 287 HapMap samples and 41 SNPs. Includes LDA call and MACGT call (with quality scores), as well as original HapMap call. Click here for file

Additional file 4 Chart interpretation is given below. Illustrative examples for each genotype case (TT, TC, CC) are shown for the SNP rs1433375. SNP Charts on the left hand side (samples 104, 148 and 67) represent auto-called genotypes, whilst those on the right hand side (samples 125, 128 and 126) represent manually-called genotypes. The y-axes (signal intensity) of each individual genotype class have been set to identical scale values for both auto- and manually-called samples, and clearly show that genotypes can be correctly called (at least by manual inspection of the data) from samples having signal intensities up to an order of magnitude lower than usual. Each chart shows four channel fluorescent intensity data (A,C,G and T) from thirty rs1433375-specific array spots (five replicate spots for six different probes – arranged along x-axis). Starting at the left hand side of each chart, the first five spots ('LEFT T/C') refer to the left-hand APEX probe that will give either a single T (blue) signal (for homozygous TT genotypes), or a C (green) signal (for homozygous CC genotypes), or a mixture of T and C (heterozygous CT). The next five spots ('RIGHT A/G') refer to the right-hand APEX probe that interrogates the complementary DNA strand nucleotide to that of the left-hand APEX probe, hence gives a single A (yellow) signal (for TT), a single G (red) signal (for CC), or a mixed A and G signal (for TC). The remaining spots represent allele-specific APEX probes in which a base-specific fluorescence signifies the presence of the allele. '_1' probes correspond to the first allele (T in the case of rs1433375), and '_2' probes correspond to the second allele (C). The redundancy and consistency of the data across different probes give high confidence in the assigned genotypes. Click here for file

Additional file 5 Re-scaled SNP Charts for rs1433375. This figure is a repeat of Additional file 4, except that the y-axes of the SNP Charts on the right hand side (manually-called samples) have been adjusted to show as much of the spot intensity data as possible. Note the relative increases in the background signals, as compared to the chart data on the left hand side (auto-called samples). Click here for file

Additional file 6 List of PCR primer sequences for 50-plex PCR experiment. PCR primer sequences that were designed for the 50 HapMap SNP loci, with amplicon sizes restricted to between 100 and 200 bp, and with common 5' linkers. Click here for file

Additional file 7 Genotyping results from second experiment (50-plex PCR). Table that lists the complete genotyping results for 49 HapMap samples and 50 SNPs. Includes LDA call and MACGT call (with quality scores), as well as original HapMap call. Click here for file

Additional file 8 Performance analyses for the different data sets, addressing the redundant probe chemistry. To further determine the effect of probe redundancy in our APEX methodology, we used LDA to reanalyze both data sets (original and 50-plex) but using non-redundant and partially-redundant probe-specific data. Three tables are shown (8, 9 and 10). Click here for file

Additional file 9 Performance analyses for the different data sets, addressing the redundant probe chemistry. To further determine the effect of probe redundancy in our APEX methodology, we used LDA to reanalyze both data sets (original and 50-plex) but using non-redundant and partially-redundant probe-specific data. Three tables are shown (8, 9 and 10). Click here for file

Additional file 10 Performance analyses for the different data sets, addressing the redundant probe chemistry. To further determine the effect of probe redundancy in our APEX methodology, we used LDA to reanalyze both data sets (original and 50-plex) but using non-redundant and partially-redundant probe-specific data. Three tables are shown (8, 9 and 10). Click here for file

Additional file 11 Simple scatter plots for all 50 SNPs from 50-plex data set. For each plot the x-axis represents signal values for X allele and the y-axis represents signal values for Y allele. All values are in log scale. Magenta, green, blue and black coloured symbols denote the classes YY, YX, XX and NN (negative control samples), respectively. Plot (**1**) combines the two ASO-APEX Left probes (one for each allele); plot (**2**) combines the two ASO-APEX Right probes (one for each allele); plot (**3**) is for the APEX Left probe; plot (**4**) is for the APEX Right probe. The plots for SNPs rs3776720, rs12472674 and rs4739199 include labeled data-points for the individual Coriell samples that gave rise to discrepancies in genotype calling. Click here for file

Additional file 12 Data structure for SNP rs12466929 & DNA sample HapMap 101 (Coriell NA18621 – AA). Illustrative table of microarray four-channel intensity data from 30 spots corresponding to one SNP (rs12466929) and one DNA sample. Click here for file

Additional file 13 List of explanatory variables listed by appropriate classifiers. Each of these classifiers consists of a pair of explanatory variables, generically denoted by X and Y, corresponding to two candidate alleles in the SNP position. Values are based on the data shown in Additional file 12. Click here for file
