# Supplementary material for: Robust SNP genotyping by multiplex PCR and arrayed primer extension
Source: BMC Med Genomics. 2008 Jan 31;1:5. doi: 10.1186/1755-8794-1-5 (PMC2266772; doi:10.1186/1755-8794-1-5)
Supplement: Additional file 1 — List of SNPs, probes and PCR primers. Table that details the rs numbers of the 50 SNPs investigated, as well as the APEX and allele-specific APEX probe sequences, and the PCR primer sequences for the initial experiment. [file 1755-8794-1-5-S1.pdf]

SNP	Probe ID	Probe Strand	Probe Sequence	APEX Signal	Allele
rs2134180	rs2134180	LEFT	ACCTCTGTAAGACAACCCCACTTCA	T/C	T/C
	rs2134180	RIGHT	GGAAGTGCCAGTTGTGGGTTGGGGA	A/G	A/G
	rs2134180_1	LEFT	CCTCTGTAAGACAACCCCACTTCAT	T/-	T/C
	rs2134180_1	RIGHT	GAAGTGCCAGTTGTGGGTTGGGGAA	T/-	A/G
	rs2134180_2	LEFT	CCTCTGTAAGACAACCCCACTTCAC	T/-	T/C
	rs2134180_2	RIGHT	GAAGTGCCAGTTGTGGGTTGGGGAG	T/-	A/G
rs2180289	rs2180289	LEFT	AATCCAAGCCTTGCCTGGGTCCCTT	C/G	C/G
	rs2180289	RIGHT	GTTCAAAGAGCTTAGGTGTGTAACT	G/C	G/C
	rs2180289_1	LEFT	ATCCAAGCCTTGCCTGGGTCCCTTC	A/-	C/G
	rs2180289_1	RIGHT	TTCAAAGAGCTTAGGTGTGTAACTG	A/-	G/C
	rs2180289_2	LEFT	ATCCAAGCCTTGCCTGGGTCCCTTG	A/-	C/G
	rs2180289_2	RIGHT	TTCAAAGAGCTTAGGTGTGTAACTC	A/-	G/C
rs1366660	rs1366660	LEFT	TTTGTCACTTACTCTGTGCTAGGGG	A/G	A/G
	rs1366660	RIGHT	GCTTTGCTGTCCTCACCTTACAACA	T/C	T/C
	rs1366660_1	LEFT	TTGTCACTTACTCTGTGCTAGGGGA	T/-	A/G
	rs1366660_1	RIGHT	CTTTGCTGTCCTCACCTTACAACAT	C/-	T/C
	rs1366660_2	LEFT	TTGTCACTTACTCTGTGCTAGGGGG	T/-	A/G
	rs1366660_2	RIGHT	CTTTGCTGTCCTCACCTTACAACAC	C/-	T/C
rs1825443	rs1825443	LEFT	GATTGCGTTAACTCTTTGTTGGACC	G/A	G/A
	rs1825443	RIGHT	AAATTTTGTGGGAAAAGTGTGACGA	C/T	C/T
	rs1825443_1	LEFT	ATTGCGTTAACTCTTTGTTGGACCG	T/-	G/A
	rs1825443_1	RIGHT	AATTTTGTGGGAAAAGTGTGACGAC	G/-	C/T
	rs1825443_2	LEFT	ATTGCGTTAACTCTTTGTTGGACCA	T/-	G/A
	rs1825443_2	RIGHT	AATTTTGTGGGAAAAGTGTGACGAT	G/-	C/T
rs8096868	rs8096868	LEFT	GGAAATGGATTTTTTAAAGAATGTG	C/T	C/T
	rs8096868	RIGHT	GAGCAAGTGGGACTCCTTCCTAGCC	G/A	G/A
	rs8096868_1	LEFT	GAAATGGATTTTTTAAAGAATGTGC	G/-	C/T
	rs8096868_1	RIGHT	AGCAAGTGGGACTCCTTCCTAGCCG	C/-	G/A
	rs8096868_2	LEFT	GAAATGGATTTTTTAAAGAATGTGT	G/-	C/T
	rs8096868_2	RIGHT	AGCAAGTGGGACTCCTTCCTAGCCA	C/-	G/A
rs12466929	rs12466929	LEFT	AGGATATCTAATCTTATTGTTTACC	A/G	A/G
	rs12466929	RIGHT	AGCTCAGTCCTAGAGTGCAGGTTGG	T/C	T/C
	rs12466929_1	LEFT	GGATATCTAATCTTATTGTTTACCA	C/-	A/G
	rs12466929_1	RIGHT	GCTCAGTCCTAGAGTGCAGGTTGGT	G/-	T/C
	rs12466929_2	LEFT	GGATATCTAATCTTATTGTTTACCG	C/-	A/G
	rs12466929_2	RIGHT	GCTCAGTCCTAGAGTGCAGGTTGGC	G/-	T/C
rs846752	rs846752	LEFT	AGAGCTTAAGATTGAGTGGCTATTC	C/G	C/G
	rs846752	RIGHT	GAACTAATAATCTGACTCACTGGGT	G/C	G/C
	rs846752_1	LEFT	GAGCTTAAGATTGAGTGGCTATTCC	A/-	C/G
	rs846752_1	RIGHT	AACTAATAATCTGACTCACTGGGTG	G/-	G/C
	rs846752_2	LEFT	GAGCTTAAGATTGAGTGGCTATTCG	A/-	C/G
	rs846752_2	RIGHT	AACTAATAATCTGACTCACTGGGTC	G/-	G/C

SNP	Probe ID	Probe Strand	Probe Sequence	APEX Signal	Allele
rs12472674	rs12472674	LEFT	ACATATTATACCCTTCGATTTTCAG	C/T	C/T
	rs12472674	RIGHT	TCTGCTGGCCTATAAAACACTGGTT	G/A	G/A
	rs12472674_1	LEFT	CATATTATACCCTTCGATTTTCAGC	A/-	C/T
	rs12472674_1	RIGHT	CTGCTGGCCTATAAAACACTGGTTG	C/-	G/A
	rs12472674_2	LEFT	CATATTATACCCTTCGATTTTCAGT	A/-	C/T
	rs12472674_2	RIGHT	CTGCTGGCCTATAAAACACTGGTTA	C/-	G/A
rs4606154	rs4606154	LEFT	AATTCCCTGTCCAGTCCTGCTGTAA	A/G	A/G
	rs4606154	RIGHT	GGCTACTGAGCTTTGGAACCATATG	T/C	T/C
	rs4606154_1	LEFT	ATTCCCTGTCCAGTCCTGCTGTAAA	C/-	A/G
	rs4606154_1	RIGHT	GCTACTGAGCTTTGGAACCATATGT	T/-	T/C
	rs4606154_2	LEFT	ATTCCCTGTCCAGTCCTGCTGTAAG	C/-	A/G
	rs4606154_2	RIGHT	GCTACTGAGCTTTGGAACCATATGC	T/-	T/C
rs7292634	rs7292634	LEFT	AGATTCACTATCTGTCTTTGTGCCA	C/T	C/T
	rs7292634	RIGHT	TCATCATTAGATTTAATAATGTTAA	G/A	G/A
	rs7292634_1	LEFT	GATTCACTATCTGTCTTTGTGCCAC	T/-	C/T
	rs7292634_1	RIGHT	CATCATTAGATTTAATAATGTTAAG	T/-	G/A
	rs7292634_2	LEFT	GATTCACTATCTGTCTTTGTGCCAT	T/-	C/T
	rs7292634_2	RIGHT	CATCATTAGATTTAATAATGTTAAA	T/-	G/A
rs273473	rs273473	LEFT	AAGGGTGGGAGAGGCGATTTCCCAA	A/G	A/G
	rs273473	RIGHT	TATTCTTATGGTATTAGTACCTCCT	T/C	T/C
	rs273473_1	LEFT	AGGGTGGGAGAGGCGATTTCCCAAA	A/-	A/G
	rs273473_1	RIGHT	ATTCTTATGGTATTAGTACCTCCTT	T/-	T/C
	rs273473_2	LEFT	AGGGTGGGAGAGGCGATTTCCCAAG	A/-	A/G
	rs273473_2	RIGHT	ATTCTTATGGTATTAGTACCTCCTC	T/-	T/C
rs2084851	rs2084851	LEFT	TGCTTCCCAGGGTTAGGCCCACACC	G/A	G/A
	rs2084851	RIGHT	ACATGGGCCAAGAAGTTACAAATCT	C/T	C/T
	rs2084851_1	LEFT	GCTTCCCAGGGTTAGGCCCACACCG	A/-	G/A
	rs2084851_1	RIGHT	CATGGGCCAAGAAGTTACAAATCTC	G/-	C/T
	rs2084851_2	LEFT	GCTTCCCAGGGTTAGGCCCACACCA	A/-	G/A
	rs2084851_2	RIGHT	CATGGGCCAAGAAGTTACAAATCTT	G/-	C/T
rs667415	rs667415	LEFT	GGAGAATTGACAGACTTTAAGGGAG	A/G	A/G
	rs667415	RIGHT	TGTCCAACCATTACTGTAGAACTAT	T/C	T/C
	rs667415_1	LEFT	GAGAATTGACAGACTTTAAGGGAGA	A/-	A/G
	rs667415_1	RIGHT	GTCCAACCATTACTGTAGAACTATT	C/-	T/C
	rs667415_2	LEFT	GAGAATTGACAGACTTTAAGGGAGG	A/-	A/G
	rs667415_2	RIGHT	GTCCAACCATTACTGTAGAACTATC	C/-	T/C
rs1486048	rs1486048	LEFT	TCGTGCAGGTCTCAGTGTGAATTGA	G/A	G/A
	rs1486048	RIGHT	TCACTCTTTTGCAATGTTTTGTTTC	C/T	C/T
	rs1486048_1	LEFT	CGTGCAGGTCTCAGTGTGAATTGAG	G/-	G/A
	rs1486048_1	RIGHT	CACTCTTTTGCAATGTTTTGTTTCC	T/-	C/T
	rs1486048_2	LEFT	CGTGCAGGTCTCAGTGTGAATTGAA	G/-	G/A
	rs1486048_2	RIGHT	CACTCTTTTGCAATGTTTTGTTTCT	T/-	C/T

SNP	Probe ID	Probe Strand	Probe Sequence	APEX Signal	Allele
rs2730648	rs2730648	LEFT	TTGATCCTTTCCCTTATCCTCATGT	T/C	T/C
	rs2730648	RIGHT	TAAAAATGTATAGGACTTGCTAAGG	A/G	A/G
	rs2730648_1	LEFT	TGATCCTTTCCCTTATCCTCATGTT	C/-	T/C
	rs2730648_1	RIGHT	AAAAATGTATAGGACTTGCTAAGGA	A/-	A/G
	rs2730648_2	LEFT	TGATCCTTTCCCTTATCCTCATGTC	C/-	T/C
	rs2730648_2	RIGHT	AAAAATGTATAGGACTTGCTAAGGG	A/-	A/G
rs1777467	rs1777467	LEFT	TTAGCATCTTTCAGTCATTCGGTCA	G/A	G/A
	rs1777467	RIGHT	GCATCAGTTAGTGTTTGTTGAGTGA	C/T	C/T
	rs1777467_1	LEFT	TAGCATCTTTCAGTCATTCGGTCAG	T/-	G/A
	rs1777467_1	RIGHT	CATCAGTTAGTGTTTGTTGAGTGAC	T/-	C/T
	rs1777467_2	LEFT	TAGCATCTTTCAGTCATTCGGTCAA	T/-	G/A
	rs1777467_2	RIGHT	CATCAGTTAGTGTTTGTTGAGTGAT	T/-	C/T
rs2835896	rs2835896	LEFT	TCCTCCACCTTGATGGTTCTTTTAG	C/T	C/T
	rs2835896	RIGHT	TATTTGAACGTGTGAGCTTGTCCTC	G/A	G/A
	rs2835896_1	LEFT	CCTCCACCTTGATGGTTCTTTTAGC	G/-	C/T
	rs2835896_1	RIGHT	ATTTGAACGTGTGAGCTTGTCCTCG	C/-	G/A
	rs2835896_2	LEFT	CCTCCACCTTGATGGTTCTTTTAGT	G/-	C/T
	rs2835896_2	RIGHT	ATTTGAACGTGTGAGCTTGTCCTCA	C/-	G/A
rs12583473	rs12583473	LEFT	AAAACATAGACATGAGCCCTTTTGG	C/G	C/G
	rs12583473	RIGHT	AGGTGGGAACAATCATGTACTCTAA	G/C	G/C
	rs12583473_1	LEFT	AAACATAGACATGAGCCCTTTTGGC	T/-	C/G
	rs12583473_1	RIGHT	GGTGGGAACAATCATGTACTCTAAG	C/-	G/C
	rs12583473_2	LEFT	AAACATAGACATGAGCCCTTTTGGG	T/-	C/G
	rs12583473_2	RIGHT	GGTGGGAACAATCATGTACTCTAAC	C/-	G/C
rs3899706	rs3899706	LEFT	GTATGTTAATGAGCAGGTCATCACT	C/G	C/G
	rs3899706	RIGHT	AAAGGGATGGAGCCCCAGTGGCCCA	G/C	G/C
	rs3899706_1	LEFT	TATGTTAATGAGCAGGTCATCACTC	T/-	C/G
	rs3899706_1	RIGHT	AAGGGATGGAGCCCCAGTGGCCCAG	A/-	G/C
	rs3899706_2	LEFT	TATGTTAATGAGCAGGTCATCACTG	T/-	C/G
	rs3899706_2	RIGHT	AAGGGATGGAGCCCCAGTGGCCCAC	A/-	G/C
rs4739199	rs4739199	LEFT	ACTTACAGGCCAAGATAGAGTGAGG	C/T	C/T
	rs4739199	RIGHT	ACACCCAACATTTCTTCTGGGGCAG	G/A	G/A
	rs4739199_1	LEFT	CTTACAGGCCAAGATAGAGTGAGGC	C/-	C/T
	rs4739199_1	RIGHT	CACCCAACATTTCTTCTGGGGCAGG	C/-	G/A
	rs4739199_2	LEFT	CTTACAGGCCAAGATAGAGTGAGGT	C/-	C/T
	rs4739199_2	RIGHT	CACCCAACATTTCTTCTGGGGCAGA	C/-	G/A
rs7855283	rs7855283	LEFT	AACCTCATATTTCACATTGTATCAC	A/G	A/G
	rs7855283	RIGHT	GGGATATCCCCACACAATATCCTAA	T/C	T/C
	rs7855283_1	LEFT	ACCTCATATTTCACATTGTATCACA	T/-	A/G
	rs7855283_1	RIGHT	GGATATCCCCACACAATATCCTAAT	G/-	T/C
	rs7855283_2	LEFT	ACCTCATATTTCACATTGTATCACG	T/-	A/G
	rs7855283_2	RIGHT	GGATATCCCCACACAATATCCTAAC	G/-	T/C

SNP	Probe ID	Probe Strand	Probe Sequence	APEX Signal	Allele
rs1607185	rs1607185	LEFT	CTCTGAATGCCAGGAGATCTGCCTC	G/A	G/A
	rs1607185	RIGHT	AAATAGGGACCACTCTGATCCATGC	C/T	C/T
	rs1607185_1	LEFT	TCTGAATGCCAGGAGATCTGCCTCG	G/-	G/A
	rs1607185_1	RIGHT	AATAGGGACCACTCTGATCCATGCC	G/-	C/T
	rs1607185_2	LEFT	TCTGAATGCCAGGAGATCTGCCTCA	G/-	G/A
	rs1607185_2	RIGHT	AATAGGGACCACTCTGATCCATGCT	G/-	C/T
rs4971653	rs4971653	LEFT	TTTGCAATGCAGCAATATCTTGTTT	A/G	A/G
	rs4971653	RIGHT	TTAAGAGGAAGATTTAATTTGCATT	T/C	T/C
	rs4971653_1	LEFT	TTGCAATGCAGCAATATCTTGTTTA	A/-	A/G
	rs4971653_1	RIGHT	TAAGAGGAAGATTTAATTTGCATTT	A/-	T/C
	rs4971653_2	LEFT	TTGCAATGCAGCAATATCTTGTTTG	A/-	A/G
	rs4971653_2	RIGHT	TAAGAGGAAGATTTAATTTGCATTC	A/-	T/C
rs2840794	rs2840794	LEFT	CATAGGTAGGTGGATGGAAAAATAC	A/G	A/G
	rs2840794	RIGHT	TTCCCTATGTGGGCTATTTGTGCCT	T/C	T/C
	rs2840794_1	LEFT	ATAGGTAGGTGGATGGAAAAATACA	A/-	A/G
	rs2840794_1	RIGHT	TCCCTATGTGGGCTATTTGTGCCTT	G/-	T/C
	rs2840794_2	LEFT	ATAGGTAGGTGGATGGAAAAATACG	A/-	A/G
	rs2840794_2	RIGHT	TCCCTATGTGGGCTATTTGTGCCTC	G/-	T/C
rs12426585	rs12426585	LEFT	TGTATGTGGGAAATCCTATGAAAAG	C/T	C/T
	rs12426585	RIGHT	AAAAATCCAGGTGGACAGAGACTTC	G/A	G/A
	rs12426585_1	LEFT	GTATGTGGGAAATCCTATGAAAAGC	G/-	C/T
	rs12426585_1	RIGHT	AAAATCCAGGTGGACAGAGACTTCG	C/-	G/A
	rs12426585_2	LEFT	GTATGTGGGAAATCCTATGAAAAGT	G/-	C/T
	rs12426585_2	RIGHT	AAAATCCAGGTGGACAGAGACTTCA	C/-	G/A
rs2938675	rs2938675	LEFT	AGCTCTCCACTTCCCTCACTCTGCA	C/A	C/A
	rs2938675	RIGHT	TGTGGAGCAGAGGAAAACCCAGGAG	G/T	G/T
	rs2938675_1	LEFT	GCTCTCCACTTCCCTCACTCTGCAC	C/-	C/A
	rs2938675_1	RIGHT	GTGGAGCAGAGGAAAACCCAGGAGG	T/-	G/T
	rs2938675_2	LEFT	GCTCTCCACTTCCCTCACTCTGCAA	C/-	C/A
	rs2938675_2	RIGHT	GTGGAGCAGAGGAAAACCCAGGAGT	T/-	G/T
rs3776720	rs3776720	LEFT	TTCTTCTGGTCACTAGCTGCATGTG	G/A	G/A
	rs3776720	RIGHT	CATTTATTTATGTAGATTACCACCC	C/T	C/T
	rs3776720_1	LEFT	TCTTCTGGTCACTAGCTGCATGTGG	G/-	G/A
	rs3776720_1	RIGHT	ATTTATTTATGTAGATTACCACCCC	C/-	C/T
	rs3776720_2	LEFT	TCTTCTGGTCACTAGCTGCATGTGA	G/-	G/A
	rs3776720_2	RIGHT	ATTTATTTATGTAGATTACCACCCT	C/-	C/T
rs4306755	rs4306755	LEFT	TATACACATGGCTGTAAATGTGTCC	A/G	A/G
	rs4306755	RIGHT	AACTGCCACTGCATGTTGAAGCACT	T/C	T/C
	rs4306755_1	LEFT	ATACACATGGCTGTAAATGTGTCCA	A/-	A/G
	rs4306755_1	RIGHT	ACTGCCACTGCATGTTGAAGCACTT	G/-	T/C
	rs4306755_2	LEFT	ATACACATGGCTGTAAATGTGTCCG	A/-	A/G
	rs4306755_2	RIGHT	ACTGCCACTGCATGTTGAAGCACTC	G/-	T/C

SNP	Probe ID	Probe Strand	Probe Sequence	APEX Signal	Allele
rs7693776	rs7693776	LEFT	CATTTTTACAAGTCTTCACTGCCTG	C/T	C/T
	rs7693776	RIGHT	CAGCTTATACAGTCTCTTATAGGAG	G/A	G/A
	rs7693776_1	LEFT	ATTTTTACAAGTCTTCACTGCCTGC	C/-	C/T
	rs7693776_1	RIGHT	AGCTTATACAGTCTCTTATAGGAGG	C/-	G/A
	rs7693776_2	LEFT	ATTTTTACAAGTCTTCACTGCCTGT	C/-	C/T
	rs7693776_2	RIGHT	AGCTTATACAGTCTCTTATAGGAGA	C/-	G/A
rs2760396	rs2760396	LEFT	ATGGCATGGGGGTGTTGTGTGTGGG	A/G	A/G
	rs2760396	RIGHT	GACCTCACAACCGCTTACTCCCCAC	T/C	T/C
	rs2760396m	RIGHT	GACCTTACAACCGCTTACTTCCCAC	T/C	
	rs2760396_1	LEFT	TGGCATGGGGGTGTTGTGTGTGGGA	G/-	A/G
	rs2760396_1	RIGHT	ACCTCACAACCGCTTACTCCCCACT	C/-	T/C
	rs2760396_1m	RIGHT	ACCTTACAACCGCTTACTTCCCACT	C/-	
	rs2760396_2	LEFT	TGGCATGGGGGTGTTGTGTGTGGGG	G/-	A/G
	rs2760396_2	RIGHT	ACCTCACAACCGCTTACTCCCCACC	C/-	T/C
	rs2760396_2m	RIGHT	ACCTTACAACCGCTTACTTCCCACC	C/-	
rs4933826	rs4933826	LEFT	AGACTTAATCTTTAAGGCAGAAGGA	C/G	C/G
	rs4933826	RIGHT	TTTCTCATAAAACCACCAGCCTGTC	G/C	G/C
	rs4933826_1	LEFT	GACTTAATCTTTAAGGCAGAAGGAC	G/-	C/G
	rs4933826_1	RIGHT	TTCTCATAAAACCACCAGCCTGTCG	T/-	G/C
	rs4933826_2	LEFT	GACTTAATCTTTAAGGCAGAAGGAG	G/-	C/G
	rs4933826_2	RIGHT	TTCTCATAAAACCACCAGCCTGTCC	T/-	G/C
rs592069	rs592069	LEFT	ATTATTCCTCCCCCTGCTTTCCTGC	G/A	G/A
	rs592069	RIGHT	AGGTTTTAAAGGATGAATAGGGAAG	C/T	C/T
	rs592069_1	LEFT	TTATTCCTCCCCCTGCTTTCCTGCG	C/-	G/A
	rs592069_1	RIGHT	GGTTTTAAAGGATGAATAGGGAAGC	G/-	C/T
	rs592069_2	LEFT	TTATTCCTCCCCCTGCTTTCCTGCA	C/-	G/A
	rs592069_2	RIGHT	GGTTTTAAAGGATGAATAGGGAAGT	G/-	C/T
rs2925067	rs2925067	LEFT	GAGCAGAGCCATCAGCATCCTTCTC	T/C	T/C
	rs2925067	RIGHT	CACATGCTGTAGTTAGAAAAGTCAA	A/G	A/G
	rs2925067_1	LEFT	AGCAGAGCCATCAGCATCCTTCTCT	T/-	T/C
	rs2925067_1	RIGHT	ACATGCTGTAGTTAGAAAAGTCAAA	G/-	A/G
	rs2925067_2	LEFT	AGCAGAGCCATCAGCATCCTTCTCC	T/-	T/C
	rs2925067_2	RIGHT	ACATGCTGTAGTTAGAAAAGTCAAG	G/-	A/G
rs2071748	rs2071748	LEFT	ACCTGAGCCAGACATCACCCTGGCT	A/G	A/G
	rs2071748	RIGHT	GCTATCTGTGAGACATGGAACACCG	T/C	T/C
	rs2071748_1	LEFT	CCTGAGCCAGACATCACCCTGGCTA	C/-	A/G
	rs2071748_1	RIGHT	CTATCTGTGAGACATGGAACACCGT	A/-	T/C
	rs2071748_2	LEFT	CCTGAGCCAGACATCACCCTGGCTG	C/-	A/G
	rs2071748_2	RIGHT	CTATCTGTGAGACATGGAACACCGC	A/-	T/C
rs7555995	rs7555995	LEFT	CCAAATACCTACCCTCTGAGAACGC	C/G	C/G
	rs7555995	RIGHT	GTGATTGCTTTTATAGGTTAGTTTG	G/C	G/C
	rs7555995_1	LEFT	CAAATACCTACCCTCTGAGAACGCC	C/-	C/G

SNP	Probe ID	Probe Strand	Probe Sequence	APEX Signal	Allele
	rs7555995_1	RIGHT	TGATTGCTTTTATAGGTTAGTTTGG	G/-	G/C
	rs7555995_2	LEFT	CAAATACCTACCCTCTGAGAACGCG	C/-	C/G
	rs7555995_2	RIGHT	TGATTGCTTTTATAGGTTAGTTTGC	G/-	G/C
rs4873622	rs4873622	LEFT	GAGATTCCCCCTTGAATGGCTTGGT	C/G	C/G
	rs4873622	RIGHT	AATTCACTCCTTGCTGCAGGGACAG	G/C	G/C
	rs4873622_1	LEFT	AGATTCCCCCTTGAATGGCTTGGTC	C/-	C/G
	rs4873622_1	RIGHT	ATTCACTCCTTGCTGCAGGGACAGG	A/-	G/C
	rs4873622_2	LEFT	AGATTCCCCCTTGAATGGCTTGGTG	C/-	C/G
	rs4873622_2	RIGHT	ATTCACTCCTTGCTGCAGGGACAGC	A/-	G/C
rs1891403	rs1891403	LEFT	TTCTCTCAGACTAACATCATCGACG	C/T	C/T
	rs1891403	RIGHT	GAGGCGCGCTGCCACAACTCCCCCT	G/A	G/A
	rs1891403_1	LEFT	TCTCTCAGACTAACATCATCGACGC	A/-	C/T
	rs1891403_1	RIGHT	AGGCGCGCTGCCACAACTCCCCCTG	C/-	G/A
	rs1891403_2	LEFT	TCTCTCAGACTAACATCATCGACGT	A/-	C/T
	rs1891403_2	RIGHT	AGGCGCGCTGCCACAACTCCCCCTA	C/-	G/A
rs2901585	rs2901585	LEFT	GCCACTCCTTCTTCTCTATTTGCTC	T/C	T/C
	rs2901585	RIGHT	GGAGTTTTGGAGACTCAAAGTTTCA	A/G	A/G
	rs2901585_1	LEFT	CCACTCCTTCTTCTCTATTTGCTCT	T/-	T/C
	rs2901585_1	RIGHT	GAGTTTTGGAGACTCAAAGTTTCAA	G/-	A/G
	rs2901585_2	LEFT	CCACTCCTTCTTCTCTATTTGCTCC	T/-	T/C
	rs2901585_2	RIGHT	GAGTTTTGGAGACTCAAAGTTTCAG	G/-	A/G
rs6478813	rs6478813	LEFT	AACTTTTTTCCTTTTTTCATTCACG	A/T	A/T
	rs6478813	RIGHT	CTGGCAAACAAAAAATACACCCAAG	T/A	T/A
	rs6478813_1	LEFT	ACTTTTTTCCTTTTTTCATTCACGA	C/-	A/T
	rs6478813_1	RIGHT	TGGCAAACAAAAAATACACCCAAGT	C/-	T/A
	rs6478813_2	LEFT	ACTTTTTTCCTTTTTTCATTCACGT	C/-	A/T
	rs6478813_2	RIGHT	TGGCAAACAAAAAATACACCCAAGA	C/-	T/A
rs318841	rs318841	LEFT	CCCTTGTTTATCAGTAGGTCAGTCT	C/G	C/G
	rs318841	RIGHT	GTGTACTTGATAGACACAGTTTTCA	G/C	G/C
	rs318841_1	LEFT	CCTTGTTTATCAGTAGGTCAGTCTC	T/-	C/G
	rs318841_1	RIGHT	TGTACTTGATAGACACAGTTTTCAG	A/-	G/C
	rs318841_2	LEFT	CCTTGTTTATCAGTAGGTCAGTCTG	T/-	C/G
	rs318841_2	RIGHT	TGTACTTGATAGACACAGTTTTCAC	A/-	G/C
rs1258464	rs1258464	LEFT	GCATCTGGACAAGTATATCAGGAAC	T/C	T/C
	rs1258464	RIGHT	CAGGGGTGGAGGGCCATATCCACAT	A/G	A/G
	rs1258464_1	LEFT	CATCTGGACAAGTATATCAGGAACT	A/-	T/C
	rs1258464_1	RIGHT	AGGGGTGGAGGGCCATATCCACATA	G/-	A/G
	rs1258464_2	LEFT	CATCTGGACAAGTATATCAGGAACC	A/-	T/C
	rs1258464_2	RIGHT	AGGGGTGGAGGGCCATATCCACATG	G/-	A/G
rs1433375	rs1433375	LEFT	GAAAGAGAGAATACCTTATTTCCTA	T/C	T/C
	rs1433375	RIGHT	TGTAAGCTTCTGATTCCCACAGAGA	A/G	A/G
	rs1433375_1	LEFT	AAAGAGAGAATACCTTATTTCCTAT	T/-	T/C

SNP	Probe ID	Probe Strand	Probe Sequence	APEX Signal	Allele
	rs1433375_1	RIGHT	GTAAGCTTCTGATTCCCACAGAGAA	T/-	A/G
	rs1433375_2	LEFT	AAAGAGAGAATACCTTATTTCCTAC	T/-	T/C
	rs1433375_2	RIGHT	GTAAGCTTCTGATTCCCACAGAGAG	T/-	A/G
rs2401810	rs2401810	LEFT	TGCTAAATTTCCTTCACCACCTCTA	T/C	T/C
	rs2401810	RIGHT	TTTTTGAGTCCCGAGTTAAATCTCA	A/G	A/G
	rs2401810_1	LEFT	GCTAAATTTCCTTCACCACCTCTAT	T/-	T/C
	rs2401810_1	RIGHT	TTTTGAGTCCCGAGTTAAATCTCAA	T/-	A/G
	rs2401810_2	LEFT	GCTAAATTTCCTTCACCACCTCTAC	T/-	T/C
	rs2401810_2	RIGHT	TTTTGAGTCCCGAGTTAAATCTCAG	T/-	A/G
rs803422	rs803422	LEFT	CTACCATGAGACAACGTTCCAGAGA	G/A	G/A
	rs803422	RIGHT	GCACCTGCTCTGGTTAGTCCCTCCT	C/T	C/T
	rs803422_1	LEFT	TACCATGAGACAACGTTCCAGAGAG	A/-	G/A
	rs803422_1	RIGHT	CACCTGCTCTGGTTAGTCCCTCCTC	T/-	C/T
	rs803422_2	LEFT	TACCATGAGACAACGTTCCAGAGAA	A/-	G/A
	rs803422_2	RIGHT	CACCTGCTCTGGTTAGTCCCTCCTT	T/-	C/T
rs2803543	rs2803543	LEFT	ACATAAGATGATCCTGGAGTACCTT	A/G	A/G
	rs2803543	RIGHT	AGTACTCCCTTACTTTCTGACACTA	T/C	T/C
	rs2803543_1	LEFT	CATAAGATGATCCTGGAGTACCTTA	T/-	A/G
	rs2803543_1	RIGHT	GTACTCCCTTACTTTCTGACACTAT	A/-	T/C
	rs2803543_2	LEFT	CATAAGATGATCCTGGAGTACCTTG	T/-	A/G
	rs2803543_2	RIGHT	GTACTCCCTTACTTTCTGACACTAC	A/-	T/C
rs6068122	rs6068122	LEFT	CACCTGGGGGAAAAACTAACAAAAG	A/T	A/T
	rs6068122	RIGHT	TTTTTCTCTGTGGATATCTTACACA	T/A	T/A
	rs6068122_1	LEFT	ACCTGGGGGAAAAACTAACAAAAGA	T/-	A/T
	rs6068122_1	RIGHT	TTTTCTCTGTGGATATCTTACACAT	C/-	T/A
	rs6068122_2	LEFT	ACCTGGGGGAAAAACTAACAAAAGT	T/-	A/T
	rs6068122_2	RIGHT	TTTTCTCTGTGGATATCTTACACAA	C/-	T/A
rs365063	rs365063	LEFT	TTGGGGATGGGGTCCCTGTGACCAC	A/G	A/G
	rs365063	RIGHT	CCGAAGAAGCTGGTACTTCCCACAC	T/C	T/C
	rs365063_1	LEFT	TGGGGATGGGGTCCCTGTGACCACA	G/-	A/G
	rs365063_1	RIGHT	CGAAGAAGCTGGTACTTCCCACACT	G/-	T/C
	rs365063_2	LEFT	TGGGGATGGGGTCCCTGTGACCACG	G/-	A/G
	rs365063_2	RIGHT	CGAAGAAGCTGGTACTTCCCACACC	G/-	T/C
rs2156208	rs2156208	LEFT	ACAAATATGCAAATAGTGAAGCGTG	C/T	C/T
	rs2156208	RIGHT	AGAGCATCCTAGGGTTCTCCTATGC	G/A	G/A
	rs2156208_1	LEFT	CAAATATGCAAATAGTGAAGCGTGC	G/-	C/T
	rs2156208_1	RIGHT	GAGCATCCTAGGGTTCTCCTATGCG	C/-	G/A
	rs2156208_2	LEFT	CAAATATGCAAATAGTGAAGCGTGT	G/-	C/T
	rs2156208_2	RIGHT	GAGCATCCTAGGGTTCTCCTATGCA	C/-	G/A
rs1347423	rs1347423	LEFT	ATCTCAAGTTGTCCAATGCCCATCT	C/A	C/A
	rs1347423	RIGHT	CAGAGTTATAACATCTGACAATTGG	G/T	G/T
	rs1347423_1	LEFT	TCTCAAGTTGTCCAATGCCCATCTC	C/-	C/A

SNP	Probe ID	Probe Strand	Probe Sequence	APEX Signal	Allele
	rs1347423_1	RIGHT	AGAGTTATAACATCTGACAATTGGG	A/-	G/T
	rs1347423_2	LEFT	TCTCAAGTTGTCCAATGCCCATCTA	C/-	C/A
	rs1347423_2	RIGHT	AGAGTTATAACATCTGACAATTGGT	A/-	G/T
rs1560434	rs1560434	LEFT	GGAATATACTAAAGGCAATCAGTGA	C/T	C/T
	rs1560434	RIGHT	CTGCACTCATCACTACCAGCTTTAC	G/A	G/A
	rs1560434_1	LEFT	GAATATACTAAAGGCAATCAGTGAC	G/-	C/T
	rs1560434_1	RIGHT	TGCACTCATCACTACCAGCTTTACG	T/-	G/A
	rs1560434_2	LEFT	GAATATACTAAAGGCAATCAGTGAT	G/-	C/T
	rs1560434_2	RIGHT	TGCACTCATCACTACCAGCTTTACA	T/-	G/A

SNP	PCR Left Primer 5'-3'	PCR Right Primer 5'-3'	PCR size (bp)	PCR sequence
rs2134180	TGGAAATCACAGCTTTCCTGAATCAA	AAATCTCCAGCGAATGTGACAAACCA	556	TGGAAATCACAGCTTTC
rs2180289	TGCTGATATTGAATTGGGAAGCCAAA	TCAAATCACAATCCACATTCCGTCA	551	TGCTGATATTGAATTGG
rs1366660	TCACCATCGCTCATGAATTTTGGAA	TGTTTTGCTTCCAAAGGAGCTCTGAA	315	TCACCATCGCTCATGAA
rs1825443	TGATTATGTTGTATGCATGGGCAGGA	TGGACGTTTCGTTCAAACACAAATGA	486	TGATTATGTTGTATGCA
rs8096868	TGAAATGCCTGCCTTTTGTTAGAGCA	TGCATTACTTTCCTGCAAACAACACA	785	TGAAATGCCTGCCTTTT
rs12466929	AATGGAAGTTTCCACTGGCTGCAAAT	CCATGCTTGCTTGTTTATCCTCCAAA	550	AATGGAAGTTTCCACTG
rs846752	AGGGGACATTGAGCTGCACAAAGATT	AGGGCTGATTACAATGCAATTTCTGC	646	AGGGGACATTGAGCTG

SNP	PCR Left Primer 5'-3'	PCR Right Primer 5'-3'	PCR size (bp)	PCR sequence
rs12472674	TAAAATCCAATCAGGCCAACTGTTCA	TCAATGCCATTATATGTGCCAGCCA	388	TAAAATCCAATCAGGCC
rs4606154	TCAAAATGCTTGGTGTCTTCTTTGC	TTAAAATATTTGGATCAGGGCAGCCA	432	TCAAAATGCTTGGTGTC
rs7292634	TAAAGAATCCCACCCTTAATTTGCCA	ATGCAGGCCATGTGAATTTGTTATTG	295	TAAAGAATCCCACCCTT
rs273473	ACACATGCAATTGAAAATCCAGGCAT	ATCAGGAACAAACAGGGTCTTCAGGG	707	ACACATGCAATTGAAAA
rs2084851	ACATTGCATGCTGAGTGTTGCAGTTT	TGTATGCCCTGTCATCTGAACCAAAA	216	ACATTGCATGCTGAGTC
rs667415	TTTTGAATCTGTGGTTGGCTGAATCT	CCCCTGTGATTTCTTTAGCCCATTG	717	TTTTGAATCTGTGGTTG
rs1486048	TGCCAAGTTATTTTGAGGAACGGATT	AGCCTTTTGCTTCAAAGGAAGCTCAT	454	TGCCAAGTTATTTTGAG

SNP	PCR Left Primer 5'-3'	PCR Right Primer 5'-3'	PCR size (bp)	PCR sequence
rs2730648	CAAATGGCAACACCATCCAACAAAAT	TTTGTTGCTGGGTCAAATTCTCATTG	521	CAAATGGCAACACCAT
rs1777467	CGTGTTAAGGCCATACCCCAAAGAAA	CAATCATGGATTGAAAATCTTGGCCT	485	CGTGTTAAGGCCATAC
rs2835896	TAACAGTTTTGTGTTGTGCCAGCCA	TGCACAATTCAGTCCATGACATTACG	685	TAACAGTTTTGTGTTGT
rs12583473	ATTCAACTTTGGTGCCTGTGCTTTGA	ACATCAGAATTGGCCACCCAAAAGA	502	ATTCAACTTTGGTGCCT
rs3899706	TTTGACATGTGAATATGGAGGGGACA	TTGTTATTCCCACGGGAGGAAATGTT	460	TTTGACATGTGAATATG
rs4739199	TCCAGCCAGCAAAAGATCCTCAAA	TCAAGCACATGTTACCAGTTTCCCAA	587	TCCAGCCAGCAAAAGA
rs7855283	TGATGCTTGTGTTGTCTCTGTCACCA	AATGCAAATGGGAGAATAATGAGCAA	605	TGATGCTTGTGTTGTCT

SNP	PCR Left Primer 5'-3'	PCR Right Primer 5'-3'	PCR size (bp)	PCR sequence
rs1607185	AATTCAATGAACAGCCACGAAATGCT	TTTTCTGGTCCATTCTCAGGCAAAA	279	AATTCAATGAACAGCCA
rs4971653	GGCCTCTGAGCTATGGTCCAGGATTA	TGCAATGCATGCTTGTAATGTTTGC	388	GGCCTCTGAGCTATGG
rs2840794	CAAGTAGCCAAAGGGAAGGATTTGAA	TCACATTGTGGCCAGATTATTTGCTT	618	CAAGTAGCCAAAGGGA
rs12426585	TTTGTCACTTCATTGGCACTGCATTA	TCCAAGATTCAAATTCAGGCCCCTT	607	TTTGTCACTTCATTGGC
rs2938675	CATTGGTTTATGCTGCTTCCCATTCA	TTGTGGGTGGAACCTAAATTGCTTTG	704	CATTGGTTTATGCTGCT
rs3776720	GGCCAAGGAAAAGAAATGAATCTGCT	AACTTTAGTGCAGGATTTGCCATCCA	389	GGCCAAGGAAAAGAAA
rs4306755	GCATTGCAGCACTTCTCCATTGTTTT	TTGAAATTGCACTCAAAGAAACCGA	787	GCATTGCAGCACTTCT

SNP	PCR Left Primer 5'-3'	PCR Right Primer 5'-3'	PCR size (bp)	PCR sequence
rs7693776	TTCAGGTTCAATGTTTTCAAGGACCA	TGAACACTTGTTGAGGACCGGGTAA	552	TTCAGGTTCAATGTTTT
rs2760396	TTTGGATCAGTGATTGGAGAGCAAAA	AATGCCTCATTTATCACCAGCATGGA	392	TTTGGATCAGTGATTGG
rs4933826	TTTGGTGTTATCATTGCCTTCCAATC	TGGCAAAGTGGGGCAACAAATTAAA	736	TTTGGTGTTATCATTGC
rs592069	TGAATTCATCGTACAGTTTCATGCCG	ATGATGGTTTTGGTGGAAATGAAGCA	493	TGAATTCATCGTACAGT
rs2925067	AAATGGCAGAAATGGAGGCAAATTC	AAAAGCTCAAATGCATCCTTTCCCAT	585	AAATGGCAGAAATGGA
rs2071748	TGTGTTTGGAGTTGCTCTCTGCTGAA	TCATCATGAAGGCGTCAGGGTTAAAA	542	TGTGTTTGGAGTTGCT
rs7555995	TCAACAACAAATGTCCCAACATCTCA	AGATGGTGGCTGCAACTGGAAATG	205	TCAACAACAAATGTCC

SNP	PCR Left Primer 5'-3'	PCR Right Primer 5'-3'	PCR size (bp)	PCR sequence
rs4873622	ATTCTGCTGCCAATTTGGTTCTCATT	TATTTGGCTCATGGCTCTGCAGGTT	483	ATTCTGCTGCCAATTTG
rs1891403	TGTGCCTGGCTAATCTTTTCATTCCA	TTGCCATCATTCAGCCAGTTCCTAAA	685	TGTGCCTGGCTAATCTT
rs2901585	TCACCATCAGCATCCAAACCTTCA	AAGATTGAGGGTCACATTTGAAGCAA	558	TCACCATCAGCATCCAA
rs6478813	CAAAACCCTGTTGTTGCTTTTGGTTA	GCAACTGGATGAATGCCGGCTAATA	419	CAAAACCCTGTTGTTGC
rs318841	ACAAAGCCAAATATTAGTGCCTGCAA	TTCCCGAGGTTTCACAATTCATTCA	563	ACAAAGCCAAATATTAG
rs1258464	TTGGATGATGGCATGGAGTATGGTAA	TTTGGTGGATCACTGTCTTGTCCCA	333	TTGGATGATGGCATGGA
rs1433375	TTCAGCTAACGACCACCAGGAAAACA	TGCACCAGATGGAATTAAACAGGCA	580	TTCAGCTAACGACCACC

SNP	PCR Left Primer 5'-3'	PCR Right Primer 5'-3'	PCR size (bp)	PCR sequence
rs2401810	TTTTCAGGGCTGCTCCAAGTGTTTT	AATCCAGCTTCATAAATCGCATGGTT	572	TTTTCAGGGCTGCTCCA
rs803422	AATATTGGCCAAGGGAGTGAAACCA	AAGCCTGGAAAAGATGCTCAGAAACA	550	AATATTGGCCAAGGGAG
rs2803543	AGGGCCCCTAGGAGAAACTGCTGAT	TTAAATTGTTCCTGCTTTGGCCACT	511	AGGGCCCCTAGGAGAA
rs6068122	TTGGCCTTTGTCCAGCATCCAA	ACACAAGTGTTGGAATTAGGCAGAGC	383	TTGGCCTTTGTCCAGCA
rs365063	TTGATATACGGAAAAGGGCGACATCA	TGGGATGGATACAAGGTGAGGCTTTT	159	TTGATATACGGAAAAGG
rs2156208	TCCATTGCCATCATCCTAATCGAA	ATATCTTGGGCTTCATGTTCGCTCTT	684	TCCATTGCCATCATCCT
rs1347423	TTTCATCTTTCTTTAATCCGGCACCA	AATTAATCTGTGCAAAGGCAGCATGA	307	TTTCATCTTTCTTTAATC

SNP	PCR Left Primer 5'-3'	PCR Right Primer 5'-3'	PCR size (bp)	PCR sequence
rs1560434	AAAATAACCATGCCCCTTTGCCATTT	TTTTCCCTTGTCCTCATTCTCCATCA	502	AAAATAACCATGCCCCT

SNP												
rs2134180	CTGAATCAAATTAGTTGACTCTGAATTCTAGTATCTGAAGTCTGCGTGTTTGAAAAGTAACTTCTAGAGGCTGCCAGGACCCCTGATA											
rs2180289	GAAGCCAAAACTTAGGTTTTGAATCCAAGCCTTGCCTGGGTCCCTTCAGTTACACACCTAAGCTCTTTGAACAATAATTTATTGAACTG											
rs1366660	TTTTGGAAGGCCTTGGCTCACATCAGGAACTCATTTCACCACTGTCCCCCCAGTAATGAGTGTTGAATTAATGGTTCTTCCCAGATGT											
rs1825443	TGGGCAGGAGGTCAGGCGGGTCGGGCCTTTCTGCTCCAGACCATCTCTGCATGAGCCTTTCGCCTTCAGATCTTGCTGAAACTTGCC											
rs8096868	GTTAGAGCATCATTAGACAAGTTTTTATGTGAATATCCAATCACAACTCAGTGAACAGCATTTTGAGAACAGGGATTTGTTGTTAGAAT											
rs12466929	GCTGCAAATCTGGACTCATTACTCTGTTTTGTGGGTTTCACCCTCCACGTACAGGCTCATGTATGAAAGAAATATTGCATCTCTAGTTT											
rs846752	CACAAAGATTATAGTTTACCTGTAATCAAGGGCAGCTATTGTTAGCACAAGATTTAAAAACAAGTAGATAGAGAGTCAGATAATACTTG											

SNP												
rs12472674	AACTGTTCAGATTTTTGTTAGATATGAAATACATGAATGGGTTCAGAGGCTTCGCTGTGTATTGAAAGAACATATCTGACTTTACGGCA											
rs4606154	TTCTTTGCAGAAATTGGCAAACTGATTCTAAACTTCATGTAGATGTGGAAGAAATCCAGAATAGTAAAAACAATCTTGAAAGAGAATAA											
rs7292634	AATTTGCCAGGATATGTAAAGTAGTAGGTACTTGCTATTGTCTTACGGCCTCTTACGTGAAATGTCAGATTCACTATCTGTCTTTGTGC											
rs273473	TCCAGGCATGACTGAATACAGGGCTCACATGAGTTTATATCAGGACTCAGTGCTTTTGTTGTATCCTTCATTATGAGTTGGCCTCACTC											
rs2084851	TTGCAGTTTCTGAATGAGTGTAGGTTGGGAGAACATGATGACAATCAGAGGGGCAGAAAGCTTGCTCTGATGAGTGGCAGTGTCTAT											
rs667415	GCTGAATCTGCAGATATGGAACCTGTGGATATGGAGGGCTGACTGTATAAGGTATCCTCAAATAAGATTAACAGCTGTTTTTCATCAG											
rs1486048	GAACGGATTGATGATTAATAGCAGAACTTCTGGGTGAGTCTTTAGGAAGAAAAAACCCTGTGTCCAGGCATTTGTCTCAGTGCCATAT											

**SNP**												
rs2730648	CCAACAAAATATTCAAACCAGAACTATTGAAGGCATCCTAGATTGATCCTTTCCCTTATCCTCATGTCCCTTAGCAAGTCCTATACATTTT											
rs1777467	CCCAAAGAAAGGGAGCTAGATGCCTTCTCTGGCTTCCTCTTTTCTCAACAGCCACGCCTCCGCCTACCAGCCAGGTTCTGCAGTGGGT											
rs2835896	GCCAGCCACCGGGCAAATGGTGCCTTCCTGCCAGAGGGGCGTGGGAAGTAAGAAAGTGCTGAGAAGGTTCTGGAGGGAGAAAGGC[illegible]											
rs12583473	GTGCTTTGAGAATGTAGTATTTGAGGTCACATTTACCATCCAAAAATTTCCATACATTTGAGGGACCACATACTCTCTATAGGTGTTTG[illegible]											
rs3899706	GAGGGGACACAGACATTCAGATCATAGCACAAAGATAATGGAAAAGTGAGACAAGGAGAAAAAAAGGGAGCTGACACAGGTATGTTA[illegible]											
rs4739199	TCCTCAAAGTAAGAAATGGCCTCAGAATAAAGATCAAGAGTACGGCCATAACACCATGGATTAGGAACTCAAAAAGATTTAAGACAATG											
rs7855283	CTGTCACCAAACAAAAAGGACTGTTGACTTGGTTTCAAAGTTTCAAAAGTTAAGAGGCTCATTTTCTGTAACCTCATATTTCACATTGTA											

**SNP**												
rs1607185	CGAAATGCTCTTAAGTATGACATACGGAGTTGAGAGGGTCCTGCTGCACCACAATCTCTGCACAGAAAGAGTGGGATGGTTCAGGCT											
rs4971653	TCCAGGATTACATGCATAGTAGACTTCTGAAGAAAGACAACCATGAGTTCAGTTTATTTCTCCACTAGAAGGACTGGATTTTAGAGATC											
rs2840794	AGGATTTGAACTTCATTACATTTTATCTCTCCTGCCATGCTCTGCCTTCCACTCTACAGAAAAGGGACAGGGAAATAGTCCAATCATAG											
rs12426585	ACTGCATTATAGAAACTAAGTGTAATTGCGACCTAGAAAGGTTCAACAGTGAAACCAAAAAAAGAATAAAAGTAAAGGAACCTCGGCTT											
rs2938675	TCCCATTCACTCGGGTTGTTTACTCTAATTTCAGGCAAGTTTGACTTATCTCAACAAGTCTTGCTATGGCCTAGGAGTTAAACAGCAAC											
rs3776720	TGAATCTGCTACAGATTCTCTCCCCTGACTATTGTCCAACAAAAAACAAAAACACCAACTCTATGTTAGAAGTTTTCAATCTTTTGAAAA											
rs4306755	CATTGTTTTATTTCACGCATATTGGACTCAGGGTAGAGACCATATCTTAAACGTCTTTGCTTTGAGCAAGATATTTAAACTCTCTGTGC											

**SNP**												
rs7693776	CAAGGACCATAACGGGGGAAGGTGTGAGCGCTCGTGAGATTGTGGAGAGCTGAGGAACCAGTGCTGTGTTGACCGAGGGTTCTTTT											
rs2760396	AGAGCAAAAGATAAGTATGCCCAATTGGTCTTTCCATTTCCTTTACTGACTTACTGACTTATATACCTACTTGTGTGTCCTACAAGATTC											
rs4933826	CTTCCAATCTCGTCTATCTCAAATGCTGTATCAATGTTGACTCTGACAATAGTAAGGGAAGGCTGGACAATCTGGAAGGATGGGTAAG											
rs592069	TTCATGCCGCTCCACCTTTAAACATGTTTTCCCTCTGCTTAGAGGAATTATTCCTCCCCCTGCTTTCCTGCGCTTCCCTATTCATCCTTT											
rs2925067	GGCAAATTCTGTCAAAGCTTTGATTTGCCCTCAGCAAAGTTAGACACATGTTTCTGTCTGCACAGCACATATTTCACCCTTTCTTCTGAT											
rs2071748	TCTGCTGAATCTAGATCTCTGGGGCTCTGCCAGCCTGGGGGAGCATGCTTGGTTCTCTTGGTGGCATCTGTCCCTCACTAGCTACGG											
rs7555995	AACATCTCAGTCTGCTTCCTGGGGACATGTGACAGTGGCTCTCTCCAAATACCTACCCTCTGAGAACGCCCAAACTAACCTATAAAAG											

SNP												
rs4873622	GTTCTCATTCCCCCAGCTTCTATCTGAATGCTGTTAACAGTTTTATTTGTCCTTTGGGTTCTTTTATTTTATCAGGATGTGCACAGGTGT											
rs1891403	TTCATTCCAATCAAGTGTTTTAAGCGGGGGAGGAACAGGGCCGTGTTTGGGGTTAGAGTCACTGATCAAGAACTAGCTGGGATTGGG											
rs2901585	ACCTTCACCTCTCACCTTCTTAAAATTATCTCTTCCAAGCTTGGTGTGGTGGATCCCACCTGTAATCCCAACACTTTGAGAGGCTGAA											
rs6478813	TTTTGGTTAAGGCCTTTAGATTGAGTTGGAATCCATATAATGTTAGAACTGGAGCGTTTTCAAGGTTGTTCAGTTCACTCCCCACATTTT											
rs318841	TGCCTGCAATTGATGCATGCAGTATTTTCTGTAAAATCCTCCAAGGTCAGGTATTCTGAGAAAATTTTATAGTTTTAACTTTTTTATTGCT											
rs1258464	AGTATGGTAACTACTCAAAGTTCTTTGACCATTCACAGTAGGAGAGGGATCTGTGGTTTGACTGGGACCACCAAGACTTGAGGTTGAA											
rs1433375	CAGGAAAACATTTATATTTGAACAACTTTTGTGTACTAATTGCAGTGAGAGAGAATACACATCGTGGGGACTGACCGAGATGACCTCAA											

**SNP**												
rs2401810	AGTGTTTTCAAATGAGCTTAACAAGCTCCTTATTTCATCTTAAACCTCTGCTACAGGAATTAAGGCCCAGTGATGGGCTTATTTTTGTTT											
rs803422	GTGAAACCAGAAGCAAGACTACCATGAGACAACGTTCCAGAGAAAGGAGGGACTAACCAGAGCAGGTGCCCAGCTTTGGCCAGTCAG											
rs2803543	ACTGCTGATTCTAGGACTGGGGGTAGGAAATACATAAGATGATCCTGGAGTACCTTGTAGTGTCAGAAAGTAAGGGAGTACTAAAAAA											
rs6068122	ATCCAAGGGGAGCCTTTGGAAGGTTTTAGACGGAGAAGTGGTATGTTCTAGAAAGGTGTAGCATGGTGGCGTATGCCTGTAATCCCAG											
rs365063	GGCGACATCAGCTGCCATTATGGGGGCGCACACACAGAGTAATGGTTGGGGATGGGGTCCCTGTGACCACGGTGTGGGAAGTACCA											
rs2156208	AATCGAAGGAGTCCGTTACTGTCCACCATGTCCATCCGTCAATAAGTATTTATTGACAGCCAACTACTTAGTACCCAAAGGGTGGTGT											
rs1347423	CGGCACCAACCACTTTTACGTCCTCACAACATCTCCTTATGTTGTTGTTAATGTATGTATGTGTGTTAATGGGTACAAGTTATCTCTTTC											

**SNP**												
rs1560434	TTTGCCATTTACAAGCATCTTACACTTTGGGGTGTTTTGGTATAGAAGGCTATTTAATCAAGCACTTCTGTTGCATTTAGGATGTGAAGTA											

**SNP**												
rs2134180	CCTTTATAATCTCAAGTCATTTACAGTCTTCCCTGAGAAAATTTGTCAAATGCAATGAATCATTCAGGAAAATGCTATGCCCTCCCATTCC											
rs2180289	GGCTATGACTTGGTTTGGGTTGACCCAGAAGCTGCCCTTGAGGTAAGAATTTGAGTACAAGTAGAGAAGGGAGCCCAGGAAACCCTG											
rs1366660	GCTTAAAAGTTCTTTAAAGTACCTTCTTTATTCACTCATCAAAAAGTTTGTCACTTACTCTGTGCTAGGGGATGTTGTAAGGTGAGGACA											
rs1825443	ACCTGTAAAAGCAGACAACCTAATGGGAAAAAGAGCAGCACTTAAAATGCTGTTTTTTCTCCACTGCTCTCTAGTGTAAAGAACGTTGT											
rs8096868	GAATTTGTATTTTTATAGCATCCTATGCACATAATTGGTATTGAATGGATATTTATCTAAATGAATAAAATAGCATTTGAAAAGTTGTGAAG											
rs12466929	CCACAAATTAAACCAGCTTCAATTCTGATCCTGAGATGCCAGGATATCTAATCTTATTGTTTACCGCCAACCTGCACTCTAGGACTGAG											
rs846752	TGAGTCCACTACAGGTAGGCAGTGGTCAACTTGAAATTCTGTCCAAGAGTAGGAGGGTTCCAATCACCAGCAAGAATAGAGCTTAAGAT											

**SNP**												
rs12472674	GGCCTTACCTAAGGGGTTCAGTCAGCTTGTGGTTCAACCCCATTCCTGTAAAGAATGTTTACTGACTGCTTGATTTGCTAACATATTATA											
rs4606154	AGTCAAAAGACTCATATTTGTCAATTTCAAAACTTACTACAAATCTACATTAATCTTGACTGTTTGGTACTGGCATAAGGAAAAACATACA											
rs7292634	CACTTAACATTATTAAATCTAATGATGAAGTGGGGCATTACTGAGATATTATTGACTGAAGGAAAGTGCTTATGAATACAGCCTCTGTAT											
rs273473	TTATGTAATTCTTCCTCTTCTGATGGCACAATTCTCATCTATGCCTTCTACCTTACATGATTTTCCATTACTAATAGTTTCAACCTGGAGT											
rs2084851	GCAGGTGGGGAACACATCAGGGCAGACCCTGCTTCCCAGGGTTAGGCCCACACCGAGATTTGTAACTTCTTGGCCCATGTGTCCTAT											
rs667415	AAACCATGGAGGCTAGGAGGCAGTGGGATGACACAGCTCAAATCCTGAAAGGAAAAAATCTTGTCTGCTAAGAATTCTAAATCTGGCAA											
rs1486048	AGGAAACAGAGGAAATCAGAACTGAGCCCATATGGAGAGAGGTCTTGGAGTCTAACATGTGTTATGCATTTCCTGTGTCAGTTTTCCTA											

**SNP**												
rs2730648	TATCTATAAAATATATCTTGAGTTCATGTTCTCTTCAGTAGAGGATAGAAATTTAGAGGACAGGCTCTGGAGGTAAGCTACTAGAATTTA											
rs1777467	GTTTTAGCATCTTTCAGTCATTCGGTCAGTCACTCAACAAACACTAACTGATGCCGGGTGCCAGGGCTGGGGTTTCAGATGTGCACTT											
rs2835896	CCCTCCTGGGACAGAGGTGGTGTTCCCCACACTGCAGCCTCAGATACCTGTGGGCCCTGAGTTTTCCTGAAGGCACTTTCTGCTGCT											
rs12583473	ATTGTGCCTTAGCACTGAGGATTGCAGGTCACTGATTCATAGCTACCTAAGATTCCACTTGTAAAAACATAGACATGAGCCCTTTTGG											
rs3899706	ATGAGCAGGTCATCACTCTGGGCCACTGGGGCTCCATCCCTTTGGGAACCTCTAACCCAGGCATGTGTAGAACATGCCTCAGCACTA											
rs4739199	CATCATAGACACTTTCAGCATAACAAAAGCCTTTGTAAGAATCCTGCAGTCCCATGACTTACAGGCCAAGATAGAGTGAGGCCTGCCC											
rs7855283	TCACGTTAGGATATTGTGTGGGGATATCCCAACAAGTGCCTTTTGGGTTTGCACATGTCCTTCCCATCTCTTTTGCCCAATCTGAGTCC											

**SNP**												
rs1607185	TCTGAACCAGGTAAGTGGGCACTCTGAATGCCAGGAGATCTGCCTCAGCATGGATCAGAGTGGTCCCTATTTCACAATAATCTCTGCA											
rs4971653	TCTGAAGTAAAATATCCATGCATATTTGCTCTGAAATAAAAATGTTACAGAACAATGCTTTTATTTCAAGGTTTGCATTTTTCCAAAGGTT											
rs2840794	GTAGGTGGATGGAAAAATACAAGGCACAAATAGCCCACATAGGGAATAATCATACCTTAAATAGTCACTGACAACATTTAAAAAAATATA											
rs12426585	AATATGGAGCATCAAGAACCGCCTCAGTCTCGGAGTGTTCGGAGGACTCCAGTTAACAGTGGAGGAAAACCAGGCTTGCGTTTGAAT											
rs2938675	AAGGATCTCAAAGAAAACTGAATGTAGAATTTAGATACTTCAAACTTGGAAAAAAAAAACTCACGTTTTTAGTACATAATCATTGTGTAAC											
rs3776720	CCTTTCTTCTGGTCACTAGCTGCATGTGAGGGTGGTAATCTACATAAATAAATGGACATCACGTCTGCCTGCAATAACGCCTGGATAGA											
rs4306755	AGCATTTTTCTCAACTGTAAAGTGGGGATAGTAGTACCTCTTGCATAAAGTTGTGCGCACTAATACATGTAAAGCGCTTAGAAGACGGT											

SNP												
rs7693776	CAGGAGCGATGTACGTTGTCCCTTCCTCTGGGCTTTGCGGGGGTGGGGGTGGGGGTGGGGGCTGCTGCTGCTGTGGCTGCTCCTC											
rs2760396	AATAAGGACAGACATTGTGTTACATCATTTTGTAGTTAATATGTTGTCAGACAGAAAGCATTCAATTGTCATGTGTTAATCTGAAGATAA											
rs4933826	CTTAGCACAGGTGAACAGCTGTATATATAACCAGAAAGGCAGGGCTATCAATTTGAGTCCTCTGTCTATAAGAGAGGGTGAACTTTAC											
rs592069	AAAACCTCATCTCAAAGTTCCTCTGTGAAGACATTCCTAATTTTTACCCTCCCTCTATTCCATCAACAGACTTGGCTTCAAATCTCAGGA											
rs2925067	GGTGATGCCTGCTTTCTCAGCCACAGGCATGGACATCTGATCCAGACCAGGCCTCAAGTATTAAATTCCTTAAGTCCCAGTGATTTTTT											
rs2071748	AGGACCTGAGCCAGACATCACCCTGGCTGCGGTGTTCCATGTCTCACAGATAGCCCAGTTCAGGGAGGCGACATGCCCAAGAGTGC											
rs7555995	CAATCACCTAGAGAAGCCTTGAGACCATGTGAAGAGAGTGACAGGTGCCCAGCCAGCCCCCAGCTTCTTCAACCCCCATTTCCAGTT											

**SNP**	
rs4873622	TGGTCTGGGACTACTTATCCCAAGTGGTATCTGATTAGTGATATTAGTTTGGACGTTTTTCCCCTCCAAATTTCTTGTTGAAATTTGATG
rs1891403	TGGGTGGAGCCTGGCGGCAAGGAGTCCAAGAAGAAGGCTGTTGCAGTGGGAGGTTGGGGGAGGCAGTTTCAGTAGGGCTTTGGCT
rs2901585	CAGGAGAATTGCTTGAGCCCAGGACTTCAAGACCAGCCTGGGCAACATAGTGAGACCCCCCCTCTCTCCCAAAAATAATTTTAAAAT
rs6478813	TACAGGCTGGAGAAGTTAAGCAATTTGTCCAGTGTCCTATAAAGGCATTGTAGGGGGGATCTTAGGGAGAAAGACAAGGTTCCTCAGC
rs318841	GTTGTTACACTGAAAATATTATAGAAAACATCATTTTATCAACCAAATGTGGTAAGTTAAAAGCTGATATCTCATTGCCTAGAGTGATGT
rs1258464	CTGGGGTGAACTGATACACTGGCATCTGGACAAGTATATCAGGAACCATGTGGATATGGCCCTCCACCCCTGTTTCTTGGAATATCAT
rs1433375	AGTTCCCCTTAGTTTGTCTAAACTTTAGACAGATTTCTTCCTGACTGTGGGTCCCTGACCTCCCTGTCTCAGAGCATCTACTTCAGAAA

**SNP**	
rs2401810	TTGTTGTTGCTGTTGCTGTTGTTGTGTTTTGCTGCAATCCAAGTCTGTCAGATCAAATTATTTGTTCCAAGGAGACTGTATTCATTCAG
rs803422	GCTCATCTTCCAGTCCTCAGTAGATTATTATCAACTTTAAGTGATGTCACAGACTCCCTTCCTCCAAAGCTGTGGACCTCTTCCTTCCC
rs2803543	CAAAAACCTAAAAATCCAATGCTGAGGCTATGTCAAAAAAACGAATGAGCCAGGCGCAATAGCACACGCCTGTAATCCCAGCACCTTT
rs6068122	CTACTTGGGAAGCTGAGGCACGAGAATCACTTGAACCCAGGAGGCGGAGGTTGCAGTGAGCGAGATTGTGCCACTCCACTCCAGCC
rs365063	GCTTCTTCGGCCTTGGTGGTAGGAGCCTGCCAAAAGCCTCACCTTGTATCCATCCCA
rs2156208	ATGCTGGCCGGGCGCGGTGGCTCACACCTGTAATCCCAGCATTTTGGGAGGCCGAGATGGGCAGATCATGAGGTCAAGAGATCGAA
rs1347423	ATATGTCAAGTTACTGTTAAAGGCAAGGTTATTATCTACATGTCCAAGTAGGTGACAAGACCCAAAACCTGGAGACAGGACCACATTC

**SNP**												
rs1560434	AATAAACAACAACTGTGATTATTTAACTGCTGTAGTGGAACTGAATCAGTTTAATTAATCACAAAAGACATTTCCTGCAAGTAAGCCTCAA											

**SNP**												
rs2134180	CCACCATTACCTTCTGGGTAAAACCCCAGATCCTAAACATGACCTGCAACTGCCTTTCACGATGTGATCCTGGCAACCTCTGTAAGAC											
rs2180289	GCAGGGAAGGGGATTGAGGAAGCAAGGCAGCAAAGGGAGAGCAGCTGGTAGGAGATGTATTATCAGGGTTACCAATGCAGGCACCT											
rs1366660	GCAAAGCCCCTTTGTTTTAATGTTGAGAGCCTGGAGGGAGAGAGATTTGATGTTTAAACTCATTTGATAATTACAGTGCAATGTGGAGA											
rs1825443	AAAAATATTTCAAACTGGATTTTTTGTTTTGATTGCGTTAACTCTTTGTTGGACCGTCGTCACACTTTTCCCACAAAATTTACTTTCGGTA											
rs8096868	CATTCAATACTATTGATGTTATTCCCTGTGAACCTGTTGGAACAGAAGACAGGCTGGGAACAGCATATGTTGGTAATGATTTGGCTGC											
rs12466929	CTAAATTTTCAGAGTCTCACAGACGACCAGCTGCTTAAAGAGGTGCCCGTGGGCCTTGGTGGGCTGCCCATCCTCTCATGGCAGAGG											
rs846752	TTGAGTGGCTATTCGACCCAGTGAGTCAGATTATTAGTTCAATAGAGTGAATTGGCTGCATGGGGGAAAATATGAAAGCCCAGTGATA											

**SNP**												
rs12472674	ACCCTTCGATTTTCAGCAACCAGTGTTTTATAGGCCAGCAGATTGCCTTTGATTTTTGAGAATTTGTGGTAACATATTGAGCTTACCAGA											
rs4606154	AATCAATGGGATAGAATTGGAAGTCCATAAATAAACCCTCACAGAAAAATTCCCTGTCCAGTCCTGCTGTAAGCATATGGTTCCAAAGC											
rs7292634	ATACTTCACTCACATTTAGCCTTGTTTCACGCTCACATGGCCAGCATGAGTCCTCTCTATTCACTGATGTTACCAATAACAAATTCACAT											
rs273473	TTTCTTGAGTGGAGCCACTTGAATTACCTGGAAGTCCCTTTGGCCAAGGAGGCAGAAGGTATTGACTGATTTAGGCATTACAAACAAGA											
rs2084851	TTTGGTTCAGATGACAGGGCATACA											
rs667415	AAACTACCCTTCAAGAATGAAGGAGAAATTAAGTCATTTCCAGAGACAAAGAAGGACATTATATTTTAATAAAAGGTTCAACTCATCAAG											
rs1486048	ATCTCTGTGATAACACAAAGAAACTGCAGCTTAAAGAAGGAAGACAAAGGTTATAAATGTACAGCATTAAATAGCCAGAACACAGTTAGC											

**SNP**												
rs2730648	ATATGACAGTAATAATTGCTAGCATTTATTGAGCACTTACTAGTTGCCTGACACTACCCTAATTATTTTACATATATAAACTCATTTAATCT											
rs1777467	TAGCTCACACTTGAAAAGGATTCCCATTCCAGCCACAGAGTGACCTTCTGACCTTAAAAACCCACCTCTGGAGAAGGCTGTTGATCATA											
rs2835896	GTGGAGGTGACGATTGGACCCATGGATGGCCGGCTGTGTGTTTATCTTAGTGCTTCTCACCAGGACAGCCTCCCATCACCTTCACTCA											
rs12583473	GTTAGAGTACATGATTGTTCCCACCTTTGCATTTAGGAGTAAGTTTTCATGTTTGGTCTGGTTTCTTCTATCCATTTGATGAGTACTAGAT											
rs3899706	CCCCATCTAAAGGGTGATGAAGCTAGGGTATTTATCTGTCAAATACTGTTCTGTATTAATTGAGAACTGCTCCCAGGGATGTTAGCTCCA											
rs4739199	CAGAAGAAATGTTGGGTGTGGCATTCACCTAATGAAGTGGACAGTAATCTCACTCATCAAATACTCACAAAGTTTTTAAAGGAAGTATT											
rs7855283	GGTTGTGCCTCTGGGGCTCCTCCCAATGGTTCAAGGGATTTTAAACTTGATCTACATTTGAAGCTCTCAGCCTATTTTCCAGATCCTCT											

**SNP**												
rs1607185	TAAAAAGGATGAAGAGGCTCAGTCTGCTGAACCAGGCTAGTGGGTGCTCTAAATACCCAGATTTTTGCCTGAGAATGGACCAGAAAA											
rs4971653	TTTTTTTTCAATTCCTCTGTTTGCAATGCAGCAATATCTTGTTTGAATGCAAATTAAATCTTCCTCTTAATAAGAAGGATGTGTCTCTGCC											
rs2840794	ATTTTTATTTCCATTTATGTAATCAACTTAGGGTTGTTATTGACCTGCCACTGAGGCATTTGGTTTTTCTTTTCATAGGACCTTTTTCATTT											
rs12426585	ATTACTTTAGCTGGAAAACACTATTTCATTACATTAATGTCTGCACTAATTATTATAATAATCCTGAGTTTACACCAGTAACAAATCAATT											
rs2938675	GAAGCGAGTCTTCAATCAATATGTCAGAAGATGTCATTTTGCACTTCCAGAGTTAAAGATTTTTTACTGCCATGACCCCTCCCTCCTCCC											
rs3776720	CTCTCACCACAACTAGGGTACATCTGGAATAATCAGTGAAATTATACTGAGTGAGGCAAACAGCTACCCTTCAGAAGGGTTGAAACTG											
rs4306755	CTTGCACACAGCAGATGATCAGAAAATGTTAGCCACTTGTTATTCCTACAATGCCTAGCACAAACACATCATAAATACTCTACAGTGCTT											

**SNP**												
rs7693776	CGTGAATCCCTTTTAACTTTCCCAACTTCTTTGACTTTCGTGCTATAAGAAACCATAAATGATAAGGCCAAATTAAAATTATGTTTGAAATA											
rs2760396	CTTTAAAGATTGGAGAGGATTTGAATAGTTCTAGAGGATAGGGCAACAGTTACAGTGATGGCATGGGGGTGTTGTGTGTGGGGGTGG											
rs4933826	CAAGATAAATATGCCTTATCCTCAGCTGAGCACAGAAGACCCATTAAAGCAAAAAGGTAACTCAGGCAATAATCATCATGGCTTGGTAAA											
rs592069	ACGTGGCCCATTTAATGAGTTCTCAAGAAATGATTATTGTTTTCTCAAATTCTTGGAGTTGACCTGGTTTCTTCTCCAGCCTCTGACAGTC											
rs2925067	TTCCTGAGATAAACATATTGTCTGAGCAGAGCCATCAGCATCCTTCTCTTTGACTTTTCTAACTACAGCATGTGGAAAACAGTCTGTTTCT											
rs2071748	TCAGTTAGCTGGTGTCAGAACTGGGCCTTGAACCTTGGTCTGCCCACCTCCAGGTCTCACTCATTCCCTTCTTTCAATAATTTGTTAGT											
rs7555995	GCAGCCACCATCT											

**SNP**												
rs4873622	CCCAGTATTGGAGGTTGGGCTGAATGGAAGGTGGTTGAGTCATGGGGGGAGATTCCCCCTTGAATGGCTTGGTCCTGTCCCTGCAGC											
rs1891403	CACTGGATTCAGAGAGTGTACTGGTTAGTGGTTTGCTTGCAAATAATAGATATCTAGCTTGCATTAACTTAGCAAAAAGGTGAGCCTATA											
rs2901585	TAGCCTGGCATGGTGACATACACCTGTAGTCTCAGCTACTTCAGGAGGCTAAGGCAGAGGATAGCTTGAGCTCAGGAGGTCAAGGTTC											
rs6478813	ACCCCTTAGTGATGCATTTATGGACACAGTTAACTTTTTTCCTTTTTTCATTCACGACTTGGGTGTATTTTTTGTTTGCCAGAGTCTCTTC											
rs318841	TAGTTAGTTATTTAATATTTCTATTAAATTTGGTTAGGTAGGACACGCTTACAGCAACTCCTTGCCCTTTTATCTTTTTTGGGGGAGAGG											
rs1258464	GTTCAATAATCTCTTCAGCTTCCTGACCTTCATGAACTTTATGTTCTATGTAGTGGAGAATTATTCTACCTACCAGCCTGTGGGGCCAAA											
rs1433375	ACTTGCAATTATAAGTGCTTTCTCTATCCCTTCAAGAAGTCAATCTTCTCTCAGCCTCTTGCCAGTTTTACAACCCATAAATGTCTTTTTC											

SNP												
rs2401810	TGAATATAAGAAAACCAAGGACAAGAATATTATTCAAACTGCCATCAGGGCCCCTTATCTCCATGAAGCCAAGGCTACAGGTGGAAGG											
rs803422	ATGCCTCTTGTTACTTGGAAGTCACTCTACTTCCAAACATGTCGGCTGGAGATCCCTGATGGGCCAGTTACTACTTTCAGGCTCTGTAC											
rs2803543	GGGAGGCTGAGGCGGGTGGATTGCTTGAGCTCAGAAGTTCAAGGCCAACATGGGCAGCATGGTGAAACCTCATTTCTACAAAAACTA											
rs6068122	TGGACGACAGAGCGAGACCCTGTCTCAAAATACAAAAACAACCTTCTCAAAAACAAAAACAAACCTACACCTGGGGGAAAAACTAACA											
rs365063												
rs2156208	AGCATCCTGGCCAAAATGGTGAAACCCCGTCTCTACTAAAAATACAAAAAATTAGCTGGGCGTGGTGGCACGTGCCTGTAGTCCCAGC											
rs1347423	AGGCTGTTGTAAGGCAGATCCTTCATCTCAAGTTGTCCAATGCCCATCTCCCAATTGTCAGATGTTATAACTCTGCCTCTCCCTCTCATC											

**SNP**												
rs1560434	AAAGCAAAGAGATTATTGTGATTATACAAAATAGGGGAAACCAGAGATTGTTGCTATTAACTTCTTTGGGAGTGGTTTGAAAGCCCTAG											

SNP												
rs2134180	AACCCCACTTCATTCCCCAACCCACAACTGGCACTTCCTGTGGGGAAAGTTTCCAACATGTGCTGGTCCTTCCATCAATCCCTGCCTT											
rs2180289	GGAGCTTAATCCCACTGAGGAAGCCTGGGAGTACGGCAGACCAGATGCCTCAGAGATATCCTGTGTGTGAGAGTAGGGAGCTGGGG											
rs1366660	CGGCTTTCAGAGCTCCTTTGGAAGCAAAACA											
rs1825443	ACCAGAGATGTTTTGGAGAAACTGAATATTATGTCTGCAAACAGAGGGAAGCAGTCAGAGAAACCCAAAGGGAGGCTTGCTAATGTT											
rs8096868	AAAAAGTGTGAGACTTTTTAATAAAACTGTACTAGACCACATTCTCTCCCATGATGATATTTATGACATTTTAGGTAGCAGGAGAACAAG											
rs12466929	TGGTCCCTTCAGCCTCTCCTCTCTGCTTTTAGGACCCCCAGATCCCACTCTTCCCCTACCCTCCTGCCCTAGAGAATACCACTTCAGG											
rs846752	ATCACTAGATTTGAAATCACTAGATACCAAGTCCCAGGTTATAATAGTTGGATGGATGGCTCAAGGTGTGTGCTTTCTTAGTGGTCAGT											

SNP												
rs12472674	TTTATGCAGGAATGTAAAATAAAAATAGTGTAGCAGGCACTCGGGATTTTTGAACACAGAGGGGATTAGCTTTACAGATGGCTGGCAC											
rs4606154	TCAGTAGCCTATCCAGAGATGTCCAATATAGTTGGGATACCAGCTGTATCCCTACGTGGAACAGGGCCATCTTGTGTATACTTTTCAT											
rs7292634	GGCCTGCAT											
rs273473	CTAAGGGTGGGAGAGGCGATTTCCCAAGAGGAGGTACTAATACCATAAGAATAAAGAGACACTACTGGGTAGAAAAACTTTATTTATT											
rs2084851												
rs667415	AAGATATAATAAACATCTGCATCTAGCAACAGAACCCCAAAATATTTGAAGGGAGAATTGACAGACTTTAAGGGAGGATAGTTCTACAG											
rs1486048	ACTTCAAGATGAATTCATGTTAATCTCCCTGGGAAAGAGCAGTTTCCACAACTTCGTGCAGGTCTCAGTGTGAATTGAAGAAACAAAA											

SNP												
rs2730648	TCATAACAATCTCACAAGGTAAGTACTATTATTATTACCTCCATTTTAAAGAGAAGCATGGAGGTGTTAAGCAATTTGCCCAATCCTGTT											
rs1777467	TTTTTTGTATCTTACCAGTGTGGATACTTTGAAACAGAGAATTTAAATAACTTGTCTAAAATTACAAAACTAGCAGAAAGTATTTACACCA											
rs2835896	GACCCTTGACCTCCTCCACCTTGATGGTTCTTTTAGCGAGGACAAGCTCACACGTTCAAATAGAGAAGGGGCATGGCCAGCTTTGGA											
rs12583473	TTATCTTTTAAGGCAACTTCCCATCCTTTTCCCTTGTCTTGGAATAGCATTCCTTTTATGTGGCCTATTTTTTACATGGAATTTTTCTCTAA											
rs3899706	ATAGCATTTCTGGCCTGCCCCATGCACCTGCAGACAGACATATGCCTGAAGCCAGAAGAAATCCTGAGGCAGAGATGAGTTGGTGATT											
rs4739199	TCACTTCTTACTAAAAGGGGACAGTGACAGATCAGAATGAAAAGAGGCCTCTACCCCTATCCCAACTCAACTGAGGCAGGAAGCAGAT											
rs7855283	GATACTCAAACATTATTCTTGCTCCCCTCTCATCACCCAGCCTTCTCCATCTCCTACACACTCTGAAATAATCTGTCAGAAGATCCTCAT											

SNP												
rs1607185												
rs4971653	TCAGGACCATGTACAAACAGTCTGGTGATGGCAGGCAGTGACCCAGGGATGTTCATGATGGACTGAGAGAGACATGGCAAACATTAC											
rs2840794	ATTTCTTCAGAAGCATTATCTAATTAAAAACAAACATTTAAATTAACATGGCCTGTCAAGGAAGTACAAACCAGTCTTGAGTTAAGCTAT											
rs12426585	GTAATGAGCTGAACAAAAGAATCTAGGTGGAATTCTTCCCTATAAATATGCTCCTGTCAGTGCAGTCAAAACACAGTGCCAGAACACTG											
rs2938675	CATGACAGCTTCCGGATTCCTATAAAGCCCTAGCACCTCACATTCCCCCAGCCACTTCTAACTCTGCCTTCCTCTTCTTCTAGCTCTCC											
rs3776720	AGGAAAAAGGAGAGAATGATGTAACAACTGATGTAGAAAGATGTGTCATAAATATTAAGCCACCTTGGGTACCAAAACCCTGGATGGC											
rs4306755	TAGAATCAGTTTCATATATTTGTTCTTGGAGGTTTAAATCTCAGCTTTGTCAGAACTGCTCCAAGAAGGAAACGGTAAATATATATACAC											

**SNP**												
rs7693776	ATGCTGACTCACACTGGTTAAAGTACAGTTTTCCTCTAAATAGATCTTGTTATGTCAATAGGAACATGACCGTTTATTCCACTAAGAGAA											
rs2760396	GGAGTAAGCGGTTGTGAGGTCTGTGTGAGTGTGGGATATGTGAGGGCTAGACAGTGTGCAACAGCCTAGCTCTGTTGGAAAGTCCAT											
rs4933826	AAAGCAGTTCTGATAGACTTAATCTTTAAGGCAGAAGGAGGACAGGCTGGTGGTTTTATGAGAAAGTAATGAATCCTTTTGACAATTGT											
rs592069	CAGACCAAATGCAATGCAGGCGGCTAGAAATCAACTATGCAGTTGTGAAACCTGCAATTTGAAGCAAGATTCTTGATTGCTCAAATGAG											
rs2925067	TCTTTTCACAAGTGATTCTGGAGTTGCTCATGGTAGTTTCTCCCAGTGAGAAAATGAAGTCAATTAATAGGACAAACAGAAGGACAGAT											
rs2071748	ATTTTTTTTTTTAACTCCTGGGCTTAAGCATCCTCCCACCTCAGCCTCCAGAGTAGCTGGGACTTACAGACATGTGCCATTTCTCCTGA											
rs7555995												

SNP	
rs4873622	CAAGGAGTGAATTCTCTCTCTATTAGTTCATGTGAGAGCTAGTTGTTTAAAAGAACGTGGCACCTTCCCCTCTGTCTTGCCATGTGACAT
rs1891403	AGACAGGCTTGCTTTAACGTAGGAGTAAGCTGGAACCAGGGGTCTGAATGCTACTGAGGGTCGGCTTTATTCTCTCAGACTAACATCA
rs2901585	GCAGTGAGCCATGATCCTGCCACTGCACTCCAGTCTGAGTGACAAAGGGAGACCCTGTCTCAGAATAATAATAAAATAAAATTCTTTCT
rs6478813	CCTCTCTAACCTACAGAAGAAGGAAAAATAGGATCTAAAGGTAGATAGTTCTATAGCTAAAGAATTCCTTTTCTGAATTGTTTAGGAGAG
rs318841	ATTTGGCTTATATCACCTCACTCGACTAGCCATTTATACTTGTCAGAGTCACCCTTGTTTATCAGTAGGTCAGTCTCTGAAAACTGTGTC
rs1258464	GCAGTATCCTTTTAATAATCTGTACTTGGGACAAGACAGTGATCCACCAAA
rs1433375	AAGGACTTGGAAGACATCCCTTTGAAATGTAATCATCAAGAAAGAGAGAATACCTTATTTCCTATTCTCTGTGGGAATCAGAAGCTTACA

**SNP**												
rs2401810	AGAGTCTCAAATACCAGTGACAGAGGTGGCCCAAAAGACCCTCAGGAACTTCCACTTGCTCCAGGTCACATGCTATTGCTCAAGAAT											
rs803422	GTTTAAAGAATGGTTATATCCAGTTAGTTACAAATAGAAATCGATCCTTTCTCTGTCCGTCTGTCTAAACGGAAGGATATTCTGTGGAC											
rs2803543	CAAAAATTAGCCAGGCATAGTGGCACATGTCTGTGGTGCCTGTCTGTAGTCACTGTTACTCAGGAGGCTAAAGTAGAAGGATCACTT											
rs6068122	AAAGATGTGTAAGATATCCACAGAGAAAAATTACAATGCATTAGTGAAGGAAATAAAATAAGCTCTTTAAAAATATAGAGCTCTGCCTAA											
rs365063												
rs2156208	TCCTCAGGAGGCTGAGGCAGGAGAATCGCTTGAACACAGGAGGTGGAGGTTGCAGTGAGCCGAGATCCCACCATTGCACTCTAGCC											
rs1347423	GCTGCCTTTGCACAGATTAATT											

SNP												
rs1560434	TTATTTAATGTGCATATTTCAGGAATATACTAAAGGCAATCAGTGATGTAAAGCTGGTAGTGATGAGTGCAGATCATAAGACTTTTCATC											

SNP												
rs2134180	GCCTATGTCACCTCCTCTGTGCTCAACATTGAGGCCAGTGCATTACTCTTTTAACCCTAGGATAACAGCTCACACATCATCTATGGGGT											
rs2180289	TGTTTATACACCCACTGCCATCAGTCTTTGGTTCAGGGCTGCTCCCAGGAGACGTTACTTGCCTGGAATGTCCAGCCAGCCTATGGC											
rs1366660												
rs1825443	TGATCACTTAAACTCGTCTGTCCTATCATACACAATGACCACAGCTTTGGAAAGCTAACAAACCAGTTTCTTTTTTAGGAACTCTCTTT											
rs8096868	ACTCTTGAAAGTAGAAATAATGGAACTAGAGATAGAACACAGAATTGGAAATGGATTTTTTAAAGAATGTGTGGCTAGGAAGGAGTCC											
rs12466929	CAACACAATCCCTCCGCCTCTCTCCACGTTTCTGCATATGACCTTACCTACTCTATCCTCAAACCACTTCGGTTGGTTTAACTTCCAGA											
rs846752	GCAGGGCTACCATTGTCTTAGAATAACTTAGTTAGAAGGATGAAGATGATTGAGATGGCCTAAGTACTTCTCACATCATCAAGATTGAT											

**SNP**												
rs12472674	ATATAATGGCATTGA											
rs4606154	GCTTCTCTGCCTCACTCTCTTTTTCCTCACTTTGGCTGCCCTGATCCAAATATTTTAA											
rs7292634												
rs273473	TTCATTTTTATTTTTTGAGATGGAGTCTCACTCTGTCACCCAGGCTGGAGTGCAGTGGCACGATCTCAGCTCACTGCAGCCTTCGCCTC											
rs2084851												
rs667415	TAATGGTTGGACATTTCAATACCCCATTTTTAATCATGGATAGAACCAGATAGAAGATCAATAAGAAAATAGAGGCCATGAACAACACT											
rs1486048	CATTGCAAAAGAGTGAGGGAGAGAATTATTGATTTAGTGGGGTTGCTAATAGCGTAAATGAGCTTCCTTTGAAGCAAAAGGCT											

SNP												
rs2730648	TGGGGCCAGAATTCAAACCCCTAAGTTTGGTTTCAGATGTCACTATGCTAGGCTGTCTCTTGGAAAAGATAGACCAAAGATGGGGAGTT											
rs1777467	AGGGATTTCTTTAAATCCCCTGCTCTTTCCACTGAACAGTGTGGCCTCTTTAAGCATGCAAATACACTGTGGTGGGTTGCATTTCAGAGA											
rs2835896	GAAAGAGTACTGTTAGCATTTTTGGTATCTTTCAATACTGCCTGACTGTGGAATAAGAAGCCACATTTTACATATAGGGATTTTGCCTTA											
rs12583473	GGAATGCCTCTCTACATTCCTACATTACATTAGCTATAAACTCACCTTGCCCCAGTATACCTAGCGCAGCTTCAATTTTTCCAATGAGGT											
rs3899706	TGCAGGAGGAAGCTATTGACATGAATGGGAATGGGGAGTGCTGTGAGAATGCAGGCAGGGTGTCAGCAACATTTCCTCCCGTGGGAA											
rs4739199	TAGAAAAATCTCAGCTGAAAACACAGACACTTCTTATAGAAAAAGAAAAGCATCTTGAGCCTAGACCAAGAGTTCAAAGGACAGAGCCA											
rs7855283	TCCTGCCGTGCTGGCTTACTCTGGATCAAGAGACAAAGACTCCAGCTCTATCTCTGATGGACCGAGTAGGAAGAATCAGGCAAGTGCT											

**SNP**													
rs1607185													
rs4971653	AAGCATGCATTGCA												
rs2840794	AATTACAATGACAACAATGTCAAAAAGAAAAAGCAAAAACCTTTTTTAAAACCTGTAATTACTTCATTGTTTATAATTTTTAATTGGAATGC												
rs12426585	CCTGTGTCAGTGAATAACACATTTTAAAACTGGTATGTCCTTTTAATCTCTTAATTGGTTGTGTATGTGGGAAATCCTATGAAAAGCGAA												
rs2938675	ACTTCCCTCACTCTGCACCTCCTGGGTTTTCCTCTGCTCCACACCAAGCCAAACATGAAACTCATCTTCACACTCAAAGCTGGCTTTGT												
rs3776720	AAATCCTGCACTAAAGTT												
rs4306755	ATGGCTGTAAATGTGTCCGAGTGCTTCAACATGCAGTGGCAGTTGAGAACAGCAGGGAGCCTGGGTTCAAGTTCTGACTGCAGCTGAA												

**SNP**												
rs7693776	AATATATAAAGCTCAATTATTATTACTTACTATTGGTATTAAGAGAACAAACTCCTTCCTTCTTTCATTCATTTTTACAAGTCTTCACTGCC											
rs2760396	GCTGGTGATAAATGAGGCATT											
rs4933826	ATTAAGACTTTATGGACATAAGACCTAAGTCAGAGTTTTAAAAATGGTTCTCAGAGACACTGTTTTGGCTTTGTTCTTAGTCACATCCCT											
rs592069	AGTTATGTAAGCTTTGTTTGCATGGTGCAATGGGCAGGAGGACCTCACTGTGAGCAGAGTTTTGGAGGCTATTTCTGGTGTGACAGC											
rs2925067	GAGAGGTAGAAAGGTATATGCACAGATACTCCTTTTACGAGGTTACCGTACAATGGAGGAAATTTGACTTACTTAAATCTGACCATCAG											
rs2071748	GACTTTGACCAGCTGTGTTGTAGAACAGGGAAGCAGGGCAGGAGAAAAGGAAAAATGACAATTCTGTGGGGAGGCAGGGATACGG											
rs7555995												

**SNP**	
rs4873622	GCCAGGTCCCCTTCTCCTTCTGCCATGGGTGGAAGCTTCCTGAAACCCTCACCAGAAGCAGAAGCTGAAGCCATGCTCCTTGTCCAA
rs1891403	TCGACGCAGGGGGAGTTGTGGCAGCGCGCCTCCCTCACATTGCACAGCTGTGCCTCCAGAAAGGGAGTGAGTCTATCTTCCCAGGG
rs2901585	TCCTTCAGCATCCAGGACATGGCTTTTTTCCTGGTTCTTTTTTTTTTTTTACTTCTGGCCACTCCTTCTTCTCTATTTGCTCCTGAAACTT
rs6478813	CTGGCTTGGTTTAAACCAGCTATTAGCCGGCATTCATCCAGTTGC
rs318841	TATCAAGTACACTCAACACAAACATCTGGATCGCAAGCCCTCAGGGGCACCCATGCAAACAAGCCCAAGAAAAAATATGCCTCCCTCT
rs1258464	
rs1433375	ATGTAATAAGTGCCAACTAGCAAACAGAGATGGCCTAGTCACAGTGACCCACCTCCCTCCCATGTCCTCCAGTGCTTTTCCACTAGCTC

SNP												
rs2401810	CCCAGTCCCGCCTGAAGTAATTACATGACAATGCTAAATTTCCTTCACCACCTCTATTGAGATTTAACTCGGGACTCAAAAACTAAGCAA											
rs803422	GCTGATTAGGTATAAAATCTCCACGGAGCCTGAGCATGAAGAATTGACCTTCTTAGGGTTCCACAGAAACCAGAATAGACTTCTGTGT											
rs2803543	CAGTCCAGGAGGTTGAGGCTGCAATGAACCAAGATCGCACCCCTGCACTCCAGCCTTGGCAACAGAATGAGACCCTGTCTCAAAAACA											
rs6068122	ATTCCAACACTTGTGT											
rs365063												
rs2156208	TGGGCACACAGCCAGATTCTGTCTCAAAAAAAAAAGAAAAGGGGGAGTGTATGCTGAGTTCCTGCCCTCCTACCTAGAGTTATAGGCA											
rs1347423												

**SNP**												
rs1560434	AGGGTAGAGATTTTCTTACTCTTACTCCATAATTCAAAATCAAGAGTAGAGATGAGTCATCCTGGGGAGCATCTTGAGAAAATGCTGAT											

SNP												
rs2134180	TTCCCCATGATGGCCTAGGTTGAACCTTTTCCCACCTGGTACTCATCACAGGACCATATAATTGCCTGGTGGTTTGTCACATTCGCTG											
rs2180289	AGAGGAGGCTCCTGCAGCCAGCGAAAGTCTTTAGGCAAAGATATGCCAGTGATGTGCATGGAAATGGTAAATGCTGACGGAATGTGG											
rs1366660												
rs1825443	CATTTGTGTTTGAACGAAACGTCCA											
rs8096868	CACTTGCTCTGTGATACATGCCTTTTTGTTGTGTACTAAGTCATCTGAGACTCTATAGAAGGAGCTTTTGTCCTTAGAAAGCAATTTTCA											
rs12466929	AACAAGAGCATGGACATTCTGCCAGGAAATTCTCCTAAAATAGCCACACATTATATACCTCAATGTTTGGAGGATAAACAAGCAAGCAT											
rs846752	ACAGCAGTACTCAACCATCTAGAAAGCCTTTTAAAGTAGCAATGGCTTTTGAAAATGGTGTAGTAGCTCCTATTGGATCAGCCCTCCAG											

**SNP**												
rs12472674												
rs4606154												
rs7292634												
rs273473	CCTGGCTTCAAGCGATTCTTCTGCCTCAGCCTCGCGAGTAGCTGGGATTATAGGCACGTGCCACCATGCCCAGCTATTTTTTTTTTTTT[illegible]											
rs2084851												
rs667415	ATAAACCAATTAGTCCTAACAAACACATATGGAACACCCCACTTAGCAGCATACACATTCTTCTCAATGCATATGGAACAGTCTCCAATA[illegible]											
rs1486048												

**SNP**	
rs2730648	TGAAAGCTATTCAACAGTCCAGGTAAGAAACAATGAGAATTTGACCCAGCAACAAA
rs1777467	AGGCCAAGATTTTCAATCCATGATTG
rs2835896	CATAACTTTAGATAAAGCACCATTGATCAAGCTACATGTGTGGCCCTTACAACATTTTAGTCTGCAAAAAGTCAGGTTCTCTATTCCAGC
rs12583473	TTGCATTCTAGTCTTTTGGGTGGCCAATTCTGATGT
rs3899706	ATAACAA
rs4739199	CAAGTGTGGAAGATTAAAAACAGGAGCTAAGATCAACGGATGAGGAAACCACTCCAAGGGAGCATCATTAGACTCTAATTAAGGAAAA
rs7855283	TTCACTAATTAACTGGATTACACGGTCCCCCACCACCCATCAGCCAAAGGCAGGCATCCCAGTCTCTGTTGTCACCCTCACAGTCCTC

**SNP**												
rs1607185												
rs4971653												
rs2840794	CAAGTAACAGGGAATTCAAATTTCATAAAACCGTGGTCTGTATATCTTGTTATCAAAATGCTATCCAAATAACACATGGCAAAATGTAATT											
rs12426585	AGTCTCTGTCCACCTGGATTTTTGAAGGATAGCTATACTTACAGCTGTCACAAGTTGTCAGTATTTTGTAAAGTGTCTGAGTCTGATTAG											
rs2938675	TGCATAGCAGACTTTACTAAGGCTGCCTTTTCCCCTGGGATCAGCTCCCCTTCCTATTTCTCTTTGCCCTGCTCTCTCTCTCTCTCTCTC											
rs3776720												
rs4306755	ACAGCCGAATGACCAATAGAAAATTAACTAAAACTTAACTCCTCAGCTGGGGAATGGAGATGATCATAATAATATCTACTTCATCGGGTT											

**SNP**												
rs7693776	TGTCTCCTATAAGAGACTGTATAAGCTGCATAGGAACCCAGCTAATATGTTCAGTATAACCAAGCCTTACCCGGTCCTCAACAAGTGTT											
rs2760396												
rs4933826	TCATTCAGGTTAGCTATCTGCCTGCCCTGCAAAGCCCGCCTCTTTTGTGAAGCTTTTCCTGACCACACCAGTATCTGCTGATTTATCAT											
rs592069	ACCTCTGCTTCATTTCCACCAAAACCATCAT											
rs2925067	CTAATTTAAGAGTGATGGATGTCAAAACAAAGGTAAAGAATTTCAAAGAATGAAAGGATGAAGAGGTTATAGCCATTGTTCACATATTA											
rs2071748	ACCCATGACAGAAATACTCCCAGAAAGACTTTTTCTTTGACTAAAGCAGACTTTCTCAGGCTTTTTAACCCTGACGCCTTCATGATGA											
rs7555995												

**SNP**												
rs4873622	CCTGCAGAGCCATGAGCCAAATA											
rs1891403	GAAAAGGTCAGGGAGACCTGGCTTGGGTCACGTGCCTACCTTTAGGCTGATCAACTGAGCTCAACCTGGGCCAATCAACAGTGGCTT											
rs2901585	TGAGTCTCCAAAACTCCTTCCTTGGTACATGCTCATTTGAAACACTCCCTTGGGTAATTACATTCATTTCCTTTGCTTCAAATGTGACCC											
rs6478813												
rs318841	TGTGAAAGAGGACAACAAACCAATTTGTTGAAAATGTGACAGTCTGCATCCCATCTCCTGCTTCCTTGTGCCCTGAATGAATTGTGAAA											
rs1258464												
rs1433375	CGCCCTGGTGCTTAACATCTCTTCTGCCTTTTGTGTCAGCAGACTGAGTTCAGTCCCTCTCTCTTTTGCAGTAGTCTTGAATAAAATCTT											

**SNP**												
rs2401810	AGTACATATTTTGAAGGCGGTCATTACCACAACAGCAACCCAGTGGCAGGAACCAGAATCAGTATCTGCCATTCTGAGATCACTCAACC											
rs803422	TTGGTAGCTTTGTAACTTACGCCCGTTGAGGTGTACGGTTGAAAATTGAGAATAAATTTCCTGTTCTGTTTCTGAGCATCTTTTCCAGGC											
rs2803543	AGCAAAAACAAAGACAAATGGAAAAGAGCTCCCAGTGGCCAAAGCAGGAACAATTTAA											
rs6068122												
rs365063												
rs2156208	AGTAAGCAAATGATTACAAATATGCAAATAGTGAAGCGTGCGCATAGGAGAACCCTAGGATGCTCTGGGAGTGTACAATGGGAAAACC											
rs1347423												

**SNP**												
rs1560434	GGTTTGGTAAACTGATGGAGAATGAGGACAAGGGAAAA											

SNP												
rs2134180	GAGATTT											
rs2180289	ATTGTGATTTGA											
rs1366660												
rs1825443												
rs8096868	GTCAAGTTCCAAAATAATGTCAAACAATTATCTTTTTAAATACATGTTTATGTATTGTAATTAACTTTAGAATTGGTGTTTCTAATCCCCAG											
rs12466929	GG											
rs846752	GTAGATAACATTTATAAACTCTGAACAAAATATAAAAACAACTATCTGGAGGAATTGAAGAGTGACCAAGTAGGCAGAAATTGCATTGTA											

**SNP**													
rs12472674													
rs4606154													
rs7292634													
rs273473	GCATTTTTAGTAGAGATGGGGTTTCACTATATTGGCCAGGCAGGTCTCAAACTCCTGACCTCATGATCCACCCACCTCGGCCTCCCAG												
rs2084851													
rs667415	ATTCAAACAAGTCTGAATAAATTTAAAAATATTAAAGCCATATGAAATATTTTTTCTGATTAGGATGGAATGAAACTATAAATCAATAAATG												
rs1486048													

SNP												
rs2730648												
rs1777467												
rs2835896	GTGTCAGCGGTGTGTCGGGGAACTTATGTGGAGGCTACAGACTTTTAGTGATGATGAAAACATTGTGTCCCTTTATTATTGCTCTGATT											
rs12583473												
rs3899706												
rs4739199	CCCATGCGTCTGGAGTTGGGAAACTGGTAACATGTGCTTGA											
rs7855283	CAATGCACGCCTGCCACAGCCTGTCGTACATTGCTCATTATTCTCCCATTTGCATT											

SNP												
rs1607185												
rs4971653												
rs2840794	TTTTTATGTAAATGGGGAGACTTATGAGACAAAGAAGCAAATAATCTGGCCACAATGTGA											
rs12426585	GTATAGAGGTGCGGAATTTGAGTGTCACAGAAGGGGCCTGAATTTGAATCTTGGA											
rs2938675	ACACACACACACACACACACACACACACACACACATACACACACATACATACCTCCTCCTACCTTCCTGAGTTTCTGTTCTCTAGCC											
rs3776720												
rs4306755	TGTGGTGCTTGCATTCTTATCCCACTTTCACTTTGGGAGCTAGTACAAGAATGTAATGACACAGTATAGTATTTCTACCTTATATGTGTCT											

**SNP**													
rs7693776	TCA												
rs2760396													
rs4933826	CTTCTCTAATCTCTGAACTAACTGTTCAGAGGGATGAACACAGTAAATACTAATACATGCCGTATACACTCCACCCTCTCCCGGCCTCT												
rs592069													
rs2925067	CAGGTTTTATGGGAAAGGATGCATTTGAGCTTTT												
rs2071748													
rs7555995													

**SNP**													
rs4873622													
rs1891403	GGGGAGGAGACTGAAACAGTAAGGAAGTCCCAATTCCAGCTACCGGGTTCAGAAGCAGCTGTGAGGTGTGCAGGACAAACAGTGAA												
rs2901585	TCAATCTT												
rs6478813													
rs318841	CCTCGGGAA												
rs1258464													
rs1433375	CCTTGCCTGTTTAATTCCATCTGGTGCA												

**SNP**												
rs2401810	CATGCGATTTATGAAGCTGGATT											
rs803422	CTT											
rs2803543												
rs6068122												
rs365063												
rs2156208	TCATCCAATTAGAGGCTCCAGGAAAACTTCCCTCAGAGGGTCAGTTAAGCTAACATATGAATGATGAGTAGGGTTTACCAGGGCCACA											
rs1347423												

**SNP**												
rs1560434												

SNP												
rs2134180												
rs2180289												
rs1366660												
rs1825443												
rs8096868	TTTATGTATTGTAATTAACTTTAGAATTGGTGTTTCTAATCCCCAGTGCCAAAGAAATTCCCTGGAAGTAAGTTAGTTATTTAGGGAAGG											
rs12466929												
rs846752	GGAGGAATTGAAGAGTGACCAAGTAGGCAGAAATTGCATTGTAATCAGCCCT											

**SNP**												
rs12472674												
rs4606154												
rs7292634												
rs273473	TCTCAAACTCCTGACCTCATGATCCACCCACCTCGGCCTCCCAGAAAAATTTTAAATGTCCTCTCCAGGAGGTTAGGGAAACTGTTACC											
rs2084851												
rs667415	ATATTTTTTCTGATTAGGATGGAATGAAACTATAAATCAATAAATGAAAGCTGCAAAATCCACAAATATATGGAAAATAATAAGTTCTCAA											
rs1486048												

**SNP**													
rs2730648													
rs1777467													
rs2835896	TTAGTGATGATGAAAACATTGTGTCCCTTTATTATTGCTCTGATTTGGTCCAAGAAGAATAGAAGTACTTTGCGTAATGTCATGGACTGA												
rs12583473													
rs3899706													
rs4739199													
rs7855283													

**SNP**												
rs1607185												
rs4971653												
rs2840794												
rs12426585												
rs2938675	ACATACATACCTCCTCCTACCTTCCTGAGTTTCTGTTCTCTAGCCCCCTCCTCTCACACTCAATTCTTTTATTTTATTATGCAAAGCAATT											
rs3776720												
rs4306755	CAAGAATGTAATGACACAGTATAGTATTTCTACCTTATATGTGTCTCCCACTTTAACTTTTGCAAAGCACATTCATATATTATCTCATTTAA											

**SNP**												
rs7693776												
rs2760396												
rs4933826	AATACTAATACATGCCGTATACACTCCACCCTCTCCCGGCCTCTCAAGTTGTTTGTAAACTCTATTTTATAGTTACAGTCCACCCAACAC											
rs592069												
rs2925067												
rs2071748												
rs7555995												

**SNP**												
rs4873622												
rs1891403	CGGGTTCAGAAGCAGCTGTGAGGTGTGCAGGACAAACAGTGAAAGATCACTGTGGAAGAGACTCTAGGGTCACTGGCTTTAGGAACT											
rs2901585												
rs6478813												
rs318841												
rs1258464												
rs1433375												

**SNP**												
rs2401810												
rs803422												
rs2803543												
rs6068122												
rs365063												
rs2156208	TTAAGCTAACATATGAATGATGAGTAGGGTTTACCAGGGCCACATGGTAGGAGGAGAACATGCCAGGGAGATGGAAGAGCGAACATG											
rs1347423												

**SNP**												
rs1560434												

**SNP**									
rs2134180									
rs2180289									
rs1366660									
rs1825443									
rs8096868	TTTATGTATTGTAATTAACTTTAGAATTGGTGTTTCTAATCCCCAGTGCCAAAGAAATTCCCTGGAAG								
rs12466929									
rs846752									

SNP									
rs12472674									
rs4606154									
rs7292634									
rs273473									
rs2084851									
rs667415									
rs1486048									

**SNP**									
rs2730648									
rs1777467									
rs2835896									
rs12583473									
rs3899706									
rs4739199									
rs7855283									

**SNP**									
rs1607185									
rs4971653									
rs2840794									
rs12426585									
rs2938675									
rs3776720									
rs4306755	CAAGAATGTAATGACACAGTATAGTATTTCTACCTTATATGTGTCTCCCACTTTAACTTTTGCAAAGC								

**SNP**									
rs7693776									
rs2760396									
rs4933826	AATACTAATACATGCCGTATACACTCCACCCTCTCCCGGCCTCTCAAGTTGTTTGTAAACTCTATTTT								
rs592069									
rs2925067									
rs2071748									
rs7555995									

**SNP**									
rs4873622									
rs1891403									
rs2901585									
rs6478813									
rs318841									
rs1258464									
rs1433375									

SNP									
rs2401810									
rs803422									
rs2803543									
rs6068122									
rs365063									
rs2156208									
rs1347423									

**SNP**									
rs1560434									
