# Supplementary material for: Robust SNP genotyping by multiplex PCR and arrayed primer extension
Source: BMC Med Genomics. 2008 Jan 31;1:5. doi: 10.1186/1755-8794-1-5 (PMC2266772; doi:10.1186/1755-8794-1-5)
Supplement: Additional file 2 — PCR multiplex groups. Table that details the 7 groups of multiplex PCRs. [file 1755-8794-1-5-S2.pdf]

PCR Multiplex Group	SNP	PCR Amplicon Size (bp)
1	rs1258464	333
1	rs12472674	388
1	rs6478813	419
1	rs1560434	502
1	rs7693776	552
1	rs2840794	618
1	rs667415	717
2	rs1607185	279
2	rs1366660	315
2	rs1777467	485
2	rs2134180	556
2	rs1433375	580
2	rs2156208	684
2	rs4933826	736
3	rs4606154	432
3	rs592069	493
3	rs2730648	521
3	rs2901585	558
3	rs2401810	572
3	rs12426585	607
3	rs2938675	704
4	rs365063	159
4	rs7555995	205
4	rs3776720	389
4	rs1486048	454
4	rs12583473	502
4	rs318841	563
4	rs7855283	605
5	rs1347423	307
5	rs2760396	392
5	rs2803543	511
5	rs2071748	542
5	rs803422	550
5	rs846752	646
5	rs273473	707
6	rs2084851	216
6	rs7292634	295
6	rs6068122	383
6	rs1825443	486
6	rs2180289	551
6	rs4739199	587
6	rs1891403	685
6	rs4306755	787
7	rs4971653	388
7	rs3899706	460
7	rs4873622	483
7	rs12466929	550
7	rs2925067	585
7	rs2835896	685
7	rs8096868	785
