# Supplementary material for: Robust SNP genotyping by multiplex PCR and arrayed primer extension
Source: BMC Med Genomics. 2008 Jan 31;1:5. doi: 10.1186/1755-8794-1-5 (PMC2266772; doi:10.1186/1755-8794-1-5)
Supplement: Additional file 3 — Genotyping results from first experiment. Table that lists the complete genotyping results for 287 HapMap samples and 41 SNPs. Includes LDA call and MACGT call (with quality scores), as well as original HapMap call. [file 1755-8794-1-5-S3.pdf]

	MACGT discrepancy					
	LDA discrepancy					
NA	LDA output were not given for training cases					
XY	Manual calls are given in blue					
		**12426585**				
**Coriell**	**Sample**	**LDA Call**	**LDA quality score**	**MACGT Call**	**MACGT quality score**	**HapMap Call**
NA12753	1		NA	CT	0.561851836	
NA12875	2	CT	0.9893	ct	0.494426613	CT
NA12044	3	TT	0.9368	tt	0.630003097	TT
NA06993	4	TT	0.9835	tt	0.346084049	TT
NA12716	5	CC	0.9265	cc	0.157882087	CC
NA12760	6	TT	0.9832	tt	0.603313104	TT
NA07348	7	CC	1	cc	0.269668906	CC
NA12707	8	CT	0.9817	ct	0.292651545	CT
NA12156	9		NA	CC	0.322974845	
NA11992	10	TT	0.9703	tt	0.546303884	TT
NA12239	11	CC	0.984	cc	0.201668255	CC
NA12878	12	CC	0.9998	cc	0.144381471	CC
NA11993	13	TT	0.9905	tt	0.059568115	TT
NA12750	14	CC	0.9859	cc	0.347010313	CC
NA12146	15	TT	0.9284	tt	0.229475329	TT
NA11839	16	CT	0.9739	*CT*	*0*	CT
NA12156.dup	17	CC	0.9992	cc	0.194490583	nn
NA11829	18		NA	TT	0.399456629	
NA12154	19	CT	0.8203	ct	0.06487894	CT
NA10856	20	CT	0.9875	ct	0.070073054	CT
NA06985	21	TT	0.9594	tt	0.252198604	TT
NA11831	22	CT	0.978	*CT*	*0*	CT
NA12003.dup	23	TT	0.983	tt	0.191789381	nn
NA10859	24	CT	0.9839	ct	0.018661861	CT
NA07056	25	TT	0.9822	tt	0.282645737	TT
NA07022	26	CC	0.9909	cc	0.307488952	CC
NA12762	27		NA	CC	0.532023484	
NA12874	28	NN	0.718	tt	0.358663312	TT
NA12003	29	TT	0.9839	tt	0.351230074	TT
NA10835	30	CT	0.9811	ct	0.023344412	CT
NA11882	31	CC	0.9869	cc	0.333309209	CC
NA07034	32	NN	0.7104	tt	0.001567897	TT
NA12056	33	TT	0.9519	tt	0.470892124	TT
NA12145	34	TT	0.991	tt	0.340841517	TT
NA07019	35	CT	0.9871	ct	0.16077009	CT
NA06991	36		NA	TT	0.589888507	
NA12761	37	TT	0.9747	tt	0.264014675	TT
NA06994	38	TT	1	tt	0.300255471	TT
NA12864	39	CT	0.9825	ct	0.208982407	CT
NA07055	40	CT	0.9808	ct	0.186726036	CT
NA10863	41	CC	0.999	cc	0.298503288	CC
NA12763	42	CT	0.9845	ct	0.261994703	CT
NA10831	43	CC	0.9997	cc	0.265979738	CC
NA11840	44	TT	0.961	tt	0.13521162	TT

	MACGT discrepancy					
	LDA discrepancy					
NA	LDA output were not given for training cases					
XY	Manual calls are given in blue					
		**12426585**				
**Coriell**	**Sample**	**LDA Call**	**LDA quality score**	**MACGT Call**	**MACGT quality score**	**HapMap Call**
NA10830	45	CC	0.9947	cc	0.532707772	CC
NA11993.dup	46		NA	TT	0.370386799	
NA12751	47	TT	0.9815	tt	0.308490202	TT
NA12814	48	CT	0.9866	ct	0.066245168	CT
NA10857	49	TT	0.9931	tt	0.512741703	TT
NA07357	50	CC	0.9951	cc	0.30780029	CC
NA07000	51	TT	0.9981	tt	0.038624468	TT
NA12802	52	CT	0.9636	ct	0.009348362	CT
NA10855	53	CT	0.9864	ct	0.016778885	CT
NA11995	54		NA	TT	0.269161647	
NA10839	55	TT	0.8379	tt	0.018471674	TT
NA10847	56		NA	CT	0.350272153	
NA12006	57	CT	0.989	ct	0.116279633	CT
NA12144	58	CT	0.9842	ct	0.436213185	CT
NA11832	59	CT	0.9957	ct	0.123905898	CT
NA12813	60	CC	0.9639	cc	0.305036585	CC
blank	61	NN	0.7463	***NN***	***0***	nn
NA12891	62	CC	0.7787	cc	0.2521145	CC
NA12717	63		NA	CT	0.342434601	
NA10861	64	CT	0.9923	ct	0.278048839	CT
NA12004	65	CT	0.9908	ct	0.046880898	CT
NA07048	66	TT	0.9903	tt	0.22475981	TT
NA12801	67	CC	0.9167	cc	0.157730873	CC
NA11830	68	CC	0.9993	cc	0.212695528	CC
NA12057	69	TT	0.9414	tt	0.129527235	TT
NA11994	70		NA	CC	0.327210633	
NA12740	71	CT	0.9921	ct	0.090743159	CT
NA12005	72	CT	0.9682	ct	0.009214127	CT
NA07345	73	CC	1	cc	0.22478401	CC
NA10854	74	TT	0.9898	tt	0.157846233	TT
NA12872	75	CT	0.9805	ct	0.154520337	CT
NA12865	76	CT	0.9792	ct	0.354050584	CT
NA10860	77		NA	TT	0.185140164	
NA12873	78	CC	0.9997	cc	0.241533754	CC
NA12752	79	TT	0.9879	tt	0.516140486	TT
NA10851	80	TT	0.984	tt	0.3908847	TT
NA07029	81	TT	0.9831	tt	0.41009268	TT
NA12248	82	CT	0.9637	ct	0.06794917	CT
NA12155	83	CT	0.9829	ct	0.222587791	CT
NA10838	84	TT	0.9754	tt	0.17674554	TT
NA12812	85	CT	0.9846	ct	0.136785309	CT
NA12248.dup	86	CT	0.9809	ct	0.16339759	nn
NA12043	87	TT	0.9857	tt	0.534625686	TT
NA12234	88	CT	0.8501	ct	0.007589244	CT

	MACGT discrepancy					
	LDA discrepancy					
NA	LDA output were not given for training cases					
XY	Manual calls are given in blue					
		**12426585**				
**Coriell**	**Sample**	**LDA Call**	**LDA quality score**	**MACGT Call**	**MACGT quality score**	**HapMap Call**
NA12892	89	CC	0.9999	cc	0.307001671	CC
NA10846	90	CT	0.9789	ct	0.183137162	CT
NA06993.dup	91	TT	0.9802	tt	0.36079879	nn
NA12249	92	CT	0.9774	ct	0.038536211	CT
NA11881	93	TT	0.9345	tt	0.104587668	TT
NA12815	94	CT	0.9947	ct	0.161906769	CT
NA12264	95	CT	0.9676	ct	0.226160636	CT
NA18526	96	NN	0.7282	ct	0.17811461	CT
NA18562	97	CC	0.988	cc	0.065047629	CC
NA18545	98	TT	0.9326	tt	0.184234188	TT
NA18609	99	CT	0.9929	ct	0.223912448	CT
NA18566	100	TT	0.9583	tt	0.228128022	TT
NA18621	101	CC	0.9685	cc	0.374689098	CC
NA18577	102	NN	0.5899	ct	0.195528759	CT
NA18635	103	CT	0.9822	ct	0.13513785	CT
NA18524	104	CC	0.9999	cc	0.397310206	CC
NA18537	105	CT	0.9907	ct	0.099525784	CT
NA18572	106	CT	0.9901	ct	0.240525936	CT
NA18552	107	CT	0.9167	ct	0.043100263	CT
NA18563	108	CC	0.9972	cc	0.372984037	CC
NA18594	109	CC	0.9877	cc	0.198222712	nn
NA18624	110	CT	0.9874	ct	0.372082079	CT
NA18592	111	CT	0.9907	ct	0.211727572	CT
NA18529	112	TT	0.9115	tt	0.109182567	TT
NA18603	113	CC	0.9695	cc	0.348564671	nn
NA18547	114	TT	0.945	tt	0.4232141	TT
NA18611	115	CT	0.9952	ct	0.091824903	CT
NA18570	116	TT	0.785	tt	0.019131228	TT
NA18622	117	CT	0.985	ct	0.3168254	CT
NA18579	118	CC	0.7891	cc	0.028460746	CC
NA18636	119	CT	0.9857	ct	0.13421585	CT
NA18558	120	CT	0.9866	ct	0.28882553	CT
NA18540	121	CC	0.9999	cc	0.423625787	CC
NA18609	122	CT	0.9851	ct	0.222989039	nn
NA18555	123	NN	0.5203	***CC***	***0***	CC
NA18612	124	CC	0.9996	cc	0.276973372	CC
NA18573	125	CC	0.9989	cc	0.207461637	CC
NA18632	126	CC	0.9985	cc	0.105683698	CC
NA18593	127	CT	0.9684	ct	0.100311825	CT
NA18532	128	CC	0.996	cc	0.173675329	CC
NA18605	129	CC	0.9999	cc	0.092983071	CC
NA18550	130	CC	1	cc	0.31021892	CC
NA18603	131	CC	0.9994	cc	0.280119061	CC
NA18571	132	CT	0.9757	ct	0.054836701	CT

	MACGT discrepancy					
	LDA discrepancy					
NA	LDA output were not given for training cases					
XY	Manual calls are given in blue					
		**12426585**				
**Coriell**	**Sample**	**LDA Call**	**LDA quality score**	**MACGT Call**	**MACGT quality score**	**HapMap Call**
NA18623	133	CC	0.9997	cc	0.01329038	CC
NA18582	134	CC	0.7513	cc	0.457157226	CC
NA18637	135	CC	0.7513	cc	0.269364756	CC
NA18561	136	CT	0.9831	ct	0.293496509	CT
NA18542	137	CT	0.9817	ct	0.225433248	CT
NA18608	138	CT	0.9824	ct	0.099730281	CT
NA18564	139	TT	0.9944	tt	0.427591494	TT
NA18620	140	CC	0.9996	cc	0.315845649	CC
NA18576	141	CC	0.9999	cc	0.349445108	CC
NA18633	142	CC	0.9992	cc	0.069710808	CC
NA18594	143	CC	0.998	cc	0.451215957	CC
NA18942	144	CT	0.9797	ct	0.184353272	CT
NA18945	145	TT	0.9418	tt	0.503437395	TT
NA18964	146	CT	0.9875	ct	0.20637779	CT
NA18961	147	CC	0.916	cc	0.009032243	CC
NA18967	148	CC	0.9928	cc	0.485487061	CC
NA18981	149	CC	0.9927	cc	0.001449058	CC
NA18994	150	TT	0.9965	tt	0.452721476	TT
NA18998	151	CT	0.9643	ct	0.067780609	CT
NA18940	152	CT	0.948	ct	0.075626307	CT
NA18949	153	CC	0.9994	cc	0.195030208	CC
NA18953	154	CT	0.9871	ct	0.040912784	CT
NA18972	155	CC	0.9551	cc	0.209951071	CC
NA18976	156	CT	0.9772	ct	0.173878959	CT
NA18971	157	TT	0.9871	tt	0.450058273	TT
blank	158	NN	0.5001	*NN*	*0*	nn
NA19000	159	NN	0.7294	ct	0.268151429	CT
NA18951	160	NN	0.7241	ct	0.280504885	nn
NA18948	161	NN	0.732	ct	0.250930286	CT
NA18968	162	NN	0.7379	ct	0.213832399	CT
NA18965	163	NN	0.7496	cc	0.009811971	CC
NA18978	164	NN	0.7308	*CT*	*0*	CT
NA18974	165	NN	0.7317	*CT*	*0*	CT
NA18992	166	NN	0.7144	tt	0.343530387	TT
NA19005	167	CT	0.9711	ct	0.206184765	CT
NA18943	168	CC	0.9541	cc	0.189260253	CC
NA18951	169	CT	0.9785	ct	0.092650979	CT
NA18959	170	TT	0.9997	tt	0.53969134	TT
NA18973	171	TT	0.9334	tt	0.39373468	TT
NA18970	172	CC	0.9976	cc	0.514109377	CC
NA18987	173	CC	0.9993	cc	0.440058785	CC
NA18995	174	CT	0.9619	ct	0.397596025	CT
NA18999	175	CC	0.9996	cc	0.589577233	CC
NA18947	176	CT	0.9716	ct	0.205170726	CT

	MACGT discrepancy					
	LDA discrepancy					
NA	LDA output were not given for training cases					
XY	Manual calls are given in blue					
		**12426585**				
**Coriell**	**Sample**	**LDA Call**	**LDA quality score**	**MACGT Call**	**MACGT quality score**	**HapMap Call**
NA18952	177	TT	0.9864	tt	0.323302422	TT
NA18969	178	CT	0.9617	***CT***	***0***	CT
NA18966	179	TT	0.9709	tt	0.187556833	TT
NA18980	180	CC	0.9959	cc	0.427863228	CC
NA18990	181	CT	0.9595	ct	0.083109818	CT
NA18997	182	TT	0.9641	tt	0.194583925	TT
NA19007	183	CT	0.9657	ct	0.169050089	CT
NA18944	184	NN	0.7467	ct	0.04441569	CT
NA18956	185	CC	0.9981	cc	0.422587117	CC
NA18960	186	CT	0.9654	ct	0.317960744	CT
NA18975	187	TT	0.997	tt	0.266429801	TT
NA18995	188	CT	0.9687	ct	0.532214541	nn
NA18991	189	CC	0.9997	cc	0.541748284	CC
NA18996	190	CT	0.9659	ct	0.003170048	nn
NA19003	191	CT	0.9867	ct	0.381023532	CT
NA18502	192	NN	0.604	***TT***	***0***	TT
NA19153	193	CC	0.7742	***CC***	***0***	CC
NA18857	194	CT	0.9635	ct	0.107062065	CT
NA19223	195	CC	0.8543	***CC***	***0***	CC
NA19201	196	NN	0.7401	***CT***	***0***	CT
NA18504	197	CC	0.9814	***CC***	***0***	CC
NA18870	198	CC	0.9532	***CC***	***0***	CC
NA18863	199	CT	0.992	***CT***	***0***	CT
NA19145	200	CT	0.8708	***CT***	***0***	CT
NA19137	201	NN	0.7464	***CT***	***5.69E-04***	CT
NA19238	202	CT	0.9697	***CT***	***7.75E-04***	nn
NA18500	203	CT	0.9462	***TT***	***0***	TT
NA19144	204	NN	0.7426	***CC***	***0***	CC
NA19203	205	CC	0.9997	cc	0.384983597	CC
NA19200	206	CC	0.9748	***CC***	***0***	CC
NA18855	207	CT	0.7568	***CT***	***0***	CT
NA18505	208	CC	0.9658	***CC***	***0***	CC
NA19202	209	CC	0.9988	***CC***	***0***	CC
NA18501	210	NN	0.6439	***TT***	***0***	TT
NA18861	211	CT	0.8778	***TT***	***0***	TT
NA19193	212	CC	0.9982	***CC***	***6.47E-04***	CC
NA19143	213	NN	0.7319	***CT***	***0***	CT
NA18517	214	CT	0.9156	ct	0.044309009	CT
NA18862	215	CT	0.9785	***CT***	***0***	CT
NA18856	216	CT	0.9438	ct	0.043286585	nn
NA19239	217	CT	0.9362	ct	0.091496196	CT
NA19240	218	TT	0.9864	tt	0.536329842	TT
NA18856	219	CT	0.9891	ct	0.043180016	CT
NA18503	220	CC	0.9977	cc	0.008394758	CC

	MACGT discrepancy					
	LDA discrepancy					
NA	LDA output were not given for training cases					
XY	Manual calls are given in blue					
		12426585				
**Coriell**	**Sample**	**LDA Call**	**LDA quality score**	**MACGT Call**	**MACGT quality score**	**HapMap Call**
NA18871	221	CT	0.9872	ct	0.079598425	CT
NA19221	222	CC	0.9909	cc	0.009669605	CC
NA19209	223	CT	0.9855	ct	0.0044757	CT
NA19152	224	CC	0.9653	cc	0.151704681	CC
NA18515	225	CC	0.817	cc	0.037388781	CC
NA19238	226	CT	0.9833	ct	0.149027773	CT
NA19154	227	CC	0.9975	cc	0.643284015	CC
NA19210	228	CC	0.9208	cc	0.019896314	CC
NA19211	229	CT	0.9796	ct	0.083013811	CT
NA18862	230	CT	0.9171	ct	0.06605235	nn
NA18872	231	CT	0.9754	ct	0.087958094	CT
NA19139	232	TT	0.9363	tt	0.376269897	TT
NA19222	233	CC	0.9504	cc	0.08744552	CC
NA19194	234	CC	0.9997	cc	0.535253343	CC
NA19138	235	CT	0.9824	ct	0.020596724	CT
NA19204	236	CC	0.9251	*CC*	*0*	CC
NA18516	237	CC	0.9621	*CC*	*0*	CC
NA19205	238	CC	0.9805	cc	0.067925049	CC
NA19192	239	CT	0.9925	ct	0.028460871	CT
NA18912	240	CT	0.9688	ct	0.156460765	nn
NA18508	241	CC	0.9945	cc	0.189876623	CC
NA19142	242	CC	0.977	*CC*	*0*	CC
NA18852	243	CC	1	cc	0.283333893	CC
NA18507	244	CT	0.986	ct	0.255847843	CT
blank	245	NN	0.6868	*NN*	*0*	nn
NA19101	246	CC	0.9704	cc	0.268652639	CC
NA19172	247	CT	0.9793	ct	0.106294133	CT
NA19160	248	CT	0.9618	ct	0.006919253	CT
NA19129	249	CT	0.9664	ct	0.044665081	CT
NA18913	250	CT	0.9773	ct	0.161749232	CT
NA19120	251	CT	0.9661	ct	0.007494676	CT
NA19159	252	CC	0.9795	cc	0.07034456	CC
NA18523	253	TT	0.9936	tt	0.472333073	TT
NA19102	254	CC	0.932	*CC*	*5.02E-04*	CC
NA19092	255	CT	0.982	ct	0.30149145	CT
NA18521	256	CT	0.988	ct	0.122529108	CT
NA18912	257	CT	0.9875	ct	0.007292687	CT
NA19206	258	CT	0.9738	ct	0.30425014	CT
NA19128	259	TT	0.8962	tt	0.292262386	TT
NA18859	260	CC	0.9629	cc	0.014458532	nn
NA19094	261	NN	0.7251	ct	0.318662582	CT
NA19171	262	CC	0.9747	*CC*	*0*	CC
NA18860	263	CT	0.9928	ct	0.133573315	CT
NA18858	264	CT	0.9816	ct	0.371665037	CT

	MACGT discrepancy					
	LDA discrepancy					
NA	LDA output were not given for training cases					
XY	Manual calls are given in blue					
		**12426585**				
**Coriell**	**Sample**	**LDA Call**	**LDA quality score**	**MACGT Call**	**MACGT quality score**	**HapMap Call**
NA19099	265	CT	0.9909	ct	0.13366506	CT
NA18506	266	NN	0.7353	***CC***	***0***	CC
NA19131	267	CC	0.9942	cc	0.246121323	CC
NA19116	268	CT	0.986	ct	0.19812992	CT
NA19161	269	CC	0.9115	cc	0.098601224	CC
NA19140	270	CC	0.9826	cc	0.231699972	CC
NA19207	271	CC	0.9993	cc	0.087163495	CC
NA19141	272	CC	0.9787	cc	0.226080309	CC
NA18854	273	CT	0.9917	ct	0.223961358	CT
NA19098	274	CC	0.9359	cc	0.042205115	CC
NA18522	275	CC	0.8744	cc	0.141157342	CC
NA18914	276	NN	0.7089	***CC***	***0***	CC
NA18853	277	CT	0.97	ct	0.169782756	CT
NA18859	278	CC	0.9814	cc	0.281138792	CC
NA19100	279	CC	0.9615	cc	0.253608308	CC
NA19093	280	CT	0.9381	ct	0.064201792	CT
NA19132	281	CT	0.9882	ct	0.066858943	CT
NA19130	282	CT	0.9924	ct	0.104555195	CT
NA19208	283	CC	0.8373	***CC***	***8.52E-04***	CC
NA19127	284	CT	0.9816	ct	0.05065533	CT
NA19173	285	CC	0.9952	cc	0.268866769	CC
NA19119	286	CT	0.9896	ct	0.168252115	CT
NA19103	287	CC	0.9602	cc	0.119514584	CC
	Discrepancies	2		0		
	NNs	24		1		
	Automated Calls	276		238		
	Manual Calls	0		38		
	Concordant Calls	234		258		258

	MACGT discrepancy					
	LDA discrepancy					
NA	LDA output were not g					
XY	Manual calls are given					
		**12466929**				
**Coriell**	**Sample**	**LDA Call**	**LDA quality score**	**MACGT Call**	**MACGT quality score**	**HapMap Call**
NA12753	1		NA	GG	0.558355207	
NA12875	2	GG	0.998	gg	0.601700534	GG
NA12044	3	GG	0.9998	gg	0.347332443	GG
NA06993	4	AG	0.9959	ag	0.239230246	AG
NA12716	5	AG	0.9923	ag	0.253755345	AG
NA12760	6	GG	0.997	gg	0.310023927	GG
NA07348	7	GG	0.9994	gg	0.301019141	GG
NA12707	8	AG	0.9759	ag	0.206294281	AG
NA12156	9		NA	AG	0.219815223	
NA11992	10	GG	0.9988	gg	0.072981184	GG
NA12239	11	GG	0.9765	gg	0.101253415	GG
NA12878	12	GG	0.9382	gg	0.015408246	GG
NA11993	13	GG	0.9999	***gg***	***9.96E-04***	GG
NA12750	14	AG	0.9777	ag	0.207196407	AG
NA12146	15	GG	0.9954	gg	0.202901411	GG
NA11839	16	GG	0.9999	gg	0.010147635	GG
NA12156.dup	17	AG	0.997	ag	0.185692983	nn
NA11829	18		NA	GG	0.317255302	
NA12154	19	AG	0.9921	ag	0.271139942	AG
NA10856	20	GG	1	gg	0.441269647	GG
NA06985	21	GG	1	gg	0.297586407	GG
NA11831	22	AG	0.9999	ag	0.001133981	AG
NA12003.dup	23	GG	0.9337	gg	0.180013747	nn
NA10859	24	GG	0.9842	gg	0.25543158	GG
NA07056	25	GG	0.9994	gg	0.476172231	GG
NA07022	26	GG	0.9668	gg	0.449721814	GG
NA12762	27		NA	GG	0.411724539	
NA12874	28	AG	0.9991	***AG***	***0***	AG
NA12003	29	GG	0.9999	gg	0.463966083	GG
NA10835	30	GG	0.9999	gg	0.36390917	GG
NA11882	31	AG	0.9977	ag	0.187407682	AG
NA07034	32	GG	1	gg	0.313224537	GG
NA12056	33	GG	0.9995	gg	0.239442324	GG
NA12145	34	GG	0.9805	gg	0.300393375	GG
NA07019	35	GG	0.9849	gg	0.472430969	GG
NA06991	36		NA	AG	0.422918427	
NA12761	37	GG	0.9923	gg	0.449781264	GG
NA06994	38	GG	0.9818	gg	0.406154473	GG
NA12864	39	GG	0.9915	gg	0.558046895	GG
NA07055	40	GG	0.9994	gg	0.519958467	GG
NA10863	41	GG	0.989	gg	0.416797299	GG
NA12763	42	GG	0.9936	gg	0.545380513	GG
NA10831	43	AG	0.9984	ag	0.249590968	AG
NA11840	44	GG	0.9975	gg	0.60561906	GG

	MACGT discrepancy					
	LDA discrepancy					
NA	LDA output were not g					
XY	Manual calls are given					
		**12466929**				
**Coriell**	**Sample**	**LDA Call**	**LDA quality score**	**MACGT Call**	**MACGT quality score**	**HapMap Call**
NA10830	45	AG	0.9967	ag	0.214483414	AG
NA11993.dup	46	GG	0.9993	gg	0.231868738	nn
NA12751	47	GG	0.9894	gg	0.452240683	GG
NA12814	48	GG	0.9565	gg	0.327537939	GG
NA10857	49	GG	0.9987	gg	0.414544869	GG
NA07357	50	AG	0.9955	ag	0.313696977	AG
NA07000	51	GG	0.9942	gg	0.543324773	GG
NA12802	52	GG	0.9784	gg	0.292506905	GG
NA10855	53	GG	0.9065	gg	0.13403522	GG
NA11995	54		NA	GG	0.251475195	
NA10839	55	GG	0.979	gg	0.242661288	GG
NA10847	56	GG	0.9634	gg	0.418657924	GG
NA12006	57	GG	0.9798	gg	0.42105705	GG
NA12144	58	GG	0.997	gg	0.06489495	GG
NA11832	59	GG	0.9567	gg	0.388555176	GG
NA12813	60	GG	0.9938	gg	0.058251947	GG
blank	61	NN	0.5	*NN*	*0*	nn
NA12891	62	GG	0.9974	gg	0.431301569	GG
NA12717	63		NA	GG	0.410221527	
NA10861	64	GG	0.9873	gg	0.066822292	GG
NA12004	65	AG	0.9523	ag	0.15296068	AG
NA07048	66	GG	0.9971	gg	0.317684452	GG
NA12801	67	GG	0.9997	gg	0.205680641	GG
NA11830	68	GG	0.9988	gg	0.264182966	GG
NA12057	69	GG	0.9934	gg	0.167433	GG
NA11994	70		NA	GG	0.512500852	
NA12740	71	AG	0.9986	ag	0.493609176	AG
NA12005	72	NN	0.6992	*GG*	*0*	GG
NA07345	73	NN	0.4997	*NN*	*0*	GG
NA10854	74	GG	0.898	*GG*	*0*	GG
NA12872	75	NN	0.7045	*GG*	*0*	GG
NA12865	76	GG	0.8269	gg	0.001729748	GG
NA10860	77		NA	GG	0.404820251	
NA12873	78	GG	0.9611	*GG*	*0*	GG
NA12752	79	GG	0.7527	*GG*	*0*	GG
NA10851	80	GG	0.9999	*GG*	*0*	GG
NA07029	81	NN	0.75	*GG*	*0*	GG
NA12248	82	AG	0.7893	*AG*	*0*	AG
NA12155	83	GG	0.9765	*GG*	*0*	GG
NA10838	84	NN	0.6167	*AG*	*0*	AG
NA12812	85	GG	0.921	*GG*	*0*	GG
NA12248.dup	86	NN	0.742	*NN*	*0*	nn
NA12043	87	NN	0.5445	*NN*	*0*	GG
NA12234	88	GG	1	gg	0.356364205	GG

	MACGT discrepancy					
	LDA discrepancy					
NA	LDA output were not g					
XY	Manual calls are given					
		12466929				
**Coriell**	**Sample**	**LDA Call**	**LDA quality score**	**MACGT Call**	**MACGT quality score**	**HapMap Call**
NA12892	89	NN	0.5	***NN***	***0***	AG
NA10846	90	GG	0.7719	gg	0.001757029	GG
NA06993.dup	91	AG	0.9977	***AG***	***0***	nn
NA12249	92	NN	0.5001	***NN***	***0***	GG
NA11881	93	GG	0.9096	***GG***	***0***	GG
NA12815	94	GG	0.9024	***GG***	***0***	GG
NA12264	95	AG	0.9842	***AG***	***0***	AG
NA18526	96		NA	AA	0.453785131	
NA18562	97	GG	0.9939	gg	0.584485769	GG
NA18545	98	AG	0.9976	ag	0.306064054	AG
NA18609	99		NA	AA	0.628596872	
NA18566	100	AG	0.9858	ag	0.113643542	AG
NA18621	101	AA	1	aa	0.655068321	AA
NA18577	102		NA	AA	0.55940512	
NA18635	103	AA	1	aa	0.493683559	AA
NA18524	104	GG	0.9994	gg	0.603469825	GG
NA18537	105	GG	0.9995	gg	0.627493045	GG
NA18572	106	AA	1	aa	0.498680026	AA
NA18552	107	AG	0.9993	ag	0.106970517	AG
NA18563	108	AG	0.9988	ag	0.277041461	AG
NA18594	109	AG	0.9992	ag	0.254439706	nn
NA18624	110	GG	0.9996	gg	0.552964655	GG
NA18592	111		NA	AA	0.557662475	
NA18529	112	AA	1	aa	0.40283692	AA
NA18603	113	AA	1	aa	0.390467599	nn
NA18547	114	AG	0.9981	ag	0.369354403	AG
NA18611	115	AA	1	aa	0.345774771	AA
NA18570	116	AA	1	aa	0.340571432	AA
NA18622	117	AA	1	aa	0.222257714	AA
NA18579	118	AA	1	aa	0.289024193	AA
NA18636	119	AG	0.9026	ag	0.195490862	AG
NA18558	120	AA	1	aa	0.196662004	AA
NA18540	121	AA	1	aa	0.102426896	AA
NA18609	122	AA	1	aa	0.047728546	nn
NA18555	123	AG	0.9437	***AG***	***0***	AG
NA18612	124	GG	1	gg	0.565953361	GG
NA18573	125	AG	0.986	***AG***	***0***	AG
NA18632	126	AA	0.9996	aa	0.103019813	AA
NA18593	127	AA	0.996	aa	0.018411894	AA
NA18532	128	GG	0.9665	gg	0.038800768	GG
NA18605	129	AG	0.9939	***AG***	***0***	AG
NA18550	130	GG	0.7702	gg	0.001233769	GG
NA18603	131	AA	1	aa	0.297154353	AA
NA18571	132	AA	1	aa	0.188050478	AA

	MACGT discrepancy					
	LDA discrepancy					
NA	LDA output were not g					
XY	Manual calls are given					
		**12466929**				
**Coriell**	**Sample**	**LDA Call**	**LDA quality score**	**MACGT Call**	**MACGT quality score**	**HapMap Call**
NA18623	133	AG	0.7587	***AG***	***0***	AG
NA18582	134	AG	0.8587	ag	0.118461931	AG
NA18637	135	AG	0.7952	ag	0.073948325	AG
NA18561	136	AG	0.9001	***AG***	***0***	AG
NA18542	137	AA	1	aa	0.023110898	AA
NA18608	138	AA	1	aa	0.299666973	AA
NA18564	139	GG	0.9346	***GG***	***0***	GG
NA18620	140	AG	0.9822	***AG***	***0***	AG
NA18576	141		NA	AA	0.13325417	
NA18633	142	AG	0.8994	***AG***	***0***	AG
NA18594	143	AG	0.9913	***AG***	***0***	AG
NA18942	144	AG	0.9583	ag	0.222357226	AG
NA18945	145	AG	0.9601	ag	0.249462748	AG
NA18964	146	AG	0.9738	ag	0.303240043	AG
NA18961	147	GG	0.9971	gg	0.036419417	GG
NA18967	148	AG	0.9965	ag	0.504190546	AG
NA18981	149	GG	0.9999	gg	0.397261291	GG
NA18994	150	AG	0.9902	ag	0.273239075	AG
NA18998	151	GG	0.9958	gg	0.017441666	GG
NA18940	152	AG	0.9775	ag	0.020917947	AG
NA18949	153	AG	0.9032	ag	0.023593867	AG
NA18953	154	AG	0.8952	ag	0.005567856	AG
NA18972	155	GG	1	gg	0.039778872	GG
NA18976	156	AA	1	aa	0.286760504	AA
NA18971	157	AG	0.9201	ag	0.0290291	AG
blank	158	NN	0.5	***NN***	***0***	nn
NA19000	159	AA	1	aa	0.307516684	AA
NA18951	160	GG	1	gg	0.395260908	nn
NA18948	161	AG	0.7964	ag	0.045114344	AG
NA18968	162	NN	0.6922	ag	0.063585509	AG
NA18965	163	GG	1	gg	0.307579157	GG
NA18978	164	AG	0.8879	ag	0.133055877	AG
NA18974	165	NN	0.7497	ag	0.039519953	AG
NA18992	166	AA	1	aa	0.188885656	AA
NA19005	167	GG	0.9923	gg	0.465430432	GG
NA18943	168	AA	1	aa	0.071972303	AA
NA18951	169	GG	0.985	***GG***	***0***	GG
NA18959	170	AA	1	aa	0.274310093	AA
NA18973	171	AA	1	aa	0.374241292	AA
NA18970	172	AA	1	aa	0.232985008	AA
NA18987	173	AA	1	aa	0.338734613	AA
NA18995	174	AG	0.9638	ag	0.27647705	AG
NA18999	175	GG	0.9993	gg	0.661705038	GG
NA18947	176	AG	0.7825	ag	0.059575943	AG

	MACGT discrepancy					
	LDA discrepancy					
NA	LDA output were not g					
XY	Manual calls are given					
		**12466929**				
**Coriell**	**Sample**	**LDA Call**	**LDA quality score**	**MACGT Call**	**MACGT quality score**	**HapMap Call**
NA18952	177	AG	0.9348	ag	0.236783584	AG
NA18969	178	AG	0.8882	ag	0.106312782	AG
NA18966	179	GG	0.9998	gg	0.500932081	GG
NA18980	180	AA	1	aa	0.392060193	AA
NA18990	181	AG	0.9209	ag	0.050218773	AG
NA18997	182	AG	0.9353	ag	0.110660984	AG
NA19007	183	AG	0.9697	ag	0.085128028	AG
NA18944	184	AG	0.9683	ag	0.029478533	AG
NA18956	185	AA	1	aa	0.292511954	AA
NA18960	186	GG	0.9993	gg	0.404814627	GG
NA18975	187	AG	0.9895	ag	0.078550131	AG
NA18995	188	AG	0.9782	ag	0.218495706	nn
NA18991	189	AG	0.9829	ag	0.516028051	AG
NA18996	190	AG	0.9789	ag	0.273938646	nn
NA19003	191	AG	0.9857	ag	0.221081854	AG
NA18502	192	AG	0.9925	ag	0.081976313	AG
NA19153	193	GG	0.9919	gg	0.505561448	GG
NA18857	194	AG	0.9417	ag	0.211952364	AG
NA19223	195	AG	0.9224	ag	0.122664549	AG
NA19201	196	GG	0.9992	gg	0.520726031	GG
NA18504	197	GG	0.8641	gg	0.059765333	GG
NA18870	198	AA	1	aa	0.540904295	AA
NA18863	199	GG	0.9998	gg	0.453959052	GG
NA19145	200	GG	0.995	gg	0.524232983	GG
NA19137	201	GG	0.9996	gg	0.34246995	GG
NA19238	202	AG	0.9158	ag	0.092425301	nn
NA18500	203	AG	0.9592	ag	0.046913444	AG
NA19144	204	GG	0.992	gg	0.190209645	GG
NA19203	205	GG	1	gg	0.396925954	GG
NA19200	206	GG	0.9981	gg	0.552111331	GG
NA18855	207	AG	0.9382	ag	0.43373612	AG
NA18505	208	AG	0.9823	ag	0.127936305	AG
NA19202	209	GG	0.9999	gg	0.413026436	GG
NA18501	210	GG	0.9998	gg	0.35396994	GG
NA18861	211	NN	0.7366	ag	0.002701457	AG
NA19193	212	GG	1	gg	0.287765226	GG
NA19143	213	NN	0.7175	ag	0.053585372	AG
NA18517	214	GG	0.9999	gg	0.244394726	GG
NA18862	215	AG	0.8811	ag	0.02782554	AG
NA18856	216	AG	0.9468	ag	0.043655365	nn
NA19239	217	AG	0.9086	ag	0.006839124	AG
NA19240	218	AG	0.9788	ag	0.144831254	AG
NA18856	219	AG	0.9795	ag	0.065189375	AG
NA18503	220	GG	0.9809	gg	0.45409486	GG

	MACGT discrepancy					
	LDA discrepancy					
NA	LDA output were not g					
XY	Manual calls are given					
		**12466929**				
**Coriell**	**Sample**	**LDA Call**	**LDA quality score**	**MACGT Call**	**MACGT quality score**	**HapMap Call**
NA18871	221	AG	0.9639	ag	0.223396477	AG
NA19221	222	AA	1	aa	0.329373368	AA
NA19209	223	AA	1	aa	0.178880387	AA
NA19152	224	GG	0.9788	gg	0.243981879	GG
NA18515	225	GG	0.9938	gg	0.256158133	GG
NA19238	226	AG	0.9683	ag	0.185358479	AG
NA19154	227	GG	0.996	gg	0.661540432	GG
NA19210	228	GG	0.9982	gg	0.343252105	GG
NA19211	229	AG	0.8968	ag	0.290279945	AG
NA18862	230	AG	0.9464	ag	0.169447841	nn
NA18872	231	AG	0.9468	ag	0.372587766	AG
NA19139	232	GG	0.9989	gg	0.656816878	GG
NA19222	233	AG	0.9749	ag	0.078587383	AG
NA19194	234	GG	0.9991	gg	0.395024897	GG
NA19138	235	GG	0.9999	gg	0.285765074	GG
NA19204	236	GG	0.9989	gg	0.598400593	GG
NA18516	237	GG	0.9692	gg	0.16559304	GG
NA19205	238	GG	0.9814	gg	0.234566677	GG
NA19192	239	AG	0.9551	ag	0.025317147	AG
NA18912	240	AG	0.987	ag	0.13738678	nn
NA18508	241	GG	0.9553	gg	0.259156833	GG
NA19142	242	GG	0.9163	gg	0.059773643	GG
NA18852	243	AG	0.9712	ag	0.19856012	AG
NA18507	244	AG	0.9781	ag	0.085236459	AG
blank	245	NN	0.5	***NN***	***0***	nn
NA19101	246	GG	0.9978	gg	0.035207737	GG
NA19172	247	GG	0.9987	gg	0.354254228	GG
NA19160	248	GG	0.9934	gg	0.186690196	GG
NA19129	249	AG	0.9823	ag	0.028507273	AG
NA18913	250	GG	0.9927	gg	0.172357246	GG
NA19120	251	GG	0.9993	gg	0.086771399	GG
NA19159	252	GG	0.9984	gg	0.089022953	GG
NA18523	253	AG	0.9538	ag	0.15060484	AG
NA19102	254	AG	0.9979	***AG***	***6.56E-04***	AG
NA19092	255	GG	0.9978	gg	0.00884544	GG
NA18521	256	AA	1	aa	0.208154975	AA
NA18912	257	AG	0.9958	ag	0.00944401	AG
NA19206	258	GG	0.9859	***GG***	***0***	GG
NA19128	259	AG	0.9984	ag	0.213814365	AG
NA18859	260	AG	0.9983	ag	0.267461382	nn
NA19094	261	GG	0.9904	gg	0.451924516	GG
NA19171	262	AG	0.9957	ag	0.311947762	AG
NA18860	263	AG	0.9981	ag	0.122203847	AG
NA18858	264	GG	0.9862	gg	0.357663863	GG

	MACGT discrepancy					
	LDA discrepancy					
NA	LDA output were not g					
XY	Manual calls are given					
		12466929				
**Coriell**	**Sample**	**LDA Call**	**LDA quality score**	**MACGT Call**	**MACGT quality score**	**HapMap Call**
NA19099	265	AG	0.998	ag	0.389277537	AG
NA18506	266	GG	0.9937	gg	0.432489055	GG
NA19131	267	GG	0.9982	gg	0.372693316	GG
NA19116	268	AG	0.99	ag	0.225761146	AG
NA19161	269	GG	0.9933	gg	0.43583813	GG
NA19140	270	AG	0.997	ag	0.175601234	AG
NA19207	271	AG	0.8951	ag	0.010309769	AG
NA19141	272	AG	0.9869	ag	0.173049307	AG
NA18854	273	GG	0.9793	gg	0.387797258	GG
NA19098	274	GG	0.9962	gg	0.485188136	GG
NA18522	275	AA	1	aa	0.210096944	AA
NA18914	276	AG	0.9358	ag	0.044288886	AG
NA18853	277	GG	0.9999	gg	0.296364773	GG
NA18859	278	AG	0.9924	ag	0.181776156	AG
NA19100	279	AG	0.9958	ag	0.021117728	AG
NA19093	280	GG	0.9834	gg	0.408357399	GG
NA19132	281	GG	0.8633	gg	0.012376995	GG
NA19130	282	GG	0.9921	gg	0.237188026	GG
NA19208	283	GG	0.9845	gg	0.202387249	GG
NA19127	284	GG	1	gg	0.301188262	GG
NA19173	285	GG	0.9996	gg	0.495732384	GG
NA19119	286	GG	0.9855	gg	0.261937127	GG
NA19103	287	AG	0.9983	ag	0.374682533	AG
	Discrepancies	0		0		
	NNs	14		6		
	Automated Calls	273		236		
	Manual Calls	0		37		
	Concordant Calls	242		250		254

	MACGT discrepancy					
	LDA discrepancy					
NA	LDA output were not g					
XY	Manual calls are given					
		**12472674**				
**Coriell**	**Sample**	**LDA Call**	**LDA quality score**	**MACGT Call**	**MACGT quality score**	**HapMap Call**
NA12753	1		NA	TT	0.418214689	
NA12875	2	TT	1	tt	0.447359792	TT
NA12044	3	CT	0.9997	ct	0.21846959	CT
NA06993	4	CT	0.9999	ct	0.486997674	CT
NA12716	5	CT	1	ct	0.351522739	CT
NA12760	6	CT	0.9999	ct	0.312379613	CT
NA07348	7	TT	0.9996	tt	0.389143715	TT
NA12707	8	CT	0.9999	ct	0.035557232	CT
NA12156	9		NA	CT	0.123898208	
NA11992	10	CC	1	cc	0.068697713	CC
NA12239	11	CT	0.9966	ct	0.091920051	CT
NA12878	12	TT	0.9966	tt	0.09619398	TT
NA11993	13	CT	1	ct	0.033329966	CT
NA12750	14	TT	1	tt	0.074606536	TT
NA12146	15	TT	0.9999	tt	0.108098296	TT
NA11839	16	CC	0.9997	cc	0.125723848	CC
NA12156.dup	17	CT	0.9992	ct	0.257193965	nn
NA11829	18		NA	TT	0.333545462	
NA12154	19	CT	0.9999	ct	0.045641162	CT
NA10856	20	TT	1	tt	0.254507655	TT
NA06985	21	CC	1	cc	0.065681748	CC
NA11831	22	CC	0.75	***CC***	***0***	CC
NA12003.dup	23	CT	1	ct	0.088414305	nn
NA10859	24	TT	0.9984	tt	0.165608543	TT
NA07056	25	TT	0.9877	tt	0.161955139	TT
NA07022	26	TT	1	tt	0.380719785	TT
NA12762	27		NA	TT	0.345607178	
NA12874	28	TT	0.991	tt	0.208207921	TT
NA12003	29	CT	1	ct	0.450859046	CT
NA10835	30	CT	1	ct	0.316640792	CT
NA11882	31	TT	1	tt	0.304821677	TT
NA07034	32	TT	1	tt	0.336714596	TT
NA12056	33	TT	1	tt	0.478523094	TT
NA12145	34	CC	0.8229	***CC***	***0***	CC
NA07019	35	TT	1	tt	0.444841756	TT
NA06991	36		NA	CT	0.458488874	
NA12761	37	CT	1	ct	0.362077252	CT
NA06994	38	CT	1	ct	0.22004544	CT
NA12864	39	CT	1	ct	0.540700562	CT
NA07055	40	CT	1	ct	0.372503587	CT
NA10863	41	CT	1	ct	0.635363305	CT
NA12763	42	TT	1	tt	0.377089473	TT
NA10831	43	CT	1	ct	0.361374763	CT
NA11840	44	TT	1	tt	0.314282586	TT

	MACGT discrepancy					
	LDA discrepancy					
NA	LDA output were not g					
XY	Manual calls are given					
				**12472674**		
**Coriell**	**Sample**	**LDA Call**	**LDA quality score**	**MACGT Call**	**MACGT quality score**	**HapMap Call**
NA10830	45	CC	1	cc	0.366836349	CC
NA11993.dup	46	CT	1	ct	0.31675379	nn
NA12751	47	CT	1	ct	0.62377064	CT
NA12814	48	CC	0.9997	cc	0.129607057	CC
NA10857	49	CT	1	ct	0.339516735	CT
NA07357	50	CT	1	ct	0.755242926	CT
NA07000	51	CT	1	ct	0.322313041	CT
NA12802	52	CT	1	ct	0.103169364	CT
NA10855	53	CC	0.7502	***CC***	***0***	CC
NA11995	54		NA	CT	0.129210906	
NA10839	55	CT	1	ct	0.235356525	CT
NA10847	56	CT	1	ct	0.138897822	CT
NA12006	57	TT	1	tt	0.500051346	TT
NA12144	58	CC	0.9993	cc	0.200556944	CC
NA11832	59	CC	0.7704	cc	0.014756669	CC
NA12813	60	CT	1	ct	0.317252894	CT
blank	61	NN	0.6891	***NN***	***0***	nn
NA12891	62	TT	1	tt	0.429898351	TT
NA12717	63		NA	TT	0.440020822	
NA10861	64	CC	0.9858	***CC***	***0***	CC
NA12004	65		NA	TT	0.364500251	
NA07048	66	TT	1	tt	0.369170838	TT
NA12801	67	TT	1	tt	0.167460472	TT
NA11830	68	CT	0.9999	ct	0.268907742	CT
NA12057	69	TT	1	tt	0.254695072	TT
NA11994	70		NA	CT	0.427609906	
NA12740	71	TT	1	tt	0.644242362	TT
NA12005	72	CT	1	ct	0.070662	CT
NA07345	73	TT	0.9999	tt	0.051028011	TT
NA10854	74	CT	1	ct	0.236275825	CT
NA12872	75	CC	0.995	cc	0.25912795	CC
NA12865	76	TT	1	tt	0.485116006	TT
NA10860	77		NA	CT	0.345704003	
NA12873	78	TT	1	tt	0.305997632	TT
NA12752	79	CC	1	cc	0.201435127	CC
NA10851	80	TT	1	tt	0.430965826	TT
NA07029	81	CT	0.9993	ct	0.426101264	CT
NA12248	82	CT	0.9939	ct	0.169474931	CT
NA12155	83	TT	1	tt	0.157932592	TT
NA10838	84	TT	1	tt	0.629183097	TT
NA12812	85	TT	1	tt	0.542630692	TT
NA12248.dup	86	CT	0.9278	ct	0.316305877	nn
NA12043	87	CT	1	ct	0.2561771	CT
NA12234	88	TT	1	tt	0.344528128	TT

	MACGT discrepancy					
	LDA discrepancy					
NA	LDA output were not g					
XY	Manual calls are given					
		**12472674**				
**Coriell**	**Sample**	**LDA Call**	**LDA quality score**	**MACGT Call**	**MACGT quality score**	**HapMap Call**
NA12892	89	TT	1	tt	0.559659147	TT
NA10846	90		NA	CC	0.300353676	
NA06993.dup	91	CT	0.9582	ct	0.279365595	nn
NA12249	92	CC	1	cc	0.086109763	CC
NA11881	93	TT	1	tt	0.165929933	TT
NA12815	94	TT	1	tt	0.126713601	TT
NA12264	95		NA	CC	0.494994904	
NA18526	96	TT	1	tt	0.252690781	TT
NA18562	97	TT	0.9999	tt	0.380631066	TT
NA18545	98	CT	1	ct	0.109964447	CT
NA18609	99	CT	1	ct	0.4711073	CT
NA18566	100	CC	0.9992	***CC***	***0***	CC
NA18621	101	CT	1	ct	0.347033793	CT
NA18577	102	TT	0.9472	tt	0.070097181	TT
NA18635	103	CT	1	ct	0.345503709	CT
NA18524	104	CT	1	ct	0.607105673	CT
NA18537	105	CC	0.9938	cc	0.179322422	CC
NA18572	106	TT	0.9988	tt	0.147148567	TT
NA18552	107	CT	0.9999	ct	0.035462273	CT
NA18563	108	CT	1	ct	0.285442458	CT
NA18594	109	TT	0.994	tt	0.162204623	nn
NA18624	110	CT	1	ct	0.235466417	CT
NA18592	111	CT	1	ct	0.521766379	CT
NA18529	112	TT	1	tt	0.241515525	TT
NA18603	113	CC	0.7613	cc	0.05189901	nn
NA18547	114	TT	0.9977	tt	0.05372039	TT
NA18611	115	CT	1	ct	0.051353437	CT
NA18570	116	TT	1	tt	0.173372846	TT
NA18622	117	CC	0.9443	cc	0.081691064	CC
NA18579	118	CT	0.9992	ct	0.19992609	CT
NA18636	119	CC	1	cc	0.001999848	CC
NA18558	120	TT	1	tt	0.484502414	TT
NA18540	121	TT	1	tt	0.468805879	TT
NA18609	122	CT	0.9996	ct	0.14315099	nn
NA18555	123	CT	0.9866	ct	0.089736027	CT
NA18612	124	CC	0.9999	cc	0.268380863	CC
NA18573	125	TT	1	tt	0.075560939	TT
NA18632	126	CT	0.9798	ct	0.481355085	CT
NA18593	127	CC	0.9992	cc	0.095644755	CC
NA18532	128	CT	0.9999	ct	0.463935008	CT
NA18605	129	CT	0.9984	ct	0.412992219	CT
NA18550	130	TT	1	tt	0.356282912	TT
NA18603	131	CC	0.9999	cc	0.180655689	CC
NA18571	132	CC	1	cc	0.295884791	CC

	MACGT discrepancy					
	LDA discrepancy					
NA	LDA output were not g					
XY	Manual calls are given					
		**12472674**				
**Coriell**	**Sample**	**LDA Call**	**LDA quality score**	**MACGT Call**	**MACGT quality score**	**HapMap Call**
NA18623	133	CC	1	cc	0.172546526	CC
NA18582	134	CT	0.9722	ct	0.014325863	CT
NA18637	135	CT	0.9936	ct	0.172664612	CT
NA18561	136	CC	1	cc	0.306259364	CC
NA18542	137	TT	1	tt	0.646891032	TT
NA18608	138	CC	1	cc	0.288037856	CC
NA18564	139	CT	0.9999	ct	0.303287812	CT
NA18620	140	CT	0.9939	ct	0.468414794	CT
NA18576	141	CT	0.9999	ct	0.515009484	CT
NA18633	142	CT	1	ct	0.058017281	CT
NA18594	143	TT	1	tt	0.456115056	TT
NA18942	144	CT	0.9996	ct	0.614107777	CT
NA18945	145	TT	1	tt	0.444385936	TT
NA18964	146	CT	1	ct	0.612448723	CT
NA18961	147	CT	0.9999	ct	0.579766277	CT
NA18967	148	CC	0.9999	cc	0.267303737	CC
NA18981	149	CT	1	ct	0.451318278	CT
NA18994	150	CT	1	ct	0.526899509	CT
NA18998	151	TT	1	tt	0.157631474	TT
NA18940	152	CT	0.9971	ct	0.079374797	CT
NA18949	153	CT	0.9991	ct	0.01566701	CT
NA18953	154	TT	1	tt	0.120001969	TT
NA18972	155	TT	1	tt	0.129312014	TT
NA18976	156	TT	1	tt	0.326028979	TT
NA18971	157	TT	1	tt	0.363848286	TT
blank	158	NN	0.75	***NN***	***0***	nn
NA19000	159	TT	1	tt	0.477402761	TT
NA18951	160	TT	1	tt	0.446209555	nn
NA18948	161	TT	1	tt	0.384555525	TT
NA18968	162	CC	1	cc	0.647375714	CC
NA18965	163	CT	0.97	ct	0.171017624	CT
NA18978	164	TT	1	tt	0.252811978	TT
NA18974	165	TT	1	tt	0.251613004	TT
NA18992	166	TT	1	tt	0.238021402	TT
NA19005	167	CT	1	ct	0.274558276	CT
NA18943	168	CT	1	ct	0.337418826	CT
NA18951	169	TT	1	tt	0.448006559	TT
NA18959	170	TT	1	tt	0.535808734	TT
NA18973	171	TT	1	tt	0.390103798	TT
NA18970	172	CT	1	ct	0.324348426	CT
NA18987	173	CT	1	ct	0.205099399	CT
NA18995	174	CC	0.9996	cc	0.121857739	CC
NA18999	175	TT	1	tt	0.531016278	TT
NA18947	176	TT	0.7638	tt	0.027618403	TT

	MACGT discrepancy					
	LDA discrepancy					
NA	LDA output were not g					
XY	Manual calls are given					
		**12472674**				
**Coriell**	**Sample**	**LDA Call**	**LDA quality score**	**MACGT Call**	**MACGT quality score**	**HapMap Call**
NA18952	177	TT	1	tt	0.150757974	TT
NA18969	178	TT	1	tt	0.011750986	TT
NA18966	179	NN	0.75	tt	0.605034141	TT
NA18980	180	NN	0.7499	ct	0.178208591	CT
NA18990	181	TT	0.75	*TT*	*0*	TT
NA18997	182	TT	1	tt	0.499454243	TT
NA19007	183	CT	1	ct	0.005750198	CT
NA18944	184	TT	1	tt	0.01646086	TT
NA18956	185	TT	1	tt	0.531240707	TT
NA18960	186	CT	1	ct	0.30226884	CT
NA18975	187	TT	1	tt	0.245293786	TT
NA18995	188	CC	0.994	cc	0.085155175	nn
NA18991	189	CC	0.9848	cc	0.080658319	CC
NA18996	190	CT	1	ct	0.492374448	nn
NA19003	191	TT	1	tt	0.697495912	TT
NA18502	192	CC	0.9802	cc	0.05146918	CC
NA19153	193	CT	1	ct	0.158762762	CT
NA18857	194	CC	1	cc	0.28842938	CC
NA19223	195	CC	0.9339	cc	0.084458039	CC
NA19201	196	CC	0.9715	cc	0.042400082	CC
NA18504	197	CT	1	ct	0.470160132	CT
NA18870	198	CT	1	ct	0.203079079	CT
NA18863	199	CC	0.9976	cc	0.001777952	CC
NA19145	200	CC	1	cc	0.045281166	CC
NA19137	201	CT	0.9993	ct	0.11836728	CT
NA19238	202	TT	1	tt	0.03305579	nn
NA18500	203	CC	0.9929	*CC*	*0*	CC
NA19144	204	CC	0.9997	*CC*	*0*	CC
NA19203	205	CT	0.9998	ct	0.032830738	CT
NA19200	206	CT	1	ct	0.240072791	CT
NA18855	207	CT	0.9679	ct	0.247633668	CT
NA18505	208	CC	0.9995	cc	0.076036857	CC
NA19202	209	CC	1	cc	0.206963705	CC
NA18501	210	CC	1	cc	0.007067374	CC
NA18861	211	CT	0.9522	*CT*	*0*	CT
NA19193	212	CT	0.9995	ct	0.063722463	CT
NA19143	213	CT	0.9998	ct	0.180775614	CT
NA18517	214	CT	0.9961	ct	0.027971251	CT
NA18862	215	CC	1	cc	0.17814781	CC
NA18856	216	CT	0.9306	ct	0.026035151	nn
NA19239	217	CT	0.957	ct	0.024545198	CT
NA19240	218	TT	1	tt	0.434189222	TT
NA18856	219	CT	0.9634	ct	0.118430313	CT
NA18503	220	CC	0.9843	cc	0.005491585	CC

	MACGT discrepancy					
	LDA discrepancy					
NA	LDA output were not g					
XY	Manual calls are given					
		12472674				
**Coriell**	**Sample**	**LDA Call**	**LDA quality score**	**MACGT Call**	**MACGT quality score**	**HapMap Call**
NA18871	221	TT	1	tt	0.508373555	TT
NA19221	222	CC	0.9394	cc	0.03400449	CC
NA19209	223	CT	1	ct	0.401950791	CT
NA19152	224	CC	0.9432	cc	0.033551406	CC
NA18515	225	CT	1	ct	0.46399203	CT
NA19238	226	TT	1	tt	0.490074223	TT
NA19154	227	CC	0.9749	cc	0.091382155	CC
NA19210	228	CT	1	ct	0.192246127	CT
NA19211	229	CT	0.9963	ct	0.400696158	CT
NA18862	230	CC	0.9976	cc	0.020198951	nn
NA18872	231	CT	0.9995	ct	0.217211655	CT
NA19139	232	CC	0.9996	cc	0.033610519	CC
NA19222	233	CC	0.9994	cc	0.078263192	CC
NA19194	234	CC	0.998	cc	0.212459361	CC
NA19138	235	CC	1	cc	0.234833008	CC
NA19204	236	CT	1	ct	0.103452418	CT
NA18516	237	CC	0.7605	***CC***	***0***	CC
NA19205	238	TT	1	tt	0.530502724	TT
NA19192	239	CC	0.7564	***CC***	***0***	CC
NA18912	240	TT	1	tt	0.412088799	nn
NA18508	241	CC	0.9932	cc	0.019890877	CC
NA19142	242	CT	1	ct	0.168814116	CT
NA18852	243	CT	0.9975	ct	0.171475953	CT
NA18507	244	CC	0.9999	cc	0.111885712	CC
blank	245	NN	0.6902	***NN***	***0***	nn
NA19101	246	TT	1	tt	0.288251612	TT
NA19172	247	NN	0.7174	cc	0.069864984	CC
NA19160	248	CT	0.9999	ct	0.113203399	CT
NA19129	249	TT	0.9995	tt	0.113078964	TT
NA18913	250	CT	0.9999	ct	0.051803992	CT
NA19120	251	CC	1	cc	0.204608409	CC
NA19159	252		NA	CC	0.151581115	
NA18523	253	TT	1	tt	0.469208889	TT
NA19102	254	CC	0.9999	cc	0.212368293	CC
NA19092	255	TT	1	tt	0.440229661	TT
NA18521	256	CT	1	ct	0.220913022	CT
NA18912	257	TT	1	tt	0.149847743	TT
NA19206	258	CC	0.9428	cc	0.029290467	CC
NA19128	259	CT	1	ct	0.38423712	CT
NA18859	260	TT	0.8524	tt	0.452640378	nn
NA19094	261	NN	0.75	ct	0.445229387	CT
NA19171	262	NN	0.75	ct	0.424071629	CT
NA18860	263	CT	1	ct	0.248205661	CT
NA18858	264	CC	1	cc	0.159481766	CC

	MACGT discrepancy					
	LDA discrepancy					
NA	LDA output were not g					
XY	Manual calls are given					
		**12472674**				
**Coriell**	**Sample**	**LDA Call**	**LDA quality score**	**MACGT Call**	**MACGT quality score**	**HapMap Call**
NA19099	265	CT	1	ct	0.392281857	CT
NA18506	266	CC	0.8747	*CC*	*0*	CC
NA19131	267	CC	0.8255	cc	0.003599879	CC
NA19116	268	CC	0.9817	cc	0.098512644	CC
NA19161	269	CC	0.9998	cc	0.107008539	CC
NA19140	270	CC	1	cc	0.116012646	CC
NA19207	271	CC	1	cc	0.206696348	CC
NA19141	272	TT	1	tt	0.130203455	TT
NA18854	273	CT	1	ct	0.147389253	CT
NA19098	274	CC	0.9924	cc	0.019175693	CC
NA18522	275	CC	0.9501	cc	0.009373671	CC
NA18914	276	TT	1	tt	0.199112869	TT
NA18853	277	CC	1	cc	0.048560333	CC
NA18859	278	TT	0.7874	tt	0.293921077	TT
NA19100	279	NN	0.6054	*CC*	*5.92E-04*	CC
NA19093	280	CC	0.7595	cc	0.006745307	CC
NA19132	281	CC	0.9975	cc	0.154898383	CC
NA19130	282	CC	0.8783	cc	0.019496759	CC
NA19208	283	CC	0.7506	*CC*	*0*	CC
NA19127	284	CT	0.9989	ct	0.019182404	CT
NA19173	285	CC	1	cc	0.281372148	CC
NA19119	286	CC	0.9187	cc	0.013706972	CC
NA19103	287	CT	1	ct	0.071510763	CT
	Discrepancies	0		0		
	NNs	7		1		
	Automated Calls	274		257		
	Manual Calls	0		18		
	Concordant Calls	249		255		255

	MACGT discrepancy					
	LDA discrepancy					
NA	LDA output were not g					
XY	Manual calls are given					
		12583473				
**Coriell**	**Sample**	**LDA Call**	**LDA quality score**	**MACGT Call**	**MACGT quality score**	**HapMap Call**
NA12753	1		NA	GG	0.358940114	
NA12875	2	GG	0.9923	gg	0.132127366	GG
NA12044	3	GG	0.9996	gg	0.395155838	GG
NA06993	4	GG	0.9913	gg	0.122291578	GG
NA12716	5	GG	0.9997	gg	0.209487758	GG
NA12760	6	GG	0.9916	gg	0.030880404	GG
NA07348	7	GG	0.9946	gg	0.416097825	GG
NA12707	8	GG	0.9845	gg	0.186583538	GG
NA12156	9		NA	GG	0.03523753	
NA11992	10	GG	0.9892	gg	0.343115624	GG
NA12239	11	GG	0.9979	gg	0.433134422	GG
NA12878	12	GG	0.999	gg	0.497596617	GG
NA11993	13	GG	0.9881	gg	0.039025039	GG
NA12750	14	GG	0.9895	gg	0.126459686	GG
NA12146	15	GG	0.9959	gg	0.22979825	GG
NA11839	16	GG	0.9718	gg	0.073912905	GG
NA12156.dup	17	GG	0.9932	gg	0.310283196	nn
NA11829	18		NA	GG	0.233893743	
NA12154	19	GG	0.9913	gg	0.075103952	GG
NA10856	20	GG	0.9671	gg	0.050004258	GG
NA06985	21	GG	0.9946	gg	0.352553004	GG
NA11831	22	GG	0.9999	gg	0.109448339	GG
NA12003.dup	23	GG	0.9993	gg	0.196844738	nn
NA10859	24	GG	0.9997	gg	0.249373802	GG
NA07056	25	GG	0.9997	gg	0.2431776	GG
NA07022	26	GG	1	gg	0.344522187	GG
NA12762	27		NA	GG	0.512895475	
NA12874	28	GG	0.9984	gg	0.146959488	GG
NA12003	29	GG	0.9996	gg	0.280496483	GG
NA10835	30	GG	0.9989	gg	0.17863732	GG
NA11882	31	GG	0.9995	gg	0.34815476	GG
NA07034	32	GG	0.9958	gg	0.060307091	GG
NA12056	33	GG	0.9999	gg	0.356553692	GG
NA12145	34	GG	1	gg	0.150870799	GG
NA07019	35	GG	0.999	gg	0.39902993	GG
NA06991	36		NA	GG	0.511402298	
NA12761	37	GG	0.9999	gg	0.176759891	GG
NA06994	38	GG	0.9999	gg	0.229819862	GG
NA12864	39	GG	0.9996	gg	0.434503421	GG
NA07055	40	GG	0.9965	gg	0.078370571	GG
NA10863	41	GG	0.999	gg	0.20351105	GG
NA12763	42	GG	0.9994	gg	0.08194211	GG
NA10831	43	GG	0.9999	gg	0.296031236	GG
NA11840	44	GG	1	gg	0.372451761	GG

	MACGT discrepancy					
	LDA discrepancy					
NA	LDA output were not g					
XY	Manual calls are given					
		12583473				
**Coriell**	**Sample**	**LDA Call**	**LDA quality score**	**MACGT Call**	**MACGT quality score**	**HapMap Call**
NA10830	45	GG	1	gg	0.290802548	GG
NA11993.dup	46	GG	1	gg	0.257313773	nn
NA12751	47	GG	1	gg	0.236767768	GG
NA12814	48	GG	0.9783	gg	0.062068999	GG
NA10857	49	GG	0.9994	gg	0.265893723	GG
NA07357	50	GG	1	gg	0.539393853	GG
NA07000	51	GG	1	gg	0.257714873	GG
NA12802	52	GG	1	gg	0.07078063	GG
NA10855	53	GG	0.9999	gg	0.179804504	GG
NA11995	54		NA	GG	0.215730355	
NA10839	55	GG	0.9918	gg	0.214324096	GG
NA10847	56	GG	0.997	gg	0.374300555	GG
NA12006	57	GG	0.9227	gg	0.0747115	GG
NA12144	58	GG	0.9988	gg	0.42744076	GG
NA11832	59	GG	0.9999	gg	0.182360411	GG
NA12813	60	GG	0.9961	gg	0.303618057	GG
blank	61	GG	1	gg	0.509309542	nn
NA12891	62	GG	0.9992	gg	0.296435384	GG
NA12717	63		NA	GG	0.41950828	
NA10861	64	GG	1	gg	0.230538795	GG
NA12004	65	GG	0.9954	gg	0.027065484	GG
NA07048	66	GG	0.998	gg	0.480405723	GG
NA12801	67	GG	0.9899	gg	0.026724252	GG
NA11830	68	GG	0.9958	gg	0.017266663	GG
NA12057	69	GG	0.9996	***GG***	***0***	GG
NA11994	70		NA	GG	0.40804701	
NA12740	71	GG	0.999	gg	0.177840039	GG
NA12005	72	GG	0.991	gg	0.060429827	GG
NA07345	73	GG	0.9915	gg	0.082121584	GG
NA10854	74	GG	0.9896	gg	0.335510814	GG
NA12872	75	GG	0.9482	gg	0.028592015	GG
NA12865	76	GG	0.995	gg	0.373220787	GG
NA10860	77		NA	GG	0.31566619	
NA12873	78	GG	0.9987	gg	0.333016089	GG
NA12752	79	GG	0.9959	gg	0.343056928	GG
NA10851	80	GG	0.9961	gg	0.416039912	GG
NA07029	81	GG	0.9975	gg	0.390949514	GG
NA12248	82	GG	0.9997	gg	0.2519401	GG
NA12155	83	GG	0.9923	gg	0.049775817	GG
NA10838	84	GG	0.9913	gg	0.124721714	GG
NA12812	85	GG	0.9885	gg	0.229885154	GG
NA12248.dup	86	GG	0.994	gg	0.230526481	nn
NA12043	87	GG	0.9925	gg	0.328874902	GG
NA12234	88	GG	0.9997	gg	0.004035158	GG

	MACGT discrepancy					
	LDA discrepancy					
NA	LDA output were not g					
XY	Manual calls are given					
		12583473				
**Coriell**	**Sample**	**LDA Call**	**LDA quality score**	**MACGT Call**	**MACGT quality score**	**HapMap Call**
NA12892	89	GG	0.9997	gg	0.386182288	GG
NA10846	90	GG	0.9996	gg	0.278835474	GG
NA06993.dup	91	GG	0.9985	gg	0.115567404	nn
NA12249	92	GG	0.9933	gg	0.152793953	GG
NA11881	93	GG	0.9946	gg	0.147327053	GG
NA12815	94	GG	0.9835	gg	0.014942716	GG
NA12264	95	GG	0.9998	gg	0.338775517	GG
NA18526	96	CG	0.9979	cg	0.466896418	CG
NA18562	97	GG	0.9993	gg	0.33826945	GG
NA18545	98	GG	1	gg	0.325199524	GG
NA18609	99	GG	1	gg	0.300343826	GG
NA18566	100	GG	1	gg	0.191931552	GG
NA18621	101	CG	0.9938	cg	0.497416847	CG
NA18577	102	CG	0.9971	cg	0.489791211	CG
NA18635	103	GG	1	gg	0.287498551	GG
NA18524	104	GG	0.9998	gg	0.061731484	GG
NA18537	105	CG	0.9988	cg	0.471328696	CG
NA18572	106		NA	CC	0.343797055	
NA18552	107	GG	0.9989	gg	0.154541931	GG
NA18563	108	GG	0.9994	gg	0.305466277	GG
NA18594	109	CG	0.9958	cg	0.433181313	nn
NA18624	110	CG	0.9978	cg	0.577012188	CG
NA18592	111	GG	0.9964	***GG***	***0***	GG
NA18529	112	GG	0.9981	gg	0.538409077	GG
NA18603	113	GG	0.999	gg	0.560307115	nn
NA18547	114	GG	0.9999	gg	0.650419094	GG
NA18611	115	GG	0.906	gg	0.00464919	GG
NA18570	116	GG	0.9848	gg	0.103364926	GG
NA18622	117	NN	0.6025	cg	0.004981941	CG
NA18579	118	GG	0.9926	gg	0.234245861	GG
NA18636	119	NN	0.5701	cg	0.003206492	CG
NA18558	120	GG	0.9981	gg	0.431654744	GG
NA18540	121		NA	CC	0.462326554	
NA18609	122	GG	0.993	gg	0.05159157	nn
NA18555	123	CG	0.9984	cg	0.286047744	CG
NA18612	124	GG	0.9978	gg	0.60638585	GG
NA18573	125	GG	0.9919	gg	0.06466231	GG
NA18632	126	GG	0.9949	cg	0.001298356	GG
NA18593	127	GG	0.9972	gg	0.150739943	GG
NA18532	128	GG	0.9967	gg	0.339071361	GG
NA18605	129	GG	0.9998	gg	0.326989249	GG
NA18550	130	GG	0.9999	gg	0.330708886	GG
NA18603	131	GG	0.9992	gg	0.079390066	GG
NA18571	132	GG	0.9981	gg	0.255173623	GG

	MACGT discrepancy					
	LDA discrepancy					
NA	LDA output were not g					
XY	Manual calls are given					
		12583473				
**Coriell**	**Sample**	**LDA Call**	**LDA quality score**	**MACGT Call**	**MACGT quality score**	**HapMap Call**
NA18623	133	CG	0.9975	cg	0.541879395	CG
NA18582	134	GG	0.9973	gg	0.07996431	GG
NA18637	135	CG	0.9981	cg	0.499392523	CG
NA18561	136	CG	0.9945	cg	0.429400184	CG
NA18542	137	GG	0.9979	gg	0.321771736	GG
NA18608	138	GG	0.9994	gg	0.269815917	GG
NA18564	139	GG	0.9999	gg	0.346849147	GG
NA18620	140	GG	1	gg	0.562202119	GG
NA18576	141	CG	0.9947	cg	0.376053842	CG
NA18633	142	CG	0.9974	cg	0.422185595	CG
NA18594	143	CG	0.9953	cg	0.377478651	CG
NA18942	144	CG	0.9979	cg	0.310298455	CG
NA18945	145	GG	0.9911	gg	0.017779781	GG
NA18964	146	CG	0.9979	cg	0.365128859	CG
NA18961	147	CG	0.9969	cg	0.430227433	CG
NA18967	148		NA	CC	0.45433423	
NA18981	149	GG	0.9997	gg	0.406694293	GG
NA18994	150	GG	1	gg	0.241858807	GG
NA18998	151	GG	1	gg	0.095538106	GG
NA18940	152	CG	0.9957	cg	0.336518142	CG
NA18949	153	CG	0.9914	cg	0.214898013	CG
NA18953	154	GG	0.9999	gg	0.014060255	GG
NA18972	155	GG	0.9985	gg	0.306734303	GG
NA18976	156	GG	0.9704	gg	0.304623249	GG
NA18971	157	GG	0.9996	gg	0.094051974	GG
blank	158	NN	0.5003	*NN*	*0*	nn
NA19000	159	GG	1	gg	0.303323931	GG
NA18951	160	CG	0.9948	cg	0.367504303	nn
NA18948	161	GG	0.9899	gg	0.374630403	GG
NA18968	162	CG	0.9969	cg	0.290220211	CG
NA18965	163	CG	0.9866	cg	0.013271493	CG
NA18978	164	GG	0.9688	gg	0.009327137	GG
NA18974	165	GG	0.966	gg	0.007289178	GG
NA18992	166	GG	0.995	*GG*	*8.70E-04*	GG
NA19005	167	GG	0.9992	gg	0.263465659	GG
NA18943	168	GG	1	gg	0.364021467	GG
NA18951	169	CG	0.9871	cg	0.430358272	CG
NA18959	170		NA	CC	0.389312467	
NA18973	171	CG	0.9968	cg	0.495873195	CG
NA18970	172	GG	0.9999	gg	0.386097484	GG
NA18987	173	GG	1	gg	0.561222753	GG
NA18995	174	GG	0.9997	gg	0.499444969	GG
NA18999	175	CG	0.9945	cg	0.547812413	CG
NA18947	176	GG	0.9993	gg	0.02094355	GG

	MACGT discrepancy					
	LDA discrepancy					
NA	LDA output were not g					
XY	Manual calls are given					
		**12583473**				
**Coriell**	**Sample**	**LDA Call**	**LDA quality score**	**MACGT Call**	**MACGT quality score**	**HapMap Call**
NA18952	177	GG	1	gg	0.03705363	GG
NA18969	178	CG	0.9946	cg	0.440808668	CG
NA18966	179	GG	0.9994	gg	0.51481342	GG
NA18980	180	GG	1	gg	0.437354616	GG
NA18990	181	GG	0.9962	gg	0.441613723	GG
NA18997	182	CG	0.9967	cg	0.408181468	CG
NA19007	183	GG	0.9983	gg	0.342287026	GG
NA18944	184	GG	0.9953	gg	0.049760609	GG
NA18956	185	GG	1	gg	0.434926953	GG
NA18960	186	GG	0.9982	gg	0.456309196	GG
NA18975	187	CG	0.9963	cg	0.45531541	CG
NA18995	188	GG	0.9993	gg	0.469366293	nn
NA18991	189	CG	0.9953	cg	0.496498806	CG
NA18996	190	GG	0.9881	gg	0.449969836	nn
NA19003	191	CG	0.9969	cg	0.593179296	CG
NA18502	192	GG	0.995	gg	0.004719143	GG
NA19153	193	GG	1	gg	0.341770039	GG
NA18857	194	CG	0.9956	cg	0.614514142	CG
NA19223	195	GG	1	gg	0.155832737	GG
NA19201	196	GG	0.9973	gg	0.222184671	GG
NA18504	197	CG	0.9948	cg	0.556282261	CG
NA18870	198	GG	0.999	gg	0.36961313	GG
NA18863	199	GG	0.9999	***GG***	***4.91E-04***	GG
NA19145	200	CG	0.9941	cg	0.533974178	CG
NA19137	201	GG	0.9955	gg	0.194387095	GG
NA19238	202	GG	0.9974	gg	0.237170432	nn
NA18500	203		NA	CG	0.531469225	
NA19144	204		NA	CG	0.313857363	
NA19203	205	GG	0.9954	gg	0.354797861	GG
NA19200	206		NA	CG	0.51552068	
NA18855	207	GG	0.9748	gg	0.043173221	GG
NA18505	208	CG	0.9952	cg	0.002213121	CG
NA19202	209	CG	0.9931	cg	0.487300107	CG
NA18501	210	CG	0.9984	cg	0.486801775	CG
NA18861	211	GG	1	gg	0.008027935	GG
NA19193	212	GG	0.999	gg	0.307258478	GG
NA19143	213	CG	0.9971	***CG***	***0***	CG
NA18517	214	GG	0.9987	gg	0.379402346	GG
NA18862	215	GG	0.9997	gg	0.240840428	GG
NA18856	216	CG	0.9965	cg	0.035712329	nn
NA19239	217	GG	1	gg	0.105844956	GG
NA19240	218	GG	0.9998	gg	0.413007494	GG
NA18856	219	CG	0.9939	***CG***	***0***	CG
NA18503	220	CC	1	cc	0.270716196	CC

	MACGT discrepancy					
	LDA discrepancy					
NA	LDA output were not g					
XY	Manual calls are given					
		**12583473**				
**Coriell**	**Sample**	**LDA Call**	**LDA quality score**	**MACGT Call**	**MACGT quality score**	**HapMap Call**
NA18871	221	GG	1	gg	0.398295891	GG
NA19221	222	CG	0.9926	cg	0.606231792	CG
NA19209	223	GG	0.9977	gg	0.044721337	GG
NA19152	224	CG	0.9919	cg	0.405003783	CG
NA18515	225	GG	0.9952	gg	0.104405056	GG
NA19238	226	GG	0.995	gg	0.382401595	GG
NA19154	227	CG	0.9928	cg	0.565707323	CG
NA19210	228	CG	0.9924	cg	0.19932396	CG
NA19211	229	GG	0.9925	gg	0.002774218	GG
NA18862	230	GG	0.9999	gg	0.362315629	nn
NA18872	231	GG	1	gg	0.151589742	GG
NA19139	232	GG	0.9963	gg	0.189154259	GG
NA19222	233	CG	0.9972	cg	0.048230999	CG
NA19194	234	GG	0.9976	gg	0.01415101	GG
NA19138	235	GG	0.9352	***GG***	***0***	GG
NA19204	236	GG	1	gg	0.286651184	GG
NA18516	237	GG	1	gg	0.030102135	GG
NA19205	238	GG	0.9968	***GG***	***0***	GG
NA19192	239	GG	0.9976	gg	0.047876019	GG
NA18912	240	GG	0.9947	gg	0.263266996	nn
NA18508	241	GG	1	gg	0.200397994	GG
NA19142	242		NA	CC	0.261159239	
NA18852	243	GG	1	gg	0.363326518	GG
NA18507	244	GG	0.995	gg	0.003995136	GG
blank	245	NN	0.4994	***NN***	***0***	nn
NA19101	246		NA	CG	0.50762703	
NA19172	247	GG	0.997	gg	0.00139172	GG
NA19160	248	GG	0.9969	gg	0.008162673	GG
NA19129	249	CG	0.991	cg	0.448333513	CG
NA18913	250	GG	0.9997	gg	0.108715996	GG
NA19120	251	CG	0.983	***CG***	***0***	CG
NA19159	252	GG	0.9986	gg	0.03027854	GG
NA18523	253	GG	0.9906	gg	0.376405873	GG
NA19102	254	GG	0.9874	***GG***	***0***	GG
NA19092	255	GG	1	gg	0.305117234	GG
NA18521	256	CG	0.9943	cg	0.026629324	CG
NA18912	257	GG	0.9962	gg	0.00181103	GG
NA19206	258	GG	1	***GG***	***0***	GG
NA19128	259	GG	0.9984	gg	0.306883436	GG
NA18859	260	GG	0.9971	gg	0.343448158	nn
NA19094	261	GG	1	gg	0.495716226	GG
NA19171	262	CG	0.9955	cg	0.247488424	CG
NA18860	263	GG	1	gg	0.164343044	GG
NA18858	264	GG	0.9995	gg	0.11378643	GG

	MACGT discrepancy					
	LDA discrepancy					
NA	LDA output were not g					
XY	Manual calls are given					
		12583473				
**Coriell**	**Sample**	**LDA Call**	**LDA quality score**	**MACGT Call**	**MACGT quality score**	**HapMap Call**
NA19099	265	GG	0.9999	gg	0.150710538	GG
NA18506	266	GG	1	gg	0.018066079	GG
NA19131	267	GG	1	***GG***	***0***	GG
NA19116	268	CC	1	***CC***	***0***	CC
NA19161	269	GG	0.9725	***GG***	***0***	GG
NA19140	270	CG	0.9536	cg	0.117213698	CG
NA19207	271	GG	0.9995	gg	0.351832434	GG
NA19141	272	CG	0.972	cg	0.059743883	CG
NA18854	273	GG	1	gg	0.021958048	GG
NA19098	274	GG	1	gg	0.402601226	GG
NA18522	275	CG	0.994	cg	0.018488937	CG
NA18914	276	GG	0.9668	gg	0.00289909	GG
NA18853	277	GG	0.9955	gg	0.071539684	GG
NA18859	278	GG	0.9564	gg	0.024106089	GG
NA19100	279	GG	0.9999	***GG***	***0***	GG
NA19093	280	GG	0.9974	gg	0.034310534	GG
NA19132	281	GG	1	gg	0.017078935	GG
NA19130	282	CG	0.9818	***NN***	***8.71E-04***	CG
NA19208	283	GG	0.9733	gg	0.003173458	GG
NA19127	284	CG	0.9967	cg	0.229035574	CG
NA19173	285	GG	0.9991	gg	0.016909793	GG
NA19119	286	GG	0.9904	gg	0.014184216	GG
NA19103	287	GG	0.9951	gg	0.011095369	GG
	Discrepancies	0		1		
	NNs	2		1		
	Automated Calls	269		251		
	Manual Calls	0		18		
	Concordant Calls	248		248		250

	MACGT discrepancy					
	LDA discrepancy					
NA	LDA output were not g					
XY	Manual calls are given					
		1258464				
**Coriell**	**Sample**	**LDA Call**	**LDA quality score**	**MACGT Call**	**MACGT quality score**	**HapMap Call**
NA12753	1		NA	TC	0.514182977	
NA12875	2	CC	1	cc	0.589294442	CC
NA12044	3	CC	0.9997	cc	0.081601315	CC
NA06993	4	TC	0.9999	tc	0.452458108	TC
NA12716	5	CC	1	cc	0.256568914	CC
NA12760	6	TC	0.9999	tc	0.320362235	TC
NA07348	7	TT	0.9999	tt	0.256846499	TT
NA12707	8	TC	0.9999	tc	0.08952991	TC
NA12156	9		NA	CC	0.322242219	
NA11992	10	TC	1	tc	0.290477682	TC
NA12239	11	CC	0.7774	cc	0.298324973	CC
NA12878	12	CC	0.9764	cc	0.001486907	CC
NA11993	13	TT	0.9619	tt	0.007055798	TT
NA12750	14	TC	1	tc	0.137020468	TC
NA12146	15	TT	0.9922	tt	0.062227364	TT
NA11839	16	TC	1	tc	0.002917061	TC
NA12156.dup	17	CC	1	cc	0.078328098	nn
NA11829	18		NA	TC	0.210774813	
NA12154	19	CC	1	cc	0.10696344	CC
NA10856	20	TT	0.9959	tt	0.201047536	TT
NA06985	21	CC	1	cc	0.052399171	CC
NA11831	22	TC	1	tc	0.031116708	TC
NA12003.dup	23	TT	0.9697	tt	0.060852115	nn
NA10859	24	CC	1	cc	0.146602922	CC
NA07056	25	TT	0.9986	tt	0.047667586	TT
NA07022	26	TC	1	tc	0.122099985	TC
NA12762	27		NA	TC	0.261947356	
NA12874	28	TC	1	tc	0.229847596	TC
NA12003	29	TT	1	tt	0.012980574	TT
NA10835	30	TT	0.9999	tt	0.136718792	TT
NA11882	31	TC	1	tc	0.106479272	TC
NA07034	32	TC	1	tc	0.206190151	TC
NA12056	33	TC	0.9999	tc	0.29619139	TC
NA12145	34	CC	1	cc	0.280778382	CC
NA07019	35	TC	1	tc	0.427388583	TC
NA06991	36		NA	CC	0.533510477	
NA12761	37	TC	1	tc	0.297577444	TC
NA06994	38	TC	1	tc	0.1860385	TC
NA12864	39	TT	1	tt	0.194809617	TT
NA07055	40	TT	1	tt	0.321317303	TT
NA10863	41	TC	1	tc	0.368083668	TC
NA12763	42	TC	1	tc	0.344422003	TC
NA10831	43	CC	1	cc	0.157570371	CC
NA11840	44	TC	1	tc	0.459291881	TC

	MACGT discrepancy					
	LDA discrepancy					
NA	LDA output were not g					
XY	Manual calls are given					
		**1258464**				
**Coriell**	**Sample**	**LDA Call**	**LDA quality score**	**MACGT Call**	**MACGT quality score**	**HapMap Call**
NA10830	45	CC	1	cc	0.53331096	CC
NA11993.dup	46	TT	0.9987	tt	0.197619675	nn
NA12751	47	TC	1	tc	0.393146064	TC
NA12814	48	TT	0.9991	tt	0.225804053	TT
NA10857	49	CC	1	cc	0.347071772	CC
NA07357	50	TC	1	tc	0.442712409	TC
NA07000	51	TT	1	tt	0.080538663	TT
NA12802	52	TT	1	tt	0.269443169	TT
NA10855	53	TT	1	tt	0.026531222	TT
NA11995	54		NA	CC	0.123692464	
NA10839	55	TT	1	tt	0.061709626	TT
NA10847	56	TC	1	tc	0.173289979	TC
NA12006	57	TT	1	tt	0.002410716	TT
NA12144	58	TC	1	tc	0.457679621	TC
NA11832	59	TC	1	tc	0.175245053	TC
NA12813	60	TC	1	tc	0.261417241	TC
blank	61	NN	0.75	***NN***	***0***	nn
NA12891	62	TC	1	tc	0.173061095	TC
NA12717	63		NA	TT	0.404314702	
NA10861	64	TC	1	tc	0.147394608	TC
NA12004	65	TT	1	tt	0.004285017	TT
NA07048	66	TT	1	tt	0.001413037	TT
NA12801	67	TT	1	tt	0.005609629	TT
NA11830	68	TT	1	tt	0.033428981	TT
NA12057	69	TC	0.9995	tc	0.209624296	TC
NA11994	70		NA	TC	0.553459845	
NA12740	71	TC	1	tc	0.494859225	TC
NA12005	72	TT	0.9394	tt	0.007776796	TT
NA07345	73	TC	1	tc	0.052777488	TC
NA10854	74	TC	1	tc	0.230988491	TC
NA12872	75	TT	1	tt	0.285434056	TT
NA12865	76	TC	0.9999	tc	0.285635634	TC
NA10860	77		NA	TT	0.206271361	
NA12873	78	TT	1	tt	0.261825196	TT
NA12752	79	TT	1	tt	0.420753922	TT
NA10851	80	CC	1	cc	0.366589684	CC
NA07029	81	TT	1	tt	0.456022479	TT
NA12248	82	TT	1	tt	0.404960837	TT
NA12155	83	CC	1	cc	0.016272988	CC
NA10838	84	TT	0.9988	tt	0.089996827	TT
NA12812	85	TC	0.9998	tc	0.213638202	TC
NA12248.dup	86	TT	0.9996	tt	0.231277902	nn
NA12043	87	TC	0.9961	tc	0.184876425	TC
NA12234	88	TT	1	tt	0.352945126	TT

	MACGT discrepancy					
	LDA discrepancy					
NA	LDA output were not g					
XY	Manual calls are given					
		**1258464**				
**Coriell**	**Sample**	**LDA Call**	**LDA quality score**	**MACGT Call**	**MACGT quality score**	**HapMap Call**
NA12892	89	TC	0.9732	tc	0.008910422	TC
NA10846	90	TC	0.9972	tc	0.409596185	TC
NA06993.dup	91	TC	0.9999	tc	0.574685663	nn
NA12249	92	TC	1	tc	0.14386559	TC
NA11881	93	CC	1	cc	0.103159475	CC
NA12815	94	TT	0.9894	tt	0.034417862	TT
NA12264	95	TC	1	tc	0.478787234	TC
NA18526	96	TC	0.9999	tc	0.56654684	TC
NA18562	97	CC	1	cc	0.350158876	CC
NA18545	98	CC	1	cc	0.490137531	CC
NA18609	99	CC	1	cc	0.19252375	CC
NA18566	100	TT	0.9997	tt	0.049511266	TT
NA18621	101	TC	0.9999	tc	0.068076949	TC
NA18577	102	CC	1	cc	0.32609509	CC
NA18635	103	CC	1	cc	0.329818855	CC
NA18524	104	CC	1	cc	0.033520153	CC
NA18537	105	CC	1	cc	0.311872874	CC
NA18572	106	CC	0.9977	cc	0.31189437	CC
NA18552	107	TC	0.9997	tc	0.149147389	TC
NA18563	108	TC	1	tc	0.20919493	TC
NA18594	109	CC	1	cc	0.311982063	nn
NA18624	110	CC	0.9953	cc	0.198339162	CC
NA18592	111	CC	1	cc	0.302988616	CC
NA18529	112	CC	0.9939	cc	0.4197873	CC
NA18603	113	CC	0.9997	cc	0.192172042	nn
NA18547	114	CC	0.9998	cc	0.268947519	CC
NA18611	115	TC	0.9995	tc	0.003348466	TC
NA18570	116	CC	0.8862	cc	0.035716612	CC
NA18622	117	CC	1	***CC***	***0***	CC
NA18579	118	TC	0.9999	tc	0.001056076	TC
NA18636	119	CC	0.9993	cc	0.084979811	CC
NA18558	120	CC	0.9522	cc	0.311352061	CC
NA18540	121	CC	0.9997	cc	0.003614289	CC
NA18609	122	CC	0.9998	cc	0.176701635	nn
NA18555	123	CC	0.9998	cc	0.011022963	CC
NA18612	124	CC	0.9999	cc	0.404732896	CC
NA18573	125	CC	0.9973	cc	0.039886755	CC
NA18632	126	CC	1	cc	0.177089483	CC
NA18593	127	TC	1	tc	0.283700426	TC
NA18532	128	CC	1	cc	0.238010906	CC
NA18605	129	TC	0.9999	tc	0.567033097	TC
NA18550	130	CC	1	cc	0.246168478	CC
NA18603	131	CC	1	cc	0.411130033	CC
NA18571	132	TC	0.9999	tc	0.533432038	TC

	MACGT discrepancy					
	LDA discrepancy					
NA	LDA output were not g					
XY	Manual calls are given					
		**1258464**				
**Coriell**	**Sample**	**LDA Call**	**LDA quality score**	**MACGT Call**	**MACGT quality score**	**HapMap Call**
NA18623	133	CC	1	cc	0.262305154	CC
NA18582	134	CC	1	cc	0.321843749	CC
NA18637	135	TC	0.9995	tc	0.173012022	TC
NA18561	136	TC	0.9999	tc	0.276020078	TC
NA18542	137	CC	1	cc	0.207311714	CC
NA18608	138	CC	0.9752	cc	0.351163414	CC
NA18564	139	CC	1	cc	0.203987641	CC
NA18620	140	CC	1	cc	0.518459304	CC
NA18576	141	TC	1	tc	0.672195461	TC
NA18633	142	CC	1	cc	0.513828365	CC
NA18594	143	CC	1	cc	0.309333427	CC
NA18942	144	TC	1	tc	0.464935043	TC
NA18945	145	CC	1	cc	0.295247506	CC
NA18964	146	TC	1	tc	0.538818171	TC
NA18961	147	CC	1	***CC***	***0***	CC
NA18967	148	CC	1	cc	0.433893906	CC
NA18981	149	CC	1	cc	0.24904662	CC
NA18994	150	TC	1	tc	0.47294269	TC
NA18998	151	TC	0.9999	tc	0.120574545	TC
NA18940	152	CC	0.9994	cc	0.31286022	CC
NA18949	153	TC	0.9999	tc	0.236907695	TC
NA18953	154	TC	0.9999	tc	0.029998015	TC
NA18972	155	TT	1	tt	0.127261897	TT
NA18976	156	TC	0.9998	tc	0.237931203	TC
NA18971	157	CC	1	cc	0.413586178	CC
blank	158	CC	0.75	***NN***	***0***	nn
NA19000	159	TC	0.9999	tc	0.171937194	TC
NA18951	160	CC	1	cc	0.371262333	nn
NA18948	161	TC	0.9354	tc	0.035915658	TC
NA18968	162	CC	1	cc	0.178101565	CC
NA18965	163	TC	0.9999	tc	0.162524843	TC
NA18978	164	CC	1	cc	0.426020799	CC
NA18974	165	CC	1	***CC***	***4.94E-04***	CC
NA18992	166	CC	1	cc	0.153686199	CC
NA19005	167	CC	1	cc	0.174459579	CC
NA18943	168	CC	0.9185	cc	0.066378753	CC
NA18951	169	CC	0.948	***CC***	***0***	CC
NA18959	170	CC	1	cc	0.189141343	CC
NA18973	171	TC	1	tc	0.366105707	TC
NA18970	172	CC	0.9999	cc	0.211442065	CC
NA18987	173	CC	1	cc	0.410264744	CC
NA18995	174	CC	1	cc	0.30857186	CC
NA18999	175	TC	1	tc	0.126264452	TC
NA18947	176	CC	1	cc	0.071469291	CC

	MACGT discrepancy					
	LDA discrepancy					
NA	LDA output were not g					
XY	Manual calls are given					
		**1258464**				
**Coriell**	**Sample**	**LDA Call**	**LDA quality score**	**MACGT Call**	**MACGT quality score**	**HapMap Call**
NA18952	177	TC	1	tc	0.54784137	TC
NA18969	178	CC	1	cc	0.302226688	CC
NA18966	179	CC	0.9999	cc	0.317874707	CC
NA18980	180	CC	1	cc	0.344258111	CC
NA18990	181	CC	1	cc	0.303936751	CC
NA18997	182	TC	1	tc	0.644349881	TC
NA19007	183	CC	1	cc	0.219251722	CC
NA18944	184	CC	0.9927	cc	0.005944671	CC
NA18956	185	TT	0.9993	tt	0.214199242	TT
NA18960	186	TC	1	tc	0.570788906	TC
NA18975	187	CC	0.8868	cc	0.039752263	CC
NA18995	188	CC	1	cc	0.151366422	nn
NA18991	189	CC	1	cc	0.155091997	CC
NA18996	190	CC	0.997	cc	0.144141132	nn
NA19003	191	TC	1	tc	0.421730387	TC
NA18502	192	TC	1	tc	0.27775372	TC
NA19153	193	TC	0.9999	tc	0.264289253	TC
NA18857	194	TT	1	tt	0.004758962	TT
NA19223	195	TT	0.9999	tt	0.301648616	TT
NA19201	196	CC	0.9999	cc	0.086390399	CC
NA18504	197	TC	1	tc	0.672827709	TC
NA18870	198	TT	0.9995	tt	0.177582451	TT
NA18863	199	TC	1	tc	0.207323479	TC
NA19145	200	TC	0.9998	tc	0.195500888	TC
NA19137	201	TC	1	tc	0.126158436	TC
NA19238	202	TC	0.9973	tc	0.062284529	nn
NA18500	203	CC	0.8105	cc	0.002956878	CC
NA19144	204	TC	0.9994	tc	0.001319488	TC
NA19203	205	TC	0.9818	tc	0.099160011	TC
NA19200	206	CC	0.9427	cc	0.213043713	CC
NA18855	207	TC	1	tc	0.244941448	TC
NA18505	208	CC	1	cc	0.048565647	CC
NA19202	209	CC	1	cc	0.235074787	CC
NA18501	210	CC	0.9993	cc	0.113112352	CC
NA18861	211	CC	0.9979	cc	0.134789749	CC
NA19193	212	CC	0.9999	cc	0.049080635	CC
NA19143	213	TC	0.9934	tc	0.149919786	TC
NA18517	214	TC	0.9993	tc	0.21309036	TC
NA18862	215	TC	0.9992	tc	0.224677954	TC
NA18856	216	TT	0.9491	***TT***	***0***	nn
NA19239	217	TC	0.9428	tc	0.141593538	TC
NA19240	218	TT	1	tt	0.253186292	TT
NA18856	219	TT	0.9999	tt	0.019435298	TT
NA18503	220	CC	0.9987	cc	0.129746073	CC

	MACGT discrepancy					
	LDA discrepancy					
NA	LDA output were not g					
XY	Manual calls are given					
		**1258464**				
**Coriell**	**Sample**	**LDA Call**	**LDA quality score**	**MACGT Call**	**MACGT quality score**	**HapMap Call**
NA18871	221	TC	1	tc	0.260010792	TC
NA19221	222	TC	0.9999	tc	0.383663132	TC
NA19209	223	TC	1	tc	0.294512328	TC
NA19152	224	TT	1	tt	0.283678536	TT
NA18515	225	TC	1	tc	0.127196706	TC
NA19238	226	TC	1	tc	0.364826337	TC
NA19154	227	TC	1	tc	0.578836454	TC
NA19210	228	TC	1	tc	0.180657572	TC
NA19211	229	TT	0.9969	tt	0.05400734	TT
NA18862	230	TC	0.9772	tc	0.05853356	nn
NA18872	231	TC	0.9999	tc	0.310726127	TC
NA19139	232	CC	0.9062	cc	0.417109448	CC
NA19222	233	CC	0.9914	cc	0.296914646	CC
NA19194	234	CC	0.9998	cc	0.403963428	CC
NA19138	235	CC	1	cc	0.098244843	CC
NA19204	236	TC	1	tc	0.43354924	TC
NA18516	237	CC	0.8927	cc	0.031822122	CC
NA19205	238	TC	0.9973	tc	0.379369185	TC
NA19192	239	TC	0.9999	tc	0.007153045	TC
NA18912	240	TC	0.9998	tc	0.158174193	nn
NA18508	241	TC	0.9997	tc	0.522825088	TC
NA19142	242	TC	0.9994	tc	0.10148626	TC
NA18852	243	TT	1	tt	0.373797059	TT
NA18507	244	TC	1	tc	0.097472109	TC
blank	245	NN	0.4794	***NN***	***0***	nn
NA19101	246	CC	0.9989	cc	0.258835996	CC
NA19172	247	TC	0.9998	tc	0.038659513	TC
NA19160	248	CC	0.8671	cc	0.141501154	CC
NA19129	249	TT	0.9986	tt	0.030771615	TT
NA18913	250	TC	0.9998	tc	0.425424512	TC
NA19120	251	TT	0.8743	***TT***	***0***	TT
NA19159	252	TT	0.9984	tt	0.034885386	TT
NA18523	253	TT	1	tt	0.442183542	TT
NA19102	254	TC	1	***TC***	***6.91E-04***	TC
NA19092	255	CC	1	cc	0.376917931	CC
NA18521	256	TC	1	tc	0.18153618	TC
NA18912	257	TC	1	tc	0.019030595	TC
NA19206	258	TT	0.9998	tt	0.303157969	TT
NA19128	259	TC	1	tc	0.439725981	TC
NA18859	260	TC	1	tc	0.178955922	nn
NA19094	261	TC	1	tc	0.399153057	TC
NA19171	262	TC	1	tc	0.44164618	TC
NA18860	263	TT	1	tt	0.165159365	TT
NA18858	264	TC	1	tc	0.369197739	TC

	MACGT discrepancy					
	LDA discrepancy					
NA	LDA output were not g					
XY	Manual calls are given					
		1258464				
**Coriell**	**Sample**	**LDA Call**	**LDA quality score**	**MACGT Call**	**MACGT quality score**	**HapMap Call**
NA19099	265	CC	1	cc	0.31325283	CC
NA18506	266	TC	1	tc	0.525078804	TC
NA19131	267	TC	1	tc	0.477315799	TC
NA19116	268	TT	1	tt	0.319957926	TT
NA19161	269	TC	1	tc	0.342321472	TC
NA19140	270	TC	1	tc	0.47981301	TC
NA19207	271	CC	0.9999	cc	0.104085692	CC
NA19141	272	TC	1	tc	0.360281526	TC
NA18854	273	TC	1	tc	0.458447628	TC
NA19098	274	TC	1	tc	0.467663641	TC
NA18522	275	TC	1	tc	0.134965151	TC
NA18914	276	TC	0.9997	tc	0.173163183	TC
NA18853	277	TC	0.9977	tc	0.190105621	TC
NA18859	278	TC	0.9979	tc	0.123120697	TC
NA19100	279	TC	1	tc	0.394498109	TC
NA19093	280	TC	1	tc	0.256568236	TC
NA19132	281	TC	1	tc	0.208708191	TC
NA19130	282	CC	1	cc	0.25441521	CC
NA19208	283	TC	1	tc	0.094845566	TC
NA19127	284	TC	0.9819	tc	0.05903348	TC
NA19173	285	TT	0.9998	tt	0.150849147	TT
NA19119	286	TC	1	tc	0.213159171	TC
NA19103	287	CC	1	cc	0.142429592	CC
	Discrepancies	0		0		
	NNs	0		1		
	Automated Calls	278		268		
	Manual Calls	0		10		
	Concordant Calls	259		259		259

	MACGT discrepancy					
	LDA discrepancy					
NA	LDA output were not g					
XY	Manual calls are given					
		1347423				
**Coriell**	**Sample**	**LDA Call**	**LDA quality score**	**MACGT Call**	**MACGT quality score**	**HapMap Call**
NA12753	1		NA	CA	0.40906073	
NA12875	2	CC	1	cc	0.375582382	CC
NA12044	3	CA	0.9999	ca	0.554140032	CA
NA06993	4	CA	0.9831	ca	0.021315711	CA
NA12716	5	CC	1	cc	0.427977326	CC
NA12760	6	CA	1	ca	0.256529566	CA
NA07348	7	CA	1	ca	0.532418942	CA
NA12707	8	CC	1	cc	0.459869051	CC
NA12156	9		NA	CA	0.173862906	
NA11992	10	CA	0.9998	ca	0.286004501	CA
NA12239	11	AA	0.9911	aa	0.073478836	AA
NA12878	12	CA	0.9998	ca	0.377812787	CA
NA11993	13	AA	1	aa	0.020803415	AA
NA12750	14	CA	0.9995	ca	0.027098207	CA
NA12146	15	AA	0.996	aa	0.063942875	AA
NA11839	16	CC	1	cc	0.355381556	CC
NA12156.dup	17	CA	1	ca	0.002900572	nn
NA11829	18		NA	CA	0.186395378	
NA12154	19	AA	1	aa	0.164630644	AA
NA10856	20	AA	0.9999	aa	0.139367163	AA
NA06985	21	CA	1	ca	0.015259503	CA
NA11831	22	CC	1	cc	0.453577833	CC
NA12003.dup	23	CC	1	cc	0.358554753	nn
NA10859	24	CC	1	cc	0.470115814	CC
NA07056	25	CC	1	cc	0.500572045	CC
NA07022	26	AA	1	aa	0.288561098	AA
NA12762	27		NA	CC	0.522736624	
NA12874	28	CC	1	cc	0.408738061	CC
NA12003	29	CC	1	cc	0.446177769	CC
NA10835	30	AA	1	aa	0.134241997	AA
NA11882	31	CA	0.9999	ca	0.075440177	CA
NA07034	32	CC	1	cc	0.444575587	CC
NA12056	33	AA	1	aa	0.139294086	AA
NA12145	34	CA	0.9999	ca	0.435992699	CA
NA07019	35	CA	1	ca	0.443189569	CA
NA06991	36		NA	CA	0.427084854	
NA12761	37	CC	1	cc	0.442084079	CC
NA06994	38	CA	0.9979	ca	0.324689911	CA
NA12864	39	AA	0.9997	aa	0.327671081	AA
NA07055	40	CA	1	ca	0.361410306	CA
NA10863	41	CC	1	cc	0.499165075	CC
NA12763	42	CA	1	ca	0.445580421	CA
NA10831	43	CA	0.9997	ca	0.459020026	CA
NA11840	44	CC	1	cc	0.596251533	CC

	MACGT discrepancy					
	LDA discrepancy					
NA	LDA output were not g					
XY	Manual calls are given					
		**1347423**				
**Coriell**	**Sample**	**LDA Call**	**LDA quality score**	**MACGT Call**	**MACGT quality score**	**HapMap Call**
NA10830	45	CA	1	ca	0.511193866	CA
NA11993.dup	46	AA	1	aa	0.262871663	nn
NA12751	47	CC	1	cc	0.613245538	CC
NA12814	48	CA	1	ca	0.313186825	CA
NA10857	49	CC	1	cc	0.439091004	CC
NA07357	50	CA	0.9998	ca	0.292712454	CA
NA07000	51	AA	0.9805	aa	0.069037011	AA
NA12802	52	CA	0.9999	ca	0.408259257	CA
NA10855	53	CA	0.9995	ca	0.387737998	CA
NA11995	54		NA	CC	0.469659276	
NA10839	55	AA	0.9999	aa	0.179053256	AA
NA10847	56	AA	0.9998	aa	0.176463617	AA
NA12006	57	CA	0.9997	ca	0.095033173	CA
NA12144	58	CA	0.9996	ca	0.32374576	CA
NA11832	59	CA	0.9993	ca	0.104353923	CA
NA12813	60	CA	0.9984	ca	0.226015052	CA
blank	61	CA	0.9998	ca	0.289273768	nn
NA12891	62	CC	1	cc	0.699229703	CC
NA12717	63		NA	CC	0.651996747	
NA10861	64	CC	1	***CC***	***6.91E-04***	CC
NA12004	65	CA	0.9992	ca	0.093778587	CA
NA07048	66	CC	1	cc	0.441035165	CC
NA12801	67	CA	0.993	ca	0.045044475	CA
NA11830	68	AA	0.9654	***AA***	***0***	AA
NA12057	69	CA	0.9998	ca	0.195908961	CA
NA11994	70		NA	CC	0.577680489	
NA12740	71	CA	0.9999	ca	0.481952775	CA
NA12005	72	CA	1	ca	0.013672234	CA
NA07345	73	CC	1	cc	0.477201789	CC
NA10854	74	CC	1	cc	0.579913079	CC
NA12872	75	AA	1	aa	0.331517092	AA
NA12865	76	CC	1	cc	0.60262573	CC
NA10860	77		NA	AA	0.203854513	
NA12873	78	AA	1	aa	0.276406503	AA
NA12752	79	CA	1	ca	0.397384624	CA
NA10851	80	AA	1	aa	0.404959523	AA
NA07029	81	CA	1	ca	0.089715116	CA
NA12248	82	CA	0.9992	ca	0.361818001	CA
NA12155	83	CA	0.9998	ca	0.025627072	CA
NA10838	84	CA	1	ca	0.299912304	CA
NA12812	85	CC	1	cc	0.607557352	CC
NA12248.dup	86	CA	0.9996	ca	0.138811842	nn
NA12043	87	CA	1	ca	0.195885007	CA
NA12234	88	CC	1	cc	0.509144169	CC

	MACGT discrepancy					
	LDA discrepancy					
NA	LDA output were not g					
XY	Manual calls are given					
		**1347423**				
**Coriell**	**Sample**	**LDA Call**	**LDA quality score**	**MACGT Call**	**MACGT quality score**	**HapMap Call**
NA12892	89		NA	AA	0.502576875	
NA10846	90		NA	AA	0.482518539	
NA06993.dup	91	CA	1	ca	0.155777026	nn
NA12249	92	CA	1	ca	0.113235377	CA
NA11881	93	CC	1	cc	0.382601595	CC
NA12815	94	CC	1	*CC*	*0*	CC
NA12264	95	CC	1	cc	0.508969154	CC
NA18526	96	CA	1	ca	0.431172486	CA
NA18562	97	CC	1	cc	0.599303497	CC
NA18545	98	CC	1	cc	0.518024562	CC
NA18609	99	CC	1	cc	0.040665684	CC
NA18566	100	CA	0.9997	ca	0.344467984	CA
NA18621	101	CC	1	cc	0.587772532	CC
NA18577	102	CC	1	cc	0.551603256	CC
NA18635	103	CC	1	cc	0.610816552	CC
NA18524	104	CC	1	cc	0.640126759	CC
NA18537	105	CC	1	cc	0.52969802	CC
NA18572	106	CA	0.9994	ca	0.296474356	CA
NA18552	107	CA	0.9988	ca	0.195035582	CA
NA18563	108		NA	AA	0.355607232	
NA18594	109	CA	1	ca	0.083200256	nn
NA18624	110	CA	0.9999	ca	0.361687312	CA
NA18592	111	CA	0.9927	ca	0.370488924	CA
NA18529	112	CC	1	cc	0.675987526	CC
NA18603	113	CA	0.9982	ca	0.1972883	nn
NA18547	114	CC	1	cc	0.431978807	CC
NA18611	115	CA	1	ca	0.065335091	CA
NA18570	116	CC	1	cc	0.597051945	CC
NA18622	117	CA	0.9927	ca	0.465348294	CA
NA18579	118	CC	1	cc	0.381110576	CC
NA18636	119	CC	1	cc	0.670990912	CC
NA18558	120	CA	0.9995	ca	0.357052026	CA
NA18540	121	CC	1	cc	0.599093586	CC
NA18609	122	CC	1	cc	0.649501861	nn
NA18555	123	CC	1	cc	0.420208059	CC
NA18612	124	CA	0.9997	ca	0.450668035	CA
NA18573	125	CC	1	cc	0.617486169	CC
NA18632	126	CA	1	ca	0.307727299	CA
NA18593	127	CA	0.9999	ca	0.275671917	CA
NA18532	128	CA	0.9981	ca	0.327533926	CA
NA18605	129	CA	0.9994	ca	0.418120587	CA
NA18550	130	AA	1	aa	0.206269273	AA
NA18603	131	CA	0.9971	ca	0.410514328	CA
NA18571	132	CC	1	cc	0.45658902	CC

	MACGT discrepancy					
	LDA discrepancy					
NA	LDA output were not g					
XY	Manual calls are given					
		**1347423**				
**Coriell**	**Sample**	**LDA Call**	**LDA quality score**	**MACGT Call**	**MACGT quality score**	**HapMap Call**
NA18623	133	CC	1	cc	0.584374143	CC
NA18582	134	CC	1	cc	0.456501383	CC
NA18637	135	CA	0.9995	ca	0.472097824	CA
NA18561	136	CA	0.9996	ca	0.289610449	CA
NA18542	137	CC	1	cc	0.566374843	CC
NA18608	138	CC	1	cc	0.028835046	CC
NA18564	139	CA	0.9997	ca	0.403376437	CA
NA18620	140	CA	1	ca	0.498078403	CA
NA18576	141	CC	1	cc	0.034913277	CC
NA18633	142	CA	0.9996	ca	0.195870877	CA
NA18594	143	CA	0.9979	ca	0.466633675	CA
NA18942	144	CC	1	cc	0.58537835	CC
NA18945	145	CC	1	cc	0.454705617	CC
NA18964	146	CA	1	ca	0.532634281	CA
NA18961	147	CA	0.9999	ca	0.264151832	CA
NA18967	148	CC	1	cc	0.599885853	CC
NA18981	149	CC	1	cc	0.585448333	CC
NA18994	150	CC	1	cc	0.023901409	CC
NA18998	151	CA	1	ca	0.115822755	CA
NA18940	152	CA	0.9998	ca	0.306607204	CA
NA18949	153	CC	1	cc	0.035909425	CC
NA18953	154	CC	1	cc	0.180253397	CC
NA18972	155	CC	1	cc	0.465806336	CC
NA18976	156	CC	1	cc	0.533849694	CC
NA18971	157	CC	1	cc	0.368992431	CC
blank	158	CC	1	*NN*	*0*	nn
NA19000	159	CA	0.9999	ca	0.402993168	CA
NA18951	160	CC	1	cc	0.506737462	nn
NA18948	161	CA	1	ca	0.198723642	CA
NA18968	162	CA	0.9998	ca	0.534966468	CA
NA18965	163	CA	1	ca	0.376648541	CA
NA18978	164	CA	0.9997	ca	0.370539723	CA
NA18974	165		NA	AA	0.33622008	
NA18992	166	CC	1	cc	0.322569918	CC
NA19005	167	CA	1	ca	0.575666531	CA
NA18943	168	CA	0.9999	ca	0.275309682	CA
NA18951	169	CC	1	cc	0.592312741	CC
NA18959	170	CA	0.9999	ca	0.478133053	CA
NA18973	171	CC	1	cc	0.688095748	CC
NA18970	172	CA	1	ca	0.360497014	CA
NA18987	173	CA	0.9995	ca	0.331598351	CA
NA18995	174	CC	1	cc	0.668319989	CC
NA18999	175	CA	0.9997	ca	0.162718993	CA
NA18947	176	CA	0.9999	ca	0.284163413	CA

	MACGT discrepancy					
	LDA discrepancy					
NA	LDA output were not g					
XY	Manual calls are given					
		1347423				
**Coriell**	**Sample**	**LDA Call**	**LDA quality score**	**MACGT Call**	**MACGT quality score**	**HapMap Call**
NA18952	177	CC	1	cc	0.683622552	CC
NA18969	178	CC	1	cc	0.720883701	CC
NA18966	179	CA	0.9996	ca	0.293660643	CA
NA18980	180	AA	0.9992	aa	0.225876249	AA
NA18990	181	CC	1	cc	0.66605999	CC
NA18997	182	CC	1	cc	0.255232404	CC
NA19007	183	CC	1	cc	0.413363226	CC
NA18944	184	CC	1	cc	0.195782221	CC
NA18956	185	CC	1	cc	0.547958408	CC
NA18960	186	CC	1	cc	0.646985823	CC
NA18975	187	CA	0.9999	ca	0.185547453	CA
NA18995	188	CC	1	cc	0.55466158	nn
NA18991	189	CA	0.9994	ca	0.315566262	CA
NA18996	190	CC	1	cc	0.568580508	nn
NA19003	191	CC	1	cc	0.681642775	CC
NA18502	192	CC	1	cc	0.41580281	CC
NA19153	193	CC	1	cc	0.502444258	CC
NA18857	194	CC	1	cc	0.650983701	CC
NA19223	195	CC	1	cc	0.570040472	CC
NA19201	196	CC	1	cc	0.624517763	CC
NA18504	197	CC	1	cc	0.310852482	CC
NA18870	198	CC	1	cc	0.490541448	CC
NA18863	199	CC	1	cc	0.30098693	CC
NA19145	200	CC	1	cc	0.494493306	CC
NA19137	201	CC	1	cc	0.482635263	CC
NA19238	202	CC	1	cc	0.273519918	nn
NA18500	203	CC	1	cc	0.46608103	CC
NA19144	204	CC	1	cc	0.526642078	CC
NA19203	205	CC	1	cc	0.58881919	CC
NA19200	206		NA	CC	0.576547339	
NA18855	207	CC	1	cc	0.267682697	CC
NA18505	208	CC	1	cc	0.402230528	CC
NA19202	209	CC	1	cc	0.007936535	CC
NA18501	210	CC	1	cc	0.069399257	CC
NA18861	211	CC	1	cc	0.504075911	CC
NA19193	212	CC	1	cc	0.587531261	CC
NA19143	213	CC	1	cc	0.53255531	CC
NA18517	214	CC	1	cc	0.196638598	CC
NA18862	215	CC	1	cc	0.453600776	CC
NA18856	216	CC	1	cc	0.506245354	nn
NA19239	217	CC	1	cc	0.5159866	CC
NA19240	218	CC	1	cc	0.232405873	CC
NA18856	219	CC	1	cc	0.367980441	CC
NA18503	220	CC	1	cc	0.294629076	CC

	MACGT discrepancy					
	LDA discrepancy					
NA	LDA output were not g					
XY	Manual calls are given					
		**1347423**				
**Coriell**	**Sample**	**LDA Call**	**LDA quality score**	**MACGT Call**	**MACGT quality score**	**HapMap Call**
NA18871	221	CC	1	cc	0.142924656	CC
NA19221	222	CC	1	cc	0.672239711	CC
NA19209	223	CC	1	cc	0.514557349	CC
NA19152	224	CC	1	cc	0.059716917	CC
NA18515	225	CC	1	cc	0.509886823	CC
NA19238	226	CC	1	cc	0.311562858	CC
NA19154	227	CC	1	cc	0.69779837	CC
NA19210	228	CC	0.9998	cc	0.194950582	CC
NA19211	229	CC	1	cc	0.501674967	CC
NA18862	230	CC	1	cc	0.513361261	nn
NA18872	231	CC	1	cc	0.614176983	CC
NA19139	232	CC	1	cc	0.182215833	CC
NA19222	233	CC	1	cc	0.581115631	CC
NA19194	234	CC	1	cc	0.632445818	CC
NA19138	235	CC	1	cc	0.311206135	CC
NA19204	236	CC	1	cc	0.41600529	CC
NA18516	237	CC	1	cc	0.460811401	CC
NA19205	238	CC	0.9999	cc	0.442122107	CC
NA19192	239	CA	1	ca	0.261142374	CA
NA18912	240	CC	1	cc	0.595218505	nn
NA18508	241	CC	1	cc	0.440449575	CC
NA19142	242	CC	1	cc	0.272836545	CC
NA18852	243	CC	1	cc	0.539404571	CC
NA18507	244	CC	1	cc	0.559139998	CC
blank	245	CC	0.75	*NN*	*0*	nn
NA19101	246	CC	1	cc	0.537235741	CC
NA19172	247	CC	1	cc	0.113794395	CC
NA19160	248	CC	1	cc	0.170549154	CC
NA19129	249	CC	1	cc	0.523624826	CC
NA18913	250	CC	1	cc	0.395034172	CC
NA19120	251	CC	1	cc	0.407946465	CC
NA19159	252	CC	1	cc	0.52136401	CC
NA18523	253	CC	1	cc	0.61135277	CC
NA19102	254	CC	1	cc	0.342536567	CC
NA19092	255	CC	1	cc	0.500311538	CC
NA18521	256	CC	1	cc	0.52490154	CC
NA18912	257	CC	1	cc	0.184438446	CC
NA19206	258	CC	1	cc	0.54746258	CC
NA19128	259	CC	1	cc	0.563367714	CC
NA18859	260	CC	1	cc	0.568449016	nn
NA19094	261	CC	1	cc	0.612446117	CC
NA19171	262	CC	1	cc	0.497061578	CC
NA18860	263	CC	1	cc	0.274699686	CC
NA18858	264	CC	1	cc	0.566338895	CC

	MACGT discrepancy					
	LDA discrepancy					
NA	LDA output were not g					
XY	Manual calls are given					
		**1347423**				
**Coriell**	**Sample**	**LDA Call**	**LDA quality score**	**MACGT Call**	**MACGT quality score**	**HapMap Call**
NA19099	265	CC	1	cc	0.436531648	CC
NA18506	266	CC	1	cc	0.374718796	CC
NA19131	267	CC	1	cc	0.492711745	CC
NA19116	268	CC	1	cc	0.421889752	CC
NA19161	269	CC	1	cc	0.050064575	CC
NA19140	270	CC	1	cc	0.4420126	CC
NA19207	271	CC	1	cc	0.561893122	CC
NA19141	272	CC	1	cc	0.002082133	CC
NA18854	273	CC	1	cc	0.466206428	CC
NA19098	274	CC	1	cc	0.48917465	CC
NA18522	275	CC	1	cc	0.342890525	CC
NA18914	276	CC	1	cc	0.202470247	CC
NA18853	277	CC	0.9898	cc	0.284181977	CC
NA18859	278	CC	1	cc	0.348681781	CC
NA19100	279	CC	1	cc	0.081969376	CC
NA19093	280	CC	1	cc	0.245287775	CC
NA19132	281	CC	1	cc	0.297011505	CC
NA19130	282	CC	1	cc	0.304072671	CC
NA19208	283	CC	1	cc	0.068908655	CC
NA19127	284	CC	1	cc	0.156908538	CC
NA19173	285	CC	1	cc	0.602019871	CC
NA19119	286	CC	1	cc	0.167088859	CC
NA19103	287	CC	1	cc	0.021147367	CC
	Discrepancies	0		0		
	NNs	-2		0		
	Automated Calls	273		268		
	Manual Calls	0		5		
	Concordant Calls	254		254		254

	MACGT discrepancy					
	LDA discrepancy					
NA	LDA output were not g					
XY	Manual calls are given					
		**1366660**				
**Coriell**	**Sample**	**LDA Call**	**LDA quality score**	**MACGT Call**	**MACGT quality score**	**HapMap Call**
NA12753	1		NA	AG	0.346409938	
NA12875	2	GG	0.986	gg	0.190970969	GG
NA12044	3	AA	1	aa	0.246248978	AA
NA06993	4	AG	0.9985	ag	0.369961445	AG
NA12716	5	GG	0.9014	gg	0.127785656	GG
NA12760	6	GG	0.9999	gg	0.521583268	GG
NA07348	7	GG	1	gg	0.254127449	GG
NA12707	8	GG	0.9999	gg	0.186537149	GG
NA12156	9		NA	AG	0.144913039	
NA11992	10	GG	0.9999	gg	0.302951179	GG
NA12239	11	GG	0.9982	gg	0.224408165	GG
NA12878	12	AG	0.9995	ag	0.559597704	AG
NA11993	13	AG	1	ag	0.01454399	AG
NA12750	14	AG	0.9999	ag	0.104034481	AG
NA12146	15	AG	0.9999	ag	0.15644079	AG
NA11839	16	AA	0.9873	aa	0.003991918	AA
NA12156.dup	17	AG	0.9994	ag	0.395746064	nn
NA11829	18		NA	GG	0.350607587	
NA12154	19	AA	1	aa	0.103604891	AA
NA10856	20	GG	0.9997	gg	0.126935071	GG
NA06985	21	GG	1	gg	0.033300511	GG
NA11831	22	AG	1	ag	0.049241655	AG
NA12003.dup	23	AA	0.9976	aa	0.087051841	nn
NA10859	24	AG	0.9997	ag	0.232815447	AG
NA07056	25	AG	0.9995	ag	0.226983781	AG
NA07022	26	AG	1	ag	0.146936807	AG
NA12762	27		NA	GG	0.46410286	
NA12874	28	AG	0.9999	ag	0.239783229	AG
NA12003	29	AA	1	aa	0.191057857	AA
NA10835	30	AA	1	aa	0.134020734	AA
NA11882	31	GG	0.9982	gg	0.25884255	GG
NA07034	32	AG	0.9986	ag	0.087365261	AG
NA12056	33	AG	0.9997	ag	0.31107907	AG
NA12145	34	GG	0.995	gg	0.282077285	GG
NA07019	35	AG	0.9999	ag	0.721709521	AG
NA06991	36		NA	AG	0.747935684	
NA12761	37	GG	0.9987	gg	0.202307423	GG
NA06994	38	GG	0.9919	gg	0.281184938	GG
NA12864	39	AG	1	ag	0.634822001	AG
NA07055	40	AG	1	ag	0.337658074	AG
NA10863	41	GG	0.968	gg	0.276033081	GG
NA12763	42	AG	1	ag	0.694440506	AG
NA10831	43	AG	1	ag	0.252478145	AG
NA11840	44	AG	1	ag	0.418707943	AG

	MACGT discrepancy					
	LDA discrepancy					
NA	LDA output were not g					
XY	Manual calls are given					
		**1366660**				
**Coriell**	**Sample**	**LDA Call**	**LDA quality score**	**MACGT Call**	**MACGT quality score**	**HapMap Call**
NA10830	45	AA	1	aa	0.218620388	AA
NA11993.dup	46	AG	0.9999	ag	0.535083231	nn
NA12751	47	AG	1	ag	0.54576641	AG
NA12814	48	GG	0.9809	gg	0.122020661	GG
NA10857	49	AG	1	ag	0.434950469	AG
NA07357	50	GG	1	gg	0.46082336	GG
NA07000	51	AG	0.9995	ag	0.05720656	AG
NA12802	52	GG	0.8667	gg	0.112653746	GG
NA10855	53	AG	1	ag	0.213270531	AG
NA11995	54		NA	AG	0.334302541	
NA10839	55	AG	1	ag	0.386917892	AG
NA10847	56	AG	0.9997	ag	0.412832931	AG
NA12006	57	AG	1	ag	0.230971277	AG
NA12144	58	AG	0.9999	ag	0.474263591	AG
NA11832	59	GG	0.9981	gg	0.23022032	GG
NA12813	60	AG	0.9998	ag	0.33369229	AG
blank	61	AG	1	ag	0.282615445	nn
NA12891	62	AG	0.9999	ag	0.27140011	AG
NA12717	63		NA	GG	0.324103111	
NA10861	64	AG	0.9999	ag	0.299126705	AG
NA12004	65	AG	0.9996	ag	0.044426455	AG
NA07048	66	AG	0.9999	ag	0.295206106	AG
NA12801	67	GG	0.9967	gg	0.09818527	GG
NA11830	68	AG	0.9975	ag	0.305388628	AG
NA12057	69	GG	0.9933	gg	0.304287866	GG
NA11994	70		NA	AG	0.600254234	
NA12740	71	AG	1	ag	0.558569147	AG
NA12005	72	GG	0.9999	gg	0.065000755	GG
NA07345	73	AG	0.9996	ag	0.081942581	AG
NA10854	74	AG	0.9999	ag	0.280195746	AG
NA12872	75	AG	1	ag	0.464340131	AG
NA12865	76	AG	0.9999	ag	0.662798019	AG
NA10860	77		NA	GG	0.211952483	
NA12873	78	AA	1	aa	0.261582125	AA
NA12752	79	GG	0.9998	gg	0.513190973	GG
NA10851	80	AG	1	ag	0.515553783	AG
NA07029	81	GG	0.9998	gg	0.546856423	GG
NA12248	82	AG	0.9972	ag	0.474229935	AG
NA12155	83	AA	1	*AA*	*0*	AA
NA10838	84	AG	0.9999	ag	0.138222703	AG
NA12812	85	AG	0.9987	ag	0.251256409	AG
NA12248.dup	86	AG	0.9999	ag	0.217940539	nn
NA12043	87	AG	0.9998	ag	0.548670328	AG
NA12234	88	GG	0.9999	gg	0.358256099	GG

	MACGT discrepancy					
	LDA discrepancy					
NA	LDA output were not g					
XY	Manual calls are given					
				1366660		
**Coriell**	**Sample**	**LDA Call**	**LDA quality score**	**MACGT Call**	**MACGT quality score**	**HapMap Call**
NA12892	89	GG	0.9998	gg	0.329506208	GG
NA10846	90	AG	1	ag	0.186699691	AG
NA06993.dup	91	AG	0.9999	ag	0.443511363	nn
NA12249	92	AA	0.9921	aa	0.012519046	AA
NA11881	93	AG	1	ag	0.102746417	AG
NA12815	94	GG	0.9998	gg	0.019687996	GG
NA12264	95	AG	1	ag	0.210789164	AG
NA18526	96	AG	1	ag	0.430401492	AG
NA18562	97	GG	0.9736	gg	0.300270844	GG
NA18545	98	AG	0.9999	ag	0.216390752	AG
NA18609	99	AG	0.9997	ag	0.135802315	AG
NA18566	100	AA	0.9998	aa	0.005871519	AA
NA18621	101	AG	0.9995	ag	0.212884159	AG
NA18577	102	AG	0.9998	ag	0.131910485	AG
NA18635	103	GG	0.9907	gg	0.202627139	GG
NA18524	104	AA	1	aa	0.022136939	AA
NA18537	105	GG	0.9994	gg	0.162394423	GG
NA18572	106	AG	1	ag	0.202654676	AG
NA18552	107	AA	0.9997	aa	0.011244007	AA
NA18563	108	AG	0.9998	ag	0.125522917	AG
NA18594	109	AG	1	ag	0.122773726	nn
NA18624	110	AA	1	aa	0.387096683	AA
NA18592	111	AG	1	ag	0.334342072	AG
NA18529	112	AA	1	aa	0.102081514	AA
NA18603	113	AG	0.9997	ag	0.498912623	nn
NA18547	114	AA	1	aa	0.06054239	AA
NA18611	115	AA	0.9999	***AA***	***0***	AA
NA18570	116	GG	0.9877	gg	0.215576753	GG
NA18622	117	AA	1	aa	0.288994585	AA
NA18579	118	AA	1	aa	0.091483813	AA
NA18636	119	GG	0.9999	gg	0.449596507	GG
NA18558	120	GG	0.9964	gg	0.494194939	GG
NA18540	121	AG	0.9999	ag	0.395897559	AG
NA18609	122	AG	0.9998	ag	0.460808292	nn
NA18555	123	GG	0.9302	gg	0.0617003	GG
NA18612	124	AG	0.9895	ag	0.305989628	AG
NA18573	125	AG	1	ag	0.278598919	AG
NA18632	126	AG	1	ag	0.599423116	AG
NA18593	127	AG	0.9999	ag	0.498308413	AG
NA18532	128	AG	1	ag	0.450689804	AG
NA18605	129	AA	1	aa	0.226242671	AA
NA18550	130	AA	1	aa	0.290301707	AA
NA18603	131	AG	0.9988	ag	0.357265803	AG
NA18571	132	AG	1	ag	0.175406995	AG

	MACGT discrepancy					
	LDA discrepancy					
NA	LDA output were not g					
XY	Manual calls are given					
		**1366660**				
**Coriell**	**Sample**	**LDA Call**	**LDA quality score**	**MACGT Call**	**MACGT quality score**	**HapMap Call**
NA18623	133	AG	1	ag	0.418544199	AG
NA18582	134	GG	1	gg	0.55320108	GG
NA18637	135	AA	1	aa	0.1361441	AA
NA18561	136		NA	AA	0.357349196	
NA18542	137	AA	1	aa	0.183815687	AA
NA18608	138	AG	0.9996	ag	0.356857652	AG
NA18564	139	AG	1	ag	0.33231684	AG
NA18620	140	GG	1	gg	0.416552876	GG
NA18576	141	GG	0.9999	gg	0.323122968	GG
NA18633	142	AG	0.9999	ag	0.465855231	AG
NA18594	143	AG	0.9998	ag	0.451294078	AG
NA18942	144	AG	0.9988	ag	0.400227427	AG
NA18945	145	AG	0.9986	ag	0.337303234	AG
NA18964	146		NA	AA	0.431850517	
NA18961	147		NA	AA	0.547434003	
NA18967	148	GG	1	gg	0.516755219	GG
NA18981	149	AA	1	aa	0.398383483	AA
NA18994	150	AA	1	aa	0.460803995	AA
NA18998	151	GG	0.9997	gg	0.015720756	GG
NA18940	152	AA	1	aa	0.190320318	AA
NA18949	153	GG	1	gg	0.174175166	GG
NA18953	154	AG	0.9996	ag	0.09877643	AG
NA18972	155	AA	1	aa	0.134444558	AA
NA18976	156	GG	0.9994	gg	0.227350399	GG
NA18971	157	AA	1	aa	0.420932762	AA
blank	158	NN	0.5386	***NN***	***0***	nn
NA19000	159	AA	1	aa	0.340901671	AA
NA18951	160	AG	0.9135	ag	0.179419869	nn
NA18948	161	AG	0.8043	ag	0.063371016	AG
NA18968	162	AG	0.9945	ag	0.331140686	AG
NA18965	163	AG	0.9967	ag	0.258423833	AG
NA18978	164	AA	1	aa	0.110495825	AA
NA18974	165	AG	0.9967	ag	0.243799855	AG
NA18992	166	AA	1	aa	0.026210865	AA
NA19005	167	AG	0.9998	ag	0.53749151	AG
NA18943	168	AG	0.9998	ag	0.603589469	AG
NA18951	169	AG	1	ag	0.423339463	AG
NA18959	170	AG	0.9998	ag	0.600340942	AG
NA18973	171	AG	0.9994	ag	0.470643526	AG
NA18970	172	AG	0.9993	ag	0.468777069	AG
NA18987	173	GG	0.9999	gg	0.124975106	GG
NA18995	174	AG	0.9997	ag	0.180364523	AG
NA18999	175		NA	AA	0.373532437	
NA18947	176		NA	AA	0.325188224	

	MACGT discrepancy					
	LDA discrepancy					
NA	LDA output were not g					
XY	Manual calls are given					
		1366660				
**Coriell**	**Sample**	**LDA Call**	**LDA quality score**	**MACGT Call**	**MACGT quality score**	**HapMap Call**
NA18952	177	AG	0.9973	ag	0.405871455	AG
NA18969	178	AG	0.9943	ag	0.333538	AG
NA18966	179	AG	0.9953	ag	0.299904682	AG
NA18980	180	AG	0.9975	ag	0.168134054	AG
NA18990	181	AA	1	aa	0.271797733	AA
NA18997	182	AG	0.9988	ag	0.374540282	AG
NA19007	183	AG	0.9975	ag	0.218769788	AG
NA18944	184	AA	1	***AA***	***0***	AA
NA18956	185	AG	0.9998	ag	0.42247623	AG
NA18960	186	GG	0.9953	gg	0.557114162	GG
NA18975	187	AA	1	aa	0.151744999	AA
NA18995	188	AG	0.9959	ag	0.503639977	nn
NA18991	189	AG	0.9994	ag	0.375978079	AG
NA18996	190	AG	0.9986	ag	0.451805741	nn
NA19003	191	AG	0.9996	ag	0.536801619	AG
NA18502	192	AG	0.9999	ag	0.10066992	AG
NA19153	193	AA	1	aa	0.154020646	AA
NA18857	194	AA	1	aa	0.208635739	AA
NA19223	195	GG	0.9999	gg	0.108972424	GG
NA19201	196	AG	0.9999	ag	0.305885983	AG
NA18504	197	AG	1	ag	0.146617934	AG
NA18870	198	AA	1	aa	0.174847247	AA
NA18863	199	GG	0.9989	gg	0.061276898	GG
NA19145	200	AG	0.9999	***ag***	***9.39E-04***	AG
NA19137	201	AG	0.9981	ag	0.096744589	AG
NA19238	202	GG	0.9997	gg	0.032824432	nn
NA18500	203	GG	0.9995	gg	0.148541898	GG
NA19144	204	AG	1	ag	0.075206478	AG
NA19203	205	AG	0.9786	ag	0.235982453	AG
NA19200	206	GG	0.9451	gg	0.248754035	GG
NA18855	207	AA	1	aa	0.003923642	AA
NA18505	208	GG	0.9999	***GG***	***6.24E-04***	GG
NA19202	209	GG	0.9999	gg	0.073040391	GG
NA18501	210	GG	0.9962	gg	0.066098305	GG
NA18861	211	GG	0.9997	gg	0.026209952	GG
NA19193	212	AA	1	aa	0.09683081	AA
NA19143	213	AG	0.9991	ag	0.110102962	AG
NA18517	214	AG	0.9994	ag	0.280106888	AG
NA18862	215	GG	1	gg	0.201137092	GG
NA18856	216	AA	1	aa	0.008200119	nn
NA19239	217	AG	0.9921	ag	0.223451576	AG
NA19240	218	AG	0.9991	ag	0.439063863	AG
NA18856	219	AA	1	aa	0.038330746	AA
NA18503	220	AG	0.9996	ag	0.249979945	AG

	MACGT discrepancy					
	LDA discrepancy					
NA	LDA output were not g					
XY	Manual calls are given					
		**1366660**				
**Coriell**	**Sample**	**LDA Call**	**LDA quality score**	**MACGT Call**	**MACGT quality score**	**HapMap Call**
NA18871	221	AA	1	aa	0.324761369	AA
NA19221	222	AG	0.9987	ag	0.438282459	AG
NA19209	223	AG	0.9999	ag	0.42638849	AG
NA19152	224	AA	1	aa	0.229245931	AA
NA18515	225	AG	0.9997	ag	0.160513814	AG
NA19238	226	GG	0.9859	gg	0.157416579	GG
NA19154	227	AA	1	aa	0.224347469	AA
NA19210	228	AG	0.9981	ag	0.318393149	AG
NA19211	229	AG	0.9904	ag	0.173737497	AG
NA18862	230	GG	0.9869	gg	0.317842227	nn
NA18872	231	AA	1	aa	0.370275713	AA
NA19139	232	GG	0.9997	gg	0.500156476	GG
NA19222	233	AA	1	aa	0.094186365	AA
NA19194	234	AG	0.9976	ag	0.393047242	AG
NA19138	235	AG	0.9719	ag	0.138996257	AG
NA19204	236	AG	0.9997	ag	0.171593554	AG
NA18516	237	AA	1	aa	0.237827455	AA
NA19205	238	AA	1	aa	0.1709012	AA
NA19192	239	GG	0.9993	gg	0.199286359	GG
NA18912	240	AG	0.9991	ag	0.274127719	nn
NA18508	241	GG	0.9896	gg	0.308612264	GG
NA19142	242	AG	0.9996	ag	0.210228449	AG
NA18852	243	AG	0.9976	ag	0.323305116	AG
NA18507	244	AG	0.9908	ag	0.193310415	AG
blank	245	NN	0.506	***NN***	***0***	nn
NA19101	246	AG	0.9987	ag	0.356041451	AG
NA19172	247	AA	1	***AA***	***5.24E-04***	AA
NA19160	248	AA	1	aa	0.00844082	AA
NA19129	249	AG	0.9966	ag	0.261139543	AG
NA18913	250	GG	0.9999	gg	0.268308723	GG
NA19120	251	GG	0.9999	gg	0.039571614	GG
NA19159	252	AA	1	aa	0.112398826	AA
NA18523	253	AA	1	aa	0.370109802	AA
NA19102	254	GG	0.9994	***GG***	***0***	GG
NA19092	255	AG	0.9999	ag	0.633502474	AG
NA18521	256	AA	1	aa	0.164107215	AA
NA18912	257	AG	0.9999	ag	0.130155796	AG
NA19206	258	AA	1	aa	0.077901934	AA
NA19128	259	GG	0.9881	gg	0.454613957	GG
NA18859	260	AG	0.9999	ag	0.519175545	nn
NA19094	261	NN	0.75	***NN***	***0***	AA
NA19171	262	AA	0.75	***NN***	***0***	AA
NA18860	263	GG	0.9995	gg	0.560140551	GG
NA18858	264	GG	1	gg	0.535458427	GG

	MACGT discrepancy					
	LDA discrepancy					
NA	LDA output were not g					
XY	Manual calls are given					
		1366660				
**Coriell**	**Sample**	**LDA Call**	**LDA quality score**	**MACGT Call**	**MACGT quality score**	**HapMap Call**
NA19099	265	AA	1	aa	0.1166622	AA
NA18506	266	GG	0.9985	gg	0.067893852	GG
NA19131	267	AG	0.9999	ag	0.44566462	AG
NA19116	268	AG	0.9994	ag	0.629133249	AG
NA19161	269	AA	1	aa	0.005059199	AA
NA19140	270	AA	1	aa	0.213922284	AA
NA19207	271	GG	1	gg	0.32586836	GG
NA19141	272	AG	0.9998	ag	0.077718549	AG
NA18854	273	AG	0.9999	ag	0.313607723	AG
NA19098	274	AG	0.9998	ag	0.384244854	AG
NA18522	275	AG	0.9993	ag	0.134928514	AG
NA18914	276	GG	1	gg	0.245681285	GG
NA18853	277	AG	0.9991	ag	0.487146783	AG
NA18859	278	AG	0.9988	ag	0.183940682	AG
NA19100	279	AA	1	aa	0.208550604	AA
NA19093	280	AG	0.9997	ag	0.253757186	AG
NA19132	281	AG	0.9999	ag	0.059094604	AG
NA19130	282	AG	0.9993	ag	0.174889618	AG
NA19208	283	AG	0.9998	ag	0.295851861	AG
NA19127	284	AG	0.9954	ag	0.108430216	AG
NA19173	285	AA	1	aa	0.148077744	AA
NA19119	286	AG	0.9993	ag	0.218127875	AG
NA19103	287	AG	0.9999	ag	0.3469318	AG
	Discrepancies	0		0		
	NNs	1		2		
	Automated Calls	273		262		
	Manual Calls	0		11		
	Concordant Calls	253		252		254

	MACGT discrepancy					
	LDA discrepancy					
NA	LDA output were not g					
XY	Manual calls are given					
		**1433375**				
**Coriell**	**Sample**	**LDA Call**	**LDA quality score**	**MACGT Call**	**MACGT quality score**	**HapMap Call**
NA12753	1		NA	CC	0.145317953	
NA12875	2	TC	0.9968	*TC*	*5.13E-04*	TC
NA12044	3	CC	1	cc	0.643801634	CC
NA06993	4	TT	0.9993	tt	0.134409664	TT
NA12716	5	TC	0.9997	tc	0.044612542	TC
NA12760	6	TC	1	tc	0.01231217	TC
NA07348	7	TT	0.9973	tt	0.279298508	TT
NA12707	8	TT	0.9996	tt	0.127660636	TT
NA12156	9		NA	TT	0.220747304	
NA11992	10	CC	0.9997	cc	0.037218866	CC
NA12239	11	TT	0.9978	tt	0.01155868	TT
NA12878	12	TC	0.9997	tc	0.002525005	TC
NA11993	13	TT	0.9993	tt	0.038500744	TT
NA12750	14	TC	1	tc	0.025311577	TC
NA12146	15	TC	1	tc	0.129089749	TC
NA11839	16	TC	1	tc	0.011219644	TC
NA12156.dup	17	TT	0.9999	tt	0.323272053	nn
NA11829	18		NA	TC	0.283385693	
NA12154	19	TT	1	tt	0.323134179	TT
NA10856	20	TC	1	tc	0.036818628	TC
NA06985	21	NN	0.7484	*CC*	*0*	CC
NA11831	22	CC	0.7576	*CC*	*0*	CC
NA12003.dup	23	TC	1	tc	0.086064931	nn
NA10859	24	TC	1	tc	0.256973253	TC
NA07056	25	TT	1	tt	0.138198924	TT
NA07022	26	TC	1	tc	0.26737227	TC
NA12762	27		NA	TC	0.5697495	
NA12874	28	TC	0.9999	tc	0.042990973	TC
NA12003	29	TC	1	tc	0.401472453	TC
NA10835	30	TC	1	tc	0.502841365	TC
NA11882	31	TC	1	tc	0.463978602	TC
NA07034	32	TC	1	tc	0.189662561	TC
NA12056	33	TC	0.9998	tc	0.418629588	TC
NA12145	34	TT	1	tt	0.325009779	TT
NA07019	35	TT	1	tt	0.460000963	TT
NA06991	36		NA	TC	0.615549377	
NA12761	37	TC	1	tc	0.094081605	TC
NA06994	38	TC	1	tc	0.091226519	TC
NA12864	39	TC	1	tc	0.47933088	TC
NA07055	40	TT	1	tt	0.402799608	TT
NA10863	41	TC	0.9999	tc	0.339105914	TC
NA12763	42	CC	0.9985	cc	0.121585278	CC
NA10831	43	TC	1	tc	0.248216555	TC
NA11840	44	TT	1	tt	0.49463029	TT

	MACGT discrepancy					
	LDA discrepancy					
NA	LDA output were not g					
XY	Manual calls are given					
		**1433375**				
**Coriell**	**Sample**	**LDA Call**	**LDA quality score**	**MACGT Call**	**MACGT quality score**	**HapMap Call**
NA10830	45	TC	1	tc	0.391686621	TC
NA11993.dup	46	TT	1	tt	0.229037403	nn
NA12751	47	TT	0.9998	tt	0.299257728	TT
NA12814	48	TC	1	tc	0.005711886	TC
NA10857	49	TC	1	tc	0.381228934	TC
NA07357	50	TT	1	tt	0.435332444	TT
NA07000	51	CC	0.9351	cc	0.002420133	CC
NA12802	52	CC	0.9737	cc	0.007505313	CC
NA10855	53	TC	1	tc	0.234579882	TC
NA11995	54		NA	TC	0.279939827	
NA10839	55	TC	1	tc	0.362307827	TC
NA10847	56	TT	1	tt	0.096502081	TT
NA12006	57	CC	0.9415	cc	0.006903392	CC
NA12144	58	TT	1	tt	0.163509555	TT
NA11832	59	TT	1	tt	0.037353755	TT
NA12813	60	TC	1	tc	0.425083991	TC
blank	61	NN	0.75	***NN***	***0***	nn
NA12891	62	TT	1	tt	0.17371882	TT
NA12717	63		NA	TC	0.469970182	
NA10861	64	TC	1	tc	0.297110924	TC
NA12004	65	TT	1	tt	0.025256198	TT
NA07048	66	TC	0.9987	tc	0.433005051	TC
NA12801	67	CC	0.9994	cc	0.212237755	CC
NA11830	68	CC	0.9905	cc	0.023307988	CC
NA12057	69	TC	1	tc	0.30478004	TC
NA11994	70		NA	TT	0.569773942	
NA12740	71	TC	1	tc	0.560661202	TC
NA12005	72	TT	0.75	***TT***	***0***	TT
NA07345	73	TC	0.7679	***TC***	***0***	TC
NA10854	74	TT	0.75	***TT***	***0***	TT
NA12872	75	NN	0.5001	***TC***	***0***	TC
NA12865	76	NN	0.75	***CC***	***0***	CC
NA10860	77		NA	TC	0.31190989	
NA12873	78	CC	0.7661	***CC***	***0***	CC
NA12752	79	NN	0.5697	***CC***	***0***	CC
NA10851	80	TT	1	tt	0.32928261	TT
NA07029	81	NN	0.5777	***CC***	***0***	CC
NA12248	82	NN	0.7388	***TC***	***6.51E-04***	TC
NA12155	83	NN	0.7445	***TC***	***0***	TC
NA10838	84	NN	0.503	***TC***	***0***	TC
NA12812	85	NN	0.5	***TC***	***0***	TC
NA12248.dup	86	NN	0.669	***TC***	***0***	nn
NA12043	87	NN	0.5001	***NN***	***0***	TT
NA12234	88	CC	0.9999	cc	0.170989673	CC

	MACGT discrepancy					
	LDA discrepancy					
NA	LDA output were not g					
XY	Manual calls are given					
		**1433375**				
**Coriell**	**Sample**	**LDA Call**	**LDA quality score**	**MACGT Call**	**MACGT quality score**	**HapMap Call**
NA12892	89	CC	0.7506	***CC***	***0***	CC
NA10846	90	NN	0.5005	***TT***	***0***	TT
NA06993.dup	91	TT	0.7502	tt	0.001675905	nn
NA12249	92	TT	0.7501	tt	0.020770508	TT
NA11881	93	NN	0.6658	***TC***	***0***	TC
NA12815	94	NN	0.5056	***NN***	***0***	TC
NA12264	95	NN	0.5208	***TC***	***0***	TC
NA18526	96	TC	0.9876	tc	0.479178391	TC
NA18562	97	TC	0.9999	tc	0.283729206	TC
NA18545	98	CC	0.9981	cc	0.130069583	CC
NA18609	99	TC	0.9963	tc	0.146514734	TC
NA18566	100	CC	0.9952	cc	0.128762182	CC
NA18621	101	CC	0.9633	cc	0.093485164	CC
NA18577	102	TC	0.9834	tc	0.129345711	TC
NA18635	103	TT	1	tt	0.139906225	TT
NA18524	104	TT	1	tt	0.396504375	TT
NA18537	105	CC	0.9999	cc	0.167051309	CC
NA18572	106	TC	0.9828	tc	0.003842794	TC
NA18552	107	TT	0.9999	tt	0.091367296	TT
NA18563	108	CC	1	cc	0.228096053	CC
NA18594	109	CC	1	cc	0.442874903	nn
NA18624	110	TC	1	tc	0.474976432	TC
NA18592	111	TC	1	tc	0.479401402	TC
NA18529	112	TC	0.9999	tc	0.484065189	TC
NA18603	113	TC	1	tc	0.475757067	nn
NA18547	114	TC	0.9999	tc	0.150838047	TC
NA18611	115	TC	0.9999	tc	0.019890384	TC
NA18570	116	TC	1	tc	0.08693131	TC
NA18622	117	TC	0.9997	tc	0.322392687	TC
NA18579	118	TC	1	tc	0.418760981	TC
NA18636	119	CC	0.9997	cc	0.406657162	CC
NA18558	120	NN	0.7477	***CC***	***6.12E-04***	CC
NA18540	121	NN	0.747	***TC***	***0***	TC
NA18609	122	TC	0.9999	***TC***	***0***	nn
NA18555	123	NN	0.7424	***CC***	***0***	CC
NA18612	124	CC	1	cc	0.410726337	CC
NA18573	125	NN	0.7451	***TT***	***0***	TT
NA18632	126	NN	0.7474	***CC***	***0***	CC
NA18593	127	NN	0.5318	***TC***	***0***	TC
NA18532	128	NN	0.6269	***TC***	***0***	TC
NA18605	129	NN	0.5027	***TC***	***0***	TC
NA18550	130	CC	0.7507	***CC***	***0***	CC
NA18603	131	TC	0.9999	tc	0.326345096	TC
NA18571	132	TT	0.7503	***TT***	***0***	TT

	MACGT discrepancy					
	LDA discrepancy					
NA	LDA output were not g					
XY	Manual calls are given					
		**1433375**				
**Coriell**	**Sample**	**LDA Call**	**LDA quality score**	**MACGT Call**	**MACGT quality score**	**HapMap Call**
NA18623	133	NN	0.5	***TC***	***0***	TC
NA18582	134	TC	0.9999	tc	0.310264142	TC
NA18637	135	TC	0.9952	tc	0.156250597	TC
NA18561	136	NN	0.5007	***TT***	***0***	TT
NA18542	137	NN	0.5008	***TT***	***0***	TT
NA18608	138	CC	1	cc	0.277824691	CC
NA18564	139	NN	0.5567	***TC***	***0***	TC
NA18620	140	NN	0.75	***CC***	***0***	CC
NA18576	141	NN	0.5033	***TC***	***0***	TC
NA18633	142	NN	0.5	***TT***	***0***	TT
NA18594	143	NN	0.5954	***CC***	***0***	CC
NA18942	144		NA	CC	0.34964522	
NA18945	145	TC	0.9999	tc	0.368928958	TC
NA18964	146	CC	1	cc	0.065060177	CC
NA18961	147	CC	1	cc	0.003149691	CC
NA18967	148	TC	0.9998	tc	0.403455083	TC
NA18981	149		NA	CC	0.483458395	
NA18994	150		NA	CC	0.49422852	
NA18998	151	TC	0.9999	tc	0.043923758	TC
NA18940	152	CC	1	cc	0.195077045	CC
NA18949	153	TC	0.9998	tc	0.235119989	TC
NA18953	154	TC	0.9997	tc	0.06454861	TC
NA18972	155	CC	0.9995	cc	0.1914265	CC
NA18976	156	CC	1	cc	0.363009649	CC
NA18971	157	TT	1	tt	0.136944281	TT
blank	158	NN	0.5	***NN***	***0***	nn
NA19000	159	TT	0.9997	tt	0.230559115	TT
NA18951	160	TT	0.9918	tt	0.391404468	nn
NA18948	161	TC	0.953	tc	0.077039553	TC
NA18968	162	TC	1	tc	0.305263762	TC
NA18965	163	CC	0.9997	cc	0.326526563	CC
NA18978	164	TC	0.9977	tc	0.167076895	TC
NA18974	165	TT	1	tt	0.128523688	TT
NA18992	166	TT	0.9988	tt	0.019860632	TT
NA19005	167	CC	0.8822	***CC***	***7.78E-04***	CC
NA18943	168	CC	0.7917	cc	0.004565505	CC
NA18951	169	TT	1	tt	0.08302559	TT
NA18959	170	TC	1	tc	0.18790264	TC
NA18973	171	TC	1	tc	0.346787287	TC
NA18970	172	CC	0.9999	cc	0.095297515	CC
NA18987	173	TT	1	tt	0.065985104	TT
NA18995	174	TT	1	tt	0.212229121	TT
NA18999	175	CC	0.9988	cc	0.006271052	CC
NA18947	176	TC	0.9935	tc	0.12307913	TC

	MACGT discrepancy					
	LDA discrepancy					
NA	LDA output were not g					
XY	Manual calls are given					
		**1433375**				
**Coriell**	**Sample**	**LDA Call**	**LDA quality score**	**MACGT Call**	**MACGT quality score**	**HapMap Call**
NA18952	177	TC	1	tc	0.41456589	TC
NA18969	178	CC	1	cc	0.388699802	CC
NA18966	179	TT	1	tt	0.12471536	TT
NA18980	180	CC	0.9987	cc	0.071754617	CC
NA18990	181	TT	1	tt	0.024643278	TT
NA18997	182	TT	1	tt	0.039208564	TT
NA19007	183	CC	1	cc	0.284412891	CC
NA18944	184	TC	1	tc	0.019115256	TC
NA18956	185	CC	0.9969	cc	0.097430218	CC
NA18960	186	CC	0.9997	cc	0.186010815	CC
NA18975	187	CC	0.751	***CC***	***0***	CC
NA18995	188	TT	1	tt	0.5888282	nn
NA18991	189	CC	0.9991	cc	0.213067079	CC
NA18996	190	TC	0.9685	tc	0.00396348	nn
NA19003	191	TC	1	tc	0.405356298	TC
NA18502	192	NN	0.4932	***NN***	***0***	CC
NA19153	193	TC	0.9189	tc	0.001458795	TC
NA18857	194	NN	0.5	***NN***	***0***	TC
NA19223	195	NN	0.578	***CC***	***0***	CC
NA19201	196	NN	0.4386	***NN***	***0***	CC
NA18504	197	NN	0.4413	***NN***	***0***	CC
NA18870	198	NN	0.5731	***NN***	***0***	CC
NA18863	199	NN	0.5058	***TC***	***0***	TC
NA19145	200	NN	0.4755	***NN***	***0***	CC
NA19137	201	NN	0.5001	***NN***	***0***	TC
NA19238	202	CC	1	***CC***	***0***	nn
NA18500	203	NN	0.5183	***CC***	***0***	CC
NA19144	204	NN	0.746	***NN***	***0***	CC
NA19203	205	CC	1	cc	0.338384368	CC
NA19200	206	NN	0.7099	***CC***	***0***	CC
NA18855	207	NN	0.5023	***NN***	***0***	TC
NA18505	208	CC	1	cc	0.103763624	CC
NA19202	209	NN	0.6829	***NN***	***0***	CC
NA18501	210	NN	0.4992	***NN***	***0***	CC
NA18861	211	NN	0.75	***NN***	***0***	TC
NA19193	212	NN	0.6326	***CC***	***0***	CC
NA19143	213	NN	0.5014	***NN***	***0***	TC
NA18517	214	CC	1	cc	0.271974901	CC
NA18862	215	NN	0.5	***NN***	***0***	TC
NA18856	216	CC	0.9992	cc	0.167909285	nn
NA19239	217	CC	0.9998	cc	0.060677174	CC
NA19240	218	CC	0.9999	cc	0.065272786	CC
NA18856	219	CC	0.9995	cc	0.013916807	CC
NA18503	220	CC	0.967	cc	0.022701886	CC

	MACGT discrepancy					
	LDA discrepancy					
NA	LDA output were not g					
XY	Manual calls are given					
		**1433375**				
**Coriell**	**Sample**	**LDA Call**	**LDA quality score**	**MACGT Call**	**MACGT quality score**	**HapMap Call**
NA18871	221	CC	0.9889	cc	0.068615406	CC
NA19221	222	CC	0.9998	cc	0.104348314	CC
NA19209	223	CC	0.9567	cc	0.02156202	CC
NA19152	224	CC	0.9984	cc	0.070955869	CC
NA18515	225	TC	1	tc	0.154230964	TC
NA19238	226	CC	0.9998	cc	0.067330163	CC
NA19154	227	TC	1	tc	0.522639118	TC
NA19210	228	CC	0.9876	cc	0.016484428	CC
NA19211	229	CC	1	cc	0.176363438	CC
NA18862	230	TC	0.9969	tc	0.288527964	nn
NA18872	231	CC	0.9782	cc	0.050236536	CC
NA19139	232	CC	0.9997	cc	0.531360571	CC
NA19222	233	CC	0.9991	cc	0.221918014	CC
NA19194	234	CC	0.9997	cc	0.386817619	CC
NA19138	235	CC	1	cc	0.03958713	CC
NA19204	236	CC	1	cc	0.38775616	CC
NA18516	237	TC	0.9996	tc	0.31031027	TC
NA19205	238	CC	0.9535	cc	0.024409023	CC
NA19192	239	CC	1	cc	0.124529811	CC
NA18912	240	CC	0.9542	cc	0.026937068	nn
NA18508	241	TT	1	tt	0.28030404	TT
NA19142	242	CC	0.7623	***cc***	***9.22E-04***	CC
NA18852	243	CC	1	cc	0.509308188	CC
NA18507	244	CC	1	cc	0.183740286	CC
blank	245	NN	0.5093	***NN***	***0***	nn
NA19101	246	CC	0.9997	cc	0.530232824	CC
NA19172	247	CC	0.9992	cc	0.160956516	CC
NA19160	248	CC	0.9982	cc	0.027065997	CC
NA19129	249	CC	0.9997	cc	0.301643401	CC
NA18913	250	CC	0.9998	cc	0.357747139	CC
NA19120	251	CC	0.9974	cc	0.187072873	CC
NA19159	252	CC	1	***CC***	***0***	CC
NA18523	253	TC	0.9999	tc	0.45509258	TC
NA19102	254	NN	0.7472	***CC***	***0***	CC
NA19092	255	CC	0.9998	cc	0.269874724	CC
NA18521	256	CC	0.9912	cc	0.090342428	CC
NA18912	257	CC	0.9136	cc	0.01684585	CC
NA19206	258	CC	1	cc	0.351291948	CC
NA19128	259	CC	0.9953	cc	0.036501362	CC
NA18859	260	CC	0.9999	cc	0.241566785	nn
NA19094	261	CC	0.9719	cc	0.209335667	CC
NA19171	262	CC	1	cc	0.483001785	CC
NA18860	263	CC	0.9997	cc	0.067924423	CC
NA18858	264	CC	1	cc	0.001953274	CC

	MACGT discrepancy					
	LDA discrepancy					
NA	LDA output were not g					
XY	Manual calls are given					
		1433375				
**Coriell**	**Sample**	**LDA Call**	**LDA quality score**	**MACGT Call**	**MACGT quality score**	**HapMap Call**
NA19099	265	CC	1	cc	0.274796398	CC
NA18506	266	TC	1	tc	0.344552666	TC
NA19131	267	CC	0.8214	cc	0.013189028	CC
NA19116	268	CC	0.7553	cc	0.009027739	CC
NA19161	269	CC	1	***CC***	***0***	CC
NA19140	270	CC	0.9997	cc	0.144822924	CC
NA19207	271	TC	0.9958	tc	0.111172669	TC
NA19141	272	CC	1	cc	0.027933713	CC
NA18854	273	TC	1	tc	0.252809871	TC
NA19098	274	CC	1	cc	0.490789191	CC
NA18522	275	CC	1	cc	0.026000659	CC
NA18914	276	CC	0.9996	cc	0.175442091	CC
NA18853	277	TC	1	tc	0.001744439	TC
NA18859	278	CC	0.8772	cc	0.003908542	CC
NA19100	279	CC	0.8732	***CC***	***0***	CC
NA19093	280	CC	1	cc	0.20639133	CC
NA19132	281	CC	0.9996	cc	0.002579115	CC
NA19130	282	CC	1	cc	0.171194811	CC
NA19208	283	TC	1	tc	0.152133519	TC
NA19127	284	TC	0.9999	tc	0.130750397	TC
NA19173	285	CC	0.9978	***CC***	***0***	CC
NA19119	286	CC	0.9958	cc	0.250334897	CC
NA19103	287	CC	0.9999	cc	0.032807888	CC
	Discrepancies	0		0		
	NNs	52		17		
	Automated Calls	275		203		
	Manual Calls	0		72		
	Concordant Calls	206		240		256

	MACGT discrepancy					
	LDA discrepancy					
NA	LDA output were not g					
XY	Manual calls are given					
		1486048				
**Coriell**	**Sample**	**LDA Call**	**LDA quality score**	**MACGT Call**	**MACGT quality score**	**HapMap Call**
NA12753	1		NA	GA	0.499258852	
NA12875	2	GG	0.9882	gg	0.157058017	GG
NA12044	3	GA	0.9874	ga	0.481622497	GA
NA06993	4	GG	0.8857	gg	0.18956498	GG
NA12716	5	GG	0.812	gg	0.120061093	GG
NA12760	6	AA	0.8655	aa	0.368516501	AA
NA07348	7	GG	0.9994	gg	0.355590865	GG
NA12707	8	GA	0.9611	ga	0.107196455	GA
NA12156	9		NA	GA	0.183968746	
NA11992	10	GG	0.9792	gg	0.096876562	GG
NA12239	11	GG	0.908	gg	0.244990753	GG
NA12878	12	GA	0.9865	ga	0.313780607	GA
NA11993	13	GA	0.9969	ga	0.004328512	GA
NA12750	14	GA	0.9815	ga	0.061169068	GA
NA12146	15	GA	0.9943	ga	0.06318907	GA
NA11839	16	GG	0.997	gg	0.15813368	GG
NA12156.dup	17	GA	0.9467	ga	0.223298222	nn
NA11829	18		NA	GG	0.300934979	
NA12154	19	GA	0.8709	ga	0.170654383	GA
NA10856	20	GG	1	gg	0.217688902	GG
NA06985	21	GA	0.8915	ga	0.210233894	GA
NA11831	22	GA	0.9996	ga	0.001214672	GA
NA12003.dup	23	GA	0.9969	ga	0.014593302	nn
NA10859	24	GG	0.9314	gg	0.240446294	GG
NA07056	25	GA	0.8922	ga	0.102293468	GA
NA07022	26	GA	0.9968	ga	0.065280751	GA
NA12762	27		NA	GA	0.330964669	
NA12874	28	GA	0.9586	ga	0.017024179	GA
NA12003	29	GA	0.9805	ga	0.320943111	GA
NA10835	30	GA	0.9392	ga	0.300597891	GA
NA11882	31	GG	1	gg	0.279273714	GG
NA07034	32	GA	0.9504	ga	0.191582242	GA
NA12056	33	GA	0.9606	ga	0.394026548	GA
NA12145	34	GG	0.8024	gg	0.146844682	GG
NA07019	35	GA	0.9914	ga	0.26504655	GA
NA06991	36		NA	GA	0.43244604	
NA12761	37	AA	0.9528	aa	0.457433007	AA
NA06994	38	GG	0.8056	gg	0.18280946	GG
NA12864	39	GG	0.9257	gg	0.372662459	GG
NA07055	40	GA	0.9824	ga	0.285887752	GA
NA10863	41	GG	0.9218	gg	0.334077842	GG
NA12763	42	GA	0.9958	ga	0.245587707	GA
NA10831	43	GG	0.9714	gg	0.356056605	GG
NA11840	44	GG	0.9814	gg	0.211795454	GG

	MACGT discrepancy					
	LDA discrepancy					
NA	LDA output were not g					
XY	Manual calls are given					
		**1486048**				
**Coriell**	**Sample**	**LDA Call**	**LDA quality score**	**MACGT Call**	**MACGT quality score**	**HapMap Call**
NA10830	45	GA	0.9856	ga	0.046472433	GA
NA11993.dup	46	GA	0.9853	ga	0.214885111	nn
NA12751	47	GG	0.9511	gg	0.379315379	GG
NA12814	48	GA	0.9892	ga	0.271073777	GA
NA10857	49	AA	0.9672	aa	0.332221485	AA
NA07357	50	GG	0.9979	gg	0.448759027	GG
NA07000	51	GG	0.9619	gg	0.251637231	GG
NA12802	52	GG	0.7896	gg	0.232917615	GG
NA10855	53	GG	0.7562	gg	0.082505313	GG
NA11995	54		NA	GG	0.137566308	
NA10839	55	GG	0.8066	gg	0.054548875	GG
NA10847	56	GA	0.9943	ga	0.148187505	GA
NA12006	57	GG	0.9412	gg	0.367496234	GG
NA12144	58	GG	0.9885	gg	0.364151391	GG
NA11832	59	GA	0.9926	ga	0.369316808	GA
NA12813	60	GA	0.9928	ga	0.583850833	GA
blank	61	NN	0.5416	***NN***	***0***	nn
NA12891	62	GG	0.9952	gg	0.370051356	GG
NA12717	63		NA	AA	0.354196669	
NA10861	64	GG	0.9296	gg	0.025455166	GG
NA12004	65	GG	0.9942	gg	0.08666774	GG
NA07048	66	GG	0.9962	gg	0.022298922	GG
NA12801	67	GG	0.9984	gg	0.132702863	GG
NA11830	68	GA	0.9683	ga	0.471547859	GA
NA12057	69	GG	0.9858	gg	0.215726692	GG
NA11994	70		NA	GG	0.50113642	
NA12740	71	GG	0.93	gg	0.277004748	GG
NA12005	72	GA	0.9615	ga	0.030728897	GA
NA07345	73	GG	0.9855	gg	0.173564691	GG
NA10854	74	GG	0.995	gg	0.423424372	GG
NA12872	75	GA	0.9336	ga	0.355639797	GA
NA12865	76	GG	0.9719	gg	0.401513949	GG
NA10860	77		NA	GA	0.139996424	
NA12873	78	GA	0.7849	ga	0.410162362	GA
NA12752	79	AA	0.9992	aa	0.103890468	AA
NA10851	80	GA	0.9611	ga	0.566392535	GA
NA07029	81	GG	0.9993	gg	0.323533327	GG
NA12248	82	GA	0.764	ga	0.196250614	GA
NA12155	83	GG	0.9973	gg	0.367160271	GG
NA10838	84	GA	0.7618	ga	0.267781331	GA
NA12812	85	GA	0.7802	ga	0.18193934	GA
NA12248.dup	86	GA	0.8604	ga	0.21556155	nn
NA12043	87	AA	0.9969	aa	0.350118732	AA
NA12234	88	GG	0.9998	gg	0.204717519	GG

	MACGT discrepancy					
	LDA discrepancy					
NA	LDA output were not g					
XY	Manual calls are given					
		**1486048**				
**Coriell**	**Sample**	**LDA Call**	**LDA quality score**	**MACGT Call**	**MACGT quality score**	**HapMap Call**
NA12892	89	AA	0.9977	aa	0.174645499	AA
NA10846	90	GG	0.9999	gg	0.317577785	GG
NA06993.dup	91	GG	0.9978	gg	0.199441542	nn
NA12249	92	GA	0.8017	ga	0.066928336	GA
NA11881	93	GA	0.8892	ga	0.095675856	GA
NA12815	94	GG	0.9996	gg	0.309731994	GG
NA12264	95	GA	0.8113	ga	0.597387595	GA
NA18526	96	AA	0.9966	aa	0.207590175	AA
NA18562	97	AA	0.974	aa	0.534623568	AA
NA18545	98	GA	0.9836	ga	0.116630492	GA
NA18609	99	GG	0.961	gg	0.128345548	GG
NA18566	100	GA	0.9876	***GA***	***0***	GA
NA18621	101	GA	0.9823	ga	0.247106119	GA
NA18577	102	GG	0.994	gg	0.095276449	GG
NA18635	103	AA	0.8519	aa	0.335028166	AA
NA18524	104	GA	0.9737	ga	0.571140191	GA
NA18537	105	GA	0.9839	ga	0.182601654	GA
NA18572	106	AA	0.924	aa	0.44541632	AA
NA18552	107	AA	0.8251	aa	0.253710434	AA
NA18563	108	GA	0.9807	ga	0.130954012	GA
NA18594	109	GA	0.9799	ga	0.172895605	nn
NA18624	110	GA	0.8876	ga	0.257665202	GA
NA18592	111	GA	0.9869	ga	0.598712971	GA
NA18529	112	GA	0.9865	ga	0.510446392	GA
NA18603	113	GA	0.9803	ga	0.597715441	nn
NA18547	114	GA	0.9943	ga	0.396255374	GA
NA18611	115	GA	0.987	ga	0.249421024	GA
NA18570	116	GA	0.9801	ga	0.508162343	GA
NA18622	117	AA	0.999	aa	0.290041659	AA
NA18579	118	GG	0.9949	gg	0.245809603	GG
NA18636	119	GG	0.9965	gg	0.471776565	GG
NA18558	120	GG	0.9989	gg	0.407033104	GG
NA18540	121	GG	0.9994	gg	0.349468745	GG
NA18609	122	GG	0.9976	gg	0.575798876	nn
NA18555	123	AA	0.9987	aa	0.161878648	AA
NA18612	124	GA	0.826	ga	0.249430187	GA
NA18573	125	AA	0.9986	aa	0.224055904	AA
NA18632	126	AA	0.9992	aa	0.211886353	AA
NA18593	127	GA	0.8506	ga	0.454244035	GA
NA18532	128	GA	0.9565	ga	0.580448604	GA
NA18605	129	GA	0.8495	ga	0.325277543	GA
NA18550	130	AA	0.9992	aa	0.224351084	AA
NA18603	131	GA	0.9311	ga	0.290480173	GA
NA18571	132	GA	0.7865	ga	0.408007264	GA

	MACGT discrepancy					
	LDA discrepancy					
NA	LDA output were not g					
XY	Manual calls are given					
		**1486048**				
**Coriell**	**Sample**	**LDA Call**	**LDA quality score**	**MACGT Call**	**MACGT quality score**	**HapMap Call**
NA18623	133	GA	0.7917	ga	0.307274641	GA
NA18582	134	GA	0.9206	ga	0.205749319	GA
NA18637	135	GG	0.9988	gg	0.190516925	GG
NA18561	136	GA	0.8872	ga	0.292632731	GA
NA18542	137	GG	0.9998	gg	0.409022295	GG
NA18608	138	GG	0.9826	gg	0.245343032	GG
NA18564	139	GG	0.9998	gg	0.474445534	GG
NA18620	140	GA	0.8257	ga	0.304690874	GA
NA18576	141	GA	0.8249	ga	0.479421106	GA
NA18633	142	GA	0.9184	ga	0.420144504	GA
NA18594	143	GA	0.8228	ga	0.420086785	GA
NA18942	144	GA	0.9833	ga	0.499627701	GA
NA18945	145	GG	0.9893	gg	0.353526275	GG
NA18964	146	GA	0.9748	ga	0.434963488	GA
NA18961	147	GA	0.9576	ga	0.443608984	GA
NA18967	148	GA	0.9661	ga	0.51194841	GA
NA18981	149	GA	0.9514	ga	0.398919988	GA
NA18994	150	GG	0.9995	gg	0.228868512	GG
NA18998	151		NA	AA	0.442172777	
NA18940	152		NA	AA	0.435916471	
NA18949	153	GA	0.9676	ga	0.156149061	GA
NA18953	154	GG	0.9996	***GG***	***0***	GG
NA18972	155	GA	0.8957	ga	0.07263263	GA
NA18976	156	GA	0.9245	ga	0.286312138	GA
NA18971	157	GG	0.9989	gg	0.001752274	GG
blank	158	NN	0.5002	***NN***	***0***	nn
NA19000	159	GG	0.9961	gg	0.608296968	GG
NA18951	160	GA	0.8437	ga	0.110868571	nn
NA18948	161	GG	0.9989	gg	0.421719705	GG
NA18968	162	NN	0.6869	ga	0.21292827	GA
NA18965	163	AA	0.9814	aa	0.142511978	AA
NA18978	164	GA	0.8472	ga	0.149297679	GA
NA18974	165	NN	0.7371	ga	0.125589039	GA
NA18992	166	GG	0.9981	gg	0.195225013	GG
NA19005	167	GG	0.828	gg	0.420407476	GG
NA18943	168	GA	0.9906	ga	0.236431446	GA
NA18951	169	GA	0.9951	ga	0.251020237	GA
NA18959	170	GG	0.9261	gg	0.30782979	GG
NA18973	171	GA	0.9647	ga	0.432200888	GA
NA18970	172	GA	0.9765	ga	0.482353497	GA
NA18987	173	GA	0.9722	ga	0.339695349	GA
NA18995	174	GA	0.9691	ga	0.625626577	GA
NA18999	175	AA	0.9964	aa	0.14611415	AA
NA18947	176	GA	0.8506	ga	0.066651242	GA

	MACGT discrepancy					
	LDA discrepancy					
NA	LDA output were not g					
XY	Manual calls are given					
		**1486048**				
**Coriell**	**Sample**	**LDA Call**	**LDA quality score**	**MACGT Call**	**MACGT quality score**	**HapMap Call**
NA18952	177	GA	0.9615	ga	0.442946661	GA
NA18969	178	GA	0.9565	ga	0.213415815	GA
NA18966	179	GA	0.9335	ga	0.327959792	GA
NA18980	180	GG	0.9957	gg	0.136004104	GG
NA18990	181	AA	0.9935	aa	0.174437423	AA
NA18997	182	GA	0.9399	ga	0.353089124	GA
NA19007	183	GA	0.9747	ga	0.223587396	GA
NA18944	184	GA	0.9669	ga	0.005349471	GA
NA18956	185	GG	0.8856	gg	0.218146448	GG
NA18960	186	GA	0.9759	ga	0.369990797	GA
NA18975	187	AA	0.9854	aa	0.202319741	AA
NA18995	188	GA	0.9816	ga	0.522106397	nn
NA18991	189	GA	0.9776	ga	0.486328582	GA
NA18996	190	GA	0.9848	ga	0.435582295	nn
NA19003	191	GG	0.9525	gg	0.435743829	GG
NA18502	192	AA	0.9701	aa	0.344537251	AA
NA19153	193	AA	0.9805	aa	0.383896429	AA
NA18857	194	AA	0.9953	aa	0.421291795	AA
NA19223	195	AA	0.9875	aa	0.267517183	AA
NA19201	196	GA	0.9363	ga	0.505735085	GA
NA18504	197	AA	0.968	aa	0.298224284	AA
NA18870	198	AA	0.9924	aa	0.427391589	AA
NA18863	199	AA	0.9953	aa	0.388914815	AA
NA19145	200	AA	0.9991	aa	0.143834107	AA
NA19137	201	AA	0.9962	aa	0.54220418	AA
NA19238	202	GA	0.9259	ga	0.050888923	nn
NA18500	203	AA	0.996	aa	0.332519718	AA
NA19144	204	AA	0.9969	aa	0.084336073	AA
NA19203	205	AA	0.9935	aa	0.337197265	AA
NA19200	206	GA	0.9353	ga	0.40871167	GA
NA18855	207	AA	0.9991	aa	0.238647356	AA
NA18505	208	AA	0.9908	aa	0.317138435	AA
NA19202	209	GA	0.8478	ga	0.197292377	GA
NA18501	210	AA	0.9938	aa	0.039337625	AA
NA18861	211	AA	0.9966	aa	0.195616258	AA
NA19193	212	AA	0.9919	aa	0.120455187	AA
NA19143	213	AA	0.9989	aa	0.0913203	AA
NA18517	214	AA	0.9973	aa	0.15313873	AA
NA18862	215	AA	0.9982	aa	0.207316095	AA
NA18856	216	AA	0.9936	aa	0.266522328	nn
NA19239	217	AA	0.9924	aa	0.073487579	AA
NA19240	218	GA	0.8104	ga	0.112518567	GA
NA18856	219	AA	0.9894	aa	0.363443996	AA
NA18503	220	AA	0.9981	aa	0.120391851	AA

	MACGT discrepancy					
	LDA discrepancy					
NA	LDA output were not g					
XY	Manual calls are given					
				**1486048**		
**Coriell**	**Sample**	**LDA Call**	**LDA quality score**	**MACGT Call**	**MACGT quality score**	**HapMap Call**
NA18871	221	AA	0.9839	aa	0.227098989	AA
NA19221	222	AA	0.9723	aa	0.313958276	AA
NA19209	223	AA	0.9608	aa	0.071734678	AA
NA19152	224	AA	0.9118	aa	0.233769857	AA
NA18515	225	AA	0.9857	aa	0.36394031	AA
NA19238	226	GA	0.9695	ga	0.237052892	GA
NA19154	227	AA	0.9836	aa	0.1716327	AA
NA19210	228	AA	0.9863	aa	0.274726665	AA
NA19211	229	AA	0.9992	aa	0.241606876	AA
NA18862	230	AA	0.9983	aa	0.104008893	nn
NA18872	231	AA	0.9987	aa	0.292387156	AA
NA19139	232	AA	0.9936	aa	0.188088009	AA
NA19222	233	AA	0.987	aa	0.215220588	AA
NA19194	234	AA	0.989	aa	0.109150199	AA
NA19138	235	AA	0.9868	aa	0.179527096	AA
NA19204	236	AA	0.7969	aa	0.261766056	AA
NA18516	237	AA	0.9797	***AA***	***0***	AA
NA19205	238	AA	0.9958	aa	0.110569516	AA
NA19192	239	GA	0.9665	ga	0.158280175	GA
NA18912	240	AA	0.9957	aa	0.301047745	nn
NA18508	241	AA	0.9891	aa	0.060775987	AA
NA19142	242	AA	0.9902	aa	0.034416443	AA
NA18852	243	AA	0.9958	aa	0.413819018	AA
NA18507	244	GA	0.966	ga	0.299059458	GA
blank	245	NN	0.5521	***NN***	***0***	nn
NA19101	246	AA	0.9957	aa	0.274969662	AA
NA19172	247	AA	0.9717	aa	0.319148362	AA
NA19160	248	AA	0.9937	aa	0.139557159	AA
NA19129	249	GA	0.8981	ga	0.102596171	GA
NA18913	250	GA	0.9475	ga	0.32399795	GA
NA19120	251	AA	0.9721	aa	0.121308925	AA
NA19159	252	AA	0.9797	aa	0.148596853	AA
NA18523	253	AA	0.9969	aa	0.193203889	AA
NA19102	254	AA	0.9624	aa	0.399961929	AA
NA19092	255	AA	0.9856	aa	0.139284156	AA
NA18521	256	AA	0.91	aa	0.107043148	AA
NA18912	257	AA	0.8638	aa	0.103502835	AA
NA19206	258	AA	0.9909	aa	0.136796622	AA
NA19128	259	GA	0.993	ga	0.368337744	GA
NA18859	260	AA	0.9874	aa	0.461534193	nn
NA19094	261	AA	0.972	aa	0.287948156	AA
NA19171	262	AA	0.9734	aa	0.426868097	AA
NA18860	263	AA	0.766	aa	0.225511987	AA
NA18858	264	AA	0.912	aa	0.277908956	AA

	MACGT discrepancy					
	LDA discrepancy					
NA	LDA output were not g					
XY	Manual calls are given					
		1486048				
**Coriell**	**Sample**	**LDA Call**	**LDA quality score**	**MACGT Call**	**MACGT quality score**	**HapMap Call**
NA19099	265	AA	0.8603	aa	0.127313729	AA
NA18506	266	GA	0.9909	***GA***	***0***	GA
NA19131	267	AA	0.7757	aa	0.170059425	AA
NA19116	268	AA	0.7596	aa	0.088465448	AA
NA19161	269	AA	0.8172	aa	0.207421153	AA
NA19140	270	AA	0.9213	aa	0.17094549	AA
NA19207	271	AA	0.9967	aa	0.209879862	AA
NA19141	272	AA	0.8214	aa	0.317570101	AA
NA18854	273	AA	0.8391	aa	0.238080082	AA
NA19098	274	AA	0.8054	aa	0.364254268	AA
NA18522	275	AA	0.8397	aa	0.056191772	AA
NA18914	276	AA	0.9911	aa	0.01558402	AA
NA18853	277	AA	0.9971	aa	0.129882058	AA
NA18859	278	AA	0.7701	aa	0.033393362	AA
NA19100	279	AA	0.7869	aa	0.07753796	AA
NA19093	280	AA	0.888	aa	0.28476796	AA
NA19132	281	GA	0.9861	ga	0.10022719	GA
NA19130	282	GA	0.9686	***GA***	***8.62E-04***	GA
NA19208	283	AA	0.7602	aa	0.157733019	AA
NA19127	284	AA	0.9977	aa	0.29590111	AA
NA19173	285	AA	0.9934	aa	0.227272415	AA
NA19119	286	AA	0.8343	aa	0.114878307	AA
NA19103	287	AA	0.7571	aa	0.198422031	AA
	Discrepancies	0		0		
	NNs	3		1		
	Automated Calls	276		268		
	Manual Calls	0		8		
	Concordant Calls	255		257		257

	MACGT discrepancy					
	LDA discrepancy					
NA	LDA output were not g					
XY	Manual calls are given					
		1560434				
**Coriell**	**Sample**	**LDA Call**	**LDA quality score**	**MACGT Call**	**MACGT quality score**	**HapMap Call**
NA12753	1		NA	CT	0.370523161	
NA12875	2	TT	1	tt	0.139921365	TT
NA12044	3	TT	1	tt	0.492962913	TT
NA06993	4	TT	1	tt	0.181294346	TT
NA12716	5	CT	0.9704	ct	0.330013108	CT
NA12760	6	TT	1	tt	0.318723952	TT
NA07348	7	TT	1	tt	0.185392533	TT
NA12707	8	CT	0.9887	ct	0.256156328	CT
NA12156	9		NA	CT	0.231636164	
NA11992	10	CT	0.9835	ct	0.163020827	CT
NA12239	11	CT	0.941	ct	0.258612898	CT
NA12878	12	TT	1	tt	0.049506984	TT
NA11993	13	TT	0.9995	tt	0.001147879	TT
NA12750	14	CT	0.9991	ct	0.020501171	CT
NA12146	15	TT	0.998	tt	0.01354509	TT
NA11839	16	TT	1	tt	0.002660812	TT
NA12156.dup	17	CT	0.9942	ct	0.264563504	nn
NA11829	18		NA	TT	0.324486658	
NA12154	19	TT	1	tt	0.065587899	TT
NA10856	20	CT	0.9989	ct	0.100812935	CT
NA06985	21	TT	0.75	tt	0.200940567	TT
NA11831	22	CT	0.9977	ct	0.092935088	CT
NA12003.dup	23	TT	0.9993	tt	0.0213375	nn
NA10859	24	TT	1	tt	0.108346133	TT
NA07056	25	CT	0.991	ct	0.088284467	CT
NA07022	26		NA	CC	0.324971583	
NA12762	27		NA	CT	0.31989462	
NA12874	28	TT	1	tt	0.104385313	TT
NA12003	29	TT	1	tt	0.204240816	TT
NA10835	30	CT	0.9912	ct	0.48505888	CT
NA11882	31	TT	1	tt	0.264141066	TT
NA07034	32	TT	1	tt	0.174990266	TT
NA12056	33	CT	0.9892	ct	0.385820957	CT
NA12145	34	CT	0.9434	ct	0.16720496	CT
NA07019	35	CC	0.9764	cc	0.442180494	CC
NA06991	36		NA	TT	0.401285245	
NA12761	37	CT	0.9886	ct	0.040847449	CT
NA06994	38	TT	1	tt	0.07973381	TT
NA12864	39	CT	0.9739	ct	0.446746021	CT
NA07055	40	CT	0.9855	ct	0.252374677	CT
NA10863	41	CT	0.9828	ct	0.327155425	CT
NA12763	42	CT	0.9787	ct	0.512174438	CT
NA10831	43	CT	0.99	ct	0.302456094	CT
NA11840	44	CT	0.8725	ct	0.00239591	CT

	MACGT discrepancy					
	LDA discrepancy					
NA	LDA output were not g					
XY	Manual calls are given					
		1560434				
**Coriell**	**Sample**	**LDA Call**	**LDA quality score**	**MACGT Call**	**MACGT quality score**	**HapMap Call**
NA10830	45	CT	0.9751	ct	0.421104614	CT
NA11993.dup	46	TT	0.9999	tt	0.035586048	nn
NA12751	47	CC	0.9952	cc	0.241108484	CC
NA12814	48	TT	1	tt	0.051074301	TT
NA10857	49	CT	0.9794	ct	0.29644807	CT
NA07357	50	TT	1	tt	0.167948989	TT
NA07000	51	NN	0.7499	tt	0.042979689	TT
NA12802	52	TT	1	tt	0.353347007	TT
NA10855	53	CT	0.9869	ct	0.093250432	CT
NA11995	54		NA	CT	0.193595856	
NA10839	55	TT	0.7506	tt	0.057141091	TT
NA10847	56	TT	0.9901	tt	0.180172807	TT
NA12006	57	TT	0.9995	tt	0.095743676	TT
NA12144	58	CT	0.9768	ct	0.351932005	CT
NA11832	59	CT	0.9659	ct	0.203134994	CT
NA12813	60	CT	0.9618	ct	0.32531606	CT
blank	61	TT	0.7501	tt	0.001651632	nn
NA12891	62	TT	0.7546	tt	0.138494478	TT
NA12717	63		NA	TT	0.203547037	
NA10861	64	CT	0.9523	ct	0.029997864	CT
NA12004	65	NN	0.7499	tt	0.020229233	TT
NA07048	66	CT	0.9756	ct	0.47862443	CT
NA12801	67	CC	0.9968	cc	0.055759282	CC
NA11830	68		NA	CC	0.433777991	
NA12057	69	CT	0.9599	ct	0.27684694	CT
NA11994	70		NA	TT	0.504936645	
NA12740	71	CT	0.986	ct	0.40082691	CT
NA12005	72	TT	1	tt	0.001344766	TT
NA07345	73	CT	0.9964	ct	0.024938789	CT
NA10854	74	CT	0.8345	ct	0.049533801	CT
NA12872	75		NA	CC	0.485573415	
NA12865	76	TT	1	tt	0.467479364	TT
NA10860	77		NA	CT	0.305786874	
NA12873	78	CT	0.9892	ct	0.416902811	CT
NA12752	79	TT	1	tt	0.264397922	TT
NA10851	80	CT	0.9902	ct	0.484349191	CT
NA07029	81	TT	1	tt	0.482642766	TT
NA12248	82	TT	1	tt	0.191153031	TT
NA12155	83	TT	1	tt	0.043241164	TT
NA10838	84	TT	0.7501	tt	0.254014612	TT
NA12812	85	CT	0.9451	ct	0.418885584	CT
NA12248.dup	86	TT	0.7517	tt	0.441813595	nn
NA12043	87	CT	0.9923	ct	0.268514591	CT
NA12234	88	CT	0.9645	ct	0.09071295	CT

	MACGT discrepancy					
	LDA discrepancy					
NA	LDA output were not g					
XY	Manual calls are given					
		**1560434**				
**Coriell**	**Sample**	**LDA Call**	**LDA quality score**	**MACGT Call**	**MACGT quality score**	**HapMap Call**
NA12892	89	TT	1	tt	0.224863497	TT
NA10846	90	CT	0.9847	ct	0.396753339	CT
NA06993.dup	91	TT	1	tt	0.166920822	nn
NA12249	92	CT	0.9946	ct	0.043433063	CT
NA11881	93	CT	0.912	ct	0.053157319	CT
NA12815	94	TT	0.9999	tt	0.063937309	TT
NA12264	95	TT	1	tt	0.108652778	TT
NA18526	96	TT	1	tt	0.224403852	TT
NA18562	97	CT	0.9794	ct	0.021184341	CT
NA18545	98	TT	1	tt	0.165198055	TT
NA18609	99	TT	1	tt	0.120882578	TT
NA18566	100	CT	0.9779	ct	0.151830786	CT
NA18621	101	TT	1	tt	0.161218264	TT
NA18577	102	TT	1	***TT***	***0***	TT
NA18635	103	TT	1	tt	0.052159618	TT
NA18524	104	TT	1	tt	0.265091996	TT
NA18537	105	TT	1	tt	0.108636353	TT
NA18572	106	TT	1	tt	0.062391765	TT
NA18552	107	CT	0.9881	ct	0.079549803	CT
NA18563	108	TT	1	tt	0.04972335	TT
NA18594	109	TT	1	tt	0.071249312	nn
NA18624	110	TT	1	tt	0.370475758	TT
NA18592	111	TT	0.956	tt	0.252759033	TT
NA18529	112	TT	1	tt	0.281275702	TT
NA18603	113	TT	0.9995	tt	0.314379347	nn
NA18547	114	TT	1	tt	0.139569602	TT
NA18611	115	TT	0.987	tt	0.020183801	TT
NA18570	116	TT	0.9996	tt	0.109890096	TT
NA18622	117	TT	1	tt	0.291140495	TT
NA18579	118	CT	0.9417	ct	0.154808377	CT
NA18636	119	CT	0.967	ct	0.449335395	CT
NA18558	120	TT	1	tt	0.212180414	TT
NA18540	121	CT	0.9636	ct	0.363625311	CT
NA18609	122	TT	1	tt	0.318542758	nn
NA18555	123	CT	0.9865	ct	0.155346118	CT
NA18612	124	TT	1	tt	0.24283581	TT
NA18573	125	TT	1	tt	0.25181724	TT
NA18632	126	TT	1	tt	0.102267998	TT
NA18593	127	CT	0.9742	ct	0.469273572	CT
NA18532	128	CT	0.9851	ct	0.579853355	CT
NA18605	129	TT	1	tt	0.128673352	TT
NA18550	130	TT	1	tt	0.327338122	TT
NA18603	131	TT	1	tt	0.462821592	TT
NA18571	132	TT	1	tt	0.328614994	TT

	MACGT discrepancy					
	LDA discrepancy					
NA	LDA output were not g					
XY	Manual calls are given					
		1560434				
**Coriell**	**Sample**	**LDA Call**	**LDA quality score**	**MACGT Call**	**MACGT quality score**	**HapMap Call**
NA18623	133	TT	1	tt	0.47787965	TT
NA18582	134	CT	0.9636	ct	0.529342832	CT
NA18637	135	TT	1	tt	0.194937417	TT
NA18561	136	CT	0.975	ct	0.467191394	CT
NA18542	137	CT	0.9621	ct	0.51227344	CT
NA18608	138	TT	1	tt	0.277790541	TT
NA18564	139	TT	1	tt	0.25545641	TT
NA18620	140	CT	0.9791	ct	0.603479475	CT
NA18576	141	CT	0.982	ct	0.669181387	CT
NA18633	142	TT	1	tt	0.281641808	TT
NA18594	143	TT	1	tt	0.295390591	TT
NA18942	144	TT	1	tt	0.022247442	TT
NA18945	145	CT	0.9761	ct	0.310542212	CT
NA18964	146	CT	0.9717	ct	0.304686794	CT
NA18961	147		NA	CC	0.415549426	
NA18967	148	TT	1	tt	0.221839874	TT
NA18981	149	TT	1	tt	0.337306572	TT
NA18994	150	TT	1	tt	0.330476456	TT
NA18998	151	TT	1	tt	0.048597964	TT
NA18940	152	TT	1	tt	0.123469507	TT
NA18949	153	TT	1	tt	0.078267549	TT
NA18953	154	CT	0.9647	ct	0.024639846	CT
NA18972	155	TT	0.9993	tt	0.020607953	TT
NA18976	156	CT	0.9539	ct	0.411840847	CT
NA18971	157	TT	1	tt	0.08024832	TT
blank	158	NN	0.4946	***NN***	***0***	nn
NA19000	159	TT	1	tt	0.35926786	TT
NA18951	160	TT	1	tt	0.446381784	nn
NA18948	161	CT	0.9017	ct	0.413122121	CT
NA18968	162	TT	1	tt	0.123425295	TT
NA18965	163	TT	1	tt	0.386886852	TT
NA18978	164	CC	0.9976	cc	0.220041844	CC
NA18974	165	TT	1	tt	0.247928726	TT
NA18992	166	CT	0.8977	ct	0.215725766	CT
NA19005	167	TT	1	tt	0.278204709	TT
NA18943	168	TT	1	tt	0.193136363	TT
NA18951	169	TT	1	tt	0.040607316	TT
NA18959	170	TT	1	tt	0.236406795	TT
NA18973	171	TT	1	tt	0.092565316	TT
NA18970	172	TT	1	tt	0.162528743	TT
NA18987	173	TT	1	tt	0.090021986	TT
NA18995	174	CT	0.9684	ct	0.509563875	CT
NA18999	175	TT	1	tt	0.10355588	TT
NA18947	176	TT	1	tt	0.166356465	TT

	MACGT discrepancy					
	LDA discrepancy					
NA	LDA output were not g					
XY	Manual calls are given					
		**1560434**				
**Coriell**	**Sample**	**LDA Call**	**LDA quality score**	**MACGT Call**	**MACGT quality score**	**HapMap Call**
NA18952	177	CT	0.9589	ct	0.315067908	CT
NA18969	178	TT	0.9997	tt	0.064157332	TT
NA18966	179	TT	1	tt	0.117329122	TT
NA18980	180	CT	0.964	ct	0.311923189	CT
NA18990	181	TT	1	tt	0.041981548	TT
NA18997	182	TT	1	tt	0.282468045	TT
NA19007	183	TT	0.9999	tt	0.083710927	TT
NA18944	184	TT	0.9999	*TT*	*0*	TT
NA18956	185	CT	0.9915	ct	0.342261667	CT
NA18960	186	TT	1	tt	0.292676781	TT
NA18975	187	TT	1	tt	0.193044216	TT
NA18995	188	CT	0.9714	ct	0.536250521	nn
NA18991	189	TT	1	tt	0.174960398	TT
NA18996	190	TT	1	*TT*	*0*	nn
NA19003	191	TT	1	tt	0.291964501	TT
NA18502	192	NN	0.75	tt	0.013939243	TT
NA19153	193	NN	0.75	tt	0.087365448	TT
NA18857	194	TT	1	tt	0.238638144	TT
NA19223	195	TT	0.998	tt	0.004362455	TT
NA19201	196	TT	1	tt	0.018530452	TT
NA18504	197	TT	0.9874	tt	0.039370334	TT
NA18870	198	TT	1	tt	0.026855808	TT
NA18863	199	NN	0.545	*TT*	*0*	TT
NA19145	200	TT	1	tt	0.26378985	TT
NA19137	201	TT	0.9995	tt	0.006303875	TT
NA19238	202	TT	1	tt	0.019648574	nn
NA18500	203	TT	0.7644	*TT*	*0*	TT
NA19144	204	NN	0.7131	tt	0.001059595	TT
NA19203	205	TT	1	tt	0.13195913	TT
NA19200	206	TT	1	tt	0.056928618	TT
NA18855	207	TT	1	tt	0.038540546	TT
NA18505	208	TT	1	tt	0.070426344	TT
NA19202	209	TT	1	tt	0.015223802	TT
NA18501	210	TT	0.9931	*TT*	*0*	TT
NA18861	211	NN	0.4735	*NN*	*0*	TT
NA19193	212	TT	0.9904	tt	0.001807062	TT
NA19143	213	TT	0.9692	*TT*	*0*	TT
NA18517	214	CT	0.965	ct	0.242778422	CT
NA18862	215	TT	0.9894	*TT*	*0*	TT
NA18856	216	TT	0.997	*tt*	*9.29E-04*	nn
NA19239	217	TT	1	tt	0.080573645	TT
NA19240	218	TT	1	tt	0.345798701	TT
NA18856	219	TT	1	tt	0.005225485	TT
NA18503	220	TT	1	tt	0.093132832	TT

	MACGT discrepancy					
	LDA discrepancy					
NA	LDA output were not g					
XY	Manual calls are given					
		1560434				
**Coriell**	**Sample**	**LDA Call**	**LDA quality score**	**MACGT Call**	**MACGT quality score**	**HapMap Call**
NA18871	221	CT	0.961	ct	0.34123601	CT
NA19221	222	TT	1	tt	0.374325337	TT
NA19209	223	TT	1	tt	0.193237569	TT
NA19152	224	TT	0.9313	tt	0.080138153	TT
NA18515	225	CT	0.9368	ct	0.015103592	CT
NA19238	226	TT	1	tt	0.203689198	TT
NA19154	227	TT	1	tt	0.428655953	TT
NA19210	228	TT	1	tt	0.312039526	TT
NA19211	229	TT	1	tt	0.249704313	TT
NA18862	230	TT	1	tt	0.537670216	nn
NA18872	231	TT	1	tt	0.17528534	TT
NA19139	232	TT	1	tt	0.199272799	TT
NA19222	233	TT	1	tt	0.201913241	TT
NA19194	234	TT	1	tt	0.364413059	TT
NA19138	235	TT	0.9999	tt	0.010944807	TT
NA19204	236	TT	1	tt	0.102705809	TT
NA18516	237	TT	0.9999	tt	0.022808329	TT
NA19205	238	TT	1	tt	0.286001145	TT
NA19192	239	TT	0.8784	tt	0.015358489	TT
NA18912	240	TT	1	tt	0.190634135	nn
NA18508	241	TT	1	tt	0.209884778	TT
NA19142	242	CT	0.9486	ct	0.059808706	CT
NA18852	243	TT	1	tt	0.230032775	TT
NA18507	244	TT	1	tt	0.056620536	TT
blank	245	NN	0.6006	***NN***	***0***	nn
NA19101	246	TT	1	tt	0.284070392	TT
NA19172	247	TT	1	tt	0.048600523	TT
NA19160	248	TT	1	tt	0.038556158	TT
NA19129	249	TT	0.9999	tt	0.017536015	TT
NA18913	250	TT	1	tt	0.010464814	TT
NA19120	251	NN	0.7152	***TT***	***0***	TT
NA19159	252	TT	1	tt	0.097229614	TT
NA18523	253	TT	1	tt	0.205993587	TT
NA19102	254	TT	0.8809	***TT***	***0***	TT
NA19092	255	TT	1	tt	0.308847567	TT
NA18521	256	NN	0.75	tt	0.071415208	TT
NA18912	257	TT	0.75	tt	0.165427093	TT
NA19206	258	TT	1	tt	0.149636661	TT
NA19128	259	TT	1	tt	0.169561602	TT
NA18859	260	TT	1	tt	0.145138182	nn
NA19094	261	TT	1	tt	0.181461912	TT
NA19171	262	TT	0.9974	tt	0.202956463	TT
NA18860	263	TT	1	tt	0.167762149	TT
NA18858	264	TT	1	tt	0.100907164	TT

	MACGT discrepancy					
	LDA discrepancy					
NA	LDA output were not g					
XY	Manual calls are given					
		1560434				
**Coriell**	**Sample**	**LDA Call**	**LDA quality score**	**MACGT Call**	**MACGT quality score**	**HapMap Call**
NA19099	265	TT	1	tt	0.123876017	TT
NA18506	266	TT	0.8498	tt	0.056215496	TT
NA19131	267	CT	0.976	ct	0.279659916	CT
NA19116	268	CT	0.831	***CT***	***0***	CT
NA19161	269	TT	1	tt	0.172515054	TT
NA19140	270	CT	0.9629	ct	0.218382112	CT
NA19207	271	TT	1	tt	0.022745188	TT
NA19141	272	TT	1	tt	0.005693296	TT
NA18854	273	TT	1	tt	0.143593085	TT
NA19098	274	TT	1	tt	0.105712076	TT
NA18522	275	TT	1	tt	0.100387306	TT
NA18914	276	TT	1	tt	0.067360109	TT
NA18853	277	TT	1	tt	0.279305842	TT
NA18859	278	TT	1	***TT***	***0***	TT
NA19100	279	TT	1	***TT***	***0***	TT
NA19093	280	NN	0.7422	***TT***	***0***	TT
NA19132	281	CT	0.9579	ct	0.125278143	CT
NA19130	282	TT	1	tt	0.089099448	TT
NA19208	283	TT	1	tt	0.065190911	TT
NA19127	284	TT	1	tt	0.022770984	TT
NA19173	285	TT	0.7503	tt	0.1518011	TT
NA19119	286	CT	0.8617	***CT***	***0***	CT
NA19103	287	TT	1	tt	0.031581096	TT
	Discrepancies	0		0		
	NNs	10		1		
	Automated Calls	274		255		
	Manual Calls	0		19		
	Concordant Calls	245		254		255

	MACGT discrepancy					
	LDA discrepancy					
NA	LDA output were not g					
XY	Manual calls are given					
		**1607185**				
**Coriell**	**Sample**	**LDA Call**	**LDA quality score**	**MACGT Call**	**MACGT quality score**	**HapMap Call**
NA12753	1		NA	AA	0.409813201	
NA12875	2	AA	0.9976	aa	0.374452634	AA
NA12044	3	GA	0.9879	ga	0.665462556	GA
NA06993	4	AA	0.9994	aa	0.274765446	AA
NA12716	5	AA	0.9891	aa	0.28096039	AA
NA12760	6	AA	0.9996	aa	0.417899079	AA
NA07348	7	GA	0.9961	ga	0.535674245	GA
NA12707	8	AA	0.9868	aa	0.146534548	AA
NA12156	9		NA	AA	0.392152649	
NA11992	10	GA	0.9921	ga	0.364145114	GA
NA12239	11	AA	0.9999	aa	0.112545989	AA
NA12878	12	AA	0.9998	aa	0.299636024	AA
NA11993	13	AA	0.8753	ga	0.02107101	AA
NA12750	14	AA	0.9643	aa	0.091563689	AA
NA12146	15	GA	1	ga	0.082978154	GA
NA11839	16	AA	0.9884	aa	0.056270322	AA
NA12156.dup	17	AA	0.9975	aa	0.075869232	nn
NA11829	18		NA	GA	0.639559134	
NA12154	19	GA	0.9994	ga	0.440977788	GA
NA10856	20	GA	0.9992	ga	0.346864373	GA
NA06985	21	AA	0.999	aa	0.213193604	AA
NA11831	22	AA	0.9572	aa	0.013666544	AA
NA12003.dup	23	AA	0.9552	aa	0.037010446	nn
NA10859	24	AA	0.9933	aa	0.124831875	AA
NA07056	25	AA	0.9513	aa	0.110112206	AA
NA07022	26	AA	0.9974	aa	0.275767961	AA
NA12762	27		NA	AA	0.472426309	
NA12874	28	AA	0.9688	aa	0.007080044	AA
NA12003	29	AA	0.9979	aa	0.248037053	AA
NA10835	30	GA	1	ga	0.559378185	GA
NA11882	31	AA	0.9875	aa	0.214698399	AA
NA07034	32	AA	0.9711	aa	0.199080479	AA
NA12056	33	GA	0.9995	ga	0.549311283	GA
NA12145	34	GA	0.9988	ga	0.008459246	GA
NA07019	35	AA	0.9974	aa	0.485067848	AA
NA06991	36		NA	AA	0.578736542	
NA12761	37	AA	0.9907	aa	0.138873025	AA
NA06994	38	AA	0.9937	aa	0.174509734	AA
NA12864	39	AA	0.9975	aa	0.383404688	AA
NA07055	40	AA	0.9988	aa	0.349040275	AA
NA10863	41	AA	0.991	aa	0.337905825	AA
NA12763	42	GA	0.9994	ga	0.100018615	GA
NA10831	43	AA	0.9985	aa	0.362047653	AA
NA11840	44	AA	0.9912	aa	0.381633157	AA

	MACGT discrepancy					
	LDA discrepancy					
NA	LDA output were not g					
XY	Manual calls are given					
		1607185				
**Coriell**	**Sample**	**LDA Call**	**LDA quality score**	**MACGT Call**	**MACGT quality score**	**HapMap Call**
NA10830	45	GA	0.9991	ga	0.37967173	GA
NA11993.dup	46	AA	0.9964	aa	0.442237113	nn
NA12751	47	AA	0.9996	aa	0.452221302	AA
NA12814	48	AA	0.9989	aa	0.333258011	AA
NA10857	49	GA	1	ga	0.344494499	GA
NA07357	50	GA	0.9998	ga	0.475151845	GA
NA07000	51	AA	0.9983	aa	0.409569774	AA
NA12802	52	AA	0.9994	aa	0.395750057	AA
NA10855	53	AA	0.9997	aa	0.060968753	AA
NA11995	54		NA	AA	0.489811068	
NA10839	55	AA	0.9784	aa	0.180019063	AA
NA10847	56	AA	0.9723	aa	0.172663472	AA
NA12006	57	GA	0.9994	ga	0.065789766	GA
NA12144	58	AA	0.9998	aa	0.43915028	AA
NA11832	59	AA	0.989	aa	0.205719255	AA
NA12813	60	AA	0.9991	aa	0.442223218	AA
blank	61	AA	0.8881	aa	0.2239219	nn
NA12891	62	AA	0.9997	aa	0.431195753	AA
NA12717	63		NA	AA	0.299961582	
NA10861	64	AA	0.9993	aa	0.225373874	AA
NA12004	65	AA	0.9997	aa	0.131070098	AA
NA07048	66	AA	0.9993	aa	0.448646057	AA
NA12801	67	AA	0.9995	aa	0.283724266	AA
NA11830	68	AA	0.9997	aa	0.247260139	AA
NA12057	69	AA	0.9997	aa	0.327783016	AA
NA11994	70		NA	AA	0.420780349	
NA12740	71	AA	0.9956	aa	0.464738852	AA
NA12005	72	AA	0.9346	aa	0.023227002	AA
NA07345	73	AA	0.9711	aa	0.052519319	AA
NA10854	74	AA	0.9924	aa	0.229900683	AA
NA12872	75	AA	0.9997	aa	0.481588171	AA
NA12865	76	AA	0.9997	aa	0.173215323	AA
NA10860	77		NA	AA	0.495080798	
NA12873	78	AA	0.9993	aa	0.425548199	AA
NA12752	79	AA	0.9996	aa	0.442470234	AA
NA10851	80	GA	0.9995	ga	0.499269717	GA
NA07029	81	AA	0.9997	aa	0.587699321	AA
NA12248	82	GA	0.9972	ga	0.358846699	GA
NA12155	83	AA	0.9927	aa	0.033653515	AA
NA10838	84	AA	0.9989	aa	0.443104241	AA
NA12812	85	AA	0.9995	aa	0.407960091	AA
NA12248.dup	86	GA	0.9997	ga	0.478165207	nn
NA12043	87	AA	0.9975	aa	0.27779772	AA
NA12234	88	AA	0.9995	aa	0.338902756	AA

	MACGT discrepancy					
	LDA discrepancy					
NA	LDA output were not g					
XY	Manual calls are given					
		1607185				
**Coriell**	**Sample**	**LDA Call**	**LDA quality score**	**MACGT Call**	**MACGT quality score**	**HapMap Call**
NA12892	89	AA	0.9994	aa	0.405579971	AA
NA10846	90	GA	0.9981	ga	0.475152814	GA
NA06993.dup	91	AA	0.9998	aa	0.239591605	nn
NA12249	92	AA	0.9311	aa	0.036522779	AA
NA11881	93	AA	0.9353	aa	0.124309264	AA
NA12815	94	AA	0.9973	aa	0.107659691	AA
NA12264	95	AA	0.9984	aa	0.380244877	AA
NA18526	96	AA	0.9952	aa	0.460123368	AA
NA18562	97	AA	0.9821	aa	0.37677566	AA
NA18545	98	AA	0.9734	aa	0.118873556	AA
NA18609	99	AA	0.9983	aa	0.123211236	AA
NA18566	100	AA	0.9882	aa	0.150396505	AA
NA18621	101	AA	0.9831	aa	0.212584664	AA
NA18577	102	AA	0.8995	aa	0.215290699	AA
NA18635	103	AA	0.9623	aa	0.255881765	AA
NA18524	104	AA	0.9936	aa	0.468588485	AA
NA18537	105	AA	0.9049	aa	0.205310679	AA
NA18572	106	AA	0.9215	aa	0.228660579	AA
NA18552	107	AA	0.7745	aa	0.062701907	AA
NA18563	108	AA	0.9401	aa	0.143115719	AA
NA18594	109	AA	0.8505	aa	0.174219267	nn
NA18624	110	AA	0.9997	aa	0.474419851	AA
NA18592	111	AA	0.9958	aa	0.370134459	AA
NA18529	112	AA	0.9913	aa	0.467483512	AA
NA18603	113	AA	0.9933	aa	0.460452968	nn
NA18547	114	AA	0.993	aa	0.145411775	AA
NA18611	115	AA	0.9942	aa	0.340085317	AA
NA18570	116	AA	0.9774	aa	0.095650545	AA
NA18622	117	AA	0.9998	aa	0.378909326	AA
NA18579	118	AA	0.9993	aa	0.178720421	AA
NA18636	119	AA	0.9982	aa	0.180096304	AA
NA18558	120	AA	0.9995	aa	0.413964796	AA
NA18540	121	AA	0.9996	aa	0.247976747	AA
NA18609	122	AA	0.9985	aa	0.192265689	nn
NA18555	123	AA	0.9996	aa	0.083359357	AA
NA18612	124	AA	0.9996	aa	0.179501303	AA
NA18573	125	AA	0.9973	aa	0.289181865	AA
NA18632	126	AA	0.9998	aa	0.422125853	AA
NA18593	127	AA	0.9998	aa	0.331513166	AA
NA18532	128	AA	0.9939	aa	0.24138025	AA
NA18605	129	AA	0.9999	aa	0.42539436	AA
NA18550	130	AA	0.9998	aa	0.508568417	AA
NA18603	131	AA	0.9994	aa	0.476406716	AA
NA18571	132	AA	0.9996	aa	0.48569398	AA

	MACGT discrepancy					
	LDA discrepancy					
NA	LDA output were not g					
XY	Manual calls are given					
		**1607185**				
**Coriell**	**Sample**	**LDA Call**	**LDA quality score**	**MACGT Call**	**MACGT quality score**	**HapMap Call**
NA18623	133	AA	0.9998	aa	0.410215394	AA
NA18582	134	AA	0.9984	aa	0.435919725	AA
NA18637	135	AA	0.995	aa	0.244220922	AA
NA18561	136	AA	0.9996	aa	0.470045689	AA
NA18542	137	AA	0.9999	aa	0.405686157	AA
NA18608	138	AA	0.9999	aa	0.364214792	AA
NA18564	139	AA	0.9986	aa	0.438959284	AA
NA18620	140	AA	0.9985	aa	0.518145861	AA
NA18576	141	AA	0.9987	aa	0.023390388	AA
NA18633	142	AA	0.9961	aa	0.377906716	AA
NA18594	143	AA	0.9998	aa	0.419892263	AA
NA18942	144	AA	0.9998	aa	0.533775053	AA
NA18945	145	AA	0.9999	aa	0.602202578	AA
NA18964	146	AA	0.9994	aa	0.46183634	AA
NA18961	147	AA	0.9999	aa	0.543568396	AA
NA18967	148	AA	0.9997	aa	0.442942992	AA
NA18981	149	AA	0.9998	aa	0.426361733	AA
NA18994	150	AA	0.9992	aa	0.463983358	AA
NA18998	151	AA	0.9991	aa	0.32610263	AA
NA18940	152	AA	0.9952	aa	0.341734954	AA
NA18949	153	AA	0.9988	aa	0.265397193	AA
NA18953	154	AA	0.998	aa	0.135201532	AA
NA18972	155	AA	0.9979	aa	0.066716731	AA
NA18976	156	AA	0.9997	aa	0.01633645	AA
NA18971	157	AA	0.999	aa	0.268728035	AA
blank	158	GG	1	***NN***	***0***	nn
NA19000	159	AA	0.9993	aa	0.219858983	AA
NA18951	160	AA	0.9989	aa	0.025944321	nn
NA18948	161	AA	0.996	ga	0.049678671	AA
NA18968	162	AA	0.9998	aa	0.184806863	AA
NA18965	163	AA	0.9997	aa	0.236030714	AA
NA18978	164	AA	0.9996	aa	0.186761483	AA
NA18974	165	AA	0.9996	aa	0.15903359	AA
NA18992	166	AA	0.9988	ga	0.017537033	AA
NA19005	167	AA	0.9997	aa	0.451779313	AA
NA18943	168	AA	0.9998	aa	0.316955382	AA
NA18951	169	AA	0.9999	aa	0.205254698	AA
NA18959	170	AA	0.9999	aa	0.386997012	AA
NA18973	171	AA	0.9999	aa	0.506369478	AA
NA18970	172	AA	0.9999	aa	0.391674197	AA
NA18987	173	AA	0.9997	aa	0.435595594	AA
NA18995	174	AA	0.9984	aa	0.463061215	AA
NA18999	175	AA	0.9999	aa	0.390380392	AA
NA18947	176	AA	0.9976	ga	0.005535682	AA

	MACGT discrepancy					
	LDA discrepancy					
NA	LDA output were not g					
XY	Manual calls are given					
		1607185				
**Coriell**	**Sample**	**LDA Call**	**LDA quality score**	**MACGT Call**	**MACGT quality score**	**HapMap Call**
NA18952	177	AA	0.9999	aa	0.523072215	AA
NA18969	178	AA	0.9996	aa	0.101028173	AA
NA18966	179	AA	0.9999	aa	0.336093229	AA
NA18980	180	AA	0.9997	aa	0.44243685	AA
NA18990	181	AA	0.9998	ga	0.012253955	AA
NA18997	182	AA	0.9994	aa	0.455820585	AA
NA19007	183	AA	0.9999	aa	0.185051545	AA
NA18944	184	AA	0.9277	aa	0.395109742	AA
NA18956	185	AA	0.998	aa	0.188235407	AA
NA18960	186	AA	0.9985	aa	0.313382827	AA
NA18975	187	AA	1	aa	0.165845962	AA
NA18995	188	AA	0.9989	aa	0.437390884	nn
NA18991	189	AA	0.9951	aa	0.211007344	AA
NA18996	190	AA	0.9998	aa	0.36700055	nn
NA19003	191	AA	0.9996	aa	0.456337365	AA
NA18502	192	GA	0.9999	ga	0.404893173	GA
NA19153	193	AA	0.9458	aa	0.36346921	AA
NA18857	194	AA	0.9884	aa	0.689579691	AA
NA19223	195	AA	0.9591	aa	0.576565416	AA
NA19201	196	AA	0.9415	aa	0.572274687	AA
NA18504	197	GG	0.9997	gg	0.146081338	GG
NA18870	198	GA	0.9998	ga	0.733586768	GA
NA18863	199	GG	0.9992	gg	0.372968864	GG
NA19145	200	AA	0.9975	aa	0.391630028	AA
NA19137	201	AA	0.9808	aa	0.483929914	AA
NA19238	202	GA	0.9863	ga	0.295949333	nn
NA18500	203	AA	0.9896	aa	0.109719632	AA
NA19144	204	GA	0.989	ga	0.08098885	GA
NA19203	205	AA	0.9981	aa	0.246126584	AA
NA19200	206	AA	0.9929	aa	0.480350333	AA
NA18855	207	AA	0.9998	aa	0.382434864	AA
NA18505	208	AA	0.9985	aa	0.407243931	AA
NA19202	209	AA	0.987	aa	0.520929072	AA
NA18501	210	AA	0.9357	aa	0.085885066	AA
NA18861	211		NA	GA	0.408103201	
NA19193	212		NA	aa	0.060295601	
NA19143	213	GA	0.9988	ga	0.541252281	GA
NA18517	214	GA	0.9975	ga	0.483242649	GA
NA18862	215	GA	0.9997	ga	0.503857042	GA
NA18856	216	AA	0.9984	aa	0.032836332	nn
NA19239	217	AA	0.9913	aa	0.040381454	AA
NA19240	218	GA	0.8067	ga	0.076057027	GA
NA18856	219	AA	0.9997	aa	0.194818871	AA
NA18503	220	GA	0.9991	ga	0.069468593	GA

	MACGT discrepancy					
	LDA discrepancy					
NA	LDA output were not g					
XY	Manual calls are given					
		**1607185**				
**Coriell**	**Sample**	**LDA Call**	**LDA quality score**	**MACGT Call**	**MACGT quality score**	**HapMap Call**
NA18871	221	GA	0.9989	ga	0.029712808	GA
NA19221	222	AA	0.9928	aa	0.462238332	AA
NA19209	223	GA	0.9999	ga	0.010428697	GA
NA19152	224	AA	0.9993	aa	0.355076051	AA
NA18515	225	AA	0.7658	aa	0.270738326	AA
NA19238	226	GA	0.9708	ga	0.320126875	GA
NA19154	227	AA	0.9868	aa	0.54069495	AA
NA19210	228	AA	0.9983	aa	0.431136	AA
NA19211	229	GA	0.9937	ga	0.503680257	GA
NA18862	230	GA	0.9625	ga	0.449711527	nn
NA18872	231		NA	GG	0.46475089	
NA19139	232	AA	0.9977	aa	0.466572575	AA
NA19222	233	AA	0.9971	aa	0.367708279	AA
NA19194	234	AA	0.9921	aa	0.601090811	AA
NA19138	235	AA	0.9993	aa	0.417685993	AA
NA19204	236	GA	0.9985	ga	0.573056053	GA
NA18516	237	AA	0.8251	aa	0.175270395	AA
NA19205	238	AA	0.9899	aa	0.484587114	AA
NA19192	239	GA	0.9999	ga	0.236297416	GA
NA18912	240	GA	0.9977	ga	0.07123111	nn
NA18508	241	AA	0.9949	aa	0.276819235	AA
NA19142	242	AA	0.9585	aa	0.115351495	AA
NA18852	243	GA	0.9979	ga	0.394850337	GA
NA18507	244	AA	0.9928	aa	0.089994581	AA
blank	245	GG	1	***NN***	***0***	nn
NA19101	246	AA	0.9982	aa	0.453058387	AA
NA19172	247	GA	0.9999	ga	0.24785043	GA
NA19160	248	AA	0.999	aa	0.393404919	AA
NA19129	249	AA	0.975	aa	0.213596721	AA
NA18913	250		NA	GA	0.371736893	
NA19120	251	AA	0.9632	aa	0.143681966	AA
NA19159	252		NA	GA	0.563871549	
NA18523	253	GA	0.9934	GA	0.642797761	GA
NA19102	254		NA	GA	0.708009733	
NA19092	255	GA	0.9998	ga	0.294945939	GA
NA18521	256	AA	0.8032	aa	0.124551497	AA
NA18912	257	GA	0.9993	ga	0.045585339	GA
NA19206	258	GA	0.9998	ga	0.177339125	GA
NA19128	259	AA	0.926	aa	0.276309688	AA
NA18859	260	AA	0.9812	aa	0.341792129	nn
NA19094	261	AA	0.9743	aa	0.415177708	AA
NA19171	262	AA	0.9768	aa	0.45507448	AA
NA18860	263	AA	0.8854	aa	0.233061036	AA
NA18858	264	AA	0.9999	aa	0.395096185	AA

	MACGT discrepancy					
	LDA discrepancy					
NA	LDA output were not g					
XY	Manual calls are given					
		1607185				
**Coriell**	**Sample**	**LDA Call**	**LDA quality score**	**MACGT Call**	**MACGT quality score**	**HapMap Call**
NA19099	265	AA	0.9999	aa	0.273549361	AA
NA18506	266	AA	0.9976	aa	0.214560072	AA
NA19131	267	GG	1	gg	0.410764422	GG
NA19116	268	AA	0.9203	aa	0.395925231	AA
NA19161	269	AA	0.9874	aa	0.333343242	AA
NA19140	270	GA	0.9931	ga	0.603015591	GA
NA19207	271	AA	0.9991	aa	0.273646771	AA
NA19141	272	AA	0.9953	aa	0.424665041	AA
NA18854	273	AA	0.9961	aa	0.401375342	AA
NA19098	274	AA	0.9846	aa	0.422832592	AA
NA18522	275	AA	0.9961	aa	0.298404293	AA
NA18914	276	GA	0.9846	ga	0.526970223	GA
NA18853	277	AA	0.9976	aa	0.135574474	AA
NA18859	278	AA	0.9724	aa	0.299675628	AA
NA19100	279	AA	0.954	aa	0.253927354	AA
NA19093	280	GA	0.9996	ga	0.582165238	GA
NA19132	281	GG	1	gg	0.364878809	GG
NA19130	282	GA	0.9994	ga	0.305140326	GA
NA19208	283	AA	0.9319	aa	0.449280353	AA
NA19127	284	GA	0.9939	ga	0.613617038	GA
NA19173	285	GA	0.9947	ga	0.242686867	GA
NA19119	286	AA	0.9929	aa	0.22741373	AA
NA19103	287	GA	1	ga	0.213373355	GA
	Discrepancies	0		5		
	NNs	0		0		
	Automated Calls	272		270		
	Manual Calls	0		2		
	Concordant Calls	253		248		253

	MACGT discrepancy					
	LDA discrepancy					
NA	LDA output were not g					
XY	Manual calls are given					
		**1777467**				
**Coriell**	**Sample**	**LDA Call**	**LDA quality score**	**MACGT Call**	**MACGT quality score**	**HapMap Call**
NA12753	1		NA	AA	0.420329696	
NA12875	2	GA	0.9984	ga	0.268474823	GA
NA12044	3	AA	1	aa	0.574135189	AA
NA06993	4	GA	1	ga	0.208626871	GA
NA12716	5	GA	0.9998	ga	0.171633614	GA
NA12760	6	GA	0.9989	ga	0.427145064	GA
NA07348	7	AA	0.9992	aa	0.396143739	AA
NA12707	8	AA	0.9995	aa	0.308000387	AA
NA12156	9		NA	AA	0.3318587	
NA11992	10	GA	0.9998	ga	0.038791733	GA
NA12239	11	AA	0.9928	aa	0.151297513	AA
NA12878	12	GA	1	ga	0.068800087	GA
NA11993	13	AA	0.9992	aa	0.009175914	AA
NA12750	14	GG	0.9158	gg	0.277662047	GG
NA12146	15	AA	1	aa	0.0831148	AA
NA11839	16	AA	1	aa	0.044898042	AA
NA12156.dup	17	AA	0.9999	aa	0.360282459	nn
NA11829	18		NA	AA	0.38677727	
NA12154	19	AA	0.9928	aa	0.332583124	AA
NA10856	20	GA	0.9947	ga	0.304047295	GA
NA06985	21	AA	0.9921	aa	0.52182581	AA
NA11831	22	GA	0.9999	ga	0.041344376	GA
NA12003.dup	23	AA	0.9743	aa	0.114061754	nn
NA10859	24	GG	0.7509	gg	0.31795466	GG
NA07056	25	GA	1	ga	0.06427236	GA
NA07022	26	GA	1	ga	0.309236713	GA
NA12762	27		NA	AA	0.433903008	
NA12874	28	AA	0.7515	aa	0.018501071	AA
NA12003	29	AA	0.9996	aa	0.434220132	AA
NA10835	30	AA	0.9999	aa	0.408717242	AA
NA11882	31	GA	0.9998	ga	0.35986356	GA
NA07034	32	AA	0.9987	aa	0.246124743	AA
NA12056	33	AA	0.9938	aa	0.509962302	AA
NA12145	34	AA	0.9996	aa	0.174377663	AA
NA07019	35	GA	1	ga	0.622616196	GA
NA06991	36		NA	GA	0.769461175	
NA12761	37	GA	1	ga	0.119978256	GA
NA06994	38	AA	1	aa	0.228258318	AA
NA12864	39	GA	1	ga	0.516145807	GA
NA07055	40	GA	0.9997	ga	0.31872357	GA
NA10863	41	AA	0.9997	aa	0.346987329	AA
NA12763	42	AA	1	aa	0.565968851	AA
NA10831	43	AA	1	aa	0.275328936	AA
NA11840	44	AA	1	aa	0.326285532	AA

	MACGT discrepancy					
	LDA discrepancy					
NA	LDA output were not g					
XY	Manual calls are given					
		**1777467**				
**Coriell**	**Sample**	**LDA Call**	**LDA quality score**	**MACGT Call**	**MACGT quality score**	**HapMap Call**
NA10830	45	GA	1	ga	0.502482611	GA
NA11993.dup	46	AA	0.9861	aa	0.334161837	nn
NA12751	47	GG	0.7503	gg	0.429682384	GG
NA12814	48	AA	0.9782	aa	0.246603089	AA
NA10857	49	GA	1	ga	0.277506644	GA
NA07357	50	AA	0.9975	aa	0.246946141	AA
NA07000	51	GA	1	ga	0.324097341	GA
NA12802	52	GA	1	ga	0.236330032	GA
NA10855	53	GA	1	ga	0.24439719	GA
NA11995	54		NA	AA	0.257353902	
NA10839	55	AA	0.9867	aa	0.21244334	AA
NA10847	56	AA	0.9622	aa	0.13649257	AA
NA12006	57	AA	0.9437	aa	0.235520242	AA
NA12144	58	AA	0.8464	aa	0.255313488	AA
NA11832	59	GA	1	ga	0.183374127	GA
NA12813	60	GA	1	ga	0.26030979	GA
blank	61	NN	0.5	***NN***	***0***	nn
NA12891	62	GG	0.7501	gg	0.322763224	GG
NA12717	63		NA	AA	0.172562464	
NA10861	64	AA	0.7581	aa	0.014217915	AA
NA12004	65	AA	1	aa	0.135339554	AA
NA07048	66	GA	1	ga	0.287390952	GA
NA12801	67	AA	0.9154	aa	0.021199991	AA
NA11830	68	GA	1	ga	0.136635772	GA
NA12057	69	GA	1	ga	0.006970167	GA
NA11994	70		NA	GA	0.492521054	
NA12740	71	GG	0.75	gg	0.226350356	GG
NA12005	72	NN	0.5083	***AA***	***0***	AA
NA07345	73	GA	0.995	***GA***	***0***	GA
NA10854	74	AA	0.928	aa	0.020764759	AA
NA12872	75	NN	0.5203	***GA***	***0***	GA
NA12865	76	AA	0.9836	aa	0.164188014	AA
NA10860	77		NA	AA	0.265308855	
NA12873	78	AA	0.9675	aa	0.182524535	AA
NA12752	79	GG	0.7501	gg	0.379895362	GG
NA10851	80	GA	0.9996	ga	0.001105168	GA
NA07029	81	AA	0.9999	aa	0.019707094	AA
NA12248	82	AA	0.7606	***AA***	***0***	AA
NA12155	83	AA	0.9798	aa	0.005679299	AA
NA10838	84	AA	0.7516	aa	0.001493079	AA
NA12812	85	GA	1	ga	0.010740599	GA
NA12248.dup	86	AA	0.7586	aa	0.02998925	nn
NA12043	87	NN	0.5376	***GA***	***0***	GA
NA12234	88	AA	0.9999	aa	0.207795503	AA

	MACGT discrepancy					
	LDA discrepancy					
NA	LDA output were not g					
XY	Manual calls are given					
		**1777467**				
**Coriell**	**Sample**	**LDA Call**	**LDA quality score**	**MACGT Call**	**MACGT quality score**	**HapMap Call**
NA12892	89	AA	0.9998	aa	0.017310214	AA
NA10846	90	AA	0.7882	aa	0.125036301	AA
NA06993.dup	91	GA	1	ga	0.143335372	nn
NA12249	92	GA	1	ga	0.00189975	GA
NA11881	93	GA	1	ga	0.002355129	GA
NA12815	94	GA	1	ga	0.001893751	GA
NA12264	95	AA	0.9893	aa	0.177382147	AA
NA18526	96	GA	0.9861	ga	0.376789081	GA
NA18562	97	AA	1	aa	0.227421899	AA
NA18545	98	GA	1	ga	0.083458091	GA
NA18609	99	GA	1	ga	0.002481654	GA
NA18566	100	GG	0.7501	gg	0.113872989	GG
NA18621	101	AA	0.9998	aa	0.150304579	AA
NA18577	102	GA	1	ga	0.060967087	GA
NA18635	103	GA	1	ga	0.03414522	GA
NA18524	104	GA	0.9999	ga	0.486374476	GA
NA18537	105	AA	1	aa	0.034190891	AA
NA18572	106	AA	0.9334	aa	0.0710768	AA
NA18552	107	AA	0.7572	aa	0.016063928	AA
NA18563	108	AA	1	aa	0.045343342	AA
NA18594	109	GA	1	ga	0.048589279	nn
NA18624	110	AA	1	aa	0.272894739	AA
NA18592	111	AA	0.8142	aa	0.485217207	AA
NA18529	112	AA	1	aa	0.294066154	AA
NA18603	113	GA	1	ga	0.425010138	nn
NA18547	114	AA	0.7547	aa	0.262719928	AA
NA18611	115	GA	1	ga	0.079756946	GA
NA18570	116	AA	0.8968	aa	0.287413514	AA
NA18622	117	AA	1	aa	0.407977781	AA
NA18579	118	GA	1	ga	0.12151153	GA
NA18636	119	AA	0.9997	aa	0.36693239	AA
NA18558	120	AA	0.9983	aa	0.412483639	AA
NA18540	121	GA	1	ga	0.003239022	GA
NA18609	122	GA	1	ga	0.07811079	nn
NA18555	123	AA	0.7737	aa	0.001630241	AA
NA18612	124	GG	0.9703	gg	0.526418201	GG
NA18573	125	AA	0.9151	aa	0.097298195	AA
NA18632	126	GG	0.75	gg	0.376770205	GG
NA18593	127	NN	0.7039	***GA***	***6.56E-04***	GA
NA18532	128		NA	AA	0.36676113	
NA18605	129	GA	1	ga	0.040313408	GA
NA18550	130	AA	0.7947	aa	0.177841464	AA
NA18603	131	GA	0.9999	ga	0.097892964	GA
NA18571	132	GA	1	ga	0.063381321	GA

	MACGT discrepancy					
	LDA discrepancy					
NA	LDA output were not g					
XY	Manual calls are given					
		**1777467**				
**Coriell**	**Sample**	**LDA Call**	**LDA quality score**	**MACGT Call**	**MACGT quality score**	**HapMap Call**
NA18623	133	GG	0.75	gg	0.019445363	GG
NA18582	134	AA	1	aa	0.43327792	AA
NA18637	135	AA	1	aa	0.432284995	AA
NA18561	136	GA	1	ga	0.062251467	GA
NA18542	137	GA	1	ga	0.003723524	GA
NA18608	138	AA	0.9997	aa	0.389658137	AA
NA18564	139	NN	0.75	***GA***	***5.16E-04***	GA
NA18620	140	AA	0.9597	aa	0.193556805	AA
NA18576	141	GA	0.9999	ga	0.003176483	GA
NA18633	142	NN	0.75	***GA***	***0***	GA
NA18594	143	NN	0.5	***GA***	***0***	GA
NA18942	144	GA	0.9991	ga	0.260775622	GA
NA18945	145	GA	0.9996	ga	0.194834211	GA
NA18964	146	GA	1	ga	0.349707432	GA
NA18961	147	GA	0.9999	ga	0.422273019	GA
NA18967	148	AA	1	aa	0.41312673	AA
NA18981	149	GA	0.9997	ga	0.288456647	GA
NA18994	150	AA	1	aa	0.380761516	AA
NA18998	151	GA	1	ga	0.070275745	GA
NA18940	152	GA	1	ga	0.194904039	GA
NA18949	153	AA	0.9982	aa	0.239548369	AA
NA18953	154	AA	0.9977	aa	0.330947924	AA
NA18972	155	GG	0.7514	gg	0.087362727	GG
NA18976	156	AA	1	aa	0.362570252	AA
NA18971	157	GA	1	ga	0.124757879	GA
blank	158	NN	0.5249	***NN***	***0***	nn
NA19000	159	GA	0.9596	ga	0.220860999	GA
NA18951	160	GA	0.9185	ga	0.135041249	nn
NA18948	161	AA	1	aa	0.475996504	AA
NA18968	162	AA	1	aa	0.578187858	AA
NA18965	163	GA	0.9338	ga	0.107419862	GA
NA18978	164	GA	0.8563	ga	0.112321758	GA
NA18974	165	AA	1	aa	0.431828478	AA
NA18992	166	AA	1	aa	0.094116182	AA
NA19005	167	AA	0.9995	aa	0.405399016	AA
NA18943	168	AA	1	aa	0.374274391	AA
NA18951	169	GA	0.9999	ga	0.25860518	GA
NA18959	170	GA	0.999	ga	0.367522166	GA
NA18973	171	AA	1	aa	0.50831804	AA
NA18970	172	GG	0.9868	gg	0.401994028	GG
NA18987	173	GA	0.9994	ga	0.103443596	GA
NA18995	174	GA	0.9998	ga	0.342354712	GA
NA18999	175	GG	0.9342	gg	0.369332717	GG
NA18947	176	GG	1	gg	0.269223919	GG

	MACGT discrepancy					
	LDA discrepancy					
NA	LDA output were not g					
XY	Manual calls are given					
		**1777467**				
**Coriell**	**Sample**	**LDA Call**	**LDA quality score**	**MACGT Call**	**MACGT quality score**	**HapMap Call**
NA18952	177	GA	0.9987	ga	0.174148725	GA
NA18969	178	GA	0.9981	ga	0.133420017	GA
NA18966	179	GA	1	ga	0.206010041	GA
NA18980	180	GA	0.9996	ga	0.110154378	GA
NA18990	181	GA	1	ga	0.175071584	GA
NA18997	182	AA	1	aa	0.70357373	AA
NA19007	183	GA	1	ga	0.205497169	GA
NA18944	184	GG	0.9247	gg	0.125168597	GG
NA18956	185	AA	1	aa	0.437990815	AA
NA18960	186	GG	0.9726	gg	0.519115829	GG
NA18975	187	GA	1	ga	0.407279363	GA
NA18995	188	GA	1	ga	0.544285838	nn
NA18991	189	GA	1	ga	0.396301071	GA
NA18996	190	GA	1	ga	0.055136947	nn
NA19003	191	AA	0.9999	aa	0.68296423	AA
NA18502	192	NN	0.5193	***GA***	***0***	GA
NA19153	193	GA	1	ga	0.0386623	GA
NA18857	194	GG	0.75	***GG***	***0***	GG
NA19223	195	GA	1	***GA***	***0***	GA
NA19201	196	NN	0.5165	***GA***	***0***	GA
NA18504	197	NN	0.5058	***AA***	***0***	AA
NA18870	198	NN	0.7444	***AA***	***0***	AA
NA18863	199	AA	0.9948	aa	0.012494267	AA
NA19145	200	GG	0.75	gg	0.088363977	GG
NA19137	201	GA	0.993	***GA***	***0***	GA
NA19238	202	AA	1	aa	0.179976319	nn
NA18500	203	GA	1	***GA***	***0***	GA
NA19144	204	GA	0.999	***GA***	***0***	GA
NA19203	205	GA	0.9998	ga	0.094982734	GA
NA19200	206	AA	0.963	***AA***	***0***	AA
NA18855	207	GA	1	ga	0.001698039	GA
NA18505	208	GG	0.8184	gg	0.484132862	GG
NA19202	209	AA	0.9985	***AA***	***0***	AA
NA18501	210	NN	0.6146	***GA***	***0***	GA
NA18861	211	GA	0.7717	***GA***	***0***	GA
NA19193	212	NN	0.7446	***AA***	***0***	AA
NA19143	213	NN	0.75	***GA***	***0***	GA
NA18517	214	AA	1	aa	0.162842071	AA
NA18862	215	AA	0.96	***AA***	***0***	AA
NA18856	216	GA	0.9917	ga	0.070777762	nn
NA19239	217	GA	1	ga	0.058465482	GA
NA19240	218	AA	1	aa	0.175815391	AA
NA18856	219	GA	0.9996	ga	0.159032085	GA
NA18503	220	GA	1	ga	0.168179792	GA

	MACGT discrepancy					
	LDA discrepancy					
NA	LDA output were not g					
XY	Manual calls are given					
		**1777467**				
**Coriell**	**Sample**	**LDA Call**	**LDA quality score**	**MACGT Call**	**MACGT quality score**	**HapMap Call**
NA18871	221	GA	0.9999	ga	0.440740871	GA
NA19221	222	GA	0.9997	ga	0.640343292	GA
NA19209	223	GA	1	ga	0.258757557	GA
NA19152	224	GA	1	ga	0.422548612	GA
NA18515	225	GA	1	ga	0.474912303	GA
NA19238	226	AA	1	aa	0.199298459	AA
NA19154	227	AA	0.9845	aa	0.348982896	AA
NA19210	228	GA	1	ga	0.237499509	GA
NA19211	229	GA	0.9995	ga	0.266745927	GA
NA18862	230	AA	0.9998	aa	0.424793898	nn
NA18872	231	GA	0.9999	ga	0.287019852	GA
NA19139	232	GA	0.9998	ga	0.186428784	GA
NA19222	233	GA	0.9999	ga	0.124405845	GA
NA19194	234	AA	0.9104	aa	0.439041426	AA
NA19138	235	GA	0.9992	ga	0.123029138	GA
NA19204	236	GA	1	ga	0.260192066	GA
NA18516	237	GG	0.7522	gg	0.201907268	GG
NA19205	238	GG	0.7765	gg	0.225816865	GG
NA19192	239	GA	0.9992	ga	0.165309536	GA
NA18912	240	GA	0.999	ga	0.43904763	nn
NA18508	241	AA	0.998	aa	0.227018471	AA
NA19142	242	AA	0.9996	aa	0.293326259	AA
NA18852	243	GA	0.9988	ga	0.217114075	GA
NA18507	244	GA	0.9986	ga	0.112396683	GA
blank	245	NN	0.5002	***NN***	***0***	nn
NA19101	246	AA	0.9901	aa	0.224075195	AA
NA19172	247	GA	0.9992	ga	0.073485915	GA
NA19160	248	GA	0.9995	ga	0.168362702	GA
NA19129	249	GG	0.927	gg	0.326895285	GG
NA18913	250	GA	0.9998	ga	0.137924262	GA
NA19120	251	AA	0.9999	aa	0.075843347	AA
NA19159	252	AA	0.9974	aa	0.015801041	AA
NA18523	253	GA	0.9999	ga	0.186366786	GA
NA19102	254	AA	0.9999	aa	0.050223304	AA
NA19092	255	AA	1	aa	0.4550132	AA
NA18521	256	GG	0.7625	gg	0.480896743	GG
NA18912	257	GA	1	ga	0.360615865	GA
NA19206	258	GG	0.7503	gg	0.145162155	GG
NA19128	259	GA	0.9999	ga	0.364826041	GA
NA18859	260	GG	0.7553	gg	0.489311949	nn
NA19094	261	AA	0.9992	aa	0.397067045	AA
NA19171	262	GA	1	ga	0.503449509	GA
NA18860	263	GG	0.7502	gg	0.18129723	GG
NA18858	264	GG	0.75	gg	0.133245765	GG

	MACGT discrepancy					
	LDA discrepancy					
NA	LDA output were not g					
XY	Manual calls are given					
		1777467				
**Coriell**	**Sample**	**LDA Call**	**LDA quality score**	**MACGT Call**	**MACGT quality score**	**HapMap Call**
NA19099	265	GG	0.762	gg	0.339696629	GG
NA18506	266	GA	1	ga	0.098831115	GA
NA19131	267	GG	0.7588	gg	0.111099769	GG
NA19116	268	AA	1	aa	0.077217444	AA
NA19161	269	GA	1	ga	0.422254672	GA
NA19140	270	GA	1	ga	0.240122639	GA
NA19207	271	AA	0.9987	aa	0.087417056	AA
NA19141	272	AA	0.9967	aa	0.25430054	AA
NA18854	273	GA	1	ga	0.022256428	GA
NA19098	274	AA	1	aa	0.303567997	AA
NA18522	275	GG	0.7501	gg	0.305032875	GG
NA18914	276	GG	0.8357	gg	0.286408286	GG
NA18853	277	GA	0.999	ga	0.131505398	GA
NA18859	278	GG	1	gg	0.010613545	GG
NA19100	279	GA	1	ga	0.020787474	GA
NA19093	280	GA	1	ga	0.443223711	GA
NA19132	281	GG	0.75	gg	0.133709116	GG
NA19130	282	GG	0.7559	gg	0.429522458	GG
NA19208	283	GA	1	ga	0.050775409	GA
NA19127	284	GA	0.9998	ga	0.106610892	GA
NA19173	285	GA	0.9996	ga	0.065671839	GA
NA19119	286	GA	1	ga	0.261268925	GA
NA19103	287	AA	1	aa	0.21015075	AA
	Discrepancies	0		0		
	NNs	15		1		
	Automated Calls	277		249		
	Manual Calls	0		28		
	Concordant Calls	244		258		258

	MACGT discrepancy					
	LDA discrepancy					
NA	LDA output were not g					
XY	Manual calls are given					
		1825443				
**Coriell**	**Sample**	**LDA Call**	**LDA quality score**	**MACGT Call**	**MACGT quality score**	**HapMap Call**
NA12753	1		NA	GA	0.483052729	
NA12875	2	GG	1	gg	0.312964521	GG
NA12044	3	GA	0.978	ga	0.494773231	GA
NA06993	4	GA	0.9498	ga	0.263510167	GA
NA12716	5	GA	0.9791	ga	0.359583057	GA
NA12760	6	GG	0.9995	gg	0.397757272	GG
NA07348	7	GG	0.9991	gg	0.305182338	GG
NA12707	8	GA	0.9747	ga	0.297528849	GA
NA12156	9		NA	GG	0.316670507	
NA11992	10	GA	0.9733	ga	0.124570056	GA
NA12239	11	GA	0.973	ga	0.385517936	GA
NA12878	12	GG	0.9995	gg	0.367042629	GG
NA11993	13	GG	0.9995	gg	0.145559389	GG
NA12750	14	GA	0.991	ga	0.085654094	GA
NA12146	15	GA	0.9878	ga	0.141428767	GA
NA11839	16	GG	0.9999	gg	0.141717138	GG
NA12156.dup	17	GG	0.9998	gg	0.239054992	nn
NA11829	18		NA	GG	0.421247639	
NA12154	19	GA	0.9517	ga	0.18884581	GA
NA10856	20	GG	1	gg	0.090485415	GG
NA06985	21	GG	0.9999	gg	0.128018962	GG
NA11831	22	GA	0.9878	ga	0.088490108	GA
NA12003.dup	23	GG	0.9552	gg	0.124814319	nn
NA10859	24	GG	1	gg	0.156709707	GG
NA07056	25	GG	0.9992	gg	0.200663859	GG
NA07022	26	GG	0.9945	gg	0.355497569	GG
NA12762	27		NA	GA	0.364910857	
NA12874	28	GG	0.9998	gg	0.036568572	GG
NA12003	29	GG	0.9929	gg	0.213461562	GG
NA10835	30	GG	0.9979	gg	0.299058739	GG
NA11882	31	GG	0.9553	gg	0.270130038	GG
NA07034	32	GG	0.9999	gg	0.026593917	GG
NA12056	33	GA	0.9685	ga	0.064621731	GA
NA12145	34	GG	0.9999	gg	0.367421093	GG
NA07019	35	GG	0.9993	gg	0.37682943	GG
NA06991	36		NA	GG	0.424500489	
NA12761	37	GG	0.9999	gg	0.357369791	GG
NA06994	38	GG	0.9994	gg	0.378008332	GG
NA12864	39	GG	1	gg	0.183703928	GG
NA07055	40	GA	0.9882	ga	0.161888565	GA
NA10863	41	GG	0.9994	gg	0.445273998	GG
NA12763	42	GG	0.9991	gg	0.367618267	GG
NA10831	43	GG	0.9997	gg	0.361708135	GG
NA11840	44	GG	1	gg	0.239236288	GG

	MACGT discrepancy					
	LDA discrepancy					
NA	LDA output were not g					
XY	Manual calls are given					
		**1825443**				
**Coriell**	**Sample**	**LDA Call**	**LDA quality score**	**MACGT Call**	**MACGT quality score**	**HapMap Call**
NA10830	45	GG	1	gg	0.508305109	GG
NA11993.dup	46	GG	1	gg	0.33975004	nn
NA12751	47	GG	1	gg	0.002577949	GG
NA12814	48	GG	0.9999	gg	0.020263731	GG
NA10857	49	GA	0.9863	ga	0.416557175	GA
NA07357	50	GG	0.9959	gg	0.316195889	GG
NA07000	51	GA	0.9756	ga	0.469869602	GA
NA12802	52	GG	0.9878	gg	0.320825712	GG
NA10855	53	GG	0.8324	gg	0.111307195	GG
NA11995	54		NA	GG	0.180555627	
NA10839	55		NA	GG	0.156678877	
NA10847	56		NA	GA	0.396498075	
NA12006	57	GG	1	gg	0.257322936	GG
NA12144	58	GG	1	gg	0.365404333	GG
NA11832	59	GG	0.9819	gg	0.11233458	GG
NA12813	60	GG	1	gg	0.284706607	GG
blank	61	NN	0.5534	***NN***	***0***	nn
NA12891	62	GG	1	gg	0.286428114	GG
NA12717	63		NA	GG	0.275856211	
NA10861	64	GG	1	gg	0.001415412	GG
NA12004	65	GG	1	gg	0.061654739	GG
NA07048	66	GA	0.978	ga	0.349842275	GA
NA12801	67	GG	0.9997	gg	0.02962376	GG
NA11830	68	GG	1	gg	0.398160985	GG
NA12057	69	GG	1	gg	0.022905465	GG
NA11994	70		NA	GG	0.414878583	
NA12740	71	GA	0.9764	ga	0.493013627	GA
NA12005	72	GA	0.9877	ga	0.053563592	GA
NA07345	73	GG	0.9994	gg	0.167328261	GG
NA10854	74	GG	1	gg	0.206832205	GG
NA12872	75	GG	1	gg	0.294398041	GG
NA12865	76	GG	1	gg	0.220527067	GG
NA10860	77		NA	GA	0.281330463	
NA12873	78	GG	0.9941	gg	0.363938367	GG
NA12752	79	GG	1	gg	0.283047141	GG
NA10851	80	GA	0.9837	ga	0.597086964	GA
NA07029	81	GG	1	gg	0.315481467	GG
NA12248	82	GG	1	gg	0.411139833	GG
NA12155	83	GG	0.9998	gg	0.031636848	GG
NA10838	84	GG	0.9988	gg	0.047874306	GG
NA12812	85	GG	0.9997	gg	0.060575503	GG
NA12248.dup	86	GG	0.762	gg	0.061586051	nn
NA12043	87	GA	0.9739	ga	0.498683056	GA
NA12234	88	GG	0.9984	gg	0.116900714	GG

	MACGT discrepancy					
	LDA discrepancy					
NA	LDA output were not g					
XY	Manual calls are given					
		**1825443**				
**Coriell**	**Sample**	**LDA Call**	**LDA quality score**	**MACGT Call**	**MACGT quality score**	**HapMap Call**
NA12892	89	GG	1	gg	0.376561063	GG
NA10846	90	GG	1	gg	0.310662912	GG
NA06993.dup	91	GA	0.9787	ga	0.471646148	nn
NA12249	92	GG	1	gg	0.207243155	GG
NA11881	93	GG	0.999	gg	0.014636682	GG
NA12815	94	GG	0.9966	gg	0.004665585	GG
NA12264	95	GA	0.9825	ga	0.449971398	GA
NA18526	96	GG	0.9993	gg	0.390494395	GG
NA18562	97	GG	1	gg	0.356823397	GG
NA18545	98	GG	0.9957	gg	0.348313143	GG
NA18609	99	GG	0.9993	gg	0.373507912	GG
NA18566	100	GG	1	gg	0.391245156	GG
NA18621	101	GG	1	gg	0.415097974	GG
NA18577	102	GG	1	gg	0.285153509	GG
NA18635	103	GG	1	gg	0.413040619	GG
NA18524	104	GG	1	gg	0.487053048	GG
NA18537	105	GG	1	gg	0.309600286	GG
NA18572	106	GG	1	gg	0.064659277	GG
NA18552	107	GG	1	gg	0.223790681	GG
NA18563	108	GG	1	gg	0.295394793	GG
NA18594	109	GG	0.9999	gg	0.330537974	nn
NA18624	110	GG	0.9996	gg	0.580782153	GG
NA18592	111	GG	0.9961	gg	0.514052471	GG
NA18529	112	GG	0.9964	gg	0.470764165	GG
NA18603	113	GG	1	gg	0.311885013	nn
NA18547	114	GG	0.9998	gg	0.488620594	GG
NA18611	115	GG	1	gg	0.32071926	GG
NA18570	116	GG	0.9369	gg	0.349005131	GG
NA18622	117	GG	0.9988	gg	0.618346302	GG
NA18579	118	GG	0.9907	gg	0.060107076	GG
NA18636	119	GG	1	gg	0.414863881	GG
NA18558	120	GG	0.962	gg	0.162192791	GG
NA18540	121	GG	1	gg	0.252337221	GG
NA18609	122	GG	0.95	gg	0.312018267	nn
NA18555	123	GG	0.9997	gg	0.036383293	GG
NA18612	124	GG	0.9967	gg	0.375305945	GG
NA18573	125	GG	0.7818	gg	0.129277384	GG
NA18632	126	GG	1	gg	0.190847015	GG
NA18593	127	GG	0.9998	gg	0.274105095	GG
NA18532	128	GG	1	gg	0.193464815	GG
NA18605	129	GG	1	gg	0.418119828	GG
NA18550	130	GG	1	gg	0.380789013	GG
NA18603	131	GG	0.982	gg	0.536972967	GG
NA18571	132	GG	1	gg	0.463174514	GG

	MACGT discrepancy					
	LDA discrepancy					
NA	LDA output were not g					
XY	Manual calls are given					
		**1825443**				
**Coriell**	**Sample**	**LDA Call**	**LDA quality score**	**MACGT Call**	**MACGT quality score**	**HapMap Call**
NA18623	133	GG	0.9999	gg	0.148268131	GG
NA18582	134	GG	0.9999	gg	0.434185432	GG
NA18637	135	GG	0.9997	gg	0.392281895	GG
NA18561	136	GG	1	gg	0.105810833	GG
NA18542	137	GG	0.9998	gg	0.142803139	GG
NA18608	138	GG	0.9988	gg	0.435984525	GG
NA18564	139	GG	0.9999	gg	0.174305184	GG
NA18620	140	GG	0.9999	gg	0.128114276	GG
NA18576	141	GG	0.9998	gg	0.227670009	GG
NA18633	142	GG	0.9999	gg	0.142999403	GG
NA18594	143	GG	0.9999	gg	0.176643198	GG
NA18942	144	GG	1	gg	0.399564061	GG
NA18945	145	GG	1	gg	0.373512069	GG
NA18964	146	GG	0.9989	gg	0.351043331	GG
NA18961	147	GG	0.9621	gg	0.332212698	GG
NA18967	148	GG	1	gg	0.484672161	GG
NA18981	149	GG	1	gg	0.373987975	GG
NA18994	150	GG	1	gg	0.379549913	GG
NA18998	151	GG	1	gg	0.201128784	GG
NA18940	152	GG	1	gg	0.147641788	GG
NA18949	153	GG	1	gg	0.104524493	GG
NA18953	154	GG	1	gg	0.095022251	GG
NA18972	155	GG	0.995	gg	0.169919178	GG
NA18976	156	GG	1	gg	0.412488924	GG
NA18971	157	GG	0.9994	gg	0.32720202	GG
blank	158	NN	0.5346	*NN*	*0*	nn
NA19000	159	GG	0.9989	gg	0.491849532	GG
NA18951	160	GG	0.9998	gg	0.389281975	nn
NA18948	161	GG	0.9998	gg	0.353128165	GG
NA18968	162	GG	0.9994	gg	0.410194292	GG
NA18965	163	GG	1	gg	0.309973972	GG
NA18978	164	GG	0.9997	gg	0.385574044	GG
NA18974	165	GG	0.9996	gg	0.228061527	GG
NA18992	166	GG	1	gg	0.270528253	GG
NA19005	167	GG	0.9993	gg	0.480702906	GG
NA18943	168	GG	0.9927	gg	0.184309129	GG
NA18951	169	GG	0.9698	gg	0.188368071	GG
NA18959	170	GG	1	gg	0.135120855	GG
NA18973	171	GG	0.9998	gg	0.204056611	GG
NA18970	172	GG	0.9986	gg	0.360761599	GG
NA18987	173	GG	0.9996	gg	0.147995261	GG
NA18995	174	GG	1	gg	0.321793154	GG
NA18999	175	GG	1	gg	0.258643955	GG
NA18947	176	GG	0.9998	gg	0.082763055	GG

	MACGT discrepancy					
	LDA discrepancy					
NA	LDA output were not g					
XY	Manual calls are given					
		**1825443**				
**Coriell**	**Sample**	**LDA Call**	**LDA quality score**	**MACGT Call**	**MACGT quality score**	**HapMap Call**
NA18952	177	GG	0.9969	gg	0.192970894	GG
NA18969	178	GG	0.9977	gg	0.210064127	GG
NA18966	179	GG	0.9908	gg	0.326147491	GG
NA18980	180	GG	1	gg	0.154855253	GG
NA18990	181	GG	1	gg	0.225443739	GG
NA18997	182	GG	1	gg	0.181039375	GG
NA19007	183	GG	1	gg	0.111501858	GG
NA18944	184	GG	0.9998	gg	0.009057445	GG
NA18956	185	GG	0.9996	gg	0.24400315	GG
NA18960	186	GG	0.9973	gg	0.195697841	GG
NA18975	187	GG	0.9347	gg	0.077877823	GG
NA18995	188	GG	0.9998	gg	0.366841669	nn
NA18991	189	GG	0.9999	gg	0.4594339	GG
NA18996	190	GG	1	gg	0.095478727	nn
NA19003	191	GG	0.9994	gg	0.39801281	GG
NA18502	192	GG	0.8121	gg	0.016302068	GG
NA19153	193	GG	0.9983	gg	0.105444454	GG
NA18857	194	GG	1	gg	0.439153202	GG
NA19223	195	GG	0.9999	gg	0.07368849	GG
NA19201	196	GG	1	gg	0.132324051	GG
NA18504	197	GA	0.978	ga	0.230183553	GA
NA18870	198	GG	0.9806	gg	0.216958839	GG
NA18863	199	GG	0.9692	gg	0.115419982	GG
NA19145	200	GA	0.9722	ga	0.174458031	GA
NA19137	201	GA	0.9725	ga	0.393786737	GA
NA19238	202	GA	0.9731	*GA*	*0*	nn
NA18500	203	GG	1	gg	0.135840746	GG
NA19144	204	GA	0.9563	ga	0.162053942	GA
NA19203	205	GG	0.9999	gg	0.423682396	GG
NA19200	206	GG	0.999	gg	0.374826136	GG
NA18855	207	GG	0.9994	gg	0.050287745	GG
NA18505	208	GG	1	gg	0.072622952	GG
NA19202	209	GG	1	gg	0.007415844	GG
NA18501	210	GG	1	gg	0.181437927	GG
NA18861	211	GG	0.9999	gg	0.051000438	GG
NA19193	212	GG	1	gg	0.116422954	GG
NA19143	213	GG	0.9998	gg	0.120305929	GG
NA18517	214	GG	1	gg	0.212462491	GG
NA18862	215	GG	1	gg	0.194770113	GG
NA18856	216	GG	1	gg	0.09119657	nn
NA19239	217	GG	0.9993	gg	0.312231782	GG
NA19240	218	GG	0.9982	gg	0.507497457	GG
NA18856	219	GG	1	gg	0.260617016	GG
NA18503	220	GA	0.9896	ga	0.055899731	GA

	MACGT discrepancy					
	LDA discrepancy					
NA	LDA output were not g					
XY	Manual calls are given					
		**1825443**				
**Coriell**	**Sample**	**LDA Call**	**LDA quality score**	**MACGT Call**	**MACGT quality score**	**HapMap Call**
NA18871	221	GA	0.9865	ga	0.428813974	GA
NA19221	222	GG	0.9542	gg	0.381333991	GG
NA19209	223	GG	0.9984	gg	0.22404012	GG
NA19152	224	GG	0.9986	gg	0.341823028	GG
NA18515	225	GG	0.9975	gg	0.001851671	GG
NA19238	226	GA	0.9839	ga	0.561499881	GA
NA19154	227	GG	0.9967	gg	0.518537999	GG
NA19210	228		NA	AA	1	
NA19211	229	GA	0.9733	ga	0.532218609	GA
NA18862	230	GG	0.9799	gg	0.5122794	nn
NA18872	231	GG	1	gg	0.504768114	GG
NA19139	232	GA	0.9814	ga	0.612847954	GA
NA19222	233	GG	0.8616	gg	0.181209657	GG
NA19194	234	GG	0.9822	gg	0.57987634	GG
NA19138	235	GA	0.9732	ga	0.052656929	GA
NA19204	236	GG	0.9924	gg	0.311203835	GG
NA18516	237	GA	0.9779	ga	0.263994639	GA
NA19205	238	GG	0.9961	gg	0.288532921	GG
NA19192	239	GG	0.9999	gg	0.42015365	GG
NA18912	240	GG	0.9279	gg	0.328671208	nn
NA18508	241	GA	0.9826	ga	0.535783883	GA
NA19142	242	GG	0.93	gg	0.170697419	GG
NA18852	243	GG	0.9944	gg	0.323546764	GG
NA18507	244	GG	0.9999	gg	0.366085637	GG
blank	245	NN	0.5013	***NN***	***0***	nn
NA19101	246	GG	0.9995	gg	0.39676084	GG
NA19172	247	GA	0.9788	ga	0.355325525	GA
NA19160	248	GA	0.9767	ga	0.419743089	GA
NA19129	249	GG	0.9995	gg	0.380567253	GG
NA18913	250	GA	0.9803	ga	0.319175987	GA
NA19120	251	GG	0.9787	gg	0.041858621	GG
NA19159	252	GG	0.9993	gg	0.285053546	GG
NA18523	253	GG	0.9999	gg	0.27500499	GG
NA19102	254	GA	0.9685	***GA***	***0***	GA
NA19092	255	GG	0.9974	gg	0.537467801	GG
NA18521	256	GG	0.9907	gg	0.234063547	GG
NA18912	257	GG	0.9999	gg	0.298930699	GG
NA19206	258	GG	0.9998	gg	0.44742909	GG
NA19128	259	GG	0.9999	gg	0.374647558	GG
NA18859	260	GG	0.9987	gg	0.43780545	nn
NA19094	261	GG	1	gg	0.008554854	GG
NA19171	262	GG	1	gg	0.015201841	GG
NA18860	263	GA	0.982	ga	0.354804148	GA
NA18858	264	AA	0.8982	***AA***	***0***	AA

	MACGT discrepancy					
	LDA discrepancy					
NA	LDA output were not g					
XY	Manual calls are given					
		**1825443**				
**Coriell**	**Sample**	**LDA Call**	**LDA quality score**	**MACGT Call**	**MACGT quality score**	**HapMap Call**
NA19099	265	GA	0.9785	ga	0.438092669	GA
NA18506	266	GG	1	gg	0.374276083	GG
NA19131	267	AA	0.884	***AA***	***0***	AA
NA19116	268	GA	0.9791	ga	0.447759976	GA
NA19161	269	GA	0.9749	ga	0.581432225	GA
NA19140	270	GG	0.9979	gg	0.549906493	GG
NA19207	271	GA	0.9712	ga	0.151123686	GA
NA19141	272	GG	0.9992	gg	0.115272018	GG
NA18854	273	GG	0.9999	gg	0.49657558	GG
NA19098	274	GA	0.9815	ga	0.582158674	GA
NA18522	275	GA	0.9758	ga	0.446813951	GA
NA18914	276	GG	1	gg	0.070394178	GG
NA18853	277	GG	0.9998	gg	0.455448027	GG
NA18859	278	GG	0.9997	gg	0.449138968	GG
NA19100	279	GA	0.9796	ga	0.256461143	GA
NA19093	280	GG	0.9996	gg	0.055094938	GG
NA19132	281	GA	0.9732	ga	0.433362699	GA
NA19130	282	GG	1	gg	0.111496245	GG
NA19208	283	GA	0.9797	ga	0.323437978	GA
NA19127	284	GG	0.9967	gg	0.259018594	GG
NA19173	285	GA	0.969	ga	0.283684392	GA
NA19119	286	GG	0.999	gg	0.345501838	GG
NA19103	287	GA	0.9826	ga	0.191959831	GA
	Discrepancies	0		0		
	NNs	1		1		
	Automated Calls	275		268		
	Manual Calls	0		7		
	Concordant Calls	256		256		256

	MACGT discrepancy					
	LDA discrepancy					
NA	LDA output were not g					
XY	Manual calls are given					
		1891403				
**Coriell**	**Sample**	**LDA Call**	**LDA quality score**	**MACGT Call**	**MACGT quality score**	**HapMap Call**
NA12753	1		NA	TT	0.532928816	
NA12875	2	CT	0.9974	ct	0.231733719	CT
NA12044	3	CC	1	cc	0.154601331	CC
NA06993	4	TT	0.9985	tt	0.422823411	TT
NA12716	5	TT	0.9823	tt	0.343684697	TT
NA12760	6	TT	0.9947	tt	0.561761823	TT
NA07348	7	TT	0.9993	tt	0.438542664	TT
NA12707	8		NA	TT	0.124817729	
NA12156	9		NA	TT	0.501606706	
NA11992	10	TT	0.9795	tt	0.163279584	TT
NA12239	11	TT	0.9928	tt	0.099447353	TT
NA12878	12	CT	0.9997	ct	0.406848169	CT
NA11993	13	TT	0.9857	tt	0.042852811	TT
NA12750	14	TT	0.9821	tt	0.098132754	TT
NA12146	15	TT	0.9025	tt	0.103231517	TT
NA11839	16	TT	0.9446	tt	0.003773703	TT
NA12156.dup	17	TT	0.9998	tt	0.337790391	nn
NA11829	18		NA	TT	0.41751442	
NA12154	19	TT	0.9979	tt	0.425410103	TT
NA10856	20	TT	0.9988	tt	0.313484356	TT
NA06985	21	TT	0.9994	tt	0.262056783	TT
NA11831	22	TT	0.9619	tt	0.150570822	TT
NA12003.dup	23	TT	0.9085	tt	0.001320443	nn
NA10859	24	TT	0.9852	tt	0.316884033	TT
NA07056	25	TT	0.7973	tt	0.108581894	TT
NA07022	26	TT	0.9959	tt	0.453723172	TT
NA12762	27		NA	TT	0.28427598	
NA12874	28	TT	0.7617	*TT*	*0*	TT
NA12003	29	TT	0.9756	tt	0.060827003	TT
NA10835	30	TT	0.9998	tt	0.275534348	TT
NA11882	31	TT	0.9936	tt	0.274229625	TT
NA07034	32	TT	0.9966	tt	0.213951584	TT
NA12056	33	TT	0.9809	*TT*	*0*	TT
NA12145	34	CT	0.9997	ct	0.099898694	CT
NA07019	35	TT	0.9355	tt	0.196479995	TT
NA06991	36		NA	TT	0.293280626	
NA12761	37	TT	0.9819	tt	0.134701978	TT
NA06994	38	TT	0.9973	tt	0.273975969	TT
NA12864	39	TT	0.9955	tt	0.318092532	TT
NA07055	40	TT	0.9973	tt	0.487760615	TT
NA10863	41	TT	0.9906	tt	0.240267007	TT
NA12763	42	TT	0.9998	tt	0.320514647	TT
NA10831	43	TT	0.9992	tt	0.502865751	TT
NA11840	44	TT	0.9577	tt	0.128023738	TT

	MACGT discrepancy					
	LDA discrepancy					
NA	LDA output were not g					
XY	Manual calls are given					
		**1891403**				
**Coriell**	**Sample**	**LDA Call**	**LDA quality score**	**MACGT Call**	**MACGT quality score**	**HapMap Call**
NA10830	45	TT	0.9997	tt	0.033823392	TT
NA11993.dup	46	TT	0.9978	tt	0.431130788	nn
NA12751	47	TT	0.9997	tt	0.130863019	TT
NA12814	48	TT	0.9961	tt	0.098893506	TT
NA10857	49	CT	0.9999	ct	0.150443053	CT
NA07357	50	TT	0.9999	tt	0.490458457	TT
NA07000	51	TT	0.9982	tt	0.349670224	TT
NA12802	52	TT	0.9998	tt	0.26116483	TT
NA10855	53	TT	0.9987	tt	0.405366613	TT
NA11995	54		NA	TT	0.199900466	
NA10839	55	TT	0.985	tt	0.31588507	TT
NA10847	56	TT	1	tt	0.297153364	TT
NA12006	57	TT	0.9987	tt	0.346347136	TT
NA12144	58	CC	0.9985	cc	0.098100403	CT
NA11832	59	TT	0.9937	tt	0.016168687	TT
NA12813	60	CT	0.9972	ct	0.366947135	CT
blank	61	NN	0.5	*NN*	*0*	nn
NA12891	62		NA	CT	0.324039168	
NA12717	63		NA	TT	0.222465684	
NA10861	64	CC	0.9993	*CC*	*0*	CT
NA12004	65	TT	0.9996	tt	0.200967476	TT
NA07048	66	TT	0.9963	tt	0.095387399	TT
NA12801	67	CT	0.9898	ct	0.106084288	CT
NA11830	68	TT	0.9996	tt	0.067174691	TT
NA12057	69	TT	0.9993	tt	0.165358649	TT
NA11994	70		NA	CC	0.506336821	
NA12740	71	TT	0.999	tt	0.311710052	TT
NA12005	72	TT	0.8844	tt	0.118675928	TT
NA07345	73	TT	0.8226	tt	0.038031663	TT
NA10854	74	TT	0.8963	tt	0.151488381	TT
NA12872	75	TT	0.9991	tt	0.067903157	TT
NA12865	76	TT	0.9922	tt	0.171139376	TT
NA10860	77		NA	TT	0.251717787	
NA12873	78	TT	1	tt	0.483370283	TT
NA12752	79	TT	0.9988	tt	0.274174974	TT
NA10851	80	TT	0.9999	tt	0.299156736	TT
NA07029	81	TT	0.9989	tt	0.140233328	TT
NA12248	82	TT	0.9999	tt	0.183245072	TT
NA12155	83	TT	0.9974	tt	0.002484724	TT
NA10838	84	TT	0.9997	tt	0.205990942	TT
NA12812	85	TT	0.9967	tt	0.179246393	TT
NA12248.dup	86	TT	0.9953	tt	0.097235416	nn
NA12043	87	TT	0.9911	tt	0.07758036	TT
NA12234	88	CT	0.9947	ct	0.475726645	CT

	MACGT discrepancy					
	LDA discrepancy					
NA	LDA output were not g					
XY	Manual calls are given					
				1891403		
**Coriell**	**Sample**	**LDA Call**	**LDA quality score**	**MACGT Call**	**MACGT quality score**	**HapMap Call**
NA12892	89	TT	0.9991	tt	0.187736782	TT
NA10846	90	CT	0.9989	ct	0.430099633	CT
NA06993.dup	91	TT	0.9999	tt	0.305322893	nn
NA12249	92	TT	0.9686	tt	0.155105801	TT
NA11881	93	TT	0.9551	tt	0.273034149	TT
NA12815	94	TT	0.9916	tt	0.270540937	TT
NA12264	95	TT	0.9995	tt	0.236313497	TT
NA18526	96	TT	0.9998	tt	0.076204195	TT
NA18562	97	TT	0.9999	tt	0.434288616	TT
NA18545	98	TT	0.9978	tt	0.365067103	TT
NA18609	99	TT	0.9978	tt	0.251336886	TT
NA18566	100	TT	0.9993	tt	0.290104519	TT
NA18621	101	TT	0.9955	tt	0.277864128	TT
NA18577	102	TT	0.978	tt	0.130458838	TT
NA18635	103	CT	0.9995	ct	0.439991233	CT
NA18524	104	TT	1	tt	0.085752082	TT
NA18537	105	CT	0.9995	ct	0.431402471	CT
NA18572	106	TT	0.9907	tt	0.346313987	TT
NA18552	107	TT	0.9925	tt	0.098834189	TT
NA18563	108	TT	0.9829	tt	0.228535276	TT
NA18594	109	TT	0.9966	tt	0.431701479	nn
NA18624	110	TT	1	tt	0.12440106	TT
NA18592	111	TT	0.9998	tt	0.520211736	TT
NA18529	112	TT	0.9992	tt	0.598372623	TT
NA18603	113	TT	0.9996	tt	0.428781022	nn
NA18547	114		NA	CC	0.446050959	
NA18611	115	TT	0.9811	tt	0.274689001	TT
NA18570	116	TT	1	tt	0.206580949	TT
NA18622	117	TT	0.9985	tt	0.365526507	TT
NA18579	118	CT	0.9986	ct	0.178923768	CT
NA18636	119	TT	0.9779	tt	0.22591896	TT
NA18558	120	TT	0.9933	tt	0.305992466	TT
NA18540	121	CT	0.9949	ct	0.126402089	CT
NA18609	122	TT	0.9713	tt	0.033758406	nn
NA18555	123	TT	0.9939	tt	0.013832159	TT
NA18612	124	TT	0.9988	tt	0.070916122	TT
NA18573	125	TT	0.9879	tt	0.07714401	TT
NA18632	126	TT	0.9966	tt	0.337116722	TT
NA18593	127	TT	0.9843	tt	0.170117354	TT
NA18532	128	TT	0.9987	tt	0.457011132	TT
NA18605	129	TT	0.9968	tt	0.249102782	TT
NA18550	130	NN	0.7406	*TT*	*0*	TT
NA18603	131	TT	0.9971	tt	0.24738723	TT
NA18571	132	TT	0.9998	tt	0.267982099	TT

	MACGT discrepancy					
	LDA discrepancy					
NA	LDA output were not g					
XY	Manual calls are given					
		**1891403**				
**Coriell**	**Sample**	**LDA Call**	**LDA quality score**	**MACGT Call**	**MACGT quality score**	**HapMap Call**
NA18623	133	TT	0.9991	tt	0.259695683	TT
NA18582	134	TT	1	***TT***	***0***	TT
NA18637	135	TT	1	***TT***	***0***	TT
NA18561	136	TT	0.9963	tt	0.184630177	TT
NA18542	137	TT	1	tt	0.271629626	TT
NA18608	138	TT	0.9994	tt	0.329254246	TT
NA18564	139	TT	0.9914	tt	0.034619783	TT
NA18620	140	TT	0.9973	tt	0.314010974	TT
NA18576	141	CT	0.9973	ct	0.569861689	CT
NA18633	142	TT	0.9975	tt	0.329361433	TT
NA18594	143	TT	0.9972	tt	0.312125563	TT
NA18942	144	TT	0.9983	tt	0.411994911	TT
NA18945	145	TT	0.9994	tt	0.491935008	TT
NA18964	146	TT	0.9993	tt	0.109413438	TT
NA18961	147	TT	0.8425	tt	0.005868082	TT
NA18967	148	TT	0.9997	tt	0.537836446	TT
NA18981	149	TT	1	tt	0.478733821	TT
NA18994	150	TT	0.9989	tt	0.174245654	TT
NA18998	151	TT	0.9594	tt	0.002944128	TT
NA18940	152	TT	0.9869	tt	0.039173643	TT
NA18949	153	TT	0.9926	tt	0.178726587	TT
NA18953	154	TT	0.9855	tt	0.04556163	TT
NA18972	155	TT	0.9974	tt	0.029420675	TT
NA18976	156	TT	0.9988	tt	0.291349191	TT
NA18971	157	TT	0.9949	tt	0.08399499	TT
blank	158	NN	0.621	***NN***	***0***	nn
NA19000	159	CT	0.9991	ct	0.122531785	CT
NA18951	160	TT	0.9993	***TT***	***0***	nn
NA18948	161	TT	0.9998	***TT***	***0***	TT
NA18968	162	TT	0.9996	tt	0.249058194	TT
NA18965	163	CT	0.9957	ct	0.092973134	CT
NA18978	164	TT	1	***TT***	***0***	TT
NA18974	165	CT	0.9984	ct	0.047729137	CT
NA18992	166	TT	0.9975	tt	0.088578815	TT
NA19005	167	TT	1	tt	0.347901206	TT
NA18943	168	CC	0.9892	cc	0.033627979	CT
NA18951	169	TT	0.9977	tt	0.41522057	TT
NA18959	170	TT	1	tt	0.530912144	TT
NA18973	171	TT	0.9978	tt	0.507462661	TT
NA18970	172	TT	0.998	tt	0.506283279	TT
NA18987	173	TT	1	tt	0.098054637	TT
NA18995	174		NA	CT	0.538900973	
NA18999	175	TT	1	tt	0.235619158	TT
NA18947	176	TT	0.9995	tt	0.116188225	TT

	MACGT discrepancy					
	LDA discrepancy					
NA	LDA output were not g					
XY	Manual calls are given					
		**1891403**				
**Coriell**	**Sample**	**LDA Call**	**LDA quality score**	**MACGT Call**	**MACGT quality score**	**HapMap Call**
NA18952	177	TT	1	tt	0.493365494	TT
NA18969	178	TT	1	tt	0.06183328	TT
NA18966	179		NA	CT	0.451852636	
NA18980	180	TT	0.9999	tt	0.529538653	TT
NA18990	181	TT	1	tt	0.054929718	TT
NA18997	182	TT	1	tt	0.436777249	TT
NA19007	183	TT	0.9996	tt	0.060459775	TT
NA18944	184	TT	0.8857	tt	0.064996827	TT
NA18956	185	TT	0.9927	tt	0.434128457	TT
NA18960	186	TT	0.9968	tt	0.428517253	TT
NA18975	187		NA	CT	0.487547673	
NA18995	188		NA	CT	0.554371304	
NA18991	189	TT	0.9892	tt	0.44562621	TT
NA18996	190	TT	0.994	tt	0.476095133	nn
NA19003	191	TT	0.9972	tt	0.339094828	TT
NA18502	192	TT	0.9923	tt	0.266980998	TT
NA19153	193	TT	0.9755	tt	0.413536198	TT
NA18857	194	TT	0.9996	tt	0.327830953	TT
NA19223	195	CT	0.9834	ct	0.194008997	CT
NA19201	196	TT	0.9904	tt	0.187503586	TT
NA18504	197	TT	0.9174	tt	0.284906279	TT
NA18870	198	TT	0.9957	tt	0.3814327	TT
NA18863	199	CT	0.999	ct	0.422781135	CT
NA19145	200	TT	0.9996	tt	0.068980063	TT
NA19137	201		NA	CT	0.356971219	
NA19238	202	TT	0.994	tt	0.198009069	nn
NA18500	203		NA	CT	0.181102396	
NA19144	204	TT	0.9949	tt	0.05328583	TT
NA19203	205	CT	0.9976	ct	0.287930875	CT
NA19200	206	CT	0.9972	ct	0.372496045	CT
NA18855	207	TT	0.9963	tt	0.515952025	TT
NA18505	208	TT	0.9952	tt	0.386760693	TT
NA19202	209	CT	0.9986	ct	0.275811879	CT
NA18501	210	CT	0.9978	ct	0.103560493	CT
NA18861	211	TT	0.997	tt	0.003948238	TT
NA19193	212	TT	1	tt	0.054162837	TT
NA19143	213	TT	0.9968	tt	0.119689455	TT
NA18517	214	TT	0.9978	tt	0.032201965	TT
NA18862	215		NA	CT	0.386051173	
NA18856	216	TT	0.9984	tt	0.254287317	nn
NA19239	217	TT	0.9973	tt	0.016793052	TT
NA19240	218	TT	0.9985	tt	0.324026467	TT
NA18856	219	TT	0.9985	tt	0.175790415	TT
NA18503	220	TT	0.9995	tt	0.204023485	TT

	MACGT discrepancy					
	LDA discrepancy					
NA	LDA output were not g					
XY	Manual calls are given					
		**1891403**				
**Coriell**	**Sample**	**LDA Call**	**LDA quality score**	**MACGT Call**	**MACGT quality score**	**HapMap Call**
NA18871	221	CT	0.9999	ct	0.185606694	CT
NA19221	222		NA	CC	0.348660462	
NA19209	223	CT	0.9997	ct	0.028556871	CT
NA19152	224	TT	1	tt	0.267241921	TT
NA18515	225	TT	0.9973	tt	0.502586202	TT
NA19238	226	TT	0.9672	***TT***	***6.08E-04***	TT
NA19154	227	TT	0.998	tt	0.390513535	TT
NA19210	228	TT	0.9987	tt	0.03849661	TT
NA19211	229	CT	0.7612	ct	0.014947597	CT
NA18862	230	CT	0.9948	ct	0.411156826	nn
NA18872	231	TT	0.9991	tt	0.24200875	TT
NA19139	232	CT	0.999	ct	0.47847252	CT
NA19222	233	CT	0.9965	ct	0.051770775	CT
NA19194	234	TT	0.9985	tt	0.26558444	TT
NA19138	235		NA	CC	0.276360387	
NA19204	236	CT	0.9937	ct	0.061203519	CT
NA18516	237	TT	0.9998	tt	0.187664004	TT
NA19205	238	CT	0.9993	ct	0.428571089	CT
NA19192	239	TT	0.9957	tt	0.080016733	TT
NA18912	240	TT	0.9714	tt	0.124345441	nn
NA18508	241	TT	0.9982	tt	0.043967313	TT
NA19142	242	TT	0.9846	tt	0.243182549	TT
NA18852	243	TT	0.9988	tt	0.650799865	TT
NA18507	244	TT	0.9928	tt	0.128696558	TT
blank	245	NN	0.6709	***NN***	***0***	nn
NA19101	246	TT	0.9985	tt	0.071386515	TT
NA19172	247	CT	0.9988	ct	0.304649212	CT
NA19160	248	CT	0.998	ct	0.309146869	CT
NA19129	249	CT	0.9995	ct	0.160390513	CT
NA18913	250	TT	0.9969	tt	0.001326177	TT
NA19120	251	TT	0.9913	tt	0.005869375	TT
NA19159	252	CT	0.999	ct	0.130741962	CT
NA18523	253	TT	0.9995	tt	0.393314326	TT
NA19102	254	CT	0.9989	ct	0.161160536	CT
NA19092	255	CT	0.9976	ct	0.211530408	CT
NA18521	256	CT	0.9969	ct	0.203811826	CT
NA18912	257	TT	0.9317	tt	0.03729311	TT
NA19206	258	TT	0.9974	tt	0.184414743	TT
NA19128	259	CT	0.9986	ct	0.102101478	CT
NA18859	260	TT	0.9998	tt	0.41488473	nn
NA19094	261	CT	0.998	ct	0.265520172	CT
NA19171	262	TT	0.9978	tt	0.556087289	TT
NA18860	263	CT	0.9984	ct	0.195505994	CT
NA18858	264	CT	0.9998	ct	0.09618852	CT

	MACGT discrepancy					
	LDA discrepancy					
NA	LDA output were not g					
XY	Manual calls are given					
		1891403				
**Coriell**	**Sample**	**LDA Call**	**LDA quality score**	**MACGT Call**	**MACGT quality score**	**HapMap Call**
NA19099	265	CT	0.9976	ct	0.184753214	CT
NA18506	266	TT	0.7578	***TT***	***5.16E-04***	TT
NA19131	267	TT	0.9985	tt	0.084121612	TT
NA19116	268	TT	0.9998	tt	0.073501977	TT
NA19161	269	CT	0.9976	ct	0.239774632	CT
NA19140	270	TT	1	tt	0.004070146	TT
NA19207	271	TT	1	tt	0.02646087	TT
NA19141	272	CT	0.9996	ct	0.190901607	CT
NA18854	273	TT	1	tt	0.001362153	TT
NA19098	274	TT	1	***TT***	***0***	TT
NA18522	275	CT	0.9998	ct	0.1068028	CT
NA18914	276	TT	0.9963	tt	0.022973018	TT
NA18853	277	TT	0.9961	***TT***	***0***	TT
NA18859	278	TT	0.9999	tt	0.001417062	TT
NA19100	279	CT	0.9985	ct	0.07218345	CT
NA19093	280	CT	0.9996	ct	0.237244372	CT
NA19132	281	TT	0.9993	tt	0.020525832	TT
NA19130	282	CT	0.9997	ct	0.161913337	CT
NA19208	283	TT	0.9994	tt	0.360570045	TT
NA19127	284	TT	0.9988	tt	0.052044174	TT
NA19173	285	CT	0.998	ct	0.237998728	CT
NA19119	286	TT	1	tt	0.043085573	TT
NA19103	287	CT	0.9991	ct	0.160203543	CT
	Discrepancies	3		3		
	NNs	2		1		
	Automated Calls	266		250		
	Manual Calls	0		16		
	Concordant Calls	244		245		248

	MACGT discrepancy					
	LDA discrepancy					
NA	LDA output were not g					
XY	Manual calls are given					
		**2071748**				
**Coriell**	**Sample**	**LDA Call**	**LDA quality score**	**MACGT Call**	**MACGT quality score**	**HapMap Call**
NA12753	1		NA	AG	0.434776707	
NA12875	2	AG	0.8644	ag	0.061807599	AG
NA12044	3	AA	0.9958	aa	0.44952157	AA
NA06993	4	GG	0.9618	gg	0.078477243	GG
NA12716	5	AG	0.858	ag	0.059968246	AG
NA12760	6	AG	0.8326	ag	0.108385794	AG
NA07348	7	GG	0.9823	gg	0.307606206	GG
NA12707	8	AG	0.8034	ag	0.070202721	AG
NA12156	9		NA	AA	0.296876292	
NA11992	10	AG	0.8676	ag	0.071505184	AG
NA12239	11	AA	0.8566	aa	0.302111838	AA
NA12878	12	AG	0.8659	ag	0.038601585	AG
NA11993	13	GG	0.9342	gg	0.02813944	GG
NA12750	14	AA	0.9435	aa	0.11687063	AA
NA12146	15	AG	0.8219	ag	0.211576963	AG
NA11839	16	AG	0.8825	***AG***	***0***	AG
NA12156.dup	17	AA	0.9724	aa	0.320484013	nn
NA11829	18		NA	AG	0.353312841	
NA12154	19	AG	0.8146	ag	0.03302923	AG
NA10856	20	AA	0.9519	aa	0.385510505	AA
NA06985	21	GG	0.9909	gg	0.286386006	GG
NA11831	22	AG	0.867	ag	0.329638561	AG
NA12003.dup	23	GG	0.9172	***GG***	***0***	nn
NA10859	24	NN	0.7398	gg	0.122772379	GG
NA07056	25	GG	0.9202	gg	0.094867643	GG
NA07022	26	GG	0.8932	gg	0.148431612	GG
NA12762	27		NA	GG	0.149460245	
NA12874	28	GG	0.965	***GG***	***0***	GG
NA12003	29	GG	0.7547	gg	0.152004959	GG
NA10835	30	AG	0.7725	ag	0.054622729	AG
NA11882	31	AG	0.8268	ag	0.177817747	AG
NA07034	32	AA	0.9546	aa	0.438891169	AA
NA12056	33	GG	0.9841	gg	0.241201042	GG
NA12145	34	AA	0.954	aa	0.319758294	AA
NA07019	35	GG	0.9292	gg	0.172553304	GG
NA06991	36		NA	GG	0.344324195	
NA12761	37	AG	0.9067	ag	0.333072928	AG
NA06994	38	AG	0.8773	ag	0.139246726	AG
NA12864	39	GG	0.9435	gg	0.20060464	GG
NA07055	40	GG	0.8845	gg	0.311586981	GG
NA10863	41		NA	AG	0.390856835	
NA12763	42	AG	0.8614	ag	0.301107059	AG
NA10831	43	AG	0.8768	ag	0.197998236	AG
NA11840	44	AG	0.8461	ag	0.268896418	AG

	MACGT discrepancy					
	LDA discrepancy					
NA	LDA output were not g					
XY	Manual calls are given					
		**2071748**				
**Coriell**	**Sample**	**LDA Call**	**LDA quality score**	**MACGT Call**	**MACGT quality score**	**HapMap Call**
NA10830	45	AA	0.9999	aa	0.565027932	AA
NA11993.dup	46	GG	0.9745	gg	0.330342017	nn
NA12751	47	AG	0.8794	ag	0.132694419	AG
NA12814	48	AG	0.8507	ag	0.065308627	AG
NA10857	49	AG	0.8742	ag	0.246586178	AG
NA07357	50	GG	0.9428	gg	0.468452902	GG
NA07000	51	GG	0.9419	gg	0.098801888	GG
NA12802	52	AG	0.9003	ag	0.080343291	AG
NA10855	53	AG	0.899	ag	0.321316188	AG
NA11995	54		NA	GG	0.244273832	
NA10839	55	GG	0.9226	gg	0.295133188	GG
NA10847	56	AG	0.8269	ag	0.062053159	AG
NA12006	57	GG	0.9485	gg	0.097883737	GG
NA12144	58	AG	0.8378	ag	0.231429785	AG
NA11832	59	GG	0.9661	gg	0.285202211	GG
NA12813	60	AA	0.9909	aa	0.266748041	AA
blank	61	AA	0.9872	aa	0.266861893	nn
NA12891	62	GG	0.957	gg	0.481008963	GG
NA12717	63		NA	GG	0.407033129	
NA10861	64		NA	GG	0.346240673	
NA12004	65	AG	0.7897	ag	0.008320029	AG
NA07048	66	AG	0.8359	ag	0.133440565	AG
NA12801	67		NA	AA	0.238263637	
NA11830	68	AA	0.9571	aa	0.016716293	AA
NA12057	69	AG	0.8	ag	0.009102565	AG
NA11994	70		NA	GG	0.314110413	
NA12740	71	AA	0.9363	aa	0.483889859	AA
NA12005	72	AG	0.9043	***AG***	***0***	AG
NA07345	73	GG	0.7762	gg	0.013127753	GG
NA10854	74	AG	0.8697	ag	0.445025944	AG
NA12872	75	GG	0.9717	gg	0.379029247	GG
NA12865	76	AG	0.8794	ag	0.193385837	AG
NA10860	77		NA	GG	0.179116076	
NA12873	78	AG	0.8749	ag	0.00460028	AG
NA12752	79	AA	0.9745	aa	0.457408572	AA
NA10851	80	GG	0.9685	gg	0.362930569	GG
NA07029	81	GG	0.9667	gg	0.450375571	GG
NA12248	82	AA	0.9693	aa	0.293049523	AA
NA12155	83	AG	0.8556	ag	0.007878766	AG
NA10838	84	GG	0.9846	gg	0.316577906	GG
NA12812	85	AG	0.8284	ag	0.082560147	AG
NA12248.dup	86	AA	0.9315	aa	0.4749286	nn
NA12043	87	GG	0.9833	***GG***	***0***	GG
NA12234	88	GG	0.9842	***GG***	***6.06E-04***	GG

	MACGT discrepancy					
	LDA discrepancy					
NA	LDA output were not g					
XY	Manual calls are given					
				2071748		
**Coriell**	**Sample**	**LDA Call**	**LDA quality score**	**MACGT Call**	**MACGT quality score**	**HapMap Call**
NA12892	89	AA	0.9482	aa	0.416241753	AA
NA10846	90	AG	0.8832	ag	0.175763685	AG
NA06993.dup	91	GG	0.9546	gg	0.28201455	nn
NA12249	92	GG	0.9327	gg	0.190316994	GG
NA11881	93	GG	0.9392	gg	0.083898377	GG
NA12815	94	GG	0.9693	gg	0.054999811	GG
NA12264	95	AG	0.8687	ag	0.184099893	AG
NA18526	96	AG	0.8661	ag	0.213821604	AG
NA18562	97	AG	0.7963	ag	0.059440163	AG
NA18545	98	GG	0.9425	gg	0.276822807	GG
NA18609	99	GG	0.9633	gg	0.426435956	GG
NA18566	100	AG	0.8372	ag	0.129125489	AG
NA18621	101	GG	0.9618	gg	0.298497093	GG
NA18577	102	GG	0.9681	gg	0.04738431	GG
NA18635	103	AG	0.8243	ag	0.226781661	AG
NA18524	104	AG	0.8428	ag	0.005378269	AG
NA18537	105	AA	0.983	aa	0.274344331	AA
NA18572	106	GG	0.9772	gg	0.383458968	GG
NA18552	107	AA	0.9749	aa	0.264278663	AA
NA18563	108	GG	0.9719	gg	0.06960399	GG
NA18594	109	AG	0.8327	ag	0.165814702	nn
NA18624	110	AG	0.856	ag	0.32900539	AG
NA18592	111	AA	0.9961	aa	0.570534722	AA
NA18529	112	AA	0.9568	aa	0.540867669	AA
NA18603	113	GG	0.9498	gg	0.394759291	nn
NA18547	114	AG	0.8343	ag	0.102395254	AG
NA18611	115	AG	0.8685	ag	0.031599968	AG
NA18570	116	NN	0.7174	gg	0.137088515	GG
NA18622	117	AA	0.9053	aa	0.443572456	AA
NA18579	118	AG	0.8561	ag	0.238375667	AG
NA18636	119	AG	0.8129	ag	0.156649098	AG
NA18558	120	AG	0.842	ag	0.066747314	AG
NA18540	121	NN	0.7272	gg	0.003052289	GG
NA18609	122	NN	0.7271	gg	0.009338243	nn
NA18555	123	AG	0.83	*AG*	*0*	AG
NA18612	124	GG	0.9788	gg	0.269464549	GG
NA18573	125	AA	0.8176	aa	0.262510607	AA
NA18632	126	GG	0.9649	gg	0.0656286	GG
NA18593	127	AG	0.8546	ag	0.011834668	AG
NA18532	128	AG	0.8309	ag	0.014629019	AG
NA18605	129	AA	0.989	aa	0.041204363	AA
NA18550	130	GG	0.9726	gg	0.014939195	GG
NA18603	131	GG	0.9646	gg	0.430331126	GG
NA18571	132	GG	0.9887	gg	0.038273133	GG

	MACGT discrepancy					
	LDA discrepancy					
NA	LDA output were not g					
XY	Manual calls are given					
				2071748		
**Coriell**	**Sample**	**LDA Call**	**LDA quality score**	**MACGT Call**	**MACGT quality score**	**HapMap Call**
NA18623	133	GG	0.977	***GG***	***0***	GG
NA18582	134		NA	AA	0.680699484	
NA18637	135	AA	0.9998	aa	0.420686327	AA
NA18561	136	GG	0.9657	***GG***	***0***	GG
NA18542	137	AG	0.7585	***AG***	***0***	AG
NA18608	138	GG	0.959	gg	0.169808609	GG
NA18564	139	AG	0.7843	ag	0.009273761	AG
NA18620	140	AG	0.8139	ag	0.066809713	AG
NA18576	141		NA	AA	0.458141321	
NA18633	142		NA	AA	0.451481032	
NA18594	143	AG	0.8224	***AG***	***5.08E-04***	AG
NA18942	144	AA	0.7896	aa	0.044742944	AA
NA18945	145	GG	0.9777	gg	0.38564712	GG
NA18964	146	AA	0.9935	aa	0.40857741	AA
NA18961	147	AG	0.8786	ag	0.002624672	AG
NA18967	148	GG	0.9796	gg	0.349809528	GG
NA18981	149	GG	0.9803	gg	0.455416314	GG
NA18994	150	AG	0.8668	***AG***	***0***	AG
NA18998	151	AG	0.837	ag	0.007169241	AG
NA18940	152	GG	0.9456	gg	0.133040791	GG
NA18949	153	AG	0.8239	ag	0.068545325	AG
NA18953	154	AG	0.8188	***AG***	***0***	AG
NA18972	155	AG	0.7895	ag	0.082516514	AG
NA18976	156	AG	0.8362	ag	0.150581811	AG
NA18971	157	AA	0.9778	aa	0.364111833	AA
blank	158	NN	0.4758	***NN***	***0***	nn
NA19000	159	NN	0.6471	ag	0.02276762	AG
NA18951	160	NN	0.5808	ag	0.008014896	nn
NA18948	161	NN	0.6618	***AG***	***6.47E-04***	AG
NA18968	162	AG	0.7873	ag	0.005977003	AG
NA18965	163	GG	0.9099	***GG***	***0***	GG
NA18978	164	AA	0.9987	aa	0.324839882	AA
NA18974	165	NN	0.6177	***AG***	***0***	AG
NA18992	166	NN	0.6106	***AG***	***0***	AG
NA19005	167	AG	0.9018	ag	0.196397241	AG
NA18943	168	AG	0.8761	ag	0.133798013	AG
NA18951	169	AG	0.824	ag	0.023658641	AG
NA18959	170	GG	0.9435	gg	0.21293121	GG
NA18973	171	AA	0.9889	aa	0.412726558	AA
NA18970	172	GG	0.9597	gg	0.351160753	GG
NA18987	173	AA	0.9801	aa	0.30302955	AA
NA18995	174	AA	0.9964	aa	0.484892574	AA
NA18999	175	AG	0.8033	ag	0.012397378	AG
NA18947	176	GG	0.9688	***GG***	***0***	GG

	MACGT discrepancy					
	LDA discrepancy					
NA	LDA output were not g					
XY	Manual calls are given					
		2071748				
**Coriell**	**Sample**	**LDA Call**	**LDA quality score**	**MACGT Call**	**MACGT quality score**	**HapMap Call**
NA18952	177	AG	0.8179	ag	0.153172843	AG
NA18969	178	GG	0.9728	*gg*	*9.89E-04*	GG
NA18966	179	AA	0.9882	aa	0.485745088	AA
NA18980	180	GG	0.9577	*GG*	*0*	GG
NA18990	181	AG	0.8327	*AG*	*0*	AG
NA18997	182	AA	0.9859	aa	0.511538078	AA
NA19007	183	AG	0.8214	ag	0.028571294	AG
NA18944	184	GG	0.9321	*GG*	*0*	GG
NA18956	185	AG	0.8589	ag	0.140054123	AG
NA18960	186	GG	0.9533	gg	0.417146072	GG
NA18975	187	GG	0.8989	gg	0.059159394	GG
NA18995	188	AA	0.969	aa	0.013117319	nn
NA18991	189	GG	0.9667	gg	0.295059764	GG
NA18996	190	AG	0.8242	ag	0.003350763	nn
NA19003	191	AG	0.8514	ag	0.076397872	AG
NA18502	192	AA	0.9739	aa	0.633477981	AA
NA19153	193	AG	0.8592	ag	0.246857454	AG
NA18857	194	GG	0.9699	gg	0.155406041	GG
NA19223	195	AG	0.8305	ag	0.041574223	AG
NA19201	196	AA	0.9906	aa	0.182697707	AA
NA18504	197	AA	0.9934	aa	0.036516656	AA
NA18870	198	AG	0.8482	ag	0.132571765	AG
NA18863	199	AA	0.9712	aa	0.425177382	AA
NA19145	200	AG	0.8495	ag	0.079471595	AG
NA19137	201	AA	0.9593	aa	0.485812207	AA
NA19238	202	AA	0.9735	aa	0.054826453	nn
NA18500	203	AA	0.8747	aa	0.037064657	AA
NA19144	204	AG	0.8325	*AG*	*0*	AG
NA19203	205	AA	0.9728	aa	0.417482724	AA
NA19200	206	AG	0.8786	ag	0.040198983	AG
NA18855	207	AG	0.8704	ag	0.073181329	AG
NA18505	208	AG	0.8451	ag	0.05427731	AG
NA19202	209	AA	0.9754	aa	0.381717087	AA
NA18501	210	AA	0.9368	aa	0.26788282	AA
NA18861	211	AA	0.9156	aa	0.268440799	AA
NA19193	212	AA	0.9273	aa	0.237534112	AA
NA19143	213	AA	0.9212	aa	0.233622274	AA
NA18517	214	AG	0.7961	*AG*	*0*	AG
NA18862	215	AA	0.9572	aa	0.30837401	AA
NA18856	216	GG	0.9343	*GG*	*0*	nn
NA19239	217	AG	0.8168	ag	0.097698218	AG
NA19240	218	AA	0.9674	aa	0.364509884	AA
NA18856	219	GG	0.9374	gg	0.011675202	GG
NA18503	220	AA	0.9563	aa	0.403816902	AA

	MACGT discrepancy					
	LDA discrepancy					
NA	LDA output were not g					
XY	Manual calls are given					
		2071748				
**Coriell**	**Sample**	**LDA Call**	**LDA quality score**	**MACGT Call**	**MACGT quality score**	**HapMap Call**
NA18871	221	GG	0.8729	***GG***	***6.16E-04***	GG
NA19221	222	AA	0.9282	aa	0.481975285	AA
NA19209	223	AG	0.8776	ag	0.285478776	AG
NA19152	224	AG	0.8295	ag	0.124661541	AG
NA18515	225	AG	0.8665	ag	0.063105789	AG
NA19238	226	AA	0.9596	aa	0.476065507	AA
NA19154	227	GG	0.9413	***GG***	***0***	GG
NA19210	228	NN	0.6874	gg	0.029345199	GG
NA19211	229	AG	0.7895	ag	0.046394523	AG
NA18862	230	AA	0.9479	aa	0.321589766	nn
NA18872	231	GG	0.9644	gg	0.063144119	GG
NA19139	232	AA	0.9291	aa	0.625514088	AA
NA19222	233	AA	0.9321	aa	0.526989033	AA
NA19194	234	AA	0.9521	aa	0.55916764	AA
NA19138	235	AG	0.8336	ag	0.019317162	AG
NA19204	236	AA	0.9891	aa	0.38200852	AA
NA18516	237	AG	0.8434	ag	0.143131638	AG
NA19205	238	AA	0.8746	aa	0.530969552	AA
NA19192	239	AA	0.9446	aa	0.425362343	AA
NA18912	240	AA	0.9293	aa	0.482739179	nn
NA18508	241	GG	0.9287	gg	0.259649989	GG
NA19142	242	GG	0.8354	gg	0.121820167	GG
NA18852	243	AA	0.9538	aa	0.289250841	AA
NA18507	244	AA	0.9654	aa	0.3342208	AA
blank	245	NN	0.6417	***NN***	***0***	nn
NA19101	246	AA	0.8993	aa	0.468179111	AA
NA19172	247	AG	0.8543	ag	0.062344379	AG
NA19160	248	AA	0.8832	aa	0.294665249	AA
NA19129	249	AG	0.848	ag	0.020618216	AG
NA18913	250	AG	0.8742	ag	0.071625079	AG
NA19120	251	AG	0.7684	***AG***	***0***	AG
NA19159	252	AA	0.8536	aa	0.321489315	AA
NA18523	253	GG	0.9651	gg	0.239919154	GG
NA19102	254	AA	0.8809	aa	0.04490939	AA
NA19092	255	AA	0.9581	aa	0.478940549	AA
NA18521	256	AG	0.8591	ag	0.137245191	AG
NA18912	257	AA	0.9878	aa	0.293517759	AA
NA19206	258	AA	0.9737	aa	0.359208939	AA
NA19128	259	GG	0.8725	gg	0.375490961	GG
NA18859	260	AG	0.8392	ag	0.17903772	nn
NA19094	261	AG	0.8501	ag	0.416947876	AG
NA19171	262	AG	0.8416	ag	0.123682792	AG
NA18860	263	AG	0.856	ag	0.251856259	AG
NA18858	264	GG	0.9762	gg	0.460667353	GG

	MACGT discrepancy					
	LDA discrepancy					
NA	LDA output were not g					
XY	Manual calls are given					
		2071748				
**Coriell**	**Sample**	**LDA Call**	**LDA quality score**	**MACGT Call**	**MACGT quality score**	**HapMap Call**
NA19099	265	AG	0.8385	ag	0.157400842	AG
NA18506	266	NN	0.6188	ag	0.204748697	AG
NA19131	267	AA	0.9997	aa	0.495238059	AA
NA19116	268	AG	0.8383	ag	0.135843895	AG
NA19161	269	AA	0.9945	aa	0.328362439	AA
NA19140	270	NN	0.6138	ag	0.172973079	AG
NA19207	271	AG	0.8141	ag	0.0563251	AG
NA19141	272	NN	0.6144	ag	0.031356851	AG
NA18854	273	AA	0.9997	aa	0.385361274	AA
NA19098	274	AG	0.8437	ag	0.141518513	AG
NA18522	275	AG	0.8448	ag	0.05914507	AG
NA18914	276	AA	0.9372	aa	0.270300065	AA
NA18853	277	AA	0.9857	aa	0.486758546	AA
NA18859	278	AG	0.8146	ag	0.002075865	AG
NA19100	279	AG	0.8574	ag	0.003913695	AG
NA19093	280	AG	0.8434	ag	0.130999961	AG
NA19132	281	AA	0.9792	aa	0.418272943	AA
NA19130	282	AA	0.9978	aa	0.379672137	AA
NA19208	283	NN	0.7356	ag	0.125835537	AG
NA19127	284	AA	0.9493	aa	0.176096809	AA
NA19173	285	GG	0.9472	gg	0.01129914	GG
NA19119	286	AG	0.851	ag	0.220410471	AG
NA19103	287	AA	0.9995	aa	0.083438254	AA
	Discrepancies	0		0		
	NNs	14		0		
	Automated Calls	272		242		
	Manual Calls	0		30		
	Concordant Calls	241		253		253

	MACGT discrepancy					
	LDA discrepancy					
NA	LDA output were not g					
XY	Manual calls are given					
		2084851				
**Coriell**	**Sample**	**LDA Call**	**LDA quality score**	**MACGT Call**	**MACGT quality score**	**HapMap Call**
NA12753	1		NA	GA	0.430612538	
NA12875	2	AA	0.9916	aa	0.452080285	AA
NA12044	3	AA	0.9681	aa	0.384332743	AA
NA06993	4	AA	0.9885	aa	0.160692459	AA
NA12716	5	GA	0.9965	ga	0.343370949	GA
NA12760	6	GG	0.9902	gg	0.262188656	GG
NA07348	7	GA	0.9844	ga	0.295544313	GA
NA12707	8	GG	1	gg	0.368908528	GG
NA12156	9		NA	GG	0.434951129	
NA11992	10	GG	0.9944	gg	0.002797811	GG
NA12239	11	GG	0.9776	gg	0.018080466	GG
NA12878	12	AA	0.9532	aa	0.22983208	AA
NA11993	13	AA	0.9762	aa	0.009956107	AA
NA12750	14	AA	0.9488	aa	0.021508446	AA
NA12146	15	GA	0.9953	ga	0.262957047	GA
NA11839	16	GA	0.9985	ga	0.03373895	GA
NA12156.dup	17	GG	1	gg	0.073467754	nn
NA11829	18		NA	GA	0.209170424	
NA12154	19	GA	0.9799	ga	0.123570269	GA
NA10856	20	GG	1	gg	0.122543243	GG
NA06985	21	GG	1	gg	0.073548715	GG
NA11831	22	AA	0.98	aa	0.067374247	AA
NA12003.dup	23	AA	0.8991	aa	0.09665743	nn
NA10859	24	GA	0.9979	ga	0.112524496	GA
NA07056	25	AA	0.9849	aa	0.106931682	AA
NA07022	26	GA	0.9972	ga	0.277080026	GA
NA12762	27		NA	AA	0.321389076	
NA12874	28	GG	1	gg	0.146129092	GG
NA12003	29	AA	0.9826	aa	0.21533511	AA
NA10835	30	GA	0.9979	ga	0.01450087	GA
NA11882	31	AA	0.9438	aa	0.132093983	AA
NA07034	32	GA	0.9965	ga	0.168160286	GA
NA12056	33	GG	1	gg	0.149284235	GG
NA12145	34	AA	0.9992	aa	0.180847665	AA
NA07019	35	GA	0.9978	ga	0.264334542	GA
NA06991	36		NA	GA	0.390618377	
NA12761	37	GA	0.9953	ga	0.331929101	GA
NA06994	38	AA	0.9885	aa	0.171738229	AA
NA12864	39	AA	0.9694	aa	0.178554736	AA
NA07055	40	GA	0.9944	ga	0.336675826	GA
NA10863	41	GA	0.9967	ga	0.33174786	GA
NA12763	42	GG	1	gg	0.118822244	GG
NA10831	43	GA	0.9931	ga	0.272883251	GA
NA11840	44	GA	0.9968	ga	0.355548737	GA

	MACGT discrepancy					
	LDA discrepancy					
NA	LDA output were not g					
XY	Manual calls are given					
		**2084851**				
**Coriell**	**Sample**	**LDA Call**	**LDA quality score**	**MACGT Call**	**MACGT quality score**	**HapMap Call**
NA10830	45	AA	0.9787	aa	0.265776676	AA
NA11993.dup	46	AA	0.9581	aa	0.253713669	nn
NA12751	47	GA	0.9985	ga	0.402197172	GA
NA12814	48	GG	1	gg	0.01437155	GG
NA10857	49	GA	0.9891	ga	0.421814202	GA
NA07357	50	GA	0.9941	ga	0.491957608	GA
NA07000	51	AA	0.9422	aa	0.09712435	AA
NA12802	52	GA	0.9934	ga	0.288522499	GA
NA10855	53	AA	0.9985	aa	0.129392255	AA
NA11995	54		NA	GA	0.421477847	
NA10839	55	GA	0.9977	ga	0.322712465	GA
NA10847	56	GG	1	gg	0.235250929	GG
NA12006	57		NA	GG	0.246832012	
NA12144	58	GA	0.9976	ga	0.53263488	GA
NA11832	59	AA	0.9947	aa	0.290825328	AA
NA12813	60	GA	0.9951	ga	0.416465606	GA
blank	61	AA	0.9155	aa	0.204676173	nn
NA12891	62	GA	0.9456	ga	0.313852388	GA
NA12717	63		NA	GA	0.409323531	
NA10861	64	AA	0.9068	aa	0.185158649	AA
NA12004	65		NA	GA	0.252206512	
NA07048	66	AA	0.999	aa	0.277899798	AA
NA12801	67	AA	0.9968	aa	0.1241396	AA
NA11830	68	GG	1	gg	0.226892211	GG
NA12057	69	GA	0.9966	ga	0.331772905	GA
NA11994	70		NA	GA	0.475514658	
NA12740	71	GA	0.95	ga	0.465720337	GA
NA12005	72	GA	0.9985	ga	0.137261369	GA
NA07345	73	GG	1	gg	0.183361385	GG
NA10854	74	AA	0.9628	aa	0.140702798	AA
NA12872	75	GA	0.9957	ga	0.62380078	GA
NA12865	76	GA	0.9961	ga	0.270513358	GA
NA10860	77		NA	GA	0.273113465	
NA12873	78	GA	0.994	ga	0.542229607	GA
NA12752	79	GA	0.9879	ga	0.447055028	GA
NA10851	80	GA	0.9684	ga	0.323685865	GA
NA07029	81	AA	0.995	aa	0.273013981	AA
NA12248	82	AA	0.9994	aa	0.280932772	AA
NA12155	83	GA	0.9966	ga	0.044059266	GA
NA10838	84	AA	0.9714	aa	0.273515764	AA
NA12812	85	AA	0.8208	aa	0.242328183	AA
NA12248.dup	86	AA	0.8044	aa	0.199665222	nn
NA12043	87	GA	0.9847	ga	0.392420537	GA
NA12234	88	GG	1	gg	0.11177581	GG

	MACGT discrepancy					
	LDA discrepancy					
NA	LDA output were not g					
XY	Manual calls are given					
		2084851				
**Coriell**	**Sample**	**LDA Call**	**LDA quality score**	**MACGT Call**	**MACGT quality score**	**HapMap Call**
NA12892	89	AA	0.9993	aa	0.268790174	AA
NA10846	90	AA	0.9991	aa	0.511017109	AA
NA06993.dup	91	AA	0.9672	aa	0.459063589	nn
NA12249	92	GG	0.9999	gg	0.01787268	GG
NA11881	93	GA	0.9985	ga	0.309338431	GA
NA12815	94	AA	0.7585	aa	0.014576787	AA
NA12264	95	AA	0.9882	aa	0.161293558	AA
NA18526	96	AA	1	aa	0.261622899	AA
NA18562	97	GA	0.9879	ga	0.267134929	GA
NA18545	98	AA	0.9572	aa	0.257112591	AA
NA18609	99	GG	1	gg	0.018040554	GG
NA18566	100	AA	0.9129	aa	0.067488035	AA
NA18621	101	AA	0.9653	aa	0.057031261	AA
NA18577	102	GA	0.9974	ga	0.231502358	GA
NA18635	103	AA	0.9089	aa	0.076826941	AA
NA18524	104	AA	0.9999	aa	0.088023106	AA
NA18537	105	AA	0.9557	aa	0.124260222	AA
NA18572	106	GA	0.9963	ga	0.222892629	GA
NA18552	107	AA	0.7671	aa	0.131121183	AA
NA18563	108	GG	1	gg	0.385090523	GG
NA18594	109	GA	0.9964	ga	0.226572603	nn
NA18624	110	GA	0.9938	ga	0.355446787	GA
NA18592	111	AA	0.9541	aa	0.345970419	AA
NA18529	112	GA	0.9934	ga	0.287097582	GA
NA18603	113	GA	0.9959	ga	0.356646633	nn
NA18547	114	GA	0.9979	ga	0.230095001	GA
NA18611	115	GG	0.8874	gg	0.076495781	GG
NA18570	116	AA	0.889	aa	0.135770689	AA
NA18622	117	AA	0.9569	aa	0.353608447	AA
NA18579	118	AA	0.8984	aa	0.397221151	AA
NA18636	119	AA	0.9136	aa	0.053511672	AA
NA18558	120	AA	0.9457	aa	0.177824195	AA
NA18540	121	AA	0.8601	aa	0.078971131	AA
NA18609	122	GG	0.8545	gg	0.036229375	nn
NA18555	123	AA	0.7973	aa	0.019219546	AA
NA18612	124	AA	0.9608	aa	0.370609138	AA
NA18573	125		NA	AA	0.148721742	
NA18632	126	GA	0.9967	ga	0.453178009	GA
NA18593	127	AA	0.9943	aa	0.067552026	AA
NA18532	128	AA	0.9942	aa	0.188199758	AA
NA18605	129	GG	1	gg	0.355844337	GG
NA18550	130	GA	0.9884	ga	0.449943326	GA
NA18603	131	GA	0.9927	ga	0.466328426	GA
NA18571	132	AA	0.983	aa	0.382338743	AA

	MACGT discrepancy					
	LDA discrepancy					
NA	LDA output were not g					
XY	Manual calls are given					
		**2084851**				
**Coriell**	**Sample**	**LDA Call**	**LDA quality score**	**MACGT Call**	**MACGT quality score**	**HapMap Call**
NA18623	133	AA	0.9825	aa	0.458465443	AA
NA18582	134	GA	0.9935	ga	0.419803296	GA
NA18637	135	AA	0.9985	aa	0.140703645	AA
NA18561	136	GA	0.9967	ga	0.502873585	GA
NA18542	137	AA	0.9854	aa	0.294577522	AA
NA18608	138	AA	0.928	aa	0.290430721	AA
NA18564	139		NA	AA	0.302096785	
NA18620	140	GA	0.9971	ga	0.597341816	GA
NA18576	141		NA	AA	0.692887814	
NA18633	142		NA	AA	0.34710385	
NA18594	143	GA	0.9932	ga	0.088857212	GA
NA18942	144		NA	AA	0.310256373	
NA18945	145	GA	0.9877	ga	0.463398583	GA
NA18964	146		NA	AA	0.57064138	
NA18961	147	GG	1	***GG***	***0***	GG
NA18967	148	AA	0.9952	aa	0.528676589	AA
NA18981	149	AA	0.9455	aa	0.384469202	AA
NA18994	150	GA	0.9802	ga	0.544961426	GA
NA18998	151	AA	0.9368	aa	0.440256362	AA
NA18940	152	GA	0.9914	ga	0.477705701	GA
NA18949	153	GA	0.9876	ga	0.405116408	GA
NA18953	154	AA	0.8205	aa	0.232705779	AA
NA18972	155	AA	0.8306	aa	0.151917299	AA
NA18976	156	GA	0.9941	ga	0.540159783	GA
NA18971	157	GA	0.9848	ga	0.23720933	GA
blank	158	GG	0.7504	***NN***	***0***	nn
NA19000	159	GA	0.9966	ga	0.227587975	GA
NA18951	160	AA	0.936	aa	0.073959816	nn
NA18948	161	AA	0.9815	aa	0.026659814	AA
NA18968	162	AA	0.9974	aa	0.447261955	AA
NA18965	163	AA	0.982	aa	0.38467602	AA
NA18978	164	AA	0.9973	aa	0.106495776	AA
NA18974	165	AA	0.9946	aa	0.194829643	AA
NA18992	166	AA	0.8419	aa	0.217846164	AA
NA19005	167	AA	0.9999	aa	0.219101815	AA
NA18943	168	GA	0.998	ga	0.424076752	GA
NA18951	169	AA	0.999	aa	0.119813378	AA
NA18959	170	GA	0.9941	ga	0.207162725	GA
NA18973	171	GG	0.9962	gg	0.509252489	GG
NA18970	172	GG	1	gg	0.464498864	GG
NA18987	173	GG	1	gg	0.282828382	GG
NA18995	174	GG	0.9714	gg	0.39343259	GG
NA18999	175	AA	0.9882	aa	0.204365678	AA
NA18947	176	GA	0.9937	ga	0.248416222	GA

	MACGT discrepancy					
	LDA discrepancy					
NA	LDA output were not g					
XY	Manual calls are given					
		**2084851**				
**Coriell**	**Sample**	**LDA Call**	**LDA quality score**	**MACGT Call**	**MACGT quality score**	**HapMap Call**
NA18952	177	GA	0.996	ga	0.432425168	GA
NA18969	178	GA	0.9916	ga	0.361841127	GA
NA18966	179	AA	0.9957	aa	0.253950146	AA
NA18980	180	AA	0.844	aa	0.245398481	AA
NA18990	181	GA	0.9878	ga	0.237151902	GA
NA18997	182	AA	0.9172	aa	0.343805071	AA
NA19007	183	AA	0.9717	aa	0.246882765	AA
NA18944	184	AA	0.7813	aa	0.02635779	AA
NA18956	185	GG	1	***GG***	***0***	GG
NA18960	186	AA	0.9525	aa	0.318418204	AA
NA18975	187	AA	0.9225	aa	0.262952555	AA
NA18995	188	GG	1	gg	0.312903642	nn
NA18991	189	GA	0.9966	ga	0.643204185	GA
NA18996	190	GA	0.9929	ga	0.033188723	nn
NA19003	191	GA	0.9905	ga	0.658371516	GA
NA18502	192	AA	0.8355	aa	0.155371375	AA
NA19153	193	GG	1	gg	0.008364283	GG
NA18857	194	GG	1	gg	0.216777745	GG
NA19223	195	GG	1	gg	0.173009014	GG
NA19201	196	GA	0.9947	ga	0.500099802	GA
NA18504	197	GA	0.9768	ga	0.54156558	GA
NA18870	198	GG	1	gg	0.476826361	GG
NA18863	199	GG	1	gg	0.177791425	GG
NA19145	200	GG	0.9714	gg	0.074595811	GG
NA19137	201	GA	0.9974	ga	0.256398849	GA
NA19238	202	GG	1	gg	0.192066158	nn
NA18500	203	GA	0.9715	ga	0.017927449	GA
NA19144	204	GG	1	***GG***	***0***	GG
NA19203	205	GG	0.9483	gg	0.210715081	GG
NA19200	206		NA	GG	0.494018276	
NA18855	207		NA	GG	0.248233216	
NA18505	208	GG	1	gg	0.128149378	GG
NA19202	209	GG	0.9211	gg	0.015486268	GG
NA18501	210	GG	1	gg	0.201466198	GG
NA18861	211	GG	1	gg	0.002325806	GG
NA19193	212	GA	0.9968	ga	0.163992416	GA
NA19143	213	GG	1	gg	0.002233371	GG
NA18517	214	GA	0.9794	ga	0.253825178	GA
NA18862	215	GG	0.8847	gg	0.117014765	GG
NA18856	216	GG	0.9165	gg	0.139500671	nn
NA19239	217	GG	0.7918	gg	0.101389122	GG
NA19240	218	GG	1	gg	0.135357077	GG
NA18856	219	GG	1	gg	0.12345199	GG
NA18503	220	GG	0.9994	gg	0.238947749	GG

	MACGT discrepancy					
	LDA discrepancy					
NA	LDA output were not g					
XY	Manual calls are given					
				**2084851**		
**Coriell**	**Sample**	**LDA Call**	**LDA quality score**	**MACGT Call**	**MACGT quality score**	**HapMap Call**
NA18871	221	GG	0.9956	gg	0.462201566	GG
NA19221	222	GG	0.9888	gg	0.152094817	GG
NA19209	223	GG	1	gg	0.024477416	GG
NA19152	224	GG	1	gg	0.176510981	GG
NA18515	225	GA	0.9964	ga	0.288511635	GA
NA19238	226	GG	0.9934	gg	0.191967511	GG
NA19154	227	GG	1	gg	0.404298602	GG
NA19210	228	GG	1	gg	0.201016765	GG
NA19211	229	GG	1	gg	0.224012776	GG
NA18862	230	GG	0.9956	gg	0.030847297	nn
NA18872	231	GG	1	gg	0.382348097	GG
NA19139	232	GA	0.9941	ga	0.285458081	GA
NA19222	233	GG	0.8706	gg	0.207747426	GG
NA19194	234	AA	0.9983	aa	0.226161162	AA
NA19138	235	GG	1	gg	0.152171034	GG
NA19204	236	GA	0.9973	ga	0.479775549	GA
NA18516	237	GG	1	gg	0.029782105	GG
NA19205	238	GG	1	***gg***	***9.06E-04***	GG
NA19192	239	GA	0.971	ga	0.199283169	GA
NA18912	240	GG	1	gg	0.182313608	nn
NA18508	241	GG	0.9803	gg	0.052896527	GG
NA19142	242	GG	0.8838	gg	0.046912798	GG
NA18852	243	GG	1	gg	0.005473517	GG
NA18507	244	GA	0.9968	ga	0.288834005	GA
blank	245	AA	0.9751	aa	0.437326685	nn
NA19101	246	GG	1	gg	0.137057889	GG
NA19172	247	GG	0.913	***GG***	***0***	GG
NA19160	248	GG	0.9075	gg	0.192383286	GG
NA19129	249	GA	0.9939	ga	0.364772981	GA
NA18913	250	GG	0.9363	gg	0.247070826	GG
NA19120	251	GA	0.998	ga	0.280171224	GA
NA19159	252	GG	0.8771	gg	0.196283927	GG
NA18523	253	GA	0.9843	ga	0.492061615	GA
NA19102	254	GG	0.8268	gg	0.069291799	GG
NA19092	255	GA	0.9937	ga	0.26586131	GA
NA18521	256	GG	1	gg	0.049171931	GG
NA18912	257	GG	1	gg	0.151552192	GG
NA19206	258	GG	1	gg	0.22940408	GG
NA19128	259	GG	1	gg	0.11390476	GG
NA18859	260	GG	1	gg	0.124071865	nn
NA19094	261	GA	0.9918	ga	0.431381644	GA
NA19171	262	GA	0.8937	ga	0.242994738	GA
NA18860	263	GA	0.9977	ga	0.269906079	GA
NA18858	264	GA	0.9907	ga	0.44414982	GA

	MACGT discrepancy					
	LDA discrepancy					
NA	LDA output were not g					
XY	Manual calls are given					
		2084851				
**Coriell**	**Sample**	**LDA Call**	**LDA quality score**	**MACGT Call**	**MACGT quality score**	**HapMap Call**
NA19099	265	GG	1	gg	0.207198223	GG
NA18506	266	GA	0.9954	ga	0.230689123	GA
NA19131	267	GG	1	gg	0.229189174	GG
NA19116	268	GG	1	gg	0.333819549	GG
NA19161	269	GG	1	gg	0.251854119	GG
NA19140	270	GG	1	gg	0.400441163	GG
NA19207	271	GG	1	gg	0.117369606	GG
NA19141	272	GG	1	gg	0.226779667	GG
NA18854	273	GG	1	gg	0.226191401	GG
NA19098	274	GA	0.994	ga	0.433161525	GA
NA18522	275	GG	0.9681	gg	0.10992458	GG
NA18914	276	GG	1	gg	0.027305802	GG
NA18853	277	GG	0.9986	gg	0.213573997	GG
NA18859	278	GG	1	gg	0.227407361	GG
NA19100	279	GA	0.9956	ga	0.460574999	GA
NA19093	280	GG	1	gg	0.25287684	GG
NA19132	281	GA	0.9967	ga	0.346660946	GA
NA19130	282	GA	0.9988	ga	0.496585	GA
NA19208	283	GG	1	gg	0.057552496	GG
NA19127	284	GA	0.9645	ga	0.321264266	GA
NA19173	285	GA	0.9853	ga	0.067849228	GA
NA19119	286	GA	0.9969	ga	0.523707074	GA
NA19103	287	GG	1	gg	0.148022609	GG
	Discrepancies	0		0		
	NNs	-2		-1		
	Automated Calls	268		262		
	Manual Calls	0		6		
	Concordant Calls	249		249		249

	MACGT discrepancy					
	LDA discrepancy					
NA	LDA output were not g					
XY	Manual calls are given					
		**2134180**				
**Coriell**	**Sample**	**LDA Call**	**LDA quality score**	**MACGT Call**	**MACGT quality score**	**HapMap Call**
NA12753	1		NA	TC	0.454491301	
NA12875	2	TC	0.9994	tc	0.3791906	TC
NA12044	3	CC	1	cc	0.231519362	CC
NA06993	4	TC	1	tc	0.350396653	TC
NA12716	5	CC	0.9927	cc	0.001303421	CC
NA12760	6	TC	0.9988	tc	0.467335258	TC
NA07348	7	TC	0.9898	tc	0.537857078	TC
NA12707	8	CC	0.9997	cc	0.041581614	CC
NA12156	9		NA	TC	0.361544735	
NA11992	10	TT	0.9997	tt	0.239696268	TT
NA12239	11	TT	0.9995	tt	0.24860737	TT
NA12878	12	CC	0.9328	cc	0.031953158	CC
NA11993	13	CC	1	cc	0.023517175	CC
NA12750	14	CC	0.9992	cc	0.035399187	CC
NA12146	15	TC	0.9992	tc	0.217615114	TC
NA11839	16	CC	1	cc	0.040001696	CC
NA12156.dup	17	TC	0.9999	tc	0.344721731	nn
NA11829	18		NA	TT	0.401623687	
NA12154	19	CC	0.9971	cc	0.107592166	CC
NA10856	20	TT	0.772	tt	0.067553555	TT
NA06985	21	CC	1	cc	0.105545847	CC
NA11831	22	TC	0.9999	tc	0.065499554	TC
NA12003.dup	23	TC	0.9985	tc	0.077853123	nn
NA10859	24	TC	0.9997	tc	0.210148303	TC
NA07056	25	TC	1	tc	0.207007211	TC
NA07022	26	TC	0.9997	tc	0.244535318	TC
NA12762	27		NA	TC	0.623344883	
NA12874	28	TC	0.9992	tc	0.048924641	TC
NA12003	29	TC	0.9998	tc	0.326317412	TC
NA10835	30	CC	1	cc	0.015849213	CC
NA11882	31	CC	0.9815	cc	0.062576435	CC
NA07034	32	TT	0.9543	tt	0.010406013	TT
NA12056	33	CC	0.9844	cc	0.093476371	CC
NA12145	34	CC	0.9999	cc	0.087355577	CC
NA07019	35	TC	0.9998	tc	0.401678102	TC
NA06991	36		NA	CC	0.335504059	
NA12761	37	TC	1	tc	0.096561393	TC
NA06994	38	CC	1	cc	0.025881059	CC
NA12864	39	TC	0.9999	tc	0.699447915	TC
NA07055	40	CC	0.9675	cc	0.191148749	CC
NA10863	41	TC	1	tc	0.347373322	TC
NA12763	42	CC	0.9971	cc	0.119948925	CC
NA10831	43	CC	0.9997	cc	0.039827677	CC
NA11840	44	CC	0.9982	cc	0.041173708	CC

	MACGT discrepancy					
	LDA discrepancy					
NA	LDA output were not g					
XY	Manual calls are given					
		2134180				
**Coriell**	**Sample**	**LDA Call**	**LDA quality score**	**MACGT Call**	**MACGT quality score**	**HapMap Call**
NA10830	45	CC	0.9998	cc	0.005473832	CC
NA11993.dup	46	CC	0.7746	cc	0.085039938	nn
NA12751	47	CC	1	cc	0.022563485	CC
NA12814	48	CC	0.7679	*cc*	*9.86E-04*	CC
NA10857	49	CC	0.9995	cc	0.129628516	CC
NA07357	50	TT	1	tt	0.264443636	TT
NA07000	51	TC	0.9991	tc	0.375540222	TC
NA12802	52	CC	0.9609	cc	0.00595558	CC
NA10855	53	CC	0.7785	cc	0.004308454	CC
NA11995	54		NA	TT	0.260569874	
NA10839	55	CC	0.7773	cc	0.006844472	CC
NA10847	56	TT	0.9996	tt	0.057447038	TT
NA12006	57	CC	0.966	cc	0.003727678	CC
NA12144	58	TT	0.9995	tt	0.084200385	TT
NA11832	59	CC	0.9486	cc	0.001368149	CC
NA12813	60	TC	0.9995	tc	0.355805651	TC
blank	61	NN	0.5	*NN*	*0*	nn
NA12891	62	CC	0.9789	cc	0.107130105	CC
NA12717	63		NA	CC	0.278427767	
NA10861	64	TC	0.9965	tc	0.24693304	TC
NA12004	65	CC	0.9811	*CC*	*0*	CC
NA07048	66	TC	0.9995	tc	0.41419627	TC
NA12801	67	NN	0.7461	*TT*	*0*	TT
NA11830	68	TT	0.9996	tt	0.05689125	TT
NA12057	69	CC	1	cc	0.004247186	CC
NA11994	70		NA	TC	0.442638343	
NA12740	71	CC	0.9641	cc	0.08076363	CC
NA12005	72	TC	0.9654	tc	0.009310867	TC
NA07345	73	CC	0.9946	cc	0.002818164	CC
NA10854	74	NN	0.6272	*CC*	*0*	CC
NA12872	75	NN	0.6754	*TT*	*0*	TT
NA12865	76	TC	0.9993	tc	0.26982002	TC
NA10860	77		NA	TC	0.357461946	
NA12873	78	CC	0.9633	*CC*	*0*	CC
NA12752	79	TT	0.8472	tt	0.016047598	TT
NA10851	80	CC	0.9851	cc	0.119533489	CC
NA07029	81	TC	0.9247	tc	0.030074846	TC
NA12248	82	TC	0.9995	tc	0.4708468	TC
NA12155	83	NN	0.7216	*CC*	*0*	CC
NA10838	84	TC	0.9951	tc	0.032143181	TC
NA12812	85	TC	0.9704	tc	0.015032803	TC
NA12248.dup	86	TC	0.9353	tc	0.04702366	nn
NA12043	87	NN	0.6637	tc	0.004617069	TC
NA12234	88	CC	1	cc	0.097061497	CC

	MACGT discrepancy					
	LDA discrepancy					
NA	LDA output were not g					
XY	Manual calls are given					
		**2134180**				
**Coriell**	**Sample**	**LDA Call**	**LDA quality score**	**MACGT Call**	**MACGT quality score**	**HapMap Call**
NA12892	89	CC	0.8993	***CC***	***0***	CC
NA10846	90	TC	0.9926	tc	0.187483282	TC
NA06993.dup	91	TC	0.9959	tc	0.196181643	nn
NA12249	92	TC	0.9985	tc	0.02191858	TC
NA11881	93	TC	0.9928	tc	0.05258712	TC
NA12815	94	NN	0.7324	***CC***	***0***	CC
NA12264	95	TC	0.9931	tc	0.100195228	TC
NA18526	96	TC	0.9973	tc	0.641438556	TC
NA18562	97	TT	0.9999	tt	0.18241574	TT
NA18545	98	CC	0.997	cc	0.036362152	CC
NA18609	99	TC	0.9986	tc	0.592416186	TC
NA18566	100	TC	0.9981	tc	0.306188603	TC
NA18621	101	CC	0.9978	cc	0.180233107	CC
NA18577	102	CC	0.9986	cc	0.20982793	CC
NA18635	103	CC	0.9928	cc	0.169558462	CC
NA18524	104	CC	0.9996	cc	0.224098563	CC
NA18537	105	TT	0.9455	tt	0.005784483	TT
NA18572	106	TT	0.9421	tt	0.02905225	TT
NA18552	107	CC	0.988	cc	0.13347699	CC
NA18563	108	TT	0.9996	tt	0.023192031	TT
NA18594	109	TC	0.9985	tc	0.422630517	nn
NA18624	110	CC	0.9832	***CC***	***0***	CC
NA18592	111	TC	0.9849	tc	0.514251297	TC
NA18529	112	CC	0.8853	cc	0.152684901	CC
NA18603	113	CC	0.8877	cc	0.13952496	nn
NA18547	114	TT	0.9999	tt	0.123204871	TT
NA18611	115	TC	0.9967	tc	0.06183775	TC
NA18570	116	CC	0.7731	***CC***	***0***	CC
NA18622	117	TC	0.9986	tc	0.480561608	TC
NA18579	118	CC	0.962	cc	0.151450623	CC
NA18636	119	TC	0.9979	tc	0.382285236	TC
NA18558	120	TC	0.9742	tc	0.305803182	TC
NA18540	121	TC	0.9813	tc	0.050872622	TC
NA18609	122	TC	0.996	tc	0.109212087	nn
NA18555	123	TC	0.9994	tc	0.008006641	TC
NA18612	124	CC	0.9997	cc	0.094909348	CC
NA18573	125	TC	0.9972	tc	0.106648835	TC
NA18632	126	TC	0.9827	tc	0.050459801	TC
NA18593	127	NN	0.5065	***CC***	***0***	CC
NA18532	128	CC	0.8586	***CC***	***0***	CC
NA18605	129	TC	0.9987	tc	0.290481783	TC
NA18550	130	CC	0.9577	***CC***	***0***	CC
NA18603	131	CC	0.9932	cc	0.211308392	CC
NA18571	132	TC	0.9958	tc	0.267828446	TC

	MACGT discrepancy					
	LDA discrepancy					
NA	LDA output were not g					
XY	Manual calls are given					
		**2134180**				
**Coriell**	**Sample**	**LDA Call**	**LDA quality score**	**MACGT Call**	**MACGT quality score**	**HapMap Call**
NA18623	133	NN	0.5013	tt	0.00354355	TT
NA18582	134	TC	0.999	tc	0.382897937	TC
NA18637	135	TC	0.973	tc	0.378302862	TC
NA18561	136	TC	0.9866	tc	0.146593392	TC
NA18542	137	TC	0.9827	tc	0.064795803	TC
NA18608	138	CC	0.9975	cc	0.125697581	CC
NA18564	139	TC	0.963	tc	0.118449999	TC
NA18620	140	NN	0.6212	*CC*	*0*	CC
NA18576	141	TC	0.968	tc	0.113280944	TC
NA18633	142	TC	0.923	tc	0.071045845	TC
NA18594	143	TC	0.9348	tc	0.028664791	TC
NA18942	144	CC	1	cc	0.136291281	CC
NA18945	145	TT	0.9999	tt	0.225372263	TT
NA18964	146	TT	0.9999	tt	0.336075487	TT
NA18961	147	TC	0.9999	tc	0.581559757	TC
NA18967	148	TC	0.9999	tc	0.559226911	TC
NA18981	149	TC	0.9025	tc	0.520700634	TC
NA18994	150	TC	0.9948	tc	0.38938012	TC
NA18998	151	TC	0.9989	tc	0.265013109	TC
NA18940	152	TT	0.9915	tt	0.060779307	TT
NA18949	153	TT	0.9992	tt	0.168658525	TT
NA18953	154	TT	0.999	*TT*	*0*	TT
NA18972	155	TC	0.9936	tc	0.377953557	TC
NA18976	156	TT	0.9995	tt	0.066249233	TT
NA18971	157	TC	0.998	tc	0.328706077	TC
blank	158	NN	0.494	*NN*	*0*	nn
NA19000	159	TT	1	tt	0.434195894	TT
NA18951	160	TC	0.9919	tc	0.412099285	nn
NA18948	161	TT	1	tt	0.197363759	TT
NA18968	162	TT	1	tt	0.249114586	TT
NA18965	163	TC	0.9977	tc	0.242520109	TC
NA18978	164	TT	1	tt	0.330551401	TT
NA18974	165	TC	0.9349	tc	0.261044557	TC
NA18992	166	TC	0.9464	tc	0.212969119	TC
NA19005	167	NN	0.4999	*TC*	*0*	TC
NA18943	168	NN	0.4999	*TC*	*0*	TC
NA18951	169	NN	0.4999	*TC*	*0*	TC
NA18959	170	NN	0.4999	*TC*	*0*	TC
NA18973	171	NN	0.4999	*TC*	*0*	TC
NA18970	172	NN	0.5	*TT*	*0*	TT
NA18987	173	NN	0.4999	*TC*	*0*	TC
NA18995	174	NN	0.4999	*TC*	*0*	TC
NA18999	175	NN	0.4864	*CC*	*0*	CC
NA18947	176	NN	0.4999	tc	0.004306875	TC

	MACGT discrepancy					
	LDA discrepancy					
NA	LDA output were not g					
XY	Manual calls are given					
		2134180				
**Coriell**	**Sample**	**LDA Call**	**LDA quality score**	**MACGT Call**	**MACGT quality score**	**HapMap Call**
NA18952	177	NN	0.5	*TT*	*0*	TT
NA18969	178	NN	0.4999	*TC*	*0*	TC
NA18966	179	NN	0.75	*TC*	*0*	TC
NA18980	180	NN	0.4995	*NN*	*0*	CC
NA18990	181	NN	0.4999	*TC*	*0*	TC
NA18997	182	NN	0.4999	*TC*	*0*	TC
NA19007	183	NN	0.4999	tc	0.494900035	TC
NA18944	184	NN	0.5001	*CC*	*0*	CC
NA18956	185	NN	0.4994	*NN*	*0*	CC
NA18960	186	NN	0.4999	*TC*	*0*	TC
NA18975	187	NN	0.4999	*TC*	*0*	TC
NA18995	188	NN	0.75	*TC*	*0*	nn
NA18991	189	NN	0.4999	*TC*	*0*	TC
NA18996	190	NN	0.75	*TC*	*0*	nn
NA19003	191	NN	0.4999	*TC*	*0*	TC
NA18502	192	NN	0.5	*TT*	*0*	TT
NA19153	193	NN	0.5	*TT*	*0*	TT
NA18857	194	NN	0.5174	*TT*	*0*	TT
NA19223	195	NN	0.4998	*TT*	*0*	TT
NA19201	196	NN	0.5	*TT*	*0*	TT
NA18504	197	NN	0.5	*TC*	*0*	TC
NA18870	198	NN	0.5	*TT*	*0*	TT
NA18863	199	NN	0.5	*TT*	*0*	TT
NA19145	200	NN	0.5	*TT*	*0*	TT
NA19137	201	NN	0.5	*TT*	*0*	TT
NA19238	202	TT	0.9985	*TT*	*0*	nn
NA18500	203	NN	0.4974	*tt*	*9.83E-04*	TT
NA19144	204	NN	0.4999	*TT*	*0*	TT
NA19203	205	TT	0.9993	tt	0.130416169	TT
NA19200	206	NN	0.5	*TT*	*0*	TT
NA18855	207	NN	0.4995	*TT*	*0*	TT
NA18505	208	NN	0.7476	tt	0.006385058	TT
NA19202	209	NN	0.5004	*TT*	*0*	TT
NA18501	210	NN	0.5	*NN*	*0*	TT
NA18861	211	NN	0.5	*TT*	*0*	TT
NA19193	212	NN	0.5	*TT*	*0*	TT
NA19143	213	NN	0.5	*NN*	*0*	TT
NA18517	214	TT	0.9995	tt	0.378144861	TT
NA18862	215	NN	0.5	*TT*	*0*	TT
NA18856	216	NN	0.5	*TT*	*0*	nn
NA19239	217	NN	0.5	*TT*	*0*	TT
NA19240	218	TT	0.9996	tt	0.30522706	TT
NA18856	219	NN	0.5	tt	0.004983764	TT
NA18503	220	NN	0.5023	*TC*	*0*	TC

	MACGT discrepancy					
	LDA discrepancy					
NA	LDA output were not g					
XY	Manual calls are given					
		2134180				
**Coriell**	**Sample**	**LDA Call**	**LDA quality score**	**MACGT Call**	**MACGT quality score**	**HapMap Call**
NA18871	221	NN	0.5	*TT*	*0*	TT
NA19221	222	NN	0.5	*TT*	*0*	TT
NA19209	223	NN	0.5	*TT*	*0*	TT
NA19152	224	TT	1	tt	0.219954675	TT
NA18515	225	NN	0.5001	*TT*	*0*	TT
NA19238	226	NN	0.5	tt	0.431409663	TT
NA19154	227	NN	0.5	*TT*	*0*	TT
NA19210	228	NN	0.5	tt	0.05768199	TT
NA19211	229	NN	0.5	*TT*	*0*	TT
NA18862	230	TT	0.9999	tt	0.530954501	nn
NA18872	231	NN	0.5	*TT*	*0*	TT
NA19139	232	NN	0.4998	*TT*	*0*	TT
NA19222	233	NN	0.499	tt	0.002045005	TT
NA19194	234	NN	0.4996	tc	0.008631396	TT
NA19138	235	NN	0.5	*TT*	*0*	TT
NA19204	236	TT	0.9995	tt	0.142938471	TT
NA18516	237	NN	0.5	*TT*	*0*	TT
NA19205	238	NN	0.5	*TT*	*0*	TT
NA19192	239	NN	0.5	*TT*	*0*	TT
NA18912	240	NN	0.5	*TT*	*0*	nn
NA18508	241	NN	0.5	*TT*	*0*	TT
NA19142	242	NN	0.5	*TT*	*0*	TT
NA18852	243	NN	0.5	*TT*	*0*	TT
NA18507	244	NN	0.4982	tt	0.254007084	TT
blank	245	NN	0.4987	*NN*	*0*	nn
NA19101	246	NN	0.5	*TT*	*0*	TT
NA19172	247	NN	0.5	*TT*	*0*	TT
NA19160	248	NN	0.5	*TT*	*0*	TT
NA19129	249	NN	0.5	*TT*	*0*	TT
NA18913	250	NN	0.5	*TT*	*0*	TT
NA19120	251	NN	0.5	tt	0.169483386	TT
NA19159	252	NN	0.5	*TT*	*0*	TT
NA18523	253	TT	0.9997	tt	0.405421486	TT
NA19102	254	NN	0.611	*TT*	*0*	TT
NA19092	255	NN	0.5	*TT*	*0*	TT
NA18521	256	NN	0.5	tt	0.250448334	TT
NA18912	257	NN	0.5	tt	0.042360299	TT
NA19206	258	NN	0.5001	*TT*	*0*	TT
NA19128	259	NN	0.5	*TT*	*0*	TT
NA18859	260	NN	0.5	tt	0.075320624	nn
NA19094	261	NN	0.5	*TT*	*0*	TT
NA19171	262	NN	0.5	*TT*	*0*	TT
NA18860	263	TT	0.9999	tt	0.262061946	TT
NA18858	264	TT	1	tt	0.306307828	TT

	MACGT discrepancy					
	LDA discrepancy					
NA	LDA output were not g					
XY	Manual calls are given					
		2134180				
**Coriell**	**Sample**	**LDA Call**	**LDA quality score**	**MACGT Call**	**MACGT quality score**	**HapMap Call**
NA19099	265	TT	0.9998	tt	0.178989624	TT
NA18506	266	TT	0.9995	tt	0.117449291	TT
NA19131	267	TT	0.9999	tt	0.45208721	TT
NA19116	268	TT	0.9971	tt	0.308554947	TT
NA19161	269	TT	0.9998	tt	0.122426216	TT
NA19140	270	TT	0.9994	tt	0.210048725	TT
NA19207	271	TT	0.9997	tt	0.28415936	TT
NA19141	272	TT	0.9998	tt	0.292476742	TT
NA18854	273	TT	0.9997	tt	0.112919567	TT
NA19098	274	TT	0.9998	tt	0.367721301	TT
NA18522	275	TT	0.9869	tt	0.020812071	TT
NA18914	276	TT	1	tt	0.220597396	TT
NA18853	277	TT	1	tt	0.294730962	TT
NA18859	278	TT	0.9343	tt	0.024093465	TT
NA19100	279	TT	0.9999	tt	0.262828967	TT
NA19093	280	TT	0.9987	tt	0.057712063	TT
NA19132	281	TT	0.7822	tt	0.011171623	TT
NA19130	282	TT	0.9998	tt	0.111590775	TT
NA19208	283	TT	0.9991	tt	0.106160258	TT
NA19127	284	TT	0.999	tt	0.0122875	TT
NA19173	285	TT	0.9977	tt	0.061743524	TT
NA19119	286	TT	0.9989	tt	0.103686669	TT
NA19103	287	TT	0.9998	tt	0.21211916	TT
	Discrepancies	0		1		
	NNs	97		5		
	Automated Calls	278		184		
	Manual Calls	0		94		
	Concordant Calls	168		254		259

	MACGT discrepancy					
	LDA discrepancy					
NA	LDA output were not g					
XY	Manual calls are given					
		**2156208**				
**Coriell**	**Sample**	**LDA Call**	**LDA quality score**	**MACGT Call**	**MACGT quality score**	**HapMap Call**
NA12753	1		NA	TT	0.446509786	
NA12875	2	TT	0.9995	tt	0.514679462	TT
NA12044	3	CT	0.9702	ct	0.48176286	CT
NA06993	4	CT	0.9908	ct	0.342214416	CT
NA12716	5	CT	0.9255	ct	0.057934093	CT
NA12760	6	CT	0.9706	ct	0.184972001	CT
NA07348	7	CC	0.9964	cc	0.171644474	CC
NA12707	8	TT	1	tt	0.569992259	TT
NA12156	9		NA	CT	0.239288434	
NA11992	10	TT	0.9819	tt	0.593549779	TT
NA12239	11	CT	0.9778	ct	0.18029213	CT
NA12878	12	NN	0.7484	***CC***	***0***	CC
NA11993	13	CT	0.9888	***CT***	***6.05E-04***	CT
NA12750	14	CC	0.9894	cc	0.113017485	CC
NA12146	15	TT	0.9949	tt	0.277214944	TT
NA11839	16	CT	0.9809	ct	0.015403774	CT
NA12156.dup	17	CT	0.973	ct	0.317454046	nn
NA11829	18		NA	TT	0.605382256	
NA12154	19	TT	0.9991	tt	0.528156641	TT
NA10856	20	TT	0.9986	tt	0.403263586	TT
NA06985	21	CT	0.9902	ct	0.2245485	CT
NA11831	22	CT	0.9858	ct	0.009514796	CT
NA12003.dup	23	NN	0.7363	cc	0.001983916	nn
NA10859	24	CC	0.9928	cc	0.219820562	CC
NA07056	25	TT	0.9936	tt	0.239291346	TT
NA07022	26	CC	0.9787	cc	0.284310475	CC
NA12762	27		NA	TT	0.53024777	
NA12874	28	CT	0.9878	ct	0.022085423	CT
NA12003	29	CC	0.8851	cc	0.248498547	CC
NA10835	30	CT	0.982	ct	0.095709357	CT
NA11882	31	CC	0.979	cc	0.161596107	CC
NA07034	32	CC	0.965	cc	0.009802193	CC
NA12056	33	CC	0.9694	cc	0.215184012	CC
NA12145	34	TT	0.9997	tt	0.261453871	TT
NA07019	35	CT	0.9537	ct	0.519585477	CT
NA06991	36		NA	CT	0.514543307	
NA12761	37	CT	0.9976	ct	0.227549044	CT
NA06994	38	CC	0.9896	cc	0.345939928	CC
NA12864	39	CT	0.9666	ct	0.432160742	CT
NA07055	40	CT	0.9918	ct	0.27975035	CT
NA10863	41	TT	0.9948	tt	0.515880765	TT
NA12763	42	CT	0.9923	ct	0.580807547	CT
NA10831	43	TT	0.9922	tt	0.369285456	TT
NA11840	44	CC	0.9818	cc	0.347965619	CC

	MACGT discrepancy					
	LDA discrepancy					
NA	LDA output were not g					
XY	Manual calls are given					
		**2156208**				
**Coriell**	**Sample**	**LDA Call**	**LDA quality score**	**MACGT Call**	**MACGT quality score**	**HapMap Call**
NA10830	45	CT	0.9635	ct	0.437959359	CT
NA11993.dup	46	CT	0.982	ct	0.415972929	nn
NA12751	47	CC	0.973	cc	0.446900783	CC
NA12814	48	TT	0.8064	tt	0.550357878	TT
NA10857	49	CC	0.9593	cc	0.379791116	CC
NA07357	50	CC	0.9897	cc	0.207018535	CC
NA07000	51		NA	TT	0.515205792	
NA12802	52		NA	TT	0.410189939	
NA10855	53	NN	0.7396	cc	0.023798101	CC
NA11995	54		NA	CT	0.226010978	
NA10839	55	CC	0.9852	cc	0.064030568	CC
NA10847	56	CT	0.9841	ct	0.194631213	CT
NA12006	57	NN	0.7301	cc	0.116430789	CC
NA12144	58	CT	0.9579	ct	0.41382264	CT
NA11832	59	CC	0.9274	***CC***	***0***	CC
NA12813	60	CT	0.9607	ct	0.348514572	CT
blank	61	CC	0.9646	cc	0.00684811	nn
NA12891	62	CT	0.9564	ct	0.505364744	CT
NA12717	63		NA	TT	0.468042414	
NA10861	64	CT	0.9465	ct	0.093119448	CT
NA12004	65		NA	CT	0.247244084	
NA07048	66	CT	0.9926	ct	0.404890283	CT
NA12801	67	CT	0.8982	ct	0.188969172	CT
NA11830	68	CT	0.9727	ct	0.1614222	CT
NA12057	69	CC	0.7718	cc	0.03930595	CC
NA11994	70		NA	CT	0.467579648	
NA12740	71	CC	0.9606	cc	0.103264969	CC
NA12005	72	NN	0.5052	***NN***	***0***	CC
NA07345	73	NN	0.5001	***CC***	***0***	CC
NA10854	74	NN	0.4743	tt	0.002088894	CT
NA12872	75	NN	0.5307	***NN***	***0***	CC
NA12865	76	NN	0.6007	***CT***	***0***	CT
NA10860	77		NA	TT	0.414325646	
NA12873	78	NN	0.3746	***NN***	***0***	CT
NA12752	79	NN	0.5357	***NN***	***0***	CT
NA10851	80	CC	0.9956	cc	0.203433215	CC
NA07029	81	NN	0.4761	***NN***	***6.84E-04***	CT
NA12248	82	NN	0.5095	***CC***	***0***	CC
NA12155	83	NN	0.5	***NN***	***0***	CT
NA10838	84	NN	0.706	***NN***	***0***	CT
NA12812	85	NN	0.58	***NN***	***0***	CC
NA12248.dup	86	NN	0.6791	***NN***	***0***	nn
NA12043	87	NN	0.51	***NN***	***0***	CC
NA12234	88	CT	0.9663	ct	0.366978068	CT

	MACGT discrepancy					
	LDA discrepancy					
NA	LDA output were not g					
XY	Manual calls are given					
				2156208		
**Coriell**	**Sample**	**LDA Call**	**LDA quality score**	**MACGT Call**	**MACGT quality score**	**HapMap Call**
NA12892	89	NN	0.5056	*CC*	*0*	CC
NA10846	90	TT	0.9353	tt	0.004140835	TT
NA06993.dup	91	NN	0.4628	*CT*	*0*	nn
NA12249	92	NN	0.6646	ct	0.001217486	CT
NA11881	93	NN	0.6599	*CC*	*0*	CC
NA12815	94	NN	0.4857	tt	0.068211906	TT
NA12264	95	NN	0.7397	tt	0.094512162	TT
NA18526	96	TT	0.9963	tt	0.341008706	TT
NA18562	97	TT	0.9972	tt	0.565512527	TT
NA18545	98	TT	0.9982	tt	0.464659495	TT
NA18609	99	TT	0.9928	tt	0.311047836	TT
NA18566	100	TT	0.9978	tt	0.353112666	TT
NA18621	101	TT	0.9999	tt	0.443392886	TT
NA18577	102	TT	0.9994	tt	0.314855978	TT
NA18635	103	TT	0.9996	tt	0.30868015	TT
NA18524	104	TT	0.9897	tt	0.419057253	TT
NA18537	105	TT	0.9995	tt	0.346814144	TT
NA18572	106	TT	0.9995	tt	0.339375408	TT
NA18552	107	TT	0.9996	tt	0.263367149	TT
NA18563	108	TT	0.9996	tt	0.244987604	TT
NA18594	109	TT	1	tt	0.296673294	nn
NA18624	110	CT	0.9903	ct	0.411860966	CT
NA18592	111	TT	0.9985	tt	0.611359083	TT
NA18529	112	TT	0.9997	tt	0.404788912	TT
NA18603	113	CT	0.911	ct	0.480783195	nn
NA18547	114	TT	0.9999	tt	0.700514822	TT
NA18611	115	TT	0.7569	tt	0.383707868	TT
NA18570	116	TT	0.8347	tt	0.438649389	TT
NA18622	117	TT	0.9976	tt	0.53763559	TT
NA18579	118	TT	0.9875	tt	0.447756395	TT
NA18636	119	TT	0.9926	tt	0.588193098	TT
NA18558	120	CT	0.8349	ct	0.005664475	CT
NA18540	121	CT	0.7511	*CT*	*0*	CT
NA18609	122	TT	0.7744	tt	0.310347994	nn
NA18555	123	NN	0.7493	*CT*	*0*	CT
NA18612	124	CT	0.9971	ct	0.247785096	CT
NA18573	125	NN	0.5124	tt	0.076888186	TT
NA18632	126	NN	0.5	*NN*	*0*	TT
NA18593	127	NN	0.4949	tt	0.003776998	TT
NA18532	128	NN	0.6125	*CT*	*0*	CT
NA18605	129	NN	0.7108	tt	0.181485194	TT
NA18550	130	TT	0.9345	tt	0.131264593	TT
NA18603	131	CT	0.9699	ct	0.243447823	CT
NA18571	132	TT	0.7759	*TT*	*7.23E-04*	TT

	MACGT discrepancy					
	LDA discrepancy					
NA	LDA output were not g					
XY	Manual calls are given					
		2156208				
**Coriell**	**Sample**	**LDA Call**	**LDA quality score**	**MACGT Call**	**MACGT quality score**	**HapMap Call**
NA18623	133	TT	0.9919	tt	0.062653428	TT
NA18582	134	TT	0.9802	tt	0.319689631	TT
NA18637	135	TT	0.9986	tt	0.452248758	TT
NA18561	136	TT	0.8452	tt	0.090119422	TT
NA18542	137	NN	0.6586	tt	0.004993912	TT
NA18608	138	TT	0.9993	tt	0.635755679	TT
NA18564	139	NN	0.5075	tt	0.005220971	TT
NA18620	140	NN	0.7188	tt	0.16099967	TT
NA18576	141	NN	0.5047	tt	0.010017321	TT
NA18633	142	NN	0.6135	tt	0.011102094	TT
NA18594	143	NN	0.502	tt	0.005859161	TT
NA18942	144	TT	0.9858	tt	0.392614138	TT
NA18945	145	TT	0.9977	tt	0.545295543	TT
NA18964	146	TT	0.9999	tt	0.599167102	TT
NA18961	147	CT	0.92	ct	0.612960167	CT
NA18967	148	TT	0.9996	tt	0.607733953	TT
NA18981	149	CT	0.9459	ct	0.25722676	CT
NA18994	150	TT	1	tt	0.642379367	TT
NA18998	151	TT	0.9966	tt	0.545822834	TT
NA18940	152	TT	0.9998	tt	0.640876026	TT
NA18949	153	CT	0.9824	ct	0.104461426	CT
NA18953	154	TT	1	tt	0.605602056	TT
NA18972	155	TT	0.9969	tt	0.524970972	TT
NA18976	156	CT	0.9815	ct	0.227152924	CT
NA18971	157	TT	0.9973	tt	0.627506934	TT
blank	158	NN	0.7119	*NN*	*0*	nn
NA19000	159	TT	0.9993	tt	0.225605472	TT
NA18951	160	TT	0.9977	tt	0.327849487	nn
NA18948	161	TT	0.9955	tt	0.434157317	TT
NA18968	162	NN	0.7153	ct	0.10015789	CT
NA18965	163	CT	0.9503	ct	0.073401717	CT
NA18978	164	TT	0.9996	tt	0.398163133	TT
NA18974	165	TT	0.9955	tt	0.449340023	TT
NA18992	166	TT	0.9955	tt	0.424965845	TT
NA19005	167	CT	0.991	ct	0.364192484	CT
NA18943	168	TT	0.999	tt	0.558886495	TT
NA18951	169	TT	0.9999	tt	0.464780128	TT
NA18959	170	TT	0.9994	tt	0.663828682	TT
NA18973	171	TT	0.9996	tt	0.607169131	TT
NA18970	172	CT	0.951	ct	0.384291144	CT
NA18987	173	CT	0.8277	ct	0.188374667	CT
NA18995	174	TT	0.9942	tt	0.547330618	TT
NA18999	175	TT	0.9956	tt	0.55907657	TT
NA18947	176	NN	0.7028	ct	0.182930984	CT

	MACGT discrepancy					
	LDA discrepancy					
NA	LDA output were not g					
XY	Manual calls are given					
		2156208				
**Coriell**	**Sample**	**LDA Call**	**LDA quality score**	**MACGT Call**	**MACGT quality score**	**HapMap Call**
NA18952	177	TT	1	tt	0.551934577	TT
NA18969	178	TT	0.9999	tt	0.553600381	TT
NA18966	179	TT	0.9988	tt	0.579344838	TT
NA18980	180	TT	0.987	tt	0.656248354	TT
NA18990	181	TT	0.9991	tt	0.551436447	TT
NA18997	182	TT	0.9999	tt	0.557389787	TT
NA19007	183	TT	1	tt	0.658044654	TT
NA18944	184	TT	0.9962	tt	0.695489098	TT
NA18956	185	TT	0.9997	tt	0.602123463	TT
NA18960	186	TT	0.9997	tt	0.59134032	TT
NA18975	187	CT	0.9613	ct	0.247740332	CT
NA18995	188	TT	0.9998	tt	0.597419054	nn
NA18991	189	TT	0.9994	tt	0.530619366	TT
NA18996	190	TT	0.9998	tt	0.498449671	nn
NA19003	191	TT	0.9897	tt	0.713661541	TT
NA18502	192	NN	0.4866	tt	0.002388923	TT
NA19153	193	CT	0.8341	***CT***	***0***	CT
NA18857	194	NN	0.5342	***NN***	***6.45E-04***	CT
NA19223	195	NN	0.7454	***NN***	***0***	CC
NA19201	196	NN	0.7499	***NN***	***0***	CC
NA18504	197	NN	0.75	tt	0.002458257	CC
NA18870	198	NN	0.5165	***NN***	***6.94E-04***	TT
NA18863	199	NN	0.7499	***CC***	***0***	CC
NA19145	200	NN	0.7322	***NN***	***8.01E-04***	CT
NA19137	201	NN	0.7108	***NN***	***8.84E-04***	CT
NA19238	202	CT	0.9382	ct	0.13507218	nn
NA18500	203	NN	0.5292	***NN***	***0***	CT
NA19144	204	NN	0.523	***NN***	***0***	CC
NA19203	205	CC	0.9921	cc	0.022613172	CC
NA19200	206	NN	0.555	tt	0.002976327	CT
NA18855	207	NN	0.5298	tt	0.00107669	CT
NA18505	208	CT	0.9628	ct	0.324876107	CT
NA19202	209	NN	0.5001	***NN***	***0***	CT
NA18501	210	NN	0.5224	***NN***	***0***	CC
NA18861	211	NN	0.6961	***NN***	***0***	CC
NA19193	212	NN	0.7168	***NN***	***0***	CC
NA19143	213	NN	0.4973	***NN***	***0***	CT
NA18517	214	CT	0.9921	ct	0.1624146	CT
NA18862	215	CT	0.7501	***NN***	***0***	CC
NA18856	216	CT	0.9406	ct	0.105275812	nn
NA19239	217		NA	CC	0.161062369	
NA19240	218	CT	0.991	ct	0.26433788	CT
NA18856	219	CT	0.9091	ct	0.254899229	CT
NA18503	220		NA	CC	0.44749673	

	MACGT discrepancy					
	LDA discrepancy					
NA	LDA output were not g					
XY	Manual calls are given					
		2156208				
**Coriell**	**Sample**	**LDA Call**	**LDA quality score**	**MACGT Call**	**MACGT quality score**	**HapMap Call**
NA18871	221		NA	CC	0.360899173	
NA19221	222		NA	CC	0.553456063	
NA19209	223	CT	0.9852	ct	0.184725093	CT
NA19152	224	CC	0.9924	cc	0.355466034	CC
NA18515	225	CT	0.8914	ct	0.186558656	CT
NA19238	226	CT	0.9757	ct	0.542289491	CT
NA19154	227	CT	0.9976	ct	0.642789351	CT
NA19210	228		NA	CC	0.395954811	
NA19211	229	CC	0.999	cc	0.177763484	CC
NA18862	230	CC	0.9272	cc	0.378790997	nn
NA18872	231	CT	0.9937	ct	0.478835696	CT
NA19139	232	CT	0.9953	ct	0.51876731	CT
NA19222	233	CC	0.9697	cc	0.158806563	CC
NA19194	234	CC	0.9722	cc	0.365691393	CC
NA19138	235	CC	0.8875	cc	0.112732625	CC
NA19204	236	CT	0.9684	ct	0.560111431	CT
NA18516	237	CC	0.9771	cc	0.13659537	CC
NA19205	238	CT	0.9918	ct	0.576756002	CT
NA19192	239	CC	0.9897	cc	0.125816253	CC
NA18912	240	CT	0.9553	ct	0.447621851	nn
NA18508	241	CT	0.9978	ct	0.570303256	CT
NA19142	242	CC	0.977	cc	0.103863515	CC
NA18852	243	CT	0.9851	ct	0.248767408	CT
NA18507	244	CC	0.9967	cc	0.210484117	CC
blank	245	NN	0.4406	tt	0.001801603	nn
NA19101	246	CT	0.9874	ct	0.361031849	CT
NA19172	247	TT	0.9686	tt	0.569355573	TT
NA19160	248	CT	0.9565	ct	0.248806139	CT
NA19129	249	CC	0.958	cc	0.222549914	CC
NA18913	250	CT	0.971	ct	0.260419431	CT
NA19120	251	CC	0.9367	cc	0.013805037	CC
NA19159	252	CC	0.9878	cc	0.115336629	CC
NA18523	253	CC	0.9882	cc	0.122098127	CC
NA19102	254	CC	0.9568	***CC***	***0***	CC
NA19092	255		NA	CC	0.611634831	
NA18521	256	CT	0.9944	ct	0.269065631	CT
NA18912	257	CT	0.9572	ct	0.236899772	CT
NA19206	258	TT	0.9959	tt	0.286176162	TT
NA19128	259	CC	0.9833	cc	0.370471675	CC
NA18859	260	CT	0.979	ct	0.351786317	nn
NA19094	261	NN	0.7271	cc	0.48669838	CC
NA19171	262	NN	0.7349	cc	0.372388117	CC
NA18860	263	CC	0.9346	***CC***	***5.26E-04***	CC
NA18858	264	CC	0.8827	cc	0.234217129	CC

	MACGT discrepancy					
	LDA discrepancy					
NA	LDA output were not g					
XY	Manual calls are given					
		2156208				
**Coriell**	**Sample**	**LDA Call**	**LDA quality score**	**MACGT Call**	**MACGT quality score**	**HapMap Call**
NA19099	265	CT	0.9736	ct	0.437801731	CT
NA18506	266	CC	0.9938	cc	0.220423021	CC
NA19131	267	CC	0.9638	cc	0.108447756	CC
NA19116	268	CC	0.9704	cc	0.01073647	CC
NA19161	269	CT	0.9737	ct	0.29911962	CT
NA19140	270	CT	0.9772	ct	0.397197429	CT
NA19207	271	CC	0.9517	cc	0.073753899	CC
NA19141	272	CC	0.9652	cc	0.097565389	CC
NA18854	273	CT	0.9786	ct	0.284940965	CT
NA19098	274	CT	0.9753	ct	0.587300255	CT
NA18522	275	CT	0.9835	ct	0.387176796	CT
NA18914	276	TT	0.9994	tt	0.430297013	TT
NA18853	277	TT	0.9882	tt	0.202950381	TT
NA18859	278	CT	0.9907	ct	0.38437309	CT
NA19100	279	CC	0.9364	cc	0.249740253	CC
NA19093	280	CC	0.9524	cc	0.210437921	CC
NA19132	281	CT	0.9616	ct	0.490413651	CT
NA19130	282	TT	0.998	tt	0.67566273	TT
NA19208	283	CT	0.9809	ct	0.115576938	CT
NA19127	284	CT	0.9671	ct	0.057502032	CT
NA19173	285	CT	0.8134	ct	0.051233875	CT
NA19119	286	CC	0.9899	cc	0.224873587	CC
NA19103	287	CC	0.9739	cc	0.426817315	CC
	Discrepancies	1		4		
	NNs	58		24		
	Automated Calls	269		226		
	Manual Calls	0		43		
	Concordant Calls	194		222		250

	MACGT discrepancy					
	LDA discrepancy					
NA	LDA output were not g					
XY	Manual calls are given					
		2180289				
**Coriell**	**Sample**	**LDA Call**	**LDA quality score**	**MACGT Call**	**MACGT quality score**	**HapMap Call**
NA12753	1		NA	GG	0.426797384	
NA12875	2	CC	0.9364	cc	0.138418669	CC
NA12044	3	CC	0.9476	cc	0.367898955	CC
NA06993	4	CG	0.9187	cg	0.184066868	CG
NA12716	5	CC	0.9304	cc	0.070455649	CC
NA12760	6	CG	0.8748	cg	0.123916613	CG
NA07348	7	CG	0.8404	cg	0.208275775	CG
NA12707	8	CC	0.9714	cc	0.135145164	CC
NA12156	9		NA	CC	0.12891222	
NA11992	10	CC	0.906	cc	0.048171801	CC
NA12239	11	CG	0.9424	cg	0.08474594	CG
NA12878	12	CC	0.9007	cc	0.120996433	CC
NA11993	13	CG	0.8116	cg	0.068222304	CG
NA12750	14	CG	0.8632	cg	0.140361537	CG
NA12146	15	CC	0.8753	cc	0.030900552	CC
NA11839	16	CC	0.9747	cc	0.060656055	CC
NA12156.dup	17	CC	0.9695	cc	0.254202409	nn
NA11829	18		NA	CC	0.380134448	
NA12154	19	CG	0.9032	cg	0.075442211	CG
NA10856	20	CC	0.9283	cc	0.050437955	CC
NA06985	21	CC	0.9577	cc	0.182125516	CC
NA11831	22	CC	0.9877	cc	0.106064911	CC
NA12003.dup	23	CC	0.9405	cc	0.102310859	nn
NA10859	24	CC	0.7916	cc	0.109481544	CC
NA07056	25	CC	0.9647	cc	0.274000656	CC
NA07022	26	GG	0.9978	gg	0.285098612	GG
NA12762	27		NA	CG	0.444957247	
NA12874	28	CC	0.7614	***CC***	***0***	CC
NA12003	29	CC	0.9466	cc	0.053187161	CC
NA10835	30	CG	0.9262	cg	0.405710037	CG
NA11882	31	CC	0.9108	cc	0.104139998	CC
NA07034	32	CG	0.9517	cg	0.255483058	CG
NA12056	33	CC	0.8447	cc	0.010809427	CC
NA12145	34	CC	0.9538	cc	0.29842816	CC
NA07019	35	CG	0.8999	cg	0.198567282	CG
NA06991	36		NA	CG	0.238574173	
NA12761	37	CG	0.9	cg	0.290058814	CG
NA06994	38	GG	0.9979	gg	0.290372376	GG
NA12864	39	CG	0.858	cg	0.190448346	CG
NA07055	40	CC	0.8903	cc	0.211701189	CC
NA10863	41	CC	0.9582	cc	0.102799075	CC
NA12763	42	CG	0.9336	cg	0.297091539	CG
NA10831	43	CG	0.8995	cg	0.216141733	CG
NA11840	44	CC	0.822	cc	0.292325607	CC

	MACGT discrepancy					
	LDA discrepancy					
NA	LDA output were not g					
XY	Manual calls are given					
		**2180289**				
**Coriell**	**Sample**	**LDA Call**	**LDA quality score**	**MACGT Call**	**MACGT quality score**	**HapMap Call**
NA10830	45	CG	0.9122	cg	0.38508433	CG
NA11993.dup	46	CG	0.9335	cg	0.253959696	nn
NA12751	47	CC	0.9552	cc	0.183475598	CC
NA12814	48	NN	0.7179	***cc***	***9.68E-04***	CC
NA10857	49	CC	0.9738	cc	0.383885277	CC
NA07357	50	CC	0.9569	cc	0.401418807	CC
NA07000	51	CC	0.9223	cc	0.018049636	CC
NA12802	52	CC	0.9837	cc	0.128995362	CC
NA10855	53	CC	0.9117	cc	0.012026356	CC
NA11995	54		NA	CG	0.266163276	
NA10839	55	CG	0.8924	cg	0.031302319	CG
NA10847	56	CC	0.8854	cc	0.123633234	CC
NA12006	57	CG	0.9102	cg	0.227439757	CG
NA12144	58	CG	0.9324	cg	0.302555005	CG
NA11832	59	CC	0.9157	***CC***	***0***	CC
NA12813	60	CG	0.9187	cg	0.312393013	CG
blank	61	CC	0.9587	cc	0.460220483	nn
NA12891	62	CC	0.8497	cc	0.179978386	CC
NA12717	63		NA	CC	0.23228509	
NA10861	64	CC	0.8739	cc	0.003020277	CC
NA12004	65	CG	0.7868	cg	0.124004773	CG
NA07048	66	CC	0.8732	cc	0.051461902	CC
NA12801	67	CG	0.7565	cg	0.011477993	CG
NA11830	68	CC	0.788	***CC***	***0***	CC
NA12057	69	CG	0.959	cg	0.229964273	CG
NA11994	70		NA	CC	0.555751129	
NA12740	71	CG	0.9279	cg	0.438082966	CG
NA12005	72	CG	0.8123	cg	0.128056342	CG
NA07345	73	CG	0.8548	cg	0.12993346	CG
NA10854	74	CC	0.8882	cc	0.040545464	CC
NA12872	75	CG	0.8565	cg	0.351436003	CG
NA12865	76	CC	0.9167	cc	0.266487251	CC
NA10860	77		NA	CG	0.248138325	
NA12873	78	CG	0.7593	cg	0.282091529	CG
NA12752	79	CG	0.889	cg	0.409045352	CG
NA10851	80	CG	0.8619	cg	0.167364775	CG
NA07029	81	CG	0.7621	cg	0.243163033	CG
NA12248	82	CG	0.9143	cg	0.450777806	CG
NA12155	83	NN	0.7112	cg	0.058423052	CG
NA10838	84	NN	0.7309	cg	0.064094467	CG
NA12812	85	NN	0.6905	cg	0.093592964	CG
NA12248.dup	86	NN	0.6833	cg	0.076471495	nn
NA12043	87	CC	0.8465	cc	0.237464021	CC
NA12234	88	CG	0.8988	cg	0.176119489	CG

	MACGT discrepancy					
	LDA discrepancy					
NA	LDA output were not g					
XY	Manual calls are given					
		2180289				
**Coriell**	**Sample**	**LDA Call**	**LDA quality score**	**MACGT Call**	**MACGT quality score**	**HapMap Call**
NA12892	89	CG	0.8731	cg	0.32567958	CG
NA10846	90	CC	0.974	cc	0.01700568	CC
NA06993.dup	91	CG	0.8487	cg	0.17894532	nn
NA12249	92	CC	0.9254	cc	0.294046506	CC
NA11881	93	CC	0.8907	cc	0.0529658	CC
NA12815	94	CC	0.95	cc	0.183913118	CC
NA12264	95	CC	0.8712	cc	0.288277095	CC
NA18526	96	GG	0.9975	gg	0.398882629	GG
NA18562	97	CC	0.9239	cc	0.184502398	CC
NA18545	98	CC	0.8783	cc	0.120409299	CC
NA18609	99	CC	0.8609	cc	0.215136311	CC
NA18566	100	CG	0.922	cg	0.234460957	CG
NA18621	101		NA	GG	0.27376173	
NA18577	102	CC	0.8838	cc	0.173288306	CC
NA18635	103	CC	0.9486	cc	0.188105204	CC
NA18524	104	CC	0.9208	cc	0.415006514	CC
NA18537	105	CC	0.8415	cc	0.123210096	CC
NA18572	106	CG	0.9397	cg	0.372882699	CG
NA18552	107	CC	0.8746	cc	0.026063288	CC
NA18563	108	CC	0.9587	cc	0.014188552	CC
NA18594	109	CC	0.9106	cc	0.053139496	nn
NA18624	110	CC	0.9236	cc	0.012852745	CC
NA18592	111	CC	0.9247	cc	0.10270364	CC
NA18529	112	CG	0.9199	cg	0.444295917	CG
NA18603	113	CC	0.7998	cc	0.126584638	nn
NA18547	114	CC	0.8493	cc	0.036945809	CC
NA18611	115	NN	0.7326	***CC***	***0***	CC
NA18570	116	CG	0.9347	cg	0.068208414	CG
NA18622	117	CC	0.9361	cc	0.035499762	CC
NA18579	118	CG	0.9434	cg	0.174592617	CG
NA18636	119	CC	0.8796	cc	0.130938823	CC
NA18558	120	CC	0.8357	cc	0.31876774	CC
NA18540	121	CC	0.8089	cc	0.072273247	CC
NA18609	122	CC	0.8101	cc	0.139362657	nn
NA18555	123	NN	0.7039	cg	0.00999602	CG
NA18612	124	CC	0.9365	cc	0.430521267	CC
NA18573	125	CG	0.7858	cg	0.120992396	CG
NA18632	126	CC	0.8625	cc	0.185847435	CC
NA18593	127	CG	0.8572	cg	0.486733015	CG
NA18532	128	CG	0.8227	cg	0.150651125	CG
NA18605	129	CC	0.9127	cc	0.36489063	CC
NA18550	130	CC	0.9334	cc	0.375890885	CC
NA18603	131	CC	0.9402	cc	0.526608978	CC
NA18571	132	CC	0.9838	cc	0.304313354	CC

	MACGT discrepancy					
	LDA discrepancy					
NA	LDA output were not g					
XY	Manual calls are given					
		2180289				
**Coriell**	**Sample**	**LDA Call**	**LDA quality score**	**MACGT Call**	**MACGT quality score**	**HapMap Call**
NA18623	133	CC	0.7665	cc	0.214062412	CC
NA18582	134	CG	0.9003	cg	0.169069152	CG
NA18637	135	CC	0.9548	cc	0.384747537	CC
NA18561	136	NN	0.7352	cg	0.138423176	CG
NA18542	137	CC	0.9023	cc	0.165205078	CC
NA18608	138	CG	0.9098	cg	0.237917945	CG
NA18564	139	CC	0.8599	cc	0.243859561	CC
NA18620	140	CC	0.852	cc	0.406421848	CC
NA18576	141	NN	0.7417	cg	0.357969043	CG
NA18633	142	CG	0.7618	cg	0.136428731	CG
NA18594	143	CC	0.9373	cc	0.083638302	CC
NA18942	144	CC	0.8671	cc	0.188517512	CC
NA18945	145	CG	0.8551	cg	0.360931293	CG
NA18964	146	CC	0.9373	cc	0.347489737	CC
NA18961	147	CC	0.9061	cc	0.004268844	CC
NA18967	148	CC	0.9029	cc	0.472347801	CC
NA18981	149	CC	0.9009	cc	0.39542133	CC
NA18994	150	CC	0.8207	cc	0.00155987	CC
NA18998	151	CC	0.8075	cc	0.104625109	CC
NA18940	152	CG	0.8046	cg	0.0165791	CG
NA18949	153	CC	0.8523	cc	0.087578403	CC
NA18953	154	CG	0.8993	cg	0.00932907	CG
NA18972	155	CC	0.9438	cc	0.243920991	CC
NA18976	156	CC	0.8512	cc	0.394847605	CC
NA18971	157	CG	0.8751	cg	0.202813288	CG
blank	158	NN	0.7483	***NN***	***0***	nn
NA19000	159	CC	0.9652	cc	0.361821256	CC
NA18951	160	CC	0.9047	cc	0.004919325	nn
NA18948	161	CC	0.9054	cc	0.324941006	CC
NA18968	162	CC	0.9792	cc	0.250285178	CC
NA18965	163	CG	0.9388	cg	0.151212016	CG
NA18978	164	CC	0.9685	cc	0.013333999	CC
NA18974	165	CC	0.949	cc	0.241902495	CC
NA18992	166	GG	0.9986	gg	0.071819149	GG
NA19005	167	CC	0.9946	cc	0.196594359	CC
NA18943	168	NN	0.6216	cg	0.239898834	CG
NA18951	169	CC	0.9751	cc	0.15580423	CC
NA18959	170	CC	0.9841	cc	0.21281664	CC
NA18973	171	CC	0.9639	cc	0.268147836	CC
NA18970	172	CC	0.9662	cc	0.324753299	CC
NA18987	173	CC	0.9538	cc	0.312192722	CC
NA18995	174	CC	0.9825	cc	0.349381563	CC
NA18999	175	CC	0.9612	***CC***	***0***	CC
NA18947	176	CC	0.9549	cc	0.230627151	CC

	MACGT discrepancy					
	LDA discrepancy					
NA	LDA output were not g					
XY	Manual calls are given					
		2180289				
**Coriell**	**Sample**	**LDA Call**	**LDA quality score**	**MACGT Call**	**MACGT quality score**	**HapMap Call**
NA18952	177	CC	0.9802	cc	0.359233164	CC
NA18969	178	CC	0.9802	cc	0.188877402	CC
NA18966	179	CC	0.9822	cc	0.337173043	CC
NA18980	180	CC	0.9557	cc	0.337818652	CC
NA18990	181	CC	0.9338	cc	0.27120706	CC
NA18997	182	CC	0.9645	cc	0.313548268	CC
NA19007	183	CC	0.96	cc	0.203486221	CC
NA18944	184	NN	0.7472	cc	0.01734063	CC
NA18956	185	CC	0.9792	cc	0.253711552	CC
NA18960	186	CC	0.98	cc	0.310368676	CC
NA18975	187	CC	0.9593	cc	0.182754666	CC
NA18995	188	CC	0.9777	cc	0.195401965	nn
NA18991	189	CC	0.987	cc	0.388871865	CC
NA18996	190	CC	0.9742	cc	0.171397307	nn
NA19003	191	CC	0.9764	***CC***	***0***	CC
NA18502	192	CC	0.9253	cc	0.084002943	CC
NA19153	193	NN	0.614	***CG***	***0***	CG
NA18857	194	CC	0.9866	cc	0.082865629	CC
NA19223	195	CC	0.9254	cc	0.139721749	CC
NA19201	196	CC	0.9636	cc	0.155058804	CC
NA18504	197	CC	0.979	cc	0.228488029	CC
NA18870	198	NN	0.6226	cg	0.053162512	CG
NA18863	199	CC	0.935	cc	0.201445621	CC
NA19145	200	CC	0.912	cc	0.152403608	CC
NA19137	201	NN	0.6401	cg	0.042440513	CG
NA19238	202	CC	0.8382	cc	0.134480744	nn
NA18500	203	CC	0.9717	cc	0.011250765	CC
NA19144	204	CC	0.8137	***CC***	***0***	CC
NA19203	205	CC	0.9124	cc	0.311179728	CC
NA19200	206	NN	0.7214	cg	0.018073392	CG
NA18855	207	CC	0.8764	cc	0.114245759	CC
NA18505	208	CC	0.8351	cc	0.032380812	CC
NA19202	209	NN	0.7018	cg	0.090363111	CG
NA18501	210	CC	0.973	cc	0.047970997	CC
NA18861	211	CC	0.9517	cc	0.004191786	CC
NA19193	212	CC	0.9764	cc	0.051796838	CC
NA19143	213	CC	0.9786	***CC***	***0***	CC
NA18517	214	CC	0.8801	cc	0.063622817	CC
NA18862	215	CC	0.9711	cc	0.129832788	CC
NA18856	216	CC	0.8823	***CC***	***0***	nn
NA19239	217	CC	0.9862	cc	0.093642898	CC
NA19240	218	CC	0.9418	cc	0.365935793	CC
NA18856	219	CC	0.9584	***CC***	***0***	CC
NA18503	220	CC	0.993	cc	0.301363082	CC

	MACGT discrepancy					
	LDA discrepancy					
NA	LDA output were not g					
XY	Manual calls are given					
		**2180289**				
**Coriell**	**Sample**	**LDA Call**	**LDA quality score**	**MACGT Call**	**MACGT quality score**	**HapMap Call**
NA18871	221	GG	0.9964	***GG***	***0***	GG
NA19221	222	CC	0.9937	***CC***	***0***	CC
NA19209	223	GG	0.9898	***GG***	***0***	GG
NA19152	224	CG	0.8886	cg	0.307360654	CG
NA18515	225	NN	0.6798	***CG***	***0***	CG
NA19238	226	CC	0.9778	cc	0.142749113	CC
NA19154	227	NN	0.5342	cg	0.002805503	CG
NA19210	228	NN	0.5949	cg	0.001038455	CG
NA19211	229		NA	GG	0.317099171	
NA18862	230	CC	0.9583	cc	0.184602912	nn
NA18872	231		NA	GG	0.210891621	
NA19139	232		NA	GG	0.455461265	
NA19222	233	CG	0.9003	cg	0.182297016	CG
NA19194	234	CC	0.93	cc	0.569204573	CC
NA19138	235	GG	0.9951	gg	0.074191543	GG
NA19204	236	CC	0.9488	cc	0.528507735	CC
NA18516	237	CG	0.772	cg	0.103341955	CG
NA19205	238	CC	0.9607	cc	0.242788267	CC
NA19192	239	CC	0.94	cc	0.008892984	CC
NA18912	240	CC	0.9871	cc	0.375060184	nn
NA18508	241	NN	0.7374	cg	0.113964136	CG
NA19142	242	CC	0.9065	cc	0.028883853	CC
NA18852	243	CC	0.998	cc	0.326261626	CC
NA18507	244	CC	0.9035	cc	0.079455813	CC
blank	245	NN	0.7399	***NN***	***0***	nn
NA19101	246	CC	0.9382	cc	0.392724012	CC
NA19172	247	CC	0.8947	cc	0.067271864	CC
NA19160	248	CG	0.7729	cg	0.019715979	CG
NA19129	249	CG	0.8198	cg	0.025549281	CG
NA18913	250	CC	0.9647	cc	0.064043627	CC
NA19120	251	CG	0.7661	cg	0.004216364	CG
NA19159	252	GG	0.9935	gg	0.014506593	GG
NA18523	253	CC	0.8936	cc	0.408950051	CC
NA19102	254	CC	0.8872	***CC***	***0***	CC
NA19092	255	CG	0.8898	cg	0.100458066	CG
NA18521	256	CC	0.9073	cc	0.251589322	CC
NA18912	257	CC	0.9247	cc	0.102213422	CC
NA19206	258		NA	GG	0.438127664	
NA19128	259	CG	0.8999	cg	0.47499256	CG
NA18859	260	CG	0.9328	cg	0.479020139	nn
NA19094	261	NN	0.7497	***GG***	***0***	GG
NA19171	262	NN	0.4543	***CG***	***0***	CG
NA18860	263	CC	0.9467	cc	0.047823163	CC
NA18858	264	CG	0.95	cg	0.443395356	CG

	MACGT discrepancy					
	LDA discrepancy					
NA	LDA output were not g					
XY	Manual calls are given					
		2180289				
**Coriell**	**Sample**	**LDA Call**	**LDA quality score**	**MACGT Call**	**MACGT quality score**	**HapMap Call**
NA19099	265	CC	0.9493	cc	0.05686744	CC
NA18506	266	CC	0.9329	cc	0.214524411	CC
NA19131	267	CC	0.9793	cc	0.335835867	CC
NA19116	268	CG	0.9393	cg	0.241189107	CG
NA19161	269	GG	1	gg	0.021710942	GG
NA19140	270	CG	0.9618	cg	0.39552563	CG
NA19207	271	CG	0.9094	cg	0.033105175	CG
NA19141	272	CG	0.9234	cg	0.215852308	CG
NA18854	273	CC	0.9583	cc	0.109273487	CC
NA19098	274	CG	0.9317	cg	0.388421048	CG
NA18522	275	CC	0.9642	cc	0.14368353	CC
NA18914	276	CC	0.8786	cc	0.093392539	CC
NA18853	277	CC	0.9402	cc	0.088235105	CC
NA18859	278	CG	0.9268	cg	0.330801344	CG
NA19100	279	CG	0.9472	cg	0.266905658	CG
NA19093	280	CG	0.9339	cg	0.353663779	CG
NA19132	281	CC	0.8867	cc	0.016116111	CC
NA19130	282	CC	0.8701	cc	0.120907231	CC
NA19208	283	GG	1	gg	0.053881308	GG
NA19127	284	CC	0.8502	cc	0.074370439	CC
NA19173	285	CG	0.8059	cg	0.106440312	CG
NA19119	286	CC	0.9731	cc	0.082229239	CC
NA19103	287	CC	0.9878	cc	0.311855855	CC
	Discrepancies	0		0		
	NNs	22		0		
	Automated Calls	273		252		
	Manual Calls	0		21		
	Concordant Calls	233		254		254

	MACGT discrepancy					
	LDA discrepancy					
NA	LDA output were not g					
XY	Manual calls are given					
		**2401810**				
**Coriell**	**Sample**	**LDA Call**	**LDA quality score**	**MACGT Call**	**MACGT quality score**	**HapMap Call**
NA12753	1		NA	TC	0.503784022	
NA12875	2	TT	0.9429	tt	0.076868048	TT
NA12044	3	TC	1	tc	0.644229062	TC
NA06993	4	TC	0.9988	tc	0.420991175	TC
NA12716	5	TT	0.925	tt	0.141962883	TT
NA12760	6	TC	0.9999	tc	0.54076898	TC
NA07348	7	TT	0.9961	tt	0.453490845	TT
NA12707	8	TT	0.9574	tt	0.176989315	TT
NA12156	9		NA	TC	0.412295226	
NA11992	10	TC	0.9997	tc	0.286895994	TC
NA12239	11	TC	0.9954	tc	0.35849516	TC
NA12878	12	TT	1	tt	0.001435238	TT
NA11993	13	TC	1	tc	0.001009184	TC
NA12750	14	TT	0.7843	***TT***	***6.34E-04***	TT
NA12146	15	TC	1	tc	0.079363224	TC
NA11839	16	TT	0.9295	***tt***	***9.10E-04***	TT
NA12156.dup	17	TC	1	tc	0.321613317	nn
NA11829	18		NA	TC	0.399043379	
NA12154	19	TT	0.9999	tt	0.290398134	TT
NA10856	20	TC	1	tc	0.230300799	TC
NA06985	21	TT	0.9998	tt	0.298142372	TT
NA11831	22	TT	0.9598	tt	0.118169962	TT
NA12003.dup	23	TT	0.8323	tt	0.001035448	nn
NA10859	24	TT	0.9988	tt	0.005364867	TT
NA07056	25	TC	1	tc	0.169674525	TC
NA07022	26	TT	1	***TT***	***8.73E-04***	TT
NA12762	27		NA	CC	0.435974158	
NA12874	28	TT	0.9988	tt	0.010991445	TT
NA12003	29	TT	1	***tt***	***9.32E-04***	TT
NA10835	30	TT	0.9984	tt	0.0046484	TT
NA11882	31	TT	0.9668	tt	0.002481587	TT
NA07034	32	TT	0.9615	tt	0.114045062	TT
NA12056	33	TT	0.9999	tt	0.534462401	TT
NA12145	34	TT	0.9999	tt	0.300850776	TT
NA07019	35	TC	1	tc	0.465199562	TC
NA06991	36		NA	TT	0.710241037	
NA12761	37	CC	0.9994	cc	0.072768652	CC
NA06994	38	TC	1	tc	0.118162813	TC
NA12864	39	TT	0.999	tt	0.213702047	TT
NA07055	40	CC	0.9999	cc	0.2486894	CC
NA10863	41	TC	1	tc	0.277538436	TC
NA12763	42	TT	0.9999	tt	0.188583034	TT
NA10831	43	CC	0.9995	cc	0.261693126	CC
NA11840	44	TC	1	tc	0.44323815	TC

	MACGT discrepancy					
	LDA discrepancy					
NA	LDA output were not g					
XY	Manual calls are given					
				**2401810**		
**Coriell**	**Sample**	**LDA Call**	**LDA quality score**	**MACGT Call**	**MACGT quality score**	**HapMap Call**
NA10830	45	TT	0.9973	tt	0.368125204	TT
NA11993.dup	46	TC	1	tc	0.452679362	nn
NA12751	47	TC	1	tc	0.599633095	TC
NA12814	48	TT	0.9994	tt	0.005855522	TT
NA10857	49	TC	1	tc	0.239948158	TC
NA07357	50	TT	0.9999	tt	0.035059075	TT
NA07000	51	TT	0.9996	tt	0.022725606	TT
NA12802	52	TT	1	tt	0.002328197	TT
NA10855	53	TT	1	tt	0.334124309	TT
NA11995	54		NA	TC	0.18696199	
NA10839	55	NN	0.7078	tc	0.005327506	TC
NA10847	56	CC	0.9954	cc	0.217010959	CC
NA12006	57	TT	1	tt	0.011363677	TT
NA12144	58	TC	0.9999	tc	0.394144449	TC
NA11832	59	TC	1	tc	0.331269604	TC
NA12813	60	TC	1	tc	0.372075661	TC
blank	61	TT	1	tt	0.195436996	nn
NA12891	62	TC	0.9999	tc	0.284015403	TC
NA12717	63		NA	TT	0.212526401	
NA10861	64	TC	0.9999	tc	0.229891428	TC
NA12004	65	TC	1	tc	0.300578885	TC
NA07048	66	TC	0.9543	tc	0.035601623	TC
NA12801	67	TC	1	tc	0.198821133	TC
NA11830	68	TT	1	tt	0.001072475	TT
NA12057	69	TC	0.9874	tc	0.29489695	TC
NA11994	70		NA	TT	0.488535061	
NA12740	71	TT	0.9993	tt	0.386849152	TT
NA12005	72	CC	1	cc	0.00943844	CC
NA07345	73	TT	0.9885	***TT***	***6.07E-04***	TT
NA10854	74	TC	0.9998	tc	0.363357121	TC
NA12872	75	TT	1	tt	0.401023274	TT
NA12865	76	TT	1	tt	0.29893929	TT
NA10860	77		NA	TC	0.138775037	
NA12873	78	TT	1	tt	0.413114347	TT
NA12752	79	CC	1	cc	0.172857719	CC
NA10851	80	TC	0.9998	tc	0.626495374	TC
NA07029	81	TT	1	tt	0.069373588	TT
NA12248	82	TC	0.9747	tc	0.436250237	TC
NA12155	83	CC	1	cc	0.018094254	CC
NA10838	84	TC	0.9989	tc	0.412156724	TC
NA12812	85	TC	0.9948	tc	0.253448561	TC
NA12248.dup	86	TC	0.9998	tc	0.453329111	nn
NA12043	87	TT	1	tt	0.346788532	TT
NA12234	88	TT	1	tt	0.406778481	TT

	MACGT discrepancy					
	LDA discrepancy					
NA	LDA output were not g					
XY	Manual calls are given					
		**2401810**				
**Coriell**	**Sample**	**LDA Call**	**LDA quality score**	**MACGT Call**	**MACGT quality score**	**HapMap Call**
NA12892	89	TC	0.8421	tc	0.539142413	TC
NA10846	90	TT	1	tt	0.488298174	TT
NA06993.dup	91	TC	0.9996	tc	0.423271798	nn
NA12249	92	TT	0.9289	*TT*	*4.77E-04*	TT
NA11881	93	TC	1	tc	0.21465998	TC
NA12815	94	TC	0.9999	tc	0.230999382	TC
NA12264	95	CC	1	cc	0.339877467	CC
NA18526	96	TT	1	tt	0.345070435	TT
NA18562	97	TC	1	tc	0.591684641	TC
NA18545	98	TC	1	tc	0.313833384	TC
NA18609	99	TT	0.9969	tt	0.076099905	TT
NA18566	100	TT	0.8529	tt	0.001785586	TT
NA18621	101	TT	0.9986	tt	0.099290319	TT
NA18577	102	TT	0.9985	tt	0.131312721	TT
NA18635	103	TC	0.9998	tc	0.197856886	TC
NA18524	104	TT	0.9947	tt	0.138948237	TT
NA18537	105	TC	1	tc	0.171363956	TC
NA18572	106	TT	0.9909	tt	0.027255379	TT
NA18552	107	TT	0.997	tt	0.004183123	TT
NA18563	108	TT	0.8365	tt	0.042832122	TT
NA18594	109	TC	1	tc	0.234391137	nn
NA18624	110	TT	1	tt	0.388617726	TT
NA18592	111	TT	0.9862	tt	0.025619021	TT
NA18529	112	TT	1	tt	0.118959903	TT
NA18603	113	TT	1	tt	0.010183301	nn
NA18547	114	TT	1	tt	0.01611871	TT
NA18611	115	TT	1	*TT*	*0*	TT
NA18570	116	TT	1	*TT*	*7.20E-04*	TT
NA18622	117	TT	1	tt	0.389790693	TT
NA18579	118	TT	1	*TT*	*8.12E-04*	TT
NA18636	119	TT	1	tt	0.083035623	TT
NA18558	120	TC	0.9995	tc	0.229325975	TC
NA18540	121	TT	1	tt	0.053349221	TT
NA18609	122	TT	1	tt	0.024540126	nn
NA18555	123	TT	1	tt	0.002002264	TT
NA18612	124	TC	0.9899	tc	0.182836382	TC
NA18573	125	TT	1	tt	0.029669024	TT
NA18632	126	TT	1	tt	0.440212288	TT
NA18593	127	TT	1	tt	0.165668403	TT
NA18532	128	TT	1	tt	0.319476691	TT
NA18605	129	TT	1	tt	0.485609111	TT
NA18550	130	TT	1	tt	0.386525426	TT
NA18603	131	TT	1	tt	0.419802801	TT
NA18571	132	TT	1	tt	0.548801065	TT

	MACGT discrepancy					
	LDA discrepancy					
NA	LDA output were not g					
XY	Manual calls are given					
		**2401810**				
**Coriell**	**Sample**	**LDA Call**	**LDA quality score**	**MACGT Call**	**MACGT quality score**	**HapMap Call**
NA18623	133	TT	1	tt	0.369631028	TT
NA18582	134	TC	0.985	tc	0.349289156	TC
NA18637	135	TT	0.9909	tt	0.212730767	TT
NA18561	136	TT	1	tt	0.344338494	TT
NA18542	137	TC	0.9998	tc	0.341112283	TC
NA18608	138	TT	1	tt	0.404420518	TT
NA18564	139	TT	1	tt	0.438722043	TT
NA18620	140	TT	1	tt	0.521623263	TT
NA18576	141	TC	0.9996	tc	0.705493989	TC
NA18633	142	TT	1	tt	0.014280049	TT
NA18594	143	TC	0.9991	tc	0.401800356	TC
NA18942	144	TC	0.9997	tc	0.329809503	TC
NA18945	145	TT	1	tt	0.190855152	TT
NA18964	146	TT	1	tt	0.716906791	TT
NA18961	147	TT	0.9998	tt	0.193508534	TT
NA18967	148	TT	1	tt	0.616127476	TT
NA18981	149	TT	1	tt	0.495783454	TT
NA18994	150	TT	1	tt	0.046140393	TT
NA18998	151	TC	0.9979	tc	0.323131601	TC
NA18940	152	CC	1	cc	0.447932488	CC
NA18949	153	TC	0.9968	tc	0.335507279	TC
NA18953	154	TT	1	tt	0.004066965	TT
NA18972	155	TT	1	tt	0.060824142	TT
NA18976	156	TT	1	tt	0.073039463	TT
NA18971	157	TC	0.9994	tc	0.335012724	TC
blank	158	NN	0.4982	***NN***	***0***	nn
NA19000	159	TT	0.9899	tt	0.278914442	TT
NA18951	160	TC	0.9295	tc	0.159581626	nn
NA18948	161	TT	0.9941	tt	0.047994317	TT
NA18968	162	TT	0.9999	tt	0.14908593	TT
NA18965	163	TC	0.9924	tc	0.268687866	TC
NA18978	164	TC	0.987	tc	0.146820352	TC
NA18974	165	TT	0.9996	tt	0.078575188	TT
NA18992	166	TT	0.9892	***TT***	***0***	TT
NA19005	167	TT	1	tt	0.322215089	TT
NA18943	168	TC	0.9999	tc	0.478996317	TC
NA18951	169	TC	1	tc	0.383971574	TC
NA18959	170	TC	1	tc	0.471680819	TC
NA18973	171	TT	1	tt	0.247965621	TT
NA18970	172	TT	1	tt	0.214303569	TT
NA18987	173	TC	0.9978	tc	0.256430552	TC
NA18995	174	TT	0.9999	tt	0.424384148	TT
NA18999	175	TT	1	tt	0.086687396	TT
NA18947	176	TC	0.9993	tc	0.241703531	TC

	MACGT discrepancy					
	LDA discrepancy					
NA	LDA output were not g					
XY	Manual calls are given					
		2401810				
**Coriell**	**Sample**	**LDA Call**	**LDA quality score**	**MACGT Call**	**MACGT quality score**	**HapMap Call**
NA18952	177	TT	1	tt	0.395556591	TT
NA18969	178	TC	0.997	tc	0.355200637	TC
NA18966	179	TT	1	tt	0.104501885	TT
NA18980	180	TT	0.9999	tt	0.12239862	TT
NA18990	181	TT	1	tt	0.028226689	TT
NA18997	182	TT	1	tt	0.054818019	TT
NA19007	183	TT	0.9995	tt	0.047410507	TT
NA18944	184	TT	0.9008	*TT*	*0*	TT
NA18956	185	TC	0.9999	tc	0.497437077	TC
NA18960	186	TC	0.9999	tc	0.714313301	TC
NA18975	187	TT	1	tt	0.114783602	TT
NA18995	188	TT	1	tt	0.079773381	nn
NA18991	189	TT	0.9992	tt	0.379167708	TT
NA18996	190	TT	1	tt	0.032887778	nn
NA19003	191	TC	1	tc	0.729873421	TC
NA18502	192	TC	1	tc	0.117568821	TC
NA19153	193	TC	0.9992	tc	0.423254029	TC
NA18857	194	TT	1	tt	0.481928103	TT
NA19223	195	TT	0.9998	tt	0.153312798	TT
NA19201	196	TT	1	tt	0.066058461	TT
NA18504	197	TC	0.9998	tc	0.171055885	TC
NA18870	198	TC	0.9977	tc	0.444460683	TC
NA18863	199	TT	0.9884	tt	0.001688099	TT
NA19145	200	TT	0.9999	tt	0.129904152	TT
NA19137	201	TT	0.9922	tt	0.001253345	TT
NA19238	202	TC	0.9969	tc	0.182966856	nn
NA18500	203	TC	0.8993	tc	0.113500072	TC
NA19144	204	TC	0.9987	tc	0.204361612	TC
NA19203	205	TT	1	tt	0.078410076	TT
NA19200	206	TT	1	tt	0.25815385	TT
NA18855	207	TT	0.9998	tt	0.012563504	TT
NA18505	208	CC	1	cc	0.062067832	CC
NA19202	209	TT	1	tt	0.122775059	TT
NA18501	210	TT	0.9998	*TT*	*0*	TT
NA18861	211	TT	0.9992	*TT*	*0*	TT
NA19193	212	TC	0.9336	tc	0.170902596	TC
NA19143	213	TT	1	*TT*	*0*	TT
NA18517	214	TT	1	tt	0.028023538	TT
NA18862	215	TC	0.9958	tc	0.142284751	TC
NA18856	216	TT	0.9995	tt	0.002697402	nn
NA19239	217	TC	0.9361	tc	0.223934739	TC
NA19240	218	TT	1	tt	0.177330531	TT
NA18856	219	TT	1	tt	0.012880722	TT
NA18503	220		NA	CC	0.282096034	

	MACGT discrepancy					
	LDA discrepancy					
NA	LDA output were not g					
XY	Manual calls are given					
		2401810				
**Coriell**	**Sample**	**LDA Call**	**LDA quality score**	**MACGT Call**	**MACGT quality score**	**HapMap Call**
NA18871	221	TC	0.9941	tc	0.389692127	TC
NA19221	222	TC	0.9925	tc	0.502519842	TC
NA19209	223	TC	1	tc	0.411271545	TC
NA19152	224	TC	1	tc	0.27548001	TC
NA18515	225	TC	0.9997	tc	0.534637774	TC
NA19238	226	TC	0.9971	tc	0.443183589	TC
NA19154	227	CC	1	cc	0.411241778	CC
NA19210	228	TT	1	tt	0.102803378	TT
NA19211	229	TC	0.9993	tc	0.257169417	TC
NA18862	230	TC	0.8105	tc	0.155769119	nn
NA18872	231	TC	0.9966	tc	0.342292855	TC
NA19139	232	TT	1	tt	0.387270592	TT
NA19222	233	TC	0.9766	tc	0.295615793	TC
NA19194	234	TT	1	tt	0.612553894	TT
NA19138	235	TT	0.9999	***TT***	***0***	TT
NA19204	236	TT	1	tt	0.024838003	TT
NA18516	237	CC	0.9864	cc	0.270576212	CC
NA19205	238	TT	1	tt	0.10050167	TT
NA19192	239	TT	1	tt	0.038106458	TT
NA18912	240	TT	1	tt	0.272361509	nn
NA18508	241	TC	0.9949	tc	0.343908879	TC
NA19142	242	TT	1	tt	0.023673749	TT
NA18852	243	TT	1	tt	0.621907021	TT
NA18507	244	TT	0.9847	tt	0.029057679	TT
blank	245	NN	0.5	***NN***	***0***	nn
NA19101	246	TC	0.9932	tc	0.451873864	TC
NA19172	247	TC	0.9692	tc	0.200934133	TC
NA19160	248	TC	0.9702	tc	0.178199293	TC
NA19129	249	TC	0.9902	tc	0.195362385	TC
NA18913	250	TC	0.9853	tc	0.247735423	TC
NA19120	251	TC	0.9877	tc	0.07029423	TC
NA19159	252	TT	0.9999	tt	0.016932861	TT
NA18523	253	TC	0.997	tc	0.408228878	TC
NA19102	254	TC	1	tc	0.246101254	TC
NA19092	255	TC	0.9999	tc	0.67187358	TC
NA18521	256	TC	0.9999	tc	0.647272799	TC
NA18912	257	TT	0.9365	tt	0.056785575	TT
NA19206	258	TC	0.9997	tc	0.468797004	TC
NA19128	259	CC	0.9982	cc	0.268381868	CC
NA18859	260	CC	0.9996	cc	0.362225184	nn
NA19094	261	TT	0.9981	tt	0.342153104	TT
NA19171	262	TC	0.9999	tc	0.761616334	TC
NA18860	263	TC	1	tc	0.047525666	TC
NA18858	264	TT	1	tt	0.04222651	TT

	MACGT discrepancy					
	LDA discrepancy					
NA	LDA output were not g					
XY	Manual calls are given					
		**2401810**				
**Coriell**	**Sample**	**LDA Call**	**LDA quality score**	**MACGT Call**	**MACGT quality score**	**HapMap Call**
NA19099	265	TT	1	tt	0.003589656	TT
NA18506	266	TT	0.993	tt	0.306881951	TT
NA19131	267	TT	0.9999	***TT***	***0***	TT
NA19116	268	CC	0.9991	cc	0.072508815	CC
NA19161	269	TC	1	tc	0.141241123	TC
NA19140	270	TC	1	tc	0.1243402	TC
NA19207	271	NN	0.708	tc	0.083686835	TC
NA19141	272	TC	1	tc	0.045231685	TC
NA18854	273	TT	1	tt	0.0277767	TT
NA19098	274	TC	1	tc	0.543709944	TC
NA18522	275	TC	1	tc	0.097151734	TC
NA18914	276	TT	0.9998	tt	0.002369922	TT
NA18853	277	TT	1	tt	0.076897298	TT
NA18859	278	CC	0.9999	cc	0.001331375	CC
NA19100	279	TC	1	tc	0.00123142	TC
NA19093	280	TT	0.991	tt	0.045362142	TT
NA19132	281	TC	1	tc	0.184847086	TC
NA19130	282	TC	1	tc	0.280598838	TC
NA19208	283	TT	0.9879	tt	0.013247036	TT
NA19127	284	TC	0.9783	tc	0.123709854	TC
NA19173	285	CC	1	cc	0.155272963	CC
NA19119	286	TT	0.9998	tt	0.023206629	TT
NA19103	287	TC	1	tc	0.211129144	TC
	Discrepancies	0		0		
	NNs	2		0		
	Automated Calls	277		259		
	Manual Calls	0		18		
	Concordant Calls	256		258		258

	MACGT discrepancy					
	LDA discrepancy					
NA	LDA output were not g					
XY	Manual calls are given					
		**2730648**				
**Coriell**	**Sample**	**LDA Call**	**LDA quality score**	**MACGT Call**	**MACGT quality score**	**HapMap Call**
NA12753	1		NA	TC	0.405277331	
NA12875	2	TT	0.9999	tt	0.373324058	TT
NA12044	3	TC	0.9732	tc	0.430864438	TC
NA06993	4	TC	0.9975	tc	0.384204084	TC
NA12716	5	TT	0.9981	tt	0.410811596	TT
NA12760	6	TT	0.9999	tt	0.407528945	TT
NA07348	7	TT	0.9983	tt	0.358827076	TT
NA12707	8	TC	0.9852	tc	0.126056474	TC
NA12156	9		NA	TT	0.284421084	
NA11992	10	TT	0.7756	tt	0.214864081	TT
NA12239	11	TC	0.9374	tc	0.211949903	TC
NA12878	12	TT	0.9784	tt	0.141400552	TT
NA11993	13	TT	0.951	tt	0.02134912	TT
NA12750	14	TT	0.9866	tt	0.123437135	TT
NA12146	15	TT	0.9723	tt	0.225196208	TT
NA11839	16	TC	0.9993	tc	0.050019709	TC
NA12156.dup	17	TT	0.9987	tt	0.273862097	nn
NA11829	18		NA	TC	0.331585421	
NA12154	19	TT	0.9947	tt	0.33110341	TT
NA10856	20	TC	0.9772	tc	0.288536617	TC
NA06985	21	CC	1	***CC***	***0***	CC
NA11831	22	TT	0.9406	tt	0.23881081	TT
NA12003.dup	23	TT	0.8817	tt	0.156500347	nn
NA10859	24	CC	0.7583	cc	0.043766025	CC
NA07056	25	TT	0.8462	tt	0.165240426	TT
NA07022	26	TT	0.9964	tt	0.40370877	TT
NA12762	27		NA	TT	0.469134962	
NA12874	28	NN	0.5485	tt	0.022991714	TT
NA12003	29	TT	0.9472	tt	0.131764808	TT
NA10835	30	TT	0.9939	tt	0.345225909	TT
NA11882	31	TC	0.999	tc	0.334179348	TC
NA07034	32	TT	0.9042	tt	0.29530119	TT
NA12056	33	NN	0.6232	tt	0.006421581	TT
NA12145	34	TT	0.9904	tt	0.143070935	TT
NA07019	35	TT	0.9757	tt	0.306318934	TT
NA06991	36		NA	CC	0.197221656	
NA12761	37	TC	0.9999	tc	0.084416646	TC
NA06994	38	TT	0.9279	tt	0.141818924	TT
NA12864	39	TT	0.9499	tt	0.087302476	TT
NA07055	40	TT	0.9896	tt	0.564435216	TT
NA10863	41	TT	0.9522	tt	0.178098977	TT
NA12763	42		NA	TC	0.25460784	
NA10831	43	TT	0.7916	tt	0.32705497	TT
NA11840	44	NN	0.5962	***TT***	***0***	TT

	MACGT discrepancy					
	LDA discrepancy					
NA	LDA output were not g					
XY	Manual calls are given					
		**2730648**				
**Coriell**	**Sample**	**LDA Call**	**LDA quality score**	**MACGT Call**	**MACGT quality score**	**HapMap Call**
NA10830	45		NA	TT	0.307192773	
NA11993.dup	46	TT	0.995	tt	0.453560416	nn
NA12751	47	TC	0.9994	tc	0.268013893	TC
NA12814	48	TC	0.9988	tc	0.339612974	TC
NA10857	49	TC	0.9992	tc	0.223168972	TC
NA07357	50	TT	0.9978	tt	0.324453561	TT
NA07000	51	CC	0.8838	cc	0.139089736	CC
NA12802	52	TT	0.9985	tt	0.570340891	TT
NA10855	53	TT	0.9998	tt	0.026845048	TT
NA11995	54		NA	TT	0.153335717	
NA10839	55	TT	0.9958	tt	0.004072974	TT
NA10847	56	TC	0.9996	tc	0.228889274	TC
NA12006	57	TC	0.9998	tc	0.187636441	TC
NA12144	58	CC	0.9819	cc	0.004353774	CC
NA11832	59	TT	0.9998	tt	0.185774675	TT
NA12813	60	TT	0.9999	tt	0.244469183	TT
blank	61	TT	1	tt	0.205970192	nn
NA12891	62	TT	0.9999	tt	0.352807748	TT
NA12717	63		NA	TC	0.41523817	
NA10861	64	TC	0.9995	***TC***	***0***	TC
NA12004	65	TC	0.9938	tc	0.26532927	TC
NA07048	66		NA	TT	0.120873436	
NA12801	67	TC	0.9974	tc	0.149522634	TC
NA11830	68	TT	1	tt	0.235551859	TT
NA12057	69	TT	0.9994	tt	0.130544016	TT
NA11994	70		NA	TC	0.506423492	
NA12740	71	TT	0.9988	tt	0.419323494	TT
NA12005	72	TT	0.9956	tt	0.063662527	TT
NA07345	73	TT	0.9948	tt	0.108498894	TT
NA10854	74	TC	0.979	tc	0.313738326	TC
NA12872	75	TT	0.9999	tt	0.371967233	TT
NA12865	76	TT	1	tt	0.45274375	TT
NA10860	77		NA	TT	0.277217117	
NA12873	78	TT	1	tt	0.339724285	TT
NA12752	79	TT	1	tt	0.500384696	TT
NA10851	80	TT	1	tt	0.354148259	TT
NA07029	81	TC	0.9349	tc	0.252212045	TC
NA12248	82	TT	1	tt	0.300044323	TT
NA12155	83	TC	0.8624	tc	0.073217376	TC
NA10838	84	TT	0.9999	tt	0.551498969	TT
NA12812	85	TC	0.8538	tc	0.144028841	TC
NA12248.dup	86	TT	1	tt	0.380759183	nn
NA12043	87	TC	0.9387	tc	0.281666785	TC
NA12234	88	TC	0.9258	tc	0.332010411	TC

	MACGT discrepancy					
	LDA discrepancy					
NA	LDA output were not g					
XY	Manual calls are given					
		**2730648**				
**Coriell**	**Sample**	**LDA Call**	**LDA quality score**	**MACGT Call**	**MACGT quality score**	**HapMap Call**
NA12892	89	TC	0.983	tc	0.32120927	TC
NA10846	90	TC	0.8773	tc	0.16176252	TC
NA06993.dup	91	TC	0.8928	tc	0.148412338	nn
NA12249	92	TT	0.9984	tt	0.112769202	TT
NA11881	93	CC	0.9992	cc	0.257749525	CC
NA12815	94	TC	0.9461	tc	0.198129745	TC
NA12264	95	TT	0.9996	tt	0.548766314	TT
NA18526	96	CC	0.9999	cc	0.309055527	CC
NA18562	97	TC	0.9974	tc	0.498397536	TC
NA18545	98	TC	0.9981	tc	0.265275831	TC
NA18609	99	NN	0.7375	cc	0.146411876	CC
NA18566	100	CC	0.9574	***CC***	***0***	CC
NA18621	101	CC	0.9118	cc	0.137663702	CC
NA18577	102	TT	0.9746	tt	0.129757708	TT
NA18635	103	NN	0.7481	tc	0.086948637	TC
NA18524	104	TC	0.9697	tc	0.45022787	TC
NA18537	105	TC	0.9983	tc	0.203090409	TC
NA18572	106	TT	0.8147	tt	0.146326188	TT
NA18552	107	CC	0.9996	cc	0.028332344	CC
NA18563	108	TC	0.9958	tc	0.256310096	TC
NA18594	109	CC	0.999	cc	0.07845057	nn
NA18624	110	TC	0.9404	tc	0.311121985	TC
NA18592	111	TC	0.9999	tc	0.158194888	TC
NA18529	112	TC	0.9983	tc	0.371883368	TC
NA18603	113	TT	0.9987	tt	0.199314468	nn
NA18547	114	TC	0.9998	tc	0.216323936	TC
NA18611	115	TC	0.9829	tc	0.079809485	TC
NA18570	116	TC	0.9988	tc	0.3828686	TC
NA18622	117	CC	0.9019	cc	0.155397141	CC
NA18579	118	TC	0.984	tc	0.192586854	TC
NA18636	119	TT	0.999	tt	0.040399718	TT
NA18558	120	TC	0.8856	tc	0.258747235	TC
NA18540	121	CC	1	cc	0.420305096	CC
NA18609	122	CC	0.9996	cc	0.597024219	nn
NA18555	123	TT	0.7523	tt	0.212088065	TT
NA18612	124	TC	0.9811	tc	0.204681382	TC
NA18573	125	TC	0.8327	tc	0.078066091	TC
NA18632	126	TT	1	tt	0.205796318	TT
NA18593	127	TT	1	tt	0.42327593	TT
NA18532	128	TT	1	tt	0.477832963	TT
NA18605	129	TC	0.9176	tc	0.216649698	TC
NA18550	130	TC	0.9578	tc	0.233215335	TC
NA18603	131	TT	1	tt	0.384775774	TT
NA18571	132	TC	0.9378	tc	0.287570746	TC

	MACGT discrepancy					
	LDA discrepancy					
NA	LDA output were not g					
XY	Manual calls are given					
		2730648				
**Coriell**	**Sample**	**LDA Call**	**LDA quality score**	**MACGT Call**	**MACGT quality score**	**HapMap Call**
NA18623	133	CC	0.9999	*CC*	*0*	CC
NA18582	134	TT	1	tt	0.353374999	TT
NA18637	135	TC	0.93	tc	0.129130251	TC
NA18561	136	TT	1	tt	0.020829251	TT
NA18542	137	TT	1	tt	0.584753756	TT
NA18608	138	TT	0.9998	tt	0.338366502	TT
NA18564	139	TT	1	tt	0.507104899	TT
NA18620	140	TC	0.9231	tc	0.294578193	TC
NA18576	141	TT	1	tt	0.54422246	TT
NA18633	142	TC	0.9733	tc	0.427875607	TC
NA18594	143	CC	0.9864	cc	0.09989455	CC
NA18942	144	TT	1	tt	0.489244659	TT
NA18945	145	TT	0.9999	tt	0.32932573	TT
NA18964	146	TC	0.9827	tc	0.399187078	TC
NA18961	147	TC	0.9855	tc	0.260010113	TC
NA18967	148	TC	0.9936	tc	0.415027037	TC
NA18981	149	CC	1	cc	0.270152874	CC
NA18994	150	TC	0.9834	tc	0.369697939	TC
NA18998	151	TC	0.9905	tc	0.17434687	TC
NA18940	152	TT	0.8722	tt	0.294864263	TT
NA18949	153	TC	0.9579	tc	0.141453163	TC
NA18953	154	TT	0.9995	tt	0.205820533	TT
NA18972	155	TC	0.955	tc	0.069101081	TC
NA18976	156	CC	0.9994	cc	0.388090127	CC
NA18971	157	TC	0.9755	tc	0.227814607	TC
blank	158	TC	0.9982	*NN*	*0*	nn
NA19000	159	CC	0.9997	cc	0.380227817	CC
NA18951	160	TC	0.8343	tc	0.079831569	nn
NA18948	161	NN	0.7433	tc	0.086792712	TC
NA18968	162	TT	0.9984	tt	0.521161305	TT
NA18965	163		NA	CC	0.441375927	
NA18978	164		NA	CC	0.484530809	
NA18974	165		NA	CC	0.49526079	
NA18992	166	NN	0.7469	tc	0.12563475	TC
NA19005	167	TC	0.9957	tc	0.156580538	TC
NA18943	168	TT	1	tt	0.380808641	TT
NA18951	169	TC	0.9911	tc	0.12038573	TC
NA18959	170	TC	0.9659	tc	0.256221897	TC
NA18973	171	CC	0.9978	cc	0.330888657	CC
NA18970	172	TT	1	tt	0.438816443	TT
NA18987	173	TT	1	tt	0.168427023	TT
NA18995	174	TC	0.9774	tc	0.355941789	TC
NA18999	175	TC	0.9542	tc	0.316977246	TC
NA18947	176	TC	0.927	tc	0.21547886	TC

	MACGT discrepancy					
	LDA discrepancy					
NA	LDA output were not g					
XY	Manual calls are given					
		**2730648**				
**Coriell**	**Sample**	**LDA Call**	**LDA quality score**	**MACGT Call**	**MACGT quality score**	**HapMap Call**
NA18952	177	TT	1	tt	0.400150004	TT
NA18969	178	TT	1	tt	0.360465065	TT
NA18966	179	TT	0.9999	tt	0.096760975	TT
NA18980	180	TC	0.9741	tc	0.318433862	TC
NA18990	181	TC	0.9498	tc	0.268100563	TC
NA18997	182	TC	0.9733	tc	0.292688065	TC
NA19007	183	TC	0.9838	tc	0.275178032	TC
NA18944	184	TT	0.9894	tt	0.060198758	TT
NA18956	185	TC	0.9871	tc	0.323105061	TC
NA18960	186	CC	0.9861	cc	0.20341132	CC
NA18975	187	TC	0.998	tc	0.206452185	TC
NA18995	188	TC	0.9861	tc	0.483722033	nn
NA18991	189	TC	0.9848	tc	0.482152475	TC
NA18996	190	TC	0.9296	tc	0.00380848	nn
NA19003	191	TT	0.9406	tt	0.497125579	TT
NA18502	192	TC	1	tc	0.001034488	TC
NA19153	193	NN	0.6611	***TT***	***0***	TT
NA18857	194	NN	0.749	tc	0.018286415	TC
NA19223	195	TC	0.9991	tc	0.004072618	TC
NA19201	196	TC	0.7555	tc	0.002997881	TC
NA18504	197	NN	0.748	***TT***	***0***	TT
NA18870	198	TC	0.7612	tc	0.009585008	TC
NA18863	199	NN	0.7461	***TT***	***0***	TT
NA19145	200	TC	0.9997	tc	0.012406633	TC
NA19137	201	TC	0.7546	tc	0.005015593	TC
NA19238	202	TT	1	tt	0.07167073	nn
NA18500	203	NN	0.513	***CC***	***0***	CC
NA19144	204	TC	1	***TC***	***0***	TC
NA19203	205	TT	0.9887	tt	0.011220822	TT
NA19200	206	TC	0.9792	tc	0.001451804	TC
NA18855	207	TC	0.9989	tc	0.069577985	TC
NA18505	208	TC	0.9292	tc	0.183222693	TC
NA19202	209	NN	0.6786	***TT***	***0***	TT
NA18501	210	NN	0.7487	***NN***	***0***	TC
NA18861	211	NN	0.6988	***TT***	***0***	TT
NA19193	212	TT	0.9145	***TT***	***0***	TT
NA19143	213	TT	0.9915	***TT***	***0***	TT
NA18517	214	TC	0.8984	tc	0.069103479	TC
NA18862	215	TC	0.9992	***NN***	***0***	TC
NA18856	216	TC	0.8882	tc	0.056109273	nn
NA19239	217	TT	0.9988	tt	0.007860041	TT
NA19240	218	TT	0.9999	tt	0.266246902	TT
NA18856	219	TC	0.9251	tc	0.13025103	TC
NA18503	220	TT	0.927	tt	0.297557995	TT

	MACGT discrepancy					
	LDA discrepancy					
NA	LDA output were not g					
XY	Manual calls are given					
		**2730648**				
**Coriell**	**Sample**	**LDA Call**	**LDA quality score**	**MACGT Call**	**MACGT quality score**	**HapMap Call**
NA18871	221	TC	0.9999	tc	0.079423754	TC
NA19221	222	TT	0.9995	tt	0.567799567	TT
NA19209	223	NN	0.6008	tt	0.004781907	TT
NA19152	224	TT	0.9949	tt	0.077146719	TT
NA18515	225	TT	0.9759	tt	0.051480208	TT
NA19238	226	TT	0.9854	***TT***	***0***	TT
NA19154	227	TT	0.9943	tt	0.202045779	TT
NA19210	228	NN	0.7275	tt	0.003153787	TT
NA19211	229	TT	1	tt	0.389044632	TT
NA18862	230	TC	0.9281	tc	0.205497556	nn
NA18872	231		NA	CC	0.338826271	
NA19139	232	TC	0.9906	tc	0.320367834	TC
NA19222	233	TT	0.982	tt	0.002809657	TT
NA19194	234	TT	0.9986	tt	0.113271165	TT
NA19138	235	CC	0.9968	cc	0.012480639	CC
NA19204	236	TT	0.9989	tt	0.304398702	TT
NA18516	237	TC	0.999	tc	0.127968633	TC
NA19205	238	TT	0.9997	tt	0.17794929	TT
NA19192	239	TC	0.9991	tc	0.016401741	TC
NA18912	240	TT	0.9979	tt	0.268144557	nn
NA18508	241	TT	0.9984	tt	0.317042389	TT
NA19142	242	TC	0.9924	tc	0.186115871	TC
NA18852	243	TT	0.9997	tt	0.320289876	TT
NA18507	244	TC	0.9716	tc	0.295494748	TC
blank	245	NN	0.3782	***NN***	***0***	nn
NA19101	246	TC	0.9762	tc	0.155125159	TC
NA19172	247	TT	0.9964	tt	0.184431311	TT
NA19160	248	TT	0.9636	tt	0.025317832	TT
NA19129	249	TT	0.9972	tt	0.164445381	TT
NA18913	250	TT	0.7659	***TT***	***6.59E-04***	TT
NA19120	251	TC	0.9393	tc	0.00655117	TC
NA19159	252	NN	0.5891	***TT***	***0***	TT
NA18523	253	TC	0.9068	tc	0.17751972	TC
NA19102	254	TT	0.8619	tt	0.001768668	TT
NA19092	255	TC	0.9992	tc	0.385352287	TC
NA18521	256	TT	0.9996	tt	0.550558708	TT
NA18912	257	TT	0.9993	tt	0.030439561	TT
NA19206	258	TC	0.9987	tc	0.178954774	TC
NA19128	259	TC	0.9933	tc	0.324227448	TC
NA18859	260	TC	0.9986	tc	0.43334927	nn
NA19094	261	TT	0.9945	tt	0.212348376	TT
NA19171	262	TC	0.9985	tc	0.385245182	TC
NA18860	263	TT	0.9801	tt	0.24154424	TT
NA18858	264	TT	0.993	tt	0.146144422	TT

	MACGT discrepancy					
	LDA discrepancy					
NA	LDA output were not g					
XY	Manual calls are given					
		2730648				
**Coriell**	**Sample**	**LDA Call**	**LDA quality score**	**MACGT Call**	**MACGT quality score**	**HapMap Call**
NA19099	265	TT	0.9994	tt	0.248818407	TT
NA18506	266	TC	0.9983	tc	0.164792809	TC
NA19131	267	TC	0.9984	tc	0.21475406	TC
NA19116	268	CC	0.9981	cc	0.064987754	CC
NA19161	269	TT	0.9996	tt	0.221582765	TT
NA19140	270	TT	0.9755	tt	0.116433635	TT
NA19207	271	TT	1	tt	0.21646168	TT
NA19141	272	TC	0.9986	tc	0.030356219	TC
NA18854	273	TC	0.9933	tc	0.229723051	TC
NA19098	274	TT	0.9178	*TT*	*0*	TT
NA18522	275	TT	0.952	tt	0.041000451	TT
NA18914	276	TT	0.9929	tt	0.302307523	TT
NA18853	277	TC	0.7965	tc	0.069527556	TC
NA18859	278	TC	0.9963	tc	0.208098682	TC
NA19100	279	TT	0.9908	tt	0.09642028	TT
NA19093	280	TT	0.9998	tt	0.239814094	TT
NA19132	281	TT	0.9949	tt	0.101812755	TT
NA19130	282	TT	0.9436	tt	0.161818769	TT
NA19208	283	TC	0.9983	tc	0.073885249	TC
NA19127	284	TT	0.9943	tt	0.319087921	TT
NA19173	285	TC	0.9498	tc	0.177366877	TC
NA19119	286	TC	0.9943	tc	0.303135874	TC
NA19103	287	NN	0.7474	tc	0.316566686	TC
	Discrepancies	0		0		
	NNs	18		2		
	Automated Calls	271		249		
	Manual Calls	0		22		
	Concordant Calls	233		250		252

	MACGT discrepancy					
	LDA discrepancy					
NA	LDA output were not g					
XY	Manual calls are given					
		2803543				
**Coriell**	**Sample**	**LDA Call**	**LDA quality score**	**MACGT Call**	**MACGT quality score**	**HapMap Call**
NA12753	1		NA	GG	0.493018583	
NA12875	2	GG	0.9977	gg	0.374793214	GG
NA12044	3	GG	0.9993	gg	0.375322606	GG
NA06993	4	GG	0.9263	gg	0.299469603	GG
NA12716	5	GG	0.9701	gg	0.299314618	GG
NA12760	6	GG	0.9998	gg	0.406758281	GG
NA07348	7	AG	0.9027	ag	0.302939868	AG
NA12707	8	GG	0.9987	gg	0.163273711	GG
NA12156	9		NA	GG	0.198717945	
NA11992	10	AG	0.9618	ag	0.162650187	AG
NA12239	11	GG	0.9782	gg	0.068522728	GG
NA12878	12	GG	0.9573	gg	0.233747674	GG
NA11993	13	AG	0.9994	ag	0.004844491	AG
NA12750	14	GG	0.9608	gg	0.124477508	GG
NA12146	15	GG	0.9178	gg	0.084034649	GG
NA11839	16	GG	0.9962	gg	0.223551133	GG
NA12156.dup	17	GG	0.9999	gg	0.289120935	nn
NA11829	18		NA	GG	0.383669914	
NA12154	19	GG	0.9998	gg	0.002677775	GG
NA10856	20	GG	1	gg	0.285246323	GG
NA06985	21	GG	1	gg	0.002401141	GG
NA11831	22	GG	0.761	gg	0.017813796	GG
NA12003.dup	23	GG	0.8938	gg	0.145319023	nn
NA10859	24	AG	0.984	ag	0.293708598	AG
NA07056	25	GG	1	gg	0.273910401	GG
NA07022	26	GG	0.8833	gg	0.15358761	GG
NA12762	27		NA	GG	0.082300878	
NA12874	28	GG	1	gg	0.207276268	GG
NA12003	29	GG	1	gg	0.427277933	GG
NA10835	30	AG	0.948	ag	0.295700572	AG
NA11882	31	AG	0.9836	ag	0.490240922	AG
NA07034	32	GG	1	gg	0.152360652	GG
NA12056	33	AG	0.944	ag	0.270363725	AG
NA12145	34	AG	0.9982	ag	0.027679556	AG
NA07019	35	GG	0.9487	gg	0.352428917	GG
NA06991	36		NA	GG	0.271475567	
NA12761	37	GG	0.9929	gg	0.338725492	GG
NA06994	38	AG	0.9976	ag	0.166870381	AG
NA12864	39	GG	0.9833	gg	0.385882392	GG
NA07055	40	GG	0.9908	gg	0.484691012	GG
NA10863	41	GG	0.9811	gg	0.375744804	GG
NA12763	42	GG	0.967	gg	0.270060522	GG
NA10831	43	GG	0.9839	gg	0.300822578	GG
NA11840	44	GG	0.9978	gg	0.175102301	GG

	MACGT discrepancy					
	LDA discrepancy					
NA	LDA output were not g					
XY	Manual calls are given					
		2803543				
**Coriell**	**Sample**	**LDA Call**	**LDA quality score**	**MACGT Call**	**MACGT quality score**	**HapMap Call**
NA10830	45	GG	0.9965	gg	0.114301079	GG
NA11993.dup	46	AG	0.9895	ag	0.413029965	nn
NA12751	47	GG	0.9966	gg	0.387779861	GG
NA12814	48	GG	0.9893	gg	0.278409149	GG
NA10857	49	GG	0.9995	gg	0.112923752	GG
NA07357	50	AG	0.9822	ag	0.33947453	AG
NA07000	51	GG	0.9826	gg	0.300908489	GG
NA12802	52	GG	0.9604	gg	0.273224628	GG
NA10855	53	GG	0.8543	gg	0.081074489	GG
NA11995	54		NA	GG	0.094000503	
NA10839	55	GG	0.9226	gg	0.100878494	GG
NA10847	56	GG	0.9984	gg	0.393240222	GG
NA12006	57	GG	0.9834	gg	0.253717095	GG
NA12144	58	GG	0.9974	gg	0.424494544	GG
NA11832	59	GG	0.9992	gg	0.385538582	GG
NA12813	60	GG	0.9996	gg	0.433491683	GG
blank	61	GG	0.9998	gg	0.289787717	nn
NA12891	62	GG	0.999	gg	0.427139279	GG
NA12717	63		NA	GG	0.424274119	
NA10861	64	GG	0.9995	gg	0.410035531	GG
NA12004	65	GG	0.9998	gg	0.326916166	GG
NA07048	66	GG	0.9999	gg	0.331966902	GG
NA12801	67	GG	0.9999	gg	0.44230729	GG
NA11830	68	GG	0.9999	gg	0.480537064	GG
NA12057	69	GG	0.9997	gg	0.388814486	GG
NA11994	70		NA	GG	0.370361111	
NA12740	71	GG	0.999	gg	0.368188292	GG
NA12005	72	GG	0.9977	gg	0.131045849	GG
NA07345	73	GG	0.995	gg	0.186866799	GG
NA10854	74	GG	0.9837	gg	0.203450967	GG
NA12872	75	GG	0.9746	gg	0.368073606	GG
NA12865	76	GG	0.9951	gg	0.568300696	GG
NA10860	77		NA	AG	0.301712428	
NA12873	78	GG	0.9998	gg	0.486727254	GG
NA12752	79	GG	0.9999	gg	0.321081267	GG
NA10851	80	AG	0.9959	ag	0.562814823	AG
NA07029	81	GG	0.9998	gg	0.437855297	GG
NA12248	82	AG	0.9754	ag	0.34244688	AG
NA12155	83	GG	1	gg	0.131373288	GG
NA10838	84	GG	0.9999	gg	0.41017937	GG
NA12812	85	GG	1	gg	0.367519079	GG
NA12248.dup	86	AG	0.8037	ag	0.388741015	nn
NA12043	87	GG	1	gg	0.314060856	GG
NA12234	88	GG	1	gg	0.276595628	GG

	MACGT discrepancy					
	LDA discrepancy					
NA	LDA output were not g					
XY	Manual calls are given					
		**2803543**				
**Coriell**	**Sample**	**LDA Call**	**LDA quality score**	**MACGT Call**	**MACGT quality score**	**HapMap Call**
NA12892	89	GG	1	gg	0.345602693	GG
NA10846	90	AG	0.9699	ag	0.491716242	AG
NA06993.dup	91	GG	0.999	gg	0.023132349	nn
NA12249	92	GG	0.9996	gg	0.166132988	GG
NA11881	93	GG	0.9999	gg	0.360970289	GG
NA12815	94	GG	0.9998	gg	0.264046007	GG
NA12264	95	GG	0.9889	gg	0.200344232	GG
NA18526	96	GG	0.9994	gg	0.395812135	GG
NA18562	97	GG	0.9918	gg	0.34586494	GG
NA18545	98	GG	0.9876	gg	0.253078389	GG
NA18609	99	GG	0.9734	gg	0.248144998	GG
NA18566	100	GG	0.9965	gg	0.099161173	GG
NA18621	101	GG	0.9934	gg	0.29915357	GG
NA18577	102	GG	0.9976	gg	0.101827092	GG
NA18635	103	GG	0.9847	gg	0.217570582	GG
NA18524	104	GG	0.9986	gg	0.319867245	GG
NA18537	105	GG	0.999	gg	0.299707274	GG
NA18572	106	GG	0.9999	gg	0.447748831	GG
NA18552	107	GG	0.9999	gg	0.380883735	GG
NA18563	108	GG	0.9999	gg	0.388768608	GG
NA18594	109	GG	0.9999	gg	0.423301038	nn
NA18624	110	GG	0.9831	gg	0.354312552	GG
NA18592	111	GG	0.9975	gg	0.480446306	GG
NA18529	112	GG	0.9999	gg	0.562058634	GG
NA18603	113	GG	0.9993	gg	0.174904103	nn
NA18547	114	GG	0.9998	gg	0.308064668	GG
NA18611	115	GG	0.9999	gg	0.394516557	GG
NA18570	116	GG	0.9999	gg	0.362787157	GG
NA18622	117	GG	0.9876	gg	0.491947176	GG
NA18579	118	GG	0.9999	gg	0.367593208	GG
NA18636	119	GG	1	gg	0.098515158	GG
NA18558	120	AG	0.9068	ag	0.213413716	AG
NA18540	121	GG	0.9999	gg	0.151748059	GG
NA18609	122	GG	0.9998	gg	0.305900834	nn
NA18555	123	GG	1	gg	0.117062615	GG
NA18612	124	GG	0.9993	gg	0.523332986	GG
NA18573	125	GG	0.9999	gg	0.185003199	GG
NA18632	126	GG	0.9886	gg	0.518358599	GG
NA18593	127	GG	0.9951	gg	0.47083209	GG
NA18532	128	GG	0.9988	gg	0.43786942	GG
NA18605	129	GG	0.9997	gg	0.510674822	GG
NA18550	130	AG	0.9747	ag	0.401161258	AG
NA18603	131	GG	0.9995	gg	0.541041339	GG
NA18571	132	GG	0.9998	gg	0.482025756	GG

	MACGT discrepancy					
	LDA discrepancy					
NA	LDA output were not g					
XY	Manual calls are given					
		2803543				
**Coriell**	**Sample**	**LDA Call**	**LDA quality score**	**MACGT Call**	**MACGT quality score**	**HapMap Call**
NA18623	133	GG	0.9999	gg	0.162830993	GG
NA18582	134	GG	0.9997	gg	0.253637356	GG
NA18637	135	GG	0.9999	gg	0.044881435	GG
NA18561	136	GG	1	gg	0.417611429	GG
NA18542	137	AG	0.8512	ag	0.392194005	AG
NA18608	138	AG	0.8955	ag	0.217397124	AG
NA18564	139	GG	1	gg	0.395730657	GG
NA18620	140	GG	0.9997	gg	0.53509749	GG
NA18576	141	GG	0.9999	gg	0.516028976	GG
NA18633	142	GG	0.9994	gg	0.420574518	GG
NA18594	143	GG	0.9972	gg	0.427375406	GG
NA18942	144	GG	0.9998	gg	0.419311088	GG
NA18945	145	GG	0.9983	gg	0.422339457	GG
NA18964	146	GG	1	gg	0.441478197	GG
NA18961	147	GG	0.999	gg	0.570970595	GG
NA18967	148	GG	0.9996	gg	0.655575396	GG
NA18981	149	GG	0.9999	gg	0.404923923	GG
NA18994	150	GG	0.9999	gg	0.495180766	GG
NA18998	151	GG	0.9994	gg	0.308617357	GG
NA18940	152	AG	0.8796	ag	0.338011405	AG
NA18949	153	GG	0.9999	gg	0.429308869	GG
NA18953	154	GG	0.9999	gg	0.220033552	GG
NA18972	155	GG	1	gg	0.200548869	GG
NA18976	156	GG	0.9993	gg	0.476805192	GG
NA18971	157	GG	0.9999	gg	0.413249397	GG
blank	158	NN	0.5178	***NN***	***0***	nn
NA19000	159	GG	0.999	gg	0.033223386	GG
NA18951	160	GG	0.9999	gg	0.049658951	nn
NA18948	161		NA	AA	0.343145085	
NA18968	162	GG	0.9991	gg	0.408744912	GG
NA18965	163	AG	0.8445	ag	0.402689153	AG
NA18978	164	GG	0.9996	gg	0.184937049	GG
NA18974	165	GG	0.9999	gg	0.167811826	GG
NA18992	166	GG	0.9999	gg	0.030290162	GG
NA19005	167	GG	0.9193	gg	0.183112475	GG
NA18943	168	AG	0.9964	ag	0.314814959	AG
NA18951	169	GG	0.8844	gg	0.08352395	GG
NA18959	170	GG	0.9852	gg	0.279603548	GG
NA18973	171	GG	0.9941	gg	0.277773537	GG
NA18970	172	GG	0.9601	gg	0.216858345	GG
NA18987	173		NA	AG	0.52861203	
NA18995	174	GG	0.9986	gg	0.411027002	GG
NA18999	175	GG	0.9962	gg	0.454305116	GG
NA18947	176	GG	0.9997	gg	0.254259447	GG

	MACGT discrepancy					
	LDA discrepancy					
NA	LDA output were not g					
XY	Manual calls are given					
		2803543				
**Coriell**	**Sample**	**LDA Call**	**LDA quality score**	**MACGT Call**	**MACGT quality score**	**HapMap Call**
NA18952	177	GG	0.9995	gg	0.506399942	GG
NA18969	178	GG	0.9994	gg	0.441341777	GG
NA18966	179	GG	0.9995	gg	0.448218047	GG
NA18980	180	GG	0.9993	gg	0.385959541	GG
NA18990	181	GG	0.9995	gg	0.315214251	GG
NA18997	182	GG	0.9986	gg	0.34054068	GG
NA19007	183	GG	0.9863	gg	0.287951095	GG
NA18944	184	GG	0.9962	gg	0.143489754	GG
NA18956	185	AG	0.9809	ag	0.340845236	AG
NA18960	186	GG	0.9783	gg	0.354031845	GG
NA18975	187		NA	AG	0.497974848	
NA18995	188	GG	0.9868	gg	0.355355812	nn
NA18991	189	GG	0.9917	gg	0.3829444	GG
NA18996	190	GG	0.9736	gg	0.425058777	nn
NA19003	191	GG	0.9641	gg	0.41431026	GG
NA18502	192	GG	0.9875	gg	0.312356696	GG
NA19153	193	GG	0.983	gg	0.218678179	GG
NA18857	194	GG	0.9969	gg	0.440174304	GG
NA19223	195	GG	0.9989	gg	0.483569714	GG
NA19201	196		NA	AA	0.449800814	
NA18504	197	GG	0.9904	gg	0.209653651	GG
NA18870	198	GG	0.999	gg	0.394547781	GG
NA18863	199	AG	0.9359	ag	0.393771237	AG
NA19145	200	GG	0.9889	gg	0.123128911	GG
NA19137	201		NA	GG	0.124518798	
NA19238	202	AA	1	aa	0.233694884	nn
NA18500	203	GG	0.9991	gg	0.223894956	GG
NA19144	204	GG	0.9966	gg	0.001229204	GG
NA19203	205	GG	0.9999	gg	0.383974878	GG
NA19200	206	GG	0.9469	gg	0.447174693	GG
NA18855	207	GG	0.9513	gg	0.242481098	GG
NA18505	208	GG	0.9997	gg	0.514047278	GG
NA19202	209	AG	0.8121	ag	0.311731149	AG
NA18501	210	GG	0.9998	gg	0.286255264	GG
NA18861	211	GG	0.9999	gg	0.228559093	GG
NA19193	212	GG	1	gg	0.02620344	GG
NA19143	213	GG	0.9999	gg	0.253014853	GG
NA18517	214	GG	0.9999	gg	0.325770152	GG
NA18862	215	AG	0.8605	ag	0.363979267	AG
NA18856	216	GG	0.9999	gg	0.070382193	nn
NA19239	217		NA	AG	0.255186932	
NA19240	218	AA	1	aa	0.110065516	AA
NA18856	219	GG	0.9998	gg	0.31727884	GG
NA18503	220	GG	0.8876	gg	0.199551529	GG

	MACGT discrepancy					
	LDA discrepancy					
NA	LDA output were not g					
XY	Manual calls are given					
		**2803543**				
**Coriell**	**Sample**	**LDA Call**	**LDA quality score**	**MACGT Call**	**MACGT quality score**	**HapMap Call**
NA18871	221	GG	0.9304	gg	0.104553205	GG
NA19221	222	GG	0.9646	gg	0.319331845	GG
NA19209	223	GG	0.7686	gg	0.050850068	GG
NA19152	224	AG	0.9761	ag	0.282837247	AG
NA18515	225	AG	0.9915	ag	0.15394624	AG
NA19238	226		NA	AA	0.544066332	
NA19154	227	GG	0.993	gg	0.211011756	GG
NA19210	228	GG	0.9906	gg	0.202376269	GG
NA19211	229	GG	0.9953	gg	0.404390238	GG
NA18862	230	AG	0.968	ag	0.052744886	nn
NA18872	231	GG	0.9959	gg	0.228675612	GG
NA19139	232	GG	0.9939	gg	0.201897231	GG
NA19222	233	GG	0.9923	gg	0.451879883	GG
NA19194	234	GG	0.9979	gg	0.59135129	GG
NA19138	235		NA	AG	0.495602057	
NA19204	236	GG	0.9998	gg	0.457976838	GG
NA18516	237	AG	0.9975	ag	0.175940113	AG
NA19205	238	GG	0.9457	gg	0.323906741	GG
NA19192	239	GG	0.9787	gg	0.26778316	GG
NA18912	240	GG	0.8479	gg	0.063071497	nn
NA18508	241	GG	0.9379	gg	0.288690823	GG
NA19142	242	GG	0.8759	gg	0.113498246	GG
NA18852	243		NA	AA	0.645759926	
NA18507	244	GG	0.9899	gg	0.057352978	GG
blank	245	NN	0.75	*NN*	*0*	nn
NA19101	246	AG	0.9564	ag	0.384340221	AG
NA19172	247	GG	0.9903	gg	0.237332693	GG
NA19160	248	GG	0.9849	gg	0.402334213	GG
NA19129	249	GG	0.9896	gg	0.325280046	GG
NA18913	250		NA	AA	0.266070867	
NA19120	251	GG	0.9999	gg	0.253028354	GG
NA19159	252	GG	0.9999	gg	0.336430081	GG
NA18523	253	AG	0.9467	ag	0.385288589	AG
NA19102	254	GG	0.9996	gg	0.421106513	GG
NA19092	255	GG	0.9854	gg	0.326493128	GG
NA18521	256	GG	0.9687	gg	0.247056156	GG
NA18912	257	GG	0.9908	gg	0.018068623	GG
NA19206	258	GG	0.9889	gg	0.372512802	GG
NA19128	259	AG	0.9845	ag	0.236260916	AG
NA18859	260	AG	0.9656	ag	0.231267989	nn
NA19094	261	GG	0.9943	gg	0.432211201	GG
NA19171	262	AA	1	aa	0.467976883	AA
NA18860	263	GG	0.9983	gg	0.270141606	GG
NA18858	264	GG	0.9995	gg	0.327118329	GG

	MACGT discrepancy					
	LDA discrepancy					
NA	LDA output were not g					
XY	Manual calls are given					
		2803543				
**Coriell**	**Sample**	**LDA Call**	**LDA quality score**	**MACGT Call**	**MACGT quality score**	**HapMap Call**
NA19099	265	GG	0.9994	gg	0.30405863	GG
NA18506	266	GG	0.9987	gg	0.033230867	GG
NA19131	267	AG	0.8899	ag	0.220411981	AG
NA19116	268	GG	0.9997	gg	0.038555538	GG
NA19161	269	GG	0.9994	gg	0.373093643	GG
NA19140	270	GG	0.9993	gg	0.339919025	GG
NA19207	271	AA	1	aa	0.343428391	AA
NA19141	272	GG	0.9988	gg	0.287549866	GG
NA18854	273	AG	0.9415	ag	0.309736081	AG
NA19098	274	GG	0.9993	gg	0.229572834	GG
NA18522	275	GG	0.9992	gg	0.31773555	GG
NA18914	276	AG	0.9175	ag	0.295533733	AG
NA18853	277	GG	0.9997	gg	0.162207069	GG
NA18859	278	AG	0.926	ag	0.283935353	AG
NA19100	279	GG	0.9998	gg	0.022183958	GG
NA19093	280	AG	0.9787	ag	0.503238605	AG
NA19132	281	GG	0.999	gg	0.436848612	GG
NA19130	282	GG	0.9982	gg	0.258325547	GG
NA19208	283	AG	0.9555	ag	0.119624983	AG
NA19127	284	AG	0.8775	ag	0.174552059	AG
NA19173	285	AG	0.8738	ag	0.267185158	AG
NA19119	286	GG	0.9971	gg	0.345734972	GG
NA19103	287	AG	0.9569	ag	0.298303106	AG
	Discrepancies	0		0		
	NNs	0		0		
	Automated Calls	268		266		
	Manual Calls	0		2		
	Concordant Calls	249		249		249

	MACGT discrepancy					
	LDA discrepancy					
NA	LDA output were not g					
XY	Manual calls are given					
		**2835896**				
**Coriell**	**Sample**	**LDA Call**	**LDA quality score**	**MACGT Call**	**MACGT quality score**	**HapMap Call**
NA12753	1		NA	CC	0.616083581	
NA12875	2	CC	1	cc	0.717765163	CC
NA12044	3	CC	1	cc	0.675873595	CC
NA06993	4	CT	1	ct	0.54975539	CT
NA12716	5	TT	1	tt	0.083598571	TT
NA12760	6	CT	0.9988	ct	0.045205443	CT
NA07348	7	CC	1	cc	0.413095396	CC
NA12707	8	CT	0.9999	ct	0.029723697	CT
NA12156	9		NA	CC	0.380116032	
NA11992	10	CT	1	ct	0.199359526	CT
NA12239	11	CT	0.9999	ct	0.122751927	CT
NA12878	12	CC	1	cc	0.508795952	CC
NA11993	13	CT	1	ct	0.094588535	CT
NA12750	14	CC	1	cc	0.540682341	CC
NA12146	15	CC	0.9983	cc	0.368918891	CC
NA11839	16	CT	0.757	ct	0.012237133	CT
NA12156.dup	17	CC	1	cc	0.343832886	nn
NA11829	18		NA	TT	0.319864097	
NA12154	19	TT	1	tt	0.323750905	TT
NA10856	20	CT	1	ct	0.001412376	CT
NA06985	21	CT	0.9999	ct	0.117479576	CT
NA11831	22	CC	0.9984	cc	0.290426815	CC
NA12003.dup	23	CT	1	ct	0.016208127	nn
NA10859	24	CT	1	ct	0.054524559	CT
NA07056	25	CT	1	ct	0.233075371	CT
NA07022	26	CC	0.9996	cc	0.444938185	CC
NA12762	27		NA	CT	0.445086297	
NA12874	28	CT	1	ct	0.162235373	CT
NA12003	29	CT	1	ct	0.405237491	CT
NA10835	30	CC	1	cc	0.501136851	CC
NA11882	31	TT	1	tt	0.385438662	TT
NA07034	32	CT	0.9555	***CT***	***0***	CT
NA12056	33	CC	1	cc	0.311589762	CC
NA12145	34	CC	1	cc	0.530614215	CC
NA07019	35	CT	1	ct	0.375863195	CT
NA06991	36		NA	CT	0.515323238	
NA12761	37	CC	1	cc	0.449325187	CC
NA06994	38	CC	1	cc	0.408952048	CC
NA12864	39	CC	0.9999	cc	0.406971395	CC
NA07055	40	TT	1	tt	0.150368407	TT
NA10863	41	CC	1	cc	0.384136709	CC
NA12763	42	CC	1	cc	0.425653589	CC
NA10831	43	CT	1	ct	0.46089061	CT
NA11840	44	TT	1	tt	0.274886452	TT

	MACGT discrepancy					
	LDA discrepancy					
NA	LDA output were not g					
XY	Manual calls are given					
		**2835896**				
**Coriell**	**Sample**	**LDA Call**	**LDA quality score**	**MACGT Call**	**MACGT quality score**	**HapMap Call**
NA10830	45	CT	1	ct	0.232064179	CT
NA11993.dup	46	CT	1	ct	0.427868986	nn
NA12751	47	TT	1	tt	0.331659449	TT
NA12814	48	CT	1	ct	0.072889872	CT
NA10857	49	CT	1	ct	0.537048547	CT
NA07357	50	CC	1	cc	0.485712153	CC
NA07000	51	CT	0.9998	ct	0.02998533	CT
NA12802	52	TT	0.9999	tt	0.075320816	TT
NA10855	53	CC	0.9201	cc	0.084904604	CC
NA11995	54		NA	CC	0.293618076	
NA10839	55	CC	0.9987	cc	0.155157987	CC
NA10847	56	CC	1	cc	0.36714146	CC
NA12006	57	CC	1	cc	0.0604831	CC
NA12144	58	CT	1	ct	0.410380227	CT
NA11832	59	CT	1	ct	0.069585585	CT
NA12813	60	CC	1	cc	0.233016174	CC
blank	61	CC	0.9995	cc	0.342828983	nn
NA12891	62	CT	1	ct	0.212174005	CT
NA12717	63		NA	CT	0.414703414	
NA10861	64	CC	1	cc	0.618971604	CC
NA12004	65	TT	1	tt	0.025800784	TT
NA07048	66	TT	1	***TT***	***0***	TT
NA12801	67	CT	0.9843	ct	0.003355685	CT
NA11830	68	CC	1	cc	0.169724042	CC
NA12057	69	CT	1	ct	0.160760452	CT
NA11994	70		NA	CC	0.509468623	
NA12740	71	CT	1	ct	0.305340708	CT
NA12005	72	CC	0.8172	***CC***	***0***	CC
NA07345	73	NN	0.7474	***NN***	***0***	CC
NA10854	74	TT	0.7684	***TT***	***0***	TT
NA12872	75	NN	0.7489	***CC***	***0***	CC
NA12865	76	CT	0.9039	***CT***	***0***	CT
NA10860	77		NA	CC	0.430504869	
NA12873	78	CC	0.9924	cc	0.36866389	CC
NA12752	79	NN	0.7496	***CT***	***0***	CT
NA10851	80	CC	1	cc	0.34715303	CC
NA07029	81	CC	0.7806	***CC***	***0***	CC
NA12248	82	CC	0.9324	cc	0.007744902	CC
NA12155	83	NN	0.662	***CT***	***0***	CT
NA10838	84	NN	0.5026	***TT***	***0***	TT
NA12812	85	NN	0.6923	***CT***	***0***	CT
NA12248.dup	86	NN	0.5	***CC***	***0***	nn
NA12043	87	NN	0.4108	***NN***	***0***	CT
NA12234	88	CC	1	cc	0.377636431	CC

	MACGT discrepancy					
	LDA discrepancy					
NA	LDA output were not g					
XY	Manual calls are given					
		2835896				
**Coriell**	**Sample**	**LDA Call**	**LDA quality score**	**MACGT Call**	**MACGT quality score**	**HapMap Call**
NA12892	89	NN	0.4692	*NN*	*0*	CT
NA10846	90	NN	0.7433	*CT*	*0*	CT
NA06993.dup	91	CT	0.9755	*CT*	*0*	nn
NA12249	92	NN	0.7499	*NN*	*0*	CC
NA11881	93	NN	0.75	*CT*	*0*	CT
NA12815	94	NN	0.6115	*CT*	*0*	CT
NA12264	95	CC	0.7855	cc	0.028191443	CC
NA18526	96	CT	0.9997	ct	0.290491809	CT
NA18562	97	CC	1	cc	0.599316813	CC
NA18545	98	CC	1	cc	0.523733447	CC
NA18609	99	CC	1	cc	0.662671271	CC
NA18566	100	CC	1	cc	0.618118296	CC
NA18621	101	CC	1	cc	0.547316316	CC
NA18577	102	CC	1	cc	0.559056618	CC
NA18635	103	CC	1	cc	0.579721389	CC
NA18524	104	TT	1	tt	0.38150592	TT
NA18537	105	CC	1	cc	0.483506293	CC
NA18572	106	CT	1	ct	0.2084923	CT
NA18552	107	CC	1	cc	0.433106178	CC
NA18563	108	CC	1	cc	0.513280469	CC
NA18594	109	CT	1	ct	0.24542091	nn
NA18624	110	CC	1	cc	0.64313577	CC
NA18592	111	CC	1	cc	0.652699023	CC
NA18529	112	CC	1	cc	0.535772437	CC
NA18603	113	CT	1	ct	0.538386699	nn
NA18547	114	CC	1	cc	0.538137163	CC
NA18611	115	CC	1	cc	0.358448	CC
NA18570	116	CC	1	cc	0.448488883	CC
NA18622	117	CC	1	cc	0.598044539	CC
NA18579	118	CT	1	ct	0.281849821	CT
NA18636	119	CC	1	cc	0.428623269	CC
NA18558	120	CC	1	cc	0.07534118	CC
NA18540	121	CC	0.9999	cc	0.173233454	CC
NA18609	122	CC	0.9996	cc	0.173071231	nn
NA18555	123	NN	0.7313	cc	0.039355819	CC
NA18612	124	CC	1	cc	0.424718672	CC
NA18573	125	CC	0.9973	cc	0.018932784	CC
NA18632	126	CC	0.7565	cc	0.00951151	CC
NA18593	127	CC	0.7515	cc	0.032409012	CC
NA18532	128	CC	0.9999	cc	0.058231756	CC
NA18605	129	CC	0.9956	cc	0.165276671	CC
NA18550	130	CC	0.9636	cc	0.063070153	CC
NA18603	131	CT	0.9965	ct	0.240777922	CT
NA18571	132	CT	1	ct	0.162521181	CT

	MACGT discrepancy					
	LDA discrepancy					
NA	LDA output were not g					
XY	Manual calls are given					
		2835896				
**Coriell**	**Sample**	**LDA Call**	**LDA quality score**	**MACGT Call**	**MACGT quality score**	**HapMap Call**
NA18623	133	CC	0.7756	***CC***	***0***	CC
NA18582	134	CC	1	cc	0.427021852	CC
NA18637	135	CC	1	cc	0.4366661	CC
NA18561	136	CC	0.7975	***CC***	***0***	CC
NA18542	137	CC	0.7551	***CC***	***0***	CC
NA18608	138	CC	1	cc	0.446568022	CC
NA18564	139	CC	0.9856	cc	0.090784524	CC
NA18620	140	CT	0.9936	***CT***	***0***	CT
NA18576	141	CC	0.9999	cc	0.121263177	CC
NA18633	142	CC	0.9925	***CC***	***0***	CC
NA18594	143	NN	0.7484	***CT***	***0***	CT
NA18942	144	CC	1	cc	0.429830469	CC
NA18945	145	CC	1	cc	0.6494828	CC
NA18964	146	CC	1	cc	0.332938857	CC
NA18961	147	CC	1	cc	0.459068143	CC
NA18967	148	CC	1	cc	0.424070232	CC
NA18981	149	CC	1	cc	0.280229639	CC
NA18994	150	CC	1	cc	0.314507489	CC
NA18998	151	CC	1	cc	0.359598676	CC
NA18940	152	CC	1	cc	0.309625596	CC
NA18949	153	CT	0.93	ct	0.036065135	CT
NA18953	154	CT	0.9999	***CT***	***0***	CT
NA18972	155	CC	1	cc	0.233683325	CC
NA18976	156	CC	1	cc	0.52205925	CC
NA18971	157	CT	0.9609	ct	0.184658026	CT
blank	158	NN	0.5046	***NN***	***0***	nn
NA19000	159	CC	1	cc	0.509939935	CC
NA18951	160	CC	1	cc	0.448454417	nn
NA18948	161	CC	1	cc	0.367247728	CC
NA18968	162	CC	1	cc	0.533245844	CC
NA18965	163	CT	0.9998	ct	0.127553056	CT
NA18978	164	CC	1	cc	0.50203182	CC
NA18974	165	CC	1	cc	0.227112364	CC
NA18992	166	CC	1	cc	0.338688999	CC
NA19005	167	CC	0.9999	cc	0.502735703	CC
NA18943	168	CT	0.9998	ct	0.273621002	CT
NA18951	169	CC	0.9918	cc	0.330832882	CC
NA18959	170	CC	1	cc	0.535562724	CC
NA18973	171	CC	1	cc	0.573960605	CC
NA18970	172	CC	1	cc	0.552384055	CC
NA18987	173	CC	1	cc	0.462228279	CC
NA18995	174	CT	0.9915	ct	0.382405631	CT
NA18999	175	CT	0.9998	ct	0.128890331	CT
NA18947	176	CC	1	cc	0.343866179	CC

	MACGT discrepancy					
	LDA discrepancy					
NA	LDA output were not g					
XY	Manual calls are given					
		2835896				
**Coriell**	**Sample**	**LDA Call**	**LDA quality score**	**MACGT Call**	**MACGT quality score**	**HapMap Call**
NA18952	177	CT	0.996	ct	0.085540836	CT
NA18969	178	CC	1	cc	0.454865176	CC
NA18966	179	CT	0.9983	ct	0.116816531	CT
NA18980	180	CC	1	cc	0.471367481	CC
NA18990	181	CT	0.9995	ct	0.044962632	CT
NA18997	182	CC	1	cc	0.455096954	CC
NA19007	183	CC	1	cc	0.515503956	CC
NA18944	184	CC	1	cc	0.238855129	CC
NA18956	185	CC	0.9999	cc	0.626355282	CC
NA18960	186	CC	1	cc	0.620474775	CC
NA18975	187	CC	1	cc	0.469684141	CC
NA18995	188	CT	0.9999	ct	0.507092181	nn
NA18991	189	CT	1	ct	0.670062335	CT
NA18996	190	CC	1	cc	0.556081432	nn
NA19003	191	CC	1	cc	0.598970178	CC
NA18502	192	CC	1	cc	0.669829251	CC
NA19153	193	TT	1	tt	0.435875068	TT
NA18857	194	CC	1	cc	0.453607692	CC
NA19223	195		NA	TT	0.668647668	
NA19201	196	CC	1	cc	0.667069083	CC
NA18504	197	CC	1	cc	0.593981216	CC
NA18870	198	CC	1	cc	0.504988508	CC
NA18863	199	CT	0.9995	ct	0.015282739	CT
NA19145	200	CT	0.9994	ct	0.055133439	CT
NA19137	201	CC	1	cc	0.473712895	CC
NA19238	202	CT	1	***CT***	***0***	nn
NA18500	203	CT	0.9999	ct	0.169854553	CT
NA19144	204	CT	0.9999	ct	0.001098687	CT
NA19203	205	CC	1	cc	0.344304241	CC
NA19200	206	CC	1	cc	0.550145737	CC
NA18855	207	CC	1	cc	0.579499478	CC
NA18505	208	CC	1	cc	0.411864372	CC
NA19202	209	CC	1	cc	0.361403946	CC
NA18501	210		NA	TT	0.404631192	
NA18861	211		NA	TT	0.284607441	
NA19193	212	CT	0.9905	ct	0.006754855	CT
NA19143	213	CC	1	cc	0.342182707	CC
NA18517	214	CC	1	cc	0.311422403	CC
NA18862	215	CC	1	cc	0.366542478	CC
NA18856	216	CC	1	***CC***	***0***	nn
NA19239	217	CC	1	cc	0.324280446	CC
NA19240	218	CC	1	cc	0.497613253	CC
NA18856	219	CC	1	cc	0.4277781	CC
NA18503	220	CC	1	cc	0.574552885	CC

	MACGT discrepancy					
	LDA discrepancy					
NA	LDA output were not g					
XY	Manual calls are given					
		**2835896**				
**Coriell**	**Sample**	**LDA Call**	**LDA quality score**	**MACGT Call**	**MACGT quality score**	**HapMap Call**
NA18871	221	CT	1	ct	0.331777572	CT
NA19221	222	CT	1	ct	0.541848432	CT
NA19209	223	CC	0.9997	cc	0.509539805	CC
NA19152	224	CT	1	ct	0.042676941	CT
NA18515	225	CC	0.9601	cc	0.317887604	CC
NA19238	226	CT	0.9999	ct	0.291632916	CT
NA19154	227	CT	1	ct	0.60223902	CT
NA19210	228	CC	1	cc	0.47298619	CC
NA19211	229	CC	1	cc	0.562271518	CC
NA18862	230	CC	1	cc	0.553858522	nn
NA18872	231	CT	1	ct	0.420559892	CT
NA19139	232	CC	1	cc	0.633275515	CC
NA19222	233	CC	1	cc	0.567613974	CC
NA19194	234	CT	0.9999	ct	0.391099719	CT
NA19138	235	CC	1	cc	0.373342615	CC
NA19204	236	CT	0.948	ct	0.007076323	CT
NA18516	237	CC	0.9483	cc	0.263436675	CC
NA19205	238	CC	1	cc	0.470356495	CC
NA19192	239	CT	1	ct	0.02307476	CT
NA18912	240	CT	1	ct	0.437197574	nn
NA18508	241	CT	0.996	ct	0.417761152	CT
NA19142	242	CC	0.9965	cc	0.25133105	CC
NA18852	243	CT	0.9999	ct	0.427976641	CT
NA18507	244	CT	1	ct	0.020758703	CT
blank	245	NN	0.5042	***NN***	***0***	nn
NA19101	246	TT	1	tt	0.29302584	TT
NA19172	247	TT	1	tt	0.321497552	TT
NA19160	248	CC	1	cc	0.563211082	CC
NA19129	249	CC	1	cc	0.277557602	CC
NA18913	250	TT	1	tt	0.599860109	TT
NA19120	251	CT	0.9464	***CT***	***0***	CT
NA19159	252	CC	1	cc	0.416052407	CC
NA18523	253	CC	1	cc	0.511151369	CC
NA19102	254	CC	1	cc	0.29698485	CC
NA19092	255	CC	1	cc	0.626058992	CC
NA18521	256	CC	0.9999	cc	0.475416999	CC
NA18912	257	CT	0.9228	***CT***	***0***	CT
NA19206	258	CC	1	cc	0.543493946	CC
NA19128	259	CC	0.9999	cc	0.549524955	CC
NA18859	260	CT	1	ct	0.01405433	nn
NA19094	261	CC	1	cc	0.678433884	CC
NA19171	262	NN	0.75	ct	0.008610978	CT
NA18860	263	CT	1	ct	0.277546007	CT
NA18858	264	CT	1	ct	0.574042353	CT

	MACGT discrepancy					
	LDA discrepancy					
NA	LDA output were not g					
XY	Manual calls are given					
		**2835896**				
**Coriell**	**Sample**	**LDA Call**	**LDA quality score**	**MACGT Call**	**MACGT quality score**	**HapMap Call**
NA19099	265	CC	1	cc	0.032916072	CC
NA18506	266	CT	0.9921	ct	0.003552727	CT
NA19131	267	TT	1	tt	0.361340889	TT
NA19116	268	TT	1	tt	0.084021756	TT
NA19161	269	CC	1	cc	0.204948593	CC
NA19140	270	CC	1	cc	0.485532017	CC
NA19207	271	CT	0.7828	ct	0.0928283	CT
NA19141	272	CT	1	ct	0.191957757	CT
NA18854	273	TT	1	tt	0.262509983	TT
NA19098	274	CC	1	cc	0.657028723	CC
NA18522	275	CC	1	cc	0.346523136	CC
NA18914	276	CT	0.9994	ct	0.005968069	CT
NA18853	277	TT	1	tt	0.284426541	TT
NA18859	278	CT	1	ct	0.002783174	CT
NA19100	279	CC	1	cc	0.184649603	CC
NA19093	280	CC	1	cc	0.559389791	CC
NA19132	281	CT	1	ct	0.037328491	CT
NA19130	282	CT	0.9997	***CT***	***0***	CT
NA19208	283	CT	1	ct	0.087543167	CT
NA19127	284	CC	1	cc	0.368621404	CC
NA19173	285	CT	0.9927	ct	0.026708295	CT
NA19119	286	CC	1	cc	0.434175257	CC
NA19103	287	CT	1	ct	0.137907957	CT
	Discrepancies	0		0		
	NNs	16		4		
	Automated Calls	275		241		
	Manual Calls	0		34		
	Concordant Calls	241		252		256

	MACGT discrepancy					
	LDA discrepancy					
NA	LDA output were not g					
XY	Manual calls are given					
		**2925067**				
**Coriell**	**Sample**	**LDA Call**	**LDA quality score**	**MACGT Call**	**MACGT quality score**	**HapMap Call**
NA12753	1		NA	TT	0.345145598	
NA12875	2	TC	0.9934	tc	0.311711424	TC
NA12044	3	TT	1	tt	0.529263343	TT
NA06993	4	TT	0.9999	tt	0.376500113	TT
NA12716	5	CC	0.8171	cc	0.007463142	CC
NA12760	6	TC	0.9689	tc	0.250095105	TC
NA07348	7	CC	1	cc	0.266561605	CC
NA12707	8	CC	0.9998	cc	0.097586515	CC
NA12156	9		NA	TT	0.520246468	
NA11992	10	TC	0.9709	tc	0.266111348	TC
NA12239	11	TT	1	tt	0.237318426	TT
NA12878	12	TT	1	tt	0.456077217	TT
NA11993	13	TT	0.9153	tt	0.038885988	TT
NA12750	14	TC	1	tc	0.196403727	TC
NA12146	15	TT	0.9964	tt	0.154130119	TT
NA11839	16	TT	0.9846	tt	0.061741164	TT
NA12156.dup	17	TT	0.9998	tt	0.215555	nn
NA11829	18		NA	TT	0.408440761	
NA12154	19	TC	0.9657	tc	0.111744955	TC
NA10856	20	TT	0.8922	tt	0.227669039	TT
NA06985	21	TT	1	tt	0.325643472	TT
NA11831	22	TC	0.997	tc	0.132333911	TC
NA12003.dup	23	CC	0.8112	cc	0.001015047	nn
NA10859	24	TT	0.9989	tt	0.26940071	TT
NA07056	25	TT	0.9981	tt	0.234181593	TT
NA07022	26	TT	1	tt	0.246131902	TT
NA12762	27		NA	TT	0.260661855	
NA12874	28	TT	0.9904	tt	0.279861476	TT
NA12003	29	CC	1	cc	0.026736256	CC
NA10835	30	TT	0.9952	tt	0.141800616	TT
NA11882	31	TT	0.9978	tt	0.213796252	TT
NA07034	32	TT	0.9344	tt	0.14576622	TT
NA12056	33	TT	1	tt	0.385944226	TT
NA12145	34	TC	0.9981	tc	0.520409965	TC
NA07019	35	TT	0.9997	tt	0.352098582	TT
NA06991	36		NA	TT	0.40380954	
NA12761	37	TT	0.999	tt	0.130773078	TT
NA06994	38	CC	1	***CC***	***0***	CC
NA12864	39	TT	0.9995	tt	0.142603487	TT
NA07055	40	TC	0.9999	tc	0.579860718	TC
NA10863	41	TT	0.9928	tt	0.297330256	TT
NA12763	42	TT	0.9999	tt	0.443248383	TT
NA10831	43	TT	0.9998	tt	0.143477546	TT
NA11840	44	CC	0.9986	cc	0.056795196	CC

	MACGT discrepancy					
	LDA discrepancy					
NA	LDA output were not g					
XY	Manual calls are given					
		2925067				
**Coriell**	**Sample**	**LDA Call**	**LDA quality score**	**MACGT Call**	**MACGT quality score**	**HapMap Call**
NA10830	45	TC	0.9998	*TC*	*0*	TC
NA11993.dup	46	TT	0.9967	tt	0.318868509	nn
NA12751	47	TT	0.9985	tt	0.175652273	TT
NA12814	48	TC	0.9972	tc	0.084061674	TC
NA10857	49	TT	0.9986	tt	0.130064219	TT
NA07357	50	TC	0.9985	tc	0.385267407	TC
NA07000	51	TT	0.998	tt	0.095067128	TT
NA12802	52	TC	0.9999	tc	0.313570395	TC
NA10855	53	CC	0.8021	cc	0.001295679	CC
NA11995	54		NA	TT	0.247250197	
NA10839	55	CC	0.9336	cc	0.001753659	CC
NA10847	56	TT	0.9999	tt	0.070168505	TT
NA12006	57	TC	0.9978	tc	0.328734461	TC
NA12144	58	TC	0.9986	tc	0.316866925	TC
NA11832	59	TC	0.9977	tc	0.371686043	TC
NA12813	60	TC	0.9982	tc	0.343742549	TC
blank	61	CC	0.9991	cc	0.139780006	nn
NA12891	62	TT	1	tt	0.248754778	TT
NA12717	63		NA	CC	0.438205729	
NA10861	64	TT	1	tt	0.111993564	TT
NA12004	65	TC	0.9954	tc	0.203636469	TC
NA07048	66	TC	0.9606	tc	0.284179937	TC
NA12801	67	TC	0.9765	tc	0.096792561	TC
NA11830	68	TC	0.985	tc	0.058226473	TC
NA12057	69	CC	0.9993	cc	0.074374783	CC
NA11994	70		NA	TC	0.294927651	
NA12740	71	TC	0.9997	tc	0.331736278	TC
NA12005	72	NN	0.5256	*NN*	*0*	TC
NA07345	73	NN	0.75	*NN*	*0*	TC
NA10854	74	NN	0.4113	*NN*	*0*	TC
NA12872	75	TT	0.75	*NN*	*0*	TT
NA12865	76	NN	0.5	*NN*	*0*	TC
NA10860	77		NA	TC	0.20141688	
NA12873	78	NN	0.7111	*NN*	*0*	TC
NA12752	79	NN	0.7318	*NN*	*0*	TT
NA10851	80	TC	0.988	tc	0.326925887	TC
NA07029	81	NN	0.5	*NN*	*0*	TC
NA12248	82	NN	0.5146	*NN*	*0*	TT
NA12155	83	NN	0.6275	*NN*	*0*	TT
NA10838	84	NN	0.4168	*NN*	*0*	TC
NA12812	85	NN	0.6687	*NN*	*0*	TT
NA12248.dup	86	NN	0.7333	*NN*	*0*	nn
NA12043	87	NN	0.4912	*NN*	*0*	TT
NA12234	88	TT	1	tt	0.072379945	TT

	MACGT discrepancy					
	LDA discrepancy					
NA	LDA output were not g					
XY	Manual calls are given					
		**2925067**				
**Coriell**	**Sample**	**LDA Call**	**LDA quality score**	**MACGT Call**	**MACGT quality score**	**HapMap Call**
NA12892	89	NN	0.5	***NN***	***0***	TC
NA10846	90	NN	0.75	***NN***	***0***	TC
NA06993.dup	91	NN	0.5043	***NN***	***0***	nn
NA12249	92	NN	0.4855	***NN***	***0***	TC
NA11881	93	NN	0.5174	***NN***	***0***	TT
NA12815	94	NN	0.545	***NN***	***0***	TT
NA12264	95	TT	0.75	***NN***	***0***	TT
NA18526	96	TC	0.8947	tc	0.344626672	TC
NA18562	97	TC	0.997	tc	0.473973187	TC
NA18545	98	CC	0.8776	cc	0.032084817	CC
NA18609	99	TT	0.9968	tt	0.361994179	TT
NA18566	100	TT	0.9968	tt	0.231968192	TT
NA18621	101	TT	0.9991	tt	0.305588252	TT
NA18577	102	TC	0.9862	tc	0.199944837	TC
NA18635	103	TC	0.9977	tc	0.156715908	TC
NA18524	104	TC	0.9943	tc	0.490666942	TC
NA18537	105	TT	0.9633	tt	0.19867569	TT
NA18572	106	TC	0.9693	tc	0.059239668	TC
NA18552	107	TC	0.9933	tc	0.107267244	TC
NA18563	108	TC	0.9904	tc	0.177300843	TC
NA18594	109	TC	0.997	tc	0.140828438	nn
NA18624	110	TT	1	tt	0.395647718	TT
NA18592	111	TT	0.9996	tt	0.444124666	TT
NA18529	112	TC	0.9797	tc	0.102999504	TC
NA18603	113	TT	1	tt	0.321373232	nn
NA18547	114	TC	0.9973	tc	0.315879649	TC
NA18611	115	TC	0.9997	tc	0.098760637	TC
NA18570	116	TT	1	tt	0.196026463	TT
NA18622	117	CC	0.9908	cc	0.294211074	CC
NA18579	118	TC	0.9681	tc	0.028108905	TC
NA18636	119	TT	0.9999	tt	0.238176529	TT
NA18558	120	NN	0.7446	***NN***	***0***	TC
NA18540	121	NN	0.75	***NN***	***0***	TC
NA18609	122	NN	0.5	***NN***	***0***	nn
NA18555	123	NN	0.5	***NN***	***0***	TC
NA18612	124	TT	1	tt	0.174483571	TT
NA18573	125	NN	0.5004	***NN***	***0***	TT
NA18632	126	NN	0.5106	***NN***	***0***	TT
NA18593	127	NN	0.747	***NN***	***0***	TC
NA18532	128	NN	0.5456	***NN***	***0***	TT
NA18605	129	NN	0.5	***NN***	***0***	TC
NA18550	130	NN	0.75	***NN***	***0***	TT
NA18603	131	TT	1	tt	0.446554404	TT
NA18571	132	NN	0.6528	***TT***	***0***	TT

	MACGT discrepancy					
	LDA discrepancy					
NA	LDA output were not g					
XY	Manual calls are given					
		2925067				
**Coriell**	**Sample**	**LDA Call**	**LDA quality score**	**MACGT Call**	**MACGT quality score**	**HapMap Call**
NA18623	133	NN	0.5023	***NN***	***0***	TT
NA18582	134	CC	0.999	cc	0.226526997	CC
NA18637	135	TC	0.9954	***TC***	***0***	TC
NA18561	136	NN	0.5	***NN***	***0***	TT
NA18542	137	TT	0.7501	***NN***	***0***	TT
NA18608	138	TC	0.9443	tc	0.103825645	TC
NA18564	139	NN	0.5041	***NN***	***0***	TT
NA18620	140	NN	0.5011	***NN***	***0***	TT
NA18576	141	NN	0.5004	***NN***	***0***	TT
NA18633	142	NN	0.5	***NN***	***0***	TT
NA18594	143	NN	0.677	***NN***	***0***	TC
NA18942	144	TC	0.9601	tc	0.205430829	TC
NA18945	145	TT	1	tt	0.012022732	TT
NA18964	146	TT	1	tt	0.051431788	TT
NA18961	147	TC	0.9607	tc	0.016117904	TC
NA18967	148	TC	0.9134	tc	0.166461697	TC
NA18981	149	TC	0.9401	tc	0.111540865	TC
NA18994	150	TC	0.9727	tc	0.020465817	TC
NA18998	151	TT	1	***TT***	***0***	TT
NA18940	152	TT	1	tt	0.017850326	TT
NA18949	153	TT	1	tt	0.006619919	TT
NA18953	154	TT	1	***TT***	***0***	TT
NA18972	155	TT	1	tt	0.021177541	TT
NA18976	156	TT	1	tt	0.034427665	TT
NA18971	157	TC	0.9653	tc	0.068165259	TC
blank	158	NN	0.5131	***NN***	***0***	nn
NA19000	159	TC	0.9881	***TC***	***0***	TC
NA18951	160	CC	0.9969	cc	0.24061776	nn
NA18948	161	TC	0.9981	tc	0.010958212	TC
NA18968	162	TT	1	***TT***	***0***	TT
NA18965	163	TC	0.9755	tc	0.008497706	TC
NA18978	164	CC	0.9721	***CC***	***0***	CC
NA18974	165	CC	0.9998	***CC***	***0***	CC
NA18992	166	TT	0.9999	***TT***	***0***	TT
NA19005	167	TT	1	tt	0.046145811	TT
NA18943	168	TT	1	tt	0.001845156	TT
NA18951	169	CC	0.75	***CC***	***0***	CC
NA18959	170	TC	0.9951	tc	0.131165064	TC
NA18973	171	TT	1	tt	0.009088868	TT
NA18970	172	TT	1	tt	0.005210872	TT
NA18987	173	TT	1	tt	0.015944419	TT
NA18995	174	TT	1	tt	0.039329922	TT
NA18999	175	TT	1	***TT***	***5.39E-04***	TT
NA18947	176	TT	1	tt	0.001191858	TT

	MACGT discrepancy					
	LDA discrepancy					
NA	LDA output were not g					
XY	Manual calls are given					
		**2925067**				
**Coriell**	**Sample**	**LDA Call**	**LDA quality score**	**MACGT Call**	**MACGT quality score**	**HapMap Call**
NA18952	177	TC	0.9864	tc	0.072427842	TC
NA18969	178	TC	0.8694	tc	0.051817148	TC
NA18966	179	TC	0.9921	tc	0.049363458	TC
NA18980	180	TT	1	tt	0.004043123	TT
NA18990	181	TT	1	***TT***	***0***	TT
NA18997	182	TC	0.9955	tc	0.074818014	TC
NA19007	183	TT	1	tt	0.001557692	TT
NA18944	184	TC	0.9999	tc	0.002569738	TC
NA18956	185	TT	0.9996	tt	0.025186393	TT
NA18960	186	TT	1	tt	0.012927457	TT
NA18975	187	TT	1	tt	0.001705971	TT
NA18995	188	TT	1	tt	0.029619971	nn
NA18991	189	TC	0.9956	tc	0.117715101	TC
NA18996	190	TT	0.9999	***TT***	***0***	nn
NA19003	191	TC	0.9776	tc	0.072644178	TC
NA18502	192	CC	0.75	***CC***	***0***	CC
NA19153	193	TT	1	tt	0.182821089	TT
NA18857	194	TC	0.9437	tc	0.072161043	TC
NA19223	195	TC	0.9802	tc	0.127346981	TC
NA19201	196	TC	0.9148	tc	0.09191876	TC
NA18504	197	TC	0.96	tc	0.260535958	TC
NA18870	198	TC	0.9891	tc	0.163718438	TC
NA18863	199	TC	0.9662	tc	0.106665783	TC
NA19145	200	TC	0.9421	tc	0.120040438	TC
NA19137	201	CC	0.7835	cc	0.154437039	CC
NA19238	202	TC	0.8279	***TC***	***0***	nn
NA18500	203	TC	0.7593	tc	0.044488632	TC
NA19144	204	TC	0.839	tc	0.002362243	TC
NA19203	205	TC	0.8526	tc	0.108432836	TC
NA19200	206	TC	0.9013	tc	0.196264014	TC
NA18855	207	TT	0.9998	tt	0.030558696	TT
NA18505	208	TC	0.9418	tc	0.002278126	TC
NA19202	209	TC	0.9043	tc	0.066816853	TC
NA18501	210	TT	1	***TT***	***0***	TT
NA18861	211	CC	0.931	***CC***	***0***	CC
NA19193	212	TC	0.9688	tc	0.040368026	TC
NA19143	213	TT	1	***TT***	***7.85E-04***	TT
NA18517	214	TC	0.88	***TC***	***0***	TC
NA18862	215	TC	0.8016	tc	0.094192685	TC
NA18856	216	CC	0.9495	***CC***	***0***	nn
NA19239	217	TC	0.7536	tc	0.059506678	TC
NA19240	218	TC	0.8246	tc	0.001286284	TC
NA18856	219	CC	0.9772	***CC***	***0***	CC
NA18503	220	CC	0.9313	cc	0.019200759	CC

	MACGT discrepancy					
	LDA discrepancy					
NA	LDA output were not g					
XY	Manual calls are given					
		2925067				
**Coriell**	**Sample**	**LDA Call**	**LDA quality score**	**MACGT Call**	**MACGT quality score**	**HapMap Call**
NA18871	221	TC	0.999	tc	0.379318895	TC
NA19221	222	TC	0.9866	tc	0.48481779	TC
NA19209	223	CC	0.8436	cc	0.019488973	CC
NA19152	224	TT	1	tt	0.286310892	TT
NA18515	225	TC	0.9998	tc	0.262045781	TC
NA19238	226	TC	0.9967	tc	0.362070615	TC
NA19154	227	TT	1	tt	0.405650198	TT
NA19210	228	TT	1	tt	0.251515616	TT
NA19211	229	TC	0.9986	tc	0.187339206	TC
NA18862	230	TC	0.7807	tc	0.016961302	nn
NA18872	231	TC	0.958	tc	0.246820554	TC
NA19139	232	TC	0.9912	tc	0.221210449	TC
NA19222	233	CC	0.9881	cc	0.124240854	CC
NA19194	234	TT	1	tt	0.198840735	TT
NA19138	235	TC	0.9969	tc	0.099801981	TC
NA19204	236	TT	1	tt	0.040057631	TT
NA18516	237	TT	1	tt	0.171818929	TT
NA19205	238	TC	0.9648	tc	0.530894866	TC
NA19192	239	TT	1	tt	0.076895926	TT
NA18912	240	TT	1	tt	0.341755799	nn
NA18508	241	TC	0.9899	tc	0.545140261	TC
NA19142	242	TC	0.8975	tc	0.208886172	TC
NA18852	243	CC	1	cc	0.211909584	CC
NA18507	244	TT	0.9987	tt	0.098034661	TT
blank	245	TT	0.75	***NN***	***0***	nn
NA19101	246	TC	0.7925	tc	0.173941782	TC
NA19172	247	TT	1	tt	0.024125452	TT
NA19160	248	TC	0.9362	tc	0.264704121	TC
NA19129	249	TC	0.8849	tc	0.155171682	TC
NA18913	250	TT	0.9999	tt	0.219402626	TT
NA19120	251	TC	0.9969	tc	0.069001578	TC
NA19159	252	TT	1	tt	0.227086811	TT
NA18523	253	TT	1	tt	0.008055439	TT
NA19102	254	TT	0.9999	tt	0.010470474	TT
NA19092	255	TT	1	tt	0.135827595	TT
NA18521	256	TT	0.9979	tt	0.12510642	TT
NA18912	257	TT	0.9976	tt	0.030364692	TT
NA19206	258	TT	1	tt	0.42629979	TT
NA19128	259	TC	0.9894	tc	0.508231977	TC
NA18859	260	TC	0.9899	tc	0.49651691	nn
NA19094	261	TT	1	tt	0.638192417	TT
NA19171	262	TT	0.9999	tt	0.182438824	TT
NA18860	263	TT	1	tt	0.116758912	TT
NA18858	264	TC	0.9959	***TC***	***0***	TC

	MACGT discrepancy					
	LDA discrepancy					
NA	LDA output were not g					
XY	Manual calls are given					
		2925067				
**Coriell**	**Sample**	**LDA Call**	**LDA quality score**	**MACGT Call**	**MACGT quality score**	**HapMap Call**
NA19099	265	TC	0.9971	***TC***	***0***	TC
NA18506	266	TT	0.9997	***TT***	***0***	TT
NA19131	267	TT	1	tt	0.138351897	TT
NA19116	268	TT	1	***TT***	***0***	TT
NA19161	269	TT	1	tt	0.079077301	TT
NA19140	270	TC	0.9988	***TC***	***0***	TC
NA19207	271	NN	0.6758	***TC***	***0***	TC
NA19141	272	TT	1	tt	0.016643691	TT
NA18854	273	CC	0.9495	cc	0.207307025	CC
NA19098	274	CC	1	cc	0.095894998	CC
NA18522	275	TT	1	***TT***	***0***	TT
NA18914	276	TT	1	***TT***	***0***	TT
NA18853	277	TC	0.8874	***TC***	***0***	TC
NA18859	278	TC	0.9987	***TC***	***0***	TC
NA19100	279	TC	0.9926	***TC***	***0***	TC
NA19093	280	TT	1	***TT***	***0***	TT
NA19132	281	TT	1	tt	0.021077342	TT
NA19130	282	TT	0.9992	tt	0.057270538	TT
NA19208	283	TT	0.9841	tt	0.186436723	TT
NA19127	284	TC	0.782	***TC***	***0***	TC
NA19173	285	TT	1	tt	0.250783165	TT
NA19119	286	TC	0.9981	***TC***	***0***	TC
NA19103	287	TC	0.9998	tc	0.328783536	TC
	Discrepancies	0		0		
	NNs	37		39		
	Automated Calls	278		200		
	Manual Calls	0		78		
	Concordant Calls	224		223		259

	MACGT discrepancy					
	LDA discrepancy					
NA	LDA output were not g					
XY	Manual calls are given					
		**2938675**				
**Coriell**	**Sample**	**LDA Call**	**LDA quality score**	**MACGT Call**	**MACGT quality score**	**HapMap Call**
NA12753	1		NA	CA	0.404414441	
NA12875	2	CC	1	***CC***	***0***	CC
NA12044	3	CA	1	ca	0.400680738	CA
NA06993	4	CA	1	ca	0.372963462	CA
NA12716	5	CA	0.9999	ca	0.209687648	CA
NA12760	6	CA	1	ca	0.235190357	CA
NA07348	7	CA	0.9999	ca	0.317228994	CA
NA12707	8	CA	1	ca	0.100739956	CA
NA12156	9		NA	CA	0.1172005	
NA11992	10	CA	1	ca	0.056815969	CA
NA12239	11	CA	1	ca	0.216151118	CA
NA12878	12	CC	0.9961	cc	0.207838655	CC
NA11993	13	CC	0.999	cc	0.05362265	CC
NA12750	14	CA	1	ca	0.134378058	CA
NA12146	15	AA	1	aa	0.002846793	AA
NA11839	16	CA	1	ca	0.055959011	CA
NA12156.dup	17	CA	1	ca	0.406587706	nn
NA11829	18		NA	AA	0.308050474	
NA12154	19	CC	0.9991	cc	0.012624238	CC
NA10856	20	CA	0.9999	ca	0.208272607	CA
NA06985	21	CA	1	ca	0.2373299	CA
NA11831	22	CC	0.9993	cc	0.079237084	CC
NA12003.dup	23	CA	0.9999	ca	0.072546387	nn
NA10859	24	CA	0.9994	ca	0.102443831	CA
NA07056	25	CA	1	ca	0.17037744	CA
NA07022	26	CC	0.9977	cc	0.051006014	CC
NA12762	27		NA	AA	0.420084181	
NA12874	28	CC	0.9922	***CC***	***0***	CC
NA12003	29	CA	0.9999	ca	0.068384473	CA
NA10835	30	CA	0.9995	ca	0.186345445	CA
NA11882	31	CC	1	cc	0.151032479	CC
NA07034	32	CC	0.9395	***CC***	***0***	CC
NA12056	33	CA	0.9991	ca	0.108031154	CA
NA12145	34	CC	0.9978	cc	0.360265338	CC
NA07019	35	CC	1	cc	0.142039474	CC
NA06991	36		NA	AA	0.371115461	
NA12761	37	CC	1	cc	0.093960272	CC
NA06994	38	CA	0.9989	ca	0.096037701	CA
NA12864	39	CA	0.9996	ca	0.137441257	CA
NA07055	40	AA	0.999	aa	0.030239931	AA
NA10863	41	CA	0.9999	ca	0.311308763	CA
NA12763	42	CA	0.9999	ca	0.40524248	CA
NA10831	43	AA	1	aa	0.116832287	AA
NA11840	44	CC	0.8419	***CC***	***0***	CC

	MACGT discrepancy					
	LDA discrepancy					
NA	LDA output were not g					
XY	Manual calls are given					
		**2938675**				
**Coriell**	**Sample**	**LDA Call**	**LDA quality score**	**MACGT Call**	**MACGT quality score**	**HapMap Call**
NA10830	45	CC	1	cc	0.190774681	CC
NA11993.dup	46	CC	1	cc	0.071472155	nn
NA12751	47	CA	0.9996	ca	0.353441144	CA
NA12814	48	CA	0.9997	ca	0.029694197	CA
NA10857	49	CA	1	ca	0.518709734	CA
NA07357	50	CC	1	cc	0.095125328	CC
NA07000	51	CC	1	cc	0.00459015	CC
NA12802	52	CA	1	ca	0.012759176	CA
NA10855	53	CC	1	cc	0.4062335	CC
NA11995	54		NA	CC	0.08313289	
NA10839	55	CA	0.9974	ca	0.138119645	CA
NA10847	56	AA	0.9993	aa	0.106914227	AA
NA12006	57	CC	0.9472	cc	0.117609392	CC
NA12144	58	CC	0.9764	cc	0.173596707	CC
NA11832	59	CC	1	cc	0.006344349	CC
NA12813	60	CC	1	cc	0.007828925	CC
blank	61	CC	0.9997	***CC***	***0***	nn
NA12891	62	CC	1	cc	0.029862595	CC
NA12717	63		NA	CA	0.365392627	
NA10861	64	CC	1	cc	0.123199365	CC
NA12004	65	CA	0.9993	ca	0.130760417	CA
NA07048	66	CA	1	ca	0.191422552	CA
NA12801	67	CA	0.9996	***CA***	***0***	CA
NA11830	68	CC	1	***CC***	***0***	CC
NA12057	69	CA	1	ca	0.002544415	CA
NA11994	70		NA	CC	0.548514984	
NA12740	71	CA	0.9998	ca	0.314391587	CA
NA12005	72	CA	1	ca	0.247543694	CA
NA07345	73	AA	0.9715	aa	0.018819281	AA
NA10854	74	CC	0.9895	cc	0.195827159	CC
NA12872	75	AA	0.9814	***AA***	***0***	AA
NA12865	76	CC	1	cc	0.379181113	CC
NA10860	77		NA	CC	0.380340741	
NA12873	78	CC	1	cc	0.275705462	CC
NA12752	79	CA	1	ca	0.252215818	CA
NA10851	80	CC	1	cc	0.467114992	CC
NA07029	81	CA	1	ca	0.145124233	CA
NA12248	82	CA	1	ca	0.167116954	CA
NA12155	83	CA	1	ca	0.055641328	CA
NA10838	84	CA	0.9998	ca	0.372588322	CA
NA12812	85	CA	0.9997	ca	0.329626093	CA
NA12248.dup	86	CA	0.9996	ca	0.034808692	nn
NA12043	87	AA	0.9996	aa	0.23762823	AA
NA12234	88	CA	1	***CA***	***8.99E-04***	CA

	MACGT discrepancy					
	LDA discrepancy					
NA	LDA output were not g					
XY	Manual calls are given					
		2938675				
**Coriell**	**Sample**	**LDA Call**	**LDA quality score**	**MACGT Call**	**MACGT quality score**	**HapMap Call**
NA12892	89	CC	1	cc	0.254343886	CC
NA10846	90	CC	1	***CC***	***0***	CC
NA06993.dup	91	CA	1	ca	0.23101359	nn
NA12249	92	CA	1	ca	0.309808512	CA
NA11881	93	AA	0.9958	aa	0.002005166	AA
NA12815	94	AA	0.9999	aa	0.069105236	AA
NA12264	95	CC	1	cc	0.533162325	CC
NA18526	96	CA	1	ca	0.139363841	CA
NA18562	97	CC	1	cc	0.257227346	CC
NA18545	98	CC	1	cc	0.07146777	CC
NA18609	99	CC	0.9901	cc	0.249638861	CC
NA18566	100	CA	0.9999	ca	0.268590635	CA
NA18621	101	CC	1	cc	0.292479917	CC
NA18577	102	CC	1	cc	0.306239727	CC
NA18635	103	CC	1	cc	0.247573399	CC
NA18524	104	AA	0.9996	aa	0.058037328	AA
NA18537	105	CA	0.9999	ca	0.128822097	CA
NA18572	106	CC	1	cc	0.100574894	CC
NA18552	107	CC	1	cc	0.003450434	CC
NA18563	108	CC	1	cc	0.246309468	CC
NA18594	109	AA	0.9992	aa	0.063840819	nn
NA18624	110	CC	1	cc	0.42445748	CC
NA18592	111	CC	0.9924	cc	0.271905695	CC
NA18529	112	CC	1	cc	0.514003972	CC
NA18603	113	CC	0.9828	cc	0.364250812	nn
NA18547	114	AA	0.9994	aa	0.125700644	AA
NA18611	115	CC	0.9843	cc	0.00664936	CC
NA18570	116	CA	0.9997	ca	0.080963187	CA
NA18622	117	CC	1	cc	0.39771925	CC
NA18579	118	CA	1	***CA***	***0***	CA
NA18636	119	CC	1	cc	0.384949072	CC
NA18558	120	CC	0.945	cc	0.548037084	CC
NA18540	121	CC	0.9296	cc	0.070385369	CC
NA18609	122	CC	0.9316	cc	0.247164465	nn
NA18555	123	CA	1	ca	0.044490821	CA
NA18612	124	CA	1	ca	0.260192199	CA
NA18573	125	AA	0.9697	***AA***	***0***	AA
NA18632	126	CA	1	ca	0.22679572	CA
NA18593	127	CC	1	cc	0.578955861	CC
NA18532	128	CC	1	cc	0.4255278	CC
NA18605	129	CC	1	cc	0.498228347	CC
NA18550	130	CC	1	cc	0.447971361	CC
NA18603	131	CC	0.9875	cc	0.400768983	CC
NA18571	132	CC	1	cc	0.540373785	CC

	MACGT discrepancy					
	LDA discrepancy					
NA	LDA output were not g					
XY	Manual calls are given					
		**2938675**				
**Coriell**	**Sample**	**LDA Call**	**LDA quality score**	**MACGT Call**	**MACGT quality score**	**HapMap Call**
NA18623	133	CC	1	cc	0.108385905	CC
NA18582	134	CC	1	cc	0.113649975	CC
NA18637	135	CA	0.9999	ca	0.042087604	CA
NA18561	136	CC	1	cc	0.143417305	CC
NA18542	137	CA	0.9998	ca	0.388316975	CA
NA18608	138	CC	0.9743	cc	0.242751168	CC
NA18564	139	CA	0.9999	ca	0.032793166	CA
NA18620	140	CA	0.9999	ca	0.374930346	CA
NA18576	141	CC	1	cc	0.313979964	CC
NA18633	142	CC	1	cc	0.215228076	CC
NA18594	143	AA	0.9996	***AA***	***0***	AA
NA18942	144	CC	1	cc	0.238925153	CC
NA18945	145	CC	0.9989	cc	0.123079616	CC
NA18964	146	CC	1	cc	0.244527913	CC
NA18961	147	CC	1	cc	0.106504736	CC
NA18967	148	CC	1	cc	0.206166156	CC
NA18981	149	CC	1	cc	0.375549698	CC
NA18994	150	CC	1	cc	0.34154543	CC
NA18998	151	CC	1	***CC***	***0***	CC
NA18940	152	CC	0.9941	***cc***	***9.82E-04***	CC
NA18949	153	CA	1	ca	0.205208701	CA
NA18953	154	CA	1	***CA***	***0***	CA
NA18972	155	CC	0.9889	cc	0.063573197	CC
NA18976	156	CC	1	cc	0.076456661	CC
NA18971	157	CC	0.9976	cc	0.0229831	CC
blank	158	NN	0.4985	***NN***	***0***	nn
NA19000	159	CA	0.9999	ca	0.214173211	CA
NA18951	160	CC	1	cc	0.393109918	nn
NA18948	161	CA	0.9999	ca	0.05573026	CA
NA18968	162	CC	1	cc	0.204386914	CC
NA18965	163	CC	1	cc	0.001987041	CC
NA18978	164	CC	1	cc	0.21757542	CC
NA18974	165	CC	1	cc	0.007507537	CC
NA18992	166	CC	1	***CC***	***0***	CC
NA19005	167	CC	0.9981	cc	0.429095003	CC
NA18943	168	CC	1	cc	0.196343079	CC
NA18951	169	CC	1	cc	0.301347561	CC
NA18959	170	CC	1	cc	0.103053969	CC
NA18973	171	CC	0.9968	cc	0.007594944	CC
NA18970	172	CC	0.9948	cc	0.036602762	CC
NA18987	173	CC	0.9956	cc	0.109565744	CC
NA18995	174	CC	0.9965	cc	0.325979402	CC
NA18999	175	CC	0.9946	cc	0.039264944	CC
NA18947	176	CC	0.9049	cc	0.013745277	CC

	MACGT discrepancy					
	LDA discrepancy					
NA	LDA output were not g					
XY	Manual calls are given					
		2938675				
**Coriell**	**Sample**	**LDA Call**	**LDA quality score**	**MACGT Call**	**MACGT quality score**	**HapMap Call**
NA18952	177	CC	0.9981	cc	0.009086048	CC
NA18969	178	CC	0.9954	*CC*	*0*	CC
NA18966	179	CC	1	cc	0.07051705	CC
NA18980	180	CC	0.9833	cc	0.030817605	CC
NA18990	181	CC	0.9923	cc	0.004363478	CC
NA18997	182	AA	1	aa	0.162554636	AA
NA19007	183	CC	1	*CC*	*0*	CC
NA18944	184	CA	0.9972	ca	0.009122043	CA
NA18956	185	CA	0.9999	ca	0.235189339	CA
NA18960	186	CC	1	cc	0.43492126	CC
NA18975	187	CC	0.9866	cc	0.112449989	CC
NA18995	188	CC	0.9956	cc	0.387581351	nn
NA18991	189	CC	1	cc	0.35860902	CC
NA18996	190	CC	1	*CC*	*0*	nn
NA19003	191	CC	0.9836	cc	0.213045463	CC
NA18502	192	CA	0.75	*CA*	*0*	CA
NA19153	193	CC	0.9035	*CC*	*0*	CC
NA18857	194	CC	1	cc	0.00575132	CC
NA19223	195	NN	0.7441	*AA*	*0*	AA
NA19201	196	CA	0.9964	*CA*	*0*	CA
NA18504	197	CC	0.8587	*CC*	*0*	CC
NA18870	198	CA	0.9979	ca	0.002203507	CA
NA18863	199	NN	0.6331	*CC*	*0*	CC
NA19145	200	CA	0.9638	*CA*	*0*	CA
NA19137	201	CC	1	*CC*	*0*	CC
NA19238	202	CC	1	cc	0.050066034	nn
NA18500	203	CA	0.9961	*CA*	*0*	CA
NA19144	204	NN	0.7282	*CA*	*0*	CA
NA19203	205	CA	1	ca	0.389396074	CA
NA19200	206	CA	0.9991	ca	0.003821399	CA
NA18855	207	CC	0.7668	*CC*	*0*	CC
NA18505	208	CA	0.9997	ca	0.06493811	CA
NA19202	209	CA	0.9959	ca	0.002568424	CA
NA18501	210	CC	0.7501	*CC*	*0*	CC
NA18861	211	CC	0.9744	*CC*	*0*	CC
NA19193	212	CC	1	*CC*	*0*	CC
NA19143	213	CC	0.7819	*CC*	*0*	CC
NA18517	214	CA	1	ca	0.226987551	CA
NA18862	215	NN	0.7365	*CC*	*0*	CC
NA18856	216	CC	1	*CC*	*0*	nn
NA19239	217	CA	1	ca	0.325904684	CA
NA19240	218	CA	1	ca	0.090305362	CA
NA18856	219	CC	1	cc	0.003114196	CC
NA18503	220	CC	1	cc	0.508737357	CC

	MACGT discrepancy					
	LDA discrepancy					
NA	LDA output were not g					
XY	Manual calls are given					
		**2938675**				
**Coriell**	**Sample**	**LDA Call**	**LDA quality score**	**MACGT Call**	**MACGT quality score**	**HapMap Call**
NA18871	221	CA	0.9996	ca	0.094094657	CA
NA19221	222	CA	0.9999	ca	0.10012053	CA
NA19209	223	AA	0.9985	***AA***	***0***	AA
NA19152	224	CA	0.9997	ca	0.006125051	CA
NA18515	225	CA	0.9993	ca	0.033333973	CA
NA19238	226	CC	1	cc	0.197526818	CC
NA19154	227	CA	0.9999	ca	0.270214937	CA
NA19210	228	CA	0.9957	***CA***	***0***	CA
NA19211	229	AA	0.7731	aa	0.001271664	AA
NA18862	230	CC	1	cc	0.425167641	nn
NA18872	231	CA	0.9999	ca	0.041482028	CA
NA19139	232	CA	0.9999	ca	0.24041407	CA
NA19222	233	CC	1	cc	0.008061955	CC
NA19194	234	CC	0.9849	cc	0.519160462	CC
NA19138	235	CA	0.9994	ca	0.001043667	CA
NA19204	236	CC	1	cc	0.512166438	CC
NA18516	237	CA	0.9994	ca	0.07921643	CA
NA19205	238	CA	0.9999	ca	0.074432101	CA
NA19192	239	CA	0.9995	ca	0.065990284	CA
NA18912	240	CC	0.9852	cc	0.165292827	nn
NA18508	241	CC	0.971	cc	0.139470072	CC
NA19142	242	AA	0.7678	aa	0.003782202	AA
NA18852	243	CC	1	cc	0.435609269	CC
NA18507	244	CC	1	cc	0.024773732	CC
blank	245	NN	0.5529	***NN***	***0***	nn
NA19101	246	CA	0.9999	ca	0.175971815	CA
NA19172	247	CA	0.9999	ca	0.07272216	CA
NA19160	248	CA	0.9996	ca	0.07764003	CA
NA19129	249	CA	0.9998	ca	0.044896245	CA
NA18913	250	CA	0.9999	ca	0.040689764	CA
NA19120	251	CC	0.9249	***CC***	***0***	CC
NA19159	252	CC	0.9535	cc	0.032826371	CC
NA18523	253	CC	0.9937	cc	0.126439519	CC
NA19102	254	NN	0.7068	***AA***	***0***	AA
NA19092	255	CC	0.9999	cc	0.081620908	CC
NA18521	256	CC	0.9998	cc	0.126217187	CC
NA18912	257	CC	1	cc	0.001951739	CC
NA19206	258	AA	0.9989	aa	0.01990425	AA
NA19128	259	CA	0.9997	ca	0.231737969	CA
NA18859	260	CA	0.9997	ca	0.115437809	nn
NA19094	261	CC	1	cc	0.095359256	CC
NA19171	262	CA	0.9994	ca	0.237418444	CA
NA18860	263	CA	1	ca	0.059968469	CA
NA18858	264	AA	1	aa	0.027392895	AA

	MACGT discrepancy					
	LDA discrepancy					
NA	LDA output were not g					
XY	Manual calls are given					
		2938675				
**Coriell**	**Sample**	**LDA Call**	**LDA quality score**	**MACGT Call**	**MACGT quality score**	**HapMap Call**
NA19099	265	CC	1	***CC***	***0***	CC
NA18506	266	CC	1	***CC***	***6.23E-04***	CC
NA19131	267	CA	0.9999	ca	0.174967385	CA
NA19116	268	CC	1	***CC***	***0***	CC
NA19161	269	CC	1	***CC***	***0***	CC
NA19140	270	CA	0.9999	ca	0.026734957	CA
NA19207	271	CC	1	cc	0.119835138	CC
NA19141	272	CA	0.9999	ca	0.064366051	CA
NA18854	273	CC	1	***CC***	***0***	CC
NA19098	274	CA	1	ca	0.48070243	CA
NA18522	275	CA	0.9998	ca	0.163452655	CA
NA18914	276	CC	0.9898	***CC***	***0***	CC
NA18853	277	CA	1	ca	0.214067003	CA
NA18859	278	CA	0.9999	ca	0.119194906	CA
NA19100	279	CA	1	ca	0.295421345	CA
NA19093	280	CA	0.9999	ca	0.117498933	CA
NA19132	281	AA	0.9971	***AA***	***0***	AA
NA19130	282	AA	0.9981	aa	0.017753308	AA
NA19208	283	CA	0.9998	ca	0.009126655	CA
NA19127	284	CA	0.9998	ca	0.556353881	CA
NA19173	285	CA	0.9997	ca	0.12197571	CA
NA19119	286	CA	1	ca	0.003115355	CA
NA19103	287	CA	1	ca	0.364126553	CA
	Discrepancies	0		0		
	NNs	5		0		
	Automated Calls	278		228		
	Manual Calls	0		50		
	Concordant Calls	254		259		259

	MACGT discrepancy					
	LDA discrepancy					
NA	LDA output were not g					
XY	Manual calls are given					
		318841				
**Coriell**	**Sample**	**LDA Call**	**LDA quality score**	**MACGT Call**	**MACGT quality score**	**HapMap Call**
NA12753	1		NA	GG	0.551187273	
NA12875	2	CG	0.9678	cg	0.191170768	CG
NA12044	3	CG	0.8091	cg	0.489895809	CG
NA06993	4	GG	0.8194	gg	0.180840609	GG
NA12716	5		NA	CG	0.243628343	
NA12760	6	NN	0.6184	***GG***	***0***	GG
NA07348	7		NA	GG	0.273395083	
NA12707	8	CG	0.9663	cg	0.160042006	CG
NA12156	9		NA	GG	0.250309089	
NA11992	10	NN	0.7348	cc	0.014373331	CC
NA12239	11	NN	0.5699	gg	0.098567529	GG
NA12878	12	NN	0.649	gg	0.202594337	GG
NA11993	13	GG	0.9808	***GG***	***0***	GG
NA12750	14	GG	0.8707	gg	0.108415743	GG
NA12146	15	GG	0.9046	gg	0.004058753	GG
NA11839	16	GG	0.8294	gg	0.013794729	GG
NA12156.dup	17	GG	0.8853	gg	0.006358953	nn
NA11829	18		NA	CG	0.214750265	
NA12154	19	GG	0.8013	gg	0.04475106	GG
NA10856	20	CG	0.9628	cg	0.259445018	CG
NA06985	21	CG	0.963	cg	0.197714749	CG
NA11831	22	GG	0.8983	gg	0.022899044	GG
NA12003.dup	23	CG	0.9866	cg	0.007957311	nn
NA10859	24	CG	0.93	cg	0.338869333	CG
NA07056	25	CG	0.923	cg	0.145961912	CG
NA07022	26	GG	0.8768	gg	0.296793553	GG
NA12762	27		NA	GG	0.265890008	
NA12874	28	NN	0.6444	gg	0.083710786	GG
NA12003	29	CG	0.9389	cg	0.168415356	CG
NA10835	30	GG	0.8761	gg	0.057819121	GG
NA11882	31	CG	0.9573	cg	0.403902062	CG
NA07034	32	CG	0.9642	cg	0.206520892	CG
NA12056	33	CG	0.9257	cg	0.320369583	CG
NA12145	34	CG	0.8459	cg	0.208728188	CG
NA07019	35	CG	0.9451	cg	0.310659404	CG
NA06991	36		NA	CG	0.307313914	
NA12761	37	GG	0.9792	gg	0.48061858	GG
NA06994	38	GG	0.9835	gg	0.267525224	GG
NA12864	39	NN	0.7132	cg	0.217153448	CG
NA07055	40	CG	0.9506	cg	0.322802204	CG
NA10863	41	GG	0.8376	gg	0.144423079	GG
NA12763	42	CG	0.9244	cg	0.293455572	CG
NA10831	43	CG	0.7644	cg	0.165851053	CG
NA11840	44	CC	0.9793	cc	0.132310949	CC

	MACGT discrepancy					
	LDA discrepancy					
NA	LDA output were not g					
XY	Manual calls are given					
		**318841**				
**Coriell**	**Sample**	**LDA Call**	**LDA quality score**	**MACGT Call**	**MACGT quality score**	**HapMap Call**
NA10830	45	GG	0.9642	gg	0.020949853	GG
NA11993.dup	46	GG	0.8196	cg	0.001167341	nn
NA12751	47	CG	0.9255	cg	0.176126442	CG
NA12814	48	GG	0.7726	gg	0.01186393	GG
NA10857	49	GG	0.8894	gg	0.353769816	GG
NA07357	50	GG	0.8666	gg	0.506454973	GG
NA07000	51	CG	0.962	cg	0.10980263	CG
NA12802	52	GG	0.9811	gg	0.367519151	GG
NA10855	53	GG	0.9799	gg	0.053759395	GG
NA11995	54		NA	CC	0.448105436	
NA10839	55	CG	0.9664	cg	0.425304299	CG
NA10847	56	GG	0.9641	gg	0.039746602	GG
NA12006	57	GG	0.8072	gg	0.209081301	GG
NA12144	58	CG	0.9489	cg	0.204898059	CG
NA11832	59	GG	0.8196	gg	0.202876282	GG
NA12813	60	GG	0.9677	gg	0.145934398	GG
blank	61	NN	0.5076	***NN***	***0***	nn
NA12891	62	CG	0.8079	cg	0.201456048	CG
NA12717	63		NA	GG	0.411145467	
NA10861	64	CC	0.9145	cc	0.158239798	CC
NA12004	65	GG	0.9445	gg	0.071878782	GG
NA07048	66	GG	0.9587	gg	0.328399025	GG
NA12801	67	CG	0.8718	cg	0.192918909	CG
NA11830	68	NN	0.7375	cg	0.174435169	CG
NA12057	69	CG	0.937	cg	0.217088564	CG
NA11994	70		NA	CG	0.409330629	
NA12740	71	CG	0.9043	cg	0.105589352	CG
NA12005	72	CG	0.7901	cg	0.08474621	CG
NA07345	73	CG	0.9375	cg	0.217206705	CG
NA10854	74	CG	0.9659	cg	0.124238255	CG
NA12872	75	GG	0.8238	***GG***	***0***	GG
NA12865	76	CG	0.9478	cg	0.460780313	CG
NA10860	77		NA	CG	0.374961125	
NA12873	78	CC	0.9925	cc	0.362431143	CC
NA12752	79	GG	0.8679	gg	0.306487833	GG
NA10851	80	CC	0.9305	cc	0.332795578	CC
NA07029	81	CG	0.9441	cg	0.35027554	CG
NA12248	82	GG	0.854	gg	0.003593748	GG
NA12155	83	CG	0.8278	cg	0.181661026	CG
NA10838	84	NN	0.7405	gg	0.24732217	GG
NA12812	85	CC	0.9896	cc	0.111282843	CC
NA12248.dup	86	GG	0.8075	gg	0.326655916	nn
NA12043	87	NN	0.6967	gg	0.16503122	GG
NA12234	88	NN	0.7313	gg	0.149694365	GG

	MACGT discrepancy					
	LDA discrepancy					
NA	LDA output were not g					
XY	Manual calls are given					
		318841				
**Coriell**	**Sample**	**LDA Call**	**LDA quality score**	**MACGT Call**	**MACGT quality score**	**HapMap Call**
NA12892	89	GG	0.8982	gg	0.119652128	GG
NA10846	90	CC	0.9922	cc	0.101043858	CC
NA06993.dup	91	GG	0.9643	gg	0.254705905	nn
NA12249	92	GG	0.9079	gg	0.239022308	GG
NA11881	93	GG	0.9795	gg	0.278290368	GG
NA12815	94	CG	0.8052	cg	0.143028147	CG
NA12264	95	GG	0.981	gg	0.500823236	GG
NA18526	96	CG	0.958	cg	0.289819721	CG
NA18562	97	CG	0.9363	cg	0.414996724	CG
NA18545	98	CG	0.9553	cg	0.222120982	CG
NA18609	99	GG	0.9688	gg	0.375809208	GG
NA18566	100	GG	0.8323	gg	0.230353381	GG
NA18621	101	CG	0.9469	cg	0.178149588	CG
NA18577	102	GG	0.9851	gg	0.146843462	GG
NA18635	103	CG	0.9452	cg	0.197414351	CG
NA18524	104	GG	0.9707	gg	0.351424185	GG
NA18537	105	CG	0.7911	cg	0.069061288	CG
NA18572	106	GG	0.8229	gg	0.507606916	GG
NA18552	107	CG	0.9454	cg	0.033058775	CG
NA18563	108	GG	0.9593	***GG***	***0***	GG
NA18594	109	CG	0.9258	cg	0.12040872	nn
NA18624	110	GG	0.8326	gg	0.425044167	GG
NA18592	111	CG	0.8098	cg	0.512560484	CG
NA18529	112	GG	0.9697	gg	0.552643915	GG
NA18603	113	CC	0.9097	cc	0.375239253	nn
NA18547	114	CC	0.8894	cc	0.485567489	CC
NA18611	115	GG	0.7908	gg	0.036820495	GG
NA18570	116	CG	0.9733	cg	0.217203544	CG
NA18622	117	CC	0.9714	cc	0.404687523	CC
NA18579	118	CG	0.9754	cg	0.19455219	CG
NA18636	119	GG	0.9796	gg	0.401881841	GG
NA18558	120	GG	0.8285	gg	0.302412329	GG
NA18540	121	CC	0.9797	cc	0.229504743	CC
NA18609	122	GG	0.8092	gg	0.423836256	nn
NA18555	123	CC	0.899	cc	0.028708973	CC
NA18612	124	CG	0.9571	cg	0.212455138	CG
NA18573	125	CG	0.9714	cg	0.255651259	CG
NA18632	126	CG	0.9695	cg	0.233753852	CG
NA18593	127	CG	0.9586	cg	0.489695661	CG
NA18532	128	GG	0.97	gg	0.228118189	GG
NA18605	129	GG	0.9768	gg	0.513198931	GG
NA18550	130	GG	0.9827	gg	0.46119491	GG
NA18603	131	CC	0.9997	cc	0.323140404	CC
NA18571	132	GG	0.8847	gg	0.366330295	GG

	MACGT discrepancy					
	LDA discrepancy					
NA	LDA output were not g					
XY	Manual calls are given					
		**318841**				
**Coriell**	**Sample**	**LDA Call**	**LDA quality score**	**MACGT Call**	**MACGT quality score**	**HapMap Call**
NA18623	133	CG	0.8196	cg	0.359494831	CG
NA18582	134	GG	0.962	gg	0.022824345	GG
NA18637	135	GG	0.8752	gg	0.004620795	GG
NA18561	136	CG	0.9581	cg	0.292047225	CG
NA18542	137	GG	0.8307	gg	0.325713629	GG
NA18608	138	GG	0.9701	gg	0.422279687	GG
NA18564	139	GG	0.9784	***GG***	***0***	GG
NA18620	140	CC	0.9996	cc	0.351938624	CC
NA18576	141	GG	0.9778	gg	0.039178045	GG
NA18633	142	CG	0.9651	cg	0.357598336	CG
NA18594	143	CG	0.964	cg	0.381985965	CG
NA18942	144	CG	0.9649	cg	0.295271901	CG
NA18945	145	CG	0.9622	cg	0.349583918	CG
NA18964	146	CG	0.9445	cg	0.246306168	CG
NA18961	147	GG	0.7595	***GG***	***0***	GG
NA18967	148	CG	0.9463	cg	0.426574116	CG
NA18981	149	CC	0.9995	cc	0.317381436	CC
NA18994	150	CG	0.7931	cg	0.251535096	CG
NA18998	151	CG	0.9616	cg	0.07760164	CG
NA18940	152	CG	0.9661	cg	0.171512547	CG
NA18949	153	CC	0.8299	cc	0.427024143	CC
NA18953	154	CG	0.9349	cg	0.010258311	CG
NA18972	155	CC	0.8763	cc	0.347809666	CC
NA18976	156	CG	0.9623	cg	0.194857263	CG
NA18971	157	CC	0.8335	cc	0.421957047	CC
blank	158	NN	0.4795	***NN***	***0***	nn
NA19000	159	CG	0.8324	cg	0.037436049	CG
NA18951	160	CG	0.8329	cg	0.082340676	nn
NA18948	161	GG	0.9484	gg	0.079332006	GG
NA18968	162	CC	0.9745	cc	0.072133673	CC
NA18965	163	NN	0.6016	***CC***	***0***	CC
NA18978	164	CC	0.9712	cc	0.015691309	CC
NA18974	165	NN	0.5321	cg	0.001545743	CG
NA18992	166	CG	0.7889	***CG***	***5.86E-04***	CG
NA19005	167	GG	0.8559	gg	0.240318291	GG
NA18943	168	CG	0.9598	cg	0.048046829	CG
NA18951	169	CG	0.9682	cg	0.336508642	CG
NA18959	170	CG	0.9603	cg	0.305551786	CG
NA18973	171	CG	0.9682	cg	0.244108308	CG
NA18970	172	GG	0.9777	gg	0.017602547	GG
NA18987	173	CG	0.9741	cg	0.103147983	CG
NA18995	174	CG	0.9463	cg	0.135865644	CG
NA18999	175	NN	0.7475	cg	0.217178354	CG
NA18947	176	CG	0.8062	cg	0.030373794	CG

	MACGT discrepancy					
	LDA discrepancy					
NA	LDA output were not g					
XY	Manual calls are given					
		**318841**				
**Coriell**	**Sample**	**LDA Call**	**LDA quality score**	**MACGT Call**	**MACGT quality score**	**HapMap Call**
NA18952	177	GG	0.8562	gg	0.17990989	GG
NA18969	178	GG	0.83	gg	0.379204922	GG
NA18966	179	GG	0.8211	gg	0.257394605	GG
NA18980	180		NA	CC	0.375719446	
NA18990	181	CG	0.9488	cg	0.107092664	CG
NA18997	182	GG	0.9652	gg	0.221735662	GG
NA19007	183	CC	0.9656	cc	0.359007844	CC
NA18944	184	CC	0.7904	cc	0.259700255	CC
NA18956	185	GG	0.8655	gg	0.206983725	GG
NA18960	186	CG	0.9557	cg	0.025166066	CG
NA18975	187	CG	0.811	cg	0.324400982	CG
NA18995	188	CG	0.7926	cg	0.383377452	nn
NA18991	189	CG	0.955	cg	0.237775389	CG
NA18996	190	GG	0.8338	gg	0.004845865	nn
NA19003	191	GG	0.8565	gg	0.48038572	GG
NA18502	192	CG	0.8269	cg	0.065731747	CG
NA19153	193	CC	0.9886	cc	0.001764417	CC
NA18857	194	GG	0.7957	gg	0.319915331	GG
NA19223	195	CC	0.7636	cc	0.020682076	CC
NA19201	196	CC	0.8505	cc	0.355096293	CC
NA18504	197	CC	0.9259	cc	0.090707992	CC
NA18870	198	GG	0.7818	gg	0.438751199	GG
NA18863	199	NN	0.6599	gg	0.166746442	GG
NA19145	200	CG	0.9317	cg	0.098535923	CG
NA19137	201	CG	0.9306	cg	0.106899279	CG
NA19238	202	CG	0.9627	cg	0.033507275	nn
NA18500	203	CG	0.9073	cg	0.109573645	CG
NA19144	204	NN	0.6183	gg	0.12266711	GG
NA19203	205	CG	0.7838	cg	0.168397387	CG
NA19200	206	GG	0.7913	***GG***	***0***	GG
NA18855	207	CG	0.978	cg	0.132704806	CG
NA18505	208	CG	0.9513	cg	0.123156471	CG
NA19202	209	CG	0.9315	cg	0.032251767	CG
NA18501	210	CG	0.9355	cg	0.087107964	CG
NA18861	211	NN	0.6972	cg	0.019591711	CG
NA19193	212	CG	0.9093	cg	0.0634612	CG
NA19143	213	NN	0.7177	cc	0.122725467	CC
NA18517	214	NN	0.682	cg	0.144653734	CG
NA18862	215	CG	0.927	cg	0.11738315	CG
NA18856	216	NN	0.5057	gg	0.005309538	nn
NA19239	217	NN	0.7156	cg	0.007906798	CG
NA19240	218	CG	0.949	cg	0.262248636	CG
NA18856	219	NN	0.6364	***NN***	***0***	GG
NA18503	220	NN	0.5817	***CC***	***0***	CC

	MACGT discrepancy					
	LDA discrepancy					
NA	LDA output were not g					
XY	Manual calls are given					
		318841				
**Coriell**	**Sample**	**LDA Call**	**LDA quality score**	**MACGT Call**	**MACGT quality score**	**HapMap Call**
NA18871	221	CG	0.9768	cg	0.146393751	CG
NA19221	222	CG	0.9011	cg	0.012768373	CG
NA19209	223	NN	0.5433	*NN*	*0*	CG
NA19152	224	CC	0.7706	*CC*	*0*	CC
NA18515	225	CG	0.889	cg	0.006627496	CG
NA19238	226	CG	0.9702	cg	0.0099552	CG
NA19154	227	CC	0.8041	cc	0.007427608	CC
NA19210	228	NN	0.4398	cc	0.01138207	CC
NA19211	229	CC	0.817	cc	0.053311526	CC
NA18862	230	CG	0.9393	cg	0.29707343	nn
NA18872	231	NN	0.7356	gg	0.06237015	GG
NA19139	232	CG	0.8937	cg	0.007310078	CG
NA19222	233	NN	0.6792	*NN*	*7.20E-04*	CG
NA19194	234	CG	0.7662	*NN*	*5.83E-04*	CG
NA19138	235	NN	0.5354	*NN*	*0*	CG
NA19204	236	CG	0.9414	cg	0.132011842	CG
NA18516	237	NN	0.7199	cc	0.003018797	CC
NA19205	238	CG	0.9737	cg	0.049985274	CG
NA19192	239	GG	0.7599	cg	0.005831019	GG
NA18912	240	CC	0.7774	cc	0.11059707	nn
NA18508	241	CC	0.873	cc	0.003600328	CC
NA19142	242	NN	0.6354	cc	0.041772986	CC
NA18852	243	CG	0.925	cg	0.131551533	CG
NA18507	244	CG	0.8839	*CG*	*5.55E-04*	CG
blank	245	NN	0.6729	*NN*	*0*	nn
NA19101	246		NA	CC	0.440530475	
NA19172	247	CC	0.8033	*CC*	*0*	CC
NA19160	248	NN	0.5681	gg	0.011052681	GG
NA19129	249	CG	0.8099	cg	0.088136235	CG
NA18913	250	NN	0.544	*GG*	*0*	GG
NA19120	251	CG	0.8081	*NN*	*0*	CG
NA19159	252	NN	0.5498	*NN*	*0*	CG
NA18523	253	CG	0.9342	cg	0.029732759	CG
NA19102	254	NN	0.7176	cg	0.001503764	CG
NA19092	255	CC	0.8842	cc	0.064639764	CC
NA18521	256	GG	0.8267	gg	0.001449162	GG
NA18912	257	CC	0.8917	cc	0.002464405	CC
NA19206	258	GG	0.8546	gg	0.008048223	GG
NA19128	259	CG	0.9561	cg	0.117902766	CG
NA18859	260	CG	0.9354	cg	0.072686391	nn
NA19094	261		NA	CC	0.190089819	
NA19171	262	GG	0.9434	gg	0.00765822	GG
NA18860	263	CG	0.9437	cg	0.06396362	CG
NA18858	264	GG	0.9161	gg	0.003584436	GG

	MACGT discrepancy					
	LDA discrepancy					
NA	LDA output were not g					
XY	Manual calls are given					
		**318841**				
**Coriell**	**Sample**	**LDA Call**	**LDA quality score**	**MACGT Call**	**MACGT quality score**	**HapMap Call**
NA19099	265	CG	0.8004	cg	0.128543186	CG
NA18506	266	CC	0.9941	cc	0.003769015	CC
NA19131	267	NN	0.707	cg	0.005923667	CG
NA19116	268	NN	0.4398	***NN***	***0***	CG
NA19161	269	NN	0.6698	cg	0.004464756	CG
NA19140	270	NN	0.6072	cg	0.092257408	CG
NA19207	271	CC	0.9768	cc	0.232429015	CC
NA19141	272	CC	0.7715	cc	0.102162342	CC
NA18854	273	NN	0.7203	cg	0.101207342	CG
NA19098	274	CG	0.7757	cg	0.313581542	CG
NA18522	275	CG	0.7947	cg	0.018720958	CG
NA18914	276	NN	0.6998	***CG***	***0***	CG
NA18853	277	CG	0.8812	cg	0.014523562	CG
NA18859	278	NN	0.7336	cg	0.007974231	CG
NA19100	279	NN	0.6372	***NN***	***0***	CG
NA19093	280	CC	0.8172	cc	0.061043711	CC
NA19132	281	CG	0.9104	cg	0.089351706	CG
NA19130	282	CG	0.8529	***CG***	***0***	CG
NA19208	283	CG	0.7716	cg	0.002971828	CG
NA19127	284	CG	0.7808	cg	0.144916329	CG
NA19173	285	CG	0.9341	cg	0.132430384	CG
NA19119	286	GG	0.9543	gg	0.077566986	GG
NA19103	287	CG	0.7539	cg	0.003714703	CG
	Discrepancies	0		1		
	NNs	42		10		
	Automated Calls	273		245		
	Manual Calls	0		28		
	Concordant Calls	214		244		254

	MACGT discrepancy					
	LDA discrepancy					
NA	LDA output were not g					
XY	Manual calls are given					
		365063				
**Coriell**	**Sample**	**LDA Call**	**LDA quality score**	**MACGT Call**	**MACGT quality score**	**HapMap Call**
NA12753	1		NA	AG	0.511670895	
NA12875	2	AG	1	ag	0.086877898	AG
NA12044	3	AA	1	aa	0.377209692	AA
NA06993	4	AA	1	aa	0.310908838	AA
NA12716	5	AA	1	aa	0.312987593	AA
NA12760	6	GG	1	gg	0.504333169	GG
NA07348	7	AA	1	aa	0.566990306	AA
NA12707	8	AA	1	aa	0.276816098	AA
NA12156	9		NA	AA	0.371954408	
NA11992	10	AG	1	ag	0.010763122	AG
NA12239	11	GG	1	gg	0.153199783	GG
NA12878	12	AA	1	aa	0.293495504	AA
NA11993	13	AG	1	ag	0.026106992	AG
NA12750	14	AG	1	ag	0.002477782	AG
NA12146	15	GG	1	gg	0.013685729	GG
NA11839	16	AG	1	***AG***	***0***	AG
NA12156.dup	17	AA	1	aa	0.267870975	nn
NA11829	18		NA	GG	0.446102736	
NA12154	19	AG	1	ag	0.02856704	AG
NA10856	20	AG	1	ag	0.020152448	AG
NA06985	21	AG	1	ag	0.011084128	AG
NA11831	22	AA	0.9992	aa	0.007433748	AA
NA12003.dup	23	AG	1	***AG***	***0***	nn
NA10859	24	AA	0.9928	aa	0.09872414	AA
NA07056	25	AG	1	ag	0.09986489	AG
NA07022	26	AG	1	***AG***	***0***	AG
NA12762	27		NA	AA	0.410760967	
NA12874	28	AA	0.7666	aa	0.043000072	AA
NA12003	29	AG	1	ag	0.105169585	AG
NA10835	30	AA	1	aa	0.293310815	AA
NA11882	31	AA	1	aa	0.254580505	AA
NA07034	32	GG	1	gg	0.153563199	GG
NA12056	33	GG	1	gg	0.284600025	GG
NA12145	34	AA	1	aa	0.148454638	AA
NA07019	35	AG	1	ag	0.078831475	AG
NA06991	36		NA	AA	0.456449989	
NA12761	37	AA	0.9855	aa	0.084462833	AA
NA06994	38	AA	1	aa	0.245074884	AA
NA12864	39	AA	1	aa	0.431146893	AA
NA07055	40	AA	0.9999	aa	0.387326565	AA
NA10863	41	AG	1	ag	0.047126824	AG
NA12763	42	GG	1	gg	0.166800265	GG
NA10831	43	AA	1	aa	0.139542546	AA
NA11840	44	AA	1	aa	0.45614918	AA

	MACGT discrepancy					
	LDA discrepancy					
NA	LDA output were not g					
XY	Manual calls are given					
		**365063**				
**Coriell**	**Sample**	**LDA Call**	**LDA quality score**	**MACGT Call**	**MACGT quality score**	**HapMap Call**
NA10830	45	AA	1	aa	0.456159027	AA
NA11993.dup	46	AG	1	ag	0.477267038	nn
NA12751	47	AA	1	aa	0.332781116	AA
NA12814	48	AA	1	aa	0.31629539	AA
NA10857	49	AG	1	*AG*	*0*	AG
NA07357	50	AA	1	aa	0.421823888	AA
NA07000	51	AA	1	aa	0.545090278	AA
NA12802	52	AA	1	aa	0.336447893	AA
NA10855	53	AA	1	aa	0.054915399	AA
NA11995	54		NA	AA	0.077955665	
NA10839	55	AA	1	aa	0.178720112	AA
NA10847	56	GG	1	gg	0.085063708	GG
NA12006	57	AG	1	ag	0.004074926	AG
NA12144	58	AA	1	aa	0.367515533	AA
NA11832	59	AA	1	aa	0.279517318	AA
NA12813	60	AG	1	ag	0.162265278	AG
blank	61	AA	1	aa	0.248104473	nn
NA12891	62	AG	1	ag	0.138008757	AG
NA12717	63		NA	AA	0.276047423	
NA10861	64	AA	1	aa	0.210247155	AA
NA12004	65	AG	1	ag	0.341614175	AG
NA07048	66	AG	1	ag	0.434334253	AG
NA12801	67	AA	1	aa	0.270816162	AA
NA11830	68	AA	1	aa	0.198108478	AA
NA12057	69	AG	1	ag	0.140288292	AG
NA11994	70		NA	AA	0.466599111	
NA12740	71	AG	1	ag	0.058319574	AG
NA12005	72	AG	1	ag	0.04747155	AG
NA07345	73	AA	0.9689	aa	0.050850351	AA
NA10854	74	AA	1	aa	0.219996108	AA
NA12872	75	AA	1	aa	0.491552991	AA
NA12865	76	AA	1	aa	0.502651798	AA
NA10860	77		NA	GG	0.226862411	
NA12873	78	AA	1	aa	0.138663802	AA
NA12752	79	AG	0.9999	ag	0.008350176	AG
NA10851	80	GG	1	gg	0.57916648	GG
NA07029	81	AA	1	aa	0.236340604	AA
NA12248	82	AA	1	aa	0.115684114	AA
NA12155	83	AG	1	*AG*	*0*	AG
NA10838	84	AA	1	aa	0.445832818	AA
NA12812	85	AG	1	ag	0.001730141	AG
NA12248.dup	86	AA	1	aa	0.409932797	nn
NA12043	87	AG	1	ag	0.119470741	AG
NA12234	88	AG	1	ag	0.054940256	AG

	MACGT discrepancy					
	LDA discrepancy					
NA	LDA output were not g					
XY	Manual calls are given					
		**365063**				
**Coriell**	**Sample**	**LDA Call**	**LDA quality score**	**MACGT Call**	**MACGT quality score**	**HapMap Call**
NA12892	89	AA	1	aa	0.333340725	AA
NA10846	90	AA	1	aa	0.375481355	AA
NA06993.dup	91	AA	0.9642	aa	0.003495595	nn
NA12249	92	AA	0.8541	aa	0.030071407	AA
NA11881	93	AA	0.9985	aa	0.100205756	AA
NA12815	94	AA	0.9999	aa	0.091483531	AA
NA12264	95	AG	1	ag	0.328564677	AG
NA18526	96	AG	0.9998	ag	0.147451534	AG
NA18562	97	AA	1	aa	0.501002657	AA
NA18545	98	AA	1	aa	0.156101464	AA
NA18609	99	AA	1	aa	0.163209996	AA
NA18566	100	AG	1	***AG***	***0***	AG
NA18621	101	AG	0.9999	ag	0.003180777	AG
NA18577	102	AA	0.9999	aa	0.083498619	AA
NA18635	103	AG	1	***AG***	***0***	AG
NA18524	104	AA	1	aa	0.485796003	AA
NA18537	105	AA	0.9995	aa	0.228761654	AA
NA18572	106	AG	1	ag	0.211006836	AG
NA18552	107	AG	1	ag	0.188375641	AG
NA18563	108	AA	1	aa	0.215386851	AA
NA18594	109	AA	0.9968	aa	0.25952963	nn
NA18624	110	AA	1	aa	0.39277193	AA
NA18592	111	AG	1	ag	0.236503412	AG
NA18529	112	GG	1	gg	0.464301191	GG
NA18603	113	AA	1	aa	0.360970386	nn
NA18547	114	AG	1	ag	0.002646504	AG
NA18611	115	AG	1	ag	0.062366846	AG
NA18570	116	AA	1	aa	0.340311707	AA
NA18622	117	AG	0.9999	ag	0.10982074	AG
NA18579	118	AG	1	ag	0.314402583	AG
NA18636	119	AG	1	ag	0.024148917	AG
NA18558	120	AG	0.9999	ag	0.038813202	AG
NA18540	121	AA	1	aa	0.081396926	AA
NA18609	122	AA	1	aa	0.275026036	nn
NA18555	123	AG	0.9998	ag	0.130511915	AG
NA18612	124	AA	1	aa	0.321406966	AA
NA18573	125	AA	1	aa	0.120914654	AA
NA18632	126	AG	0.9998	ag	0.187101616	AG
NA18593	127	AA	1	aa	0.460119789	AA
NA18532	128	AG	1	ag	0.122392064	AG
NA18605	129	AA	1	aa	0.380528962	AA
NA18550	130	AG	1	ag	0.225058008	AG
NA18603	131	AA	1	aa	0.128113236	AA
NA18571	132	AA	1	aa	0.478486885	AA

	MACGT discrepancy					
	LDA discrepancy					
NA	LDA output were not g					
XY	Manual calls are given					
		365063				
**Coriell**	**Sample**	**LDA Call**	**LDA quality score**	**MACGT Call**	**MACGT quality score**	**HapMap Call**
NA18623	133	AA	1	aa	0.240060837	AA
NA18582	134	AA	0.9989	aa	0.07253979	AA
NA18637	135	AG	1	ag	0.009100047	AG
NA18561	136	AA	1	aa	0.358670169	AA
NA18542	137	AA	1	aa	0.295284153	AA
NA18608	138	AA	0.9999	aa	0.204016807	AA
NA18564	139	AG	0.9991	ag	0.004017092	AG
NA18620	140	AA	1	aa	0.530155802	AA
NA18576	141	AA	1	aa	0.564065552	AA
NA18633	142	AG	0.9999	ag	0.013197071	AG
NA18594	143	AA	1	aa	0.312040203	AA
NA18942	144	AG	1	ag	0.115492316	AG
NA18945	145	AA	1	aa	0.432215042	AA
NA18964	146	AG	1	ag	0.257745771	AG
NA18961	147	AG	1	ag	0.275825004	AG
NA18967	148	AG	1	ag	0.248686987	AG
NA18981	149	AA	1	aa	0.407578739	AA
NA18994	150	AA	1	aa	0.487426875	AA
NA18998	151	AA	1	aa	0.34569153	AA
NA18940	152	AG	1	ag	0.197162589	AG
NA18949	153	AG	1	ag	0.090049941	AG
NA18953	154	AA	1	aa	0.317956072	AA
NA18972	155	AA	1	aa	0.129167046	AA
NA18976	156	AA	1	aa	0.288117232	AA
NA18971	157	AA	1	aa	0.36155054	AA
blank	158	NN	0.5	***NN***	***0***	nn
NA19000	159	AG	1	ag	0.09679759	AG
NA18951	160	AG	1	ag	0.07162728	nn
NA18948	161	AA	0.7503	aa	0.00113776	AA
NA18968	162	AG	0.9117	ag	0.054632684	AG
NA18965	163	AG	0.9765	ag	0.145375308	AG
NA18978	164	AG	0.9979	ag	0.077519027	AG
NA18974	165	AA	0.7808	aa	0.007918322	AA
NA18992	166	AA	0.9137	aa	0.027654599	AA
NA19005	167	AA	1	aa	0.319599912	AA
NA18943	168	AA	1	aa	0.243775356	AA
NA18951	169	AG	1	***AG***	***0***	AG
NA18959	170	AA	1	aa	0.234190271	AA
NA18973	171	AG	1	ag	0.303795442	AG
NA18970	172	AA	1	aa	0.352083941	AA
NA18987	173	AA	1	aa	0.280274194	AA
NA18995	174	AA	1	aa	0.491178176	AA
NA18999	175	AA	1	aa	0.405741048	AA
NA18947	176	AA	1	aa	0.132490666	AA

	MACGT discrepancy					
	LDA discrepancy					
NA	LDA output were not g					
XY	Manual calls are given					
		365063				
**Coriell**	**Sample**	**LDA Call**	**LDA quality score**	**MACGT Call**	**MACGT quality score**	**HapMap Call**
NA18952	177	AG	1	ag	0.065367267	AG
NA18969	178	AA	1	aa	0.350552127	AA
NA18966	179	AA	1	aa	0.44367252	AA
NA18980	180	AA	1	aa	0.263425892	AA
NA18990	181	AG	1	ag	0.224889084	AG
NA18997	182	AA	1	aa	0.19433232	AA
NA19007	183	AG	0.9999	ag	0.039139061	AG
NA18944	184	AG	1	ag	0.005225135	AG
NA18956	185	AG	1	***AG***	***0***	AG
NA18960	186	AA	1	aa	0.270580486	AA
NA18975	187	AA	1	aa	0.136253499	AA
NA18995	188	AA	1	aa	0.298411879	nn
NA18991	189	AA	1	aa	0.304771195	AA
NA18996	190	AA	1	aa	0.200588028	nn
NA19003	191	AA	1	aa	0.380180621	AA
NA18502	192	AG	1	ag	0.382866516	AG
NA19153	193	AA	1	aa	0.539780905	AA
NA18857	194	AA	1	aa	0.470844452	AA
NA19223	195	AG	0.9999	ag	0.097936783	AG
NA19201	196	AA	1	aa	0.530767337	AA
NA18504	197	AA	0.9999	aa	0.300464034	AA
NA18870	198	AA	1	aa	0.430029578	AA
NA18863	199	AA	1	aa	0.465548285	AA
NA19145	200	AA	0.9987	aa	0.04266515	AA
NA19137	201	AA	1	aa	0.234354226	AA
NA19238	202	AG	1	ag	0.053916358	nn
NA18500	203	GG	1	gg	0.549731812	GG
NA19144	204	AA	1	aa	0.00789718	AA
NA19203	205	AA	1	aa	0.233510446	AA
NA19200	206	AA	1	aa	0.224331561	AA
NA18855	207	AA	0.9929	aa	0.253794081	AA
NA18505	208	AA	1	aa	0.326082571	AA
NA19202	209	AA	1	aa	0.252654888	AA
NA18501	210	AG	1	ag	0.154342889	AG
NA18861	211	AA	0.9997	aa	0.093580854	AA
NA19193	212	AA	0.9911	aa	0.030820391	AA
NA19143	213	AA	0.9958	aa	0.057279964	AA
NA18517	214	AG	0.9997	ag	0.078474058	AG
NA18862	215	AA	1	aa	0.240642972	AA
NA18856	216	AA	1	aa	0.198165912	nn
NA19239	217	AA	1	aa	0.153879581	AA
NA19240	218	AA	1	aa	0.106698509	AA
NA18856	219	AA	1	aa	0.411009036	AA
NA18503	220	AA	0.75	aa	0.010622827	AA

	MACGT discrepancy					
	LDA discrepancy					
NA	LDA output were not g					
XY	Manual calls are given					
				**365063**		
**Coriell**	**Sample**	**LDA Call**	**LDA quality score**	**MACGT Call**	**MACGT quality score**	**HapMap Call**
NA18871	221	AA	1	aa	0.085284521	AA
NA19221	222	GG	1	gg	0.161909733	GG
NA19209	223	AA	1	aa	0.142582779	AA
NA19152	224	AG	1	ag	0.065476975	AG
NA18515	225	GG	1	gg	0.083670798	GG
NA19238	226	AG	1	ag	0.074225866	AG
NA19154	227	AG	1	ag	0.225343435	AG
NA19210	228	AG	1	ag	0.053747923	AG
NA19211	229	AG	0.9999	ag	0.157842661	AG
NA18862	230	AA	1	aa	0.120335696	nn
NA18872	231	AA	1	aa	0.42269748	AA
NA19139	232	AA	1	aa	0.266928478	AA
NA19222	233	GG	1	gg	0.503834399	GG
NA19194	234	AA	1	aa	0.399133535	AA
NA19138	235	AG	0.9993	***AG***	***0***	AG
NA19204	236	AA	0.9996	aa	0.086046099	AA
NA18516	237	GG	1	gg	0.005221203	GG
NA19205	238	AA	1	aa	0.428053189	AA
NA19192	239	AA	1	aa	0.497783523	AA
NA18912	240	AA	1	aa	0.237043217	nn
NA18508	241	AA	1	aa	0.397387688	AA
NA19142	242	GG	1	gg	0.123218144	GG
NA18852	243	AG	0.9969	ag	0.216383371	AG
NA18507	244	AA	0.9999	aa	0.146218387	AA
blank	245	NN	0.5	***NN***	***0***	nn
NA19101	246	AG	0.9999	ag	0.181134055	AG
NA19172	247	AA	1	aa	0.37964548	AA
NA19160	248	AA	1	aa	0.445892007	AA
NA19129	249	AA	1	aa	0.293642068	AA
NA18913	250	AG	0.9988	ag	0.02957685	AG
NA19120	251	AA	1	aa	0.140959975	AA
NA19159	252	AG	1	ag	0.129617502	AG
NA18523	253	AA	1	aa	0.36962627	AA
NA19102	254	AA	1	aa	0.256445123	AA
NA19092	255	AA	1	aa	0.39326499	AA
NA18521	256	AG	1	***AG***	***0***	AG
NA18912	257	AA	1	aa	0.135777622	AA
NA19206	258	AA	1	aa	0.391519366	AA
NA19128	259	AA	1	aa	0.426772748	AA
NA18859	260	AA	1	aa	0.479447044	nn
NA19094	261	AA	1	aa	0.496714495	AA
NA19171	262	AA	1	aa	0.519644824	AA
NA18860	263	AG	1	***AG***	***0***	AG
NA18858	264	AG	1	ag	0.036358667	AG

	MACGT discrepancy					
	LDA discrepancy					
NA	LDA output were not g					
XY	Manual calls are given					
		365063				
**Coriell**	**Sample**	**LDA Call**	**LDA quality score**	**MACGT Call**	**MACGT quality score**	**HapMap Call**
NA19099	265	AA	1	aa	0.213533943	AA
NA18506	266	AA	0.9997	aa	0.03405182	AA
NA19131	267	AA	0.999	aa	0.023315636	AA
NA19116	268	AA	0.902	aa	0.040595015	AA
NA19161	269	AG	1	ag	0.016762213	AG
NA19140	270	AG	1	ag	0.03230148	AG
NA19207	271	AA	1	aa	0.183668491	AA
NA19141	272	GG	1	gg	0.015786944	GG
NA18854	273	AA	0.9998	aa	0.104517174	AA
NA19098	274	AA	0.9268	aa	0.070331033	AA
NA18522	275	AG	1	ag	0.015693597	AG
NA18914	276	AG	0.9361	ag	0.077541373	AG
NA18853	277	AA	1	aa	0.03963884	AA
NA18859	278	AA	0.8881	aa	0.099683721	AA
NA19100	279	AA	0.7768	***AA***	***0***	AA
NA19093	280	AA	1	aa	0.05130759	AA
NA19132	281	AA	0.9999	aa	0.070747121	AA
NA19130	282	AA	0.8487	aa	0.030687594	AA
NA19208	283	AA	0.8461	aa	0.036308742	AA
NA19127	284	AA	1	aa	0.254660807	AA
NA19173	285	AA	1	aa	0.298590235	AA
NA19119	286	AG	1	ag	0.006879392	AG
NA19103	287	AG	1	***AG***	***0***	AG
	Discrepancies	0		0		
	NNs	0		0		
	Automated Calls	278		262		
	Manual Calls	0		16		
	Concordant Calls	259		259		259

	MACGT discrepancy					
	LDA discrepancy					
NA	LDA output were not g					
XY	Manual calls are given					
		3776720				
**Coriell**	**Sample**	**LDA Call**	**LDA quality score**	**MACGT Call**	**MACGT quality score**	**HapMap Call**
NA12753	1		NA	GA	0.347708528	
NA12875	2	AA	0.9859	aa	0.038728148	AA
NA12044	3	AA	0.9927	aa	0.238534361	AA
NA06993	4	AA	0.9808	aa	0.139922229	AA
NA12716	5	GG	0.7865	gg	0.156688727	GG
NA12760	6	AA	0.9964	aa	0.004521312	AA
NA07348	7	AA	0.9879	aa	0.035651012	AA
NA12707	8	GG	0.9632	gg	0.530746445	GG
NA12156	9		NA	GA	0.160431172	
NA11992	10	GA	0.9412	ga	0.188395441	GA
NA12239	11	AA	0.9963	aa	0.005928453	AA
NA12878	12	GA	0.9369	ga	0.03778763	GA
NA11993	13	AA	0.8926	aa	0.086790106	AA
NA12750	14	GA	0.9899	ga	0.277557771	GA
NA12146	15	GA	0.991	ga	0.247655742	GA
NA11839	16	AA	0.926	aa	0.099841539	AA
NA12156.dup	17	GA	0.975	ga	0.366573285	nn
NA11829	18		NA	GA	0.431570393	
NA12154	19	AA	0.9787	aa	0.22308077	AA
NA10856	20	GA	0.8748	ga	0.281148935	GA
NA06985	21	AA	0.9935	aa	0.112176597	AA
NA11831	22	AA	0.9258	aa	0.116931073	AA
NA12003.dup	23	GA	0.9935	ga	0.125198524	nn
NA10859	24	GA	0.988	ga	0.233972829	GA
NA07056	25	GA	0.8592	ga	0.342372019	GA
NA07022	26	AA	0.9914	aa	0.561421732	AA
NA12762	27		NA	AA	0.549882497	
NA12874	28	GA	0.8242	ga	0.063499777	GA
NA12003	29	GA	0.9311	ga	0.324011467	GA
NA10835	30	GA	0.8768	ga	0.513575527	GA
NA11882	31	GA	0.8905	ga	0.368983411	GA
NA07034	32	AA	0.9931	aa	0.094310145	AA
NA12056	33	AA	0.9953	aa	0.234921346	AA
NA12145	34	GA	0.9878	ga	0.155531952	GA
NA07019	35	GA	0.9931	ga	0.23401502	GA
NA06991	36		NA	AA	0.343940876	
NA12761	37	AA	0.9328	aa	0.15731177	AA
NA06994	38	GG	0.8178	gg	0.263774251	GG
NA12864	39	GA	0.9861	ga	0.248501897	GA
NA07055	40	GA	0.9894	ga	0.139664051	GA
NA10863	41	GA	0.9903	ga	0.17286952	GA
NA12763	42	GA	0.9907	ga	0.332880711	GA
NA10831	43	GG	0.9054	gg	0.338195373	GG
NA11840	44	AA	0.9892	aa	0.337487861	AA

	MACGT discrepancy					
	LDA discrepancy					
NA	LDA output were not g					
XY	Manual calls are given					
		**3776720**				
**Coriell**	**Sample**	**LDA Call**	**LDA quality score**	**MACGT Call**	**MACGT quality score**	**HapMap Call**
NA10830	45	AA	0.9836	aa	0.456563087	AA
NA11993.dup	46	AA	0.9833	aa	0.122250317	nn
NA12751	47	AA	0.9856	aa	0.368642796	AA
NA12814	48	AA	0.987	aa	0.093942819	AA
NA10857	49	GA	0.9901	ga	0.360188202	GA
NA07357	50	AA	0.9857	aa	0.137248834	AA
NA07000	51	AA	0.9968	aa	0.137414819	AA
NA12802	52	AA	0.9957	aa	0.203633968	AA
NA10855	53	AA	0.9971	aa	0.292817924	AA
NA11995	54		NA	GA	0.103711686	
NA10839	55	AA	0.9995	aa	0.174560865	AA
NA10847	56	GA	0.9637	ga	0.299411985	GA
NA12006	57	AA	0.9996	aa	0.114713591	AA
NA12144	58	GA	0.9515	ga	0.649247885	GA
NA11832	59	AA	0.9991	aa	0.069117986	AA
NA12813	60	AA	0.9997	aa	0.066920493	AA
blank	61	AA	0.75	***NN***	***0***	nn
NA12891	62	GA	0.9517	ga	0.654435046	GA
NA12717	63		NA	GG	0.618948253	
NA10861	64	AA	0.9997	aa	0.24110953	AA
NA12004	65	GA	0.8801	ga	0.053774442	GA
NA07048	66	GA	0.9155	ga	0.055609673	GA
NA12801	67	AA	0.9999	aa	0.019877753	AA
NA11830	68	AA	0.9994	aa	0.013356288	AA
NA12057	69	GA	0.9067	ga	0.329118174	GA
NA11994	70		NA	AA	0.518010656	
NA12740	71	GA	0.9678	ga	0.475069848	GA
NA12005	72	AA	0.8658	aa	0.073391437	AA
NA07345	73	AA	0.9002	aa	0.131319682	AA
NA10854	74	AA	0.9656	aa	0.412846483	AA
NA12872	75	GA	0.9679	ga	0.400711146	GA
NA12865	76	AA	0.9927	aa	0.560081351	AA
NA10860	77		NA	AA	0.278465321	
NA12873	78	AA	0.9921	aa	0.264162079	AA
NA12752	79	AA	0.996	aa	0.517745704	AA
NA10851	80	AA	0.9969	aa	0.461806349	AA
NA07029	81	GA	0.9578	ga	0.366663284	GA
NA12248	82	GA	0.8583	ga	0.262970606	GA
NA12155	83	GA	0.8187	ga	0.199319137	GA
NA10838	84	GG	0.9885	gg	0.324425714	GG
NA12812	85	AA	0.9969	aa	0.31845257	AA
NA12248.dup	86	GA	0.8313	ga	0.158273245	nn
NA12043	87	GA	0.8491	ga	0.267437003	GA
NA12234	88	GA	0.9418	ga	0.378226806	GA

	MACGT discrepancy					
	LDA discrepancy					
NA	LDA output were not g					
XY	Manual calls are given					
		**3776720**				
**Coriell**	**Sample**	**LDA Call**	**LDA quality score**	**MACGT Call**	**MACGT quality score**	**HapMap Call**
NA12892	89	GA	0.8968	ga	0.271110832	GA
NA10846	90	AA	0.9945	aa	0.442501558	AA
NA06993.dup	91	AA	0.9795	aa	0.381179925	nn
NA12249	92	AA	0.9036	aa	0.152680197	AA
NA11881	93	AA	0.9621	aa	0.226828717	AA
NA12815	94	AA	0.9569	*AA*	*6.24E-04*	AA
NA12264	95	GA	0.964	ga	0.576968871	GA
NA18526	96	GG	0.9354	gg	0.425304038	GG
NA18562	97	AA	0.9851	aa	0.264819027	AA
NA18545	98	GG	0.8927	gg	0.200867292	GG
NA18609	99	GG	0.9251	gg	0.51314215	GG
NA18566	100	AA	0.9593	aa	0.095835587	AA
NA18621	101	GA	0.9402	ga	0.573083066	GA
NA18577	102	GA	0.93	ga	0.406909518	GA
NA18635	103	GG	0.9141	gg	0.453262527	GG
NA18524	104	GA	0.9626	ga	0.402163015	GA
NA18537	105	GG	0.9175	gg	0.562460172	GG
NA18572	106	GA	0.9135	ga	0.47796798	GA
NA18552	107	GA	0.9305	ga	0.187619424	GA
NA18563	108	GA	0.911	ga	0.487967266	GA
NA18594	109	AA	0.962	aa	0.21465198	nn
NA18624	110	AA	0.9967	aa	0.429337283	AA
NA18592	111	AA	0.9893	aa	0.069606628	AA
NA18529	112	GA	0.8626	ga	0.394317185	GA
NA18603	113	GG	0.9675	gg	0.586517053	nn
NA18547	114	GG	0.9797	gg	0.316221577	GG
NA18611	115		NA	GG	0.374617375	
NA18570	116	AA	0.9951	aa	0.118867338	AA
NA18622	117	AA	0.9984	aa	0.374912252	AA
NA18579	118	GA	0.913	ga	0.084554577	GA
NA18636	119	GA	0.8957	ga	0.43088258	GA
NA18558	120	GA	0.9232	ga	0.37879134	GA
NA18540	121	GG	0.9926	gg	0.454298207	GG
NA18609	122	GG	0.9773	gg	0.543397764	nn
NA18555	123	AA	0.9952	aa	0.096992454	AA
NA18612	124	GG	0.9803	gg	0.522282316	GG
NA18573	125	GG	0.9898	gg	0.249445432	GG
NA18632	126	GG	0.8599	gg	0.541737496	GG
NA18593	127	AA	0.9959	aa	0.573071242	AA
NA18532	128	GA	0.9514	ga	0.502207787	GA
NA18605	129	GA	0.957	ga	0.476927087	GA
NA18550	130	GA	0.9769	ga	0.510126414	GA
NA18603	131	GG	0.9346	gg	0.550236845	GG
NA18571	132	AA	0.9905	aa	0.459946725	AA

	MACGT discrepancy					
	LDA discrepancy					
NA	LDA output were not g					
XY	Manual calls are given					
		**3776720**				
**Coriell**	**Sample**	**LDA Call**	**LDA quality score**	**MACGT Call**	**MACGT quality score**	**HapMap Call**
NA18623	133	GG	0.9903	gg	0.320389158	GG
NA18582	134	GA	0.8613	ga	0.364005999	GA
NA18637	135	GA	0.8087	ga	0.136326057	GA
NA18561	136	GG	0.9971	gg	0.30920851	GG
NA18542	137	GA	0.8183	ga	0.269189041	GA
NA18608	138	GG	0.9342	gg	0.411341918	GG
NA18564	139	GA	0.9047	ga	0.236958284	GA
NA18620	140	GA	0.9324	ga	0.510444613	GA
NA18576	141	AA	0.9919	aa	0.435820842	AA
NA18633	142	GA	0.8818	ga	0.358312565	GA
NA18594	143	AA	0.9972	aa	0.460752748	AA
NA18942	144	GA	0.9711	ga	0.655055887	GA
NA18945	145	GG	0.921	***GG***	***6.99E-04***	GG
NA18964	146	GA	0.9372	ga	0.724112898	GA
NA18961	147	GA	0.9666	ga	0.002026608	GA
NA18967	148	AA	0.982	aa	0.259594583	AA
NA18981	149	AA	0.9965	aa	0.352998978	AA
NA18994	150	AA	0.9948	aa	0.582061012	AA
NA18998	151	AA	0.9941	aa	0.032945891	AA
NA18940	152	GA	0.8784	ga	0.331617694	GA
NA18949	153	GA	0.863	ga	0.237150122	GA
NA18953	154	GG	0.8891	gg	0.077713169	GG
NA18972	155	GA	0.8243	ga	0.181862152	GA
NA18976	156	GG	0.9492	gg	0.528940966	GG
NA18971	157	AA	0.9992	aa	0.129524051	AA
blank	158	NN	0.7497	***NN***	***0***	nn
NA19000	159	GA	0.9069	ga	0.324770384	GA
NA18951	160	AA	0.9917	aa	0.071231982	nn
NA18948	161	GA	0.7835	ga	0.17480287	GA
NA18968	162	GG	0.9552	gg	0.44822945	GG
NA18965	163	AA	0.9962	aa	0.017894202	AA
NA18978	164	GA	0.8934	ga	0.36675813	GA
NA18974	165	GA	0.8778	ga	0.159988808	GA
NA18992	166	GA	0.8451	ga	0.083268543	GA
NA19005	167	GA	0.9874	ga	0.312138993	GA
NA18943	168	GG	0.7797	gg	0.014767672	GG
NA18951	169	AA	0.9994	aa	0.14772553	AA
NA18959	170	GA	0.967	ga	0.484019922	GA
NA18973	171	GA	0.964	ga	0.538917929	GA
NA18970	172	AA	0.9989	aa	0.360125389	AA
NA18987	173	AA	0.9991	aa	0.19459797	AA
NA18995	174	GA	0.9727	ga	0.631999905	GA
NA18999	175	GG	0.9109	gg	0.33309765	GG
NA18947	176	GA	0.9173	ga	0.539732622	GA

	MACGT discrepancy					
	LDA discrepancy					
NA	LDA output were not g					
XY	Manual calls are given					
		3776720				
**Coriell**	**Sample**	**LDA Call**	**LDA quality score**	**MACGT Call**	**MACGT quality score**	**HapMap Call**
NA18952	177	GA	0.9279	ga	0.15073054	GA
NA18969	178	GA	0.9023	ga	0.442462567	GA
NA18966	179		NA	GG	0.530520691	
NA18980	180	GG	0.927	gg	0.097180675	GG
NA18990	181	GA	0.9503	ga	0.434129344	GA
NA18997	182	GA	0.8977	ga	0.452816102	GA
NA19007	183	GA	0.9263	ga	0.412425476	GA
NA18944	184	GA	0.9465	ga	0.104677436	GA
NA18956	185	GG	0.7963	gg	0.092861122	GG
NA18960	186	GA	0.9587	ga	0.546762425	GA
NA18975	187	GA	0.9716	ga	0.150150904	GA
NA18995	188	GA	0.9588	ga	0.386177196	nn
NA18991	189	GA	0.9591	ga	0.573449275	GA
NA18996	190	GA	0.9609	ga	0.005011494	nn
NA19003	191	GA	0.9603	ga	0.527412472	GA
NA18502	192	GA	0.9371	ga	0.130106289	GA
NA19153	193	AA	0.9967	aa	0.180144472	AA
NA18857	194	AA	0.9909	aa	0.04918488	AA
NA19223	195	GG	0.962	gg	0.595689624	GG
NA19201	196	GA	0.9418	ga	0.454695868	GA
NA18504	197	GA	0.9443	ga	0.007906259	GA
NA18870	198	GA	0.916	ga	0.352745869	GA
NA18863	199	AA	0.9956	aa	0.032931097	AA
NA19145	200	GG	0.848	gg	0.386116582	GG
NA19137	201	AA	0.9909	aa	0.01244373	AA
NA19238	202	GA	0.9064	ga	0.002369702	nn
NA18500	203	AA	0.9953	aa	0.02325312	AA
NA19144	204	GG	0.9276	gg	0.195480254	GG
NA19203	205	AA	0.9879	aa	0.02055413	AA
NA19200	206	AA	0.9939	aa	0.401578381	AA
NA18855	207	GA	0.9712	ga	0.183979559	GA
NA18505	208	GA	0.8937	ga	0.068082576	GA
NA19202	209	GA	0.8912	ga	0.245906233	GA
NA18501	210	AA	0.9911	aa	0.032820054	AA
NA18861	211	AA	0.9939	aa	0.002418864	AA
NA19193	212	GG	0.9952	gg	0.124243168	GG
NA19143	213	GA	0.8617	ga	0.085815411	GA
NA18517	214	AA	0.9994	aa	0.060717478	AA
NA18862	215	GA	0.8421	ga	0.244142786	GA
NA18856	216	AA	0.9967	aa	0.013835788	nn
NA19239	217	AA	0.9962	*AA*	*0*	AA
NA19240	218	AA	0.9987	aa	0.181160217	AA
NA18856	219	AA	0.9987	*AA*	*6.60E-04*	AA
NA18503	220	GA	0.9936	ga	0.024693239	GA

	MACGT discrepancy					
	LDA discrepancy					
NA	LDA output were not g					
XY	Manual calls are given					
		**3776720**				
**Coriell**	**Sample**	**LDA Call**	**LDA quality score**	**MACGT Call**	**MACGT quality score**	**HapMap Call**
NA18871	221	GA	0.9826	ga	0.301237243	GA
NA19221	222	GA	0.9926	ga	0.35122148	GA
NA19209	223	GA	0.992	ga	0.106301271	GA
NA19152	224	AA	0.9983	aa	0.241292897	AA
NA18515	225	GA	0.9773	ga	0.167894003	GA
NA19238	226	GA	0.9846	ga	0.370436091	GA
NA19154	227	AA	0.9944	aa	0.113298167	AA
NA19210	228	AA	0.9964	aa	0.030626267	AA
NA19211	229	AA	0.9977	aa	0.063989358	AA
NA18862	230	GA	0.9573	ga	0.364266526	nn
NA18872	231	GA	0.9449	ga	0.378325129	GA
NA19139	232	AA	0.9948	aa	0.117995947	AA
NA19222	233	GA	0.9387	ga	0.230573415	GA
NA19194	234	GG	0.9323	gg	0.516338098	GG
NA19138	235	GA	0.8876	ga	0.006649432	GA
NA19204	236	AA	0.9994	aa	0.160265723	AA
NA18516	237	GA	0.9807	ga	0.084825698	GA
NA19205	238	AA	0.9908	aa	0.088143483	AA
NA19192	239	GA	0.9704	ga	0.072391575	GA
NA18912	240	GG	0.7564	gg	0.048408123	nn
NA18508	241	AA	0.9773	aa	0.180015358	AA
NA19142	242	AA	0.9959	aa	0.05041222	AA
NA18852	243	AA	0.9933	aa	0.22044307	AA
NA18507	244	GA	0.9445	ga	0.095763464	GA
blank	245	NN	0.7398	***NN***	***0***	nn
NA19101	246	AA	0.8387	aa	0.020837208	AA
NA19172	247	AA	0.9963	aa	0.013782516	AA
NA19160	248	AA	0.9972	aa	0.033555295	AA
NA19129	249	AA	0.9866	aa	0.022303297	AA
NA18913	250	AA	0.9935	aa	0.020722441	AA
NA19120	251	AA	0.9967	***AA***	***0***	AA
NA19159	252	GG	0.9841	gg	0.210010138	GG
NA18523	253	AA	0.993	aa	0.251472254	AA
NA19102	254	AA	0.9965	***AA***	***0***	AA
NA19092	255	AA	0.9936	aa	0.254112403	AA
NA18521	256	GA	0.9739	ga	0.333393897	GA
NA18912	257	GG	0.8394	gg	0.052105018	GG
NA19206	258	GA	0.9777	ga	0.514819623	GA
NA19128	259	AA	0.9939	aa	0.435465188	AA
NA18859	260	AA	0.9879	aa	0.205290677	nn
NA19094	261	AA	0.9917	aa	0.31916253	AA
NA19171	262	GA	0.957	ga	0.621736252	GA
NA18860	263	AA	0.998	aa	0.192550101	AA
NA18858	264	AA	0.9979	aa	0.192582261	AA

	MACGT discrepancy					
	LDA discrepancy					
NA	LDA output were not g					
XY	Manual calls are given					
		**3776720**				
**Coriell**	**Sample**	**LDA Call**	**LDA quality score**	**MACGT Call**	**MACGT quality score**	**HapMap Call**
NA19099	265	AA	0.9994	aa	0.046379631	AA
NA18506	266	AA	0.9983	aa	0.004850241	AA
NA19131	267	GA	0.9454	ga	0.243381742	GA
NA19116	268	AA	0.9981	aa	0.012568003	AA
NA19161	269	GA	0.9195	ga	0.058839695	GA
NA19140	270	AA	0.9988	aa	0.058691946	AA
NA19207	271	AA	0.9975	aa	0.040485006	AA
NA19141	272	GA	0.9554	ga	0.087781948	GA
NA18854	273	AA	0.9993	aa	0.023859565	AA
NA19098	274	GG	0.9617	gg	0.465988806	GG
NA18522	275	GA	0.9505	ga	0.062688503	GA
NA18914	276	GA	0.8912	ga	0.172685787	GA
NA18853	277	AA	0.9948	aa	0.291977342	AA
NA18859	278	AA	0.9981	***AA***	***0***	AA
NA19100	279	GA	0.9475	ga	0.161306123	GA
NA19093	280	AA	0.9988	aa	0.021499489	AA
NA19132	281	GA	0.9432	ga	0.017176561	GA
NA19130	282	AA	0.9953	aa	0.004157813	AA
NA19208	283	AA	0.9978	aa	0.037570661	AA
NA19127	284	GA	0.8612	ga	0.10494572	GA
NA19173	285	GA	0.8767	ga	0.198969538	GA
NA19119	286	AA	0.999	aa	0.030472291	AA
NA19103	287	AA	0.9958	aa	0.119068668	AA
	Discrepancies	0		0		
	NNs	0		1		
	Automated Calls	276		266		
	Manual Calls	0		10		
	Concordant Calls	257		257		257

	MACGT discrepancy					
	LDA discrepancy					
NA	LDA output were not g					
XY	Manual calls are given					
		3899706				
**Coriell**	**Sample**	**LDA Call**	**LDA quality score**	**MACGT Call**	**MACGT quality score**	**HapMap Call**
NA12753	1		NA	CG	0.376888099	
NA12875	2	CG	1	cg	0.224077397	CG
NA12044	3	CG	1	cg	0.438260458	CG
NA06993	4	GG	0.9987	gg	0.121458501	GG
NA12716	5	CG	0.9977	cg	0.213282817	CG
NA12760	6	CC	0.9662	cc	0.335928607	CC
NA07348	7	CG	0.9885	cg	0.330963297	CG
NA12707	8	GG	0.8029	gg	0.036115496	GG
NA12156	9		NA	CG	0.170110253	
NA11992	10	CG	0.9999	cg	0.173263629	CG
NA12239	11	CC	0.9933	cc	0.079275182	CC
NA12878	12	CG	0.9997	cg	0.412443012	CG
NA11993	13	CC	0.9984	cc	0.116950211	CC
NA12750	14	CG	0.9994	cg	0.056816081	CG
NA12146	15	CC	1	*CC*	*0*	CC
NA11839	16	CC	0.9977	cc	0.152759418	CC
NA12156.dup	17	CG	0.9994	cg	0.377126817	nn
NA11829	18		NA	CG	0.213645734	
NA12154	19	GG	0.9998	gg	0.00194887	GG
NA10856	20	CG	0.9998	cg	0.171240997	CG
NA06985	21	GG	0.9998	gg	0.002485441	GG
NA11831	22	CC	1	cc	0.232526882	CC
NA12003.dup	23	CG	1	cg	0.140868806	nn
NA10859	24	CC	0.9973	cc	0.161407276	CC
NA07056	25	CC	0.9952	cc	0.148072936	CC
NA07022	26	CC	0.9991	cc	0.454691205	CC
NA12762	27		NA	CC	0.464664672	
NA12874	28	CG	0.9999	cg	0.124681804	CG
NA12003	29	CG	1	cg	0.275237509	CG
NA10835	30	CC	1	cc	0.139472237	CC
NA11882	31	CC	0.9885	cc	0.242263574	CC
NA07034	32	CG	1	cg	0.23748763	CG
NA12056	33	CG	1	cg	0.62336618	CG
NA12145	34	CG	0.9996	cg	0.305251036	CG
NA07019	35	CC	1	cc	0.347224706	CC
NA06991	36		NA	GG	0.369357553	
NA12761	37	CG	1	cg	0.120944077	CG
NA06994	38	CC	0.9983	cc	0.2817553	CC
NA12864	39	CC	0.9999	cc	0.266267941	CC
NA07055	40	CC	1	cc	0.410789716	CC
NA10863	41	CC	0.9986	cc	0.461434394	CC
NA12763	42	CG	0.9998	cg	0.367572577	CG
NA10831	43	CG	0.9999	cg	0.249679182	CG
NA11840	44	GG	1	gg	0.06716393	GG

	MACGT discrepancy					
	LDA discrepancy					
NA	LDA output were not g					
XY	Manual calls are given					
		**3899706**				
**Coriell**	**Sample**	**LDA Call**	**LDA quality score**	**MACGT Call**	**MACGT quality score**	**HapMap Call**
NA10830	45	CG	1	cg	0.27562096	CG
NA11993.dup	46	CC	1	cc	0.057994027	nn
NA12751	47	GG	1	*GG*	*0*	GG
NA12814	48	CG	1	cg	0.336734967	CG
NA10857	49	CG	0.9998	cg	0.318245501	CG
NA07357	50	CG	0.9997	cg	0.583986876	CG
NA07000	51	CC	1	cc	0.1328518	CC
NA12802	52	CG	0.9996	cg	0.218503137	CG
NA10855	53	CG	1	cg	0.252922216	CG
NA11995	54		NA	CG	0.315870048	
NA10839	55	CC	1	cc	0.175980606	CC
NA10847	56	CC	1	cc	0.359397796	CC
NA12006	57	CC	1	cc	0.283177643	CC
NA12144	58	CG	0.9997	cg	0.376288917	CG
NA11832	59	CG	0.9997	cg	0.454219788	CG
NA12813	60	CG	1	cg	0.315023131	CG
blank	61	GG	0.9018	*CC*	*0*	nn
NA12891	62	CG	1	cg	0.216195513	CG
NA12717	63		NA	CG	0.342830517	
NA10861	64	CC	1	cc	0.12994003	CC
NA12004	65	GG	1	*GG*	*0*	GG
NA07048	66	CC	1	*CC*	*0*	CC
NA12801	67	CC	0.9998	cc	0.084583607	CC
NA11830	68	GG	0.986	gg	0.058251427	GG
NA12057	69	CG	1	cg	0.203602891	CG
NA11994	70		NA	CG	0.473841013	
NA12740	71	GG	0.9813	gg	0.25514396	GG
NA12005	72	CG	1	cg	0.007087899	CG
NA07345	73	CC	0.8086	*NN*	*0*	CC
NA10854	74	CG	0.9991	cg	0.040253026	CG
NA12872	75	CC	0.9982	*CC*	*0*	CC
NA12865	76	CG	0.9985	cg	0.079248439	CG
NA10860	77		NA	CC	0.293425511	
NA12873	78	CG	0.9959	cg	0.278092756	CG
NA12752	79	CG	1	cg	0.022653072	CG
NA10851	80	CG	0.9984	cg	0.284974344	CG
NA07029	81	CC	0.7977	*CC*	*0*	CC
NA12248	82	CC	0.9999	cc	0.00425405	CC
NA12155	83	CC	0.9993	*CC*	*0*	CC
NA10838	84	GG	1	*GG*	*0*	GG
NA12812	85	CC	0.9991	cc	0.002026953	CC
NA12248.dup	86	CC	0.9987	*CC*	*0*	nn
NA12043	87	GG	0.7502	*GG*	*0*	GG
NA12234	88	CC	1	cc	0.329456811	CC

	MACGT discrepancy					
	LDA discrepancy					
NA	LDA output were not g					
XY	Manual calls are given					
		**3899706**				
**Coriell**	**Sample**	**LDA Call**	**LDA quality score**	**MACGT Call**	**MACGT quality score**	**HapMap Call**
NA12892	89	CG	0.9637	***NN***	***0***	CG
NA10846	90	CG	1	cg	0.135051564	CG
NA06993.dup	91	GG	1	***GG***	***0***	nn
NA12249	92	CC	0.9078	***CC***	***0***	CC
NA11881	93	CC	0.8987	***CC***	***0***	CC
NA12815	94	CC	0.76	cc	0.001369283	CC
NA12264	95	CC	1	cc	0.034855406	CC
NA18526	96	CC	0.9992	***CC***	***0***	CC
NA18562	97	CG	0.997	cg	0.363734608	CG
NA18545	98	CC	1	cc	0.214218787	CC
NA18609	99	CC	1	cc	0.385323468	CC
NA18566	100	CC	1	cc	0.247331597	CC
NA18621	101	CC	1	cc	0.268016769	CC
NA18577	102	CG	0.9822	cg	0.146496891	CG
NA18635	103	CC	1	cc	0.185530169	CC
NA18524	104	CC	1	cc	0.483090538	CC
NA18537	105	CC	1	cc	0.220729585	CC
NA18572	106	CC	1	cc	0.165551754	CC
NA18552	107	CC	1	cc	0.17502849	CC
NA18563	108	CC	1	cc	0.140654516	CC
NA18594	109	CC	1	cc	0.185582813	nn
NA18624	110	CC	0.9997	cc	0.446340785	CC
NA18592	111	CC	0.9991	cc	0.173541031	CC
NA18529	112	CC	0.9999	cc	0.350013928	CC
NA18603	113	CC	1	cc	0.449610454	nn
NA18547	114	CC	1	cc	0.278944929	CC
NA18611	115	CC	0.9909	cc	0.00174308	CC
NA18570	116	CC	0.9965	cc	0.253572718	CC
NA18622	117	CC	1	cc	0.401963905	CC
NA18579	118	CC	0.9982	cc	0.007798727	CC
NA18636	119	CC	0.9992	cc	0.313239244	CC
NA18558	120	CC	0.9985	***CC***	***0***	CC
NA18540	121	CC	0.997	cc	0.103014768	CC
NA18609	122	CC	0.9918	cc	0.091822134	nn
NA18555	123	CC	0.9998	***CC***	***0***	CC
NA18612	124	CC	1	cc	0.250635406	CC
NA18573	125	CC	1	cc	0.01316458	CC
NA18632	126	CC	0.9895	cc	0.045844248	CC
NA18593	127	CC	1	***CC***	***0***	CC
NA18532	128	CC	1	cc	0.285109852	CC
NA18605	129	CC	1	cc	0.218767804	CC
NA18550	130	CC	1	cc	0.13480077	CC
NA18603	131	CC	0.9999	cc	0.345142624	CC
NA18571	132	CC	1	***CC***	***0***	CC

	MACGT discrepancy					
	LDA discrepancy					
NA	LDA output were not g					
XY	Manual calls are given					
		**3899706**				
**Coriell**	**Sample**	**LDA Call**	**LDA quality score**	**MACGT Call**	**MACGT quality score**	**HapMap Call**
NA18623	133	CC	0.8041	***CC***	***0***	CC
NA18582	134	CC	1	cc	0.157150695	CC
NA18637	135	CC	1	cc	0.065648978	CC
NA18561	136	CC	0.9997	***CC***	***0***	CC
NA18542	137	CC	0.9997	cc	0.003813814	CC
NA18608	138	CC	1	cc	0.154568324	CC
NA18564	139	CC	1	cc	0.227659334	CC
NA18620	140	CG	0.9999	cg	0.128557135	CG
NA18576	141	CC	1	cc	0.228538195	CC
NA18633	142	CC	0.9999	cc	0.012377281	CC
NA18594	143	CC	1	cc	0.072385891	CC
NA18942	144	CC	1	***CC***	***0***	CC
NA18945	145	CC	1	cc	0.242792015	CC
NA18964	146	CC	1	cc	0.250711631	CC
NA18961	147	CC	1	cc	0.295852545	CC
NA18967	148	CC	1	cc	0.262774376	CC
NA18981	149	CC	1	cc	0.28440742	CC
NA18994	150	CG	0.9997	cg	0.631632718	CG
NA18998	151	CC	0.9999	cc	0.005008233	CC
NA18940	152	CC	0.9999	cc	0.122109004	CC
NA18949	153	CC	1	cc	0.126895679	CC
NA18953	154	CC	0.9999	***CC***	***0***	CC
NA18972	155	CC	0.9999	cc	0.070808109	CC
NA18976	156	CC	1	cc	0.197719543	CC
NA18971	157	CG	0.9988	cg	0.28947406	CG
blank	158	CC	0.8816	***NN***	***0***	nn
NA19000	159	CC	1	cc	0.384193	CC
NA18951	160	CC	1	cc	0.355758922	nn
NA18948	161	CG	1	cg	0.24514953	CG
NA18968	162	CC	1	cc	0.097692305	CC
NA18965	163	CC	0.9972	cc	0.202298109	CC
NA18978	164	CG	0.9997	cg	0.263854417	CG
NA18974	165	CC	0.9808	cc	0.068690087	CC
NA18992	166	CC	0.9999	cc	0.012815866	CC
NA19005	167	CC	1	cc	0.299544359	CC
NA18943	168	CC	1	cc	0.274196775	CC
NA18951	169	CC	1	cc	0.206372801	CC
NA18959	170	CC	1	cc	0.28418545	CC
NA18973	171	CG	1	cg	0.206078398	CG
NA18970	172	CC	1	cc	0.291499059	CC
NA18987	173	CG	0.9999	cg	0.18312474	CG
NA18995	174	CC	0.9962	cc	0.280862807	CC
NA18999	175	CC	1	cc	0.108701212	CC
NA18947	176	CC	1	cc	0.022625085	CC

	MACGT discrepancy					
	LDA discrepancy					
NA	LDA output were not g					
XY	Manual calls are given					
		3899706				
**Coriell**	**Sample**	**LDA Call**	**LDA quality score**	**MACGT Call**	**MACGT quality score**	**HapMap Call**
NA18952	177	CG	0.9999	cg	0.235985885	CG
NA18969	178	CG	0.9999	cg	0.171783458	CG
NA18966	179	CC	1	cc	0.150916321	CC
NA18980	180	GG	0.7997	gg	0.033769417	GG
NA18990	181	CC	0.9998	cc	0.152185552	CC
NA18997	182	CG	0.9999	cg	0.176976168	CG
NA19007	183	CC	0.9432	*CC*	*0*	CC
NA18944	184	CC	0.9945	*CC*	*0*	CC
NA18956	185	CC	0.9999	*CC*	*0*	CC
NA18960	186	CC	1	cc	0.398421258	CC
NA18975	187	CC	0.9999	cc	0.001042686	CC
NA18995	188	CC	0.9933	cc	0.512228365	nn
NA18991	189	CC	1	cc	0.139436746	CC
NA18996	190	CG	1	cg	0.011675291	nn
NA19003	191	CC	1	cc	0.359857004	CC
NA18502	192	CC	0.9998	cc	0.026402196	CC
NA19153	193	CC	1	cc	0.125500248	CC
NA18857	194	CC	1	cc	0.182205507	CC
NA19223	195	CG	1	cg	0.212805466	CG
NA19201	196	CC	0.9998	cc	0.178428469	CC
NA18504	197	CC	1	cc	0.009009203	CC
NA18870	198	CG	0.9999	cg	0.241926994	CG
NA18863	199	CC	1	cc	0.001661442	CC
NA19145	200	GG	1	gg	0.327238936	GG
NA19137	201	GG	0.9951	gg	0.004600936	GG
NA19238	202	GG	0.9951	gg	0.017638822	nn
NA18500	203	CG	0.9998	cg	0.266927202	CG
NA19144	204	GG	0.9555	gg	0.00252848	GG
NA19203	205	CC	0.9999	cc	0.178983888	CC
NA19200	206	GG	0.9932	gg	0.3815609	GG
NA18855	207	CC	0.9996	cc	0.039199602	CC
NA18505	208	CC	0.9999	cc	0.083084883	CC
NA19202	209	CG	0.9988	cg	0.242198508	CG
NA18501	210	CG	1	cg	0.082990045	CG
NA18861	211	CC	0.9999	*CC*	*0*	CC
NA19193	212	CG	0.9979	cg	0.165609307	CG
NA19143	213	CG	0.997	cg	0.121468497	CG
NA18517	214	CG	0.9997	cg	0.13245479	CG
NA18862	215	CC	1	cc	0.030546659	CC
NA18856	216	CC	0.9997	cc	0.011714411	nn
NA19239	217	CC	1	*CC*	*0*	CC
NA19240	218	CG	1	cg	0.436096983	CG
NA18856	219	CC	1	cc	0.04703704	CC
NA18503	220	CC	1	cc	0.180094602	CC

	MACGT discrepancy					
	LDA discrepancy					
NA	LDA output were not g					
XY	Manual calls are given					
		3899706				
**Coriell**	**Sample**	**LDA Call**	**LDA quality score**	**MACGT Call**	**MACGT quality score**	**HapMap Call**
NA18871	221	CC	1	cc	0.435358682	CC
NA19221	222	CG	0.9986	cg	0.494830269	CG
NA19209	223	CG	0.9994	cg	0.333138089	CG
NA19152	224	CC	0.9999	cc	0.006169687	CC
NA18515	225	CG	0.9997	cg	0.402231846	CG
NA19238	226		NA	GG	0.347955485	
NA19154	227	CC	1	cc	0.280759948	CC
NA19210	228	CC	0.992	*CC*	*0*	CC
NA19211	229	CC	0.9999	cc	0.248216233	CC
NA18862	230	CC	1	cc	0.246730298	nn
NA18872	231	CG	1	cg	0.383560645	CG
NA19139	232	CG	0.9999	cg	0.386768136	CG
NA19222	233	CG	1	cg	0.17628926	CG
NA19194	234	CG	1	cg	0.462158512	CG
NA19138	235	CC	0.9991	*CC*	*0*	CC
NA19204	236	CC	1	cc	0.0414111	CC
NA18516	237	CG	0.9999	cg	0.194920383	CG
NA19205	238	CC	0.9998	*CC*	*0*	CC
NA19192	239	CC	0.9832	cc	0.166484515	CC
NA18912	240	CG	0.9997	cg	0.255574029	nn
NA18508	241	CC	0.9938	cc	0.114970535	CC
NA19142	242	CG	0.9999	cg	0.145221379	CG
NA18852	243		NA	GG	0.379568892	
NA18507	244	GG	0.9937	gg	0.002780614	GG
blank	245	CC	0.7654	*NN*	*0*	nn
NA19101	246	CC	0.9951	cc	0.271351499	CC
NA19172	247	CC	0.99	cc	0.235260298	CC
NA19160	248	CC	1	cc	0.123653939	CC
NA19129	249	CC	1	cc	0.022071855	CC
NA18913	250	CG	1	cg	0.28735554	CG
NA19120	251	CC	0.9862	*CC*	*0*	CC
NA19159	252	CG	0.9988	cg	0.214240284	CG
NA18523	253	CC	0.9949	*CC*	*0*	CC
NA19102	254	CC	0.9895	*CC*	*5.00E-04*	CC
NA19092	255	CC	1	cc	0.536611522	CC
NA18521	256	CC	0.9999	cc	0.367826944	CC
NA18912	257	CG	0.9999	cg	0.46370447	CG
NA19206	258	CC	0.9985	cc	0.252065574	CC
NA19128	259	CC	0.9999	cc	0.488631788	CC
NA18859	260	CC	1	cc	0.215504629	nn
NA19094	261	CC	1	cc	0.453594928	CC
NA19171	262	CC	0.9999	cc	0.402564182	CC
NA18860	263	CC	1	cc	0.104163856	CC
NA18858	264	CG	1	cg	0.528140229	CG

	MACGT discrepancy					
	LDA discrepancy					
NA	LDA output were not g					
XY	Manual calls are given					
		3899706				
**Coriell**	**Sample**	**LDA Call**	**LDA quality score**	**MACGT Call**	**MACGT quality score**	**HapMap Call**
NA19099	265	CC	1	cc	0.041794639	CC
NA18506	266	CG	1	cg	0.258188647	CG
NA19131	267	CG	0.9998	cg	0.480659861	CG
NA19116	268	CC	1	cc	0.016266031	CC
NA19161	269	CG	1	cg	0.355419299	CG
NA19140	270	CG	0.9999	cg	0.519298722	CG
NA19207	271	CG	0.9999	cg	0.103416898	CG
NA19141	272	CG	1	cg	0.318887804	CG
NA18854	273	CG	1	cg	0.408111879	CG
NA19098	274	GG	1	gg	0.294534925	GG
NA18522	275	CC	0.9999	cc	0.035061489	CC
NA18914	276	CC	0.9999	cc	0.00197534	CC
NA18853	277	CC	1	***CC***	***0***	CC
NA18859	278	CC	0.9997	cc	0.0516787	CC
NA19100	279	CG	0.9999	cg	0.46823882	CG
NA19093	280	CG	1	cg	0.300534856	CG
NA19132	281	CC	0.9999	cc	0.002030624	CC
NA19130	282	CG	0.9991	cg	0.309498297	CG
NA19208	283	CG	1	cg	0.331960312	CG
NA19127	284	CC	1	cc	0.102321159	CC
NA19173	285	CC	1	***CC***	***0***	CC
NA19119	286	CC	1	cc	0.194262696	CC
NA19103	287	CC	1	cc	0.121021893	CC
	Discrepancies	0		0		
	NNs	-2		2		
	Automated Calls	276		236		
	Manual Calls	0		40		
	Concordant Calls	257		255		257

	MACGT discrepancy					
	LDA discrepancy					
NA	LDA output were not g					
XY	Manual calls are given					
				4606154		
**Coriell**	**Sample**	**LDA Call**	**LDA quality score**	**MACGT Call**	**MACGT quality score**	**HapMap Call**
NA12753	1		NA	GG	0.409740466	
NA12875	2	AG	1	ag	0.50671689	AG
NA12044	3	AG	1	ag	0.524117451	AG
NA06993	4	AG	1	ag	0.29909424	AG
NA12716	5	AA	1	aa	0.204070108	AA
NA12760	6	GG	1	gg	0.308373347	GG
NA07348	7	GG	1	gg	0.362258766	GG
NA12707	8	AG	1	ag	0.276391291	AG
NA12156	9		NA	AG	0.283842737	
NA11992	10	AA	1	aa	0.355315961	AA
NA12239	11	AG	1	ag	0.508943563	AG
NA12878	12	AA	1	aa	0.021636875	AA
NA11993	13	GG	1	gg	0.084432418	GG
NA12750	14	AA	0.7506	aa	0.039397197	AA
NA12146	15	AG	1	ag	0.157624874	AG
NA11839	16	AG	1	ag	0.168040457	AG
NA12156.dup	17	AG	1	ag	0.281795037	nn
NA11829	18		NA	AA	0.482329787	
NA12154	19	GG	1	gg	0.129955182	GG
NA10856	20	AA	0.9841	aa	0.023270487	AA
NA06985	21	GG	1	gg	0.175951957	GG
NA11831	22	GG	0.759	gg	0.067834145	GG
NA12003.dup	23	AG	1	ag	0.170237131	nn
NA10859	24	AG	1	ag	0.190105488	AG
NA07056	25	AG	1	ag	0.348167294	AG
NA07022	26	AA	1	aa	0.248370123	AA
NA12762	27		NA	AG	0.456576647	
NA12874	28	GG	1	gg	0.251097058	GG
NA12003	29	AG	1	ag	0.135933008	AG
NA10835	30	AG	1	ag	0.155330934	AG
NA11882	31	AG	1	ag	0.191519075	AG
NA07034	32	GG	1	gg	0.008834166	GG
NA12056	33	GG	1	gg	0.208451805	GG
NA12145	34	GG	1	gg	0.528980578	GG
NA07019	35	AA	0.9986	aa	0.26895865	AA
NA06991	36		NA	GG	0.596749293	
NA12761	37	GG	1	gg	0.491766953	GG
NA06994	38	GG	1	gg	0.480840129	GG
NA12864	39	AG	1	ag	0.539202789	AG
NA07055	40	GG	1	gg	0.585926518	GG
NA10863	41	AG	1	ag	0.474517279	AG
NA12763	42	GG	1	gg	0.429342318	GG
NA10831	43	AG	1	ag	0.385073611	AG
NA11840	44	AG	1	ag	0.34778457	AG

	MACGT discrepancy					
	LDA discrepancy					
NA	LDA output were not g					
XY	Manual calls are given					
		**4606154**				
**Coriell**	**Sample**	**LDA Call**	**LDA quality score**	**MACGT Call**	**MACGT quality score**	**HapMap Call**
NA10830	45	GG	1	gg	0.212945927	GG
NA11993.dup	46	GG	1	gg	0.370628847	nn
NA12751	47	GG	1	gg	0.36128517	GG
NA12814	48	AA	0.9993	aa	0.227128723	AA
NA10857	49	AA	0.9999	aa	0.247398377	AA
NA07357	50	GG	1	gg	0.319741219	GG
NA07000	51	AG	1	ag	0.38487739	AG
NA12802	52	AG	1	ag	0.246910994	AG
NA10855	53	GG	0.9904	gg	0.101074498	GG
NA11995	54		NA	GG	0.122831407	
NA10839	55	AG	1	ag	0.311075684	AG
NA10847	56	GG	0.9957	gg	0.249085642	GG
NA12006	57	GG	0.9871	gg	0.150386129	GG
NA12144	58	AG	1	ag	0.471045233	AG
NA11832	59	GG	1	gg	0.12820738	GG
NA12813	60	AA	0.9998	aa	0.214524819	AA
blank	61	NN	0.5	***NN***	***0***	nn
NA12891	62	AG	1	ag	0.243506463	AG
NA12717	63		NA	GG	0.166809257	
NA10861	64	AG	1	ag	0.188186593	AG
NA12004	65	AG	1	ag	0.367696504	AG
NA07048	66	GG	1	gg	0.133939378	GG
NA12801	67	AG	1	ag	0.358842926	AG
NA11830	68	AG	1	ag	0.361887308	AG
NA12057	69	AG	1	ag	0.339176262	AG
NA11994	70		NA	AA	0.344419728	
NA12740	71	AG	1	ag	0.598804578	AG
NA12005	72	AG	1	ag	0.03107029	AG
NA07345	73	AG	0.9988	ag	0.031930595	AG
NA10854	74	AG	0.9998	ag	0.195111787	AG
NA12872	75	GG	1	gg	0.461564528	GG
NA12865	76	GG	1	gg	0.459292211	GG
NA10860	77		NA	AG	0.280127408	
NA12873	78	AG	0.9987	ag	0.322321861	AG
NA12752	79	GG	1	gg	0.397679228	GG
NA10851	80	AG	1	ag	0.608788865	AG
NA07029	81	AG	0.9965	ag	0.278746845	AG
NA12248	82	GG	1	gg	0.355222594	GG
NA12155	83	AG	1	ag	0.003564763	AG
NA10838	84	AG	1	ag	0.370087275	AG
NA12812	85	GG	1	gg	0.471425964	GG
NA12248.dup	86	GG	1	gg	0.498982124	nn
NA12043	87	AA	1	aa	0.525485929	AA
NA12234	88	AA	1	aa	0.513287579	AA

	MACGT discrepancy					
	LDA discrepancy					
NA	LDA output were not g					
XY	Manual calls are given					
		**4606154**				
**Coriell**	**Sample**	**LDA Call**	**LDA quality score**	**MACGT Call**	**MACGT quality score**	**HapMap Call**
NA12892	89	AA	1	aa	0.551101754	AA
NA10846	90	GG	1	gg	0.392160978	GG
NA06993.dup	91	AG	0.9906	ag	0.562083189	nn
NA12249	92	AG	1	ag	0.085893626	AG
NA11881	93	GG	1	gg	0.122435077	GG
NA12815	94	AG	0.9998	ag	0.181788307	AG
NA12264	95	GG	1	gg	0.17384918	GG
NA18526	96	AG	1	ag	0.529708146	AG
NA18562	97	AG	1	ag	0.389981406	AG
NA18545	98	AG	1	ag	0.294162436	AG
NA18609	99	AA	0.9999	aa	0.135466434	AA
NA18566	100	AA	0.9932	aa	0.070119172	AA
NA18621	101	AG	1	ag	0.246749348	AG
NA18577	102	GG	1	***GG***	***0***	GG
NA18635	103	AA	0.9272	aa	0.06444476	AA
NA18524	104	AG	1	ag	0.464251912	AG
NA18537	105	GG	1	gg	0.02219729	GG
NA18572	106	AG	1	ag	0.1499707	AG
NA18552	107	AG	1	ag	0.065916724	AG
NA18563	108	AG	1	ag	0.048119069	AG
NA18594	109	AG	1	ag	0.248528925	nn
NA18624	110	AG	1	ag	0.423555332	AG
NA18592	111	AA	0.9837	aa	0.438374151	AA
NA18529	112	GG	1	gg	0.154443181	GG
NA18603	113	AG	1	ag	0.298557148	nn
NA18547	114	AG	1	ag	0.309551513	AG
NA18611	115	AG	1	ag	0.107549578	AG
NA18570	116	AG	1	ag	0.364358369	AG
NA18622	117	GG	1	gg	0.297637672	GG
NA18579	118	AA	1	aa	0.108988038	AA
NA18636	119	AG	1	ag	0.238545963	AG
NA18558	120	AG	0.9998	ag	0.403281496	AG
NA18540	121	AG	1	ag	0.082044621	AG
NA18609	122	AA	1	aa	0.184946074	nn
NA18555	123	AA	1	aa	0.137449539	AA
NA18612	124	AG	1	ag	0.345218133	AG
NA18573	125	AA	1	aa	0.254532898	AA
NA18632	126	AA	1	aa	0.453012461	AA
NA18593	127	GG	1	gg	0.262233495	GG
NA18532	128	GG	1	gg	0.468052968	GG
NA18605	129	AA	1	aa	0.446044049	AA
NA18550	130	AG	1	ag	0.440738458	AG
NA18603	131	AG	0.9998	ag	0.333949084	AG
NA18571	132	GG	1	gg	0.148881101	GG

	MACGT discrepancy					
	LDA discrepancy					
NA	LDA output were not g					
XY	Manual calls are given					
		**4606154**				
**Coriell**	**Sample**	**LDA Call**	**LDA quality score**	**MACGT Call**	**MACGT quality score**	**HapMap Call**
NA18623	133	AA	1	aa	0.417539052	AA
NA18582	134	AG	0.9998	ag	0.419527902	AG
NA18637	135	AG	0.9998	ag	0.009292655	AG
NA18561	136	AG	0.9988	ag	0.458279415	AG
NA18542	137	AA	1	aa	0.391016783	AA
NA18608	138	AG	1	ag	0.189491164	AG
NA18564	139	AA	1	aa	0.419896971	AA
NA18620	140	AG	1	ag	0.478356594	AG
NA18576	141	AG	1	ag	0.431099288	AG
NA18633	142	AG	1	ag	0.490705012	AG
NA18594	143	AG	0.9994	ag	0.424606035	AG
NA18942	144	AG	0.7503	ag	0.353633596	AG
NA18945	145	AG	1	ag	0.428248604	AG
NA18964	146	GG	1	gg	0.387257266	GG
NA18961	147	AG	1	ag	0.549387127	AG
NA18967	148	AG	1	ag	0.354393542	AG
NA18981	149	AG	1	ag	0.67095143	AG
NA18994	150	AA	1	aa	0.651289107	AA
NA18998	151	AG	1	ag	0.361364972	AG
NA18940	152	GG	1	gg	0.324513376	GG
NA18949	153	AA	1	aa	0.356193291	AA
NA18953	154	AG	1	ag	0.244549857	AG
NA18972	155	AG	1	ag	0.275228397	AG
NA18976	156	AG	1	ag	0.35530855	AG
NA18971	157	GG	1	gg	0.226992261	GG
blank	158	NN	0.4998	***NN***	***0***	nn
NA19000	159	AG	0.9999	ag	0.209789176	AG
NA18951	160	AA	1	aa	0.113101242	nn
NA18948	161	AG	0.9863	ag	0.062858205	AG
NA18968	162	GG	1	gg	0.610112349	GG
NA18965	163	AG	1	ag	0.39581126	AG
NA18978	164	AG	0.9999	ag	0.196048614	AG
NA18974	165	AG	0.9996	ag	0.148052159	AG
NA18992	166	GG	1	gg	0.175440233	GG
NA19005	167	GG	0.9999	gg	0.328173663	GG
NA18943	168	AG	1	ag	0.435648982	AG
NA18951	169	AA	1	aa	0.319470908	AA
NA18959	170	AG	1	ag	0.498906165	AG
NA18973	171	GG	1	gg	0.453720235	GG
NA18970	172	AG	1	ag	0.494476672	AG
NA18987	173	AG	0.9997	ag	0.241009966	AG
NA18995	174	AG	1	ag	0.513038607	AG
NA18999	175		NA	AA	0.304639406	
NA18947	176	AA	1	aa	0.15761306	AA

	MACGT discrepancy					
	LDA discrepancy					
NA	LDA output were not g					
XY	Manual calls are given					
		**4606154**				
**Coriell**	**Sample**	**LDA Call**	**LDA quality score**	**MACGT Call**	**MACGT quality score**	**HapMap Call**
NA18952	177	AA	1	aa	0.554293963	AA
NA18969	178	GG	1	gg	0.51804884	GG
NA18966	179	GG	1	gg	0.419723583	GG
NA18980	180	AA	1	aa	0.366385652	AA
NA18990	181	AA	1	aa	0.196276936	AA
NA18997	182	AG	1	ag	0.401548386	AG
NA19007	183	AG	1	ag	0.504039219	AG
NA18944	184	AG	1	ag	0.182111564	AG
NA18956	185	AG	1	ag	0.489477053	AG
NA18960	186	AG	1	ag	0.441914	AG
NA18975	187	GG	0.9417	gg	0.08109158	GG
NA18995	188	AG	1	ag	0.599541008	nn
NA18991	189	AG	1	ag	0.491780394	AG
NA18996	190	AG	0.9998	ag	0.018397052	nn
NA19003	191	AA	1	aa	0.46265961	AA
NA18502	192	AG	1	ag	0.515805766	AG
NA19153	193	GG	1	gg	0.130871125	GG
NA18857	194	GG	1	gg	0.24359679	GG
NA19223	195	GG	1	gg	0.436422541	GG
NA19201	196	GG	0.9998	gg	0.225918996	GG
NA18504	197	GG	1	gg	0.333116752	GG
NA18870	198	GG	1	gg	0.442634681	GG
NA18863	199	GG	1	gg	0.142226377	GG
NA19145	200	AA	1	aa	0.468093405	AA
NA19137	201	GG	1	gg	0.00916076	GG
NA19238	202	AG	0.9999	ag	0.200408469	nn
NA18500	203	AG	0.9746	ag	0.258893198	AG
NA19144	204	AG	0.9955	ag	0.122671393	AG
NA19203	205	AG	0.9998	ag	0.058446589	AG
NA19200	206	GG	1	gg	0.366669779	GG
NA18855	207	GG	0.9993	gg	0.164078361	GG
NA18505	208	GG	1	gg	0.144467107	GG
NA19202	209	GG	1	gg	0.142132681	GG
NA18501	210	GG	1	gg	0.026546815	GG
NA18861	211	GG	1	gg	0.062056156	GG
NA19193	212	AG	0.9999	*AG*	*0*	AG
NA19143	213	AA	1	aa	0.173244208	AA
NA18517	214	GG	1	gg	0.428283373	GG
NA18862	215	GG	1	gg	0.27145493	GG
NA18856	216	AG	1	ag	0.06778678	nn
NA19239	217	AA	1	aa	0.013887002	AA
NA19240	218	AG	0.9969	ag	0.169339875	AG
NA18856	219	AG	1	ag	0.35266001	AG
NA18503	220	GG	1	gg	0.032318978	GG

	MACGT discrepancy					
	LDA discrepancy					
NA	LDA output were not g					
XY	Manual calls are given					
		**4606154**				
**Coriell**	**Sample**	**LDA Call**	**LDA quality score**	**MACGT Call**	**MACGT quality score**	**HapMap Call**
NA18871	221	AG	1	ag	0.003122362	AG
NA19221	222	GG	1	gg	0.001275084	GG
NA19209	223	GG	0.9991	gg	0.09134987	GG
NA19152	224	AA	1	aa	0.197551644	AA
NA18515	225	AG	1	ag	0.395422587	AG
NA19238	226	AG	1	ag	0.00175302	AG
NA19154	227	AG	0.9996	ag	0.200895951	AG
NA19210	228	GG	1	gg	0.002710122	GG
NA19211	229	GG	1	gg	0.207669045	GG
NA18862	230	GG	1	gg	0.017757535	nn
NA18872	231	GG	1	gg	0.350070225	GG
NA19139	232	GG	1	***GG***	***0***	GG
NA19222	233	GG	1	gg	0.027532209	GG
NA19194	234	AG	1	ag	0.031605104	AG
NA19138	235	AG	1	ag	0.275070295	AG
NA19204	236	GG	1	gg	0.029574682	GG
NA18516	237	AG	1	ag	0.445599821	AG
NA19205	238	AG	0.9999	ag	0.235966022	AG
NA19192	239	AA	1	aa	0.263642956	AA
NA18912	240	GG	0.9999	gg	0.036162162	nn
NA18508	241	AA	1	aa	0.127339488	AA
NA19142	242	AG	1	ag	0.542168619	AG
NA18852	243	GG	1	gg	0.248146507	GG
NA18507	244	AG	1	***AG***	***0***	AG
blank	245	NN	0.5	***NN***	***0***	nn
NA19101	246	GG	1	gg	0.062355969	GG
NA19172	247	GG	0.9999	gg	0.091403985	GG
NA19160	248	AG	1	ag	0.323394419	AG
NA19129	249	GG	1	gg	0.213920109	GG
NA18913	250	GG	1	gg	0.199128877	GG
NA19120	251	AG	0.9998	ag	0.12801277	AG
NA19159	252	AA	1	aa	0.190804111	AA
NA18523	253	GG	1	gg	0.463504479	GG
NA19102	254	GG	1	gg	0.044580663	GG
NA19092	255	AA	1	aa	0.226366946	AA
NA18521	256	GG	1	gg	0.418817777	GG
NA18912	257	GG	0.9998	gg	0.01303633	GG
NA19206	258	AG	0.999	ag	0.164888661	AG
NA19128	259	AG	1	ag	0.514572946	AG
NA18859	260	AG	1	ag	0.507274721	nn
NA19094	261	NN	0.75	ag	0.044328053	AG
NA19171	262	GG	0.75	***GG***	***0***	GG
NA18860	263	GG	1	***GG***	***0***	GG
NA18858	264	GG	1	gg	0.464798734	GG

	MACGT discrepancy					
	LDA discrepancy					
NA	LDA output were not g					
XY	Manual calls are given					
		4606154				
**Coriell**	**Sample**	**LDA Call**	**LDA quality score**	**MACGT Call**	**MACGT quality score**	**HapMap Call**
NA19099	265	AG	1	ag	0.015957039	AG
NA18506	266	AA	0.8128	aa	0.038162404	AA
NA19131	267	GG	1	gg	0.290116206	GG
NA19116	268	AG	1	ag	0.3945685	AG
NA19161	269	AA	0.978	aa	0.106449841	AA
NA19140	270	AG	1	ag	0.417824856	AG
NA19207	271	AG	0.9515	ag	0.141500639	AG
NA19141	272	AA	1	aa	0.197504909	AA
NA18854	273	GG	1	gg	0.148896334	GG
NA19098	274	AG	1	ag	0.539550568	AG
NA18522	275	GG	1	gg	0.111581827	GG
NA18914	276	GG	1	gg	0.563151174	GG
NA18853	277	GG	1	gg	0.42702148	GG
NA18859	278	AG	1	ag	0.427042333	AG
NA19100	279	AG	1	ag	0.474414266	AG
NA19093	280	GG	1	gg	0.153403834	GG
NA19132	281	AG	1	ag	0.349177284	AG
NA19130	282	AG	1	ag	0.181569925	AG
NA19208	283	GG	0.999	gg	0.17738101	GG
NA19127	284	GG	1	gg	0.33387704	GG
NA19173	285	GG	1	gg	0.415860647	GG
NA19119	286	GG	1	gg	0.034159645	GG
NA19103	287	GG	1	gg	0.257095695	GG
	Discrepancies	0		0		
	NNs	2		1		
	Automated Calls	277		268		
	Manual Calls	0		9		
	Concordant Calls	257		258		258

	MACGT discrepancy					
	LDA discrepancy					
NA	LDA output were not g					
XY	Manual calls are given					
		4739199				
**Coriell**	**Sample**	**LDA Call**	**LDA quality score**	**MACGT Call**	**MACGT quality score**	**HapMap Call**
NA12753	1		NA	TT	0.310843203	
NA12875	2	CT	0.9867	ct	0.320000526	CT
NA12044	3	CC	0.9833	cc	0.292132588	CC
NA06993	4	CT	0.9664	ct	0.249845707	CT
NA12716	5	CC	0.905	cc	0.335881136	CC
NA12760	6	CC	0.9968	cc	0.163078607	CC
NA07348	7	CC	0.9996	cc	0.306473082	CC
NA12707	8	CT	0.9775	ct	0.387304301	CT
NA12156	9		NA	TT	0.324047747	
NA11992	10	CC	0.9757	cc	0.29852378	CC
NA12239	11	CT	0.9753	ct	0.313892114	CT
NA12878	12	CT	0.9572	ct	0.377081581	CT
NA11993	13	CC	0.9941	cc	0.256688057	CC
NA12750	14	CC	0.9895	cc	0.260180168	CC
NA12146	15	CT	1	***CT***	***0***	CT
NA11839	16	CT	0.9957	***CT***	***0***	CT
NA12156.dup	17	TT	0.9937	tt	0.002429578	nn
NA11829	18		NA	CT	0.298413679	
NA12154	19	CT	0.7908	ct	0.1808937	CT
NA10856	20	CT	0.9107	ct	0.188812201	CT
NA06985	21	CT	0.8733	ct	0.227675978	CT
NA11831	22	CT	0.9999	***CT***	***0***	CT
NA12003.dup	23	TT	0.9917	tt	0.034998362	nn
NA10859	24	CT	0.9998	ct	0.095615271	CT
NA07056	25	TT	0.9945	tt	0.037319751	TT
NA07022	26	TT	0.9987	tt	0.023266608	TT
NA12762	27		NA	TT	0.297773653	
NA12874	28	TT	0.989	tt	0.065842786	TT
NA12003	29	TT	0.9977	tt	0.062045699	TT
NA10835	30	TT	1	tt	0.074291994	TT
NA11882	31	CT	0.9971	ct	0.081171657	CT
NA07034	32	NN	0.5907	cc	0.002616965	CC
NA12056	33	TT	0.9952	tt	0.103762429	TT
NA12145	34	CC	0.899	cc	0.125286141	CC
NA07019	35	TT	1	tt	0.246813141	TT
NA06991	36		NA	CT	0.143547417	
NA12761	37	CC	0.9379	cc	0.17278754	CC
NA06994	38	CT	0.9998	ct	0.163288815	CT
NA12864	39	TT	1	tt	0.058837542	TT
NA07055	40		NA	CT	0.18067779	
NA10863	41	TT	0.9692	tt	0.248528442	TT
NA12763	42	TT	0.988	tt	0.230430036	TT
NA10831	43	TT	1	tt	0.140668877	TT
NA11840	44	NN	0.7499	ct	0.164916826	CT

	MACGT discrepancy					
	LDA discrepancy					
NA	LDA output were not g					
XY	Manual calls are given					
		4739199				
**Coriell**	**Sample**	**LDA Call**	**LDA quality score**	**MACGT Call**	**MACGT quality score**	**HapMap Call**
NA10830	45	CC	0.7641	cc	0.029026819	CC
NA11993.dup	46	CC	0.9033	***CC***	***0***	nn
NA12751	47	CT	0.9993	ct	0.085782228	CT
NA12814	48	CT	0.75	***CT***	***0***	CT
NA10857	49	CC	0.9984	cc	0.218343388	CC
NA07357	50	CC	0.9942	cc	0.477449931	CC
NA07000	51		NA	CT	0.3212072	
NA12802	52	CC	0.8711	cc	0.042315219	CC
NA10855	53	CT	0.9991	ct	0.358855477	CT
NA11995	54		NA	CT	0.373724993	
NA10839	55	TT	1	tt	0.198121224	TT
NA10847	56	CT	0.9961	ct	0.105218143	CT
NA12006	57	CT	0.9966	ct	0.279682308	CT
NA12144	58	CT	0.9927	ct	0.305242802	CT
NA11832	59	TT	1	tt	0.101009824	TT
NA12813	60	CT	0.9847	ct	0.345250362	CT
blank	61	CT	0.7576	ct	0.040866967	nn
NA12891	62	CC	0.879	cc	0.245351741	CC
NA12717	63		NA	CT	0.452097571	
NA10861	64	CT	0.9973	ct	0.313337335	CT
NA12004	65	CC	0.892	cc	0.0177313	CC
NA07048	66	CC	0.9133	cc	0.049520681	CC
NA12801	67	CT	0.9977	***CT***	***5.86E-04***	CT
NA11830	68	CC	0.9923	cc	0.001824009	CC
NA12057	69	CT	0.9807	ct	0.346009385	CT
NA11994	70		NA	CT	0.419740759	
NA12740	71	CT	0.9947	ct	0.480890908	CT
NA12005	72	TT	1	tt	0.002350634	TT
NA07345	73	CC	0.996	***CC***	***0***	CC
NA10854	74	CT	0.9865	ct	0.070683411	CT
NA12872	75	TT	0.7955	tt	0.096610915	TT
NA12865	76	CT	0.9983	ct	0.448416015	CT
NA10860	77		NA	CC	0.234094107	
NA12873	78	CT	0.966	ct	0.414442306	CT
NA12752	79	CC	0.9993	cc	0.014832353	CC
NA10851	80	CT	0.9746	ct	0.270404008	CT
NA07029	81	TT	0.8643	tt	0.304063448	TT
NA12248	82	CT	0.8855	ct	0.193488023	CT
NA12155	83	TT	0.7791	tt	0.085123501	TT
NA10838	84	CT	0.9533	ct	0.07929988	CT
NA12812	85	CT	0.9879	ct	0.033948719	CT
NA12248.dup	86	CT	0.9838	ct	0.107598804	nn
NA12043	87	CT	0.9015	ct	0.169326824	CT
NA12234	88	CT	0.9483	ct	0.391940897	CT

	MACGT discrepancy					
	LDA discrepancy					
NA	LDA output were not g					
XY	Manual calls are given					
		**4739199**				
**Coriell**	**Sample**	**LDA Call**	**LDA quality score**	**MACGT Call**	**MACGT quality score**	**HapMap Call**
NA12892	89	TT	0.9729	tt	0.261235119	TT
NA10846	90	CT	0.9076	ct	0.23055493	CT
NA06993.dup	91	NN	0.6701	ct	0.218928638	nn
NA12249	92	TT	0.8996	tt	0.021712888	TT
NA11881	93	CT	0.9988	ct	0.002817546	CT
NA12815	94	NN	0.7114	cc	0.06705326	CC
NA12264	95	TT	0.7775	tt	0.150997293	TT
NA18526	96	CC	0.9965	cc	0.33809211	CC
NA18562	97	CT	0.9985	ct	0.161921518	CT
NA18545	98	CC	0.8964	cc	0.442972683	CC
NA18609	99	CC	0.9019	cc	0.411332351	CC
NA18566	100	CC	0.9726	cc	0.111232276	CC
NA18621	101	TT	0.7802	*TT*	*0*	TT
NA18577	102	CC	0.9942	cc	0.374032037	CC
NA18635	103	CT	0.9586	ct	0.632749631	CT
NA18524	104	CT	0.9598	ct	0.446020659	CT
NA18537	105	CT	0.9733	ct	0.451904861	CT
NA18572	106	CC	0.9986	cc	0.337138441	CC
NA18552	107	CC	0.9829	cc	0.419540814	CC
NA18563	108	CC	0.997	cc	0.466785177	CC
NA18594	109	CT	0.8287	ct	0.367896279	nn
NA18624	110	CT	0.8569	ct	0.306020915	CT
NA18592	111	CT	0.9878	ct	0.472165862	CT
NA18529	112	CC	0.9974	cc	0.26070506	CC
NA18603	113	CC	0.9301	cc	0.083616384	nn
NA18547	114	CC	0.9319	cc	0.351379787	CC
NA18611	115	TT	0.7634	tt	0.044114659	TT
NA18570	116	CC	0.9014	cc	0.304925568	CC
NA18622	117	CC	0.9333	cc	0.002079564	CC
NA18579	118	CC	0.755	cc	0.433425585	CC
NA18636	119	CC	0.9436	cc	0.427413031	CC
NA18558	120	CC	0.8894	cc	0.189695239	CC
NA18540	121	CC	0.9903	cc	0.108630342	CC
NA18609	122	CC	0.8586	cc	0.076978429	nn
NA18555	123	NN	0.7488	*CC*	*0*	CC
NA18612	124	CC	0.9998	cc	0.182072902	CC
NA18573	125	CC	0.9921	cc	0.094499222	CC
NA18632	126	NN	0.6564	cc	0.221713897	CC
NA18593	127	TT	1	tt	0.118383143	TT
NA18532	128	NN	0.7192	cc	0.113426628	CC
NA18605	129	CC	0.9927	cc	0.145756456	CC
NA18550	130	CT	0.9704	ct	0.501589308	CT
NA18603	131	CC	0.9979	cc	0.301610066	CC
NA18571	132	CC	0.9986	cc	0.141823009	CC

	MACGT discrepancy					
	LDA discrepancy					
NA	LDA output were not g					
XY	Manual calls are given					
		**4739199**				
**Coriell**	**Sample**	**LDA Call**	**LDA quality score**	**MACGT Call**	**MACGT quality score**	**HapMap Call**
NA18623	133	NN	0.732	***CC***	***6.39E-04***	CC
NA18582	134	CC	0.9956	cc	0.265221037	CC
NA18637	135	CC	0.9769	cc	0.389407034	CC
NA18561	136	CC	0.9861	cc	0.005588095	CC
NA18542	137	CT	0.7695	ct	0.120139659	CT
NA18608	138	CT	0.8864	ct	0.462857492	CT
NA18564	139	CC	0.9695	cc	0.13016039	CC
NA18620	140	CT	0.9903	ct	0.272619133	CT
NA18576	141	CT	0.9762	ct	0.312511036	CT
NA18633	142	NN	0.6527	cc	0.005749437	CC
NA18594	143	CT	0.9889	ct	0.250633607	CT
NA18942	144	CC	0.9294	cc	0.039850697	CC
NA18945	145	CC	0.9637	***NN***	***0***	CC
NA18964	146	CC	0.9998	cc	0.391915219	CC
NA18961	147	CT	0.9712	ct	0.313767976	CT
NA18967	148	CC	0.9985	cc	0.181849697	CC
NA18981	149	CC	0.9936	cc	0.478414922	CC
NA18994	150	CC	0.9801	***CC***	***0***	CC
NA18998	151	NN	0.5575	cc	0.002663373	CC
NA18940	152	CC	0.7874	***CC***	***0***	CC
NA18949	153	CC	0.8236	cc	0.015945946	CC
NA18953	154	NN	0.7471	***CC***	***0***	CC
NA18972	155	CC	0.9149	cc	0.028015436	CC
NA18976	156	CC	0.9855	cc	0.042400673	CC
NA18971	157	CT	0.9324	ct	0.255175749	CT
blank	158	NN	0.6542	***NN***	***0***	nn
NA19000	159	CC	0.9963	cc	0.463295387	CC
NA18951	160		NA	CC	0.485606313	
NA18948	161		NA	CC	0.58686516	
NA18968	162	CT	0.9704	ct	0.112966085	CT
NA18965	163	CT	0.9719	ct	0.243098958	CT
NA18978	164	CC	0.7642	cc	0.248497642	CC
NA18974	165	CC	0.9762	cc	0.114039147	CC
NA18992	166	CC	0.9977	cc	0.028690384	CC
NA19005	167	CT	0.9954	ct	0.335753364	CT
NA18943	168	CT	0.9988	ct	0.234604746	CT
NA18951	169	CC	0.776	cc	0.023109744	CC
NA18959	170	CC	0.9778	cc	0.075038966	CC
NA18973	171	NN	0.7335	cc	0.037223357	CC
NA18970	172	CC	0.9548	cc	0.156565625	CC
NA18987	173	CC	0.9307	cc	0.073590067	CC
NA18995	174	CC	0.9235	cc	0.275077889	CC
NA18999	175	NN	0.5035	***TT***	***0***	TT
NA18947	176	CC	0.8404	***CC***	***4.80E-04***	CC

	MACGT discrepancy					
	LDA discrepancy					
NA	LDA output were not g					
XY	Manual calls are given					
		**4739199**				
**Coriell**	**Sample**	**LDA Call**	**LDA quality score**	**MACGT Call**	**MACGT quality score**	**HapMap Call**
NA18952	177	CC	0.9839	cc	0.277739248	CC
NA18969	178	CC	0.7584	*CC*	*0*	CC
NA18966	179	CC	0.9777	cc	0.116910197	CC
NA18980	180	NN	0.7201	ct	0.041297371	CT
NA18990	181	CT	0.8918	ct	0.140455684	CT
NA18997	182	CC	0.9899	*CC*	*0*	CC
NA19007	183	CT	0.978	ct	0.050829409	CT
NA18944	184	NN	0.7498	ct	0.013292647	CT
NA18956	185	CT	0.9748	ct	0.655551904	CT
NA18960	186	NN	0.7149	cc	0.22865522	CC
NA18975	187	CC	0.7596	cc	0.007494753	CC
NA18995	188	CC	0.912	cc	0.316198374	nn
NA18991	189	CC	0.9656	cc	0.311894646	CC
NA18996	190	NN	0.6421	*cc*	*9.06E-04*	nn
NA19003	191	CC	0.9634	cc	0.195208002	CC
NA18502	192	NN	0.7493	ct	0.031542094	CT
NA19153	193	CT	0.9984	ct	0.039894864	CT
NA18857	194	CT	0.7958	ct	0.27921579	CT
NA19223	195	CT	0.772	ct	0.073361506	CT
NA19201	196	CT	0.7525	ct	0.085223025	CT
NA18504	197	TT	0.9571	*TT*	*0*	TT
NA18870	198	CC	0.9815	cc	0.049937427	CC
NA18863	199	NN	0.7334	ct	0.125235763	CT
NA19145	200	CT	0.9742	ct	0.149714717	CT
NA19137	201	CT	0.9295	ct	0.160667322	CT
NA19238	202	CT	0.8726	ct	0.131197696	nn
NA18500	203	NN	0.5481	*TT*	*0*	TT
NA19144	204	NN	0.6321	*TT*	*0*	TT
NA19203	205	CT	0.824	ct	0.376037273	CT
NA19200	206	CT	0.9451	ct	0.19206212	CT
NA18855	207	TT	0.8879	tt	0.049398502	TT
NA18505	208	CC	0.9965	cc	0.008646206	CC
NA19202	209	NN	0.6425	*TT*	*0*	TT
NA18501	210	NN	0.7258	*TT*	*0*	TT
NA18861	211	TT	0.9793	*TT*	*0*	TT
NA19193	212	CT	0.7969	*CT*	*7.47E-04*	CT
NA19143	213	NN	0.6397	ct	0.016878061	CT
NA18517	214	NN	0.6981	ct	0.166067891	CT
NA18862	215	NN	0.5554	ct	0.075207243	CT
NA18856	216	CT	0.8567	ct	0.003084608	nn
NA19239	217	CT	0.7545	ct	0.288634113	CT
NA19240	218	CT	0.9678	ct	0.339303182	CT
NA18856	219	CT	0.9782	ct	0.05579347	CT
NA18503	220	CT	0.9858	ct	0.054280707	CT

	MACGT discrepancy					
	LDA discrepancy					
NA	LDA output were not g					
XY	Manual calls are given					
		**4739199**				
**Coriell**	**Sample**	**LDA Call**	**LDA quality score**	**MACGT Call**	**MACGT quality score**	**HapMap Call**
NA18871	221	CC	0.8153	cc	0.570977246	CC
NA19221	222	TT	0.9322	tt	0.00694242	TT
NA19209	223	TT	1	tt	0.193578189	TT
NA19152	224	TT	1	*TT*	*0*	TT
NA18515	225	CC	0.7588	cc	0.004451022	CC
NA19238	226	CT	0.9999	*CT*	*0*	CT
NA19154	227	CT	0.957	ct	0.213584403	CT
NA19210	228	CT	0.9964	ct	0.208478783	CT
NA19211	229	CT	0.9901	ct	0.05669102	CT
NA18862	230	CT	0.9042	ct	0.483669921	nn
NA18872	231	CC	0.9932	cc	0.048417532	CC
NA19139	232	CC	0.9883	cc	0.241359291	CC
NA19222	233	TT	0.7653	*TT*	*0*	TT
NA19194	234	CT	0.9581	ct	0.369130119	CT
NA19138	235	CT	0.946	ct	0.012628184	CT
NA19204	236	TT	1	tt	0.355279078	TT
NA18516	237	CC	0.7799	cc	0.062329596	CC
NA19205	238	CT	0.9644	ct	0.231563271	CT
NA19192	239	CT	0.9806	ct	0.09088605	CT
NA18912	240	CT	0.9367	ct	0.354429194	nn
NA18508	241	CC	0.9206	cc	0.289209256	CC
NA19142	242	CT	0.9974	ct	0.019859047	CT
NA18852	243	CT	0.8572	ct	0.439446476	CT
NA18507	244	CT	0.9891	ct	0.272357776	CT
blank	245	NN	0.5058	*NN*	*0*	nn
NA19101	246	CT	0.8834	ct	0.223490834	CT
NA19172	247	CT	0.9871	ct	0.151889691	CT
NA19160	248	NN	0.7474	cc	0.006208072	CC
NA19129	249	CT	0.8511	ct	0.192853626	CT
NA18913	250	TT	0.9826	tt	0.178983146	TT
NA19120	251	CT	0.9845	ct	0.015670476	CT
NA19159	252	CT	0.9694	ct	0.095275558	CT
NA18523	253	CC	0.9927	cc	0.178809601	CC
NA19102	254	CT	0.9986	ct	0.006025111	CT
NA19092	255	CC	0.9205	cc	0.204010989	CC
NA18521	256	CT	0.999	ct	0.172216063	CT
NA18912	257	CT	0.9987	ct	0.055735726	CT
NA19206	258	CT	0.9858	ct	0.401100032	CT
NA19128	259	TT	0.8233	tt	0.186622336	TT
NA18859	260	TT	0.9434	tt	0.1948671	nn
NA19094	261	CC	0.9528	cc	0.337464646	CC
NA19171	262	CT	0.9959	ct	0.325451309	CT
NA18860	263	CT	0.9676	ct	0.396718175	CT
NA18858	264	CC	0.9761	cc	0.344970183	CC

	MACGT discrepancy					
	LDA discrepancy					
NA	LDA output were not g					
XY	Manual calls are given					
		4739199				
**Coriell**	**Sample**	**LDA Call**	**LDA quality score**	**MACGT Call**	**MACGT quality score**	**HapMap Call**
NA19099	265	CT	0.9887	ct	0.443736323	CT
NA18506	266	CT	0.9993	ct	0.252189935	CT
NA19131	267	CT	0.9879	ct	0.452775132	CT
NA19116	268	TT	0.7508	*TT*	*0*	TT
NA19161	269	CC	0.9911	cc	0.238487473	CC
NA19140	270	NN	0.6968	ct	0.264071568	CT
NA19207	271	TT	0.9263	tt	0.335276373	TT
NA19141	272	TT	1	*TT*	*0*	TT
NA18854	273	CC	0.969	cc	0.247707455	CC
NA19098	274	CT	0.9682	ct	0.474109241	CT
NA18522	275	CT	0.9977	ct	0.118229292	CT
NA18914	276	TT	0.8819	tt	0.025682362	TT
NA18853	277	CT	0.9634	ct	0.406046871	CT
NA18859	278	TT	0.9068	tt	0.045137417	TT
NA19100	279	CT	0.8933	ct	0.19476845	CT
NA19093	280	CC	0.9836	cc	0.166431567	CC
NA19132	281	CT	0.9954	ct	0.268808374	CT
NA19130	282	TT	1	*TT*	*0*	TT
NA19208	283	TT	0.9621	tt	0.378218243	TT
NA19127	284	CT	0.8625	ct	0.046169105	CT
NA19173	285	CC	0.995	cc	0.123312225	CC
NA19119	286	CC	0.9691	cc	0.119593979	CC
NA19103	287	CT	0.9924	ct	0.241942127	CT
	Discrepancies	0		0		
	NNs	28		1		
	Automated Calls	274		240		
	Manual Calls	0		34		
	Concordant Calls	230		255		256

	MACGT discrepancy					
	LDA discrepancy					
NA	LDA output were not g					
XY	Manual calls are given					
		**4873622**				
**Coriell**	**Sample**	**LDA Call**	**LDA quality score**	**MACGT Call**	**MACGT quality score**	**HapMap Call**
NA12753	1		NA	CG	0.284862792	
NA12875	2	CC	1	cc	0.107880171	CC
NA12044	3	CC	1	cc	0.140451348	CC
NA06993	4	CC	1	cc	0.522696872	CC
NA12716	5	CC	1	cc	0.093568545	CC
NA12760	6	CC	1	cc	0.186665441	CC
NA07348	7	CC	1	cc	0.077125648	CC
NA12707	8	CG	0.9957	***CG***	***0***	CG
NA12156	9		NA	CC	0.054935133	
NA11992	10	CC	1	cc	0.316779249	CC
NA12239	11	CC	1	cc	0.173908815	CC
NA12878	12	CC	1	cc	0.315802545	CC
NA11993	13	CC	1	cc	0.203537089	CC
NA12750	14	CC	1	cc	0.232275901	CC
NA12146	15	CG	0.9931	cg	0.10972312	CG
NA11839	16	CC	1	cc	0.283671735	CC
NA12156.dup	17	CC	1	cc	0.357337286	nn
NA11829	18		NA	CC	0.27995092	
NA12154	19	CC	1	cc	0.365872481	CC
NA10856	20	CC	1	cc	0.127531761	CC
NA06985	21	CC	1	cc	0.098335654	CC
NA11831	22	CG	0.9946	cg	0.129704532	CG
NA12003.dup	23	CC	1	cc	0.25189407	nn
NA10859	24	CG	0.9655	cg	0.042667416	CG
NA07056	25	CC	1	cc	0.380254576	CC
NA07022	26	CC	1	cc	0.42326259	CC
NA12762	27		NA	GG	0.579704903	
NA12874	28	GG	1	gg	0.001728183	GG
NA12003	29	CC	1	cc	0.489894061	CC
NA10835	30	CC	1	cc	0.148858694	CC
NA11882	31	CC	1	cc	0.444572194	CC
NA07034	32	CG	0.7537	cg	0.050395156	CG
NA12056	33	CC	1	cc	0.419300807	CC
NA12145	34	CC	1	cc	0.178327547	CC
NA07019	35	CC	1	cc	0.48214309	CC
NA06991	36		NA	CC	0.520031163	
NA12761	37	CG	1	cg	0.270728402	CG
NA06994	38	CG	0.9962	cg	0.001720074	CG
NA12864	39	CC	1	cc	0.452598982	CC
NA07055	40	CC	1	cc	0.390997661	CC
NA10863	41	CC	1	cc	0.506104275	CC
NA12763	42	CC	1	cc	0.579482303	CC
NA10831	43	CC	1	cc	0.311987426	CC
NA11840	44	CC	1	cc	0.610515158	CC

	MACGT discrepancy					
	LDA discrepancy					
NA	LDA output were not g					
XY	Manual calls are given					
		4873622				
**Coriell**	**Sample**	**LDA Call**	**LDA quality score**	**MACGT Call**	**MACGT quality score**	**HapMap Call**
NA10830	45	CC	1	cc	0.45942983	CC
NA11993.dup	46	CC	1	cc	0.475430782	nn
NA12751	47	CC	1	cc	0.518446369	CC
NA12814	48	CC	1	cc	0.257032383	CC
NA10857	49	CC	1	cc	0.441174639	CC
NA07357	50	CC	1	cc	0.635887815	CC
NA07000	51	CC	1	cc	0.046686512	CC
NA12802	52	CG	1	cg	0.003948381	CG
NA10855	53	CC	1	cc	0.223237494	CC
NA11995	54		NA	CC	0.163950778	
NA10839	55	CG	0.9362	cg	0.258429855	CG
NA10847	56	CG	0.9986	cg	0.023370352	CG
NA12006	57	CC	1	cc	0.230877803	CC
NA12144	58	CG	0.9987	cg	0.343732655	CG
NA11832	59	CC	1	cc	0.386919837	CC
NA12813	60	CC	1	cc	0.362809465	CC
blank	61	CC	1	cc	0.431250506	nn
NA12891	62	CC	1	cc	0.363942291	CC
NA12717	63		NA	CG	0.46942707	
NA10861	64	CC	1	cc	0.18474256	CC
NA12004	65	CG	0.904	cg	0.128792167	CG
NA07048	66	CC	1	cc	0.525610348	CC
NA12801	67	CC	1	cc	0.014768906	CC
NA11830	68	CC	1	cc	0.189714643	CC
NA12057	69	CC	1	cc	0.364015426	CC
NA11994	70		NA	CC	0.537022938	
NA12740	71	CC	1	cc	0.397624839	CC
NA12005	72	CG	0.9926	cg	0.296423816	CG
NA07345	73	CC	1	*CC*	*0*	CC
NA10854	74	CC	1	cc	0.212578791	CC
NA12872	75	CC	1	cc	0.017914979	CC
NA12865	76	CG	0.9815	cg	0.146688365	CG
NA10860	77		NA	CC	0.408735005	
NA12873	78	CC	1	cc	0.01364792	CC
NA12752	79	CG	0.9267	cg	0.056832228	CG
NA10851	80	CC	1	cc	0.524049079	CC
NA07029	81	CG	0.7655	*CG*	*0*	CG
NA12248	82	CG	0.9998	*CG*	*0*	CG
NA12155	83	CC	1	*CC*	*0*	CC
NA10838	84	CC	1	*CC*	*0*	CC
NA12812	85	CC	1	cc	0.013890775	CC
NA12248.dup	86	NN	0.5118	*CG*	*0*	nn
NA12043	87	CC	0.9994	*CC*	*0*	CC
NA12234	88	CC	1	cc	0.13495939	CC

	MACGT discrepancy					
	LDA discrepancy					
NA	LDA output were not g					
XY	Manual calls are given					
		**4873622**				
**Coriell**	**Sample**	**LDA Call**	**LDA quality score**	**MACGT Call**	**MACGT quality score**	**HapMap Call**
NA12892	89	CC	0.7502	***CC***	***0***	CC
NA10846	90	CG	0.9998	cg	0.128902494	CG
NA06993.dup	91	CC	1	cc	0.168138937	nn
NA12249	92	CC	1	cc	0.048387665	CC
NA11881	93	CG	0.7598	***CG***	***0***	CG
NA12815	94	GG	1	gg	0.190223152	GG
NA12264	95	CC	1	cc	0.314337993	CC
NA18526	96	GG	0.9994	gg	0.585417005	GG
NA18562	97	GG	1	gg	0.384754888	GG
NA18545	98	CG	0.9868	cg	0.005594815	CG
NA18609	99	GG	0.9991	gg	0.310708789	GG
NA18566	100	CG	0.9966	cg	0.090910791	CG
NA18621	101	CG	0.984	cg	0.049833151	CG
NA18577	102	GG	0.7534	gg	0.549046298	GG
NA18635	103	GG	0.9241	gg	0.388237622	GG
NA18524	104	CG	0.876	cg	0.46348033	CG
NA18537	105	GG	1	gg	0.439362879	GG
NA18572	106	CG	0.9992	cg	0.212968604	CG
NA18552	107	GG	0.9999	gg	0.116076821	GG
NA18563	108	GG	0.8167	gg	0.001488968	GG
NA18594	109	CG	0.9875	cg	0.064054561	nn
NA18624	110	CC	1	cc	0.638713747	CC
NA18592	111	GG	1	gg	0.081797356	GG
NA18529	112	CG	0.8765	cg	0.086951088	CG
NA18603	113	GG	1	gg	0.526036975	nn
NA18547	114	CC	1	cc	0.12563459	CC
NA18611	115	CC	1	***CC***	***0***	CC
NA18570	116	GG	0.9997	gg	0.219462309	GG
NA18622	117	CG	1	cg	0.080304385	CG
NA18579	118	CG	0.9999	cg	0.003299353	CG
NA18636	119	CC	1	cc	0.126465973	CC
NA18558	120	CG	0.9994	cg	0.02471689	CG
NA18540	121	CG	0.9998	***CG***	***0***	CG
NA18609	122	GG	0.9924	gg	0.338536332	nn
NA18555	123	CC	0.9999	***CC***	***0***	CC
NA18612	124	CG	1	cg	0.162084075	CG
NA18573	125	GG	0.9999	gg	0.210210826	GG
NA18632	126	GG	1	gg	0.473536529	GG
NA18593	127	CC	1	cc	0.071049743	CC
NA18532	128	GG	1	gg	0.489836004	GG
NA18605	129	CG	0.9991	***CG***	***0***	CG
NA18550	130	CC	1	cc	0.51505747	CC
NA18603	131	GG	1	gg	0.379986198	GG
NA18571	132	CG	1	cg	0.001788722	CG

	MACGT discrepancy					
	LDA discrepancy					
NA	LDA output were not g					
XY	Manual calls are given					
		**4873622**				
**Coriell**	**Sample**	**LDA Call**	**LDA quality score**	**MACGT Call**	**MACGT quality score**	**HapMap Call**
NA18623	133	GG	1	gg	0.479975016	GG
NA18582	134	CG	0.9984	cg	0.244996705	CG
NA18637	135	CG	0.9987	cg	0.164329672	CG
NA18561	136	CG	0.8771	cg	0.13339982	CG
NA18542	137	GG	1	gg	0.024043441	GG
NA18608	138	CG	0.9998	cg	0.090405009	CG
NA18564	139	GG	1	gg	0.456154285	GG
NA18620	140	CC	1	cc	0.467315962	CC
NA18576	141	CG	0.9991	cg	0.189758178	CG
NA18633	142	GG	0.9999	gg	0.406244573	GG
NA18594	143	CG	0.9161	***CG***	***0***	CG
NA18942	144	GG	0.9186	gg	0.106911199	GG
NA18945	145	CG	0.9956	cg	0.304036449	CG
NA18964	146	CG	1	cg	0.333237505	CG
NA18961	147	CG	0.9981	cg	0.293610498	CG
NA18967	148	CC	1	cc	0.47037577	CC
NA18981	149	GG	0.9834	gg	0.516221807	GG
NA18994	150	GG	0.9991	gg	0.1054019	GG
NA18998	151	CG	0.9999	***CG***	***0***	CG
NA18940	152	GG	0.983	gg	0.453045236	GG
NA18949	153	GG	0.9998	gg	0.430175393	GG
NA18953	154		NA	GG	0.295383542	
NA18972	155	CC	1	cc	0.171022848	CC
NA18976	156	GG	1	gg	0.040756571	GG
NA18971	157	GG	0.7729	gg	0.469414979	GG
blank	158	NN	0.5	***NN***	***0***	nn
NA19000	159	CG	0.9975	cg	0.245917195	CG
NA18951	160	CG	0.9817	cg	0.151431761	nn
NA18948	161	CG	0.9989	cg	0.076155376	CG
NA18968	162	CC	1	cc	0.256507129	CC
NA18965	163	GG	0.929	gg	0.148721791	GG
NA18978	164	GG	0.874	gg	0.569280412	GG
NA18974	165	GG	0.9299	gg	0.273242805	GG
NA18992	166	CG	0.9501	cg	0.002518436	CG
NA19005	167	GG	1	gg	0.439926972	GG
NA18943	168	CG	0.9994	cg	0.323512345	CG
NA18951	169	CG	0.9999	cg	0.197719156	CG
NA18959	170	CG	0.9997	cg	0.324743153	CG
NA18973	171	GG	0.9969	gg	0.481916361	GG
NA18970	172	CG	1	cg	0.299292597	CG
NA18987	173	CC	1	cc	0.0740145	CC
NA18995	174	GG	0.9968	gg	0.516390322	GG
NA18999	175	CC	1	cc	0.613637517	CC
NA18947	176	CG	0.9991	cg	0.176759994	CG

	MACGT discrepancy					
	LDA discrepancy					
NA	LDA output were not g					
XY	Manual calls are given					
		**4873622**				
**Coriell**	**Sample**	**LDA Call**	**LDA quality score**	**MACGT Call**	**MACGT quality score**	**HapMap Call**
NA18952	177	CC	1	cc	0.206767462	CC
NA18969	178	GG	0.981	gg	0.081601277	GG
NA18966	179	GG	0.9998	gg	0.451746091	GG
NA18980	180	GG	1	gg	0.342819202	GG
NA18990	181	CG	0.9996	cg	0.101151997	CG
NA18997	182	GG	1	gg	0.065440664	GG
NA19007	183	GG	1	gg	0.378273028	GG
NA18944	184	GG	1	gg	0.114534441	GG
NA18956	185	CC	1	cc	0.536705215	CC
NA18960	186	CC	1	cc	0.603305157	CC
NA18975	187	GG	1	gg	0.395402093	GG
NA18995	188	GG	1	gg	0.40653475	nn
NA18991	189	CG	1	cg	0.20095885	CG
NA18996	190	GG	1	gg	0.431099438	nn
NA19003	191	GG	0.9999	gg	0.550988491	GG
NA18502	192	CG	0.9864	cg	0.120625328	CG
NA19153	193	GG	0.9999	gg	0.380489329	GG
NA18857	194	CG	1	cg	0.237361601	CG
NA19223	195	CG	0.9972	cg	0.067395874	CG
NA19201	196	CG	0.9995	cg	0.096059228	CG
NA18504	197	CG	1	cg	0.148561241	CG
NA18870	198	GG	0.8967	gg	0.51654097	GG
NA18863	199	GG	0.9963	gg	0.260258966	GG
NA19145	200	CC	1	cc	0.170894943	CC
NA19137	201	CC	1	cc	0.174671915	CC
NA19238	202	CG	0.9994	cg	0.020193847	nn
NA18500	203	CG	0.9999	***CG***	***0***	CG
NA19144	204	CC	1	***CC***	***0***	CC
NA19203	205	GG	0.7524	gg	0.606826394	GG
NA19200	206	CG	1	cg	0.025417753	CG
NA18855	207	CG	0.9999	cg	0.084110355	CG
NA18505	208	CG	0.9565	cg	0.007828191	CG
NA19202	209	CG	0.9999	cg	0.211980769	CG
NA18501	210	CC	1	cc	0.232255995	CC
NA18861	211	GG	0.8462	gg	0.026165465	GG
NA19193	212	CG	1	cg	0.013691855	CG
NA19143	213	CC	1	cc	0.056830157	CC
NA18517	214	GG	0.979	gg	0.397623618	GG
NA18862	215	CG	1	cg	0.031948918	CG
NA18856	216	CG	0.9426	***cg***	***9.75E-04***	nn
NA19239	217	CC	1	***CC***	***0***	CC
NA19240	218	CG	1	cg	0.05217758	CG
NA18856	219	CG	0.9552	cg	0.05774761	CG
NA18503	220	GG	0.7933	gg	0.138723029	GG

	MACGT discrepancy					
	LDA discrepancy					
NA	LDA output were not g					
XY	Manual calls are given					
		**4873622**				
**Coriell**	**Sample**	**LDA Call**	**LDA quality score**	**MACGT Call**	**MACGT quality score**	**HapMap Call**
NA18871	221	GG	0.8377	gg	0.559002237	GG
NA19221	222	CG	0.9996	cg	0.44267988	CG
NA19209	223	CG	0.9991	cg	0.358876177	CG
NA19152	224	CC	1	cc	0.13816197	CC
NA18515	225	GG	1	gg	0.402146651	GG
NA19238	226	CG	0.9994	cg	0.133083899	CG
NA19154	227	CG	1	cg	0.41205904	CG
NA19210	228	GG	0.9976	gg	0.197508773	GG
NA19211	229	GG	1	gg	0.184067972	GG
NA18862	230	CG	1	***cg***	***9.35E-04***	nn
NA18872	231	GG	0.9973	gg	0.480904607	GG
NA19139	232	CG	1	cg	0.146245141	CG
NA19222	233	CG	0.991	cg	0.040932461	CG
NA19194	234	GG	0.9818	gg	0.408896344	GG
NA19138	235	GG	1	gg	0.378668163	GG
NA19204	236	CC	1	cc	0.00260015	CC
NA18516	237	GG	1	gg	0.175370725	GG
NA19205	238	CG	0.9964	cg	0.090172592	CG
NA19192	239	CG	0.7595	cg	0.04402015	CG
NA18912	240	GG	0.9384	gg	0.005874458	nn
NA18508	241	CG	1	cg	0.13533225	CG
NA19142	242	CG	0.882	cg	0.006428055	CG
NA18852	243	GG	0.9946	gg	0.569720437	GG
NA18507	244	GG	0.8626	gg	0.341730363	GG
blank	245	NN	0.5204	***NN***	***0***	nn
NA19101	246	CG	0.9974	cg	0.171992388	CG
NA19172	247	CG	0.9992	***CG***	***0***	CG
NA19160	248	GG	1	gg	0.212341339	GG
NA19129	249	GG	0.9904	gg	0.21703822	GG
NA18913	250	CC	1	cc	0.275791295	CC
NA19120	251	CG	0.8593	***CG***	***0***	CG
NA19159	252	GG	0.9999	gg	0.363393187	GG
NA18523	253	CG	1	cg	0.325358378	CG
NA19102	254	GG	1	gg	0.218600761	GG
NA19092	255	GG	0.9732	gg	0.27583066	GG
NA18521	256	CG	0.9962	cg	0.156753617	CG
NA18912	257	GG	1	gg	0.045731673	GG
NA19206	258	CC	1	cc	0.531128053	CC
NA19128	259	CG	0.9854	cg	0.141582119	CG
NA18859	260	CG	0.9974	cg	0.202862825	nn
NA19094	261	NN	0.7304	cg	0.472343822	CG
NA19171	262	NN	0.7259	***CG***	***0***	CG
NA18860	263	CC	1	cc	0.038131892	CC
NA18858	264	CC	1	cc	0.411307501	CC

	MACGT discrepancy					
	LDA discrepancy					
NA	LDA output were not g					
XY	Manual calls are given					
		4873622				
**Coriell**	**Sample**	**LDA Call**	**LDA quality score**	**MACGT Call**	**MACGT quality score**	**HapMap Call**
NA19099	265	CG	0.9375	cg	0.16435836	CG
NA18506	266	GG	1	gg	0.291242402	GG
NA19131	267	CG	0.9941	cg	0.016261984	CG
NA19116	268	CG	0.7819	cg	0.027762759	CG
NA19161	269	GG	1	gg	0.274391738	GG
NA19140	270	CG	0.8575	cg	0.13628006	CG
NA19207	271	CC	1	cc	0.289834387	CC
NA19141	272	CG	0.891	cg	0.164989593	CG
NA18854	273	GG	1	gg	0.366246446	GG
NA19098	274	GG	1	gg	0.083802142	GG
NA18522	275	CC	1	cc	0.036340045	CC
NA18914	276	CG	0.9904	cg	0.040141705	CG
NA18853	277	GG	0.8451	gg	0.167732364	GG
NA18859	278	CG	0.9558	cg	0.01328066	CG
NA19100	279	GG	1	*GG*	*0*	GG
NA19093	280	CC	1	cc	0.12112079	CC
NA19132	281	CG	0.9598	cg	0.006227761	CG
NA19130	282	CC	1	cc	0.013637738	CC
NA19208	283	CC	1	cc	0.030732611	CC
NA19127	284	CG	0.9864	cg	0.025536244	CG
NA19173	285	GG	0.9998	gg	0.288610257	GG
NA19119	286	CC	1	cc	0.088758785	CC
NA19103	287	GG	1	gg	0.094892126	GG
	Discrepancies	0		0		
	NNs	3		0		
	Automated Calls	277		250		
	Manual Calls	0		27		
	Concordant Calls	256		258		258

	MACGT discrepancy					
	LDA discrepancy					
NA	LDA output were not g					
XY	Manual calls are given					
		4971653				
**Coriell**	**Sample**	**LDA Call**	**LDA quality score**	**MACGT Call**	**MACGT quality score**	**HapMap Call**
NA12753	1		NA	GG	0.387124181	
NA12875	2	GG	1	gg	0.338238293	GG
NA12044	3	GG	1	gg	0.360438764	GG
NA06993	4	GG	0.9999	gg	0.145092246	GG
NA12716	5	GG	0.9999	gg	0.176863322	GG
NA12760	6	AG	0.9994	ag	0.212538474	AG
NA07348	7	GG	1	gg	0.195897023	GG
NA12707	8	GG	1	***GG***	***0***	GG
NA12156	9		NA	GG	0.137081369	
NA11992	10	GG	1	gg	0.035273076	GG
NA12239	11	GG	1	gg	0.014360817	GG
NA12878	12	GG	0.9979	gg	0.005654183	GG
NA11993	13	GG	1	gg	0.001382317	GG
NA12750	14	GG	1	gg	0.161228777	GG
NA12146	15	GG	0.9994	gg	0.104190538	GG
NA11839	16	GG	1	gg	0.094730779	GG
NA12156.dup	17	GG	1	gg	0.096243281	nn
NA11829	18		NA	GG	0.181626971	
NA12154	19	GG	1	gg	0.21826178	GG
NA10856	20	GG	1	gg	0.198435569	GG
NA06985	21	AG	1	ag	0.307231116	AG
NA11831	22	GG	0.7512	gg	0.004496317	GG
NA12003.dup	23	GG	0.9266	gg	0.004585404	nn
NA10859	24	AG	1	ag	0.214427741	AG
NA07056	25	GG	1	gg	0.191262941	GG
NA07022	26	AG	1	ag	0.378382149	AG
NA12762	27		NA	AG	0.280639561	
NA12874	28	GG	1	***GG***	***0***	GG
NA12003	29	GG	1	gg	0.183287419	GG
NA10835	30	GG	1	gg	0.187356887	GG
NA11882	31	AG	1	ag	0.247847906	AG
NA07034	32	AG	0.9997	ag	0.222863229	AG
NA12056	33	GG	1	gg	0.215646981	GG
NA12145	34	GG	0.9734	gg	0.032061635	GG
NA07019	35	GG	1	gg	0.305636788	GG
NA06991	36		NA	GG	0.252356785	
NA12761	37	GG	1	gg	0.445853007	GG
NA06994	38	GG	0.9997	gg	0.207775102	GG
NA12864	39	AG	0.9999	ag	0.575529791	AG
NA07055	40	GG	1	gg	0.388604507	GG
NA10863	41	GG	1	gg	0.314285836	GG
NA12763	42	GG	0.9997	gg	0.223038342	GG
NA10831	43	GG	1	gg	0.211815469	GG
NA11840	44	AG	1	ag	0.596264737	AG

	MACGT discrepancy					
	LDA discrepancy					
NA	LDA output were not g					
XY	Manual calls are given					
		**4971653**				
**Coriell**	**Sample**	**LDA Call**	**LDA quality score**	**MACGT Call**	**MACGT quality score**	**HapMap Call**
NA10830	45	GG	1	gg	0.359150378	GG
NA11993.dup	46	GG	1	gg	0.5361745	nn
NA12751	47	GG	1	gg	0.408779122	GG
NA12814	48	GG	1	gg	0.212196715	GG
NA10857	49	GG	1	gg	0.457807794	GG
NA07357	50	AG	0.9995	ag	0.507849819	AG
NA07000	51	AG	0.9997	ag	0.233853003	AG
NA12802	52	GG	0.9997	gg	0.112446333	GG
NA10855	53	GG	0.8657	gg	0.04305409	GG
NA11995	54		NA	GG	0.086615056	
NA10839	55	AG	0.9997	ag	0.248766644	AG
NA10847	56	GG	0.9999	gg	0.2511941	GG
NA12006	57	GG	1	gg	0.261636801	GG
NA12144	58	GG	1	gg	0.459806419	GG
NA11832	59	GG	0.9999	gg	0.499817847	GG
NA12813	60	GG	1	gg	0.485340672	GG
blank	61	NN	0.5	***NN***	***0***	nn
NA12891	62	GG	1	gg	0.652351354	GG
NA12717	63		NA	GG	0.628901119	
NA10861	64	GG	1	gg	0.227109027	GG
NA12004	65	AG	0.9978	ag	0.123211771	AG
NA07048	66	AG	0.9995	ag	0.146991113	AG
NA12801	67	GG	1	gg	0.508381076	GG
NA11830	68	GG	1	gg	0.340884401	GG
NA12057	69	AA	1	aa	0.157418149	AA
NA11994	70		NA	GG	0.423089492	
NA12740	71	GG	1	gg	0.492996204	GG
NA12005	72	AA	0.8251	***AA***	***0***	AA
NA07345	73	GG	0.9941	***GG***	***0***	GG
NA10854	74	NN	0.7335	ag	0.001967957	AG
NA12872	75	GG	1	gg	0.002486812	GG
NA12865	76	GG	1	gg	0.088893423	GG
NA10860	77		NA	GG	0.266555051	
NA12873	78	NN	0.5622	ag	0.007789495	AG
NA12752	79	NN	0.7493	***AG***	***0***	AG
NA10851	80	AG	0.9999	ag	0.026594013	AG
NA07029	81	NN	0.7494	***AG***	***0***	AG
NA12248	82	NN	0.7299	***AG***	***0***	AG
NA12155	83	GG	1	***GG***	***0***	GG
NA10838	84	GG	1	***GG***	***0***	GG
NA12812	85	NN	0.517	***AG***	***0***	AG
NA12248.dup	86	GG	0.7516	***NN***	***0***	nn
NA12043	87	GG	1	***GG***	***0***	GG
NA12234	88	AG	0.9984	ag	0.228433701	AG

	MACGT discrepancy					
	LDA discrepancy					
NA	LDA output were not g					
XY	Manual calls are given					
		**4971653**				
**Coriell**	**Sample**	**LDA Call**	**LDA quality score**	**MACGT Call**	**MACGT quality score**	**HapMap Call**
NA12892	89	GG	0.9999	***GG***	***0***	GG
NA10846	90	GG	1	***GG***	***0***	GG
NA06993.dup	91	GG	1	gg	0.222809387	nn
NA12249	92	GG	1	***GG***	***0***	GG
NA11881	93	NN	0.7479	***AG***	***0***	AG
NA12815	94	GG	1	gg	0.002991826	GG
NA12264	95	GG	1	gg	0.064798241	GG
NA18526	96	AG	0.9999	ag	0.493149623	AG
NA18562	97	GG	1	gg	0.675188836	GG
NA18545	98	GG	1	gg	0.175264254	GG
NA18609	99	AG	0.9999	ag	0.182950158	AG
NA18566	100	GG	1	***GG***	***0***	GG
NA18621	101	AG	0.9985	ag	0.241507912	AG
NA18577	102	GG	1	gg	0.106178117	GG
NA18635	103	AG	0.9987	ag	0.041008255	AG
NA18524	104	AG	1	ag	0.380333252	AG
NA18537	105	AA	0.9993	aa	0.017919807	AA
NA18572	106	AA	0.9986	aa	0.061884218	AA
NA18552	107	GG	1	gg	0.143003331	GG
NA18563	108	AG	0.9974	ag	0.219093727	AG
NA18594	109	AG	0.9992	ag	0.25507591	nn
NA18624	110	AG	0.9857	ag	0.22750977	AG
NA18592	111	GG	1	gg	0.014130967	GG
NA18529	112	GG	1	gg	0.498460116	GG
NA18603	113	AG	0.9994	ag	0.1243687	nn
NA18547	114	AA	1	aa	0.113615496	AA
NA18611	115	GG	1	gg	0.157695382	GG
NA18570	116	AG	0.9573	ag	0.04888424	AG
NA18622	117	AG	0.9855	ag	0.19179682	AG
NA18579	118	AG	0.7955	ag	0.064625279	AG
NA18636	119	AG	0.9995	ag	0.039602401	AG
NA18558	120	NN	0.6126	***AG***	***7.47E-04***	AG
NA18540	121	GG	1	gg	0.010403434	GG
NA18609	122	NN	0.7266	***AG***	***0***	nn
NA18555	123	GG	1	***GG***	***0***	GG
NA18612	124	AG	0.8884	ag	0.145865993	AG
NA18573	125	NN	0.5001	***AG***	***0***	AG
NA18632	126	GG	1	gg	0.022659692	GG
NA18593	127	AA	1	***AA***	***0***	AA
NA18532	128	GG	1	gg	0.095856275	GG
NA18605	129	NN	0.7347	***AG***	***0***	AG
NA18550	130	NN	0.6325	***AG***	***0***	AG
NA18603	131	AG	0.8828	ag	0.075668141	AG
NA18571	132	NN	0.713	ag	0.004872048	AG

	MACGT discrepancy					
	LDA discrepancy					
NA	LDA output were not g					
XY	Manual calls are given					
				**4971653**		
**Coriell**	**Sample**	**LDA Call**	**LDA quality score**	**MACGT Call**	**MACGT quality score**	**HapMap Call**
NA18623	133	AA	1	***AA***	***0***	AA
NA18582	134	GG	1	gg	0.506794874	GG
NA18637	135	AG	0.8373	ag	0.133760315	AG
NA18561	136	NN	0.7485	***AG***	***0***	AG
NA18542	137	GG	1	gg	0.002597898	GG
NA18608	138	AG	0.8581	ag	0.155267571	AG
NA18564	139	NN	0.7418	***AG***	***0***	AG
NA18620	140	NN	0.7408	***AG***	***0***	AG
NA18576	141	GG	1	gg	0.021529098	GG
NA18633	142	GG	1	***GG***	***0***	GG
NA18594	143	NN	0.671	***AG***	***0***	AG
NA18942	144	AA	1	aa	0.279315484	AA
NA18945	145	GG	1	gg	0.610150523	GG
NA18964	146	GG	1	***GG***	***0***	GG
NA18961	147	AA	1	aa	0.019235721	AA
NA18967	148	AA	1	aa	0.130469646	AA
NA18981	149	GG	1	gg	0.376343342	GG
NA18994	150	GG	1	gg	0.004315516	GG
NA18998	151	NN	0.7443	***AG***	***7.54E-04***	AG
NA18940	152	AG	0.8341	ag	0.003535486	AG
NA18949	153	GG	1	gg	0.142176673	GG
NA18953	154	NN	0.7445	***AG***	***0***	AG
NA18972	155	NN	0.7441	***AG***	***0***	AG
NA18976	156	AG	0.8932	ag	0.143710571	AG
NA18971	157	NN	0.6713	ag	0.001053303	AG
blank	158	NN	0.5113	***NN***	***0***	nn
NA19000	159	AG	0.9915	ag	0.191300436	AG
NA18951	160	AG	0.8662	ag	0.214812454	nn
NA18948	161	GG	1	gg	0.213858331	GG
NA18968	162	GG	1	gg	0.39995997	GG
NA18965	163	AG	0.917	ag	0.170769021	AG
NA18978	164	AG	0.9372	ag	0.095461064	AG
NA18974	165		NA	AA	0.381580625	
NA18992	166	AG	0.7535	ag	0.028544693	AG
NA19005	167	AG	0.9848	ag	0.29821486	AG
NA18943	168	AG	0.9968	ag	0.298023369	AG
NA18951	169	AG	0.9683	ag	0.145193352	AG
NA18959	170	AG	0.9984	ag	0.237710794	AG
NA18973	171		NA	AA	0.33798464	
NA18970	172	AG	0.9578	ag	0.217422235	AG
NA18987	173	AG	0.7929	ag	0.077388805	AG
NA18995	174	AG	0.9869	ag	0.202824027	AG
NA18999	175		NA	AA	0.355441803	
NA18947	176	AG	0.7548	ag	0.0912561	AG

	MACGT discrepancy					
	LDA discrepancy					
NA	LDA output were not g					
XY	Manual calls are given					
		**4971653**				
**Coriell**	**Sample**	**LDA Call**	**LDA quality score**	**MACGT Call**	**MACGT quality score**	**HapMap Call**
NA18952	177	AG	0.9088	ag	0.175002881	AG
NA18969	178	AG	0.7952	ag	0.015617055	AG
NA18966	179	AG	0.954	ag	0.039603367	AG
NA18980	180	NN	0.7466	ag	0.025168627	AG
NA18990	181	AG	0.7851	ag	0.024307193	AG
NA18997	182	GG	1	gg	0.484846907	GG
NA19007	183	GG	1	gg	0.012213339	GG
NA18944	184	GG	1	gg	0.005024508	GG
NA18956	185	AG	0.9947	ag	0.176506663	AG
NA18960	186	AG	0.9629	ag	0.132183155	AG
NA18975	187	AG	0.998	ag	0.057848407	AG
NA18995	188	AG	0.9607	ag	0.141176432	nn
NA18991	189		NA	AA	0.317463866	
NA18996	190		NA	AA	0.496216568	
NA19003	191	AA	1	aa	0.41355873	AA
NA18502	192	GG	1	gg	0.153940084	GG
NA19153	193	GG	0.9996	gg	0.221564388	GG
NA18857	194	GG	1	gg	0.402893211	GG
NA19223	195	GG	1	gg	0.316810258	GG
NA19201	196	AG	0.9715	ag	0.106306012	AG
NA18504	197	GG	1	gg	0.053662676	GG
NA18870	198	GG	1	gg	0.304360926	GG
NA18863	199	GG	1	gg	0.206049636	GG
NA19145	200	GG	1	gg	0.104186282	GG
NA19137	201	GG	1	gg	0.055996394	GG
NA19238	202	GG	1	gg	0.279214511	nn
NA18500	203	GG	1	gg	0.009295163	GG
NA19144	204	AG	0.8389	***AG***	***0***	AG
NA19203	205	AG	0.9676	ag	0.037426958	AG
NA19200	206	AG	0.9512	ag	0.025255286	AG
NA18855	207	GG	1	gg	0.500742401	GG
NA18505	208	GG	1	gg	0.193774397	GG
NA19202	209	AG	0.7602	ag	0.062479107	AG
NA18501	210	GG	1	gg	0.189851499	GG
NA18861	211	GG	1	***GG***	***5.56E-04***	GG
NA19193	212	GG	1	***GG***	***5.72E-04***	GG
NA19143	213	GG	1	gg	0.359772063	GG
NA18517	214	GG	1	gg	0.023815601	GG
NA18862	215	GG	1	gg	0.22635348	GG
NA18856	216	GG	1	gg	0.078841189	nn
NA19239	217	GG	1	gg	0.096192711	GG
NA19240	218	GG	0.9997	gg	0.168809428	GG
NA18856	219	GG	1	gg	0.284736891	GG
NA18503	220	GG	0.9988	gg	0.132876914	GG

	MACGT discrepancy					
	LDA discrepancy					
NA	LDA output were not g					
XY	Manual calls are given					
		**4971653**				
**Coriell**	**Sample**	**LDA Call**	**LDA quality score**	**MACGT Call**	**MACGT quality score**	**HapMap Call**
NA18871	221	AG	0.9996	ag	0.291488433	AG
NA19221	222	GG	0.9999	gg	0.326214362	GG
NA19209	223	AG	1	ag	0.445451479	AG
NA19152	224	GG	1	gg	0.001090536	GG
NA18515	225	AG	0.9998	ag	0.432148943	AG
NA19238	226	GG	1	gg	0.011944225	GG
NA19154	227	GG	1	gg	0.453557153	GG
NA19210	228	GG	1	gg	0.217745228	GG
NA19211	229	AG	0.9707	ag	0.162535868	AG
NA18862	230	GG	1	gg	0.001056869	nn
NA18872	231	GG	1	gg	0.54683882	GG
NA19139	232	GG	1	gg	0.352360037	GG
NA19222	233	GG	1	gg	0.377542295	GG
NA19194	234	GG	1	gg	0.540871564	GG
NA19138	235	GG	1	gg	0.06079508	GG
NA19204	236	GG	1	gg	0.081172182	GG
NA18516	237	AG	1	ag	0.350665618	AG
NA19205	238	GG	0.9995	***GG***	***5.18E-04***	GG
NA19192	239	GG	1	gg	0.227042784	GG
NA18912	240	GG	0.9999	gg	0.014785145	nn
NA18508	241	GG	0.9963	gg	0.005053742	GG
NA19142	242	GG	0.9947	gg	0.01861266	GG
NA18852	243	GG	1	gg	0.428528893	GG
NA18507	244	GG	1	gg	0.046332771	GG
blank	245	NN	0.5	***NN***	***0***	nn
NA19101	246	GG	1	gg	0.252663859	GG
NA19172	247	AG	0.9877	ag	0.116173676	AG
NA19160	248	GG	1	gg	0.114248283	GG
NA19129	249	GG	1	gg	0.121631407	GG
NA18913	250	GG	1	gg	0.069227142	GG
NA19120	251	GG	1	gg	0.107644732	GG
NA19159	252	AG	0.8861	ag	0.05192159	AG
NA18523	253	GG	1	gg	0.409386014	GG
NA19102	254	GG	1	gg	0.116610908	GG
NA19092	255	GG	1	gg	0.113276062	GG
NA18521	256	GG	0.9999	gg	0.12719466	GG
NA18912	257	GG	1	gg	0.283101693	GG
NA19206	258	GG	1	gg	0.310098521	GG
NA19128	259	GG	1	gg	0.249026116	GG
NA18859	260	GG	1	gg	0.432404063	nn
NA19094	261	GG	1	gg	0.539106441	GG
NA19171	262	GG	1	gg	0.478345367	GG
NA18860	263	GG	1	gg	0.145111116	GG
NA18858	264	GG	1	gg	0.17846164	GG

	MACGT discrepancy					
	LDA discrepancy					
NA	LDA output were not g					
XY	Manual calls are given					
		4971653				
**Coriell**	**Sample**	**LDA Call**	**LDA quality score**	**MACGT Call**	**MACGT quality score**	**HapMap Call**
NA19099	265	GG	1	gg	0.316734674	GG
NA18506	266	GG	1	***GG***	***0***	GG
NA19131	267	GG	1	gg	0.174147536	GG
NA19116	268	GG	1	gg	0.397797711	GG
NA19161	269	GG	1	gg	0.058157314	GG
NA19140	270	AG	0.9999	ag	0.28075656	AG
NA19207	271	GG	1	gg	0.162388512	GG
NA19141	272	GG	1	gg	0.332830333	GG
NA18854	273	GG	1	gg	0.224751058	GG
NA19098	274	GG	1	gg	0.4390413	GG
NA18522	275	GG	1	gg	0.001081639	GG
NA18914	276	GG	1	gg	0.140080934	GG
NA18853	277	GG	1	***GG***	***6.59E-04***	GG
NA18859	278	GG	1	gg	0.001684771	GG
NA19100	279	GG	1	gg	0.177310318	GG
NA19093	280	GG	1	gg	0.131238392	GG
NA19132	281	GG	1	gg	0.154433886	GG
NA19130	282	GG	1	gg	0.133220068	GG
NA19208	283	GG	1	gg	0.222357884	GG
NA19127	284	GG	1	gg	0.329010635	GG
NA19173	285	GG	1	gg	0.284382825	GG
NA19119	286	GG	1	gg	0.421734375	GG
NA19103	287	GG	1	gg	0.101841945	GG
	Discrepancies	0		0		
	NNs	23		2		
	Automated Calls	273		230		
	Manual Calls	0		43		
	Concordant Calls	234		255		255

	MACGT discrepancy					
	LDA discrepancy					
NA	LDA output were not g					
XY	Manual calls are given					
		592069				
**Coriell**	**Sample**	**LDA Call**	**LDA quality score**	**MACGT Call**	**MACGT quality score**	**HapMap Call**
NA12753	1		NA	GA	0.474963102	
NA12875	2	GA	1	ga	0.377488123	GA
NA12044	3	AA	0.9991	aa	0.275729434	AA
NA06993	4	GA	0.9999	ga	0.574746257	GA
NA12716	5	GG	0.9754	gg	0.059205574	GG
NA12760	6	GA	0.9999	ga	0.467070977	GA
NA07348	7	GA	1	ga	0.353777693	GA
NA12707	8	GG	0.9999	gg	0.348877451	GG
NA12156	9		NA	GG	0.509971069	
NA11992	10	AA	1	aa	0.103063009	AA
NA12239	11	AA	1	aa	0.230629757	AA
NA12878	12	AA	1	aa	0.121896742	AA
NA11993	13	GA	1	ga	0.033396987	GA
NA12750	14	GA	1	ga	0.03101216	GA
NA12146	15	GA	1	ga	0.019718139	GA
NA11839	16	AA	0.9999	aa	0.031460198	AA
NA12156.dup	17	GG	1	gg	0.264124403	nn
NA11829	18		NA	GG	0.285947157	
NA12154	19	AA	1	aa	0.118845431	AA
NA10856	20	GG	1	gg	0.292220421	GG
NA06985	21	GG	0.9998	gg	0.08730754	GG
NA11831	22	GA	1	ga	0.010257582	GA
NA12003.dup	23	AA	1	aa	0.060239426	nn
NA10859	24	GA	1	ga	0.028751673	GA
NA07056	25	GG	0.9986	gg	0.207234608	GG
NA07022	26	GA	1	ga	0.106235493	GA
NA12762	27		NA	AA	0.409654581	
NA12874	28	AA	1	aa	0.146896955	AA
NA12003	29	AA	1	aa	0.201861591	AA
NA10835	30	AA	0.9999	aa	0.153821475	AA
NA11882	31	GA	1	ga	0.12103605	GA
NA07034	32	GA	1	ga	0.061844065	GA
NA12056	33	GG	1	gg	0.3476562	GG
NA12145	34	AA	1	aa	0.029269485	AA
NA07019	35	GA	1	ga	0.354634595	GA
NA06991	36		NA	GA	0.167017831	
NA12761	37	AA	1	aa	0.176371319	AA
NA06994	38	GA	1	ga	0.124892673	GA
NA12864	39	AA	1	aa	0.120544722	AA
NA07055	40	GA	1	ga	0.502658637	GA
NA10863	41	GA	1	ga	0.145338974	GA
NA12763	42	GA	1	ga	0.185836497	GA
NA10831	43	GG	0.9999	gg	0.135148585	GG
NA11840	44	GG	1	gg	0.055265699	GG

	MACGT discrepancy					
	LDA discrepancy					
NA	LDA output were not g					
XY	Manual calls are given					
		**592069**				
**Coriell**	**Sample**	**LDA Call**	**LDA quality score**	**MACGT Call**	**MACGT quality score**	**HapMap Call**
NA10830	45	AA	1	aa	0.17569304	AA
NA11993.dup	46	GA	1	ga	0.263719393	nn
NA12751	47	AA	1	aa	0.390346119	AA
NA12814	48	GA	1	ga	0.125906413	GA
NA10857	49	GA	1	ga	0.36770303	GA
NA07357	50	GA	1	ga	0.330077811	GA
NA07000	51	GA	1	ga	0.028314767	GA
NA12802	52	AA	1	aa	0.332127693	AA
NA10855	53	AA	1	aa	0.024699551	AA
NA11995	54		NA	GA	0.127800718	
NA10839	55	GG	0.771	***GG***	***0***	GG
NA10847	56		NA	GA	0.193075387	
NA12006	57	GG	0.999	gg	0.002061332	GG
NA12144	58	GG	1	***GG***	***0***	GG
NA11832	59	AA	1	***AA***	***0***	AA
NA12813	60	GA	1	ga	0.085538591	GA
blank	61	AA	0.9999	aa	0.237431066	nn
NA12891	62	AA	1	aa	0.018004203	AA
NA12717	63		NA	GG	0.363417379	
NA10861	64	GA	1	ga	0.180106111	GA
NA12004	65	GA	1	ga	0.046797517	GA
NA07048	66	GA	1	ga	0.252680684	GA
NA12801	67	GA	1	ga	0.029020365	GA
NA11830	68	GA	1	ga	0.210808985	GA
NA12057	69	AA	1	***AA***	***5.03E-04***	AA
NA11994	70		NA	AA	0.276491529	
NA12740	71	AA	1	aa	0.083179697	AA
NA12005	72	GA	1	ga	0.395558268	GA
NA07345	73	AA	0.9997	aa	0.030674704	AA
NA10854	74	GA	1	ga	0.268805303	GA
NA12872	75	AA	1	aa	0.358363817	AA
NA12865	76	AA	1	aa	0.193746196	AA
NA10860	77		NA	GA	0.196050024	
NA12873	78	GA	1	ga	0.270157213	GA
NA12752	79	GA	1	ga	0.282085368	GA
NA10851	80	GA	1	ga	0.490877554	GA
NA07029	81	GG	1	gg	0.228084436	GG
NA12248	82	GA	1	ga	0.125997482	GA
NA12155	83	GG	1	gg	0.01190324	GG
NA10838	84	AA	0.9986	aa	0.478046924	AA
NA12812	85	AA	1	aa	0.424934916	AA
NA12248.dup	86	GA	1	ga	0.256131673	nn
NA12043	87	GG	1	gg	0.087723864	GG
NA12234	88	AA	1	***AA***	***0***	AA

	MACGT discrepancy					
	LDA discrepancy					
NA	LDA output were not g					
XY	Manual calls are given					
		592069				
**Coriell**	**Sample**	**LDA Call**	**LDA quality score**	**MACGT Call**	**MACGT quality score**	**HapMap Call**
NA12892	89	GA	1	ga	0.320905396	GA
NA10846	90	GA	1	ga	0.303732017	GA
NA06993.dup	91	GA	1	ga	0.218705821	nn
NA12249	92	GA	1	ga	0.489292846	GA
NA11881	93	GA	1	ga	0.273774764	GA
NA12815	94	GA	1	ga	0.46327531	GA
NA12264	95	GG	1	gg	0.331962062	GG
NA18526	96	GG	1	gg	0.432939661	GG
NA18562	97	GA	1	ga	0.234092246	GA
NA18545	98	AA	1	aa	0.027678059	AA
NA18609	99	GG	1	gg	0.12614694	GG
NA18566	100	GA	1	ga	0.094249465	GA
NA18621	101	GA	1	ga	0.11993961	GA
NA18577	102	GA	0.9999	ga	0.22346519	GA
NA18635	103	GG	1	gg	0.088014847	GG
NA18524	104	AA	1	aa	0.397854965	AA
NA18537	105	GA	1	ga	0.022044186	GA
NA18572	106	GG	0.9952	gg	0.230879516	GG
NA18552	107	AA	0.9999	aa	0.016434995	AA
NA18563	108	GA	1	ga	0.10091077	GA
NA18594	109	GG	0.945	gg	0.125052126	nn
NA18624	110	GA	1	ga	0.371766549	GA
NA18592	111	GG	1	gg	0.087473126	GG
NA18529	112	GG	1	gg	0.315012828	GG
NA18603	113	AA	1	aa	0.02015174	nn
NA18547	114	GG	1	gg	0.31055457	GG
NA18611	115	AA	0.8907	***AA***	***0***	AA
NA18570	116	AA	0.9975	aa	0.003339779	AA
NA18622	117	AA	1	aa	0.308138722	AA
NA18579	118	GG	0.7505	gg	0.054278237	GG
NA18636	119	GA	1	ga	0.200585177	GA
NA18558	120	GA	1	ga	0.352735186	GA
NA18540	121	GA	0.9994	ga	0.186008718	GA
NA18609	122	GG	1	gg	0.016246281	nn
NA18555	123	AA	0.999	aa	0.102842553	AA
NA18612	124	GA	1	ga	0.313232228	GA
NA18573	125	GA	1	ga	0.254577895	GA
NA18632	126	AA	0.9999	aa	0.22744964	AA
NA18593	127	GG	1	gg	0.068327778	GG
NA18532	128	GA	1	ga	0.383399661	GA
NA18605	129	GA	1	ga	0.497222919	GA
NA18550	130	AA	1	aa	0.260076686	AA
NA18603	131	AA	1	aa	0.29685966	AA
NA18571	132	AA	1	aa	0.321169914	AA

	MACGT discrepancy					
	LDA discrepancy					
NA	LDA output were not g					
XY	Manual calls are given					
		592069				
**Coriell**	**Sample**	**LDA Call**	**LDA quality score**	**MACGT Call**	**MACGT quality score**	**HapMap Call**
NA18623	133	AA	0.9955	aa	0.191783864	AA
NA18582	134	AA	1	aa	0.357935188	AA
NA18637	135	AA	0.9973	aa	0.222942973	AA
NA18561	136	GG	1	gg	0.202002816	GG
NA18542	137	GA	0.9997	ga	0.204395992	GA
NA18608	138	GG	1	gg	0.255789204	GG
NA18564	139	GA	1	ga	0.278391927	GA
NA18620	140	GG	1	gg	0.196342678	GG
NA18576	141	GA	1	ga	0.579144877	GA
NA18633	142	GA	1	ga	0.542866503	GA
NA18594	143	GG	1	gg	0.227758346	GG
NA18942	144	AA	1	aa	0.313982459	AA
NA18945	145	GA	1	ga	0.282945001	GA
NA18964	146	GG	1	gg	0.125690585	GG
NA18961	147	GG	1	gg	0.288176194	GG
NA18967	148	GA	1	ga	0.181157135	GA
NA18981	149	GA	1	ga	0.230969947	GA
NA18994	150	AA	1	aa	0.340838877	AA
NA18998	151	GA	1	ga	0.048560022	GA
NA18940	152	GA	0.9999	ga	0.050569756	GA
NA18949	153	GA	1	ga	0.023966433	GA
NA18953	154	GA	0.9998	ga	0.027688108	GA
NA18972	155	GA	1	ga	0.032547645	GA
NA18976	156	AA	1	aa	0.25176552	AA
NA18971	157	GA	1	ga	0.171300934	GA
blank	158	NN	0.5021	***NN***	***0***	nn
NA19000	159	GA	1	ga	0.186443346	GA
NA18951	160	AA	0.9999	aa	0.260597479	nn
NA18948	161	GA	1	ga	0.184896338	GA
NA18968	162	GA	1	ga	0.208680608	GA
NA18965	163	AA	1	aa	0.525282656	AA
NA18978	164	GA	1	ga	0.23855036	GA
NA18974	165	GA	1	ga	0.19133881	GA
NA18992	166	AA	0.996	aa	0.156668604	AA
NA19005	167	GG	1	gg	0.073198239	GG
NA18943	168	GA	1	ga	0.127455703	GA
NA18951	169	AA	1	aa	0.058749156	AA
NA18959	170	GA	1	ga	0.267564204	GA
NA18973	171	AA	1	aa	0.14011941	AA
NA18970	172	AA	1	aa	0.124470434	AA
NA18987	173	GA	1	ga	0.234309792	GA
NA18995	174	GA	1	ga	0.419769633	GA
NA18999	175	GG	1	gg	0.39680084	GG
NA18947	176	GA	1	ga	0.190695538	GA

	MACGT discrepancy					
	LDA discrepancy					
NA	LDA output were not g					
XY	Manual calls are given					
		592069				
**Coriell**	**Sample**	**LDA Call**	**LDA quality score**	**MACGT Call**	**MACGT quality score**	**HapMap Call**
NA18952	177	AA	1	aa	0.07643514	AA
NA18969	178	AA	1	***AA***	***8.49E-04***	AA
NA18966	179	AA	0.9999	aa	0.015662202	AA
NA18980	180	AA	1	aa	0.008738079	AA
NA18990	181	AA	1	aa	0.002132884	AA
NA18997	182	AA	1	aa	0.034060539	AA
NA19007	183	AA	1	aa	0.003835064	AA
NA18944	184	GA	1	ga	0.018063884	GA
NA18956	185	AA	1	aa	0.23305887	AA
NA18960	186	GA	1	ga	0.267936823	GA
NA18975	187	AA	1	aa	0.029450927	AA
NA18995	188	GA	1	ga	0.294534705	nn
NA18991	189	AA	1	aa	0.270438201	AA
NA18996	190	AA	1	aa	0.111625517	nn
NA19003	191	AA	1	aa	0.397616157	AA
NA18502	192	GA	1	ga	0.03951602	GA
NA19153	193	AA	1	aa	0.348006976	AA
NA18857	194	AA	0.9998	aa	0.411181983	AA
NA19223	195	AA	1	aa	0.515055333	AA
NA19201	196	AA	1	aa	0.303699149	AA
NA18504	197	GA	0.9997	ga	0.286373487	GA
NA18870	198	GA	1	ga	0.118166007	GA
NA18863	199	AA	1	aa	0.359269818	AA
NA19145	200	AA	0.9954	aa	0.068505344	AA
NA19137	201	AA	0.9993	aa	0.283867546	AA
NA19238	202	AA	1	aa	0.08817461	nn
NA18500	203	AA	1	aa	0.010446866	AA
NA19144	204	AA	0.9999	***AA***	***0***	AA
NA19203	205	AA	1	aa	0.263766641	AA
NA19200	206	AA	1	aa	0.241920287	AA
NA18855	207	AA	0.9974	aa	0.197640511	AA
NA18505	208	AA	0.9999	aa	0.27457397	AA
NA19202	209	AA	1	aa	0.331002638	AA
NA18501	210	AA	0.9998	aa	0.374821785	AA
NA18861	211	AA	0.9989	aa	0.2031437	AA
NA19193	212	AA	1	aa	0.115390843	AA
NA19143	213	AA	0.9992	aa	0.059705734	AA
NA18517	214	AA	1	aa	0.298788238	AA
NA18862	215	AA	1	aa	0.409280757	AA
NA18856	216	AA	1	aa	0.263410401	nn
NA19239	217	AA	1	aa	0.404105856	AA
NA19240	218	AA	1	aa	0.11672409	AA
NA18856	219	AA	0.999	aa	0.578253038	AA
NA18503	220	GA	1	ga	0.094733754	GA

	MACGT discrepancy					
	LDA discrepancy					
NA	LDA output were not g					
XY	Manual calls are given					
				**592069**		
**Coriell**	**Sample**	**LDA Call**	**LDA quality score**	**MACGT Call**	**MACGT quality score**	**HapMap Call**
NA18871	221	AA	0.9999	aa	0.24465744	AA
NA19221	222	AA	1	aa	0.221119361	AA
NA19209	223	GA	1	ga	0.166200033	GA
NA19152	224	GA	1	ga	0.046434707	GA
NA18515	225	AA	0.9999	aa	0.140657887	AA
NA19238	226	AA	1	aa	0.345357377	AA
NA19154	227	GA	0.9999	ga	0.458402505	GA
NA19210	228	GA	1	ga	0.470674964	GA
NA19211	229	GA	1	ga	0.381733522	GA
NA18862	230	AA	1	aa	0.183857399	nn
NA18872	231	GA	1	ga	0.14303617	GA
NA19139	232	AA	1	aa	0.571479513	AA
NA19222	233	AA	1	aa	0.494963222	AA
NA19194	234	AA	1	aa	0.458799645	AA
NA19138	235	GA	1	ga	0.378511668	GA
NA19204	236	GA	0.75	ga	0.001315187	GA
NA18516	237	AA	1	aa	0.172935964	AA
NA19205	238	GA	1	ga	0.30248215	GA
NA19192	239	AA	1	aa	0.404313442	AA
NA18912	240	AA	1	aa	0.195440907	nn
NA18508	241	GA	1	ga	0.320118138	GA
NA19142	242	AA	1	aa	0.326587815	AA
NA18852	243	AA	1	aa	0.447273407	AA
NA18507	244	AA	1	aa	0.470916487	AA
blank	245	NN	0.5175	*NN*	*0*	nn
NA19101	246	AA	1	aa	0.557679037	AA
NA19172	247	AA	1	aa	0.306476778	AA
NA19160	248	GA	1	ga	0.229609936	GA
NA19129	249	AA	0.9999	aa	0.518109971	AA
NA18913	250	GA	0.9917	ga	0.100820787	GA
NA19120	251	AA	1	aa	0.131928357	AA
NA19159	252	AA	1	aa	0.334801397	AA
NA18523	253	GA	0.9999	ga	0.279441889	GA
NA19102	254	AA	1	aa	0.254373083	AA
NA19092	255	AA	1	aa	0.292353467	AA
NA18521	256	GA	1	ga	0.29827888	GA
NA18912	257	AA	0.9999	aa	0.155679018	AA
NA19206	258	AA	0.9997	aa	0.08421962	AA
NA19128	259	AA	1	aa	0.168738914	AA
NA18859	260	AA	1	aa	0.266448831	nn
NA19094	261	AA	1	aa	0.446259054	AA
NA19171	262	AA	1	aa	0.345024705	AA
NA18860	263	AA	1	*AA*	*0*	AA
NA18858	264	AA	1	*AA*	*0*	AA

	MACGT discrepancy					
	LDA discrepancy					
NA	LDA output were not g					
XY	Manual calls are given					
		592069				
**Coriell**	**Sample**	**LDA Call**	**LDA quality score**	**MACGT Call**	**MACGT quality score**	**HapMap Call**
NA19099	265	AA	1	***AA***	***0***	AA
NA18506	266	AA	0.9997	***AA***	***0***	AA
NA19131	267	GA	1	ga	0.012842454	GA
NA19116	268	AA	1	***AA***	***0***	AA
NA19161	269	AA	1	***AA***	***0***	AA
NA19140	270	AA	1	***AA***	***0***	AA
NA19207	271	AA	1	aa	0.158974926	AA
NA19141	272	AA	1	***AA***	***0***	AA
NA18854	273	AA	0.9999	***AA***	***0***	AA
NA19098	274	NN	0.6771	***AA***	***0***	AA
NA18522	275	GA	1	ga	0.002577136	GA
NA18914	276	GA	1	ga	0.217318552	GA
NA18853	277	AA	0.9999	aa	0.242462766	AA
NA18859	278	AA	1	***AA***	***0***	AA
NA19100	279	AA	0.7766	***AA***	***0***	AA
NA19093	280	AA	0.8605	***AA***	***0***	AA
NA19132	281	GA	0.9998	ga	0.004440518	GA
NA19130	282	AA	0.7816	***AA***	***0***	AA
NA19208	283	AA	1	***AA***	***0***	AA
NA19127	284	AA	1	aa	0.290505322	AA
NA19173	285	AA	1	aa	0.293139911	AA
NA19119	286	AA	1	***AA***	***0***	AA
NA19103	287	AA	1	***AA***	***0***	AA
	Discrepancies	0		0		
	NNs	1		0		
	Automated Calls	277		250		
	Manual Calls	0		27		
	Concordant Calls	257		258		258

	MACGT discrepancy					
	LDA discrepancy					
NA	LDA output were not g					
XY	Manual calls are given					
		**6068122**				
**Coriell**	**Sample**	**LDA Call**	**LDA quality score**	**MACGT Call**	**MACGT quality score**	**HapMap Call**
NA12753	1		NA	TT	0.436842269	
NA12875	2	AA	0.9997	aa	0.503290136	AA
NA12044	3	AT	0.964	at	0.184355239	AT
NA06993	4	AT	0.9284	at	0.052763292	AT
NA12716	5	AA	0.9998	aa	0.291894074	AA
NA12760	6	TT	0.8846	tt	0.091484387	TT
NA07348	7	AT	0.9391	at	0.021534722	AT
NA12707	8	AT	0.9624	at	0.036455887	AT
NA12156	9		NA	AA	0.361374089	
NA11992	10	AA	0.9996	aa	0.042900715	AA
NA12239	11	AT	0.8508	at	0.004790708	AT
NA12878	12	TT	0.9704	tt	0.003529305	TT
NA11993	13	AA	0.9848	aa	0.019301564	AA
NA12750	14	AA	0.9999	aa	0.193426739	AA
NA12146	15	AA	0.9865	aa	0.211668722	AA
NA11839	16	AA	0.9964	aa	0.051188599	AA
NA12156.dup	17	AA	0.984	aa	0.431644786	nn
NA11829	18		NA	AT	0.593757777	
NA12154	19	AT	0.9269	at	0.32588338	AT
NA10856	20	AA	0.7563	aa	0.36182394	AA
NA06985	21	AT	0.9277	at	0.393077438	AT
NA11831	22	AA	1	aa	0.136525671	AA
NA12003.dup	23	AA	0.9999	aa	0.193803326	nn
NA10859	24	AA	0.9983	aa	0.129201524	AA
NA07056	25	AT	0.8701	at	0.184419916	AT
NA07022	26	AA	1	aa	0.244234355	AA
NA12762	27		NA	AT	0.254781733	
NA12874	28	NN	0.6545	***AT***	***0***	AT
NA12003	29	AA	0.9999	aa	0.375224179	AA
NA10835	30	TT	0.9612	tt	0.104528189	TT
NA11882	31	AT	0.9351	at	0.46250438	AT
NA07034	32	AT	0.9621	at	0.374031266	AT
NA12056	33	AA	1	aa	0.37171596	AA
NA12145	34	AT	0.9909	at	0.476961526	AT
NA07019	35	AA	0.9998	aa	0.557071276	AA
NA06991	36		NA	AT	0.548278594	
NA12761	37	AA	0.9999	aa	0.257415256	AA
NA06994	38	AA	0.9999	aa	0.249896795	AA
NA12864	39	AA	0.9995	aa	0.330489072	AA
NA07055	40	AA	1	aa	0.374240066	AA
NA10863	41	AA	0.9999	aa	0.56337723	AA
NA12763	42	TT	0.9915	tt	0.137808616	TT
NA10831	43	AA	0.7707	aa	0.402439737	AA
NA11840	44	AT	0.9511	at	0.236940887	AT

	MACGT discrepancy					
	LDA discrepancy					
NA	LDA output were not g					
XY	Manual calls are given					
		**6068122**				
**Coriell**	**Sample**	**LDA Call**	**LDA quality score**	**MACGT Call**	**MACGT quality score**	**HapMap Call**
NA10830	45	AA	0.9243	aa	0.22848132	AA
NA11993.dup	46	AA	0.9999	aa	0.477459374	nn
NA12751	47	AA	0.9999	aa	0.205666017	AA
NA12814	48	AA	0.9999	aa	0.278659183	AA
NA10857	49	TT	0.9956	tt	0.124524791	TT
NA07357	50	AA	0.7531	aa	0.518930821	AA
NA07000	51	AA	0.998	aa	0.667697623	AA
NA12802	52	AT	0.9562	at	0.234706161	AT
NA10855	53	AA	0.9992	aa	0.436387172	AA
NA11995	54		NA	AA	0.352503079	
NA10839	55	AA	0.8456	aa	0.165105691	AA
NA10847	56	AT	0.9655	at	0.357049023	AT
NA12006	57	AA	0.9402	aa	0.291272474	AA
NA12144	58	AT	0.9656	at	0.531282226	AT
NA11832	59	AA	0.9939	aa	0.400666807	AA
NA12813	60	AT	0.9685	at	0.471716019	AT
blank	61	AA	0.9995	aa	0.384081019	nn
NA12891	62	TT	0.9423	tt	0.257174548	TT
NA12717	63		NA	AT	0.359688651	
NA10861	64	AA	0.9994	aa	0.011863164	AA
NA12004	65	AA	0.9823	***AA***	***0***	AA
NA07048	66	AA	0.9749	aa	0.15429681	AA
NA12801	67	AA	0.9968	aa	0.248117117	AA
NA11830	68	AT	0.9338	at	0.107604782	AT
NA12057	69	AT	0.8293	at	0.016845425	AT
NA11994	70		NA	AA	0.587572795	
NA12740	71	AA	0.9998	aa	0.665994988	AA
NA12005	72	AT	0.9485	at	0.174251097	AT
NA07345	73	NN	0.6274	tt	0.003292747	TT
NA10854	74	NN	0.7013	aa	0.205157736	AA
NA12872	75	AA	0.9957	aa	0.105663599	AA
NA12865	76	AA	0.9941	aa	0.292873221	AA
NA10860	77		NA	AA	0.26629255	
NA12873	78	AA	0.9648	aa	0.261491781	AA
NA12752	79	AT	0.9458	at	0.017478185	AT
NA10851	80	AA	1	aa	0.374235782	AA
NA07029	81	AA	0.8437	aa	0.270035335	AA
NA12248	82	AT	0.9558	at	0.006694986	AT
NA12155	83	AA	0.9147	***AA***	***0***	AA
NA10838	84	AA	0.8023	aa	0.144400778	AA
NA12812	85	AA	0.9798	aa	0.163900408	AA
NA12248.dup	86	AT	0.9147	at	0.005118602	nn
NA12043	87	AT	0.7568	at	0.001219821	AT
NA12234	88	AA	0.9999	aa	0.430151491	AA

	MACGT discrepancy					
	LDA discrepancy					
NA	LDA output were not g					
XY	Manual calls are given					
		**6068122**				
**Coriell**	**Sample**	**LDA Call**	**LDA quality score**	**MACGT Call**	**MACGT quality score**	**HapMap Call**
NA12892	89	AT	0.9555	at	0.037619926	AT
NA10846	90		NA	TT	0.35273432	
NA06993.dup	91	AT	0.956	at	0.056073501	nn
NA12249	92	AT	0.9585	at	0.124008812	AT
NA11881	93	AA	0.8812	aa	0.123930003	AA
NA12815	94	AT	0.9405	at	0.128252305	AT
NA12264	95	AA	0.998	aa	0.276325527	AA
NA18526	96	AA	1	aa	0.44692777	AA
NA18562	97	AA	1	aa	0.578498991	AA
NA18545	98	AA	0.9997	aa	0.271623879	AA
NA18609	99	AA	0.9998	aa	0.376919563	AA
NA18566	100	AA	1	***AA***	***0***	AA
NA18621	101	AA	0.9997	aa	0.453866424	AA
NA18577	102	AA	0.9981	aa	0.144531462	AA
NA18635	103	AA	0.9987	aa	0.236724558	AA
NA18524	104	AT	0.9645	at	0.414206642	AT
NA18537	105	AT	0.9701	at	0.264673523	AT
NA18572	106		NA	TT	0.531894057	
NA18552	107	AA	0.9997	aa	0.206320744	AA
NA18563	108	AA	0.9984	aa	0.174070113	AA
NA18594	109	AA	0.999	aa	0.3419443	nn
NA18624	110	AA	0.9984	aa	0.01702088	AA
NA18592	111	AT	0.9776	at	0.501387018	AT
NA18529	112	AA	0.9962	aa	0.465327196	AA
NA18603	113	AT	0.9751	at	0.525464803	nn
NA18547	114	AA	0.9382	aa	0.542781984	AA
NA18611	115	AA	0.999	aa	0.349172078	AA
NA18570	116	AT	0.974	at	0.316850097	AT
NA18622	117	AA	0.9973	aa	0.223569501	AA
NA18579	118	AA	0.9227	aa	0.33160405	AA
NA18636	119	TT	0.9824	tt	0.065942521	TT
NA18558	120	NN	0.7272	tt	0.107839658	TT
NA18540	121	AA	0.8571	aa	0.205681866	AA
NA18609	122	AA	0.7942	aa	0.065478956	nn
NA18555	123	AT	0.969	***AT***	***8.21E-04***	AT
NA18612	124	AA	0.9885	aa	0.324806858	AA
NA18573	125	AA	0.7887	aa	0.123972544	AA
NA18632	126	AA	0.9907	aa	0.182762061	AA
NA18593	127	AA	0.8735	aa	0.177595153	AA
NA18532	128	AA	0.7831	aa	0.223318994	AA
NA18605	129	AA	0.9947	aa	0.314020447	AA
NA18550	130	AA	0.9835	aa	0.31004838	AA
NA18603	131	AT	0.9772	at	0.20807413	AT
NA18571	132	AA	0.9987	aa	0.268194644	AA

	MACGT discrepancy					
	LDA discrepancy					
NA	LDA output were not g					
XY	Manual calls are given					
		**6068122**				
**Coriell**	**Sample**	**LDA Call**	**LDA quality score**	**MACGT Call**	**MACGT quality score**	**HapMap Call**
NA18623	133	AA	0.967	***AA***	***0***	AA
NA18582	134	AA	1	aa	0.535282186	AA
NA18637	135	AA	0.9999	aa	0.008547601	AA
NA18561	136	AA	0.977	***AA***	***6.06E-04***	AA
NA18542	137	AA	0.998	aa	0.03912921	AA
NA18608	138	AT	0.9844	***AT***	***0***	AT
NA18564	139	AA	1	aa	0.016786633	AA
NA18620	140	AT	0.9348	at	0.044489664	AT
NA18576	141	AA	0.9805	aa	0.18158128	AA
NA18633	142	AA	0.9999	aa	0.161812947	AA
NA18594	143	AA	0.7762	aa	0.376014433	AA
NA18942	144	AA	0.9922	aa	0.447942996	AA
NA18945	145	AA	0.8897	aa	0.474326833	AA
NA18964	146	AA	0.9941	aa	0.320606606	AA
NA18961	147	AA	0.9982	aa	0.575468983	AA
NA18967	148	AA	0.9991	aa	0.393938595	AA
NA18981	149	AA	0.9979	aa	0.43588592	AA
NA18994	150	AA	0.9997	aa	0.005281048	AA
NA18998	151	AA	0.9956	aa	0.105000606	AA
NA18940	152	AA	0.9372	aa	0.133947104	AA
NA18949	153	AA	0.969	aa	0.191034184	AA
NA18953	154	AA	0.9621	aa	0.08325158	AA
NA18972	155	AA	0.9656	aa	0.047751771	AA
NA18976	156	AA	0.9973	aa	0.401432388	AA
NA18971	157	AT	0.969	at	0.023093153	AT
blank	158	NN	0.5027	***NN***	***0***	nn
NA19000	159	AT	0.9642	at	0.190018744	AT
NA18951	160	AA	1	aa	0.098984973	nn
NA18948	161	AA	0.9998	aa	0.010626448	AA
NA18968	162	AA	1	aa	0.243698189	AA
NA18965	163	AA	1	aa	0.242811954	AA
NA18978	164		NA	TT	0.29072883	
NA18974	165	AT	0.9835	at	0.040572494	AT
NA18992	166	AT	0.9866	at	0.002998647	AT
NA19005	167	AA	0.9999	aa	0.329835334	AA
NA18943	168	AT	0.9156	at	0.098727047	AT
NA18951	169	AA	0.9986	aa	0.226377201	AA
NA18959	170	AA	0.9948	aa	0.344397325	AA
NA18973	171	AT	0.9692	at	0.184930026	AT
NA18970	172	AA	0.9955	aa	0.332915992	AA
NA18987	173	AT	0.9695	at	0.035172238	AT
NA18995	174	AT	0.9773	at	0.325616304	AT
NA18999	175	AA	0.9873	aa	0.26642764	AA
NA18947	176	AT	0.9692	at	0.002473855	AT

	MACGT discrepancy					
	LDA discrepancy					
NA	LDA output were not g					
XY	Manual calls are given					
		**6068122**				
**Coriell**	**Sample**	**LDA Call**	**LDA quality score**	**MACGT Call**	**MACGT quality score**	**HapMap Call**
NA18952	177	AA	0.9903	aa	0.378574059	AA
NA18969	178	AA	0.9907	aa	0.23224545	AA
NA18966	179	AT	0.9769	at	0.034167626	AT
NA18980	180	AA	0.9727	aa	0.31753849	AA
NA18990	181	AA	0.9987	aa	0.346105149	AA
NA18997	182	AA	0.9771	***AA***	***0***	AA
NA19007	183	AA	0.9925	aa	0.254187207	AA
NA18944	184	AT	0.7874	***AT***	***0***	AT
NA18956	185	AT	0.977	at	0.24654963	AT
NA18960	186	AA	0.9889	aa	0.321261771	AA
NA18975	187	AT	0.9813	at	0.167424066	AT
NA18995	188	AT	0.969	at	0.007782797	nn
NA18991	189	AA	0.9955	***AA***	***0***	AA
NA18996	190	AA	0.9965	aa	0.37000658	nn
NA19003	191	AA	0.9923	aa	0.469852339	AA
NA18502	192	AA	0.9668	aa	0.096069141	AA
NA19153	193	AT	0.9004	at	0.01066238	AT
NA18857	194	AA	0.9707	aa	0.105693764	AA
NA19223	195	AA	0.9995	aa	0.08653019	AA
NA19201	196	AA	0.9895	aa	0.116175332	AA
NA18504	197	AA	0.9951	aa	0.009415297	AA
NA18870	198	AA	0.9631	aa	0.076569564	AA
NA18863	199	AA	0.993	aa	0.018448071	AA
NA19145	200	AA	0.9919	aa	0.174666356	AA
NA19137	201	AA	0.999	aa	0.002837921	AA
NA19238	202	AA	0.9782	aa	0.225629662	nn
NA18500	203	AA	0.9955	aa	0.112622512	AA
NA19144	204	AA	0.9736	aa	0.224116745	AA
NA19203	205	AA	0.9591	aa	0.091501558	AA
NA19200	206	AA	0.9924	aa	0.173544344	AA
NA18855	207	AA	0.9971	aa	0.041461379	AA
NA18505	208	AA	0.7716	aa	0.21188951	AA
NA19202	209	AA	0.9781	aa	0.077284763	AA
NA18501	210	AA	0.9513	aa	0.022829134	AA
NA18861	211	AA	0.9738	aa	0.0041318	AA
NA19193	212	AA	0.9967	***AA***	***0***	AA
NA19143	213	NN	0.7271	aa	0.165829359	AA
NA18517	214	AA	0.984	aa	0.174232246	AA
NA18862	215	AA	0.8307	aa	0.17560394	AA
NA18856	216	AA	0.9979	aa	0.009743342	nn
NA19239	217	AA	0.996	aa	0.019937917	AA
NA19240	218	AA	0.9992	aa	0.205045563	AA
NA18856	219	AA	0.9993	aa	0.162152959	AA
NA18503	220	AA	0.9197	aa	0.036069001	AA

	MACGT discrepancy					
	LDA discrepancy					
NA	LDA output were not g					
XY	Manual calls are given					
		**6068122**				
**Coriell**	**Sample**	**LDA Call**	**LDA quality score**	**MACGT Call**	**MACGT quality score**	**HapMap Call**
NA18871	221	AA	0.9994	aa	0.022509574	AA
NA19221	222	AA	0.9998	aa	0.508147623	AA
NA19209	223	AA	0.9999	aa	0.025144996	AA
NA19152	224	AA	1	aa	0.189470316	AA
NA18515	225	AA	0.9997	aa	0.170388131	AA
NA19238	226	AA	0.9993	aa	0.201714405	AA
NA19154	227	AT	0.9824	***AT***	***0***	AT
NA19210	228	AA	0.9979	aa	0.202362646	AA
NA19211	229	AA	0.9999	aa	0.480357987	AA
NA18862	230	AA	0.9884	aa	0.232154574	nn
NA18872	231	AA	0.9959	aa	0.416198737	AA
NA19139	232	AA	0.9997	aa	0.061406597	AA
NA19222	233	AA	0.999	aa	0.043130164	AA
NA19194	234	AA	0.9997	aa	0.331245686	AA
NA19138	235	AA	0.8578	aa	0.314933674	AA
NA19204	236	AA	1	aa	0.542270426	AA
NA18516	237	AA	0.9997	aa	0.010587268	AA
NA19205	238	AA	0.9999	aa	0.220659002	AA
NA19192	239	AA	0.9974	aa	0.079802348	AA
NA18912	240	AA	0.9313	aa	0.083498522	nn
NA18508	241	AA	0.9998	aa	0.079486958	AA
NA19142	242	AA	0.9996	aa	0.02416713	AA
NA18852	243	AA	0.9999	aa	0.475028648	AA
NA18507	244	AT	0.9768	***AT***	***0***	AT
blank	245	NN	0.7191	***NN***	***0***	nn
NA19101	246	AA	0.9999	aa	0.143031624	AA
NA19172	247	AA	0.9983	aa	0.138235374	AA
NA19160	248	AA	0.9998	aa	0.105362298	AA
NA19129	249	AA	0.9999	aa	0.019409947	AA
NA18913	250	AA	0.9998	aa	0.039831748	AA
NA19120	251	AA	0.9961	aa	0.013832033	AA
NA19159	252	AA	0.9973	aa	0.061162807	AA
NA18523	253	AT	0.9791	at	0.152756359	AT
NA19102	254	AA	0.8012	aa	0.137998125	AA
NA19092	255	AA	0.9997	***AA***	***6.05E-04***	AA
NA18521	256	AA	0.999	aa	0.385023356	AA
NA18912	257	AA	0.9993	aa	0.434040644	AA
NA19206	258	AA	1	aa	0.534344325	AA
NA19128	259	AA	0.9999	aa	0.605170777	AA
NA18859	260	AA	0.9998	aa	0.602831455	nn
NA19094	261	AA	0.9992	aa	0.509166494	AA
NA19171	262	AA	0.9992	aa	0.314431762	AA
NA18860	263	AT	0.9842	at	0.210096268	AT
NA18858	264	AT	0.9678	at	0.227845615	AT

	MACGT discrepancy					
	LDA discrepancy					
NA	LDA output were not g					
XY	Manual calls are given					
		6068122				
**Coriell**	**Sample**	**LDA Call**	**LDA quality score**	**MACGT Call**	**MACGT quality score**	**HapMap Call**
NA19099	265	AA	1	aa	0.377582726	AA
NA18506	266	AA	0.9999	aa	0.240002398	AA
NA19131	267	AA	0.9995	aa	0.393644525	AA
NA19116	268	AA	0.9994	aa	0.538598858	AA
NA19161	269	AA	1	aa	0.483449007	AA
NA19140	270	AA	1	aa	0.345868512	AA
NA19207	271	AA	0.9897	aa	0.158440462	AA
NA19141	272	AA	1	aa	0.541557221	AA
NA18854	273	AA	1	aa	0.367835762	AA
NA19098	274	AA	1	aa	0.519918463	AA
NA18522	275	AA	0.9999	aa	0.267946782	AA
NA18914	276	AA	0.9264	***AA***	***0***	AA
NA18853	277	AA	0.9211	aa	0.377038426	AA
NA18859	278	AA	0.9999	aa	0.44633947	AA
NA19100	279	AA	1	aa	0.018584154	AA
NA19093	280	AA	0.9999	aa	0.510420852	AA
NA19132	281	AA	1	aa	0.349230195	AA
NA19130	282	AA	0.9999	aa	0.224313835	AA
NA19208	283	AA	0.9997	aa	0.484566515	AA
NA19127	284	AA	0.9955	aa	0.058977078	AA
NA19173	285	AA	0.9982	aa	0.35117113	AA
NA19119	286	AA	1	aa	0.456773446	AA
NA19103	287	AA	0.9999	aa	0.728857062	AA
	Discrepancies	0		0		
	NNs	5		0		
	Automated Calls	275		257		
	Manual Calls	0		18		
	Concordant Calls	251		256		256

	MACGT discrepancy					
	LDA discrepancy					
NA	LDA output were not g					
XY	Manual calls are given					
		**6478813**				
**Coriell**	**Sample**	**LDA Call**	**LDA quality score**	**MACGT Call**	**MACGT quality score**	**HapMap Call**
NA12753	1		NA	AT	0.452100125	
NA12875	2	AT	0.9974	at	0.546772251	AT
NA12044	3	AT	0.9904	at	0.599862713	AT
NA06993	4	AT	0.9165	at	0.550945124	AT
NA12716	5	TT	1	tt	0.510508535	TT
NA12760	6	TT	1	tt	0.374614593	TT
NA07348	7	TT	1	tt	0.485997205	TT
NA12707	8	TT	1	tt	0.287785958	TT
NA12156	9		NA	TT	0.266533306	
NA11992	10	AT	0.9441	at	0.042733624	AT
NA12239	11	TT	1	tt	0.433519438	TT
NA12878	12	TT	1	tt	0.210198182	TT
NA11993	13	TT	1	*TT*	*0*	TT
NA12750	14	TT	1	tt	0.143447	TT
NA12146	15	TT	1	tt	0.186527959	TT
NA11839	16	TT	1	tt	0.196004609	TT
NA12156.dup	17	TT	1	tt	0.469320649	nn
NA11829	18		NA	TT	0.498505002	
NA12154	19	AT	0.8065	at	0.455961896	AT
NA10856	20	TT	1	tt	0.291734576	TT
NA06985	21	AT	0.7847	at	0.5495509	AT
NA11831	22	TT	1	tt	0.182840251	TT
NA12003.dup	23	TT	1	tt	0.234453037	nn
NA10859	24	TT	1	tt	0.331590959	TT
NA07056	25	TT	1	tt	0.202509822	TT
NA07022	26	AT	0.8825	at	0.404177242	AT
NA12762	27		NA	AT	0.566189752	
NA12874	28	AT	0.8895	*AT*	*0*	AT
NA12003	29	TT	1	tt	0.428869377	TT
NA10835	30	AT	0.9338	at	0.43306241	AT
NA11882	31	TT	1	tt	0.396426512	TT
NA07034	32	AT	0.9316	at	0.544083098	AT
NA12056	33	TT	1	tt	0.434201786	TT
NA12145	34	TT	1	tt	0.163549832	TT
NA07019	35	AT	0.9329	at	0.433575942	AT
NA06991	36		NA	AA	0.460202737	
NA12761	37	TT	1	tt	0.200209512	TT
NA06994	38	TT	1	tt	0.199517475	TT
NA12864	39	TT	1	tt	0.466374758	TT
NA07055	40	AT	0.9895	at	0.542101169	AT
NA10863	41	AT	0.8399	at	0.547150876	AT
NA12763	42	AT	0.9068	at	0.5600318	AT
NA10831	43	AT	0.9952	at	0.569828984	AT
NA11840	44	TT	1	tt	0.216686421	TT

	MACGT discrepancy					
	LDA discrepancy					
NA	LDA output were not g					
XY	Manual calls are given					
		**6478813**				
**Coriell**	**Sample**	**LDA Call**	**LDA quality score**	**MACGT Call**	**MACGT quality score**	**HapMap Call**
NA10830	45	AT	0.9862	at	0.606789549	AT
NA11993.dup	46	TT	1	tt	0.227732707	nn
NA12751	47	TT	1	tt	0.522270626	TT
NA12814	48	TT	1	tt	0.106560843	TT
NA10857	49	AT	0.9868	at	0.652691239	AT
NA07357	50	TT	1	tt	0.360880989	TT
NA07000	51	AT	0.9715	at	0.258236161	AT
NA12802	52	TT	1	tt	0.27857372	TT
NA10855	53	TT	1	tt	0.470143109	TT
NA11995	54		NA	TT	0.314533815	
NA10839	55	TT	1	tt	0.091039716	TT
NA10847	56	TT	1	tt	0.291570556	TT
NA12006	57	TT	1	tt	0.455611058	TT
NA12144	58	TT	1	tt	0.524016073	TT
NA11832	59	TT	1	tt	0.227183083	TT
NA12813	60	TT	1	tt	0.108583621	TT
blank	61	TT	1	tt	0.263228061	nn
NA12891	62	TT	1	tt	0.431690252	TT
NA12717	63		NA	TT	0.521146193	
NA10861	64	AT	0.9815	at	0.007616218	AT
NA12004	65	TT	1	tt	0.11596094	TT
NA07048	66	AT	0.9274	at	0.281704687	AT
NA12801	67	TT	1	tt	0.199614841	TT
NA11830	68	TT	1	tt	0.211309722	TT
NA12057	69	TT	1	tt	0.329862452	TT
NA11994	70		NA	AT	0.567402674	
NA12740	71	TT	1	tt	0.367970319	TT
NA12005	72	TT	1	tt	0.046453359	TT
NA07345	73	TT	1	tt	0.14631209	TT
NA10854	74	TT	1	tt	0.509216941	TT
NA12872	75	TT	1	tt	0.44675814	TT
NA12865	76	AT	0.8218	at	0.332223378	AT
NA10860	77		NA	TT	0.422491478	
NA12873	78	TT	1	tt	0.527000682	TT
NA12752	79	TT	1	tt	0.455096531	TT
NA10851	80	TT	1	tt	0.359038692	TT
NA07029	81	AT	0.8207	at	0.448888713	AT
NA12248	82	TT	1	tt	0.345837255	TT
NA12155	83	AT	0.7816	at	0.006024463	AT
NA10838	84	TT	1	tt	0.01834654	TT
NA12812	85	TT	1	tt	0.440475098	TT
NA12248.dup	86	TT	1	tt	0.541519167	nn
NA12043	87	TT	1	tt	0.480611049	TT
NA12234	88	AT	0.9359	at	0.392561152	AT

	MACGT discrepancy					
	LDA discrepancy					
NA	LDA output were not g					
XY	Manual calls are given					
		**6478813**				
**Coriell**	**Sample**	**LDA Call**	**LDA quality score**	**MACGT Call**	**MACGT quality score**	**HapMap Call**
NA12892	89	AT	0.7789	at	0.515537373	AT
NA10846	90	TT	1	tt	0.553480911	TT
NA06993.dup	91	AT	0.9173	at	0.395308616	nn
NA12249	92	AT	0.9767	at	0.367290396	AT
NA11881	93	TT	1	tt	0.265864485	TT
NA12815	94	TT	1	tt	0.290225349	TT
NA12264	95	TT	1	tt	0.328352043	TT
NA18526	96	TT	1	tt	0.360634619	TT
NA18562	97	TT	1	tt	0.300193474	TT
NA18545	98	AT	0.9678	at	0.468673928	AT
NA18609	99	TT	1	tt	0.166466531	TT
NA18566	100	TT	1	tt	0.100818204	TT
NA18621	101	AT	0.9837	at	0.507429332	AT
NA18577	102	TT	1	tt	0.064244103	TT
NA18635	103	AT	0.9337	at	0.351692616	AT
NA18524	104	AT	0.9969	at	0.663907933	AT
NA18537	105	AT	0.9837	at	0.240721868	AT
NA18572	106	AA	0.9998	aa	0.586126501	AA
NA18552	107	TT	1	tt	0.21509441	TT
NA18563	108	TT	1	tt	0.129409083	TT
NA18594	109	TT	1	tt	0.263035927	nn
NA18624	110	TT	0.9998	tt	0.227580792	TT
NA18592	111	TT	1	tt	0.433223794	TT
NA18529	112	AT	0.8766	at	0.317584437	AT
NA18603	113	TT	1	tt	0.439122975	nn
NA18547	114	TT	1	tt	0.449598769	TT
NA18611	115	TT	1	tt	0.103411346	TT
NA18570	116	TT	1	***TT***	***0***	TT
NA18622	117	AT	0.7725	at	0.279996417	AT
NA18579	118	AT	0.8788	at	0.508497076	AT
NA18636	119	TT	1	tt	0.504858845	TT
NA18558	120	AT	0.8714	at	0.64272419	AT
NA18540	121	AT	0.8867	at	0.103248024	AT
NA18609	122	TT	0.8798	tt	0.040405149	nn
NA18555	123	AT	0.8512	at	0.431879733	AT
NA18612	124	AT	0.7758	at	0.207495632	AT
NA18573	125	AA	0.9997	aa	0.417871987	AA
NA18632	126	TT	1	tt	0.285127342	TT
NA18593	127	TT	1	tt	0.153854272	TT
NA18532	128	TT	1	tt	0.01091388	TT
NA18605	129	AT	0.8767	at	0.490663567	AT
NA18550	130	TT	1	tt	0.471934169	TT
NA18603	131	TT	1	tt	0.259983765	TT
NA18571	132	TT	1	tt	0.210384521	TT

	MACGT discrepancy					
	LDA discrepancy					
NA	LDA output were not g					
XY	Manual calls are given					
		6478813				
**Coriell**	**Sample**	**LDA Call**	**LDA quality score**	**MACGT Call**	**MACGT quality score**	**HapMap Call**
NA18623	133	TT	1	tt	0.635441537	TT
NA18582	134	AT	0.8001	at	0.511249367	AT
NA18637	135	AT	0.8231	at	0.070434172	AT
NA18561	136	TT	1	tt	0.54483958	TT
NA18542	137	TT	1	tt	0.248504065	TT
NA18608	138	TT	1	tt	0.014462129	TT
NA18564	139	TT	1	tt	0.653441925	TT
NA18620	140	TT	1	tt	0.503723308	TT
NA18576	141	AT	0.8634	at	0.434209175	AT
NA18633	142	TT	1	*TT*	*0*	TT
NA18594	143	TT	1	tt	0.257515134	TT
NA18942	144	TT	1	tt	0.343405247	TT
NA18945	145	AT	0.9947	at	0.364331507	AT
NA18964	146	TT	1	tt	0.392221679	TT
NA18961	147	AT	0.991	at	0.623287165	AT
NA18967	148	TT	1	tt	0.352568014	TT
NA18981	149	AT	0.9871	at	0.454678898	AT
NA18994	150	TT	1	tt	0.495765105	TT
NA18998	151	TT	1	tt	0.129833146	TT
NA18940	152	TT	1	tt	0.230789333	TT
NA18949	153	TT	1	tt	0.023140254	TT
NA18953	154	TT	1	tt	0.024689633	TT
NA18972	155	TT	1	tt	0.126254892	TT
NA18976	156	AT	0.7916	at	0.216956665	AT
NA18971	157	AT	0.9457	at	0.119961997	AT
blank	158	NN	0.5	*NN*	*0*	nn
NA19000	159	AT	0.7685	at	0.399845806	AT
NA18951	160	TT	1	tt	0.066324348	nn
NA18948	161	TT	1	tt	0.013806386	TT
NA18968	162	TT	1	tt	0.021621753	TT
NA18965	163	TT	1	tt	0.205501113	TT
NA18978	164	AT	0.8375	at	0.363153199	AT
NA18974	165	TT	1	tt	0.024692367	TT
NA18992	166	TT	1	*TT*	*0*	TT
NA19005	167	TT	1	tt	0.305320537	TT
NA18943	168	AT	0.9982	at	0.466866217	AT
NA18951	169	TT	1	tt	0.392562744	TT
NA18959	170	TT	1	tt	0.53130133	TT
NA18973	171	TT	1	tt	0.435701605	TT
NA18970	172	TT	1	tt	0.364949438	TT
NA18987	173	TT	1	tt	0.301396345	TT
NA18995	174	AT	0.979	at	0.539406383	AT
NA18999	175	TT	1	tt	0.589747755	TT
NA18947	176	AT	0.9987	at	0.2354601	AT

	MACGT discrepancy					
	LDA discrepancy					
NA	LDA output were not g					
XY	Manual calls are given					
		6478813				
**Coriell**	**Sample**	**LDA Call**	**LDA quality score**	**MACGT Call**	**MACGT quality score**	**HapMap Call**
NA18952	177	AA	0.9993	aa	0.424921848	AA
NA18969	178	AT	0.9919	at	0.186708198	AT
NA18966	179	TT	1	tt	0.477494365	TT
NA18980	180	TT	1	tt	0.279874954	TT
NA18990	181	AT	0.9572	at	0.424536446	AT
NA18997	182	AT	0.9758	at	0.624753691	AT
NA19007	183	AT	0.9654	at	0.133941515	AT
NA18944	184	AT	0.9662	at	0.018026698	AT
NA18956	185	AA	0.9968	aa	0.483739228	AA
NA18960	186	TT	1	tt	0.256671341	TT
NA18975	187	TT	1	tt	0.607494613	TT
NA18995	188	AT	0.9784	at	0.455752026	nn
NA18991	189	AT	0.9775	at	0.667357529	AT
NA18996	190	AT	0.869	at	0.185366138	nn
NA19003	191	TT	1	tt	0.449312366	TT
NA18502	192	AT	0.9551	at	0.312419601	AT
NA19153	193	AT	0.8546	at	0.178336118	AT
NA18857	194	AT	0.7637	at	0.218542671	AT
NA19223	195	AT	0.8288	*AT*	*0*	AT
NA19201	196	AT	0.9242	at	0.271514362	AT
NA18504	197	TT	1	tt	0.185640124	TT
NA18870	198	AT	0.8493	at	0.246057105	AT
NA18863	199	AT	0.9457	at	0.00473966	AT
NA19145	200	AT	0.8077	at	0.09889586	AT
NA19137	201	AT	0.8384	at	0.014187866	AT
NA19238	202	AA	0.9954	aa	0.590863766	nn
NA18500	203	AA	0.9979	aa	0.350845125	AA
NA19144	204	AA	0.9992	aa	0.373141193	AA
NA19203	205	TT	1	tt	0.01960013	TT
NA19200	206	AA	0.9997	aa	0.555937835	AA
NA18855	207	AA	0.9723	aa	0.459141042	AA
NA18505	208	AA	0.9973	aa	0.428699265	AA
NA19202	209	AA	0.9983	aa	0.333711635	AA
NA18501	210	AT	0.7699	at	0.086729499	AT
NA18861	211	AA	0.8872	*AA*	*0*	AA
NA19193	212	AT	0.8177	at	0.001637592	AT
NA19143	213	AT	0.9228	at	0.257323789	AT
NA18517	214	TT	1	tt	0.123718962	TT
NA18862	215	AT	0.9025	at	0.295643361	AT
NA18856	216	TT	1	tt	0.017928374	nn
NA19239	217	AT	0.7527	*AT*	*0*	AT
NA19240	218	AT	0.7592	at	0.258331768	AT
NA18856	219	TT	1	tt	0.246676781	TT
NA18503	220	AT	0.7944	at	0.083707633	AT

	MACGT discrepancy					
	LDA discrepancy					
NA	LDA output were not g					
XY	Manual calls are given					
		**6478813**				
**Coriell**	**Sample**	**LDA Call**	**LDA quality score**	**MACGT Call**	**MACGT quality score**	**HapMap Call**
NA18871	221	AT	0.9209	at	0.22484586	AT
NA19221	222	AT	0.8233	at	0.705513897	AT
NA19209	223	AT	0.896	at	0.569240835	AT
NA19152	224	AT	0.9968	at	0.486926834	AT
NA18515	225	TT	1	tt	0.149596968	TT
NA19238	226	AA	0.9959	aa	0.56033871	AA
NA19154	227	AT	0.788	at	0.492003483	AT
NA19210	228	AT	0.8091	at	0.186976661	AT
NA19211	229	TT	1	tt	0.263745088	TT
NA18862	230	AT	0.7557	at	0.214381799	nn
NA18872	231	AT	0.8439	at	0.427488704	AT
NA19139	232	AA	0.9994	aa	0.419278111	AA
NA19222	233	AT	0.7969	at	0.22705889	AT
NA19194	234	TT	1	tt	0.401866195	TT
NA19138	235	AT	0.8522	at	0.366936913	AT
NA19204	236	AT	0.9945	at	0.078812992	AT
NA18516	237	TT	1	tt	0.038994381	TT
NA19205	238	TT	1	tt	0.229493872	TT
NA19192	239	TT	1	tt	0.014278012	TT
NA18912	240	AT	0.8939	at	0.305817407	nn
NA18508	241	AT	0.841	at	0.252584725	AT
NA19142	242		NA	AA	0.335070168	
NA18852	243	AT	0.8003	at	0.395823613	AT
NA18507	244	TT	1	tt	0.002155322	TT
blank	245	NN	0.5	***NN***	***0***	nn
NA19101	246	TT	1	tt	0.047162954	TT
NA19172	247	AT	0.85	at	0.061637198	AT
NA19160	248	AA	0.9784	aa	0.541516512	AA
NA19129	249	AT	0.7784	at	0.04509978	AT
NA18913	250	AA	0.9896	aa	0.551122771	AA
NA19120	251		NA	AA	0.398329666	
NA19159	252	AA	0.9996	aa	0.410246234	AA
NA18523	253	AA	0.9997	aa	0.484586032	AA
NA19102	254	AT	0.8294	at	0.041511061	AT
NA19092	255	TT	1	tt	0.093383562	TT
NA18521	256	AA	0.9985	aa	0.642983235	AA
NA18912	257	AT	0.7864	at	0.116655271	AT
NA19206	258	AT	0.8088	at	0.41882698	AT
NA19128	259	AA	0.999	aa	0.470277844	AA
NA18859	260	AA	0.9994	aa	0.654902756	nn
NA19094	261	AT	0.7728	at	0.401462362	AT
NA19171	262	TT	1	tt	0.418399777	TT
NA18860	263	AT	0.9754	at	0.317227313	AT
NA18858	264	AT	0.8772	at	0.614084762	AT

	MACGT discrepancy					
	LDA discrepancy					
NA	LDA output were not g					
XY	Manual calls are given					
		6478813				
**Coriell**	**Sample**	**LDA Call**	**LDA quality score**	**MACGT Call**	**MACGT quality score**	**HapMap Call**
NA19099	265	AA	0.9996	aa	0.367421941	AA
NA18506	266	TT	1	tt	0.227354372	TT
NA19131	267	AT	0.9755	at	0.249761641	AT
NA19116	268	AT	0.969	at	0.229154545	AT
NA19161	269	AA	0.9991	aa	0.417278689	AA
NA19140	270	AA	0.9993	aa	0.413753871	AA
NA19207	271	AT	0.7815	at	0.02309031	AT
NA19141	272	AA	0.9964	aa	0.401601834	AA
NA18854	273	AT	0.9933	at	0.413269441	AT
NA19098	274	TT	1	tt	0.055200995	TT
NA18522	275	AA	0.9946	aa	0.347315926	AA
NA18914	276	AA	0.9848	aa	0.149224585	AA
NA18853	277	AT	0.7831	at	0.017873696	AT
NA18859	278	AA	0.9957	aa	0.355634925	AA
NA19100	279	AT	0.8906	at	0.003056627	AT
NA19093	280	AT	0.9931	at	0.121428053	AT
NA19132	281	AT	0.9912	at	0.389826832	AT
NA19130	282	AT	0.9663	at	0.089770396	AT
NA19208	283	AA	0.9904	***AA***	***0***	AA
NA19127	284	AT	0.7775	at	0.096344606	AT
NA19173	285	TT	1	tt	0.225344895	TT
NA19119	286	AA	0.9973	aa	0.090525322	AA
NA19103	287	TT	0.9969	***TT***	***0***	TT
	Discrepancies	0		0		
	NNs	0		0		
	Automated Calls	276		264		
	Manual Calls	0		12		
	Concordant Calls	257		257		257

	MACGT discrepancy					
	LDA discrepancy					
NA	LDA output were not g					
XY	Manual calls are given					
		**7292634**				
**Coriell**	**Sample**	**LDA Call**	**LDA quality score**	**MACGT Call**	**MACGT quality score**	**HapMap Call**
NA12753	1		NA	CT	0.442293492	
NA12875	2	CC	0.8233	cc	0.072123699	CC
NA12044	3	CC	0.9719	cc	0.236566582	CC
NA06993	4	NN	0.6281	cc	0.166502368	CC
NA12716	5	CT	0.9418	ct	0.613626578	CT
NA12760	6	CC	0.9045	cc	0.065459129	CC
NA07348	7	CC	0.8071	cc	0.087762265	CC
NA12707	8	CC	0.9073	cc	0.191491959	CC
NA12156	9		NA	CC	0.223288299	
NA11992	10	NN	0.7142	cc	0.034502976	CC
NA12239	11	NN	0.5974	cc	0.088188505	CC
NA12878	12	NN	0.6001	cc	0.012894575	CC
NA11993	13	CC	0.9951	cc	0.126457556	CC
NA12750	14	CC	0.983	cc	0.189125884	CC
NA12146	15	NN	0.7304	ct	0.182746918	CT
NA11839	16	CC	0.9963	cc	0.087096133	CC
NA12156.dup	17	CC	0.9913	cc	0.165430662	nn
NA11829	18		NA	CC	0.237282323	
NA12154	19	CC	0.977	cc	0.14709032	CC
NA10856	20	CC	0.9719	cc	0.309624631	CC
NA06985	21	CC	0.977	cc	0.129666388	CC
NA11831	22	CC	0.8423	cc	0.106297326	CC
NA12003.dup	23	CC	0.7675	cc	0.198413928	nn
NA10859	24	CC	0.9824	cc	0.35399196	CC
NA07056	25	CC	0.9397	cc	0.150587688	CC
NA07022	26	CC	0.8246	cc	0.09607176	CC
NA12762	27		NA	CC	0.457437335	
NA12874	28	CC	0.9963	cc	0.090097516	CC
NA12003	29	CC	0.9799	cc	0.207326863	CC
NA10835	30	CC	0.9916	cc	0.054912453	CC
NA11882	31	CC	0.9492	cc	0.317520445	CC
NA07034	32	CC	0.9314	cc	0.180875626	CC
NA12056	33	CC	0.9542	cc	0.251005738	CC
NA12145	34	CC	0.9948	cc	0.312657059	CC
NA07019	35	CC	0.9893	cc	0.445693626	CC
NA06991	36		NA	CC	0.44863767	
NA12761	37	CC	0.9646	cc	0.192118207	CC
NA06994	38	CC	0.9873	cc	0.417870637	CC
NA12864	39	CC	0.9903	cc	0.343283348	CC
NA07055	40	CC	0.9933	cc	0.291332616	CC
NA10863	41	CC	0.9906	cc	0.368520426	CC
NA12763	42	CT	0.7915	ct	0.220258844	CT
NA10831	43	CC	0.9882	cc	0.061031544	CC
NA11840	44	CC	0.9891	cc	0.419625512	CC

	MACGT discrepancy					
	LDA discrepancy					
NA	LDA output were not g					
XY	Manual calls are given					
		**7292634**				
**Coriell**	**Sample**	**LDA Call**	**LDA quality score**	**MACGT Call**	**MACGT quality score**	**HapMap Call**
NA10830	45	CC	0.9982	cc	0.290137425	CC
NA11993.dup	46	CC	0.9679	cc	0.065387506	nn
NA12751	47	CC	0.8729	cc	0.194427695	CC
NA12814	48	CT	0.8992	ct	0.131183201	CT
NA10857	49	CC	0.9875	cc	0.373751188	CC
NA07357	50	CC	0.9521	cc	0.172187299	CC
NA07000	51	CC	0.9825	cc	0.306021863	CC
NA12802	52	CT	0.8646	ct	0.071716481	CT
NA10855	53	NN	0.7385	cc	0.262955688	CC
NA11995	54		NA	CC	0.258733255	
NA10839	55	CC	0.9711	cc	0.348066434	CC
NA10847	56		NA	CT	0.38048421	
NA12006	57	CC	0.9836	cc	0.293084947	CC
NA12144	58	CT	0.8275	ct	0.356889161	CT
NA11832	59	CC	0.9529	cc	0.312133652	CC
NA12813	60	CC	0.9834	cc	0.403257303	CC
blank	61	CC	0.9928	cc	0.249281867	nn
NA12891	62	CC	0.8841	cc	0.20397936	CC
NA12717	63		NA	CC	0.240902095	
NA10861	64	CC	0.9766	cc	0.047461853	CC
NA12004	65	CC	0.9585	cc	0.293843534	CC
NA07048	66	CC	0.9366	cc	0.468677517	CC
NA12801	67	CC	0.977	cc	0.121951223	CC
NA11830	68	CC	0.971	cc	0.069500172	CC
NA12057	69	CC	0.9622	cc	0.313275595	CC
NA11994	70		NA	CC	0.525073027	
NA12740	71	CC	0.9853	cc	0.459912254	CC
NA12005	72	CC	0.8943	cc	0.046206579	CC
NA07345	73	CC	0.8902	cc	0.04251662	CC
NA10854	74	CC	0.8628	cc	0.197493193	CC
NA12872	75	CC	0.9436	cc	0.540182593	CC
NA12865	76	NN	0.736	cc	0.411731798	CC
NA10860	77		NA	CC	0.293303662	
NA12873	78	CC	0.7875	cc	0.316444044	CC
NA12752	79	CC	0.966	cc	0.284184109	CC
NA10851	80	CC	0.9997	cc	0.318077639	CC
NA07029	81	CC	0.8637	cc	0.371331132	CC
NA12248	82	CC	0.921	cc	0.313914603	CC
NA12155	83	CC	0.852	*CC*	*0*	CC
NA10838	84	CC	0.8617	cc	0.414083987	CC
NA12812	85	CC	0.9285	cc	0.462441599	CC
NA12248.dup	86	CC	0.8358	cc	0.268676927	nn
NA12043	87	CC	0.9473	cc	0.38086154	CC
NA12234	88	CC	0.9982	cc	0.316233218	CC

	MACGT discrepancy					
	LDA discrepancy					
NA	LDA output were not g					
XY	Manual calls are given					
		**7292634**				
**Coriell**	**Sample**	**LDA Call**	**LDA quality score**	**MACGT Call**	**MACGT quality score**	**HapMap Call**
NA12892	89	NN	0.7006	cc	0.479688492	CC
NA10846	90	NN	0.6087	cc	0.392044735	CC
NA06993.dup	91	CC	0.967	cc	0.288246363	nn
NA12249	92	CC	0.9102	cc	0.046074732	CC
NA11881	93	CC	0.921	cc	0.171849999	CC
NA12815	94	NN	0.6132	cc	0.052919206	CC
NA12264	95	CC	0.9604	cc	0.084983547	CC
NA18526	96	CC	0.9968	cc	0.48533469	CC
NA18562	97	CC	0.9018	cc	0.300051905	CC
NA18545	98	CC	0.988	cc	0.184952915	CC
NA18609	99	CC	0.9934	cc	0.235474798	CC
NA18566	100	CC	0.9823	cc	0.124132275	CC
NA18621	101	CT	0.8399	ct	0.477699541	CT
NA18577	102	CC	0.9926	cc	0.00259108	CC
NA18635	103	CC	0.996	cc	0.12843824	CC
NA18524	104	CC	0.9986	cc	0.342565613	CC
NA18537	105	CC	0.9769	cc	0.136563885	CC
NA18572	106	CC	0.974	cc	0.251154323	CC
NA18552	107	CC	0.9839	cc	0.207014702	CC
NA18563	108	CC	0.9744	cc	0.05374371	CC
NA18594	109	CC	0.966	cc	0.21141867	nn
NA18624	110	CC	0.9608	cc	0.450226585	CC
NA18592	111	CC	0.9937	cc	0.203324707	CC
NA18529	112	CC	0.9888	cc	0.325222192	CC
NA18603	113	CC	0.964	cc	0.500639931	nn
NA18547	114	CC	0.9573	cc	0.435413193	CC
NA18611	115	CC	0.9895	cc	0.042122315	CC
NA18570	116	CC	0.9853	cc	0.151239941	CC
NA18622	117	CC	0.9615	cc	0.015785198	CC
NA18579	118	CC	0.7807	cc	0.046846905	CC
NA18636	119	CC	0.9621	cc	0.375941653	CC
NA18558	120	NN	0.6816	cc	0.255226609	CC
NA18540	121	NN	0.698	cc	0.022087254	CC
NA18609	122	NN	0.6086	cc	0.020773958	nn
NA18555	123	NN	0.5339	*CC*	*0*	CC
NA18612	124	CC	0.9712	cc	0.486450405	CC
NA18573	125	NN	0.5939	cc	0.019122837	CC
NA18632	126	NN	0.7177	cc	0.357805425	CC
NA18593	127	CC	0.758	cc	0.340458049	CC
NA18532	128	CC	0.8726	cc	0.371514035	CC
NA18605	129	NN	0.6387	cc	0.195337293	CC
NA18550	130	NN	0.618	cc	0.266045752	CC
NA18603	131	CC	0.9741	cc	0.382695607	CC
NA18571	132	NN	0.5808	cc	0.251634512	CC

	MACGT discrepancy					
	LDA discrepancy					
NA	LDA output were not g					
XY	Manual calls are given					
		7292634				
**Coriell**	**Sample**	**LDA Call**	**LDA quality score**	**MACGT Call**	**MACGT quality score**	**HapMap Call**
NA18623	133	NN	0.5878	cc	0.249312462	CC
NA18582	134	CC	0.9721	cc	0.149413809	CC
NA18637	135	CC	0.9966	cc	0.009162507	CC
NA18561	136	NN	0.6804	cc	0.327291882	CC
NA18542	137	NN	0.5637	cc	0.151290559	CC
NA18608	138	CC	0.9909	cc	0.262964083	CC
NA18564	139	NN	0.6014	cc	0.00269371	CC
NA18620	140	NN	0.6076	cc	0.345734146	CC
NA18576	141	NN	0.6168	cc	0.228772128	CC
NA18633	142	NN	0.6271	cc	0.13406322	CC
NA18594	143	CC	0.9593	cc	0.527973073	CC
NA18942	144	CC	0.9895	cc	0.129019563	CC
NA18945	145	CC	0.9908	cc	0.311291944	CC
NA18964	146	NN	0.6995	cc	0.292223069	CC
NA18961	147	NN	0.6802	cc	0.008181494	CC
NA18967	148	CC	0.8857	cc	0.317381108	CC
NA18981	149	NN	0.6867	cc	0.405027278	CC
NA18994	150	NN	0.6897	ct	0.053913887	CT
NA18998	151	CT	0.7773	ct	0.228803948	CT
NA18940	152	NN	0.6437	ct	0.177926736	CT
NA18949	153	CC	0.8914	cc	0.393990108	CC
NA18953	154	CC	0.7616	cc	0.1713359	CC
NA18972	155	NN	0.6605	cc	0.251449658	CC
NA18976	156	CC	0.9804	cc	0.362684989	CC
NA18971	157	CC	0.8988	cc	0.050114095	CC
blank	158	NN	0.5301	***NN***	***0***	nn
NA19000	159	CC	0.9713	cc	0.084509034	CC
NA18951	160	CC	0.9691	cc	0.057990899	nn
NA18948	161	CC	0.9857	cc	0.011151997	CC
NA18968	162	CC	0.9767	cc	0.335010329	CC
NA18965	163	CC	0.9645	cc	0.004005518	CC
NA18978	164	CC	0.9845	cc	0.211982179	CC
NA18974	165	CC	0.9909	cc	0.002226795	CC
NA18992	166	CC	0.9863	cc	0.007479048	CC
NA19005	167		NA	CT	0.309993663	
NA18943	168	CC	0.9644	cc	0.306188662	CC
NA18951	169	CC	0.9885	cc	0.363640991	CC
NA18959	170	CC	0.9622	cc	0.548458461	CC
NA18973	171	CC	0.98	cc	0.407958071	CC
NA18970	172	CC	0.9323	cc	0.165360462	CC
NA18987	173	CC	0.981	cc	0.612113973	CC
NA18995	174	CC	0.9479	cc	0.480855854	CC
NA18999	175	NN	0.7143	***CC***	***0***	CC
NA18947	176	CC	0.9891	cc	0.203424309	CC

	MACGT discrepancy					
	LDA discrepancy					
NA	LDA output were not g					
XY	Manual calls are given					
		**7292634**				
**Coriell**	**Sample**	**LDA Call**	**LDA quality score**	**MACGT Call**	**MACGT quality score**	**HapMap Call**
NA18952	177	CC	0.9887	cc	0.34707766	CC
NA18969	178	CC	0.9908	cc	0.565545896	CC
NA18966	179	CC	0.9563	cc	0.44454716	CC
NA18980	180	CC	0.9359	cc	0.201210031	CC
NA18990	181	CC	0.9615	cc	0.256987477	CC
NA18997	182	CC	0.9522	cc	0.30182529	CC
NA19007	183	NN	0.713	cc	0.119237893	CC
NA18944	184	NN	0.6173	cc	0.012767697	CC
NA18956	185	CC	0.8102	cc	0.129505428	CC
NA18960	186	CC	0.8525	cc	0.254844798	CC
NA18975	187	CC	0.8775	cc	0.438589168	CC
NA18995	188	NN	0.6214	cc	0.278048041	nn
NA18991	189	NN	0.6207	cc	0.129574508	CC
NA18996	190	NN	0.6876	cc	0.029337952	nn
NA19003	191	NN	0.6049	cc	0.107284632	CC
NA18502	192	CT	0.8898	ct	0.340876924	CT
NA19153	193	NN	0.7323	***CT***	***0***	CT
NA18857	194	NN	0.5905	cc	0.068077987	CC
NA19223	195	NN	0.6063	cc	0.147480279	CC
NA19201	196	NN	0.6171	***CC***	***0***	CC
NA18504	197	NN	0.5184	cc	0.003854192	CC
NA18870	198	NN	0.5772	cc	0.092972818	CC
NA18863	199	CT	0.898	ct	0.1579323	CT
NA19145	200	NN	0.6068	cc	0.100003543	CC
NA19137	201	CT	0.7908	ct	0.283078184	CT
NA19238	202	CC	0.992	cc	0.159573173	nn
NA18500	203	NN	0.7173	ct	0.121551805	CT
NA19144	204	NN	0.7341	cc	0.237661547	CC
NA19203	205	CC	0.9719	cc	0.066691145	CC
NA19200	206	NN	0.6932	cc	0.241073481	CC
NA18855	207	CT	0.8966	ct	0.375608835	CT
NA18505	208	CC	0.9665	cc	0.301608742	CC
NA19202	209	NN	0.6155	cc	0.032526762	CC
NA18501	210	CT	0.9347	ct	0.364199835	CT
NA18861	211	NN	0.63	ct	0.248032109	CT
NA19193	212	NN	0.6751	ct	0.276398212	CT
NA19143	213	CC	0.9951	cc	0.226978074	CC
NA18517	214	CC	0.9503	cc	0.212341434	CC
NA18862	215	CC	0.8882	cc	0.348647278	CC
NA18856	216	CC	0.983	cc	0.08342512	nn
NA19239	217	CC	0.933	cc	0.001012716	CC
NA19240	218	CC	0.9776	cc	0.194816512	CC
NA18856	219	CC	0.846	cc	0.146465373	CC
NA18503	220	NN	0.7463	cc	0.154091227	CC

	MACGT discrepancy					
	LDA discrepancy					
NA	LDA output were not g					
XY	Manual calls are given					
		**7292634**				
**Coriell**	**Sample**	**LDA Call**	**LDA quality score**	**MACGT Call**	**MACGT quality score**	**HapMap Call**
NA18871	221	CC	0.9939	cc	0.045947841	CC
NA19221	222	CC	0.9752	cc	0.093828339	CC
NA19209	223	CC	0.9772	cc	0.218770564	CC
NA19152	224	CT	0.8332	ct	0.232665782	CT
NA18515	225	CC	0.9161	cc	0.293135343	CC
NA19238	226	CC	0.9723	cc	0.013023256	CC
NA19154	227	CC	0.9757	cc	0.384175282	CC
NA19210	228	CT	0.8212	ct	0.484024962	CT
NA19211	229	CC	0.9902	cc	0.361567589	CC
NA18862	230	CC	0.8583	cc	0.016897475	nn
NA18872	231	CC	0.9836	cc	0.489486287	CC
NA19139	232	CC	0.9564	***CC***	***0***	CC
NA19222	233	CC	0.9416	cc	0.206507784	CC
NA19194	234	CC	0.9486	cc	0.104496787	CC
NA19138	235	CC	0.983	cc	0.02082946	CC
NA19204	236	CC	0.9889	cc	0.144341982	CC
NA18516	237	CC	0.7644	cc	0.17388237	CC
NA19205	238	CC	0.765	cc	0.197371625	CC
NA19192	239	CC	0.9643	cc	0.070135442	CC
NA18912	240	NN	0.6985	***CC***	***0***	nn
NA18508	241	NN	0.6811	cc	0.084672202	CC
NA19142	242	NN	0.7381	cc	0.013218531	CC
NA18852	243		NA	TT	0.506665327	
NA18507	244	NN	0.6877	***CC***	***0***	CC
blank	245	NN	0.5002	***NN***	***0***	nn
NA19101	246	CC	0.9654	cc	0.010226171	CC
NA19172	247	CT	0.8387	ct	0.225783717	CT
NA19160	248	CC	0.965	cc	0.237149141	CC
NA19129	249	CC	0.7879	cc	0.012437247	CC
NA18913	250	NN	0.6733	cc	0.086626307	CC
NA19120	251	CC	0.9595	cc	0.167306885	CC
NA19159	252	CC	0.9207	cc	0.020966077	CC
NA18523	253	CC	0.8222	cc	0.30821076	CC
NA19102	254		NA	CT	0.386151719	
NA19092	255	CC	0.9923	cc	0.347726919	CC
NA18521	256	CT	0.8588	ct	0.090332108	CT
NA18912	257	CC	0.9314	cc	0.242355865	CC
NA19206	258	CC	0.7758	cc	0.298621928	CC
NA19128	259	NN	0.7354	cc	0.156134209	CC
NA18859	260	CC	0.8513	cc	0.263076961	nn
NA19094	261	CC	0.9087	cc	0.370451125	CC
NA19171	262	CC	0.9746	cc	0.376521891	CC
NA18860	263	CC	0.9836	cc	0.272550618	CC
NA18858	264	CC	0.9708	cc	0.243868417	CC

	MACGT discrepancy					
	LDA discrepancy					
NA	LDA output were not g					
XY	Manual calls are given					
		7292634				
**Coriell**	**Sample**	**LDA Call**	**LDA quality score**	**MACGT Call**	**MACGT quality score**	**HapMap Call**
NA19099	265	CC	0.989	cc	0.146797223	CC
NA18506	266	CC	0.9637	cc	0.05903521	CC
NA19131	267	CC	0.9666	cc	0.170057816	CC
NA19116	268	CC	0.9915	cc	0.178690537	CC
NA19161	269	CC	0.9249	cc	0.113885245	CC
NA19140	270	NN	0.5246	ct	0.200125929	CT
NA19207	271	CC	0.9634	cc	0.017863689	CC
NA19141	272	CT	0.9081	ct	0.184540131	CT
NA18854	273	NN	0.6043	ct	0.225882516	CT
NA19098	274	CC	0.9869	cc	0.149700097	CC
NA18522	275	NN	0.5026	tt	0.007155684	TT
NA18914	276	CC	0.9927	cc	0.231666033	CC
NA18853	277	CC	0.9745	***CC***	***5.69E-04***	CC
NA18859	278	CC	0.9515	cc	0.144875626	CC
NA19100	279	CC	0.9526	cc	0.155817634	CC
NA19093	280	CC	0.9091	cc	0.164928641	CC
NA19132	281	CC	0.9883	cc	0.05159494	CC
NA19130	282	CC	0.978	cc	0.132822242	CC
NA19208	283	CC	0.9824	cc	0.255139109	CC
NA19127	284	CC	0.987	cc	0.321873955	CC
NA19173	285	CC	0.782	cc	0.001174346	CC
NA19119	286	CC	0.9762	cc	0.152711949	CC
NA19103	287	CC	0.9814	cc	0.158747951	CC
	Discrepancies	0		0		
	NNs	62		0		
	Automated Calls	274		263		
	Manual Calls	0		11		
	Concordant Calls	197		255		255

	MACGT discrepancy					
	LDA discrepancy					
NA	LDA output were not g					
XY	Manual calls are given					
		**7555995**				
**Coriell**	**Sample**	**LDA Call**	**LDA quality score**	**MACGT Call**	**MACGT quality score**	**HapMap Call**
NA12753	1		NA	CC	0.263452355	
NA12875	2	CG	0.8976	cg	0.105099262	CG
NA12044	3	CC	0.9845	cc	0.253693078	CC
NA06993	4	CC	0.9864	cc	0.157362669	CC
NA12716	5	CC	0.9851	cc	0.102627587	CC
NA12760	6	CG	0.9072	cg	0.161180176	CG
NA07348	7	CC	0.9811	cc	0.105977992	CC
NA12707	8	CC	0.8954	cc	0.087063046	CC
NA12156	9		NA	CG	0.051290286	
NA11992	10	CG	0.9171	cg	0.061847035	CG
NA12239	11	CC	0.9771	cc	0.118937012	CC
NA12878	12	CC	0.9791	cc	0.235085995	CC
NA11993	13	CC	0.9915	cc	0.240490252	CC
NA12750	14	CC	0.9885	cc	0.093697441	CC
NA12146	15	CG	0.9496	cg	0.207339143	CG
NA11839	16	CC	0.9898	cc	0.345201832	CC
NA12156.dup	17	CG	0.9181	cg	0.160490718	nn
NA11829	18		NA	CC	0.333135821	
NA12154	19	CG	0.915	cg	0.140414538	CG
NA10856	20	CC	0.9824	cc	0.010452421	CC
NA06985	21	CG	0.9128	cg	0.277246405	CG
NA11831	22	CC	0.877	cc	0.018396648	CC
NA12003.dup	23	CC	0.9889	cc	0.029015002	nn
NA10859	24	CC	0.9298	cc	0.129458072	CC
NA07056	25	CC	0.9886	cc	0.243846456	CC
NA07022	26	CC	0.9895	***CC***	***0***	CC
NA12762	27		NA	CC	0.245137561	
NA12874	28	CC	0.8991	***cc***	***9.52E-04***	CC
NA12003	29	CC	0.9873	cc	0.426577206	CC
NA10835	30	CC	0.9869	cc	0.317060028	CC
NA11882	31	CC	0.9864	cc	0.490294892	CC
NA07034	32	CC	0.8832	cc	0.006917085	CC
NA12056	33	CG	0.8956	cg	0.460578635	CG
NA12145	34	CC	0.9898	cc	0.055298025	CC
NA07019	35	CC	0.9885	cc	0.088986185	CC
NA06991	36		NA	CG	0.401530099	
NA12761	37	CC	0.8676	cc	0.028266146	CC
NA06994	38	CG	0.9428	cg	0.381365874	CG
NA12864	39	CC	0.9859	cc	0.027164105	CC
NA07055	40	GG	0.9999	gg	0.170791756	GG
NA10863	41	CC	0.9878	cc	0.051195023	CC
NA12763	42	CG	0.9086	cg	0.371407445	CG
NA10831	43	CG	0.9176	cg	0.333605472	CG
NA11840	44	CG	0.9069	cg	0.417434638	CG

	MACGT discrepancy					
	LDA discrepancy					
NA	LDA output were not g					
XY	Manual calls are given					
		**7555995**				
**Coriell**	**Sample**	**LDA Call**	**LDA quality score**	**MACGT Call**	**MACGT quality score**	**HapMap Call**
NA10830	45	CC	0.9871	cc	0.290836119	CC
NA11993.dup	46	CC	0.986	cc	0.087280582	nn
NA12751	47	CG	0.9054	cg	0.417154372	CG
NA12814	48	CC	0.9845	cc	0.002095268	CC
NA10857	49	CC	0.988	cc	0.384167063	CC
NA07357	50	CC	0.9847	cc	0.288750668	CC
NA07000	51	GG	1	gg	0.282832187	GG
NA12802	52	CC	0.8908	cc	0.039804886	CC
NA10855	53	CG	0.9048	cg	0.142558534	CG
NA11995	54		NA	CG	0.288883655	
NA10839	55	CC	0.8785	cc	0.051079217	CC
NA10847	56	CG	0.9441	cg	0.311717221	CG
NA12006	57	CC	0.985	***CC***	***8.81E-04***	CC
NA12144	58	CC	0.8765	cc	0.414487079	CC
NA11832	59	GG	1	gg	0.3565206	GG
NA12813	60	CC	0.9825	cc	0.236105668	CC
blank	61	CG	0.9209	cg	0.181841943	nn
NA12891	62	CC	0.9851	cc	0.301070079	CC
NA12717	63		NA	CC	0.369121704	
NA10861	64	CC	0.9841	***CC***	***0***	CC
NA12004	65	CC	0.9046	cc	0.014818602	CC
NA07048	66	CG	0.9083	cg	0.498816709	CG
NA12801	67	CC	0.9768	cc	0.023230523	CC
NA11830	68	CG	0.8761	cg	0.286084766	CG
NA12057	69	CC	0.9831	cc	0.224727956	CC
NA11994	70		NA	CC	0.463710262	
NA12740	71	CG	0.8987	cg	0.333518744	CG
NA12005	72	CC	0.9217	cc	0.218797897	CC
NA07345	73	CC	0.9916	cc	0.389793699	CC
NA10854	74	CC	0.9901	cc	0.441508052	CC
NA12872	75	CC	0.9903	cc	0.411181626	CC
NA12865	76	CC	0.9131	cc	0.355355678	CC
NA10860	77		NA	CC	0.25778596	
NA12873	78	CC	0.9904	cc	0.149413711	CC
NA12752	79	CC	0.9336	cc	0.240489439	CC
NA10851	80	CC	0.9872	cc	0.447811007	CC
NA07029	81	CG	0.947	cg	0.040974913	CG
NA12248	82	CC	0.9414	cc	0.227134551	CC
NA12155	83	NN	0.7489	gg	0.009756684	GG
NA10838	84	CC	0.9833	cc	0.020300064	CC
NA12812	85	CC	0.9851	cc	0.03369795	CC
NA12248.dup	86	CC	0.9524	cc	0.01893806	nn
NA12043	87	CC	0.984	cc	0.138626814	CC
NA12234	88	CC	0.9798	cc	0.249013626	CC

	MACGT discrepancy					
	LDA discrepancy					
NA	LDA output were not g					
XY	Manual calls are given					
		7555995				
**Coriell**	**Sample**	**LDA Call**	**LDA quality score**	**MACGT Call**	**MACGT quality score**	**HapMap Call**
NA12892	89	CC	0.9879	cc	0.162806958	CC
NA10846	90	CC	0.9174	cc	0.298588239	CC
NA06993.dup	91	CC	0.9865	cc	0.324981308	nn
NA12249	92	CC	0.9901	cc	0.255020553	CC
NA11881	93	CC	0.9425	cc	0.221338674	CC
NA12815	94	CG	0.9212	cg	0.001170542	CG
NA12264	95	CC	0.9903	cc	0.507451468	CC
NA18526	96	CG	0.9587	cg	0.020238524	CG
NA18562	97	GG	1	gg	0.536723037	GG
NA18545	98	CC	0.8965	cc	0.192754846	CC
NA18609	99	CC	0.8583	cc	0.124200032	CC
NA18566	100	CG	0.9404	cg	0.193935216	CG
NA18621	101		NA	GG	0.362196324	
NA18577	102	CG	0.9661	cg	0.025944965	CG
NA18635	103	CG	0.9543	cg	0.025635665	CG
NA18524	104	CG	0.9366	cg	0.182804203	CG
NA18537	105	CG	0.9537	cg	0.011493698	CG
NA18572	106	CG	0.936	cg	0.103270746	CG
NA18552	107	CG	0.9621	cg	0.005295889	CG
NA18563	108	CG	0.9594	cg	0.00996049	CG
NA18594	109	CG	0.9376	cg	0.059074179	nn
NA18624	110	GG	0.9998	gg	0.474351946	GG
NA18592	111	CG	0.9494	cg	0.023020356	CG
NA18529	112	CG	0.9354	cg	0.128698092	CG
NA18603	113	CG	0.9477	cg	0.015065477	nn
NA18547	114	GG	0.9992	gg	0.443203608	GG
NA18611	115		NA	GG	0.156019168	
NA18570	116	CG	0.9546	cg	0.01160259	CG
NA18622	117	CG	0.9542	cg	0.113112324	CG
NA18579	118	CG	0.952	cg	0.037957708	CG
NA18636	119	GG	0.9904	gg	0.372370746	GG
NA18558	120	CC	0.8809	cc	0.169464354	CC
NA18540	121	CG	0.9559	cg	0.053390608	CG
NA18609	122	CC	0.8823	cc	0.080984468	nn
NA18555	123	CG	0.9417	cg	0.089971423	CG
NA18612	124	CG	0.9478	cg	0.039014313	CG
NA18573	125	CG	0.9507	cg	0.010387365	CG
NA18632	126	CG	0.946	cg	0.019351784	CG
NA18593	127	CG	0.9402	cg	0.26781295	CG
NA18532	128	CG	0.9505	cg	0.021919335	CG
NA18605	129	CG	0.9431	cg	0.087351625	CG
NA18550	130	CG	0.9454	cg	0.208761214	CG
NA18603	131	CG	0.9621	cg	0.008983722	CG
NA18571	132	CG	0.9492	cg	0.160474946	CG

	MACGT discrepancy					
	LDA discrepancy					
NA	LDA output were not g					
XY	Manual calls are given					
		7555995				
**Coriell**	**Sample**	**LDA Call**	**LDA quality score**	**MACGT Call**	**MACGT quality score**	**HapMap Call**
NA18623	133	CG	0.9359	cg	0.01174858	CG
NA18582	134	CC	0.8977	cc	0.354746576	CC
NA18637	135	CG	0.9466	cg	0.073921222	CG
NA18561	136	GG	0.9943	gg	0.421543154	GG
NA18542	137	CG	0.9455	cg	0.010515222	CG
NA18608	138	CC	0.8617	cc	0.134107996	CC
NA18564	139	CG	0.9576	cg	0.007772588	CG
NA18620	140	GG	1	gg	0.32105603	GG
NA18576	141	CC	0.9023	cc	0.491065456	CC
NA18633	142	GG	0.9993	gg	0.508754747	GG
NA18594	143	CG	0.9456	cg	0.112975086	CG
NA18942	144	CG	0.9601	cg	0.024024972	CG
NA18945	145	CG	0.9361	cg	0.219291376	CG
NA18964	146	CG	0.9445	cg	0.171444275	CG
NA18961	147	CG	0.9434	cg	0.182869265	CG
NA18967	148	CG	0.919	cg	0.41999194	CG
NA18981	149	CG	0.9684	cg	0.01267005	CG
NA18994	150	CC	0.8507	cc	0.310120417	CC
NA18998	151	GG	0.9904	gg	0.13632825	GG
NA18940	152	CG	0.9648	cg	0.002205402	CG
NA18949	153	CG	0.9656	cg	0.001676452	CG
NA18953	154	CG	0.9582	***CG***	***0***	CG
NA18972	155	CG	0.9333	cg	0.051596429	CG
NA18976	156	CC	0.8799	cc	0.066665708	CC
NA18971	157	CC	0.8532	cc	0.117669445	CC
blank	158	NN	0.5002	***NN***	***0***	nn
NA19000	159		NA	GG	0.618715342	
NA18951	160	CG	0.943	cg	0.082422076	nn
NA18948	161	CG	0.9537	cg	0.055909426	CG
NA18968	162	CG	0.9242	cg	0.137394128	CG
NA18965	163	CC	0.8883	cc	0.121204213	CC
NA18978	164	CG	0.9535	cg	0.008057373	CG
NA18974	165	CG	0.9508	cg	0.00378007	CG
NA18992	166	CG	0.9365	cg	0.007166662	CG
NA19005	167	CG	0.951	cg	0.035132093	CG
NA18943	168	GG	1	gg	0.191901935	GG
NA18951	169	CG	0.9528	cg	0.189934093	CG
NA18959	170	GG	1	gg	0.597592271	GG
NA18973	171	CG	0.9596	cg	0.015461188	CG
NA18970	172	CG	0.9479	cg	0.123754377	CG
NA18987	173		NA	GG	0.482597242	
NA18995	174	CC	0.853	cc	0.152282859	CC
NA18999	175	CG	0.9154	cg	0.220602634	CG
NA18947	176	CC	0.8875	cc	0.008117543	CC

	MACGT discrepancy					
	LDA discrepancy					
NA	LDA output were not g					
XY	Manual calls are given					
		7555995				
**Coriell**	**Sample**	**LDA Call**	**LDA quality score**	**MACGT Call**	**MACGT quality score**	**HapMap Call**
NA18952	177	GG	0.989	gg	0.361598544	GG
NA18969	178	CG	0.962	cg	0.007755248	CG
NA18966	179	GG	1	gg	0.413634478	GG
NA18980	180	CG	0.9615	cg	0.002521138	CG
NA18990	181	CC	0.8789	cc	0.008666976	CC
NA18997	182		NA	GG	0.392000885	
NA19007	183		NA	GG	0.399545115	
NA18944	184	CC	0.8439	*CC*	*0*	CC
NA18956	185	CC	0.9852	cc	0.236001041	CC
NA18960	186	CG	0.9186	cg	0.294933793	CG
NA18975	187	GG	0.9997	gg	0.379488597	GG
NA18995	188	CC	0.8543	cc	0.25562908	nn
NA18991	189	GG	0.9997	gg	0.520025782	GG
NA18996	190	CG	0.9405	*CG*	*5.97E-04*	nn
NA19003	191	CG	0.9599	cg	0.021676451	CG
NA18502	192	CG	0.9387	cg	0.034787184	CG
NA19153	193	CC	0.8879	cc	0.12226582	CC
NA18857	194	CG	0.9472	cg	0.083479401	CG
NA19223	195	CC	0.892	cc	0.174074179	CC
NA19201	196	CG	0.9451	cg	0.042336463	CG
NA18504	197	GG	1	gg	0.564429407	GG
NA18870	198	CC	0.8749	cc	0.138578722	CC
NA18863	199	CC	0.8734	cc	0.00687388	CC
NA19145	200	CC	0.986	cc	0.021770429	CC
NA19137	201	CG	0.9293	cg	0.068551717	CG
NA19238	202	CC	0.8996	*CC*	*0*	nn
NA18500	203	GG	0.8658	gg	0.079572842	GG
NA19144	204	CC	0.979	cc	0.026933062	CC
NA19203	205	CC	0.847	cc	0.069663264	CC
NA19200	206	CC	0.8876	cc	0.321814986	CC
NA18855	207	GG	0.9751	gg	0.350473935	GG
NA18505	208	CG	0.9424	cg	0.002615074	CG
NA19202	209	CG	0.9546	cg	0.054120089	CG
NA18501	210	CG	0.9178	cg	0.045304156	CG
NA18861	211	CC	0.8604	*CC*	*0*	CC
NA19193	212	CG	0.9548	cg	0.008649403	CG
NA19143	213	CC	0.9767	*CC*	*0*	CC
NA18517	214	GG	0.9773	gg	0.235667766	GG
NA18862	215	CC	0.8933	cc	0.054248672	CC
NA18856	216	CC	0.9131	*CC*	*5.69E-04*	nn
NA19239	217	CG	0.9363	cg	0.058481699	CG
NA19240	218	CG	0.9596	cg	0.106057426	CG
NA18856	219	CC	0.9117	cc	0.00447146	CC
NA18503	220	GG	0.9988	gg	0.338894388	GG

	MACGT discrepancy					
	LDA discrepancy					
NA	LDA output were not g					
XY	Manual calls are given					
		**7555995**				
**Coriell**	**Sample**	**LDA Call**	**LDA quality score**	**MACGT Call**	**MACGT quality score**	**HapMap Call**
NA18871	221	CG	0.9554	cg	0.046494324	CG
NA19221	222	CC	0.8738	cc	0.279522261	CC
NA19209	223	CC	0.8976	cc	0.128511687	CC
NA19152	224	CC	0.9879	cc	0.016196509	CC
NA18515	225	GG	0.9997	gg	0.303818627	GG
NA19238	226	CC	0.882	cc	0.172232426	CC
NA19154	227	CC	0.8999	cc	0.538617034	CC
NA19210	228	CC	0.8971	cc	0.021490688	CC
NA19211	229	CC	0.9033	cc	0.027430707	CC
NA18862	230	CC	0.8587	cc	0.26799324	nn
NA18872	231	CC	0.8951	cc	0.271566701	CC
NA19139	232	CG	0.9555	cg	0.046282653	CG
NA19222	233	CG	0.9406	cg	0.018411141	CG
NA19194	234	CC	0.8572	cc	0.298393436	CC
NA19138	235	CG	0.9418	cg	0.002953833	CG
NA19204	236	CG	0.8641	cg	0.08796819	CG
NA18516	237	GG	1	gg	0.230700499	GG
NA19205	238	CC	0.8734	cc	0.197207683	CC
NA19192	239	CC	0.8637	cc	0.117218581	CC
NA18912	240	CC	0.8636	cc	0.085555792	nn
NA18508	241	CC	0.8802	cc	0.191742413	CC
NA19142	242	GG	0.9708	gg	0.275777669	GG
NA18852	243	CC	0.8911	cc	0.252959775	CC
NA18507	244	GG	0.955	gg	0.248074408	GG
blank	245	NN	0.5602	***NN***	***0***	nn
NA19101	246	GG	0.9353	gg	0.248307283	GG
NA19172	247	CG	0.9438	cg	0.01097309	CG
NA19160	248	CC	0.8688	cc	0.002458827	CC
NA19129	249	CC	0.8769	cc	0.025345606	CC
NA18913	250	CC	0.9768	cc	0.225741287	CC
NA19120	251	GG	0.9018	gg	0.075411446	GG
NA19159	252	CC	0.8792	***CC***	***0***	CC
NA18523	253	CC	0.8838	cc	0.368000523	CC
NA19102	254	CG	0.9308	***cg***	***9.87E-04***	CG
NA19092	255	GG	0.9999	gg	0.408071813	GG
NA18521	256	CC	0.8802	cc	0.153626439	CC
NA18912	257	CC	0.8903	cc	0.00732718	CC
NA19206	258	CG	0.9364	cg	0.620463157	CG
NA19128	259	CC	0.8973	cc	0.384652555	CC
NA18859	260	CG	0.9411	cg	0.29727855	nn
NA19094	261	CG	0.9378	cg	0.204893793	CG
NA19171	262	CG	0.9509	cg	0.103425015	CG
NA18860	263	CG	0.8898	cg	0.060678572	CG
NA18858	264	CG	0.8885	cg	0.452526508	CG

	MACGT discrepancy					
	LDA discrepancy					
NA	LDA output were not g					
XY	Manual calls are given					
		7555995				
**Coriell**	**Sample**	**LDA Call**	**LDA quality score**	**MACGT Call**	**MACGT quality score**	**HapMap Call**
NA19099	265	CC	0.9068	cc	0.006095837	CC
NA18506	266	CG	0.9246	cg	0.002803093	CG
NA19131	267	CC	0.9026	***CC***	***0***	CC
NA19116	268	CG	0.9057	cg	0.021187241	CG
NA19161	269	CC	0.8774	***CC***	***0***	CC
NA19140	270	CG	0.9194	cg	0.060949693	CG
NA19207	271	CC	0.984	cc	0.032571359	CC
NA19141	272	CG	0.9219	cg	0.08137502	CG
NA18854	273	CC	0.8812	cc	0.03353825	CC
NA19098	274	CC	0.8602	cc	0.069721836	CC
NA18522	275	CG	0.8615	***CG***	***0***	CG
NA18914	276	CC	0.9044	cc	0.02451569	CC
NA18853	277	CG	0.9539	cg	0.011535884	CG
NA18859	278	CG	0.91	cg	0.040906048	CG
NA19100	279	CC	0.8721	***CC***	***0***	CC
NA19093	280	CC	0.9847	cc	0.046116904	CC
NA19132	281	CG	0.9296	cg	0.023340096	CG
NA19130	282	CG	0.8905	cg	0.019257418	CG
NA19208	283	CC	0.9846	***CC***	***0***	CC
NA19127	284	CG	0.9547	cg	0.010180163	CG
NA19173	285	CG	0.9561	cg	0.00488738	CG
NA19119	286	CG	0.8728	cg	0.10153964	CG
NA19103	287	CG	0.9321	cg	0.031288776	CG
	Discrepancies	0		0		
	NNs	1		0		
	Automated Calls	272		252		
	Manual Calls	0		20		
	Concordant Calls	252		253		253

	MACGT discrepancy					
	LDA discrepancy					
NA	LDA output were not g					
XY	Manual calls are given					
		**803422**				
**Coriell**	**Sample**	**LDA Call**	**LDA quality score**	**MACGT Call**	**MACGT quality score**	**HapMap Call**
NA12753	1		NA	AA	0.270224896	
NA12875	2	GG	0.9254	gg	0.387829807	GG
NA12044	3	GA	0.9183	ga	0.109031552	GA
NA06993	4	GA	0.938	ga	0.137750911	GA
NA12716	5	GA	0.9427	ga	0.241138106	GA
NA12760	6	GA	0.7664	ga	0.080411793	GA
NA07348	7	GA	0.9321	ga	0.100858439	GA
NA12707	8	GA	0.8837	ga	0.031317119	GA
NA12156	9		NA	GG	0.225947291	
NA11992	10	GA	0.9451	ga	0.03622092	GA
NA12239	11	GG	0.9318	gg	0.323008225	GG
NA12878	12	GG	0.9155	gg	0.261313465	GG
NA11993	13	GA	0.9693	ga	0.002170565	GA
NA12750	14	GG	0.9627	gg	0.027891819	GG
NA12146	15	GG	0.9374	gg	0.057437457	GG
NA11839	16	GA	0.9286	ga	0.060916976	GA
NA12156.dup	17	GG	0.9762	gg	0.16531892	nn
NA11829	18		NA	GG	0.451790635	
NA12154	19	GA	0.9124	ga	0.160015149	GA
NA10856	20	GG	0.9311	gg	0.278907995	GG
NA06985	21	GG	0.9491	gg	0.176206682	GG
NA11831	22	AA	0.9897	aa	0.041385782	AA
NA12003.dup	23	GA	0.75	ga	0.179179399	nn
NA10859	24	GA	0.9377	ga	0.424474078	GA
NA07056	25	GA	0.9192	ga	0.173277718	GA
NA07022	26	GG	0.9578	gg	0.108178414	GG
NA12762	27		NA	AA	0.459696379	
NA12874	28	GA	0.8943	ga	0.15055865	GA
NA12003	29	GA	0.9112	ga	0.492172712	GA
NA10835	30	GG	0.9686	gg	0.535976143	GG
NA11882	31	AA	0.9952	aa	0.279287942	AA
NA07034	32	GG	0.8222	gg	0.228915159	GG
NA12056	33	GG	0.9982	gg	0.079124356	GG
NA12145	34	GG	0.9996	gg	0.309119255	GG
NA07019	35	GA	0.9076	ga	0.146957293	GA
NA06991	36		NA	GG	0.320899449	
NA12761	37	AA	0.9952	aa	0.028159855	AA
NA06994	38	GA	0.9294	ga	0.39307399	GA
NA12864	39	GG	0.9122	***GG***	***0***	GG
NA07055	40	GG	0.9624	gg	0.488317471	GG
NA10863	41	GG	0.9994	gg	0.089761408	GG
NA12763	42	GA	0.9147	ga	0.366153811	GA
NA10831	43	GG	0.9568	gg	0.438717029	GG
NA11840	44	GA	0.7766	ga	0.441183309	GA

	MACGT discrepancy					
	LDA discrepancy					
NA	LDA output were not g					
XY	Manual calls are given					
		**803422**				
**Coriell**	**Sample**	**LDA Call**	**LDA quality score**	**MACGT Call**	**MACGT quality score**	**HapMap Call**
NA10830	45	GG	0.938	gg	0.065045485	GG
NA11993.dup	46	GA	0.8791	ga	0.477104796	nn
NA12751	47	GA	0.9432	ga	0.463526913	GA
NA12814	48	GG	0.7823	gg	0.03187285	GG
NA10857	49	GG	0.9994	gg	0.472279452	GG
NA07357	50	GG	0.9977	gg	0.446060934	GG
NA07000	51	GG	0.8834	gg	0.134737467	GG
NA12802	52	GG	0.9769	gg	0.041223396	GG
NA10855	53	GA	0.8884	ga	0.216461101	GA
NA11995	54		NA	AA	0.277298315	
NA10839	55	GG	0.7822	gg	0.062737139	GG
NA10847	56	GG	0.8833	gg	0.278168689	GG
NA12006	57	GA	0.8909	ga	0.108636567	GA
NA12144	58	GA	0.8604	ga	0.15048543	GA
NA11832	59	GA	0.8555	ga	0.070729544	GA
NA12813	60	AA	0.9717	aa	0.203054196	AA
blank	61	AA	0.9759	aa	0.17166081	nn
NA12891	62	GG	0.7971	gg	0.049378029	GG
NA12717	63		NA	GG	0.290184944	
NA10861	64	GA	0.7548	ga	0.060640039	GA
NA12004	65	GA	0.8447	ga	0.028833123	GA
NA07048	66	GG	0.9303	***GG***	***7.33E-04***	GG
NA12801	67	GA	0.8277	ga	0.020030232	GA
NA11830	68	GG	0.9984	gg	0.180947434	GG
NA12057	69	GG	0.9987	gg	0.042149018	GG
NA11994	70		NA	GA	0.384905616	
NA12740	71	GG	0.9808	gg	0.190931596	GG
NA12005	72	GA	0.8962	ga	0.00550743	GA
NA07345	73	AA	0.9881	aa	0.030363073	AA
NA10854	74	GA	0.8834	ga	0.219984171	GA
NA12872	75	GG	0.9081	gg	0.377542518	GG
NA12865	76	GA	0.8586	ga	0.150259225	GA
NA10860	77		NA	AA	0.272131114	
NA12873	78	GG	0.9439	gg	0.500110514	GG
NA12752	79	AA	0.9811	aa	0.423782531	AA
NA10851	80	GG	0.9994	gg	0.629190471	GG
NA07029	81	GA	0.8472	ga	0.132220847	GA
NA12248	82	GA	0.8612	ga	0.039929526	GA
NA12155	83	GG	0.8585	***GG***	***5.17E-04***	GG
NA10838	84	GA	0.8692	ga	0.078658176	GA
NA12812	85	NN	0.7258	ga	0.037996425	GA
NA12248.dup	86	GA	0.7961	ga	0.027307605	nn
NA12043	87	GG	0.9153	gg	0.479081931	GG
NA12234	88	GG	0.9994	gg	0.256909862	GG

	MACGT discrepancy					
	LDA discrepancy					
NA	LDA output were not g					
XY	Manual calls are given					
		803422				
**Coriell**	**Sample**	**LDA Call**	**LDA quality score**	**MACGT Call**	**MACGT quality score**	**HapMap Call**
NA12892	89	GA	0.7556	ga	0.119145669	GA
NA10846	90	GG	0.9982	gg	0.309414743	GG
NA06993.dup	91	GA	0.8708	ga	0.03496674	nn
NA12249	92	GG	0.9985	gg	0.208893844	GG
NA11881	93	GG	0.8641	gg	0.224079187	GG
NA12815	94	GA	0.848	ga	0.093940826	GA
NA12264	95	GG	0.9985	gg	0.541934265	GG
NA18526	96	GG	0.9995	gg	0.445780903	GG
NA18562	97	GG	0.9975	gg	0.214881841	GG
NA18545	98	GG	0.9459	gg	0.300308573	GG
NA18609	99	GG	0.9991	gg	0.269673348	GG
NA18566	100	GG	0.938	gg	0.10486055	GG
NA18621	101	GG	0.9295	gg	0.067333165	GG
NA18577	102	GG	0.935	gg	0.299489972	GG
NA18635	103	GG	0.9988	gg	0.474733736	GG
NA18524	104	GG	0.9995	gg	0.573699378	GG
NA18537	105	GG	0.9592	gg	0.11751882	GG
NA18572	106	GG	0.9991	gg	0.414459583	GG
NA18552	107	GG	0.9992	gg	0.30575931	GG
NA18563	108	GG	0.9422	gg	0.252501403	GG
NA18594	109	GG	0.9409	gg	0.284032315	nn
NA18624	110	GG	0.9278	gg	0.410287782	GG
NA18592	111	GG	0.8984	gg	0.137226396	GG
NA18529	112	GG	0.9347	gg	0.556905295	GG
NA18603	113	GG	0.9964	gg	0.196089648	nn
NA18547	114	GG	0.9984	gg	0.528402932	GG
NA18611	115	GG	0.8978	gg	0.146686523	GG
NA18570	116	GG	0.891	gg	0.235749168	GG
NA18622	117	GG	0.9027	gg	0.443741266	GG
NA18579	118	GG	0.896	gg	0.277174051	GG
NA18636	119	GG	0.9992	gg	0.414136633	GG
NA18558	120	GG	0.8983	gg	0.56172331	GG
NA18540	121	GG	0.8587	gg	0.078606817	GG
NA18609	122	GG	0.916	gg	0.322566965	nn
NA18555	123	GG	0.8042	gg	0.007779149	GG
NA18612	124	GG	0.9988	gg	0.355496978	GG
NA18573	125	GG	0.8679	gg	0.240191133	GG
NA18632	126	GG	0.7776	gg	0.36381569	GG
NA18593	127	GG	0.8554	gg	0.438351626	GG
NA18532	128	GG	0.7634	gg	0.404973431	GG
NA18605	129	GG	0.9982	gg	0.49509621	GG
NA18550	130	GG	0.9985	gg	0.505148332	GG
NA18603	131	GG	0.9991	gg	0.518011727	GG
NA18571	132	GG	0.894	gg	0.348746674	GG

	MACGT discrepancy					
	LDA discrepancy					
NA	LDA output were not g					
XY	Manual calls are given					
		**803422**				
**Coriell**	**Sample**	**LDA Call**	**LDA quality score**	**MACGT Call**	**MACGT quality score**	**HapMap Call**
NA18623	133	GG	0.9256	gg	0.515877565	GG
NA18582	134	GG	0.9235	gg	0.20643839	GG
NA18637	135	GG	0.9193	gg	0.021527149	GG
NA18561	136	GG	0.8648	gg	0.080788473	GG
NA18542	137	GG	0.9488	gg	0.367355179	GG
NA18608	138	GG	0.9297	gg	0.437252182	GG
NA18564	139	GG	0.7593	gg	0.506328967	GG
NA18620	140	GG	0.9488	gg	0.465514887	GG
NA18576	141	GG	0.75	gg	0.421925736	GG
NA18633	142	NN	0.75	gg	0.331693425	GG
NA18594	143	GG	0.9079	gg	0.43278167	GG
NA18942	144	GG	0.9444	gg	0.07035119	GG
NA18945	145	GG	0.9133	gg	0.503164318	GG
NA18964	146	GG	0.9515	gg	0.455367011	GG
NA18961	147	GG	0.9331	***GG***	***0***	GG
NA18967	148	GG	0.9423	gg	0.005865811	GG
NA18981	149	GG	0.9463	gg	0.452969406	GG
NA18994	150	GG	0.9468	gg	0.464953039	GG
NA18998	151	GG	0.927	gg	0.178715314	GG
NA18940	152	GG	0.9074	gg	0.37898973	GG
NA18949	153	GG	0.9062	gg	0.296457364	GG
NA18953	154	GG	0.7903	gg	0.002558652	GG
NA18972	155	GG	0.999	gg	0.132789828	GG
NA18976	156	GG	0.7855	gg	0.507273758	GG
NA18971	157	GG	0.9157	gg	0.223772814	GG
blank	158	NN	0.5	***NN***	***0***	nn
NA19000	159	GG	0.9189	gg	0.169722011	GG
NA18951	160	GG	0.9323	gg	0.097216992	nn
NA18948	161	GG	0.9348	gg	0.029071856	GG
NA18968	162	GG	0.9357	gg	0.263439523	GG
NA18965	163	GG	0.9001	gg	0.247318302	GG
NA18978	164	GG	0.9473	gg	0.193174641	GG
NA18974	165	GG	0.9097	gg	0.051181749	GG
NA18992	166	GG	0.9441	gg	0.002385505	GG
NA19005	167	GG	0.9973	gg	0.116934722	GG
NA18943	168	GG	0.819	gg	0.080127186	GG
NA18951	169	NN	0.659	gg	0.052261217	GG
NA18959	170	GG	0.9166	gg	0.476273299	GG
NA18973	171	NN	0.75	gg	0.527856094	GG
NA18970	172	GG	0.9069	gg	0.401886386	GG
NA18987	173	GG	0.999	gg	0.008764892	GG
NA18995	174	GG	0.8972	gg	0.148274303	GG
NA18999	175	GG	0.9168	gg	0.486756932	GG
NA18947	176	GG	0.8954	gg	0.458789454	GG

	MACGT discrepancy					
	LDA discrepancy					
NA	LDA output were not g					
XY	Manual calls are given					
		**803422**				
**Coriell**	**Sample**	**LDA Call**	**LDA quality score**	**MACGT Call**	**MACGT quality score**	**HapMap Call**
NA18952	177	GG	0.9199	gg	0.467872453	GG
NA18969	178	GG	0.9042	gg	0.449242691	GG
NA18966	179	GG	0.8979	gg	0.542117198	GG
NA18980	180	GG	0.8176	gg	0.002979602	GG
NA18990	181	GG	0.8067	gg	0.232202422	GG
NA18997	182	GG	0.7889	gg	0.504918956	GG
NA19007	183	GG	0.9971	gg	0.276714967	GG
NA18944	184	NN	0.7076	gg	0.068222227	GG
NA18956	185	GG	0.9287	gg	0.254987052	GG
NA18960	186	GG	0.9993	gg	0.383821513	GG
NA18975	187	GG	0.8808	gg	0.115701664	GG
NA18995	188	GG	0.8958	gg	0.230472922	nn
NA18991	189	GG	0.9062	gg	0.400963905	GG
NA18996	190	GG	0.9055	gg	0.21288877	nn
NA19003	191	GG	0.9467	gg	0.530750229	GG
NA18502	192	GG	0.913	***GG***	***0***	GG
NA19153	193	GA	0.9106	ga	0.106456339	GA
NA18857	194	GG	0.8122	gg	0.061996467	GG
NA19223	195	GG	0.9136	gg	0.562552406	GG
NA19201	196	NN	0.75	gg	0.272292658	GG
NA18504	197	GA	0.75	ga	0.042985129	GA
NA18870	198	GA	0.9005	ga	0.108576304	GA
NA18863	199	NN	0.75	gg	0.031826048	GG
NA19145	200	GG	0.7848	gg	0.398313033	GG
NA19137	201	GG	0.8972	gg	0.003343684	GG
NA19238	202	GG	0.9124	gg	0.421100337	nn
NA18500	203	GG	0.8796	gg	0.400739987	GG
NA19144	204	GG	0.9198	gg	0.175133062	GG
NA19203	205	GG	0.9029	gg	0.078562814	GG
NA19200	206	GG	0.8867	gg	0.305553232	GG
NA18855	207	GG	0.8799	gg	0.058151028	GG
NA18505	208	GG	0.9942	gg	0.263540939	GG
NA19202	209	GG	0.999	gg	0.185934247	GG
NA18501	210	GG	0.9299	gg	0.050148202	GG
NA18861	211	GG	0.8948	gg	0.012587225	GG
NA19193	212	GG	0.9163	***GG***	***0***	GG
NA19143	213	GG	0.9351	gg	0.008596566	GG
NA18517	214	GG	0.9323	gg	0.090631254	GG
NA18862	215	GG	0.9279	gg	0.410164049	GG
NA18856	216	GG	0.9459	gg	0.006169431	nn
NA19239	217	GG	0.9986	gg	0.02955655	GG
NA19240	218	GG	0.9992	gg	0.216784564	GG
NA18856	219	GG	0.8277	gg	0.508562779	GG
NA18503	220	GG	0.9087	gg	0.163515159	GG

	MACGT discrepancy					
	LDA discrepancy					
NA	LDA output were not g					
XY	Manual calls are given					
		**803422**				
**Coriell**	**Sample**	**LDA Call**	**LDA quality score**	**MACGT Call**	**MACGT quality score**	**HapMap Call**
NA18871	221	GA	0.9322	ga	0.314001088	GA
NA19221	222	GG	0.9403	gg	0.076845105	GG
NA19209	223	GG	0.9229	gg	0.035998406	GG
NA19152	224	GG	0.9996	gg	0.130674694	GG
NA18515	225	GG	0.8841	gg	0.013873798	GG
NA19238	226	GG	0.8774	gg	0.18243016	GG
NA19154	227	GA	0.9165	ga	0.256154277	GA
NA19210	228	GG	0.9473	gg	0.149442417	GG
NA19211	229	GG	0.9359	gg	0.638645978	GG
NA18862	230	NN	0.7499	gg	0.041265353	nn
NA18872	231	GG	0.9389	gg	0.389687678	GG
NA19139	232	GG	0.9088	gg	0.035521349	GG
NA19222	233	GG	0.8929	gg	0.375369013	GG
NA19194	234	GG	0.9039	gg	0.331134061	GG
NA19138	235	GG	0.9049	gg	0.546314562	GG
NA19204	236	GA	0.8958	ga	0.048800773	GA
NA18516	237	GG	0.7617	gg	0.014837766	GG
NA19205	238	GA	0.9501	ga	0.107084925	GA
NA19192	239	GG	0.9262	gg	0.117556666	GG
NA18912	240	GG	0.8807	gg	0.00312295	nn
NA18508	241	GG	0.872	gg	0.261573281	GG
NA19142	242	GG	0.8857	gg	0.031877524	GG
NA18852	243	GG	0.9994	gg	0.328655216	GG
NA18507	244	GG	0.9325	gg	0.004371807	GG
blank	245	NN	0.6323	***NN***	***0***	nn
NA19101	246	GG	0.8837	gg	0.113699034	GG
NA19172	247	GG	0.9324	gg	0.047762534	GG
NA19160	248	GG	0.9345	gg	0.236465375	GG
NA19129	249	GG	0.8913	gg	0.047785604	GG
NA18913	250	GG	0.9395	gg	0.294966911	GG
NA19120	251	GG	0.9252	gg	0.087498165	GG
NA19159	252	GG	0.9993	gg	0.236580813	GG
NA18523	253	GG	0.9233	gg	0.399499951	GG
NA19102	254	GG	0.8939	gg	0.141219361	GG
NA19092	255	GG	0.9035	gg	0.383356854	GG
NA18521	256	GG	0.8963	gg	0.12398553	GG
NA18912	257	GG	0.7779	gg	0.117679531	GG
NA19206	258	GG	0.9994	gg	0.142075839	GG
NA19128	259	GA	0.9291	ga	0.549127712	GA
NA18859	260	GG	0.9992	gg	0.381940166	nn
NA19094	261	NN	0.6635	gg	0.459214037	GG
NA19171	262	GG	0.9315	***GG***	***0***	GG
NA18860	263	GG	0.9995	gg	0.137202079	GG
NA18858	264	GG	0.9996	gg	0.269450479	GG

	MACGT discrepancy					
	LDA discrepancy					
NA	LDA output were not g					
XY	Manual calls are given					
		803422				
**Coriell**	**Sample**	**LDA Call**	**LDA quality score**	**MACGT Call**	**MACGT quality score**	**HapMap Call**
NA19099	265	GG	0.9991	gg	0.065641015	GG
NA18506	266	GG	0.9036	***GG***	***0***	GG
NA19131	267	GG	0.9989	gg	0.057609654	GG
NA19116	268	GG	0.9992	gg	0.254954821	GG
NA19161	269	GG	0.9995	gg	0.085045854	GG
NA19140	270	GG	0.9994	gg	0.171560989	GG
NA19207	271	GG	0.9986	gg	0.151244012	GG
NA19141	272	GG	0.9994	gg	0.190711347	GG
NA18854	273	GG	0.9994	gg	0.060959522	GG
NA19098	274	GA	0.9027	ga	0.106436321	GA
NA18522	275	GG	0.9993	gg	0.159161678	GG
NA18914	276	GG	0.8971	gg	0.120234606	GG
NA18853	277	GG	0.9275	gg	0.140044273	GG
NA18859	278	GG	0.9996	gg	0.06494761	GG
NA19100	279	GG	0.9973	gg	0.149495091	GG
NA19093	280	GG	0.933	gg	0.161322193	GG
NA19132	281	GG	0.934	gg	0.089153705	GG
NA19130	282	GG	0.9978	gg	0.096761157	GG
NA19208	283	GG	0.9993	gg	0.086175261	GG
NA19127	284	GG	0.9311	gg	0.345000677	GG
NA19173	285	GG	0.9046	gg	0.469922695	GG
NA19119	286	GG	0.9995	gg	0.272567378	GG
NA19103	287	GG	0.9992	gg	0.058745427	GG
	Discrepancies	0		0		
	NNs	9		0		
	Automated Calls	278		268		
	Manual Calls	0		10		
	Concordant Calls	251		259		259

	MACGT discrepancy					
	LDA discrepancy					
NA	LDA output were not g					
XY	Manual calls are given					
		**846752**				
**Coriell**	**Sample**	**LDA Call**	**LDA quality score**	**MACGT Call**	**MACGT quality score**	**HapMap Call**
NA12753	1		NA	CC	0.504356529	
NA12875	2	CG	1	cg	0.26184608	CG
NA12044	3	GG	1	gg	0.177586701	GG
NA06993	4	CG	1	cg	0.105505645	CG
NA12716	5	CG	1	cg	0.091389514	CG
NA12760	6	CG	1	cg	0.276799107	CG
NA07348	7	GG	1	gg	0.192213002	GG
NA12707	8	GG	0.9997	gg	0.004359406	GG
NA12156	9		NA	GG	0.316758766	
NA11992	10	CG	1	cg	0.019563305	CG
NA12239	11	CC	1	cc	0.005015936	CC
NA12878	12	CG	1	cg	0.069052065	CG
NA11993	13	CC	1	cc	0.304414874	CC
NA12750	14	CC	0.9901	cc	0.329283008	CC
NA12146	15	GG	0.9998	***GG***	***0***	GG
NA11839	16	CG	1	cg	0.219450033	CG
NA12156.dup	17	GG	1	gg	0.135879319	nn
NA11829	18		NA	CG	0.093439389	
NA12154	19	CC	0.9966	cc	0.357464517	CC
NA10856	20	CG	1	cg	0.155721028	CG
NA06985	21	CG	1	cg	0.130758531	CG
NA11831	22	CG	1	cg	0.027199221	CG
NA12003.dup	23	CG	1	cg	0.056818594	nn
NA10859	24	CC	0.9555	cc	0.261558853	CC
NA07056	25	CG	1	cg	0.306040173	CG
NA07022	26	GG	1	***GG***	***0***	GG
NA12762	27		NA	CG	0.305924942	
NA12874	28	CC	0.7509	cc	0.012625059	CC
NA12003	29	CG	1	cg	0.349431491	CG
NA10835	30	GG	1	gg	0.310679098	GG
NA11882	31	CC	0.9989	cc	0.427709284	CC
NA07034	32	GG	1	gg	0.037803269	GG
NA12056	33	CG	1	cg	0.342248564	CG
NA12145	34	CG	1	cg	0.054418311	CG
NA07019	35	CG	1	cg	0.373082606	CG
NA06991	36		NA	CC	0.486711719	
NA12761	37	GG	1	gg	0.040847678	GG
NA06994	38	CG	1	cg	0.102371027	CG
NA12864	39	CC	1	cc	0.274618545	CC
NA07055	40	CC	1	cc	0.495904017	CC
NA10863	41	CC	1	cc	0.037971297	CC
NA12763	42	CG	1	cg	0.209838555	CG
NA10831	43	CG	1	cg	0.229838939	CG
NA11840	44	CC	1	cc	0.021738394	CC

	MACGT discrepancy					
	LDA discrepancy					
NA	LDA output were not g					
XY	Manual calls are given					
		**846752**				
**Coriell**	**Sample**	**LDA Call**	**LDA quality score**	**MACGT Call**	**MACGT quality score**	**HapMap Call**
NA10830	45	CG	1	cg	0.668849677	CG
NA11993.dup	46	CC	0.9286	cc	0.450897103	nn
NA12751	47	CG	1	cg	0.135141146	CG
NA12814	48	CG	1	cg	0.330138918	CG
NA10857	49	CG	1	cg	0.176919053	CG
NA07357	50	CG	1	cg	0.404788611	CG
NA07000	51	CG	1	cg	0.239003755	CG
NA12802	52	CG	1	cg	0.076349618	CG
NA10855	53	CG	1	cg	0.012425265	CG
NA11995	54		NA	CC	0.12810546	
NA10839	55	CG	1	cg	0.051976615	CG
NA10847	56	CG	1	cg	0.069004832	CG
NA12006	57	CG	1	cg	0.092528173	CG
NA12144	58	CG	1	cg	0.173760905	CG
NA11832	59	CG	1	cg	0.217295518	CG
NA12813	60	CG	1	cg	0.412230302	CG
blank	61	CC	0.993	cc	0.113038512	nn
NA12891	62	CG	1	cg	0.28235027	CG
NA12717	63		NA	CG	0.298655169	
NA10861	64	CC	0.8118	cc	0.074047456	CC
NA12004	65	CG	1	cg	0.170813762	CG
NA07048	66	CG	1	cg	0.156766531	CG
NA12801	67	CG	1	cg	0.155284636	CG
NA11830	68		NA	GG	0.28321774	
NA12057	69	CG	1	cg	0.061563096	CG
NA11994	70		NA	CG	0.418924876	
NA12740	71	CG	1	cg	0.333860177	CG
NA12005	72	GG	1	gg	0.111253266	GG
NA07345	73	CG	1	cg	0.345069793	CG
NA10854	74	CC	0.9553	cc	0.506203441	CC
NA12872	75	CC	1	cc	0.345562063	CC
NA12865	76	CG	1	cg	0.496789081	CG
NA10860	77		NA	CG	0.199903971	
NA12873	78	CG	1	cg	0.283299127	CG
NA12752	79	CG	1	cg	0.35060737	CG
NA10851	80	GG	1	gg	0.155671555	GG
NA07029	81	CG	1	cg	0.282965424	CG
NA12248	82	CG	1	cg	0.013593088	CG
NA12155	83	CC	1	cc	0.269732664	CC
NA10838	84	GG	1	gg	0.200926218	GG
NA12812	85	CG	1	cg	0.286787171	CG
NA12248.dup	86	CG	1	cg	0.31301517	nn
NA12043	87	CC	0.9201	cc	0.101545217	CC
NA12234	88	CG	1	cg	0.402058558	CG

	MACGT discrepancy					
	LDA discrepancy					
NA	LDA output were not g					
XY	Manual calls are given					
		**846752**				
**Coriell**	**Sample**	**LDA Call**	**LDA quality score**	**MACGT Call**	**MACGT quality score**	**HapMap Call**
NA12892	89	CG	1	cg	0.104091822	CG
NA10846	90	CG	1	cg	0.339908865	CG
NA06993.dup	91	CG	1	cg	0.217116385	nn
NA12249	92	GG	1	gg	0.182925855	GG
NA11881	93	CC	0.9976	cc	0.339046363	CC
NA12815	94	CC	1	cc	0.398121774	CC
NA12264	95	CC	1	cc	0.597048416	CC
NA18526	96	CC	0.9994	cc	0.497495361	CC
NA18562	97	CC	1	cc	0.480086151	CC
NA18545	98	CC	0.8212	cc	0.04107499	CC
NA18609	99	CG	0.9999	cg	0.213868573	CG
NA18566	100	CC	1	*CC*	*0*	CC
NA18621	101	CC	1	cc	0.085072785	CC
NA18577	102	CC	0.9678	cc	0.154425081	CC
NA18635	103	CC	1	cc	0.035019974	CC
NA18524	104	CC	1	cc	0.642044048	CC
NA18537	105	CC	0.9317	cc	0.061261842	CC
NA18572	106	CC	0.9602	cc	0.040695535	CC
NA18552	107	CC	0.8919	*CC*	*0*	CC
NA18563	108	CC	1	cc	0.018546981	CC
NA18594	109	CC	1	cc	0.031070329	nn
NA18624	110	CC	1	cc	0.397815051	CC
NA18592	111	CG	1	cg	0.403857998	CG
NA18529	112	CC	1	cc	0.132178205	CC
NA18603	113	CC	0.9582	cc	0.324368518	nn
NA18547	114	CG	1	cg	0.1138815	CG
NA18611	115	CC	0.7632	cc	0.009935651	CC
NA18570	116	CC	0.9403	cc	0.128223981	CC
NA18622	117	CG	1	cg	0.367137249	CG
NA18579	118	CC	0.8254	cc	0.080680944	CC
NA18636	119	CC	0.9705	cc	0.101102151	CC
NA18558	120	CC	0.9761	cc	0.476306401	CC
NA18540	121	CC	0.9532	cc	0.221064989	CC
NA18609	122	CG	1	cg	0.113901296	nn
NA18555	123	CC	0.854	cc	0.123991633	CC
NA18612	124	CC	1	cc	0.451163055	CC
NA18573	125	CC	0.9604	cc	0.340008431	CC
NA18632	126	CC	0.9477	cc	0.296025114	CC
NA18593	127	CC	0.9791	cc	0.404926432	CC
NA18532	128	CC	0.9608	cc	0.53656085	CC
NA18605	129	CC	1	cc	0.533371889	CC
NA18550	130	CC	1	cc	0.45806691	CC
NA18603	131	CC	1	cc	0.279757058	CC
NA18571	132	CG	1	cg	0.468856605	CG

	MACGT discrepancy					
	LDA discrepancy					
NA	LDA output were not g					
XY	Manual calls are given					
		**846752**				
**Coriell**	**Sample**	**LDA Call**	**LDA quality score**	**MACGT Call**	**MACGT quality score**	**HapMap Call**
NA18623	133	CG	1	cg	0.25450521	CG
NA18582	134		NA	CC	0.392214545	
NA18637	135	CC	1	cc	0.140779087	CC
NA18561	136	CC	1	cc	0.24739411	CC
NA18542	137	CC	1	cc	0.223557892	CC
NA18608	138	CC	1	cc	0.214176566	CC
NA18564	139	CC	0.9542	cc	0.223160112	CC
NA18620	140	CC	1	cc	0.433990188	CC
NA18576	141	CC	1	cc	0.203961965	CC
NA18633	142	CC	1	cc	0.250523467	CC
NA18594	143	CC	1	cc	0.440706052	CC
NA18942	144	CC	1	cc	0.47378965	CC
NA18945	145	CC	1	cc	0.411600282	CC
NA18964	146	CG	1	cg	0.44494609	CG
NA18961	147	CC	0.7503	cc	0.267952367	CC
NA18967	148	CC	1	cc	0.178640429	CC
NA18981	149	CC	0.9671	cc	0.376691378	CC
NA18994	150	CC	1	cc	0.230100522	CC
NA18998	151	CC	0.8458	cc	0.00148647	CC
NA18940	152	CC	1	cc	0.049424794	CC
NA18949	153	CC	0.8797	cc	0.059303328	CC
NA18953	154	CC	1	***CC***	***4.72E-04***	CC
NA18972	155	CC	0.8331	cc	0.001792928	CC
NA18976	156	CC	1	cc	0.214432137	CC
NA18971	157	CG	1	cg	0.203243789	CG
blank	158	NN	0.5205	***NN***	***0***	nn
NA19000	159	CC	1	cc	0.59716259	CC
NA18951	160	CC	1	cc	0.516142327	nn
NA18948	161	CC	0.9903	cc	0.326666517	CC
NA18968	162	CC	1	cc	0.217672693	CC
NA18965	163	CC	1	cc	0.092615882	CC
NA18978	164	CC	1	cc	0.185435062	CC
NA18974	165	CC	1	cc	0.311752661	CC
NA18992	166	CC	1	cc	0.100027807	CC
NA19005	167	CG	1	cg	0.432675572	CG
NA18943	168	CC	0.9731	cc	0.173076641	CC
NA18951	169	CC	0.922	cc	0.114808445	CC
NA18959	170	CC	0.9004	cc	0.458674948	CC
NA18973	171	CC	0.9202	cc	0.433312973	CC
NA18970	172	CG	1	cg	0.387327294	CG
NA18987	173	CC	0.8969	cc	0.181132681	CC
NA18995	174	CC	1	cc	0.430706748	CC
NA18999	175	CG	1	cg	0.040368646	CG
NA18947	176	CC	0.9831	cc	0.20959751	CC

	MACGT discrepancy					
	LDA discrepancy					
NA	LDA output were not g					
XY	Manual calls are given					
		**846752**				
**Coriell**	**Sample**	**LDA Call**	**LDA quality score**	**MACGT Call**	**MACGT quality score**	**HapMap Call**
NA18952	177	CC	1	cc	0.358026271	CC
NA18969	178	CC	1	cc	0.006571438	CC
NA18966	179	CC	0.8232	cc	0.184607426	CC
NA18980	180	CC	0.9091	cc	0.262964192	CC
NA18990	181	CC	0.8302	cc	0.101137703	CC
NA18997	182	CC	1	cc	0.311032233	CC
NA19007	183	CG	1	cg	0.12255151	CG
NA18944	184	CC	1	***CC***	***0***	CC
NA18956	185	CC	0.9167	cc	0.388071904	CC
NA18960	186	CC	0.9869	cc	0.531278152	CC
NA18975	187	CC	0.8718	cc	0.156413816	CC
NA18995	188	CC	1	cc	0.516561125	nn
NA18991	189	CC	0.9649	cc	0.587610325	CC
NA18996	190	CC	0.8887	cc	0.299073297	nn
NA19003	191	CC	0.9293	cc	0.329969534	CC
NA18502	192	CC	0.8494	cc	0.285734638	CC
NA19153	193	CC	0.8658	cc	0.300781288	CC
NA18857	194	CC	0.9034	cc	0.236898144	CC
NA19223	195	CC	0.8904	cc	0.140363775	CC
NA19201	196	CC	1	cc	0.109627571	CC
NA18504	197	CC	0.8395	cc	0.186977169	CC
NA18870	198	CG	1	cg	0.198113426	CG
NA18863	199	CC	0.8603	cc	0.061722332	CC
NA19145	200	CG	1	cg	0.154221506	CG
NA19137	201	CG	1	cg	0.097998634	CG
NA19238	202	CG	1	cg	0.124581807	nn
NA18500	203	CC	0.8077	***CC***	***7.35E-04***	CC
NA19144	204	CC	0.8792	***CC***	***0***	CC
NA19203	205	CC	0.7693	cc	0.142926465	CC
NA19200	206	CG	1	cg	0.100045689	CG
NA18855	207	CG	1	cg	0.320486497	CG
NA18505	208	CC	0.8344	cc	0.128562976	CC
NA19202	209	CC	1	cc	0.133971706	CC
NA18501	210	CC	0.8312	cc	0.00405989	CC
NA18861	211	CC	1	cc	0.006947925	CC
NA19193	212	CG	1	cg	0.059717148	CG
NA19143	213	CG	1	cg	0.115344925	CG
NA18517	214	CC	1	cc	0.019993017	CC
NA18862	215	CC	0.9329	cc	0.088720276	CC
NA18856	216	CC	0.9424	cc	0.167807485	nn
NA19239	217	CC	0.8721	***CC***	***0***	CC
NA19240	218	CG	1	cg	0.158859948	CG
NA18856	219	CC	1	cc	0.139597208	CC
NA18503	220	CC	1	cc	0.278278159	CC

	MACGT discrepancy					
	LDA discrepancy					
NA	LDA output were not g					
XY	Manual calls are given					
		**846752**				
**Coriell**	**Sample**	**LDA Call**	**LDA quality score**	**MACGT Call**	**MACGT quality score**	**HapMap Call**
NA18871	221	CC	0.9728	cc	0.364803002	CC
NA19221	222	CC	0.9728	cc	0.453203846	CC
NA19209	223	CC	1	cc	0.204793355	CC
NA19152	224	CC	1	cc	0.428078834	CC
NA18515	225	CC	0.9577	cc	0.265678304	CC
NA19238	226	CG	1	cg	0.285305413	CG
NA19154	227	CC	0.8826	cc	0.260558002	CC
NA19210	228	CC	0.8424	cc	0.117816385	CC
NA19211	229	CC	0.9818	cc	0.238140624	CC
NA18862	230	CC	0.9455	cc	0.316828102	nn
NA18872	231	CC	0.8979	cc	0.237316343	CC
NA19139	232	CC	0.9596	cc	0.278014895	CC
NA19222	233	CC	0.8307	cc	0.023433347	CC
NA19194	234	CG	1	cg	0.3426258	CG
NA19138	235	CC	0.9643	cc	0.030703836	CC
NA19204	236	CC	1	cc	0.328808722	CC
NA18516	237	CC	0.7792	*cc*	*9.70E-04*	CC
NA19205	238	CC	0.7518	cc	0.160371255	CC
NA19192	239	CC	1	cc	0.050724248	CC
NA18912	240	CC	0.7803	cc	0.424837758	nn
NA18508	241	CC	0.8137	cc	0.188387387	CC
NA19142	242	CC	0.7683	cc	0.014918647	CC
NA18852	243	CC	0.9728	cc	0.23757625	CC
NA18507	244	CG	1	cg	0.134007819	CG
blank	245	NN	0.7499	*NN*	*0*	nn
NA19101	246	CC	0.8244	cc	0.016973506	CC
NA19172	247	CC	0.8333	cc	0.235615176	CC
NA19160	248	CC	1	cc	0.001348354	CC
NA19129	249	CG	1	cg	0.045334939	CG
NA18913	250	CG	1	cg	0.112678878	CG
NA19120	251	CC	0.8971	cc	0.004552159	CC
NA19159	252	CC	0.7629	*cc*	*9.59E-04*	CC
NA18523	253	CC	0.9441	cc	0.370090866	CC
NA19102	254	CC	0.7669	cc	0.001669081	CC
NA19092	255	CC	1	cc	0.350201965	CC
NA18521	256	CC	1	cc	0.280521806	CC
NA18912	257	CC	0.8182	cc	0.001818966	CC
NA19206	258	CG	1	cg	0.36515702	CG
NA19128	259	CG	1	cg	0.436232139	CG
NA18859	260	CC	0.9052	cc	0.290621131	nn
NA19094	261	CC	1	cc	0.255002774	CC
NA19171	262	CG	1	cg	0.571511393	CG
NA18860	263	CC	1	cc	0.053934836	CC
NA18858	264	CC	1	cc	0.142262719	CC

	MACGT discrepancy					
	LDA discrepancy					
NA	LDA output were not g					
XY	Manual calls are given					
		**846752**				
**Coriell**	**Sample**	**LDA Call**	**LDA quality score**	**MACGT Call**	**MACGT quality score**	**HapMap Call**
NA19099	265	CC	1	cc	0.382137651	CC
NA18506	266	CG	1	cg	0.304935574	CG
NA19131	267	CG	1	cg	0.262802119	CG
NA19116	268	CG	1	cg	0.24603977	CG
NA19161	269	CC	1	cc	0.31325236	CC
NA19140	270	CC	1	cc	0.032093625	CC
NA19207	271	CC	0.8327	cc	0.015197945	CC
NA19141	272	CC	1	cc	0.075823442	CC
NA18854	273	CC	1	cc	0.193423213	CC
NA19098	274	CC	1	cc	0.120693199	CC
NA18522	275	CC	0.7596	cc	0.001471774	CC
NA18914	276	CC	1	***CC***	***0***	CC
NA18853	277	CC	0.8751	cc	0.151752128	CC
NA18859	278	CC	0.9063	cc	0.181355387	CC
NA19100	279	CC	0.7774	***CC***	***0***	CC
NA19093	280	CC	0.8612	cc	0.259642113	CC
NA19132	281	CG	1	cg	0.188622729	CG
NA19130	282	CC	0.7793	cc	0.010052266	CC
NA19208	283	CC	1	cc	0.198229682	CC
NA19127	284	CC	0.8809	cc	0.037482637	CC
NA19173	285	CC	0.843	cc	0.047491387	CC
NA19119	286	CC	1	cc	0.121135974	CC
NA19103	287	CC	0.8483	cc	0.22105602	CC
	Discrepancies	0		0		
	NNs	0		0		
	Automated Calls	276		261		
	Manual Calls	0		15		
	Concordant Calls	257		257		257

	MACGT discrepancy					
	LDA discrepancy					
NA	LDA output were not g					
XY	Manual calls are given					
		7855283				
**Coriell**	**Sample**	**LDA Call**	**LDA quality score**	**MACGT Call**	**MACGT quality score**	**HapMap Call**
NA12753	1		NA	AA	0.48759881	AA
NA12875	2	AA	0.9999	aa	0.35700322	AA
NA12044	3	AA	1	aa	0.517174567	AA
NA06993	4	GG	0.9819	gg	0.114290065	GG
NA12716	5	AA	0.9998	aa	0.237326539	AA
NA12760	6	GG	0.9953	*GG*	*0*	GG
NA07348	7	GG	0.999	gg	0.180107377	GG
NA12707	8	AA	0.9994	aa	0.213453707	AA
NA12156	9		NA	AA	0.266321921	AA
NA11992	10	AA	0.9844	aa	0.161752478	AA
NA12239	11	AA	0.821	aa	0.049284511	AA
NA12878	12	AG	0.9771	ag	0.338979827	AG
NA11993	13	AA	0.9884	aa	0.020002088	AA
NA12750	14	AA	1	aa	0.073060916	AA
NA12146	15	AA	1	aa	0.025596425	AA
NA11839	16	AG	0.9984	ag	0.186472779	AG
NA12156.dup	17	AA	0.9999	aa	0.400786926	AA
NA11829	18		NA	AA	0.470431057	AA
NA12154	19	GG	0.9998	gg	0.229444069	GG
NA10856	20	AG	0.9939	ag	0.326529416	AG
NA06985	21	AG	0.975	ag	0.228109524	AG
NA11831	22	AG	0.9982	ag	0.039793447	AG
NA12003.dup	23	AA	0.9999	aa	0.05970415	AA
NA10859	24	AA	0.9993	aa	0.16546466	AA
NA07056	25	GG	0.9997	gg	0.132948969	GG
NA07022	26	AA	0.9999	aa	0.264431923	AA
NA12762	27		NA	AA	0.370732102	AA
NA12874	28	AA	0.9946	aa	0.073392297	AA
NA12003	29	AA	0.9996	aa	0.500004598	AA
NA10835	30	GG	0.9985	gg	0.005273224	GG
NA11882	31	AA	0.9999	aa	0.407213073	AA
NA07034	32	AG	0.9819	ag	0.293293965	AG
NA12056	33	AG	0.9522	ag	0.081530896	AG
NA12145	34	AA	0.9992	aa	0.179152075	AA
NA07019	35	AG	0.9804	ag	0.491192308	AG
NA06991	36		NA	GG	0.369457754	GG
NA12761	37	GG	0.9948	gg	0.258753793	GG
NA06994	38	AG	0.9796	ag	0.395770027	AG
NA12864	39	AA	0.9991	aa	0.230144819	AA
NA07055	40	AA	1	aa	0.318689724	AA
NA10863	41	AA	0.9995	aa	0.244989353	AA
NA12763	42	AG	0.9684	ag	0.411650874	AG
NA10831	43	AG	0.9924	ag	0.331757372	AG
NA11840	44	AA	0.9701	aa	0.310362212	AA

	MACGT discrepancy					
	LDA discrepancy					
NA	LDA output were not g					
XY	Manual calls are given					
		7855283				
**Coriell**	**Sample**	**LDA Call**	**LDA quality score**	**MACGT Call**	**MACGT quality score**	**HapMap Call**
NA10830	45	AG	0.9807	ag	0.35552191	AG
NA11993.dup	46	AA	0.9987	aa	0.087269606	AA
NA12751	47	AA	1	aa	0.130324637	AA
NA12814	48	GG	0.9312	gg	0.118561125	GG
NA10857	49	AA	0.9995	aa	0.298455619	AA
NA07357	50	GG	0.9985	gg	0.494481135	GG
NA07000	51	AG	0.9843	ag	0.280818562	AG
NA12802	52	AG	0.9818	ag	0.371333741	AG
NA10855	53	AG	0.9916	ag	0.050874765	AG
NA11995	54		NA	AG	0.149791385	AG
NA10839	55	AG	0.9799	ag	0.038244646	AG
NA10847	56	AA	0.9999	aa	0.110139054	AA
NA12006	57	AG	0.9629	ag	0.173644507	AG
NA12144	58	AG	0.9659	ag	0.323113438	AG
NA11832	59	AA	0.9789	aa	0.091044081	AA
NA12813	60	AG	0.9199	ag	0.158214971	AG
blank	61	AG	0.9991	ag	0.008262308	nn
NA12891	62	AG	0.9302	ag	0.164459652	AG
NA12717	63		NA	AA	0.159908021	AA
NA10861	64	GG	0.9952	gg	0.010660733	GG
NA12004	65	GG	0.9985	gg	0.018651604	GG
NA07048	66	AA	1	aa	0.163164156	AA
NA12801	67	AA	0.9999	aa	0.046136489	AA
NA11830	68	AG	0.9173	ag	0.105449627	AG
NA12057	69	AA	1	aa	0.062917755	AA
NA11994	70		NA	GG	0.433616302	GG
NA12740	71	AA	0.9997	aa	0.293685113	AA
NA12005	72	AA	1	aa	0.021226703	AA
NA07345	73	AG	0.9909	ag	0.143854606	AG
NA10854	74	AG	0.9696	ag	0.065478016	AG
NA12872	75	AA	0.9985	aa	0.392625496	AA
NA12865	76	AA	1	aa	0.115567683	AA
NA10860	77		NA	AA	0.195947054	AA
NA12873	78	AA	0.9999	aa	0.460728102	AA
NA12752	79	GG	0.9996	gg	0.217338807	GG
NA10851	80	AG	0.983	ag	0.475984986	AG
NA07029	81	AG	0.9824	ag	0.231404667	AG
NA12248	82	AG	0.9627	ag	0.059491558	AG
NA12155	83	AG	0.9348	ag	0.077232248	AG
NA10838	84	AG	0.9413	ag	0.163951817	AG
NA12812	85	AA	0.9999	aa	0.277285868	AA
NA12248.dup	86	AG	0.9619	ag	0.070341175	AG
NA12043	87	AA	0.9988	aa	0.209309497	AA
NA12234	88	AG	0.9691	ag	0.22231022	AG

	MACGT discrepancy					
	LDA discrepancy					
NA	LDA output were not g					
XY	Manual calls are given					
		**7855283**				
**Coriell**	**Sample**	**LDA Call**	**LDA quality score**	**MACGT Call**	**MACGT quality score**	**HapMap Call**
NA12892	89	AA	1	aa	0.170144723	AA
NA10846	90	AA	1	aa	0.321602211	AA
NA06993.dup	91	GG	0.9985	gg	0.127362438	GG
NA12249	92	AG	0.9928	ag	0.128029463	AG
NA11881	93	AG	0.9888	ag	0.095522764	AG
NA12815	94	AG	0.9745	ag	0.09486608	AG
NA12264	95	AG	0.9851	ag	0.388360645	AG
NA18526	96	AA	1	aa	0.434119304	AA
NA18562	97	AA	1	aa	0.562531077	AA
NA18545	98	GG	0.9806	gg	0.073211927	GG
NA18609	99	GG	0.9845	gg	0.24755666	GG
NA18566	100	AG	0.9572	***AG***	***0***	AG
NA18621	101	AG	0.9887	ag	0.249511715	AG
NA18577	102	AG	0.9916	ag	0.154388019	AG
NA18635	103	GG	0.9936	gg	0.134363659	GG
NA18524	104	AA	1	aa	0.547492653	AA
NA18537	105	AG	0.9921	ag	0.311234031	AG
NA18572	106	AA	0.993	aa	0.154051111	AA
NA18552	107	AA	0.9697	aa	0.098792201	AA
NA18563	108	AA	1	aa	0.127144547	AA
NA18594	109	AG	0.9795	ag	0.333139408	AG
NA18624	110	GG	0.9832	gg	0.27579282	GG
NA18592	111	AG	0.9857	ag	0.661285584	AG
NA18529	112	AA	1	aa	0.496632927	AA
NA18603	113	AA	1	aa	0.412268986	AA
NA18547	114	AA	0.9971	aa	0.365154635	AA
NA18611	115	AG	0.9681	ag	0.143205743	AG
NA18570	116	AG	9303	ag	0.174744187	AG
NA18622	117	AG	0.9412	ag	0.051475257	AG
NA18579	118	GG	0.9952	gg	0.29912693	GG
NA18636	119	AA	0.9983	aa	0.167359923	AA
NA18558	120	AA	1	aa	0.493641487	AA
NA18540	121	AG	0.8817	ag	0.020684309	AG
NA18609	122	GG	0.9991	gg	0.360048041	GG
NA18555	123	AG	0.9309	ag	0.065662565	AG
NA18612	124	AA	1	aa	0.470413673	AA
NA18573	125	AA	0.9964	aa	0.339564109	AA
NA18632	126	AG	0.989	ag	0.349198507	AG
NA18593	127	GG	0.9899	gg	0.405290438	GG
NA18532	128	AA	1	aa	0.586347716	AA
NA18605	129	AA	1	aa	0.270794702	AA
NA18550	130	AA	0.9999	aa	0.501308709	AA
NA18603	131	AA	1	aa	0.294376917	AA
NA18571	132	AA	1	aa	0.624594064	AA

	MACGT discrepancy					
	LDA discrepancy					
NA	LDA output were not g					
XY	Manual calls are given					
		7855283				
**Coriell**	**Sample**	**LDA Call**	**LDA quality score**	**MACGT Call**	**MACGT quality score**	**HapMap Call**
NA18623	133	AG	0.9863	ag	0.164104708	AG
NA18582	134	AA	1	aa	0.087789182	AA
NA18637	135	AA	1	aa	0.201090589	AA
NA18561	136	AG	0.971	ag	0.133478283	AG
NA18542	137	AG	0.9611	ag	0.130890204	AG
NA18608	138	GG	0.9947	gg	0.056054021	GG
NA18564	139	AG	0.9483	ag	0.133526047	AG
NA18620	140	AA	1	aa	0.613201386	AA
NA18576	141	AA	1	aa	0.39897576	AA
NA18633	142	AG	0.9843	ag	0.242276446	AG
NA18594	143	AG	0.9647	ag	0.153834799	AG
NA18942	144	AG	0.9857	ag	0.216992083	AG
NA18945	145	AA	1	aa	0.442327657	AA
NA18964	146	AA	0.9999	aa	0.170271915	AA
NA18961	147	AG	0.9797	ag	0.414941975	AG
NA18967	148	AA	0.9999	aa	0.415055098	AA
NA18981	149	AG	0.9608	ag	0.264578741	AG
NA18994	150	AG	0.9777	ag	0.336542691	AG
NA18998	151	AG	0.9525	ag	0.155050263	AG
NA18940	152	AG	0.941	ag	0.14538521	AG
NA18949	153	AG	0.9311	ag	0.120863171	AG
NA18953	154	AA	0.9751	aa	0.022075678	AA
NA18972	155	AG	0.848	ag	0.06575271	AG
NA18976	156	AA	0.9999	aa	0.244298326	AA
NA18971	157	AG	0.9079	ag	0.133655199	AG
blank	158	NN	0.5	***NN***	***0***	
NA19000	159	GG	0.9984	gg	0.2982364	GG
NA18951	160	GG	0.9992	gg	0.177981426	GG
NA18948	161	AG	0.877	ag	0.036152629	AG
NA18968	162	AG	0.928	ag	0.101759245	AG
NA18965	163	AA	0.9936	aa	0.04601836	AA
NA18978	164	AA	0.9981	aa	0.117378409	AA
NA18974	165	AA	0.9997	aa	0.040818753	AA
NA18992	166	AA	0.9918	aa	0.011838372	AA
NA19005	167	AG	0.9792	ag	0.435450521	AG
NA18943	168	AG	0.9895	ag	0.263086638	AG
NA18951	169	GG	0.9451	gg	0.165503605	GG
NA18959	170	AG	0.9756	ag	0.329106909	AG
NA18973	171	AG	0.953	ag	0.313799791	AG
NA18970	172	AA	0.9974	aa	0.209874188	AA
NA18987	173	AG	0.9444	ag	0.132273097	AG
NA18995	174	AG	0.9661	ag	0.2659516	AG
NA18999	175	AG	0.9598	ag	0.20771295	AG
NA18947	176	AA	1	aa	0.047469261	AA

	MACGT discrepancy					
	LDA discrepancy					
NA	LDA output were not g					
XY	Manual calls are given					
		7855283				
**Coriell**	**Sample**	**LDA Call**	**LDA quality score**	**MACGT Call**	**MACGT quality score**	**HapMap Call**
NA18952	177	AA	1	aa	0.205388888	AA
NA18969	178	AG	0.9568	ag	0.199849689	AG
NA18966	179	AG	0.9476	ag	0.144979121	AG
NA18980	180	AA	1	aa	0.173023617	AA
NA18990	181	AG	0.9387	ag	0.176658447	AG
NA18997	182	AG	0.9528	ag	0.030222272	AG
NA19007	183	AA	0.9803	aa	0.115689288	AA
NA18944	184	AG	0.9725	ag	0.1921611	AG
NA18956	185	AA	0.9998	aa	0.434195125	AA
NA18960	186	AG	0.9826	ag	0.449051318	AG
NA18975	187	AG	0.9834	ag	0.448311348	AG
NA18995	188	AG	0.9807	ag	0.566363665	AG
NA18991	189	AG	0.9813	ag	0.547547975	AG
NA18996	190	AA	0.9503	aa	0.467470558	
NA19003	191	AA	0.9994	aa	0.562759292	AA
NA18502	192	AG	0.9803	ag	0.335053212	AG
NA19153	193	AG	0.9828	ag	0.192641852	AG
NA18857	194	AA	1	aa	0.422583513	AA
NA19223	195	AG	0.9207	ag	0.142207607	AG
NA19201	196	AA	1	aa	0.352642646	AA
NA18504	197	AG	0.9765	ag	0.196208388	AG
NA18870	198	AG	0.954	ag	0.294030918	AG
NA18863	199	AA	1	aa	0.308649993	AA
NA19145	200	AG	0.9653	ag	0.219842156	AG
NA19137	201	AA	0.9988	aa	0.173983274	AA
NA19238	202	AA	0.999	aa	0.184314535	AA
NA18500	203	AA	0.9918	aa	0.011254449	AA
NA19144	204	AG	0.9806	ag	0.053246873	AG
NA19203	205	AG	0.913	ag	0.031552531	AG
NA19200	206	GG	0.9832	gg	0.339887486	GG
NA18855	207	AA	1	aa	0.514256445	AA
NA18505	208	AG	0.9261	ag	0.115577015	AG
NA19202	209	AG	0.9025	ag	0.049728531	AG
NA18501	210	AA	0.9934	aa	0.04874129	AA
NA18861	211	AA	0.9996	aa	0.056173208	AA
NA19193	212	AA	0.9994	aa	0.149245607	AA
NA19143	213	AG	0.9649	ag	0.069479283	AG
NA18517	214	AG	0.8902	ag	0.068223546	AG
NA18862	215	AG	0.9155	ag	0.033740911	AG
NA18856	216	AA	1	aa	0.25584367	AA
NA19239	217	AA	0.9958	aa	0.00455813	AA
NA19240	218	AA	1	aa	0.214294476	AA
NA18856	219	AA	1	aa	0.0865209	AA
NA18503	220	AG	0.9624	ag	0.401965539	AG

	MACGT discrepancy					
	LDA discrepancy					
NA	LDA output were not g					
XY	Manual calls are given					
		7855283				
**Coriell**	**Sample**	**LDA Call**	**LDA quality score**	**MACGT Call**	**MACGT quality score**	**HapMap Call**
NA18871	221	AA	0.9995	aa	0.149982178	AA
NA19221	222	AG	0.9709	ag	0.214080756	AG
NA19209	223	AG	0.9911	ag	0.087023706	AG
NA19152	224	AA	0.9517	***AA***	***0***	AA
NA18515	225	AA	0.9873	aa	0.057545729	AA
NA19238	226	AA	0.9999	aa	0.106294616	AA
NA19154	227	AG	0.9643	ag	0.20263426	AG
NA19210	228	AG	0.9452	ag	0.152145335	AG
NA19211	229	GG	0.9916	gg	0.308002929	GG
NA18862	230	AG	0.9318	ag	0.068898774	AG
NA18872	231	AA	0.9997	aa	0.222860018	AA
NA19139	232	AA	1	aa	0.21117587	AA
NA19222	233	AA	0.9996	aa	0.140298719	AA
NA19194	234	AA	0.9988	aa	0.111787956	AA
NA19138	235	AA	0.9093	aa	0.035053769	AA
NA19204	236	AA	0.767	aa	0.0576481	AA
NA18516	237	AA	0.994	aa	0.041777304	AA
NA19205	238	AG	0.9891	ag	0.050056176	AG
NA19192	239	AA	1	aa	0.099694806	AA
NA18912	240	AA	0.9904	aa	0.236619929	AA
NA18508	241	AG	0.9803	ag	0.138027306	AG
NA19142	242	AA	0.9994	aa	0.073822876	AA
NA18852	243	AA	1	aa	0.099230449	AA
NA18507	244	AA	0.964	aa	0.047354212	AA
blank	245	NN	0.5	***NN***	***0***	
NA19101	246	AA	0.9989	aa	0.1622376	AA
NA19172	247	AA	0.9993	aa	0.109529159	AA
NA19160	248	AA	0.9855	aa	0.073282558	AA
NA19129	249	AG	0.9304	ag	0.140006296	AG
NA18913	250	AA	0.9998	aa	0.011876278	AA
NA19120	251	AA	0.8917	aa	0.005519396	AA
NA19159	252	AG	0.8961	ag	0.08068575	AG
NA18523	253	AG	0.9345	ag	0.127699526	AG
NA19102	254	AG	0.9787	ag	0.063214417	AG
NA19092	255	AA	1	aa	0.101261369	AA
NA18521	256	AG	0.9882	ag	0.116430939	AG
NA18912	257	AA	0.9778	aa	0.016349974	AA
NA19206	258	AG	0.9829	ag	0.197385591	AG
NA19128	259	AA	1	aa	0.203119623	AA
NA18859	260	AA	1	aa	0.202516717	AA
NA19094	261	AA	0.9997	aa	0.255696654	AA
NA19171	262	NN	0.75	ag	0.002697087	AG
NA18860	263	AA	1	aa	0.093861758	AA
NA18858	264	AA	1	aa	0.001902251	AA

	MACGT discrepancy					
	LDA discrepancy					
NA	LDA output were not g					
XY	Manual calls are given					
		7855283				
**Coriell**	**Sample**	**LDA Call**	**LDA quality score**	**MACGT Call**	**MACGT quality score**	**HapMap Call**
NA19099	265	AA	1	aa	0.22301721	AA
NA18506	266	AA	1	aa	0.032716977	AA
NA19131	267	AA	1	aa	0.077557713	AA
NA19116	268	AA	1	aa	0.001954442	AA
NA19161	269	AA	1	aa	0.035563196	AA
NA19140	270	AA	0.9999	aa	0.001977733	AA
NA19207	271	AA	0.9996	aa	0.058663869	AA
NA19141	272	AA	0.7654	aa	0.003318684	AA
NA18854	273	AA	1	aa	0.110921864	AA
NA19098	274	AA	1	aa	0.102503473	AA
NA18522	275	AA	1	aa	0.002659265	AA
NA18914	276	AA	0.7555	aa	0.025787187	AA
NA18853	277	AA	0.9996	aa	0.153696426	AA
NA18859	278	AA	1	aa	0.107092374	AA
NA19100	279	AA	0.9982	***AA***	***0***	AA
NA19093	280	AA	0.8625	aa	0.095456351	AA
NA19132	281	AA	1	aa	0.033452288	AA
NA19130	282	AA	0.9999	***AA***	***0***	AA
NA19208	283	AA	1	aa	0.013436549	AA
NA19127	284	AG	0.9347	ag	0.022812507	AG
NA19173	285	AA	0.9998	aa	0.179142835	AA
NA19119	286	AA	1	aa	0.147399525	AA
NA19103	287	AA	1	aa	0.021444635	AA
	Discrepancies	0		0		
	NNs	1		0		
	Automated Calls	278		271		
	Manual Calls	0		7		
	Concordant Calls					
