# Supplementary figures and images for: Robust SNP genotyping by multiplex PCR and arrayed primer extension
Source: BMC Med Genomics. 2008 Jan 31;1:5. doi: 10.1186/1755-8794-1-5 (PMC2266772; doi:10.1186/1755-8794-1-5)

## Additional file 4

TT: Sample 104 / NA18524

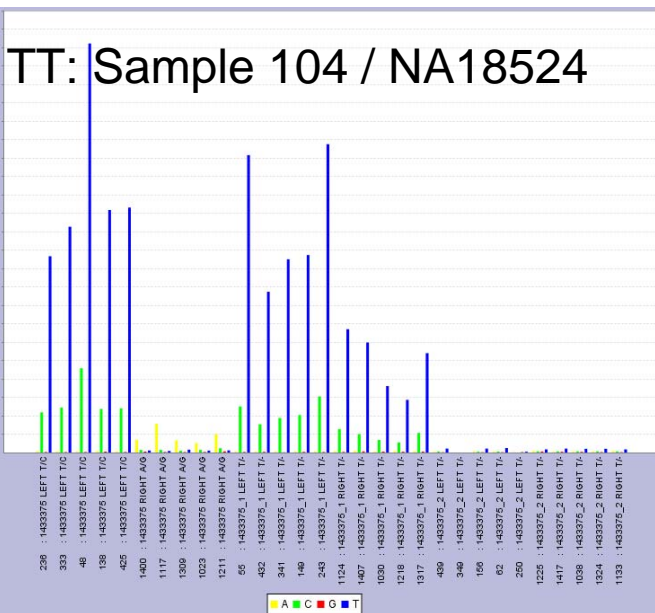

TT: Sample 125 / NA18573

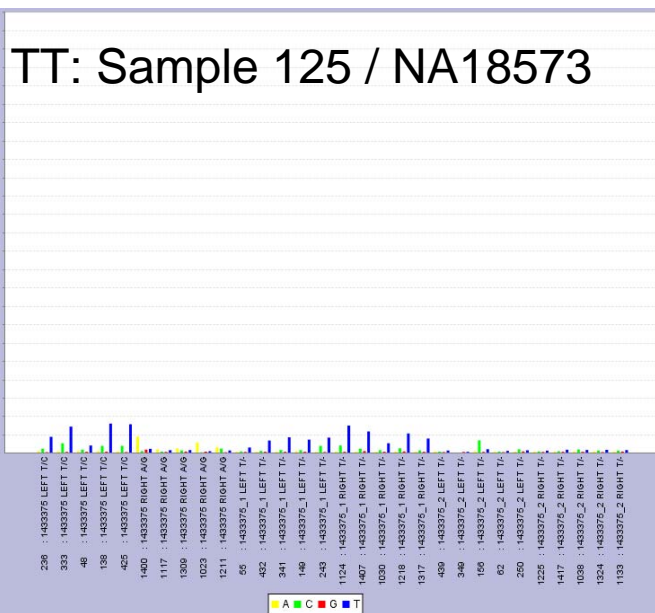

TC: Sample 148 / NA18967

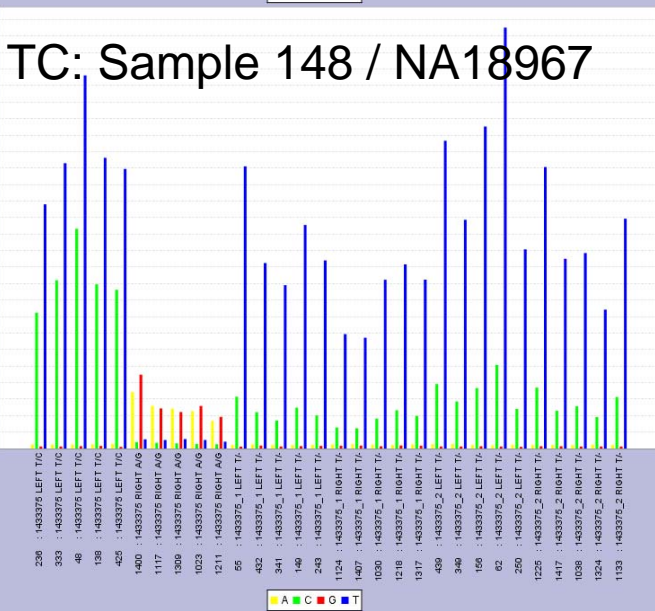

TC: Sample 128 / NA18532

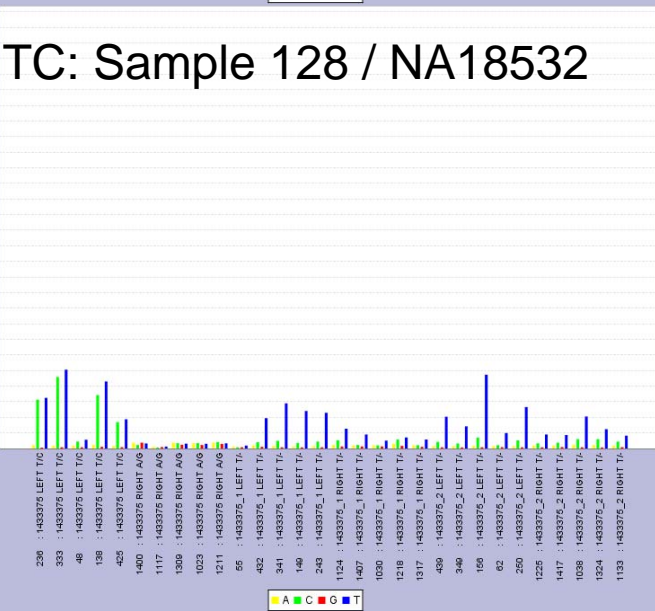

CC: Sample 67 / NA12801

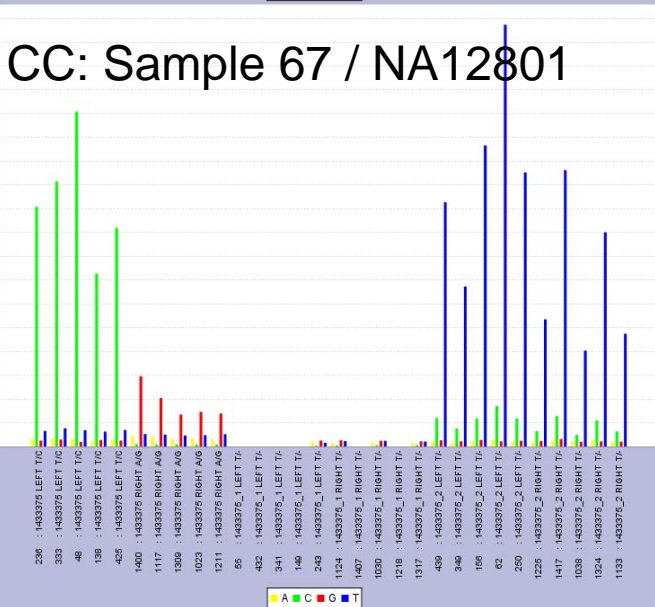

CC: Sample 126 / NA18632

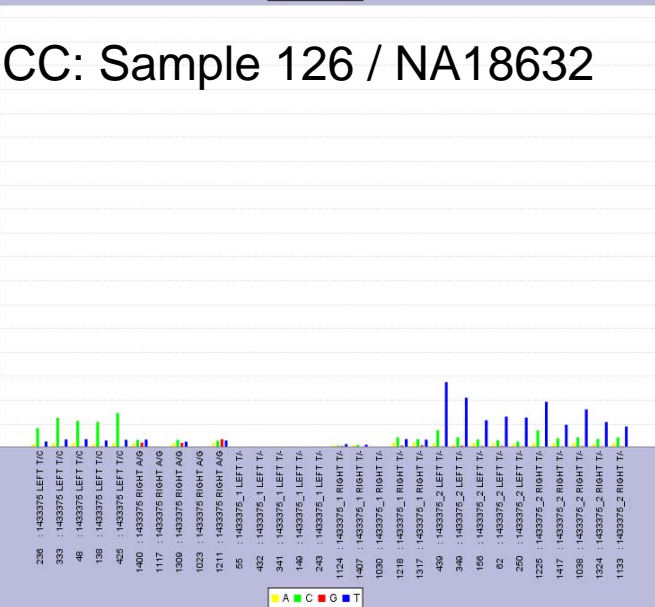

Supplement: Additional file 4 — Chart interpretation is given below. Illustrative examples for each genotype case (TT, TC, CC) are shown for the SNP rs1433375. SNP Charts on the left hand side (samples 104, 148 and 67) represent auto-called genotypes, whilst those on the right hand side (samples 125, 128 and 126) represent manually-called genotypes. The y-axes (signal intensity) of each individual genotype class have been set to identical scale values for both auto- and manually-called samples, and clearly show that genotypes can be correctly called (at least by manual inspection of the data) from samples having signal intensities up to an order of magnitude lower than usual. Each chart shows four channel fluorescent intensity data (A,C,G and T) from thirty rs1433375-specific array spots (five replicate spots for six different probes – arranged along x-axis). Starting at the left hand side of each chart, the first five spots ('LEFT T/C') refer to the left-hand APEX probe that will give either a single T (blue) signal (for homozygous TT genotypes), or a C (green) signal (for homozygous CC genotypes), or a mixture of T and C (heterozygous CT). The next five spots ('RIGHT A/G') refer to the right-hand APEX probe that interrogates the complementary DNA strand nucleotide to that of the left-hand APEX probe, hence gives a single A (yellow) signal (for TT), a single G (red) signal (for CC), or a mixed A and G signal (for TC). The remaining spots represent allele-specific APEX probes in which a base-specific fluorescence signifies the presence of the allele. '_1' probes correspond to the first allele (T in the case of rs1433375), and '_2' probes correspond to the second allele (C). The redundancy and consistency of the data across different probes give high confidence in the assigned genotypes. [file 1755-8794-1-5-S4.pdf]

## Additional file 5

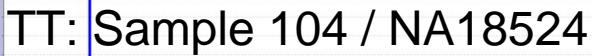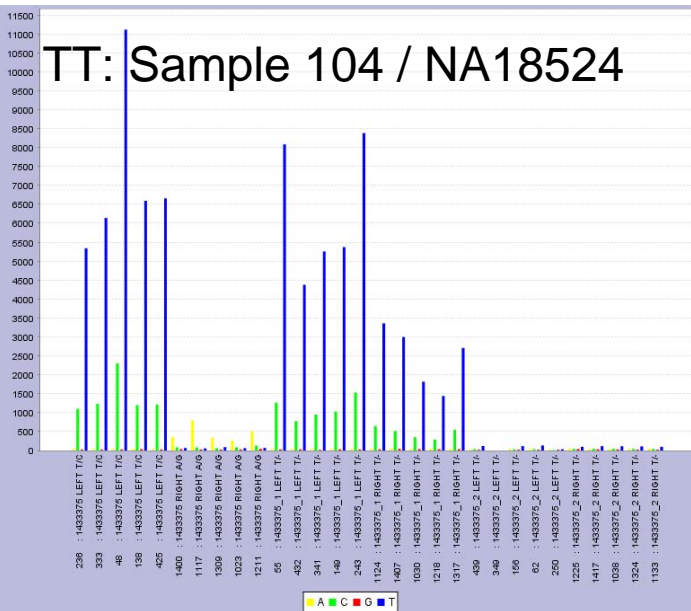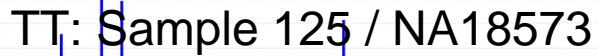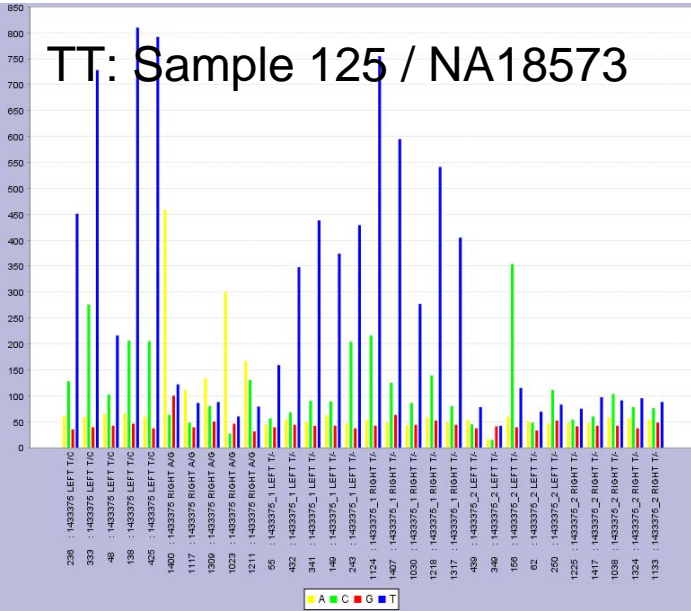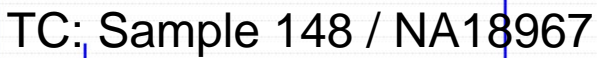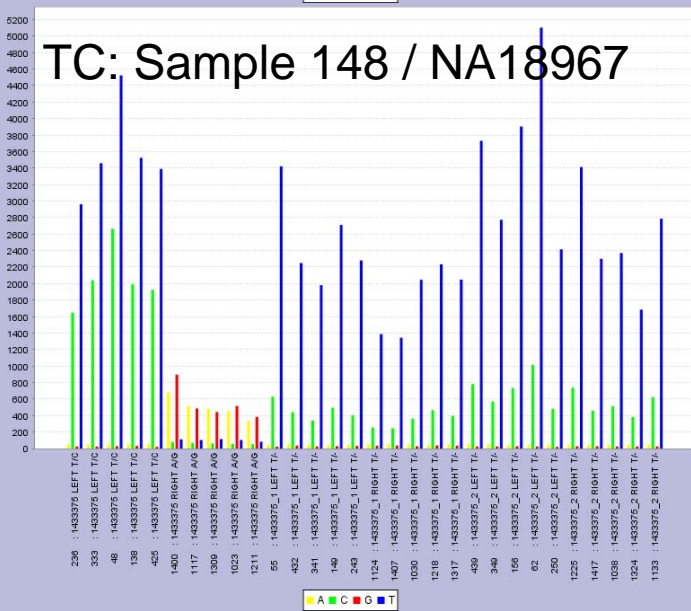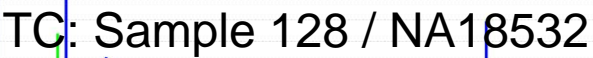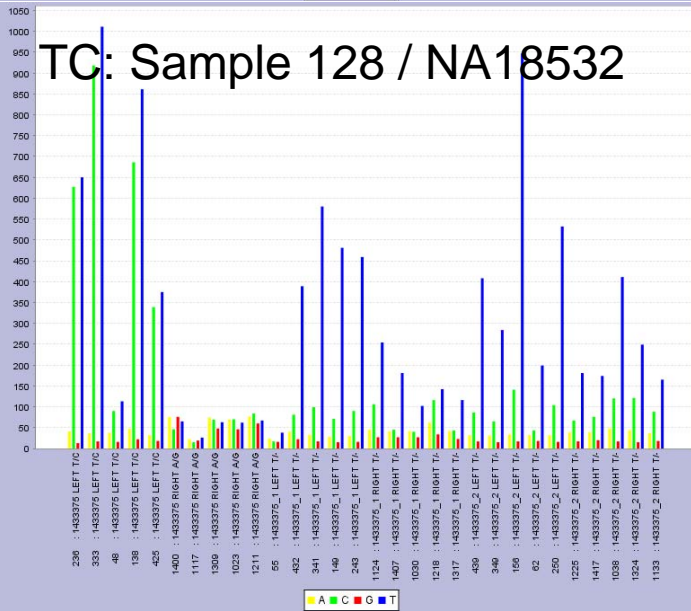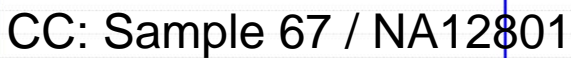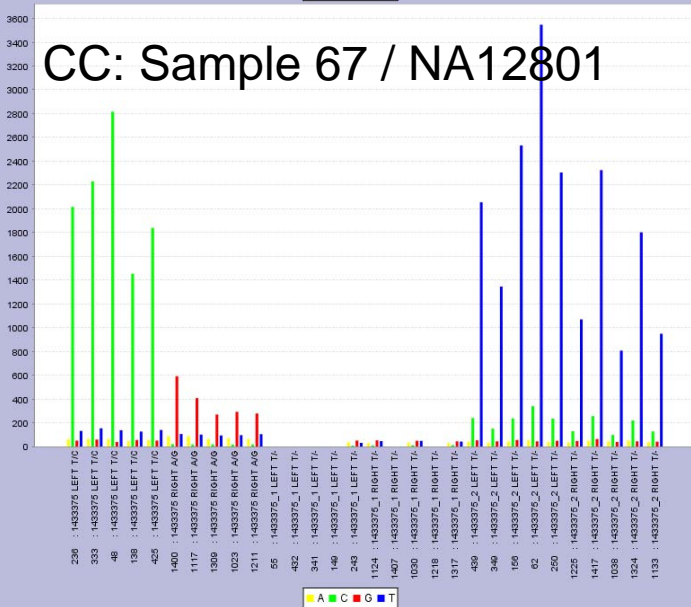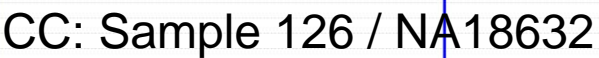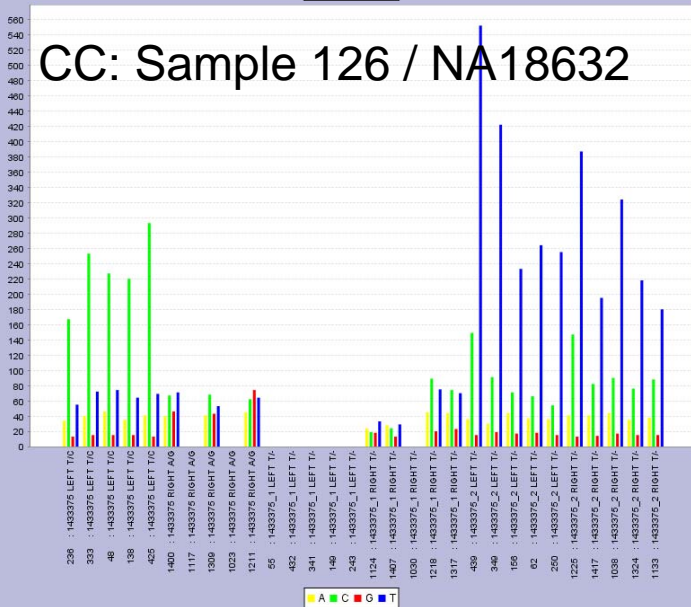

Supplement: Additional file 5 — Re-scaled SNP Charts for rs1433375. This figure is a repeat of Additional file 4, except that the y-axes of the SNP Charts on the right hand side (manually-called samples) have been adjusted to show as much of the spot intensity data as possible. Note the relative increases in the background signals, as compared to the chart data on the left hand side (auto-called samples). [file 1755-8794-1-5-S5.pdf]
