# Supplementary material for: Robust SNP genotyping by multiplex PCR and arrayed primer extension
Source: BMC Med Genomics. 2008 Jan 31;1:5. doi: 10.1186/1755-8794-1-5 (PMC2266772; doi:10.1186/1755-8794-1-5)
Supplement: Additional file 6 — List of PCR primer sequences for 50-plex PCR experiment. PCR primer sequences that were designed for the 50 HapMap SNP loci, with amplicon sizes restricted to between 100 and 200 bp, and with common 5' linkers. [file 1755-8794-1-5-S6.pdf]

No.	dbSNP_ID	Left Primer with 5' Linker (5'-3')	Right Primer with 5' Linker (5'-3')
1	rs12426585	TAC GAC TCA CTT AGG GAG GTG AAT AAC ACA TTT TAA	CGA TGT AGG TGA CAC TAG AAC TTG TGA CAG CTG TAA
2	rs12466929	TAC GAC TCA CTT AGG GAG TCA ATT CTG ATC CTG AGA	CGA TGT AGG TGA CAC TAG CGG GCA CCT CTT TAA GCA
3	rs12472674	TAC GAC TCA CTT AGG GAG GAC TGC TTG ATT TGC TAA	CGA TGT AGG TGA CAC TAG CTC AAT ATG TTA CCA CAA
4	rs12583473	TAC GAC TCA CTT AGG GAG TTG CAG GTC ACT GAT TCA	CGA TGT AGG TGA CAC TAG AAC CAG ACC AAA CAT GAA
5	rs1258464	TAC GAC TCA CTT AGG GAG CCA AGA CTT GAG GTT GAA	CGA TGT AGG TGA CAC TAG GTC AGG AAG CTG AAG AGA
6	rs1347423	TAC GAC TCA CTT AGG GAG CCT GGA GAC AGG ACC ACA	CGA TGT AGG TGA CAC TAG GCA TGA GAG GGA GAG GCA
7	rs1366660	TAC GAC TCA CTT AGG GAG TAA AAG TTC TTT AAA GTA	CGA TGT AGG TGA CAC TAG CAA ATC TCT CTC CCT CCA
8	rs1433375	TAC GAC TCA CTT AGG GAG TCA AGG ACT TGG AAG ACA	CGA TGT AGG TGA CAC TAG GTC ACT GTG ACT AGG CCA
9	rs1486048	TAC GAC TCA CTT AGG GAG AAA GAG CAG TTT CCA CAA	CGA TGT AGG TGA CAC TAG TCA TTT ACG CTA TTA GCA
10	rs1560434	TAC GAC TCA CTT AGG GAG CTT TGG GAG TGG TTT GAA	CGA TGT AGG TGA CAC TAG AAT CTC TAC CCT GAT GAA
11	rs1607185	TAC GAC TCA CTT AGG GAG GGC TTC TGA ACC AGG TAA	CGA TGT AGG TGA CAC TAG GCA GAC TGA GCC TCT TCA
12	rs1777467	TAC GAC TCA CTT AGG GAG CTC TTT TCT CAA CAG CCA	CGA TGT AGG TGA CAC TAG GCT AAG TGC ACA TCT GAA
13	rs1825443	TAC GAC TCA CTT AGG GAG AGT GTA AAG AAC GTT GTA	CGA TGT AGG TGA CAC TAG ACA TCT CTG GTT ACC GAA
14	rs1891403	TAC GAC TCA CTT AGG GAG CGT AGG AGT AAG CTG GAA	CGA TGT AGG TGA CAC TAG GGA GGC ACA GCT GTG CAA
15	rs2071748	TAC GAC TCA CTT AGG GAG TGC CAG CCT GGG GGA GCA	CGA TGT AGG TGA CAC TAG GCA TGT CGC CTC CCT GAA
16	rs2084851	TAC GAC TCA CTT AGG GAG GAT GAG TGG CAG TGT CTA	CGA TGT AGG TGA CAC TAG ATC TGA ACC AAA ATA GGA
17	rs2134180	TAC GAC TCA CTT AGG GAG ACC TGC AAC TGC CTT TCA	CGA TGT AGG TGA CAC TAG GAC CAG CAC ATG TTG GAA
18	rs2156208	TAC GAC TCA CTT AGG GAG CCT GCC CTC CTA CCT AGA	CGA TGT AGG TGA CAC TAG AGG TTT TCC CAT TGT ACA
19	rs2180289	TAC GAC TCA CTT AGG GAG ATT GAA TTG GGA AGC CAA	CGA TGT AGG TGA CAC TAG TGG GTC AAC CCA AAC CAA
20	rs2401810	TAC GAC TCA CTT AGG GAG CAT GCT ATT GCT CAA GAA	CGA TGT AGG TGA CAC TAG CCG CCT TCA AAA TAT GTA
21	rs2730648	TAC GAC TCA CTT AGG GAG GGC AAC ACC ATC CAA CAA	CGA TGT AGG TGA CAC TAG GAA CAT GAA CTC AAG ATA
22	rs273473	TAC GAC TCA CTT AGG GAG TTT AGG CAT TAC AAA CAA	CGA TGT AGG TGA CAC TAG AAG TTT TTC TAC CCA GTA
23	rs2760396	TAC GAC TCA CTT AGG GAG TTC TAG AGG ATA GGG CAA	CGA TGT AGG TGA CAC TAG CAC ACT GTC TAG CCC TCA
24	rs2803543	TAC GAC TCA CTT AGG GAG TAG GAC TGG GGG TAG GAA	CGA TGT AGG TGA CAC TAG CAT AGC CTC AGC ATT GGA
25	rs2835896	TAC GAC TCA CTT AGG GAG CAG GAC AGC CTC CCA TCA	CGA TGT AGG TGA CAC TAG TTT CTC CAA AGC TGG CCA
26	rs2840794	TAC GAC TCA CTT AGG GAG GCC TTC CAC TCT ACA GAA	CGA TGT AGG TGA CAC TAG TGA CTA TTT AAG GTA TGA
27	rs2901585	TAC GAC TCA CTT AGG GAG CCT TCA GCA TCC AGG ACA	CGA TGT AGG TGA CAC TAG TGT TTC AAA TGA GCA
28	rs2925067	TAC GAC TCA CTT AGG GAG TTC CTT AAG TCC CAG TGA	CGA TGT AGG TGA CAC TAG CCA TGA GCA ACT CCA GAA
29	rs2938675	TAC GAC TCA CTT AGG GAG TCC CCC AGC CAC TTC TAA	CGA TGT AGG TGA CAC TAG GCA CAA AGC CAG CTT TGA
30	rs318841	TAC GAC TCA CTT AGG GAG ACC TCA CTC GAC TAG CCA	CGA TGT AGG TGA CAC TAG CTG AGG GCT TGC GAT CCA
31	rs365063	TAC GAC TCA CTT AGG GAG GGC GCA CAC ACA GAG TAA	CGA TGT AGG TGA CAC TAG GCA GGC TCC TAC CAC CAA
32	rs3776720	TAC GAC TCA CTT AGG GAG CAC CAA CTC TAT GTT AGA	CGA TGT AGG TGA CAC TAG TAT TGC AGG CAG ACG TGA
33	rs3899706	TAC GAC TCA CTT AGG GAG AAA GTG AGA CAA GGA GAA	CGA TGT AGG TGA CAC TAG GTG CTG AGG CAT GTT CTA
34	rs4306755	TAC GAC TCA CTT AGG GAG ATC TCA GCT TTG TCA GAA	CGA TGT AGG TGA CAC TAG GCT CCC TGC TGT TCT CAA
35	rs4606154	TAC GAC TCA CTT AGG GAG GAT AGA ATT GGA AGT CCA	CGA TGT AGG TGA CAC TAG ATT GGA CAT CTC TGG ATA
36	rs4739199	TAC GAC TCA CTT AGG GAG AAT CCT GCA GTC CCA TGA	CGA TGT AGG TGA CAC TAG TCC ACT TCA TTA GGT GAA
37	rs4873622	TAC GAC TCA CTT AGG GAG TGG AAG GTG GTT GAG TCA	CGA TGT AGG TGA CAC TAG CCA CGT TCT TTT AAA CAA
38	rs4933826	TAC GAC TCA CTT AGG GAG AAA AGG TAA CTC AGG CAA	CGA TGT AGG TGA CAC TAG ACT CTG ACT TAG GTC
39	rs4971653	TAC GAC TCA CTT AGG GAG AGG TTT GCA TTT TTC CAA	CGA TGT AGG TGA CAC TAG CAC CAG ACT GTT TGT ACA
40	rs592069	TAC GAC TCA CTT AGG GAG TCC CTC TGC TTA GAG GAA	CGA TGT AGG TGA CAC TAG TTC ACA GAG GAA CTT TGA
41	rs6068122	TAC GAC TCA CTT AGG GAG CTC AAA ATA CAA AAA CAA	CGA TGT AGG TGA CAC TAG AAG TGT TGG AAT TAG GCA
42	rs6478813	TAC GAC TCA CTT AGG GAG CTC AGC ACC CCT TAG TGA	CGA TGT AGG TGA CAC TAG CTG TAG GTT AGA GAG GAA

43	rs667415	TAC GAC TCA CTT AGG GAG AAC ATC TGC ATC TAG CAA	CGA TGT AGG TGA CAC TAG ATC TGG TTC TAT CCA TGA
44	rs7292634	TAC GAC TCA CTT AGG GAG TAC GGC CTC TTA CGT GAA	CGA TGT AGG TGA CAC TAG TCA ATA ATA TCT CAG TAA
45	rs7555995	TAC GAC TCA CTT AGG GAG GAC AGT GGC TCT CTC CAA	CGA TGT AGG TGA CAC TAG CTG GCT GGG CAC CTG TCA
46	rs7693776	TAC GAC TCA CTT AGG GAG AAC TCC TTC CTT CTT TCA	CGA TGT AGG TGA CAC TAG TTG TTG AGG ACC GGG TAA
47	rs7855283	TAC GAC TCA CTT AGG GAG GAG GCT CAT TTT CTG TAA	CGA TGT AGG TGA CAC TAG CGG ACT CAG ATT GGG CAA
48	rs803422	TAC GAC TCA CTT AGG GAG GAG TGA AAC CAG AAG CAA	CGA TGT AGG TGA CAC TAG ACT GGA AGA TGA GCC TGA
49	rs8096868	TAC GAC TCA CTT AGG GAG TAG AGA TAG AAC ACA GAA	CGA TGT AGG TGA CAC TAG ACA AAA AGG CAT GTA TCA
50	rs846752	TAC GAC TCA CTT AGG GAG TTC TGT CCA AGA GTA GGA	CGA TGT AGG TGA CAC TAG TTT CCC CCA TGC AGC CAA
