# Supplementary material for: Robust SNP genotyping by multiplex PCR and arrayed primer extension
Source: BMC Med Genomics. 2008 Jan 31;1:5. doi: 10.1186/1755-8794-1-5 (PMC2266772; doi:10.1186/1755-8794-1-5)
Supplement: Additional file 7 — Genotyping results from second experiment (50-plex PCR). Table that lists the complete genotyping results for 49 HapMap samples and 50 SNPs. Includes LDA call and MACGT call (with quality scores), as well as original HapMap call. [file 1755-8794-1-5-S7.pdf]

MACGT discrepancy
LDA discrepancy
LDA NN (0.65 threshold)

		273473				
Coriell	Sample	HapMap Call	LDA Call	LDA quality score	MACGT Call	MACGT quality score
NA12753	1	GG	GG	1	gg	0.00178768
NA12707	8	AA	AA	1	aa	0.094809019
NA11839	16	AA	AA	1	AA	0.326934025
NA10859	24	AA	AA	1	aa	0.038765846
NA07034	32	AG	AG	1	ag	0.032212695
NA07055	40	AA	AA	1	AA	0.635116971
NA12814	48	AG	AG	1	ag	0.174654318
NA10839	55	AA	AA	1	aa	0.167396814
NA10847	56	AA	AA	1	aa	0.121217829
NA12717	63	AA	AA	1	aa	0.083709029
NA10861	64	AA	AA	0.8712	aa	0
NA12740	71	AA	AA	1	aa	0.125234947
NA12005	72	AA	AA	1	aa	0.000860207
NA12752	79	AG	AG	1	ag	0.172205946
NA10851	80	GG	GG	1	gg	0.162287684
NA12043	87	AA	AA	1	aa	0.250848764
NA12264	95	AA	AA	1	aa	0.218986893
NA18621	101	AG	AG	1	ag	0.376768499
NA18594	109	AA	AA	1	aa	0.01987388
NA18622	117	GG	GG	1	GG	0.553919244
NA18573	125	AG	AG	1	AG	0.344168115
NA18623	133	AG	AG	1	ag	0.408553418
NA18576	141	AA	AA	1	aa	0.171824468
NA18633	142	AG	AG	1	ag	0.405242572
NA18964	146	GG	GG	1	gg	0.543043764
NA18994	150	AA	AA	1	aa	0.437958957
NA18953	154	AA	AA	1	aa	0.24102754
NA18968	162	GG	GG	1	gg	0.294401433
NA18992	166	AG	AG	1	ag	0.174074334
NA18959	170	AG	AG	1	AG	0.455260826
NA18995	174	AG	AG	1	ag	0.157074187
NA18969	178	AG	AG	1	AG	0.441800071
NA18997	182	AA	AA	1	aa	0.001680909
NA18960	186	AA	AA	1	AA	0.453869141
NA18502	192	AA	AA	1	aa	0.038272907
NA18863	199	AA	AA	1	aa	0.555305574
NA19145	200	AG	AG	1	ag	0.052425501
NA18855	207	AA	AA	1	aa	0.28317106
NA18505	208	AG	AG	1	ag	0.174933417
NA18862	215	AA	AA	1	aa	0.027947411
NA18856	216	AA	AA	1	aa	0.233913929
NA18503	220	GG	GG	1	GG	0.553919244
NA19152	224	AG	AG	1	ag	0.186242285
NA19210	228	AA	AA	1	aa	0.019121583
NA19139	232	AG	AG	1	AG	0.466346559
NA19204	236	AA	AA	1	aa	0
NA18507	244	AA	AA	1	aa	0.122871237
NA19159	252	AA	AA	1	aa	0.274861246
NA18859	260	AG	AG	1	ag	0.274813626

	MACGT discrepancy
	LDA discrepancy
	LDA NN (0.65 threshold)

		318841				
**Coriell**	**Sample**	**HapMap Call**	**LDA Call**	**LDA quality score**	**MACGT Call**	**MACGT quality score**
NA12753	1	GG	GG	0.9932	gg	0.026638433
NA12707	8	CG	CG	0.9502	cg	0.057140984
NA11839	16	GG	GG	0.9917	gg	0.004030237
NA10859	24	CG	CG	0.9583	cg	0.009767181
NA07034	32	CG	CG	0.9566	cg	0
NA07055	40	CG	CG	0.9576	cg	0.233685133
NA12814	48	GG	GG	0.9791	gg	0.247941301
NA10839	55	CG	CG	0.9613	cg	0.151873063
NA10847	56	GG	GG	0.9767	GG	0.474824966
NA12717	63	GG	GG	0.9758	gg	0.295901233
NA10861	64	CC	CC	1	cc	0
NA12740	71	CG	CG	0.9356	cg	0.136424497
NA12005	72	CG	CG	0.9498	CG	0.509877301
NA12752	79	GG	GG	0.9726	gg	0.17292943
NA10851	80	CC	CC	0.9993	cc	0.21063382
NA12043	87	GG	GG	0.9593	gg	0.45088034
NA12264	95	GG	GG	0.9737	GG	0.516051459
NA18621	101	CG	CG	0.9546	cg	0.158203211
NA18594	109	CG	CG	0.9667	cg	0.261414244
NA18622	117	CC	CC	0.9998	cc	0.027341219
NA18573	125	CG	CG	0.9707	cg	0.30996435
NA18623	133	CG	CG	0.9643	cg	0.359557001
NA18576	141	GG	GG	0.9497	GG	0.532059356
NA18633	142	CG	CG	0.9597	cg	0.496918655
NA18964	146	CG	CG	0.942	cg	0.212574461
NA18994	150	CG	CG	0.9694	CG	0.587674051
NA18953	154	CG	CG	0.9407	cg	0.113094798
NA18968	162	CC	CC	0.9998	cc	0.225075827
NA18992	166	CG	CG	0.9625	cg	0.129051072
NA18959	170	CG	CG	0.9476	CG	0.466806485
NA18995	174	CG	CG	0.9664	cg	0.290324026
NA18969	178	GG	GG	0.9353	gg	0.273676369
NA18997	182	GG	GG	0.9595	gg	0.025803418
NA18960	186	CG	CG	0.9494	cg	0.277094975
NA18502	192	CG	CG	0.9761	cg	0.234591251
NA18863	199	GG	GG	0.9998	gg	0.198765136
NA19145	200	CG	CG	0.9305	cg	0.121628758
NA18855	207	CG	CG	0.9459	cg	0.204507831
NA18505	208	CG	CG	0.9517	cg	0.221287009
NA18862	215	CG	CG	0.9364	cg	0.124813234
NA18856	216	GG	GG	0.9802	GG	0.667568097
NA18503	220	CC	CC	1	CC	0.610128565
NA19152	224	CC	CC	0.9994	CC	0.592443173
NA19210	228	CC	CC	0.9997	CC	0.460589055
NA19139	232	CG	CG	0.9605	cg	0.456424664
NA19204	236	CG	CG	0.9474	cg	0.202201997
NA18507	244	CG	CG	0.9593	cg	0.159038026
NA19159	252	CG	CG	0.9512	cg	0.203368848
NA18859	260	CG	CG	0.9437	cg	0.258417896

MACGT discrepancy
LDA discrepancy
LDA NN (0.65 threshold)

		365063				
Coriell	Sample	HapMap Call	LDA Call	LDA quality score	MACGT Call	MACGT quality score
NA12753	1	AG	AG	1	ag	0
NA12707	8	AA	AA	0.9999	aa	0.011569574
NA11839	16	AG	AG	1	ag	0
NA10859	24	AA	AA	0.75	aa	0.00303723
NA07034	32	GG	GG	1	GG	0.554380002
NA07055	40	AA	AA	1	aa	0.03536874
NA12814	48	AA	AA	0.9939	aa	0.055291781
NA10839	55	AA	AA	0.9999	aa	0.094952365
NA10847	56	GG	GG	1	GG	0.554380002
NA12717	63	AA	AA	1	aa	0.022872778
NA10861	64	AA	AA	0.9879	aa	0.041297799
NA12740	71	AG	AG	1	AG	0.375097968
NA12005	72	AG	AG	1	ag	0.045561804
NA12752	79	AG	AG	0.7536	ag	0.045407598
NA10851	80	GG	GG	1	gg	0.100871845
NA12043	87	AG	AG	1	ag	0.152415122
NA12264	95	AG	AG	1	ag	0.147868029
NA18621	101	AG	AG	1	ag	0.072874202
NA18594	109	AA	AA	1	AA	0.481373744
NA18622	117	AG	AG	1	ag	0.084916604
NA18573	125	AA	AA	0.999	AA	0.472162666
NA18623	133	AA	AA	1	aa	0.358914322
NA18576	141	AA	AA	1	aa	0.25480424
NA18633	142	AG	AG	1	ag	0.10457247
NA18964	146	AG	AG	1	AG	0.50300496
NA18994	150	AA	AA	0.9489	aa	0.335694873
NA18953	154	AA	AA	1	aa	0.18413664
NA18968	162	AG	AG	0.9982	ag	0.046562552
NA18992	166	AA	AA	1	aa	0.294289234
NA18959	170	AA	AA	1	aa	0.287252783
NA18995	174	AA	AA	1	aa	0.089492655
NA18969	178	AA	AA	1	aa	0.324500813
NA18997	182	AA	AA	1	aa	0.002773095
NA18960	186	AA	AA	1	aa	0.163384134
NA18502	192	AG	AG	1	ag	0.126266454
NA18863	199	AA	AA	1	aa	0.037549854
NA19145	200	AA	AA	1	aa	0.259110703
NA18855	207	AA	AA	0.9995	aa	0.218579314
NA18505	208	AA	AA	1	AA	0.606925557
NA18862	215	AA	AA	1	aa	0.061170315
NA18856	216	AA	AA	0.9947	aa	0.099305121
NA18503	220	AA	AA	1	aa	0.290526755
NA19152	224	AG	AG	1	ag	0.113461734
NA19210	228	AG	AG	1	AG	0.411148691
NA19139	232	AA	AA	1	aa	0.353948118
NA19204	236	AA	AA	1	aa	0.237751693
NA18507	244	AA	AA	1	aa	0.111912937
NA19159	252	AG	AG	1	ag	0.078995555
NA18859	260	AA	AA	1	aa	0.043303752

MACGT discrepancy
LDA discrepancy
LDA NN (0.65 threshold)

		592069				
**Coriell**	**Sample**	**HapMap Call**	**LDA Call**	**LDA quality score**	**MACGT Call**	**MACGT quality score**
NA12753	1	CT	CT	0.999	ct	0.390666389
NA12707	8	CC	CC	1	cc	0.000625654
NA11839	16	TT	TT	0.9985	tt	0.005202209
NA10859	24	CT	CT	0.9968	ct	0.168216437
NA07034	32	CT	CT	0.9999	CT	0.231860583
NA07055	40	CT	CT	0.9996	ct	0.54321067
NA12814	48	CT	CT	0.9993	CT	0.615404516
NA10839	55	CC	CC	1	cc	0.002738028
NA10847	56	CT	CT	0.9989	ct	0.694497241
NA12717	63	CC	CC	1	cc	0.080866309
NA10861	64	CT	CT	0.7312	ct	0.146620686
NA12740	71	TT	TT	0.9989	TT	0.372200273
NA12005	72	CT	CT	0.9982	ct	0.368012851
NA12752	79	CT	CT	1	ct	0.498210478
NA10851	80	CT	CT	0.9999	ct	0.396310481
NA12043	87	CC	CC	1	CC	0.641031596
NA12264	95	CC	CC	1	CC	0.49589775
NA18621	101	CT	CT	0.9996	ct	0.580453276
NA18594	109	CC	CC	1	CC	0.493373301
NA18622	117	TT	TT	0.9993	tt	0.293173567
NA18573	125	CT	CT	0.9964	ct	0.419488051
NA18623	133	TT	TT	0.9997	tt	0.353019738
NA18576	141	CT	CT	0.9998	ct	0.497937321
NA18633	142	CT	CT	0.9997	ct	0.636606056
NA18964	146	CC	CC	1	CC	0.643144972
NA18994	150	TT	TT	0.9997	tt	0.187820203
NA18953	154	CT	CT	0.9719	ct	0.532109991
NA18968	162	CT	CT	0.9999	ct	0.409974739
NA18992	166	TT	TT	0.9999	tt	0.053399683
NA18959	170	CT	CT	1	CT	0.516179166
NA18995	174	CT	CT	0.9995	ct	0.659858142
NA18969	178	TT	TT	0.9994	tt	0.067471163
NA18997	182	TT	TT	0.9949	tt	0.051411653
NA18960	186	CT	CT	0.9985	ct	0.449727505
NA18502	192	CT	CT	0.9989	ct	0.491184711
NA18863	199	TT	TT	0.9981	TT	0.476381054
NA19145	200	TT	TT	0.9997	tt	0.18895555
NA18855	207	TT	TT	0.9996	TT	0.658378838
NA18505	208	TT	TT	0.9999	tt	0.095868486
NA18862	215	TT	TT	1	tt	0
NA18856	216	TT	TT	1	tt	0.033620022
NA18503	220	CT	CT	0.9978	CT	0.430692592
NA19152	224	CT	CT	0.9992	ct	0.527012871
NA19210	228	CT	CT	0.9998	ct	0.322785658
NA19139	232	TT	TT	0.9997	tt	0.531321218
NA19204	236	CT	CT	0.9985	ct	0.488674764
NA18507	244	TT	TT	0.9996	tt	0.407059596
NA19159	252	TT	TT	0.9999	tt	0.443749975
NA18859	260	TT	TT	0.999	TT	0.599282105

MACGT discrepancy
LDA discrepancy
LDA NN (0.65 threshold)

		667415				
**Coriell**	**Sample**	**HapMap Call**	**LDA Call**	**LDA quality score**	**MACGT Call**	**MACGT quality score**
NA12753	1	AA	AA	0.9705	aa	0.090567371
NA12707	8	AG	AG	0.9904	ag	0
NA11839	16	AG	AG	1	ag	0.132766319
NA10859	24	AG	AG	0.9967	AG	0.278997747
NA07034	32	AG	AG	0.9739	ag	0.017100288
NA07055	40	AA	AA	0.983	aa	0.347598802
NA12814	48	AA	AA	0.9821	aa	0.322991919
NA10839	55	AA	AA	0.9984	aa	0.254717108
NA10847	56	AA	AA	0.9969	aa	0.208977859
NA12717	63	AG	AG	0.9994	AG	0.487886385
NA10861	64	AA	AA	0.9999	aa	0
NA12740	71	AA	AA	0.9996	aa	0.08396334
NA12005	72	AA	AA	0.9912	aa	0.157538733
NA12752	79	AG	AG	0.9959	AG	0.482985838
NA10851	80	AG	AG	0.9738	ag	0.0642167
NA12043	87	AA	AA	0.9986	AA	0.431165575
NA12264	95	AG	AG	0.9965	ag	0.04842524
NA18621	101	AA	AA	0.9564	aa	0.346493573
NA18594	109	AA	AA	0.9924	aa	0.336881044
NA18622	117	AA	AA	0.9944	AA	0.525288336
NA18573	125	AA	AA	0.9954	aa	0.409062748
NA18623	133	AA	AA	0.9911	aa	0.43428129
NA18576	141	AA	AA	0.9962	aa	0.380229589
NA18633	142	AA	AA	0.9895	aa	0.345234571
NA18964	146	AA	AA	0.9995	AA	0.607224953
NA18994	150	AA	AA	0.9972	aa	0.327358403
NA18953	154	AA	AA	0.9999	aa	0.202192365
NA18968	162	AA	AA	0.9994	aa	0.009127665
NA18992	166	AA	AA	0.9959	aa	0.218927445
NA18959	170	AA	AA	0.9921	aa	0.244351449
NA18995	174	AA	AA	0.9893	aa	0.118102422
NA18969	178	AA	AA	0.9985	aa	0.001895405
NA18997	182	AA	AA	0.9999	aa	0
NA18960	186	AA	AA	0.9997	aa	0.338755735
NA18502	192	AA	AA	0.9992	aa	0.119090218
NA18863	199	AA	AA	0.992	AA	0.429026139
NA19145	200	AA	AA	0.999	aa	0.162290079
NA18855	207	AA	AA	0.9975	aa	0.433592136
NA18505	208	AA	AA	0.9996	aa	0.132383034
NA18862	215	AG	AG	0.8958	ag	0.013723826
NA18856	216	AA	AA	0.999	aa	0.256376993
NA18503	220	AA	AA	0.9992	aa	0.398879937
NA19152	224	AA	AA	0.9956	aa	0.377495283
NA19210	228	AA	AA	0.9972	aa	0.063518923
NA19139	232	AA	AA	0.999	aa	0.36163126
NA19204	236	AG	AG	0.9427	ag	0.005163313
NA18507	244	AG	AG	0.9962	AG	0.429371843
NA19159	252	AA	AA	0.9963	aa	0.245770078
NA18859	260	AA	AA	0.998	aa	0.368035475

Color	Legend
Red	MACGT discrepancy
Green	LDA discrepancy
Peach	LDA NN (0.65 threshold)

		803422				
**Coriell**	**Sample**	**HapMap Call**	**LDA Call**	**LDA quality score**	**MACGT Call**	**MACGT quality score**
NA12753	1	TT	TT	0.9341	TT	0.524805243
NA12707	8	CT	CT	0.9493	ct	0.060382109
NA11839	16	CT	CT	0.9689	ct	0.014239319
NA10859	24	CT	CT	0.9427	CT	0.385184304
NA07034	32	CC	CC	0.9844	cc	0
NA07055	40	CC	CC	0.9684	cc	0.067279473
NA12814	48	CC	CC	0.9661	cc	0.126157641
NA10839	55	CC	CC	0.9778	cc	0.037715929
NA10847	56	CC	CC	0.965	cc	0.077607603
NA12717	63	CC	CC	0.9659	cc	0.264863443
NA10861	64	CT	CT	0.8948	CT	0.403590647
NA12740	71	CC	CC	0.9687	cc	0.077335888
NA12005	72	CT	CT	0.9403	CT	0.490402645
NA12752	79	TT	TT	0.9523	TT	0.524805243
NA10851	80	CC	CC	0.9525	cc	0.226791446
NA12043	87	CC	CC	0.9064	cc	0.043242831
NA12264	95	CC	CC	0.9516	cc	0.223978744
NA18621	101	CC	CC	0.9522	cc	0.301816705
NA18594	109	CC	CC	0.9403	cc	0.031059045
NA18622	117	CC	CC	0.951	CC	0.439334646
NA18573	125	CC	CC	0.9566	CC	0.527600882
NA18623	133	CC	CC	0.9452	cc	0.599047736
NA18576	141	CC	CC	0.9557	CC	0.544541155
NA18633	142	CC	CC	0.949	cc	0.605810887
NA18964	146	CC	CC	0.9532	cc	0.124993661
NA18994	150	CC	CC	0.9516	cc	0.423069455
NA18953	154	CC	CC	0.9689	cc	0.000640807
NA18968	162	CC	CC	0.9479	cc	0.248156745
NA18992	166	CC	CC	0.9367	cc	0.318036442
NA18959	170	CC	CC	0.9479	CC	0.484545093
NA18995	174	CC	CC	0.9408	cc	0.057737848
NA18969	178	CC	CC	0.942	cc	0.239228929
NA18997	182	CC	CC	0.9691	cc	0.010527717
NA18960	186	CC	CC	0.9488	cc	0.232248819
NA18502	192	CC	CC	0.9655	cc	0.036483652
NA18863	199	CC	CC	0.9654	cc	0.031124788
NA19145	200	CC	CC	0.9713	cc	0.169894058
NA18855	207	CC	CC	0.9772	cc	0.2141422
NA18505	208	CC	CC	0.9578	cc	0.193004992
NA18862	215	CC	CC	0.8735	cc	0.134555323
NA18856	216	CC	CC	0.9668	cc	0.190369963
NA18503	220	CC	CC	0.9591	cc	0.024636366
NA19152	224	CC	CC	0.9629	cc	0.170508546
NA19210	228	CC	CC	0.7978	cc	0.101460508
NA19139	232	CC	CC	0.9406	cc	0.369964234
NA19204	236	CT	CT	0.8894	ct	0.005530789
NA18507	244	CC	CC	0.9685	cc	0.293343822
NA19159	252	CC	CC	0.969	cc	0.250895192
NA18859	260	CC	CC	0.9688	cc	0.176473798

MACGT discrepancy
LDA discrepancy
LDA NN (0.65 threshold)

		846752				
**Coriell**	**Sample**	**HapMap Call**	**LDA Call**	**LDA quality score**	**MACGT Call**	**MACGT quality score**
NA12753	1	CC	CC	1	cc	0.098966541
NA12707	8	GG	GG	1	GG	0.311500918
NA11839	16	CG	CG	1	cg	0.037469411
NA10859	24	CC	CC	1	CC	0.371694407
NA07034	32	GG	GG	1	GG	0.522098331
NA07055	40	CC	CC	1	CC	0.561629186
NA12814	48	CG	CG	1	CG	0.401579131
NA10839	55	CG	CG	1	CG	0.326267788
NA10847	56	CG	CG	1	cg	0.332248963
NA12717	63	CG	CG	1	cg	0.250117935
NA10861	64	CC	CC	1	cc	0.051456805
NA12740	71	CG	CG	1	cg	0.316091557
NA12005	72	GG	GG	1	GG	0.3268383
NA12752	79	CG	CG	1	CG	0.456501315
NA10851	80	GG	GG	1	GG	0.653341808
NA12043	87	CC	CC	1	cc	0.247457646
NA12264	95	CC	CC	1	cc	0.409519201
NA18621	101	CC	CC	1	cc	0.478864243
NA18594	109	CC	CC	1	CC	0.503409747
NA18622	117	CG	CG	1	CG	0.40648946
NA18573	125	CC	CC	1	cc	0.354036785
NA18623	133	CG	CG	1	cg	0.33455818
NA18576	141	CC	CC	1	cc	0.401897468
NA18633	142	CC	CC	1	cc	0.457672498
NA18964	146	CG	CG	1	cg	0.214222644
NA18994	150	CC	CC	1	cc	0.364107543
NA18953	154	CC	CC	1	cc	0.349748604
NA18968	162	CC	CC	1	cc	0.122557173
NA18992	166	CC	CC	1	cc	0.29534081
NA18959	170	CC	CC	1	cc	0.353315514
NA18995	174	CC	CC	1	CC	0.481569649
NA18969	178	CC	CC	1	cc	0.373459625
NA18997	182	CC	CC	1	cc	0.002740286
NA18960	186	CC	CC	1	cc	0.452476695
NA18502	192	CC	CC	1	cc	0.349364048
NA18863	199	CC	CC	1	cc	0.628954264
NA19145	200	CG	CG	1	cg	0.087039286
NA18855	207	CG	CG	1	cg	0.647594202
NA18505	208	CC	CC	1	cc	0.346323659
NA18862	215	CC	CC	1	cc	0.152188894
NA18856	216	CC	CC	1	cc	0.362319749
NA18503	220	CC	CC	1	cc	0.39336946
NA19152	224	CC	CC	1	cc	0.374191866
NA19210	228	CC	CC	1	cc	0.202176413
NA19139	232	CC	CC	1	cc	0.464631216
NA19204	236	CC	CC	1	cc	0.148915471
NA18507	244	CG	CG	1	cg	0.548738904
NA19159	252	CC	CC	1	cc	0.446108132
NA18859	260	CC	CC	1	cc	0.470696002

MACGT discrepancy
LDA discrepancy
LDA NN (0.65 threshold)

		1258464				
**Coriell**	**Sample**	**HapMap Call**	**LDA Call**	**LDA quality score**	**MACGT Call**	**MACGT quality score**
NA12753	1	AG	AG	0.9998	ag	0
NA12707	8	AG	AG	0.9906	ag	0
NA11839	16	AG	AG	0.9999	ag	0
NA10859	24	GG	GG	1	gg	0
NA07034	32	AG	AG	1	ag	0.001662088
NA07055	40	AA	AA	1	AA	0.459076137
NA12814	48	AA	AA	1	AA	0.552473722
NA10839	55	AA	AA	0.75	aa	0.262849061
NA10847	56	AG	AG	1	ag	0.043605182
NA12717	63	AA	AA	1	aa	0.192055034
NA10861	64	AG	AG	1	ag	0.020386435
NA12740	71	AG	AG	0.9999	ag	0.060544435
NA12005	72	AA	AA	1	aa	0.026183512
NA12752	79	AA	AA	1	aa	0.128683681
NA10851	80	GG	GG	1	gg	0.021868347
NA12043	87	AG	AG	1	ag	0.188120742
NA12264	95	AG	AG	1	AG	0.471894806
NA18621	101	AG	AG	1	ag	0.173348449
NA18594	109	GG	GG	1	gg	0.023583742
NA18622	117	GG	GG	1	gg	0.116251036
NA18573	125	GG	GG	1	GG	0.380694751
NA18623	133	GG	GG	1	GG	0.300562453
NA18576	141	AG	AG	1	AG	0.590419826
NA18633	142	GG	GG	1	gg	0.233261496
NA18964	146	AG	AG	1	ag	0.19589254
NA18994	150	AG	AG	0.9999	ag	0.142172815
NA18953	154	AG	AG	1	ag	0.069144313
NA18968	162	GG	GG	1	gg	0.079925091
NA18992	166	GG	GG	1	GG	0.358954674
NA18959	170	GG	GG	1	gg	0.160240394
NA18995	174	GG	GG	0.8224	GG	0.437902159
NA18969	178	GG	GG	1	gg	0.313431438
NA18997	182	AG	AG	1	ag	0.04295643
NA18960	186	AG	AG	1	AG	0.398251517
NA18502	192	AG	AG	1	ag	0.017461561
NA18863	199	AG	AG	1	ag	0.227631987
NA19145	200	AG	AG	1	ag	0.265794579
NA18855	207	AG	AG	1	ag	0.240871977
NA18505	208	GG	GG	1	gg	0.215848686
NA18862	215	AG	AG	1	ag	0.055154168
NA18856	216	AA	AA	0.75	aa	0.38792166
NA18503	220	GG	GG	1	gg	0.007557839
NA19152	224	AA	AA	1	AA	0.428861949
NA19210	228	AG	AG	0.9998	ag	0.023327447
NA19139	232	GG	GG	1	GG	0.397291557
NA19204	236	AG	AG	1	ag	0.001824674
NA18507	244	AG	AG	1	ag	0.136265651
NA19159	252	AA	AA	1	aa	0.179234466
NA18859	260	AG	AG	1	ag	0.027895535

MACGT discrepancy
LDA discrepancy
LDA NN (0.65 threshold)

		1347423				
**Coriell**	**Sample**	**HapMap Call**	**LDA Call**	**LDA quality score**	**MACGT Call**	**MACGT quality score**
NA12753	1	GT	GT	1	gt	0.040379079
NA12707	8	GG	GG	1	gg	0.048071782
NA11839	16	GG	GG	1	gg	0.03470957
NA10859	24	GG	GG	1	gg	0.047879963
NA07034	32	GG	GG	1	gg	0
NA07055	40	GT	GT	1	gt	0.098195034
NA12814	48	GT	GT	1	gt	0.11329473
NA10839	55	TT	TT	1	TT	0.488692675
NA10847	56	TT	TT	1	TT	0.606093446
NA12717	63	GG	GG	1	GG	0.590607032
NA10861	64	GG	GG	1	GG	0.488475799
NA12740	71	GT	GT	1	GT	0.404364583
NA12005	72	GT	GT	1	gt	0.121429571
NA12752	79	GT	GT	1	gt	0.17177644
NA10851	80	TT	TT	1	TT	0.52569394
NA12043	87	GT	GT	1	gt	0.326430386
NA12264	95	GG	GG	1	gg	0.665528707
NA18621	101	GG	GG	1	gg	0.457749669
NA18594	109	GT	GT	1	GT	0.478915764
NA18622	117	GT	GT	1	gt	0.29184947
NA18573	125	GG	GG	1	GG	0.552918427
NA18623	133	GG	GG	1	gg	0.595241599
NA18576	141	GG	GG	1	gg	0.451791574
NA18633	142	GT	GT	1	gt	0.347066374
NA18964	146	GT	GT	1	GT	0.517005151
NA18994	150	GG	GG	1	gg	0.590026557
NA18953	154	GG	GG	1	gg	0.380211012
NA18968	162	GT	GT	1	gt	0.018049147
NA18992	166	GG	GG	1	gg	0.077365649
NA18959	170	GT	GT	1	gt	0.053297044
NA18995	174	GG	GG	1	gg	0.196096176
NA18969	178	GG	GG	1	gg	0.578539763
NA18997	182	GG	GG	1	gg	0.232241159
NA18960	186	GG	GG	1	gg	0.506680174
NA18502	192	GG	GG	1	gg	0.238375042
NA18863	199	GG	GG	1	gg	0.27268827
NA19145	200	GG	GG	1	gg	0.466863301
NA18855	207	GG	GG	1	gg	0.508139251
NA18505	208	GG	GG	1	gg	0.542947244
NA18862	215	GG	GG	1	gg	0.248991175
NA18856	216	GG	GG	1	GG	0.441491795
NA18503	220	GG	GG	1	gg	0.543466535
NA19152	224	GG	GG	1	gg	0.537085203
NA19210	228	GG	GG	1	gg	0.534503968
NA19139	232	GG	GG	1	gg	0.457058878
NA19204	236	GG	GG	1	gg	0.168254631
NA18507	244	GG	GG	1	gg	0.413605533
NA19159	252	GG	GG	1	gg	0.472620357
NA18859	260	GG	GG	1	gg	0.198500965

MACGT discrepancy
LDA discrepancy
LDA NN (0.65 threshold)

		1366660				
**Coriell**	**Sample**	**HapMap Call**	**LDA Call**	**LDA quality score**	**MACGT Call**	**MACGT quality score**
NA12753	1	AG	AG	0.9094	ag	0.052578375
NA12707	8	GG	GG	1	gg	0.00533212
NA11839	16	AA	AA	0.9776	aa	0.036223054
NA10859	24	AG	AG	0.9226	ag	0.005434894
NA07034	32	AG	AG	0.9791	ag	0
NA07055	40	AG	AG	0.962	AG	0.564625477
NA12814	48	GG	GG	0.9999	GG	0.501745196
NA10839	55	AG	AG	0.9697	AG	0.480458249
NA10847	56	AG	AG	0.9641	ag	0.006282885
NA12717	63	GG	GG	0.9469	gg	0.323186602
NA10861	64	AG	AG	0.9771	ag	0.072900832
NA12740	71	AG	AG	0.9768	ag	0.283665469
NA12005	72	GG	GG	0.8969	gg	0.264348875
NA12752	79	GG	GG	0.9998	gg	0.30335615
NA10851	80	AG	AG	0.9573	ag	0.039227772
NA12043	87	AG	AG	0.9751	ag	0.29206013
NA12264	95	AG	AG	0.9728	ag	0.348426682
NA18621	101	AG	AG	0.9317	ag	0.121766752
NA18594	109	AG	AG	0.9059	ag	0.010063736
NA18622	117	AA	AA	0.9831	AA	0.501480791
NA18573	125	AG	AG	0.9877	ag	0.12692977
NA18623	133	AG	AG	0.981	ag	0.039542109
NA18576	141	GG	GG	0.9787	GG	0.549472432
NA18633	142	AG	AG	0.9847	ag	0.538784227
NA18964	146	AA	AA	0.9841	AA	0.608580344
NA18994	150	AA	AA	0.9799	aa	0.408145384
NA18953	154	AG	AG	0.9754	ag	0.067013581
NA18968	162	AG	AG	0.7988	ag	0.000583949
NA18992	166	AA	AA	0.9885	aa	0.042064595
NA18959	170	AG	AG	0.9747	AG	0.487051555
NA18995	174	AG	AG	0.9791	ag	0.080169806
NA18969	178	AG	AG	0.9827	ag	0.051538521
NA18997	182	AG	AG	0.9688	ag	0.003280875
NA18960	186	GG	GG	0.9864	gg	0.183313265
NA18502	192	AG	AG	0.9791	ag	0.129104776
NA18863	199	GG	GG	0.9999	gg	0.297048987
NA19145	200	AG	AG	0.9164	ag	0.000695027
NA18855	207	AA	AA	0.9834	AA	0.589172874
NA18505	208	GG	GG	0.8806	GG	0.566054213
NA18862	215	GG	GG	0.8824	gg	0.145345868
NA18856	216	AA	AA	0.9869	aa	0.235108076
NA18503	220	AG	AG	0.9326	ag	0.042103145
NA19152	224	AA	AA	0.98	aa	0.217696058
NA19210	228	AG	AG	0.9768	ag	0.187082586
NA19139	232	GG	GG	0.9921	gg	0.531834454
NA19204	236	AG	AG	0.9608	ag	0.10815914
NA18507	244	AG	AG	0.9343	ag	0.100956737
NA19159	252	AA	AA	0.9858	aa	0.320173186
NA18859	260	AG	AG	0.977	ag	0.232196499

MACGT discrepancy
LDA discrepancy
LDA NN (0.65 threshold)

		1433375				
**Coriell**	**Sample**	**HapMap Call**	**LDA Call**	**LDA quality score**	**MACGT Call**	**MACGT quality score**
NA12753	1	GG	GG	1	gg	0.005097956
NA12707	8	AA	AA	0.995	AA	0.577187125
NA11839	16	AG	AG	0.998	ag	0
NA10859	24	AG	AG	0.9998	ag	0.000526913
NA07034	32	AG	AG	1	ag	0.001361356
NA07055	40	AA	AA	0.8934	aa	0.555843501
NA12814	48	AG	AG	0.9084	ag	0.64563357
NA10839	55	AG	AG	1	ag	0.467355175
NA10847	56	AA	AA	0.7747	aa	0.335630837
NA12717	63	AG	AG	0.9997	ag	0.390101926
NA10861	64	AG	AG	1	ag	0.07630913
NA12740	71	AG	AG	0.9998	AG	0.325771839
NA12005	72	AA	AA	0.977	aa	0.449695574
NA12752	79	GG	GG	1	gg	0.413406308
NA10851	80	AA	AA	0.8541	aa	0.667036162
NA12043	87	AA	AA	0.9935	aa	0.498512855
NA12264	95	AG	AG	0.9999	ag	0.372729534
NA18621	101	GG	GG	1	GG	0.633236152
NA18594	109	GG	GG	1	gg	0.48751449
NA18622	117	AG	AG	0.9984	ag	0.152386057
NA18573	125	AA	AA	0.9999	AA	0.648048012
NA18623	133	AG	AG	0.959	ag	0.328385212
NA18576	141	AG	AG	0.9712	AG	0.472596789
NA18633	142	AA	AA	0.998	aa	0.423705894
NA18964	146	GG	GG	0.9997	gg	0.41295893
NA18994	150	GG	GG	1	gg	0.402957802
NA18953	154	AG	AG	1	ag	0.481694435
NA18968	162	AG	AG	0.9571	ag	0.031861379
NA18992	166	AA	AA	1	AA	0.432016686
NA18959	170	AG	AG	0.9999	AG	0.707836266
NA18995	174	AA	AA	0.9993	aa	0.441509662
NA18969	178	GG	GG	0.9981	gg	0.261303986
NA18997	182	AA	AA	0.9708	AA	0.601435219
NA18960	186	GG	GG	0.9889	gg	0.395620499
NA18502	192	GG	GG	0.9999	gg	0.32817115
NA18863	199	AG	AG	0.9997	ag	0.21414358
NA19145	200	GG	GG	1	GG	0.578335573
NA18855	207	AG	AG	0.9999	AG	0.488126155
NA18505	208	GG	GG	0.9954	gg	0.200774101
NA18862	215	AG	AG	0.9997	ag	0.417752691
NA18856	216	GG	GG	0.9999	gg	0.439782285
NA18503	220	GG	GG	0.9999	gg	0.339013411
NA19152	224	GG	GG	0.9998	gg	0.27659716
NA19210	228	GG	GG	0.9818	gg	0.152634425
NA19139	232	GG	GG	1	gg	0.567581221
NA19204	236	GG	GG	0.9495	gg	0.106438165
NA18507	244	GG	GG	1	GG	0.555587097
NA19159	252	GG	GG	1	gg	0.426477054
NA18859	260	GG	GG	1	gg	0.357881755

MACGT discrepancy
LDA discrepancy
LDA NN (0.65 threshold)

		1486048				
Coriell	Sample	HapMap Call	LDA Call	LDA quality score	MACGT Call	MACGT quality score
NA12753	1	CT	CT	0.7872	ct	0.013994585
NA12707	8	CT	CT	0.7783	ct	0.00182636
NA11839	16	CC	CC	0.9999	cc	0
NA10859	24	CC	CC	0.9993	cc	0
NA07034	32	CT	CT	0.9604	ct	0.004979463
NA07055	40	CT	CT	0.7981	ct	0.107343063
NA12814	48	CT	CT	0.8917	ct	0.277750593
NA10839	55	CC	CC	0.9971	cc	0.016282415
NA10847	56	CT	CT	0.914	ct	0.283640821
NA12717	63	TT	TT	0.9998	tt	0.075068748
NA10861	64	CC	CC	0.8736	cc	0.000971856
NA12740	71	CC	CC	0.8836	cc	0.191811699
NA12005	72	CT	CT	0.9784	ct	0.226103725
NA12752	79	TT	TT	0.9927	TT	0.428807516
NA10851	80	CT	CT	0.9225	CT	0.348644837
NA12043	87	TT	TT	0.9997	tt	0.144386695
NA12264	95	CT	CT	0.8495	ct	0.628606636
NA18621	101	CT	CT	0.8564	CT	0.39901789
NA18594	109	CT	CT	0.9505	ct	0.505695266
NA18622	117	TT	TT	0.9983	tt	0.174266709
NA18573	125	TT	TT	0.9998	TT	0.504278497
NA18623	133	CT	CT	0.8166	ct	0.436655599
NA18576	141	CT	CT	0.9384	ct	0.391641979
NA18633	142	CT	CT	0.9195	ct	0.527001611
NA18964	146	CT	CT	0.9918	ct	0.338503832
NA18994	150	CC	CC	0.8705	CC	0.511723058
NA18953	154	CC	CC	0.824	cc	0.204621272
NA18968	162	CT	CT	0.9782	ct	0.107982143
NA18992	166	CC	CC	0.9234	CC	0.470503293
NA18959	170	CC	CC	0.9745	CC	0.504494983
NA18995	174	CT	CT	0.9482	ct	0.43927745
NA18969	178	CT	CT	0.9774	CT	0.574289863
NA18997	182	CT	CT	0.9182	ct	0.024837552
NA18960	186	CT	CT	0.9864	CT	0.376651405
NA18502	192	TT	TT	0.9995	tt	0.171321588
NA18863	199	TT	TT	0.9997	tt	0.231788503
NA19145	200	TT	TT	0.9975	tt	0.115814653
NA18855	207	TT	TT	0.9995	tt	0.001835058
NA18505	208	TT	TT	0.9995	tt	0.091427368
NA18862	215	TT	TT	0.9955	tt	0.026185353
NA18856	216	TT	TT	0.9992	tt	0.357416448
NA18503	220	TT	TT	0.9977	tt	0.22304844
NA19152	224	TT	TT	0.9997	TT	0.458530028
NA19210	228	TT	TT	1	tt	0.070993746
NA19139	232	TT	TT	0.9993	tt	0.191376533
NA19204	236	TT	TT	0.9978	tt	0.115845891
NA18507	244	CT	CT	0.8823	ct	0.295074537
NA19159	252	TT	TT	0.999	tt	0.280763222
NA18859	260	TT	TT	0.9985	tt	0.328291474

MACGT discrepancy
LDA discrepancy
LDA NN (0.65 threshold)

		1560434				
Coriell	Sample	HapMap Call	LDA Call	LDA quality score	MACGT Call	MACGT quality score
NA12753	1	CT	CT	0.7574	ct	0.002525691
NA12707	8	CT	CT	0.9996	ct	0
NA11839	16	TT	TT	0.9993	tt	0
NA10859	24	TT	TT	0.9988	tt	0
NA07034	32	TT	TT	0.9858	tt	0
NA07055	40	CT	CT	0.9992	ct	0.001088036
NA12814	48	TT	TT	1	tt	0.001049823
NA10839	55	TT	TT	0.9999	tt	0.03580183
NA10847	56	TT	TT	0.9985	tt	0.147898965
NA12717	63	TT	TT	0.9993	tt	0.007682847
NA10861	64	CT	CT	0.9999	ct	0.003314864
NA12740	71	CT	CT	1	CT	0.380317854
NA12005	72	TT	TT	0.9982	tt	0.025142421
NA12752	79	TT	TT	0.9999	tt	0.033641479
NA10851	80	CT	CT	1	ct	0.022326332
NA12043	87	CT	CT	0.9993	CT	0.563705478
NA12264	95	TT	TT	0.9997	tt	0.266786557
NA18621	101	TT	TT	0.9999	tt	0.199198275
NA18594	109	TT	TT	1	tt	0.079165716
NA18622	117	TT	TT	0.9999	tt	0.028858792
NA18573	125	TT	TT	1	tt	0.003980114
NA18623	133	TT	TT	1	TT	0.350284862
NA18576	141	CT	CT	0.9947	CT	0.467769878
NA18633	142	TT	TT	1	tt	0.286021265
NA18964	146	CT	CT	0.9997	ct	0.22232272
NA18994	150	TT	TT	1	tt	0.073589021
NA18953	154	CT	CT	0.9993	ct	0.111326919
NA18968	162	TT	TT	1	tt	0.0104816
NA18992	166	CT	CT	0.9951	ct	0.109593203
NA18959	170	TT	TT	0.9999	TT	0.411697507
NA18995	174	CT	CT	0.9964	ct	0.058524275
NA18969	178	TT	TT	0.9999	TT	0.456815245
NA18997	182	TT	TT	0.9999	tt	0
NA18960	186	TT	TT	0.9999	TT	0.33206729
NA18502	192	TT	TT	0.9962	tt	0.075709757
NA18863	199	TT	TT	1	tt	0.134138647
NA19145	200	TT	TT	0.9994	tt	0.184089151
NA18855	207	TT	TT	0.9998	tt	0.046766563
NA18505	208	TT	TT	0.9998	tt	0.220865915
NA18862	215	TT	TT	0.9996	TT	0.327398749
NA18856	216	TT	TT	0.9998	tt	0.140446394
NA18503	220	TT	TT	1	tt	0.119214048
NA19152	224	TT	TT	0.9999	tt	0.135162574
NA19210	228	TT	TT	0.9998	tt	0.025255156
NA19139	232	TT	TT	1	tt	0.031155887
NA19204	236	TT	TT	0.999	tt	0.268880004
NA18507	244	TT	TT	1	tt	0.042020278
NA19159	252	TT	TT	0.9999	tt	0.067091438
NA18859	260	TT	TT	0.9998	tt	0.188032378

MACGT discrepancy
LDA discrepancy
LDA NN (0.65 threshold)

		1607185				
**Coriell**	**Sample**	**HapMap Call**	**LDA Call**	**LDA quality score**	**MACGT Call**	**MACGT quality score**
NA12753	1	TT	TT	1	tt	0.028402135
NA12707	8	TT	TT	1	tt	0.001712215
NA11839	16	TT	TT	0.9999	tt	0
NA10859	24	TT	TT	1	tt	0
NA07034	32	TT	TT	1	tt	0
NA07055	40	TT	TT	1	tt	0.104647552
NA12814	48	TT	TT	1	TT	0.270642663
NA10839	55	TT	TT	1	TT	0.3288625
NA10847	56	TT	TT	1	tt	0.18246967
NA12717	63	TT	TT	1	tt	0.198976544
NA10861	64	TT	TT	1	tt	0.000518262
NA12740	71	TT	TT	1	tt	0.089227543
NA12005	72	TT	TT	1	tt	0.218454321
NA12752	79	TT	TT	1	tt	0.013815566
NA10851	80	CT	CT	1	ct	0.063288418
NA12043	87	TT	TT	1	tt	0.29114196
NA12264	95	TT	TT	1	TT	0.500936871
NA18621	101	TT	TT	1	tt	0
NA18594	109	TT	TT	1	TT	0.547780582
NA18622	117	TT	TT	1	tt	0.225234722
NA18573	125	TT	TT	1	tt	0.068739445
NA18623	133	TT	TT	1	tt	0.447139311
NA18576	141	TT	TT	1	tt	0.250408505
NA18633	142	TT	TT	1	tt	0.560559505
NA18964	146	TT	TT	1	tt	0.319032042
NA18994	150	TT	TT	1	TT	0.312788296
NA18953	154	TT	TT	1	tt	0.274066091
NA18968	162	TT	TT	1	tt	0.049856711
NA18992	166	TT	TT	1	tt	0.405616149
NA18959	170	TT	TT	1	tt	0.390444066
NA18995	174	TT	TT	1	tt	0.307304203
NA18969	178	TT	TT	1	tt	0.356750809
NA18997	182	TT	TT	1	tt	0
NA18960	186	TT	TT	1	tt	0.13898979
NA18502	192	CT	CT	1	ct	0.093850042
NA18863	199	CC	CC	1	CC	1
NA19145	200	TT	TT	1	tt	0.246467807
NA18855	207	TT	TT	1	tt	0.241798641
NA18505	208	TT	TT	1	tt	0.294541684
NA18862	215	CT	CT	1	CT	0.591174587
NA18856	216	TT	TT	1	tt	0.310885225
NA18503	220	CT	CT	1	CT	0.390604067
NA19152	224	TT	TT	1	tt	0.407828418
NA19210	228	TT	TT	1	tt	0.053475307
NA19139	232	TT	TT	1	tt	0.354858208
NA19204	236	CT	CT	1	ct	0.002778376
NA18507	244	TT	TT	1	tt	0.126965517
NA19159	252	CT	CT	1	CT	0.399941383
NA18859	260	TT	TT	1	tt	0.288104952

MACGT discrepancy
LDA discrepancy
LDA NN (0.65 threshold)

		1777467				
**Coriell**	**Sample**	**HapMap Call**	**LDA Call**	**LDA quality score**	**MACGT Call**	**MACGT quality score**
NA12753	1	TT	TT	0.9989	tt	0.013870853
NA12707	8	TT	TT	0.9993	tt	0.019817556
NA11839	16	TT	TT	0.9991	tt	0.060495912
NA10859	24	CC	CC	1	cc	0.001512946
NA07034	32	TT	TT	0.9997	tt	0.015580535
NA07055	40	CT	CT	0.9972	CT	0.380726444
NA12814	48	TT	TT	0.9999	TT	0.583767557
NA10839	55	TT	TT	0.9999	tt	0.32025554
NA10847	56	TT	TT	0.9997	tt	0.253352349
NA12717	63	TT	TT	0.9998	tt	0.115798853
NA10861	64	TT	TT	0.9994	tt	0
NA12740	71	CC	CC	0.9701	CC	0.452028934
NA12005	72	TT	TT	0.9994	tt	0.295514855
NA12752	79	CC	CC	1	cc	0.098121456
NA10851	80	CT	CT	0.9995	ct	0.243007575
NA12043	87	CT	CT	0.9999	ct	0.539949969
NA12264	95	TT	TT	0.9995	tt	0.558947776
NA18621	101	TT	TT	0.9996	tt	0.307187115
NA18594	109	CT	CT	0.991	CT	0.507321429
NA18622	117	TT	TT	0.9995	tt	0.281593162
NA18573	125	TT	TT	0.9991	TT	0.518343053
NA18623	133	CC	CC	1	CC	0.495662178
NA18576	141	CT	CT	0.9991	ct	0.578221143
NA18633	142	CT	CT	0.9998	ct	0.552430441
NA18964	146	CT	CT	0.9997	ct	0.470282735
NA18994	150	TT	TT	0.999	TT	0.641765918
NA18953	154	TT	TT	0.9999	tt	0.125739668
NA18968	162	TT	TT	0.9994	tt	0.06514376
NA18992	166	TT	TT	0.9997	tt	0.034245152
NA18959	170	CT	CT	0.9995	CT	0.578448149
NA18995	174	CT	CT	0.9995	ct	0.454223319
NA18969	178	CT	CT	0.9996	ct	0.335316433
NA18997	182	TT	TT	0.9996	tt	0.002377984
NA18960	186	CC	CC	0.9878	cc	0.243659453
NA18502	192	CT	CT	0.9985	ct	0.009202035
NA18863	199	TT	TT	0.9995	tt	0.385851442
NA19145	200	CC	CC	0.9997	cc	0.283817072
NA18855	207	CT	CT	0.9995	ct	0.428173366
NA18505	208	CC	CC	0.9747	CC	0.428638332
NA18862	215	TT	TT	0.9994	tt	0.070094788
NA18856	216	CT	CT	0.9903	ct	0.273034951
NA18503	220	CT	CT	0.9989	ct	0.386921569
NA19152	224	CT	CT	0.9988	ct	0.468220725
NA19210	228	CT	CT	0.9998	CT	0.435322547
NA19139	232	CT	CT	0.9997	ct	0.418091804
NA19204	236	CT	CT	0.9998	ct	0.374344637
NA18507	244	CT	CT	0.9987	ct	0.333894465
NA19159	252	TT	TT	0.9997	tt	0.317929205
NA18859	260	CC	CC	1	CC	0.463529615

MACGT discrepancy
LDA discrepancy
LDA NN (0.65 threshold)

		1825443				
Coriell	Sample	HapMap Call	LDA Call	LDA quality score	MACGT Call	MACGT quality score
NA12753	1	CT	CT	0.9888	ct	0
NA12707	8	CT	CT	0.9978	ct	0
NA11839	16	CC	CC	0.9999	cc	0.004090049
NA10859	24	CC	CC	0.9999	cc	0.002049144
NA07034	32	CC	CC	0.9998	cc	0.006984486
NA07055	40	CT	CT	0.991	ct	0.003500531
NA12814	48	CC	CC	0.9997	cc	0.184151427
NA10839	55	CC	CC	0.9998	cc	0.017742599
NA10847	56	CT	CT	0.997	ct	0.106612369
NA12717	63	CC	CC	1	cc	0.080159716
NA10861	64	CC	CC	0.9999	cc	0.00244629
NA12740	71	CT	CT	0.9967	CT	0.310057517
NA12005	72	CT	CT	0.9973	ct	0.043065898
NA12752	79	CC	CC	1	cc	0.494862323
NA10851	80	CT	CT	0.9971	ct	0.03607213
NA12043	87	CT	CT	0.9985	CT	0.481862121
NA12264	95	CT	CT	0.9979	CT	0.529137741
NA18621	101	CC	CC	0.9999	cc	0.383032274
NA18594	109	CC	CC	0.9999	CC	0.402272834
NA18622	117	CC	CC	0.9998	CC	0.703644339
NA18573	125	CC	CC	0.9993	CC	0.481953756
NA18623	133	CC	CC	0.9998	cc	0.18896457
NA18576	141	CC	CC	0.9998	cc	0.270321314
NA18633	142	CC	CC	0.9998	cc	0.163613366
NA18964	146	CC	CC	0.9998	cc	0.067791991
NA18994	150	CC	CC	0.9999	cc	0.323310088
NA18953	154	CC	CC	0.9999	cc	0.097895403
NA18968	162	CC	CC	0.9998	cc	0.220344148
NA18992	166	CC	CC	0.9999	cc	0.144696262
NA18959	170	CC	CC	0.9999	cc	0.06105133
NA18995	174	CC	CC	0.9998	cc	0.100931928
NA18969	178	CC	CC	0.9998	cc	0.08289164
NA18997	182	CC	CC	0.9999	cc	0.053479178
NA18960	186	CC	CC	0.9999	cc	0.09552202
NA18502	192	CC	CC	0.9998	cc	0.049521443
NA18863	199	CC	CC	0.9998	cc	0.245171148
NA19145	200	CT	CT	0.9983	ct	0.182226099
NA18855	207	CC	CC	0.9998	cc	0.451270664
NA18505	208	CC	CC	0.9999	cc	0.028675249
NA18862	215	CC	CC	0.9999	cc	0.094749292
NA18856	216	CC	CC	0.9999	cc	0.200181987
NA18503	220	CT	CT	0.9983	ct	0.093921617
NA19152	224	CC	CC	0.9999	cc	0.18110804
NA19210	228	TT	TT	0.977	TT	1
NA19139	232	CT	CT	0.9986	CT	0.454928121
NA19204	236	CC	CC	0.9999	cc	0.008218032
NA18507	244	CC	CC	0.9999	cc	0.475128746
NA19159	252	CC	CC	0.9997	cc	0.206373699
NA18859	260	CC	CC	0.9999	cc	0.293859535

(red)	MACGT discrepancy
(green)	LDA discrepancy
(orange)	LDA NN (0.65 threshold)

		1891403				
**Coriell**	**Sample**	**HapMap Call**	**LDA Call**	**LDA quality score**	**MACGT Call**	**MACGT quality score**
NA12753	1	TT	TT	0.9012	tt	0.393026799
NA12707	8	TT	TT	0.8736	tt	0.071012033
NA11839	16	TT	TT	0.8683	tt	0.028357796
NA10859	24	TT	TT	0.8125	tt	0.133207587
NA07034	32	TT	TT	0.8005	TT	0.364685355
NA07055	40	TT	TT	0.8597	tt	0.263888929
NA12814	48	TT	TT	0.9132	TT	0.499047163
NA10839	55	TT	TT	0.8678	tt	0.390755327
NA10847	56	TT	TT	0.8792	tt	0.212416243
NA12717	63	TT	TT	0.8132	tt	0.25382794
NA10861	64	CT	CT	0.9511	ct	0
NA12740	71	TT	TT	0.7145	tt	0.302296064
NA12005	72	TT	TT	0.8645	tt	0.22667836
NA12752	79	TT	TT	0.9037	tt	0.168460229
NA10851	80	TT	TT	0.8348	tt	0.413520251
NA12043	87	TT	TT	0.591	tt	0.039485019
NA12264	95	TT	TT	0.8951	TT	0.452033777
NA18621	101	TT	TT	0.6145	tt	0.18036825
NA18594	109	TT	TT	0.8947	tt	0.210365097
NA18622	117	TT	TT	0.8538	tt	0.237239119
NA18573	125	TT	TT	0.6785	tt	0.126660609
NA18623	133	TT	TT	0.9122	tt	0.37288594
NA18576	141	CT	CT	0.9129	CT	0.456240644
NA18633	142	TT	TT	0.862	tt	0.341201102
NA18964	146	TT	TT	0.7242	tt	0.224692841
NA18994	150	TT	TT	0.6677	tt	0.299347559
NA18953	154	TT	TT	0.564	tt	0.110097274
NA18968	162	TT	TT	0.8642	tt	0.341586327
NA18992	166	TT	TT	0.6227	tt	0.014696395
NA18959	170	TT	TT	0.696	tt	0.208412126
NA18995	174	CT	CT	0.9224	CT	0.531889715
NA18969	178	TT	TT	0.8781	tt	0.092430921
NA18997	182	TT	TT	0.8621	tt	0.020826382
NA18960	186	TT	TT	0.773	tt	0.027576195
NA18502	192	TT	TT	0.6125	tt	0.204770249
NA18863	199	CT	CT	0.8929	CT	0.40467119
NA19145	200	TT	TT	0.5831	tt	0.22336753
NA18855	207	TT	TT	0.6099	tt	0.524552534
NA18505	208	TT	TT	0.6067	tt	0.285906796
NA18862	215	CT	CT	0.9524	ct	0.002873482
NA18856	216	TT	TT	0.607	tt	0.141232926
NA18503	220	TT	TT	0.5948	tt	0.180938917
NA19152	224	TT	TT	0.8299	tt	0.146121948
NA19210	228	TT	TT	0.6193	tt	0.003458443
NA19139	232	CT	CT	0.9108	ct	0.058400834
NA19204	236	CT	CT	0.8567	ct	0
NA18507	244	TT	TT	0.9126	tt	0.113311267
NA19159	252	CT	CT	0.9262	CT	0.377082663
NA18859	260	TT	TT	0.8887	tt	0

MACGT discrepancy
LDA discrepancy
LDA NN (0.65 threshold)

		2071748				
**Coriell**	**Sample**	**HapMap Call**	**LDA Call**	**LDA quality score**	**MACGT Call**	**MACGT quality score**
NA12753	1	AG	AG	0.9758	ag	0.095614514
NA12707	8	AG	AG	1	ag	0.002648016
NA11839	16	AG	AG	1	ag	0
NA10859	24	GG	GG	1	GG	0.35058132
NA07034	32	AA	AA	1	aa	0.039872635
NA07055	40	GG	GG	1	gg	0.016522326
NA12814	48	AG	AG	1	AG	0.521905644
NA10839	55	GG	GG	1	GG	0.590318167
NA10847	56	AG	AG	1	AG	0.53329413
NA12717	63	GG	GG	1	GG	0.45122126
NA10861	64	GG	GG	0.75	gg	0.012465266
NA12740	71	AA	AA	1	aa	0.238056026
NA12005	72	AG	AG	1	ag	0.004049006
NA12752	79	AA	AA	1	aa	0.216976497
NA10851	80	GG	GG	0.7501	gg	0.017739025
NA12043	87	GG	GG	0.9832	gg	0.137768806
NA12264	95	AG	AG	1	ag	0.301470665
NA18621	101	GG	GG	1	GG	0.533387577
NA18594	109	AG	AG	1	ag	0.06308587
NA18622	117	AA	AA	1	aa	0.272569823
NA18573	125	AA	AA	1	AA	0.443277667
NA18623	133	GG	GG	0.9959	gg	0.133376473
NA18576	141	AA	AA	1	aa	0.369540799
NA18633	142	AA	AA	1	AA	0.502848774
NA18964	146	AA	AA	1	aa	0.477359417
NA18994	150	AG	AG	1	ag	0.07605285
NA18953	154	AG	AG	1	ag	0.403618589
NA18968	162	AG	AG	0.9998	ag	0.181498775
NA18992	166	AG	AG	1	ag	0.042191606
NA18959	170	GG	GG	0.8597	gg	0.142363214
NA18995	174	AA	AA	0.9998	aa	0.426336009
NA18969	178	GG	GG	0.8523	gg	0.086481299
NA18997	182	AA	AA	0.9746	aa	0.001396143
NA18960	186	GG	GG	1	gg	0.375769873
NA18502	192	AA	AA	1	aa	0.215771436
NA18863	199	AA	AA	1	aa	0.270365797
NA19145	200	AG	AG	1	ag	0.150901112
NA18855	207	AG	AG	1	ag	0.189894502
NA18505	208	AG	AG	1	AG	0.530553092
NA18862	215	AA	AA	0.7845	aa	0.432598372
NA18856	216	GG	GG	1	gg	0.289955121
NA18503	220	AA	AA	1	aa	0.336720847
NA19152	224	AG	AG	1	ag	0.365648149
NA19210	228	GG	GG	0.75	gg	0.033393794
NA19139	232	AA	AA	1	AA	0.615091125
NA19204	236	AA	AA	1	aa	0.087531731
NA18507	244	AA	AA	1	aa	0.406840301
NA19159	252	AA	AA	1	aa	0.518857682
NA18859	260	AG	AG	1	ag	0.136488459

MACGT discrepancy
LDA discrepancy
LDA NN (0.65 threshold)

		2084851				
**Coriell**	**Sample**	**HapMap Call**	**LDA Call**	**LDA quality score**	**MACGT Call**	**MACGT quality score**
NA12753	1	CT	CT	0.9851	ct	0
NA12707	8	CC	CC	0.7491	cc	0
NA11839	16	CT	CT	0.8352	ct	0
NA10859	24	CT	CT	0.9949	ct	0.002225524
NA07034	32	CT	CT	0.9994	ct	0
NA07055	40	CT	CT	0.9956	ct	0.002325357
NA12814	48	CC	CC	0.9996	cc	0.3706269
NA10839	55	CT	CT	0.9996	ct	0.031364921
NA10847	56	CC	CC	0.9991	cc	0.150555792
NA12717	63	CT	CT	0.9962	ct	0.129073989
NA10861	64	TT	TT	0.7505	tt	0.003932703
NA12740	71	CT	CT	0.9996	CT	0.353095845
NA12005	72	CT	CT	0.9992	ct	0.081199205
NA12752	79	CT	CT	0.9978	ct	0.117288776
NA10851	80	CT	CT	0.9993	ct	0.18246768
NA12043	87	CT	CT	0.9981	ct	0.379828791
NA12264	95	TT	TT	0.9559	tt	0.353132732
NA18621	101	TT	TT	0.9971	TT	0.409476818
NA18594	109	CT	CT	0.8434	CT	0.482735377
NA18622	117	TT	TT	0.9989	tt	0.19239335
NA18573	125	TT	TT	0.9995	tt	0.173637121
NA18623	133	TT	TT	0.9989	tt	0.462840106
NA18576	141	TT	TT	0.9998	tt	0.377397399
NA18633	142	TT	TT	0.9992	TT	0.50812042
NA18964	146	TT	TT	0.9996	TT	0.48076103
NA18994	150	CT	CT	0.9407	ct	0.118230172
NA18953	154	TT	TT	0.7496	tt	0.454461415
NA18968	162	TT	TT	0.996	TT	0.187098036
NA18992	166	TT	TT	0.9998	TT	0.422168863
NA18959	170	CT	CT	0.9998	CT	0.502518164
NA18995	174	CC	CC	0.9999	cc	0.008607183
NA18969	178	CT	CT	0.9996	CT	0.421608802
NA18997	182	TT	TT	0.7509	tt	0
NA18960	186	TT	TT	0.8196	tt	0.398133329
NA18502	192	TT	TT	0.75	tt	0.050876546
NA18863	199	CC	CC	0.9986	cc	0.341731006
NA19145	200	CC	CC	0.9978	CC	0.487777318
NA18855	207	CC	CC	0.9998	CC	0.655466053
NA18505	208	CC	CC	0.9995	cc	0.182577064
NA18862	215	CC	CC	1	cc	0.393247429
NA18856	216	CC	CC	0.9943	cc	0.272107996
NA18503	220	CC	CC	0.9998	cc	0.179184083
NA19152	224	CC	CC	0.9998	cc	0.436968349
NA19210	228	CC	CC	0.9965	cc	0.017756143
NA19139	232	CT	CT	0.8284	ct	0.418155357
NA19204	236	CT	CT	0.9985	ct	0.12625675
NA18507	244	CT	CT	0.9755	ct	0.099638504
NA19159	252	CC	CC	0.9998	cc	0.323083836
NA18859	260	CC	CC	0.9995	CC	0.592867878

MACGT discrepancy
LDA discrepancy
LDA NN (0.65 threshold)

		2134180				
Coriell	Sample	HapMap Call	LDA Call	LDA quality score	MACGT Call	MACGT quality score
NA12753	1	AG	AG	0.9952	ag	0.013791684
NA12707	8	GG	GG	0.75	gg	0.070805866
NA11839	16	GG	GG	1	gg	0.015419958
NA10859	24	AG	AG	0.9999	ag	0.001918518
NA07034	32	AA	AA	0.9811	aa	0.002837457
NA07055	40	GG	GG	1	GG	0.38029694
NA12814	48	GG	GG	1	GG	0.543644394
NA10839	55	GG	GG	1	GG	0.616555146
NA10847	56	AA	AA	0.9998	aa	0.238053275
NA12717	63	GG	GG	1	gg	0.235468046
NA10861	64	AG	AG	1	ag	0.065470372
NA12740	71	GG	GG	1	GG	0.401673808
NA12005	72	AG	AG	1	ag	0.194731637
NA12752	79	AA	AA	1	aa	0.24677479
NA10851	80	GG	GG	1	gg	0.159509571
NA12043	87	AG	AG	1	ag	0.395197521
NA12264	95	AG	AG	1	ag	0.407774391
NA18621	101	GG	GG	1	gg	0.076651576
NA18594	109	AG	AG	1	ag	0.60920938
NA18622	117	AG	AG	1	ag	0.28442497
NA18573	125	AG	AG	0.9991	ag	0.133544091
NA18623	133	AA	AA	1	aa	0.240833742
NA18576	141	AG	AG	1	ag	0.481733313
NA18633	142	AG	AG	1	ag	0.602414812
NA18964	146	AA	AA	1	aa	0.33987078
NA18994	150	AG	AG	1	ag	0.237822642
NA18953	154	AA	AA	0.9998	aa	0.363112155
NA18968	162	AA	AA	1	aa	0.177469164
NA18992	166	AG	AG	1	ag	0.304103121
NA18959	170	AG	AG	1	AG	0.505892843
NA18995	174	AG	AG	1	ag	0.365021469
NA18969	178	AG	AG	1	ag	0.578917729
NA18997	182	AG	AG	1	AG	0.289615736
NA18960	186	AG	AG	1	ag	0.55279949
NA18502	192	AA	AA	1	aa	0.117215919
NA18863	199	AA	AA	1	aa	0.354766253
NA19145	200	AA	AA	1	aa	0.537241114
NA18855	207	AA	AA	1	aa	0.531263938
NA18505	208	AA	AA	1	aa	0.54274899
NA18862	215	AA	AA	1	AA	0.622964825
NA18856	216	AA	AA	0.9999	AA	0.610546529
NA18503	220	AG	AG	1	AG	0.564830057
NA19152	224	AA	AA	1	aa	0.064530119
NA19210	228	AA	AA	1	aa	0.217413507
NA19139	232	AA	AA	1	AA	0.611105769
NA19204	236	AA	AA	0.9996	aa	0.051157406
NA18507	244	AA	AA	1	aa	0.125061885
NA19159	252	AA	AA	1	AA	0.578760287
NA18859	260	AA	AA	1	aa	0.510672113

MACGT discrepancy
LDA discrepancy
LDA NN (0.65 threshold)

		2156208				
Coriell	Sample	HapMap Call	LDA Call	LDA quality score	MACGT Call	MACGT quality score
NA12753	1	TT	TT	1	tt	0.020965768
NA12707	8	TT	TT	0.9999	tt	0.026576613
NA11839	16	CT	CT	0.9998	CT	0.298404721
NA10859	24	CC	CC	0.7533	cc	0.004617076
NA07034	32	CC	CC	1	cc	0.005760758
NA07055	40	CT	CT	1	CT	0.583336444
NA12814	48	TT	TT	1	tt	0.111502636
NA10839	55	CC	CC	1	cc	0.144382674
NA10847	56	CT	CT	1	CT	0.680633311
NA12717	63	TT	TT	1	tt	0.237787431
NA10861	64	CT	CT	1	ct	0.081968707
NA12740	71	CC	CC	1	CC	0.567465845
NA12005	72	CC	CC	1	cc	0.045945773
NA12752	79	CT	CT	1	ct	0.553250804
NA10851	80	CC	CC	1	cc	0.397907102
NA12043	87	CC	CC	1	cc	0.243271191
NA12264	95	TT	TT	1	tt	0.286016674
NA18621	101	TT	TT	1	tt	0.302307009
NA18594	109	TT	TT	1	TT	0.550717325
NA18622	117	TT	TT	1	TT	0.602280416
NA18573	125	TT	TT	1	tt	0.201054633
NA18623	133	TT	TT	1	tt	0.322646041
NA18576	141	TT	TT	1	tt	0.220244153
NA18633	142	TT	TT	1	tt	0.419449096
NA18964	146	TT	TT	1	tt	0.277536882
NA18994	150	TT	TT	1	TT	0.572306331
NA18953	154	TT	TT	1	tt	0.472526868
NA18968	162	CT	CT	0.9991	ct	0.295536885
NA18992	166	TT	TT	1	tt	0.433340639
NA18959	170	TT	TT	1	tt	0.435845046
NA18995	174	TT	TT	1	tt	0.474321623
NA18969	178	TT	TT	0.999	tt	0.385473817
NA18997	182	TT	TT	1	tt	0.005545967
NA18960	186	TT	TT	1	TT	0.542679213
NA18502	192	TT	TT	1	tt	0.14433973
NA18863	199	CC	CC	1	cc	0.172452388
NA19145	200	CT	CT	1	ct	0.173790068
NA18855	207	CT	CT	1	ct	0.660220477
NA18505	208	CT	CT	1	ct	0.349838148
NA18862	215	CC	CC	1	cc	0.416602613
NA18856	216	CT	CT	1	ct	0.230009237
NA18503	220	CC	CC	1	CC	0.517023466
NA19152	224	CC	CC	1	CC	0.621360863
NA19210	228	CC	CC	0.9987	cc	0.027515828
NA19139	232	CT	CT	0.9981	ct	0.317607935
NA19204	236	CT	CT	1	CT	0.385640185
NA18507	244	CC	CC	1	CC	0.566294429
NA19159	252	CC	CC	1	cc	0.687109073
NA18859	260	CT	CT	1	ct	0.702142464

MACGT discrepancy
LDA discrepancy
LDA NN (0.65 threshold)

		2180289				
**Coriell**	**Sample**	**HapMap Call**	**LDA Call**	**LDA quality score**	**MACGT Call**	**MACGT quality score**
NA12753	1	GG	GG	1	gg	0.043944303
NA12707	8	CC	CC	0.9999	cc	0.004684966
NA11839	16	CC	CC	1	cc	0.000959109
NA10859	24	CC	CC	0.9999	cc	0.008061213
NA07034	32	CG	CG	0.9684	cg	0.000905117
NA07055	40	CC	CC	0.9996	cc	0.045887878
NA12814	48	CC	CC	0.9991	cc	0.103280103
NA10839	55	CG	CG	0.9935	cg	0.084155964
NA10847	56	CC	CC	0.9951	cc	0.231114554
NA12717	63	CC	CC	1	cc	0.258936189
NA10861	64	CC	CC	0.9455	cc	0.077982255
NA12740	71	CG	CG	0.9934	cg	0.223095609
NA12005	72	CG	CG	0.9982	CG	0.387297857
NA12752	79	CG	CG	0.9844	cg	0.026791688
NA10851	80	CG	CG	0.9953	cg	0.086307453
NA12043	87	CC	CC	0.997	cc	0.488551405
NA12264	95	CC	CC	0.9993	cc	0.437557051
NA18621	101	GG	GG	1	GG	0.618585715
NA18594	109	CC	CC	0.9998	CC	0.472006801
NA18622	117	CC	CC	0.9999	cc	0.18187004
NA18573	125	CG	CG	0.7961	cg	0.021822011
NA18623	133	CC	CC	0.9999	cc	0.507166019
NA18576	141	CG	CG	0.9891	CG	0.449605221
NA18633	142	CG	CG	0.9961	cg	0.329830784
NA18964	146	CC	CC	0.9987	cc	0.372791215
NA18994	150	CC	CC	1	cc	0.214067316
NA18953	154	CG	CG	0.992	cg	0.176285125
NA18968	162	CC	CC	0.9995	cc	0.207320212
NA18992	166	GG	GG	1	GG	0.600966628
NA18959	170	CC	CC	0.9925	CC	0.533754095
NA18995	174	CC	CC	0.9996	cc	0
NA18969	178	CC	CC	0.9978	CC	0.691975539
NA18997	182	CC	CC	0.9547	cc	0.016705791
NA18960	186	CC	CC	0.9979	cc	0.373559626
NA18502	192	CC	CC	0.9055	cc	0.134016556
NA18863	199	CC	CC	0.9992	cc	0.277027996
NA19145	200	CC	CC	0.9943	CC	0.42334361
NA18855	207	CC	CC	0.9999	cc	0.310643339
NA18505	208	CC	CC	0.9995	cc	0.498261944
NA18862	215	CC	CC	0.9995	cc	0.376075791
NA18856	216	CC	CC	0.9906	cc	0.397474519
NA18503	220	CC	CC	0.9994	cc	0.334023679
NA19152	224	CG	CG	0.9986	CG	0.527234307
NA19210	228	CG	CG	0.984	cg	0.180824529
NA19139	232	GG	GG	1	GG	0.548338818
NA19204	236	CC	CC	0.9557	cc	0.16062363
NA18507	244	CC	CC	0.9987	cc	0.377152399
NA19159	252	GG	GG	1	gg	0.458599315
NA18859	260	CG	CG	0.9977	cg	0.208459863

MACGT discrepancy
LDA discrepancy
LDA NN (0.65 threshold)

		2401810				
Coriell	Sample	HapMap Call	LDA Call	LDA quality score	MACGT Call	MACGT quality score
NA12753	1	AG	AG	0.7492	ag	0.142240185
NA12707	8	AA	AA	0.9991	aa	0.181722321
NA11839	16	AA	AA	0.9456	aa	0.112609276
NA10859	24	AA	AA	0.8559	aa	0.041435017
NA07034	32	AA	AA	0.9994	aa	0.193199368
NA07055	40	GG	GG	0.7561	gg	0
NA12814	48	AA	AA	0.9999	aa	0.538180884
NA10839	55	AG	AG	0.7499	AG	0.464250697
NA10847	56	GG	GG	0.9944	GG	0.527937183
NA12717	63	AA	AA	0.9966	aa	0.256758093
NA10861	64	AG	AG	0.7498	ag	0.0524788
NA12740	71	AA	AA	0.9999	aa	0.311034232
NA12005	72	GG	GG	0.7577	gg	0.069396534
NA12752	79	GG	GG	0.8841	GG	0.40368213
NA10851	80	AG	AG	0.75	ag	0.18662496
NA12043	87	AA	AA	0.9999	aa	0.455830392
NA12264	95	GG	GG	0.7588	gg	0.212471396
NA18621	101	AA	AA	1	aa	0.375926917
NA18594	109	AG	AG	0.75	AG	0.435381266
NA18622	117	AA	AA	0.9999	AA	0.54584817
NA18573	125	AA	AA	0.997	aa	0.257019051
NA18623	133	AA	AA	0.9997	aa	0.390836283
NA18576	141	AG	AG	0.7497	ag	0.319037585
NA18633	142	AA	AA	1	AA	0.568480525
NA18964	146	AA	AA	0.9999	AA	0.579560802
NA18994	150	AA	AA	0.9995	aa	0.436158126
NA18953	154	AA	AA	0.9997	aa	0.677271791
NA18968	162	AA	AA	0.9994	aa	0.29184175
NA18992	166	AA	AA	0.9999	aa	0.566211905
NA18959	170	AG	AG	0.7492	AG	0.521590163
NA18995	174	AA	AA	1	aa	0.66326052
NA18969	178	AG	AG	0.7495	ag	0.525563445
NA18997	182	AA	AA	1	aa	0.041826809
NA18960	186	AG	AG	0.7499	AG	0.454535431
NA18502	192	AG	AG	0.75	ag	0.273243705
NA18863	199	AA	AA	1	aa	0.677083532
NA19145	200	AA	AA	0.9988	aa	0.23135043
NA18855	207	AA	AA	1	aa	0.523639268
NA18505	208	GG	GG	0.7625	gg	0.057918774
NA18862	215	AG	AG	0.75	ag	0.019094842
NA18856	216	AA	AA	0.9999	aa	0.354904237
NA18503	220	GG	GG	0.7526	gg	0.307406207
NA19152	224	AG	AG	0.7496	ag	0.469657886
NA19210	228	AA	AA	0.9999	AA	0.495342209
NA19139	232	AA	AA	0.9991	aa	0.414436781
NA19204	236	AA	AA	1	aa	0.225609638
NA18507	244	AA	AA	1	aa	0.526079125
NA19159	252	AA	AA	1	aa	0.403559538
NA18859	260	GG	GG	0.9107	GG	0.598083522

MACGT discrepancy
LDA discrepancy
LDA NN (0.65 threshold)

		2730648				
**Coriell**	**Sample**	**HapMap Call**	**LDA Call**	**LDA quality score**	**MACGT Call**	**MACGT quality score**
NA12753	1	AG	AG	0.8942	ag	0.093251728
NA12707	8	AG	AG	0.8696	ag	0.002332808
NA11839	16	AG	AG	0.9087	ag	0.025829082
NA10859	24	GG	GG	0.8106	gg	0
NA07034	32	AA	AA	1	aa	0.007317334
NA07055	40	AA	AA	1	aa	0.09026196
NA12814	48	AG	AG	0.9115	AG	0.514067143
NA10839	55	AA	AA	1	AA	0.446022238
NA10847	56	AG	AG	0.9218	ag	0.311880093
NA12717	63	AG	AG	0.9122	ag	0.144105108
NA10861	64	AG	AG	0.9141	ag	0
NA12740	71	AA	AA	1	aa	0.344345832
NA12005	72	AA	AA	1	AA	0.533900395
NA12752	79	AA	AA	1	aa	0.126664416
NA10851	80	AA	AA	1	aa	0.221858387
NA12043	87	AG	AG	0.9247	ag	0.311784235
NA12264	95	AA	AA	1	aa	0.033646717
NA18621	101	GG	GG	0.9222	GG	0.522762953
NA18594	109	GG	GG	0.9019	GG	0.513935054
NA18622	117	GG	GG	0.9205	gg	0.49129252
NA18573	125	AG	AG	0.9162	ag	0.246227649
NA18623	133	GG	GG	0.9152	GG	0.537949645
NA18576	141	AA	AA	1	AA	0.544809127
NA18633	142	AG	AG	0.9205	AG	0.53086042
NA18964	146	AG	AG	0.9166	AG	0.407640932
NA18994	150	AG	AG	0.9291	ag	0.210626153
NA18953	154	AA	AA	1	aa	0.34106753
NA18968	162	AA	AA	1	aa	0.048582352
NA18992	166	AG	AG	0.9156	ag	0.46235669
NA18959	170	AG	AG	0.9211	AG	0.482631763
NA18995	174	AG	AG	0.9182	ag	0.36940778
NA18969	178	AA	AA	1	aa	0.349232612
NA18997	182	AG	AG	0.8852	ag	0.002709508
NA18960	186	GG	GG	0.9146	GG	0.515403855
NA18502	192	AG	AG	0.921	ag	0.172938955
NA18863	199	AA	AA	1	aa	0.073998487
NA19145	200	AG	AG	0.9234	ag	0.221480273
NA18855	207	AG	AG	0.9037	ag	0.204044132
NA18505	208	AG	AG	0.9218	ag	0.379953633
NA18862	215	AG	AG	0.9081	ag	0.061181678
NA18856	216	AG	AG	0.9186	ag	0.374530742
NA18503	220	AA	AA	1	AA	0.409382593
NA19152	224	AA	AA	1	aa	0.208325502
NA19210	228	AA	AA	1	aa	0.016652154
NA19139	232	AG	AG	0.9239	ag	0.371798801
NA19204	236	AA	AA	1	aa	0.094223045
NA18507	244	AG	AG	0.9042	ag	0.138587539
NA19159	252	AA	AA	1	aa	0.290038109
NA18859	260	AG	AG	0.8969	ag	0.164901488

MACGT discrepancy
LDA discrepancy
LDA NN (0.65 threshold)

		2760396				
**Coriell**	**Sample**	**HapMap Call**	**LDA Call**	**LDA quality score**	**MACGT Call**	**MACGT quality score**
NA12753	1	GG	GG	1	gg	0.00109385
NA12707	8	GG	GG	1	gg	0.001472013
NA11839	16	GG	GG	1	gg	0
NA10859	24	GG	GG	1	gg	0.001081745
NA07034	32	GG	GG	1	gg	0.002913071
NA07055	40	GG	GG	1	gg	0.025585088
NA12814	48	GG	GG	1	gg	0.026339201
NA10839	55	GG	GG	1	gg	0.028558563
NA10847	56	AG	AG	1	ag	0.001926594
NA12717	63	GG	GG	1	gg	0.107954955
NA10861	64	GG	GG	1	gg	0
NA12740	71	GG	GG	1	gg	0.105066085
NA12005	72	GG	GG	1	gg	0.011610669
NA12752	79	AG	AG	0.9968	ag	0.155921556
NA10851	80	GG	GG	1	gg	0.063787645
NA12043	87	GG	GG	1	gg	0.134306084
NA12264	95	GG	GG	1	gg	0.282401151
NA18621	101	GG	GG	1	gg	0.329078183
NA18594	109	AG	AG	0.9999	ag	0.136785328
NA18622	117	GG	GG	1	GG	0.51763129
NA18573	125	AG	AG	0.9795	AG	0.415635759
NA18623	133	GG	GG	1	gg	0.281621773
NA18576	141	GG	GG	1	GG	0.417387253
NA18633	142	GG	GG	1	gg	0.329985785
NA18964	146	GG	GG	1	gg	0.352919616
NA18994	150	AG	AG	0.9966	ag	0.414874222
NA18953	154	AG	AG	1	ag	0.065591826
NA18968	162	AG	AG	0.8025	ag	0.06613375
NA18992	166	GG	GG	1	gg	0.073540848
NA18959	170	GG	GG	1	GG	0.547176979
NA18995	174	AG	AG	1	ag	0.075698788
NA18969	178	GG	GG	1	GG	0.547626091
NA18997	182	AG	AG	1	ag	0
NA18960	186	AG	AG	0.9841	AG	0.452957362
NA18502	192	AA	AA	0.9459	aa	0.24562579
NA18863	199	AA	AA	0.9915	AA	0.619838923
NA19145	200	AG	AG	1	ag	0.219900173
NA18855	207	AG	AG	1	ag	0.172578274
NA18505	208	AG	AG	0.943	AG	0.523194007
NA18862	215	AG	AG	1	ag	0
NA18856	216	AG	AG	1	ag	0.012899735
NA18503	220	AG	AG	0.9998	ag	0.108404865
NA19152	224	AG	AG	0.9995	ag	0.184130699
NA19210	228	GG	GG	1	gg	0.138894355
NA19139	232	AA	AA	0.8509	AA	0.619838923
NA19204	236	AG	AG	0.9999	ag	0.031436384
NA18507	244	AG	AG	0.999	ag	0.142223906
NA19159	252	AG	AG	1	ag	0.260422094
NA18859	260	AG	AG	0.999	ag	0.104284606

	MACGT discrepancy
	LDA discrepancy
	LDA NN (0.65 threshold)

		2803543				
**Coriell**	**Sample**	**HapMap Call**	**LDA Call**	**LDA quality score**	**MACGT Call**	**MACGT quality score**
NA12753	1	GG	GG	1	gg	0
NA12707	8	GG	GG	0.9969	gg	0
NA11839	16	GG	GG	1	gg	0
NA10859	24	AG	AG	0.9994	ag	0
NA07034	32	GG	GG	0.8495	gg	0
NA07055	40	GG	GG	0.9978	gg	0.005265659
NA12814	48	GG	GG	0.9999	gg	0.002489061
NA10839	55	GG	GG	0.9996	gg	0.023397509
NA10847	56	GG	GG	0.9542	gg	0.015305892
NA12717	63	GG	GG	1	gg	0.020176526
NA10861	64	GG	GG	0.743	gg	0.001649571
NA12740	71	GG	GG	1	gg	0.067492249
NA12005	72	GG	GG	0.9987	gg	0.283967534
NA12752	79	GG	GG	1	gg	0.013390394
NA10851	80	AG	AG	1	ag	0.008239926
NA12043	87	GG	GG	0.9999	gg	0.232453697
NA12264	95	GG	GG	0.995	gg	0.062827698
NA18621	101	GG	GG	1	gg	0
NA18594	109	GG	GG	1	gg	0.387561325
NA18622	117	GG	GG	1	gg	0.118725094
NA18573	125	GG	GG	1	gg	0.039736492
NA18623	133	GG	GG	1	gg	0.166657651
NA18576	141	GG	GG	1	gg	0.065093474
NA18633	142	GG	GG	1	GG	0.530297947
NA18964	146	GG	GG	1	GG	0.458631835
NA18994	150	GG	GG	1	GG	0.433659513
NA18953	154	GG	GG	0.9699	gg	0.432724099
NA18968	162	GG	GG	0.999	gg	0.007367499
NA18992	166	GG	GG	1	gg	0.18714264
NA18959	170	GG	GG	1	gg	0.129802658
NA18995	174	GG	GG	1	gg	0.182381206
NA18969	178	GG	GG	1	gg	0.202756002
NA18997	182	GG	GG	0.8771	gg	0
NA18960	186	GG	GG	1	gg	0.055476013
NA18502	192	GG	GG	1	gg	0.166141628
NA18863	199	AG	AG	0.9969	AG	0.521975385
NA19145	200	GG	GG	1	gg	0.069995734
NA18855	207	GG	GG	1	gg	0.084369532
NA18505	208	GG	GG	0.9997	gg	0.140361093
NA18862	215	AG	AG	1	ag	0.172926169
NA18856	216	GG	GG	1	gg	0.015946877
NA18503	220	GG	GG	0.9998	GG	0.45381036
NA19152	224	AG	AG	1	AG	0.414127712
NA19210	228	GG	GG	0.8148	gg	0.016817305
NA19139	232	GG	GG	1	gg	0.427081119
NA19204	236	GG	GG	1	gg	0.00456548
NA18507	244	GG	GG	1	gg	0.122404028
NA19159	252	GG	GG	1	gg	0.103522045
NA18859	260	AG	AG	0.9938	AG	0.509263285

MACGT discrepancy
LDA discrepancy
LDA NN (0.65 threshold)

		2835896				
**Coriell**	**Sample**	**HapMap Call**	**LDA Call**	**LDA quality score**	**MACGT Call**	**MACGT quality score**
NA12753	1	CC	CC	0.9462	cc	0
NA12707	8	CT	CT	0.7567	ct	0.003681725
NA11839	16	CT	CT	0.977	ct	0.002493343
NA10859	24	CT	CT	0.9703	ct	0.00687362
NA07034	32	CT	CT	0.9677	ct	0.003052969
NA07055	40	TT	TT	0.9243	TT	1
NA12814	48	CT	CT	0.9978	CT	0.436755995
NA10839	55	CC	CC	0.7618	cc	0.035803664
NA10847	56	CC	CC	0.9823	CC	0.328308227
NA12717	63	CT	CT	0.9878	ct	0.225186223
NA10861	64	CC	CC	0.991	cc	0.127061303
NA12740	71	CT	CT	0.9989	CT	0.357222765
NA12005	72	CC	CC	0.9935	cc	0.139511434
NA12752	79	CT	CT	0.9612	ct	0.205072317
NA10851	80	CC	CC	0.9746	cc	0.226390966
NA12043	87	CT	CT	1	ct	0.293788217
NA12264	95	CC	CC	0.9217	cc	0.275089322
NA18621	101	CC	CC	0.9734	cc	0.087005913
NA18594	109	CT	CT	0.9986	ct	0.26496218
NA18622	117	CC	CC	0.9901	cc	0.19274501
NA18573	125	CC	CC	0.9932	cc	0.19064896
NA18623	133	CC	CC	0.9907	cc	0.573019578
NA18576	141	CC	CC	0.9938	CC	0.5542099
NA18633	142	CC	CC	0.9675	cc	0.395410374
NA18964	146	CC	CC	0.7385	cc	0.339857486
NA18994	150	CC	CC	0.9916	cc	0.326775691
NA18953	154	CT	CT	0.9962	ct	0.339622196
NA18968	162	CC	CC	0.9932	cc	0.264882065
NA18992	166	CC	CC	0.9947	cc	0.495305562
NA18959	170	CC	CC	0.9839	CC	0.610646082
NA18995	174	CT	CT	0.9623	CT	0.480713512
NA18969	178	CC	CC	0.99	CC	0.694569783
NA18997	182	CC	CC	0.9852	cc	0.035094605
NA18960	186	CC	CC	0.986	cc	0.320233383
NA18502	192	CC	CC	0.8162	cc	0.182532301
NA18863	199	CT	CT	0.9997	ct	0.329542517
NA19145	200	CT	CT	0.9326	ct	0.316437196
NA18855	207	CC	CC	0.9501	cc	0.155534427
NA18505	208	CC	CC	0.869	cc	0.341143925
NA18862	215	CC	CC	0.7208	cc	0.172199079
NA18856	216	CC	CC	0.8126	cc	0.559761362
NA18503	220	CC	CC	0.9842	cc	0.275592758
NA19152	224	CT	CT	0.9537	CT	0.55030331
NA19210	228	CC	CC	0.7787	cc	0.19603285
NA19139	232	CC	CC	0.8299	cc	0.365580238
NA19204	236	CT	CT	1	ct	0.33381708
NA18507	244	CT	CT	1	ct	0.509951329
NA19159	252	CC	CC	0.855	cc	0.270888834
NA18859	260	CT	CT	1	ct	0.143830226

	MACGT discrepancy
	LDA discrepancy
	LDA NN (0.65 threshold)

		2840794				
**Coriell**	**Sample**	**HapMap Call**	**LDA Call**	**LDA quality score**	**MACGT Call**	**MACGT quality score**
NA12753	1	AG	AG	0.9997	ag	0.015235569
NA12707	8	GG	GG	1	gg	0
NA11839	16	GG	GG	1	gg	0
NA10859	24	GG	GG	1	gg	0
NA07034	32	GG	GG	1	gg	0
NA07055	40	GG	GG	1	gg	0.001770048
NA12814	48	GG	GG	1	gg	0.010151126
NA10839	55	GG	GG	1	gg	0.074774634
NA10847	56	GG	GG	1	gg	0.025249442
NA12717	63	GG	GG	1	gg	0.004852494
NA10861	64	GG	GG	1	gg	0.122580292
NA12740	71	GG	GG	1	gg	0.072235106
NA12005	72	AG	AG	0.9998	ag	0.034932554
NA12752	79	GG	GG	1	gg	0.111334936
NA10851	80	GG	GG	1	gg	0.112255797
NA12043	87	GG	GG	1	gg	0.096210543
NA12264	95	AG	AG	0.9997	ag	0.311474811
NA18621	101	GG	GG	1	GG	0.563360234
NA18594	109	GG	GG	1	gg	0.161838903
NA18622	117	GG	GG	1	gg	0.161707941
NA18573	125	GG	GG	1	GG	0.45239794
NA18623	133	GG	GG	1	gg	0.330215444
NA18576	141	GG	GG	1	gg	0.1929096
NA18633	142	AG	AG	0.9997	AG	0.471682475
NA18964	146	GG	GG	1	gg	0.345343957
NA18994	150	GG	GG	1	GG	0.525662875
NA18953	154	GG	GG	0.75	gg	0.123098711
NA18968	162	GG	GG	1	gg	0.020706337
NA18992	166	GG	GG	1	gg	0.002596379
NA18959	170	AG	AG	0.9998	AG	0.311272884
NA18995	174	AG	AG	0.9998	ag	0.193400378
NA18969	178	GG	GG	1	gg	0.185203205
NA18997	182	GG	GG	1	gg	0
NA18960	186	GG	GG	0.75	gg	0.008295031
NA18502	192	AG	AG	0.9997	ag	0.033196266
NA18863	199	AG	AG	0.9998	AG	0.519591485
NA19145	200	GG	GG	1	gg	0.209084856
NA18855	207	AA	AA	0.9535	AA	0.612874053
NA18505	208	GG	GG	1	gg	0.011614569
NA18862	215	AA	AA	1	AA	0.612874053
NA18856	216	GG	GG	1	gg	0.074880593
NA18503	220	AG	AG	0.9998	ag	0.03427415
NA19152	224	GG	GG	1	gg	0.128457203
NA19210	228	AA	AA	0.7738	aa	0.015600737
NA19139	232	GG	GG	1	gg	0.252442598
NA19204	236	AG	AG	0.9998	ag	0.008849467
NA18507	244	AG	AG	0.9999	AG	0.413783388
NA19159	252	AG	AG	0.9998	ag	0.379371291
NA18859	260	AG	AG	0.9999	ag	0.36163496

(red)	MACGT discrepancy
(green)	LDA discrepancy
(peach)	LDA NN (0.65 threshold)

		2901585				
**Coriell**	**Sample**	**HapMap Call**	**LDA Call**	**LDA quality score**	**MACGT Call**	**MACGT quality score**
NA12753	1	AG	AG	0.9999	ag	0.000548318
NA12707	8	GG	GG	0.75	gg	0
NA11839	16	AG	AG	1	ag	0
NA10859	24	AG	AG	1	ag	0
NA07034	32	GG	GG	1	gg	0.01423983
NA07055	40	AG	AG	1	ag	0.013719894
NA12814	48	GG	GG	1	gg	0.045997984
NA10839	55	GG	GG	1	GG	0.33537708
NA10847	56	AG	AG	1	ag	0.040224313
NA12717	63	GG	GG	1	gg	0.072313258
NA10861	64	AA	AA	1	aa	0.004083923
NA12740	71	AG	AG	0.9998	ag	0.175646397
NA12005	72	AG	AG	1	AG	0.395167127
NA12752	79	GG	GG	1	gg	0.039205346
NA10851	80	GG	GG	1	gg	0.24351509
NA12043	87	AG	AG	1	ag	0.489739142
NA12264	95	AG	AG	1	AG	0.605190267
NA18621	101	AA	AA	1	AA	0.529517593
NA18594	109	GG	GG	1	GG	0.495262156
NA18622	117	AA	AA	1	AA	0.498064516
NA18573	125	AG	AG	1	AG	0.415317423
NA18623	133	AG	AG	1	ag	0.296616331
NA18576	141	AA	AA	1	AA	0.482477569
NA18633	142	AA	AA	1	aa	0.364077844
NA18964	146	GG	GG	1	GG	0.600250846
NA18994	150	AA	AA	0.999	aa	0.208271817
NA18953	154	GG	GG	0.9059	gg	0.182101653
NA18968	162	AG	AG	1	ag	0.154622007
NA18992	166	AA	AA	1	AA	0.464619024
NA18959	170	GG	GG	1	GG	0.437881173
NA18995	174	GG	GG	1	gg	0.211069284
NA18969	178	AG	AG	1	ag	0.32411622
NA18997	182	AG	AG	0.9977	ag	0.006158906
NA18960	186	GG	GG	0.9731	gg	0.01854337
NA18502	192	AG	AG	1	ag	0.190727892
NA18863	199	AA	AA	1	aa	0.043403456
NA19145	200	GG	GG	1	gg	0.403411106
NA18855	207	GG	GG	1	gg	0.309856353
NA18505	208	AG	AG	0.75	ag	0.131162788
NA18862	215	AG	AG	0.9997	ag	0.062544877
NA18856	216	GG	GG	1	gg	0.329259458
NA18503	220	AA	AA	1	aa	0.197749582
NA19152	224	AG	AG	1	AG	0.400336766
NA19210	228	AA	AA	0.9266	aa	0.008200067
NA19139	232	AG	AG	1	ag	0.359206708
NA19204	236	AG	AG	1	ag	0.13966216
NA18507	244	AG	AG	1	ag	0.056368224
NA19159	252	AG	AG	1	ag	0.292852928
NA18859	260	GG	GG	1	gg	0.251228399

MACGT discrepancy
LDA discrepancy
LDA NN (0.65 threshold)

		2925067				
Coriell	Sample	HapMap Call	LDA Call	LDA quality score	MACGT Call	MACGT quality score
NA12753	1	AA	AA	1	aa	0.001790979
NA12707	8	GG	GG	0.7488	gg	0
NA11839	16	AA	AA	0.9996	aa	0
NA10859	24	AA	AA	0.8497	aa	0.000997065
NA07034	32	AA	AA	0.8234	aa	0.011993378
NA07055	40	AG	AG	0.8969	ag	0.007164896
NA12814	48	AG	AG	0.6715	ag	0.08502608
NA10839	55	GG	GG	0.7498	gg	0
NA10847	56	AA	AA	0.9925	aa	0.200211552
NA12717	63	GG	GG	0.9694	GG	0.609717655
NA10861	64	AA	AA	0.9998	aa	0.125863328
NA12740	71	AG	AG	0.9998	ag	0.068088552
NA12005	72	AG	AG	1	ag	0.058241344
NA12752	79	AA	AA	1	aa	0.173401168
NA10851	80	AG	AG	0.7758	ag	0.061252886
NA12043	87	AA	AA	1	aa	0.168116272
NA12264	95	AA	AA	0.9979	aa	0.025366779
NA18621	101	AA	AA	1	aa	0.233128008
NA18594	109	AG	AG	0.9873	AG	0.464819104
NA18622	117	GG	GG	0.9996	gg	0.018631795
NA18573	125	AA	AA	1	aa	0.069382265
NA18623	133	AA	AA	1	AA	0.660968541
NA18576	141	AA	AA	1	aa	0.630497329
NA18633	142	AA	AA	1	AA	0.49963098
NA18964	146	AA	AA	0.9992	AA	0.454469347
NA18994	150	AG	AG	0.9997	AG	0.366323544
NA18953	154	AA	AA	0.9984	aa	0.269818225
NA18968	162	AA	AA	1	aa	0.420730429
NA18992	166	AA	AA	1	aa	0.146781706
NA18959	170	AG	AG	1	ag	0.07612226
NA18995	174	AA	AA	1	aa	0.249363035
NA18969	178	AG	AG	1	ag	0.04848359
NA18997	182	AG	AG	0.9862	ag	0.015447862
NA18960	186	AA	AA	0.8965	aa	0.062802347
NA18502	192	GG	GG	0.9969	gg	0.074970696
NA18863	199	AG	AG	0.7806	ag	0.11450295
NA19145	200	AG	AG	1	ag	0.17753368
NA18855	207	AA	AA	0.9998	aa	0.176087687
NA18505	208	AG	AG	0.9991	AG	0.439401472
NA18862	215	AG	AG	0.9936	ag	0.029992099
NA18856	216	GG	GG	0.9934	gg	0.060505836
NA18503	220	GG	GG	0.9995	GG	0.609717655
NA19152	224	AA	AA	1	aa	0.406782676
NA19210	228	AA	AA	1	AA	0.452446009
NA19139	232	AG	AG	0.9987	AG	0.505189357
NA19204	236	AA	AA	0.9996	aa	0.071429417
NA18507	244	AA	AA	1	aa	0.200703511
NA19159	252	AA	AA	1	aa	0.348629666
NA18859	260	AG	AG	0.9962	ag	0.125420983

MACGT discrepancy
LDA discrepancy
LDA NN (0.65 threshold)

		2938675				
**Coriell**	**Sample**	**HapMap Call**	**LDA Call**	**LDA quality score**	**MACGT Call**	**MACGT quality score**
NA12753	1	GT	GT	1	gt	0.046767071
NA12707	8	GT	GT	1	gt	0.006100723
NA11839	16	GT	GT	1	gt	0.079370691
NA10859	24	GT	GT	1	gt	0.022721224
NA07034	32	GG	GG	1	gg	0.030483931
NA07055	40	TT	TT	1	TT	0.54086998
NA12814	48	GT	GT	1	gt	0.482643019
NA10839	55	GT	GT	1	GT	0.478324083
NA10847	56	TT	TT	1	tt	0.342210891
NA12717	63	GT	GT	1	gt	0.408496738
NA10861	64	GG	GG	0.9999	GG	0.522743947
NA12740	71	GT	GT	1	gt	0.485326014
NA12005	72	GT	GT	1	GT	0.440328791
NA12752	79	GT	GT	1	gt	0.179613652
NA10851	80	GG	GG	1	GG	0.484524793
NA12043	87	TT	TT	1	TT	0.520215919
NA12264	95	GG	GG	1	gg	0.339457096
NA18621	101	GG	GG	1	gg	0.307849723
NA18594	109	TT	TT	1	tt	0.332691832
NA18622	117	GG	GG	1	gg	0.281759314
NA18573	125	TT	TT	1	TT	0.578693335
NA18623	133	GG	GG	1	gg	0.311079134
NA18576	141	GG	GG	1	gg	0.444919249
NA18633	142	GG	GG	1	gg	0.363798036
NA18964	146	GG	GG	1	GG	0.456949937
NA18994	150	GG	GG	1	gg	0.476213998
NA18953	154	GT	GT	1	gt	0.488216989
NA18968	162	GG	GG	1	GG	0.534745686
NA18992	166	GG	GG	1	gg	0.292305236
NA18959	170	GG	GG	1	gg	0.380927445
NA18995	174	GG	GG	1	gg	0.203960146
NA18969	178	GG	GG	1	gg	0.403382675
NA18997	182	TT	TT	0.9203	tt	0
NA18960	186	GG	GG	1	gg	0.325044964
NA18502	192	GT	GT	1	gt	0.339504578
NA18863	199	GG	GG	0.9059	gg	0.16608031
NA19145	200	GT	GT	1	gt	0.380650693
NA18855	207	GG	GG	1	gg	0.41925494
NA18505	208	GT	GT	1	GT	0.501747908
NA18862	215	GG	GG	1	gg	0.344844675
NA18856	216	GG	GG	1	gg	0.241452708
NA18503	220	GG	GG	1	gg	0.327034445
NA19152	224	GT	GT	1	gt	0.535975442
NA19210	228	GT	GT	1	gt	0.38258256
NA19139	232	GT	GT	1	gt	0.360548031
NA19204	236	GG	GG	0.9665	gg	0.120918388
NA18507	244	GG	GG	1	gg	0.435765779
NA19159	252	GG	GG	1	gg	0.378977986
NA18859	260	GT	GT	1	gt	0.410783739

MACGT discrepancy
LDA discrepancy
LDA NN (0.65 threshold)

		3776720				
Coriell	Sample	HapMap Call	LDA Call	LDA quality score	MACGT Call	MACGT quality score
NA12753	1	CT	CT	0.8125	ct	0.016104722
NA12707	8	CC	CC	1	cc	0
NA11839	16	TT	TT	1	tt	0.000683823
NA10859	24	CT	CT	0.8322	ct	0.011389482
NA07034	32	TT	TT	1	tt	0.001189613
NA07055	40	CT	CT	0.8663	ct	0.097983907
NA12814	48	TT	TT	1	tt	0.146702943
NA10839	55	TT	TT	1	tt	0.109211467
NA10847	56	CT	CT	0.9635	ct	0.279143794
NA12717	63	CC	CC	0.9137	cc	0.003220306
NA10861	64	TT	TT	1	tt	0.001655637
NA12740	71	CT	CT	0.9796	ct	0.38206857
NA12005	72	TT	TT	1	tt	0
NA12752	79	TT	TT	1	tt	0.158925603
NA10851	80	TT	TT	1	tt	0.010761844
NA12043	87	CT	CT	0.9441	ct	0.65843662
NA12264	95	CT	CT	0.9479	ct	0.590742075
NA18621	101	CT	CT	0.8606	ct	0.533670632
NA18594	109	TT	TT	1	TT	0.535238835
NA18622	117	TT	TT	1	tt	0.344849217
NA18573	125	CC	CC	0.9941	cc	0.009606791
NA18623	133	CC	CC	0.9447	CC	0.63913868
NA18576	141	TT	TT	1	TT	0.568464307
NA18633	142	CT	CT	0.9504	ct	0.604177868
NA18964	146	CT	CT	0.96	CT	0.556620844
NA18994	150	TT	TT	1	TT	0.538257955
NA18953	154	CC	CC	0.894	CC	0.517682438
NA18968	162	CC	CC	0.9474	cc	0
NA18992	166	CT	CT	0.8951	CT	0.528788163
NA18959	170	CT	CT	0.9789	ct	0.375466856
NA18995	174	CT	CT	0.9726	ct	0.452402288
NA18969	178	CT	CT	0.9814	CT	0.441504531
NA18997	182	CT	CT	0.9542	ct	0.033698133
NA18960	186	CT	CT	0.9945	ct	0.256354736
NA18502	192	CT	CC	0.9236	**cc**	**0**
NA18863	199	TT	TT	1	tt	0.222970197
NA19145	200	CC	CC	0.8909	CC	0.647786988
NA18855	207	CT	CT	0.8799	ct	0.407332039
NA18505	208	CT	CT	0.9908	ct	0.355406578
NA18862	215	CT	CT	0.9127	ct	0.23775239
NA18856	216	TT	TT	1	tt	0.072095235
NA18503	220	CT	CT	0.9021	ct	0.41656876
NA19152	224	TT	TT	1	tt	0.107726332
NA19210	228	TT	TT	1	tt	0.026636046
NA19139	232	TT	TT	1	tt	0.384954217
NA19204	236	TT	TT	1	tt	0.014206681
NA18507	244	CT	CT	0.8671	CT	0.416179558
NA19159	252	CC	CC	0.9664	cc	0.288253382
NA18859	260	TT	TT	1	tt	0.253297655

MACGT discrepancy
LDA discrepancy
LDA NN (0.65 threshold)

		3899706				
**Coriell**	**Sample**	**HapMap Call**	**LDA Call**	**LDA quality score**	**MACGT Call**	**MACGT quality score**
NA12753	1	CG	CG	1	cg	0.048624601
NA12707	8	GG	GG	1	gg	0.010895996
NA11839	16	CC	CC	1	cc	0.141695053
NA10859	24	CC	CC	1	cc	0.110958254
NA07034	32	CG	CG	1	CG	0.170968344
NA07055	40	CC	CC	1	cc	0.159419104
NA12814	48	CG	CG	1	CG	0.590161315
NA10839	55	CC	CC	1	cc	0.087069902
NA10847	56	CC	CC	1	cc	0.117259315
NA12717	63	CG	CG	0.9999	cg	0.161593416
NA10861	64	CC	CC	1	cc	0.191702976
NA12740	71	GG	GG	1	GG	0.515766759
NA12005	72	CG	CG	1	cg	0.103725224
NA12752	79	CG	CG	1	cg	0.252290303
NA10851	80	CG	CG	1	cg	0.291874982
NA12043	87	GG	GG	1	GG	0.585161782
NA12264	95	CC	CC	1	cc	0.176216457
NA18621	101	CC	CC	1	CC	0.647225854
NA18594	109	CC	CC	1	CC	0.681095653
NA18622	117	CC	CC	1	cc	0.165361507
NA18573	125	CC	CC	1	CC	0.468643386
NA18623	133	CC	CC	1	cc	0.51702197
NA18576	141	CC	CC	1	cc	0.676409084
NA18633	142	CC	CC	1	cc	0.464213227
NA18964	146	CC	CC	1	cc	0.360450678
NA18994	150	CG	CG	1	cg	0.057332524
NA18953	154	CC	CC	1	cc	0.408647208
NA18968	162	CC	CC	1	cc	0.364843707
NA18992	166	CC	CC	1	cc	0.247734704
NA18959	170	CC	CC	1	CC	0.57260366
NA18995	174	CC	CC	1	cc	0.36618548
NA18969	178	CG	CG	1	CG	0.528693812
NA18997	182	CG	CG	1	cg	0.077770816
NA18960	186	CC	CC	1	cc	0.395487562
NA18502	192	CC	CC	1	cc	0.127547597
NA18863	199	CC	CC	1	cc	0.154831726
NA19145	200	GG	GG	1	GG	0.522120825
NA18855	207	CC	CC	1	cc	0.13548186
NA18505	208	CC	CC	1	cc	0.294853658
NA18862	215	CC	CC	1	cc	0.23532055
NA18856	216	CC	CC	1	cc	0.296927177
NA18503	220	CC	CC	1	cc	0.165206768
NA19152	224	CC	CC	1	cc	0.248706977
NA19210	228	CC	CC	1	cc	0.012005091
NA19139	232	CG	CG	1	CG	0.568520864
NA19204	236	CC	CC	1	cc	0.015302424
NA18507	244	GG	GG	1	GG	0.440333633
NA19159	252	CG	CG	1	cg	0.321766013
NA18859	260	CC	CC	1	cc	0.043811645

(red)	MACGT discrepancy
(green)	LDA discrepancy
(peach)	LDA NN (0.65 threshold)

		4306755				
**Coriell**	**Sample**	**HapMap Call**	**LDA Call**	**LDA quality score**	**MACGT Call**	**MACGT quality score**
NA12753	1	AG	AG	1	ag	0.080445262
NA12707	8	AG	AG	1	AG	0.287301979
NA11839	16	AA	AA	1	aa	0.043041951
NA10859	24	GG	GG	1	gg	0
NA07034	32	AG	AG	1	ag	0.081513279
NA07055	40	AG	AG	1	AG	0.534019082
NA12814	48	AG	AG	1	ag	0.163452829
NA10839	55	AG	AG	1	AG	0.473663784
NA10847	56	AG	AG	1	ag	0.244006876
NA12717	63	AG	AG	1	ag	0.25002946
NA10861	64	AA	AA	1	aa	0.140735827
NA12740	71	AG	AG	1	ag	0.155416125
NA12005	72	GG	GG	1	gg	0.286152497
NA12752	79	AA	AA	1	aa	0.203632463
NA10851	80	AA	AA	1	aa	0.317267536
NA12043	87	AA	AA	1	aa	0.459127427
NA12264	95	AG	AG	1	ag	0.046426832
NA18621	101	GG	GG	1	GG	0.472844986
NA18594	109	AG	AG	1	ag	0.056341272
NA18622	117	AA	AA	1	aa	0.305213178
NA18573	125	AG	AG	1	AG	0.396015003
NA18623	133	AA	AA	1	AA	0.619483685
NA18576	141	GG	GG	1	GG	0.457281461
NA18633	142	AG	AG	1	ag	0.120393159
NA18964	146	GG	GG	1	GG	0.540584683
NA18994	150	AG	AG	1	ag	0.145671186
NA18953	154	GG	GG	1	gg	0.33516346
NA18968	162	AG	AG	1	ag	0.09491441
NA18992	166	GG	GG	1	gg	0.184967922
NA18959	170	AA	AA	1	aa	0.472928125
NA18995	174	AG	AG	1	ag	0.171312749
NA18969	178	AA	AA	1	aa	0.50601344
NA18997	182	GG	GG	1	gg	0.000888322
NA18960	186	GG	GG	1	GG	0.436051048
NA18502	192	AA	AA	0.9923	aa	0.215804155
NA18863	199	AA	AA	0.9992	aa	0.026780231
NA19145	200	AA	AA	1	aa	0.372656016
NA18855	207	AA	AA	1	AA	0.510023293
NA18505	208	AA	AA	1	AA	0.586028168
NA18862	215	AA	AA	1	aa	0.397262324
NA18856	216	AA	AA	1	aa	0.414348007
NA18503	220	AA	AA	1	aa	0.424233499
NA19152	224	AA	AA	1	aa	0.455856603
NA19210	228	AA	AA	1	AA	0.366913626
NA19139	232	AA	AA	1	aa	0.363975693
NA19204	236	AA	AA	1	aa	0.283554321
NA18507	244	AA	AA	1	aa	0.429130628
NA19159	252	AA	AA	1	aa	0.378995314
NA18859	260	AA	AA	1	aa	0.392038162

MACGT discrepancy
LDA discrepancy
LDA NN (0.65 threshold)

		4606154				
Coriell	Sample	HapMap Call	LDA Call	LDA quality score	MACGT Call	MACGT quality score
NA12753	1	GG	GG	0.9546	gg	0.002271993
NA12707	8	AG	AG	0.8103	ag	0.025299573
NA11839	16	AG	AG	0.995	ag	0.001116653
NA10859	24	AG	AG	0.9954	ag	0.000852228
NA07034	32	GG	GG	0.9993	gg	0.119477202
NA07055	40	GG	GG	0.9975	gg	0.181358396
NA12814	48	AA	AA	1	aa	0.308549079
NA10839	55	AG	AG	0.9995	ag	0.286886445
NA10847	56	GG	GG	0.9989	gg	0.145438105
NA12717	63	GG	GG	0.997	gg	0.266231212
NA10861	64	AG	AG	0.9997	ag	0.154157165
NA12740	71	AG	AG	0.9997	AG	0.364330749
NA12005	72	AG	AG	0.9996	AG	0.441919297
NA12752	79	GG	GG	1	gg	0.373871905
NA10851	80	AG	AG	0.9997	ag	0.22910038
NA12043	87	AA	AA	0.9957	aa	0.413069187
NA12264	95	GG	GG	0.9839	gg	0.495045986
NA18621	101	AG	AG	0.9977	ag	0.401231656
NA18594	109	AG	AG	0.9525	AG	0.546806254
NA18622	117	GG	GG	0.9999	GG	0.432076125
NA18573	125	AA	AA	0.9997	AA	0.520582515
NA18623	133	AA	AA	0.9998	AA	0.538860924
NA18576	141	AG	AG	0.9641	AG	0.638305101
NA18633	142	AG	AG	0.9951	ag	0.51411274
NA18964	146	GG	GG	0.9996	gg	0.190877319
NA18994	150	AA	AA	0.9999	AA	0.529234596
NA18953	154	AG	AG	0.9972	ag	0.40975659
NA18968	162	GG	GG	0.9997	GG	0.566142341
NA18992	166	GG	GG	0.9935	gg	0.197736576
NA18959	170	AG	AG	0.9987	ag	0.536637265
NA18995	174	AG	AG	0.9935	ag	0.293508904
NA18969	178	GG	GG	0.985	gg	0.010204992
NA18997	182	AG	AG	0.9981	ag	0.217015817
NA18960	186	AG	AG	0.9943	ag	0.365612479
NA18502	192	AG	AG	0.9722	ag	0.198284386
NA18863	199	GG	GG	0.9996	gg	0.355439043
NA19145	200	AA	AA	1	AA	0.519966128
NA18855	207	GG	GG	0.9974	gg	0.350665343
NA18505	208	GG	GG	0.9694	GG	0.597870017
NA18862	215	GG	GG	0.9999	gg	0.186889619
NA18856	216	AG	AG	0.9964	ag	0.423106965
NA18503	220	GG	GG	0.9941	gg	0.316908862
NA19152	224	AA	AA	0.9997	aa	0.273642729
NA19210	228	GG	GG	0.8984	GG	0.5273337
NA19139	232	GG	GG	0.999	gg	0.333471547
NA19204	236	GG	GG	0.8827	gg	0.182004712
NA18507	244	AG	AG	0.9935	ag	0.357910553
NA19159	252	AA	AA	0.9999	aa	0.251752784
NA18859	260	AG	AG	0.9985	ag	0.344869582

MACGT discrepancy
LDA discrepancy
LDA NN (0.65 threshold)

		4739199				
Coriell	Sample	HapMap Call	LDA Call	LDA quality score	MACGT Call	MACGT quality score
NA12753	1	TT	TT	0.8987	tt	0.030238449
NA12707	8	CT	CT	0.8635	ct	0.103394314
NA11839	16	CT	CT	0.8371	ct	0.299647661
NA10859	24	CT	CT	0.8653	ct	0.376029746
NA07034	32	CC	CC	0.9642	CC	0.379061504
NA07055	40	CT	CT	0.8507	ct	0.42063855
NA12814	48	CT	CT	0.8642	CT	0.581143267
NA10839	55	TT	TT	0.9633	TT	0.549891649
NA10847	56	CT	CT	0.8485	ct	0.339061836
NA12717	63	CT	CT	0.8172	ct	0.165930303
NA10861	64	CT	CT	0.8978	ct	0.255318629
NA12740	71	CT	CT	0.7218	ct	0.1218356
NA12005	72	TT	TT	0.9434	TT	0.474715763
NA12752	79	CC	CC	0.9343	cc	0.367092792
NA10851	80	CT	CT	0.862	CT	0.306810726
NA12043	87	CT	CT	0.8442	ct	0.407033142
NA12264	95	TT	TT	0.9569	tt	0.167298265
NA18621	101	TT	TT	0.7773	TT	0.351457348
NA18594	109	CT	CT	0.8947	CT	0.496384238
NA18622	117	CC	CC	0.9307	cc	0.510767752
NA18573	125	CC	CC	0.8888	cc	0.03395547
NA18623	133	CC	CC	0.901	cc	0.375463132
NA18576	141	CT	CT	0.8572	CT	0.501766182
NA18633	142	CC	CC	0.8969	CC	0.492937909
NA18964	146	CC	CC	0.9078	cc	0.331370484
NA18994	150	CC	CC	0.9159	CC	0.609702884
NA18953	154	CC	CC	0.902	cc	0.387647534
NA18968	162	CT	CT	0.8724	ct	0.409921684
NA18992	166	CC	CC	0.8858	cc	0.172681476
NA18959	170	CC	CC	0.9044	cc	0
NA18995	174	CC	CC	0.9295	cc	0.392468039
NA18969	178	CC	CC	0.9069	cc	0.206581725
NA18997	182	CC	CC	0.9279	cc	0.202663137
NA18960	186	CC	CC	0.8973	cc	0.249152439
NA18502	192	CT	CT	0.8728	ct	0.314097157
NA18863	199	CT	CT	0.8572	ct	0.470897258
NA19145	200	CT	CT	0.7743	ct	0.225190551
NA18855	207	TT	TT	0.9555	tt	0.311518453
NA18505	208	CC	CC	0.9147	cc	0.011969163
NA18862	215	CT	CT	0.8026	ct	0.124377195
NA18856	216	CT	CT	0.8084	ct	0.310555645
NA18503	220	CT	CT	0.8469	ct	0.059724923
NA19152	224	TT	TT	0.7736	tt	0.266958938
NA19210	228	CT	CT	0.4876	**tt**	**0**
NA19139	232	CC	CC	0.8962	CC	0.634400369
NA19204	236	TT	TT	0.9221	TT	0.61796497
NA18507	244	CT	CT	0.8537	ct	0.366063176
NA19159	252	CT	CT	0.8197	ct	0.485317086
NA18859	260	TT	TT	0.9524	tt	0.531058224

MACGT discrepancy
LDA discrepancy
LDA NN (0.65 threshold)

		4873622				
**Coriell**	**Sample**	**HapMap Call**	**LDA Call**	**LDA quality score**	**MACGT Call**	**MACGT quality score**
NA12753	1	CG	CG	0.7531	cg	0.092659668
NA12707	8	CG	CG	0.9709	cg	0.001724378
NA11839	16	CC	CC	0.9998	cc	0.011273489
NA10859	24	CG	CG	0.9999	cg	0.01471727
NA07034	32	CG	CG	0.9998	cg	0.002480236
NA07055	40	CC	CC	0.9999	CC	0.459137987
NA12814	48	CC	CC	0.9998	cc	0.456103507
NA10839	55	CG	CG	0.9996	cg	0.238143669
NA10847	56	CG	CG	1	cg	0.198587566
NA12717	63	CG	CG	0.7691	cg	0.439172131
NA10861	64	CC	CC	0.9998	cc	0.002325493
NA12740	71	CC	CC	0.9995	cc	0.313982799
NA12005	72	CG	CG	0.999	CG	0.433061133
NA12752	79	CG	CG	0.9438	cg	0.263629549
NA10851	80	CC	CC	0.9879	CC	0.473475499
NA12043	87	CC	CC	0.9416	CC	0.568695975
NA12264	95	CC	CC	0.9973	cc	0.296246114
NA18621	101	CG	CG	0.988	CG	0.477240924
NA18594	109	CG	CG	0.9859	cg	0.34577644
NA18622	117	CG	CG	0.7737	cg	0.496473474
NA18573	125	GG	GG	1	gg	0.336931982
NA18623	133	GG	GG	1	gg	0.580818949
NA18576	141	CG	CG	0.8883	cg	0.427372046
NA18633	142	GG	GG	1	gg	0.538483048
NA18964	146	CG	CG	1	CG	0.569464882
NA18994	150	GG	GG	1	gg	0.027486164
NA18953	154	GG	GG	0.75	gg	0.417297163
NA18968	162	CC	CC	0.9999	cc	0.149885832
NA18992	166	CG	CG	0.8485	cg	0.336319957
NA18959	170	CG	CG	0.9948	CG	0.566179664
NA18995	174	GG	GG	1	gg	0.169533642
NA18969	178	GG	GG	0.75	gg	0.272266991
NA18997	182	GG	GG	1	GG	0.410685905
NA18960	186	CC	CC	0.9773	cc	0
NA18502	192	CG	CG	1	cg	0.232560918
NA18863	199	GG	GG	1	GG	0.662986869
NA19145	200	CC	CC	1	cc	0.40009381
NA18855	207	CG	CG	0.8513	cg	0.579914181
NA18505	208	CG	CG	0.9889	cg	0.185851507
NA18862	215	CG	CG	0.9818	cg	0.295355094
NA18856	216	CG	CG	0.9987	cg	0.403342587
NA18503	220	GG	GG	1	GG	0.558532389
NA19152	224	CC	CC	1	CC	0.558765448
NA19210	228	GG	GG	1	GG	0.549248858
NA19139	232	CG	CG	0.8897	cg	0.330736384
NA19204	236	CC	CC	0.931	cc	0.163313116
NA18507	244	GG	GG	0.7502	gg	0
NA19159	252	GG	GG	0.7501	gg	0.02205235
NA18859	260	CG	CG	0.9992	cg	0.341247019

MACGT discrepancy
LDA discrepancy
LDA NN (0.65 threshold)

		4933826				
Coriell	Sample	HapMap Call	LDA Call	LDA quality score	MACGT Call	MACGT quality score
NA12753	1	CG	CG	0.9988	cg	0.153285397
NA12707	8	CC	CC	1	cc	0.045229592
NA11839	16	CC	CC	1	cc	0.03555188
NA10859	24	CC	CC	0.9999	cc	0.006457022
NA07034	32	CG	CG	1	CG	0.229967251
NA07055	40	CC	CC	1	CC	0.432703561
NA12814	48	CG	CG	1	CG	0.582156353
NA10839	55	CG	CG	1	cg	0.108036867
NA10847	56	GG	GG	1	gg	0.091092498
NA12717	63	CC	CC	1	cc	0.207384696
NA10861	64	CC	CC	1	cc	0.086735127
NA12740	71	CG	CG	1	cg	0.21465818
NA12005	72	CG	CG	1	cg	0.317384245
NA12752	79	CG	CG	1	cg	0.379609563
NA10851	80	CC	CC	1	CC	0.406697481
NA12043	87	CC	CC	1	cc	0.250020502
NA12264	95	CG	CG	1	cg	0.275254487
NA18621	101	CG	CG	1	cg	0.122274419
NA18594	109	CG	CG	1	cg	0.257486829
NA18622	117	CG	CG	1	cg	0.485705891
NA18573	125	CC	CC	1	cc	0.296945057
NA18623	133	GG	GG	1	gg	0.14294326
NA18576	141	CC	CC	1	CC	0.594870469
NA18633	142	CG	CG	1	CG	0.388324711
NA18964	146	CC	CC	1	cc	0.274260998
NA18994	150	GG	GG	1	GG	0.593037197
NA18953	154	GG	GG	1	gg	0.154779067
NA18968	162	CG	CG	1	cg	0.314456443
NA18992	166	CG	CG	1	cg	0.231708959
NA18959	170	CC	CC	1	CC	0.435702068
NA18995	174	CC	CC	1	cc	0.130232725
NA18969	178	CG	CG	1	cg	0.164219692
NA18997	182	CC	CC	1	cc	0.064598168
NA18960	186	CC	CC	1	cc	0.198255966
NA18502	192	CC	CC	1	cc	0.347115286
NA18863	199	GG	GG	1	gg	0.037678808
NA19145	200	CC	CC	1	cc	0.086407942
NA18855	207	CG	CG	1	CG	0.537988229
NA18505	208	CC	CC	1	cc	0.223115214
NA18862	215	GG	GG	1	GG	0.445134077
NA18856	216	CG	CG	1	cg	0.112055183
NA18503	220	CC	CC	1	cc	0.281693614
NA19152	224	CC	CC	1	cc	0.336624188
NA19210	228	CC	CC	1	cc	0.202698032
NA19139	232	CC	CC	1	cc	0.410392563
NA19204	236	GG	GG	1	GG	0.564697907
NA18507	244	CC	CC	1	cc	0.415403881
NA19159	252	CG	CG	1	cg	0.391786232
NA18859	260	CG	CG	1	cg	0.432158347

	MACGT discrepancy
	LDA discrepancy
	LDA NN (0.65 threshold)

		4971653				
**Coriell**	**Sample**	**HapMap Call**	**LDA Call**	**LDA quality score**	**MACGT Call**	**MACGT quality score**
NA12753	1	GG	GG	1	gg	0.002693655
NA12707	8	GG	GG	1	gg	0.004486909
NA11839	16	GG	GG	1	gg	0
NA10859	24	AG	AG	0.9722	ag	0.255690871
NA07034	32	AG	AG	0.9841	AG	0.310713024
NA07055	40	GG	GG	1	gg	0.074663267
NA12814	48	GG	GG	1	gg	0.167260255
NA10839	55	AG	AG	1	ag	0.147583944
NA10847	56	GG	GG	1	gg	0.222297399
NA12717	63	GG	GG	1	gg	0.159554846
NA10861	64	GG	GG	1	gg	0.027213446
NA12740	71	GG	GG	1	gg	0.37191268
NA12005	72	AA	AA	0.9982	AA	0.638832608
NA12752	79	AG	AG	0.9991	ag	0.215779217
NA10851	80	AG	AG	0.9999	ag	0.308231274
NA12043	87	GG	GG	0.9884	gg	0.193696981
NA12264	95	GG	GG	1	gg	0.3532534
NA18621	101	AG	AG	0.7545	ag	0.250474334
NA18594	109	AG	AG	0.9997	AG	0.49963987
NA18622	117	AG	AG	0.9895	ag	0.245779432
NA18573	125	AG	AG	0.9993	AG	0.370659013
NA18623	133	AA	AA	0.9989	AA	0.638832608
NA18576	141	GG	GG	1	GG	0.498692679
NA18633	142	GG	GG	1	gg	0.450320619
NA18964	146	GG	GG	1	GG	0.55653472
NA18994	150	GG	GG	1	GG	0.423498025
NA18953	154	AG	AG	1	ag	0.184912906
NA18968	162	GG	GG	1	gg	0.025134619
NA18992	166	AG	AG	0.9999	ag	0.4405795
NA18959	170	AG	AG	1	AG	0.453557571
NA18995	174	AG	AG	0.9997	ag	0.156371794
NA18969	178	AG	AG	1	ag	0.441359283
NA18997	182	GG	GG	1	gg	0.001415898
NA18960	186	AG	AG	1	ag	0.075518502
NA18502	192	GG	GG	1	gg	0.187672324
NA18863	199	GG	GG	1	gg	0.158635761
NA19145	200	GG	GG	1	gg	0.385158921
NA18855	207	GG	GG	1	gg	0.366724634
NA18505	208	GG	GG	0.8036	gg	0.089091309
NA18862	215	GG	GG	1	gg	0.168194578
NA18856	216	GG	GG	1	gg	0.357243525
NA18503	220	GG	GG	1	GG	0.440454406
NA19152	224	GG	GG	1	gg	0.360254185
NA19210	228	GG	GG	0.75	gg	0.095957338
NA19139	232	GG	GG	1	gg	0.415755294
NA19204	236	GG	GG	0.75	gg	0.054720062
NA18507	244	GG	GG	1	gg	0.205730304
NA19159	252	AG	AG	0.9999	ag	0.189057545
NA18859	260	GG	GG	1	gg	0.199624971

(red)	MACGT discrepancy
(green)	LDA discrepancy
(peach)	LDA NN (0.65 threshold)

		6068122				
**Coriell**	**Sample**	**HapMap Call**	**LDA Call**	**LDA quality score**	**MACGT Call**	**MACGT quality score**
NA12753	1	TT	TT	0.982	TT	1
NA12707	8	AT	AT	0.9994	AT	0.244538577
NA11839	16	AA	AA	0.9997	aa	0.003215029
NA10859	24	AA	AA	0.9994	aa	0.013563158
NA07034	32	AT	AT	0.9991	at	0.092380344
NA07055	40	AA	AA	0.9999	aa	0.209278033
NA12814	48	AA	AA	0.9999	aa	0.146436117
NA10839	55	AA	AA	0.8505	aa	0.017680247
NA10847	56	AT	AT	0.9991	at	0.464640329
NA12717	63	AT	AT	0.9984	at	0.351963395
NA10861	64	AA	AA	0.9966	aa	0.01647585
NA12740	71	AA	AA	0.9999	aa	0.004059497
NA12005	72	AT	AT	0.9982	AT	0.410760292
NA12752	79	AT	AT	0.9978	at	0.242710679
NA10851	80	AA	AA	0.9792	AA	0.357398087
NA12043	87	AT	AT	0.9972	AT	0.453107202
NA12264	95	AA	AA	0.9566	aa	0
NA18621	101	AA	AA	0.9402	aa	0.003109802
NA18594	109	AA	AA	0.9989	AA	0.444987919
NA18622	117	AA	AA	1	aa	0.156756688
NA18573	125	AA	AA	1	aa	0.033842036
NA18623	133	AA	AA	1	aa	0.26656722
NA18576	141	AA	AA	1	aa	0.345933351
NA18633	142	AA	AA	1	AA	0.646626439
NA18964	146	AA	AA	0.9968	aa	0.141366701
NA18994	150	AA	AA	0.9999	AA	0.461188451
NA18953	154	AA	AA	0.9501	aa	0.028627751
NA18968	162	AA	AA	0.996	aa	0.139849943
NA18992	166	AT	AT	0.9978	at	0.038402001
NA18959	170	AA	AA	0.9984	aa	0.263716428
NA18995	174	AT	AT	0.9987	at	0.056852531
NA18969	178	AA	AA	0.9999	aa	0.131410278
NA18997	182	AA	AA	0.8753	aa	0.007785115
NA18960	186	AA	AA	0.9999	aa	0.152744997
NA18502	192	AA	AA	0.7488	aa	0.006571228
NA18863	199	AA	AA	0.9999	aa	0.083906673
NA19145	200	AA	AA	0.792	aa	0.014654329
NA18855	207	AA	AA	0.9998	aa	0.314802991
NA18505	208	AA	AA	0.7727	aa	0.063153427
NA18862	215	AA	AA	0.7542	aa	0.009485259
NA18856	216	AA	AA	0.7493	aa	0.018404222
NA18503	220	AA	AA	0.7496	aa	0.001648694
NA19152	224	AA	AA	0.7563	aa	0.014967448
NA19210	228	AA	AA	0.7501	aa	0
NA19139	232	AA	AA	0.7545	aa	0.032427982
NA19204	236	AA	AA	0.62	aa	0.001280547
NA18507	244	AT	AT	0.9986	AT	0.586173386
NA19159	252	AA	AA	0.9948	aa	0.360788056
NA18859	260	AA	AA	1	aa	0.256891094

MACGT discrepancy
LDA discrepancy
LDA NN (0.65 threshold)

		6478813				
**Coriell**	**Sample**	**HapMap Call**	**LDA Call**	**LDA quality score**	**MACGT Call**	**MACGT quality score**
NA12753	1	AT	AT	0.7749	at	0.051000063
NA12707	8	TT	TT	1	tt	0.046801581
NA11839	16	TT	TT	1	tt	0.070295064
NA10859	24	TT	TT	1	tt	0.0593614
NA07034	32	AT	AT	0.9921	AT	0.243498364
NA07055	40	AT	AT	0.9774	at	0.334085746
NA12814	48	TT	TT	0.9999	tt	0.458257763
NA10839	55	TT	TT	0.9994	TT	0.543847936
NA10847	56	TT	TT	1	tt	0.574859916
NA12717	63	TT	TT	1	tt	0.500714625
NA10861	64	AT	AT	1	at	0.044017062
NA12740	71	TT	TT	0.75	tt	0.466094917
NA12005	72	TT	TT	1	tt	0.055837112
NA12752	79	TT	TT	0.9123	tt	0.002775575
NA10851	80	TT	TT	1	TT	0.390799832
NA12043	87	TT	TT	0.9824	tt	0.001757226
NA12264	95	TT	TT	0.9994	tt	0.617684727
NA18621	101	AT	AT	0.9998	AT	0.497333081
NA18594	109	TT	TT	0.9999	tt	0.641252397
NA18622	117	AT	AT	1	AT	0.413762921
NA18573	125	AA	AA	0.9824	AA	0.48101969
NA18623	133	TT	TT	0.9961	tt	0.567639554
NA18576	141	AT	AT	0.999	at	0
NA18633	142	TT	TT	1	tt	0.531476167
NA18964	146	TT	TT	0.9962	tt	0.328390311
NA18994	150	TT	TT	1	TT	0.453335012
NA18953	154	TT	TT	0.75	tt	0.167902768
NA18968	162	TT	TT	0.9307	tt	0.339925691
NA18992	166	TT	TT	0.9999	tt	0.086696877
NA18959	170	TT	TT	0.9996	TT	0.623593685
NA18995	174	AT	AT	1	at	0.005149853
NA18969	178	AT	AT	1	at	0.250432052
NA18997	182	AT	AT	1	at	0
NA18960	186	TT	TT	0.8388	tt	0.046636485
NA18502	192	AT	AT	0.7821	at	0.21075931
NA18863	199	AT	AT	0.9867	at	0.410368476
NA19145	200	AT	AT	1	at	0.004321874
NA18855	207	AA	AA	1	AA	0.514785743
NA18505	208	AA	AA	1	AA	0.472857079
NA18862	215	AT	AT	1	at	0.004500172
NA18856	216	TT	TT	0.75	tt	0.347910269
NA18503	220	AT	AT	0.9851	at	0.194923559
NA19152	224	AT	AT	0.9996	at	0.386073356
NA19210	228	AT	AT	1	AT	0.567016862
NA19139	232	AA	AA	0.9736	aa	0.414708256
NA19204	236	AT	AT	1	at	0.287879888
NA18507	244	TT	TT	0.8686	tt	0.38968336
NA19159	252	AA	AA	0.9532	aa	0.285563733
NA18859	260	AA	AA	0.9993	aa	0.438595795

MACGT discrepancy
LDA discrepancy
LDA NN (0.65 threshold)

		7292634				
Coriell	Sample	HapMap Call	LDA Call	LDA quality score	MACGT Call	MACGT quality score
NA12753	1	CT	CT	0.7574	ct	0.003888069
NA12707	8	CC	CC	1	cc	0
NA11839	16	CC	CC	1	cc	0
NA10859	24	CC	CC	1	cc	0
NA07034	32	CC	CC	1	cc	0
NA07055	40	CC	CC	1	cc	0.002583331
NA12814	48	CT	CT	0.9238	CT	0.300780642
NA10839	55	CC	CC	1	cc	0.052653759
NA10847	56	CT	CT	0.7658	ct	0.038568039
NA12717	63	CC	CC	0.995	cc	0.266234892
NA10861	64	CC	CC	0.9979	cc	0.097142121
NA12740	71	CC	CC	0.9939	cc	0.212424925
NA12005	72	CC	CC	0.9995	CC	0.484248271
NA12752	79	CC	CC	0.9999	cc	0.007985033
NA10851	80	CC	CC	0.9998	CC	0.406276419
NA12043	87	CC	CC	1	cc	0.34768896
NA12264	95	CC	CC	0.9999	cc	0.476749856
NA18621	101	CT	CT	0.9084	ct	0.340271682
NA18594	109	CC	CC	0.968	CC	0.446858605
NA18622	117	CC	CC	1	cc	0.267994462
NA18573	125	CC	CC	0.999	cc	0.057429078
NA18623	133	CC	CC	1	cc	0.420122933
NA18576	141	CC	CC	0.9722	cc	0.418262713
NA18633	142	CC	CC	0.9997	cc	0.403561235
NA18964	146	CC	CC	1	cc	0.313092076
NA18994	150	CT	CT	0.9976	CT	0.477212603
NA18953	154	CC	CC	0.9361	cc	0.268031029
NA18968	162	CC	CC	1	cc	0.105230308
NA18992	166	CC	CC	0.8991	cc	0.029566051
NA18959	170	CC	CC	0.9958	cc	0.333707053
NA18995	174	CC	CC	1	cc	0.015245422
NA18969	178	CC	CC	1	CC	0.3934622
NA18997	182	CC	CC	0.7607	cc	0.009483651
NA18960	186	CC	CC	1	cc	0.204311797
NA18502	192	CT	CT	0.9997	ct	0.400243075
NA18863	199	CT	CT	1	CT	0.576153786
NA19145	200	CC	CC	0.8391	cc	0.158290134
NA18855	207	CT	CT	1	CT	0.452898965
NA18505	208	CC	CC	0.7518	cc	0.145106605
NA18862	215	CC	CC	0.9752	cc	0.097186672
NA18856	216	CC	CC	0.985	cc	0.342364525
NA18503	220	CC	CC	0.8388	cc	0.260837931
NA19152	224	CT	CT	0.9985	ct	0.351244822
NA19210	228	CT	CT	1	ct	0.084827598
NA19139	232	CC	CC	0.9874	cc	0.146549992
NA19204	236	CC	CC	0.7639	cc	0.072265587
NA18507	244	CC	CC	0.9985	cc	0.112293132
NA19159	252	CC	CC	0.9904	cc	0.289691293
NA18859	260	CC	CC	1	cc	0.010235752

(red)	MACGT discrepancy
(green)	LDA discrepancy
(peach)	LDA NN (0.65 threshold)

		7555995				
**Coriell**	**Sample**	**HapMap Call**	**LDA Call**	**LDA quality score**	**MACGT Call**	**MACGT quality score**
NA12753	1	CC	CC	0.9965	cc	0.209643097
NA12707	8	CC	CC	0.9999	cc	0.244042505
NA11839	16	CC	CC	1	cc	0.176274513
NA10859	24	CC	CC	0.9993	cc	0.225832188
NA07034	32	CC	CC	1	CC	0.338600866
NA07055	40	GG	GG	1	GG	0.48833371
NA12814	48	CC	CC	1	cc	0.406620971
NA10839	55	CC	CC	0.9991	cc	0.441883088
NA10847	56	CG	CG	0.812	cg	0.214612942
NA12717	63	CC	CC	1	cc	0.474339771
NA10861	64	CC	CC	1	cc	0.274681576
NA12740	71	CG	CG	0.751	cg	0.109755969
NA12005	72	CC	CC	0.9998	CC	0.613801439
NA12752	79	CC	CC	0.9824	cc	0.603519856
NA10851	80	CC	CC	1	cc	0.321659131
NA12043	87	CC	CC	1	cc	0.377563583
NA12264	95	CC	CC	1	cc	0.439926423
NA18621	101	GG	GG	1	GG	0.810491234
NA18594	109	CG	CG	0.9962	CG	0.410303543
NA18622	117	CG	CG	0.9698	cg	0.271361274
NA18573	125	CG	CG	1	CG	0.503667573
NA18623	133	CG	CG	0.9999	CG	0.637875186
NA18576	141	CC	CC	0.9853	CC	0.648373474
NA18633	142	GG	GG	1	GG	0.473282585
NA18964	146	CG	CG	0.9984	cg	0.264457007
NA18994	150	CC	CC	0.9733	CC	0.52211529
NA18953	154	CG	CG	0.9997	cg	0.192347954
NA18968	162	CG	CG	0.9886	cg	0.060676319
NA18992	166	CG	CG	0.9937	cg	0.347852978
NA18959	170	GG	GG	1	GG	0.685635676
NA18995	174	CC	CC	0.8962	cc	0.317849741
NA18969	178	CG	CG	0.9991	CG	0.579313549
NA18997	182	GG	GG	0.9971	gg	0.340665697
NA18960	186	CG	CG	0.8516	cg	0.054085132
NA18502	192	CG	CG	0.9996	cg	0.293418809
NA18863	199	CC	CC	0.9877	cc	0.336262677
NA19145	200	CC	CC	1	cc	0.347534161
NA18855	207	GG	GG	1	gg	0.682420299
NA18505	208	CG	CG	0.9956	cg	0.388585264
NA18862	215	CC	CC	0.9984	cc	0.459956409
NA18856	216	CC	CC	0.9998	cc	0.276073124
NA18503	220	GG	GG	1	gg	0.494304601
NA19152	224	CC	CC	1	cc	0.21576859
NA19210	228	CC	CC	0.9999	cc	0.403122308
NA19139	232	CG	CG	0.9989	cg	0.454052622
NA19204	236	CG	CG	0.7695	cg	0.073018468
NA18507	244	GG	GG	1	gg	0.464157501
NA19159	252	CC	CC	0.9292	cc	0.407349531
NA18859	260	CG	CG	0.996	cg	0.272303633

MACGT discrepancy
LDA discrepancy
LDA NN (0.65 threshold)

		7693776				
**Coriell**	**Sample**	**HapMap Call**	**LDA Call**	**LDA quality score**	**MACGT Call**	**MACGT quality score**
NA12753	1	TT	TT	0.9995	tt	0.24100675
NA12707	8	TT	TT	0.9998	tt	0.055303806
NA11839	16	TT	TT	0.9997	tt	0.010113799
NA10859	24	TT	TT	0.9985	tt	0.086497893
NA07034	32	TT	TT	0.9987	TT	0.233719569
NA07055	40	TT	TT	0.9996	TT	0.502079826
NA12814	48	TT	TT	0.9986	TT	0.445072684
NA10839	55	TT	TT	0.9927	tt	0.242264077
NA10847	56	TT	TT	0.998	tt	0.443010018
NA12717	63	TT	TT	0.9997	tt	0.381808361
NA10861	64	TT	TT	0.949	tt	0.000728235
NA12740	71	TT	TT	0.9896	tt	0.195953064
NA12005	72	TT	TT	0.9985	tt	0.481620673
NA12752	79	TT	TT	0.9984	tt	0.250956087
NA10851	80	TT	TT	0.9978	tt	0.161581071
NA12043	87	TT	TT	0.9947	tt	0.347851021
NA12264	95	TT	TT	0.9994	tt	0.339927404
NA18621	101	TT	TT	0.9992	tt	0.267795918
NA18594	109	TT	TT	0.9985	tt	0.296553304
NA18622	117	TT	TT	0.9984	tt	0.351046277
NA18573	125	TT	TT	0.9982	tt	0.348682817
NA18623	133	TT	TT	0.9992	tt	0.550708109
NA18576	141	TT	TT	0.9983	tt	0.383165057
NA18633	142	TT	TT	0.999	tt	0.450087814
NA18964	146	TT	TT	0.994	tt	0.31399658
NA18994	150	TT	TT	0.9986	tt	0.341271794
NA18953	154	TT	TT	0.9753	tt	0.083233178
NA18968	162	TT	TT	0.9987	tt	0.34240448
NA18992	166	TT	TT	0.9982	tt	0.267354738
NA18959	170	TT	TT	0.9992	tt	0.528888554
NA18995	174	TT	TT	0.9988	tt	0.227696388
NA18969	178	TT	TT	0.9973	TT	0.448221402
NA18997	182	TT	TT	0.9997	tt	0.246026898
NA18960	186	TT	TT	0.9959	tt	0.344158954
NA18502	192	TT	TT	0.9829	tt	0.197433397
NA18863	199	TT	TT	0.9995	tt	0.580102906
NA19145	200	TT	TT	0.9991	tt	0.354165533
NA18855	207	TT	TT	0.993	tt	0.472312504
NA18505	208	TT	TT	0.9969	tt	0.314535268
NA18862	215	TT	TT	0.7473	tt	0.031505794
NA18856	216	TT	TT	0.9743	tt	0.14850036
NA18503	220	TT	TT	0.9973	tt	0.247889659
NA19152	224	TT	TT	0.9992	tt	0.496144484
NA19210	228	TT	TT	0.9991	tt	0.342231137
NA19139	232	TT	TT	0.9983	tt	0.339066098
NA19204	236	TT	TT	0.9943	tt	0.252968679
NA18507	244	TT	TT	0.998	tt	0.427171288
NA19159	252	TT	TT	0.9935	TT	0.323920661
NA18859	260	TT	TT	0.9981	tt	0.504111978

MACGT discrepancy
LDA discrepancy
LDA NN (0.65 threshold)

		7855283				
Coriell	Sample	HapMap Call	LDA Call	LDA quality score	MACGT Call	MACGT quality score
NA12753	1	AA	AA	0.9997	aa	0.055084488
NA12707	8	AA	AA	0.9998	aa	0.022806885
NA11839	16	AG	AG	0.9999	ag	0
NA10859	24	AA	AA	1	aa	0.007157662
NA07034	32	AG	AG	0.9999	ag	0.001091234
NA07055	40	AA	AA	0.9998	AA	0.503802254
NA12814	48	GG	GG	1	GG	0.569986783
NA10839	55	AG	AG	0.9998	ag	0.037487923
NA10847	56	AA	AA	0.9999	aa	0.308149468
NA12717	63	AA	AA	1	aa	0.237798777
NA10861	64	GG	GG	0.9933	gg	0
NA12740	71	AA	AA	1	aa	0.196563826
NA12005	72	AA	AA	0.9998	AA	0.513728194
NA12752	79	GG	GG	1	GG	0.569986783
NA10851	80	AG	AG	0.9998	ag	0.223869548
NA12043	87	AA	AA	0.9999	aa	0.239768113
NA12264	95	AG	AG	0.9998	ag	0.261511592
NA18621	101	AG	AG	0.9995	ag	0.267472731
NA18594	109	AG	AG	0.9999	AG	0.523080478
NA18622	117	AG	AG	0.9998	AG	0.450702301
NA18573	125	AA	AA	0.9999	aa	0.122085026
NA18623	133	AG	AG	0.9999	AG	0.480924635
NA18576	141	AA	AA	1	AA	0.527280084
NA18633	142	AG	AG	1	ag	0.512304146
NA18964	146	AA	AA	0.9999	aa	0.357317983
NA18994	150	AG	AG	1	ag	0.184902438
NA18953	154	AA	AA	1	aa	0.177665831
NA18968	162	AG	AG	0.9999	ag	0.229971337
NA18992	166	AA	AA	0.9999	aa	0.074485276
NA18959	170	AG	AG	1	AG	0.505898436
NA18995	174	AG	AG	0.9999	ag	0.271744115
NA18969	178	AG	AG	0.9999	ag	0.44523301
NA18997	182	AG	AG	0.9999	ag	0.005314631
NA18960	186	AG	AG	0.9998	ag	0.234125594
NA18502	192	AG	AG	1	ag	0.104920199
NA18863	199	AA	AA	1	AA	0.578491936
NA19145	200	AG	AG	0.9999	ag	0.410178561
NA18855	207	AA	AA	1	aa	0.373467136
NA18505	208	AG	AG	1	ag	0.332115725
NA18862	215	AG	AG	0.9999	ag	0.055609554
NA18856	216	AA	AA	0.9999	aa	0.279557627
NA18503	220	AG	AG	0.9999	ag	0.124846978
NA19152	224	AA	AA	0.9999	aa	0.294653535
NA19210	228	AG	AG	1	ag	0.155033423
NA19139	232	AA	AA	0.9999	aa	0.230644122
NA19204	236	AA	AA	1	aa	0.082832939
NA18507	244	AA	AA	1	aa	0.333654285
NA19159	252	AG	AG	0.9999	ag	0.319092014
NA18859	260	AA	AA	1	aa	0.344702501

MACGT discrepancy
LDA discrepancy
LDA NN (0.65 threshold)

		8096868				
Coriell	Sample	HapMap Call	LDA Call	LDA quality score	MACGT Call	MACGT quality score
NA12753	1	CT	CT	0.9998	ct	0.020820455
NA12707	8	TT	TT	0.9992	tt	0.20430919
NA11839	16	TT	TT	0.998	tt	0.180139558
NA10859	24	CT	CT	0.9999	ct	0.005683318
NA07034	32	TT	TT	0.9992	TT	0.312983011
NA07055	40	TT	TT	0.9999	TT	0.586961605
NA12814	48	CT	CT	0.9996	CT	0.455413711
NA10839	55	CC	CC	1	cc	0.008702676
NA10847	56	TT	TT	1	tt	0.331090241
NA12717	63	CT	CT	0.9083	ct	0.139728194
NA10861	64	TT	TT	0.9994	tt	0.014696489
NA12740	71	TT	TT	0.9999	TT	0.439213575
NA12005	72	CC	CC	1	cc	0.299321209
NA12752	79	CT	CT	1	ct	0.38622223
NA10851	80	CT	CT	1	CT	0.459390236
NA12043	87	TT	TT	0.9999	tt	0.415161261
NA12264	95	TT	TT	0.9998	tt	0.312623675
NA18621	101	CT	CT	0.9996	ct	0.349427919
NA18594	109	CC	CC	1	CC	0.508759228
NA18622	117	CC	CC	1	cc	0.238053866
NA18573	125	CC	CC	1	cc	0.055894128
NA18623	133	CC	CC	1	cc	0.064867998
NA18576	141	TT	TT	0.9995	TT	0.57141106
NA18633	142	TT	TT	0.9998	tt	0.506359843
NA18964	146	CC	CC	1	CC	0.508706283
NA18994	150	CC	CC	1	CC	0.530314838
NA18953	154	CT	CT	0.9941	ct	0.099582808
NA18968	162	CT	CT	1	ct	0.069904594
NA18992	166	CC	CC	1	cc	0.29056872
NA18959	170	CC	CC	1	CC	0.571141145
NA18995	174	CC	CC	1	cc	0.25762575
NA18969	178	CT	CT	1	ct	0.209052876
NA18997	182	CC	CC	1	cc	0.012774188
NA18960	186	TT	TT	0.9999	tt	0.209857439
NA18502	192	TT	TT	1	tt	0.352065093
NA18863	199	CT	CT	0.9988	ct	0.345467332
NA19145	200	TT	TT	0.9999	tt	0.324787044
NA18855	207	TT	TT	0.9999	tt	0.489741126
NA18505	208	TT	TT	1	tt	0.303122375
NA18862	215	CT	CT	0.9977	CT	0.469767004
NA18856	216	TT	TT	1	tt	0.228344085
NA18503	220	TT	TT	0.9999	tt	0.412660927
NA19152	224	CT	CT	0.9919	ct	0.285352904
NA19210	228	CT	CT	0.5872	ct	0.043087885
NA19139	232	CT	CT	0.9999	ct	0.243523435
NA19204	236	TT	TT	0.9999	tt	0.201011434
NA18507	244	TT	TT	0.9999	tt	0.600081967
NA19159	252	CT	CT	0.9956	CT	0.369712238
NA18859	260	TT	TT	0.9998	tt	0.308355697

MACGT discrepancy
LDA discrepancy
LDA NN (0.65 threshold)

		12426585				
Coriell	Sample	HapMap Call	LDA Call	LDA quality score	MACGT Call	MACGT quality score
NA12753	1	CT	CT	0.9998	ct	0.053503958
NA12707	8	CT	CT	0.9993	ct	0.031312924
NA11839	16	CT	CT	1	ct	0.002430714
NA10859	24	CT	CT	1	ct	0.007429939
NA07034	32	TT	TT	1	TT	0.316851021
NA07055	40	CT	CT	0.8709	ct	0.038279354
NA12814	48	CT	CT	1	CT	0.412680806
NA10839	55	TT	TT	1	TT	0.475041174
NA10847	56	CT	CT	1	ct	0.405541572
NA12717	63	CT	CT	1	ct	0.329458626
NA10861	64	CT	CT	0.9999	ct	0.095082639
NA12740	71	CT	CT	1	ct	0.461172066
NA12005	72	CT	CT	1	ct	0.242746456
NA12752	79	TT	TT	1	tt	0.490896125
NA10851	80	TT	TT	1	TT	0.580378782
NA12043	87	TT	TT	1	tt	0.349369928
NA12264	95	CT	CT	1	ct	0.428013914
NA18621	101	CC	CC	1	CC	0.37033574
NA18594	109	CC	CC	1	cc	0.225890734
NA18622	117	CT	CT	1	CT	0.656803845
NA18573	125	CC	CC	1	cc	0.157504958
NA18623	133	CC	CC	1	CC	0.590006019
NA18576	141	CC	CC	1	CC	0.570171724
NA18633	142	CC	CC	1	cc	0.244714552
NA18964	146	CT	CT	0.9969	CT	0.46909112
NA18994	150	TT	TT	1	tt	0.449811596
NA18953	154	CT	CT	1	ct	0.314167288
NA18968	162	CT	CT	0.993	ct	0.206808402
NA18992	166	TT	TT	1	tt	0.470597904
NA18959	170	TT	TT	1	TT	0.473502791
NA18995	174	CT	CT	0.9993	ct	0.291978521
NA18969	178	CT	CT	1	ct	0.233887148
NA18997	182	TT	TT	1	tt	0.36375746
NA18960	186	CT	CT	1	CT	0.484962228
NA18502	192	TT	TT	1	tt	0.137996413
NA18863	199	CT	CT	1	ct	0.271018808
NA19145	200	CT	CT	1	ct	0.510301914
NA18855	207	CT	CT	1	ct	0.440330884
NA18505	208	CC	CC	1	cc	0
NA18862	215	CT	CT	1	ct	0.416179745
NA18856	216	CT	CT	1	ct	0.114142464
NA18503	220	CC	CC	1	CC	0.554125043
NA19152	224	CC	CC	1	cc	0.481665677
NA19210	228	CC	CC	0.9948	cc	0.059288239
NA19139	232	TT	TT	1	tt	0.455365138
NA19204	236	CC	CC	0.8016	cc	0.014066597
NA18507	244	CT	CT	1	ct	0.473998079
NA19159	252	CC	CC	1	cc	0.167376116
NA18859	260	CC	CC	1	cc	0.113348464

MACGT discrepancy
LDA discrepancy
LDA NN (0.65 threshold)

		12466929				
Coriell	Sample	HapMap Call	LDA Call	LDA quality score	MACGT Call	MACGT quality score
NA12753	1	GG	GG	1	gg	0.236703936
NA12707	8	AG	AG	0.9514	ag	0
NA11839	16	GG	GG	1	gg	0.19403334
NA10859	24	GG	GG	1	gg	0.336871345
NA07034	32	GG	GG	1	GG	0.451956931
NA07055	40	GG	GG	1	GG	0.690492566
NA12814	48	GG	GG	1	gg	0.464124743
NA10839	55	GG	GG	1	GG	0.641441442
NA10847	56	GG	GG	1	gg	0.486542347
NA12717	63	GG	GG	1	gg	0.25987427
NA10861	64	GG	GG	0.75	gg	0.019627769
NA12740	71	AG	AG	0.9995	ag	0.222340299
NA12005	72	GG	GG	0.9104	gg	0.05016362
NA12752	79	GG	GG	1	gg	0.23526049
NA10851	80	GG	GG	1	gg	0.240496122
NA12043	87	GG	GG	0.75	gg	0.053261901
NA12264	95	AG	AG	0.9998	ag	0.337511301
NA18621	101	AA	AA	0.9972	AA	0.601014631
NA18594	109	AG	AG	0.9995	AG	0.589394135
NA18622	117	AA	AA	0.9998	aa	0.204232306
NA18573	125	AG	AG	0.9999	ag	0.28549314
NA18623	133	AG	AG	0.9998	ag	0.563902517
NA18576	141	AA	AA	0.9999	aa	0.191608487
NA18633	142	AG	AG	0.9999	AG	0.692917107
NA18964	146	AG	AG	0.9996	ag	0.207697412
NA18994	150	AG	AG	0.9999	ag	0.143628562
NA18953	154	AG	AG	0.9989	ag	0.251765879
NA18968	162	AG	AG	0.9998	ag	0.188237449
NA18992	166	AA	AA	0.9999	AA	0.508953328
NA18959	170	AA	AA	0.999	AA	0.657515866
NA18995	174	AG	AG	0.9996	ag	0.521997791
NA18969	178	AG	AG	0.9994	AG	0.574849254
NA18997	182	AG	AG	0.9997	ag	0.004517809
NA18960	186	GG	GG	0.9324	gg	0.022284659
NA18502	192	AG	AG	0.9996	ag	0.150579119
NA18863	199	GG	GG	1	gg	0.544296636
NA19145	200	GG	GG	0.7929	gg	0.063347446
NA18855	207	AG	AG	0.9997	ag	0.260285647
NA18505	208	AG	AG	0.9999	ag	0.48282435
NA18862	215	AG	AG	0.9988	ag	0.096445082
NA18856	216	AG	AG	0.9996	ag	0.182206074
NA18503	220	GG	GG	1	GG	0.322221368
NA19152	224	GG	GG	1	gg	0.194619953
NA19210	228	GG	GG	0.75	gg	0.012892282
NA19139	232	GG	GG	0.7524	gg	0.066556261
NA19204	236	GG	GG	0.75	gg	0.017719636
NA18507	244	AG	AG	0.9997	ag	0.156966886
NA19159	252	GG	GG	1	gg	0.353526165
NA18859	260	AG	AG	0.9996	AG	0.365843036

MACGT discrepancy
LDA discrepancy
LDA NN (0.65 threshold)

		12472674				
Coriell	Sample	HapMap Call	LDA Call	LDA quality score	MACGT Call	MACGT quality score
NA12753	1	TT	TT	1	tt	0.051965772
NA12707	8	CT	CT	0.8263	ct	0.004729372
NA11839	16	CC	CC	1	cc	0.009801616
NA10859	24	TT	TT	0.9887	tt	0.165816899
NA07034	32	TT	TT	0.9996	TT	0.377176712
NA07055	40	CT	CT	0.9773	ct	0.314966392
NA12814	48	CC	CC	1	CC	0.510922315
NA10839	55	CT	CT	0.9974	ct	0.046645504
NA10847	56	CT	CT	0.9999	ct	0.285729972
NA12717	63	TT	TT	1	tt	0.118446328
NA10861	64	CC	CC	0.5264	cc	0.115153112
NA12740	71	TT	TT	1	tt	0.286185906
NA12005	72	CT	CT	1	ct	0.091688042
NA12752	79	CC	CC	1	cc	0.323140178
NA10851	80	TT	TT	1	TT	0.529992534
NA12043	87	CT	CT	0.9997	ct	0.654143385
NA12264	95	CC	CC	0.9949	cc	0.006098683
NA18621	101	CT	CC	0.9995	**cc**	**0.005282608**
NA18594	109	TT	TT	1	TT	0.561126458
NA18622	117	CC	CC	0.9997	CC	0.613032294
NA18573	125	TT	TT	1	TT	0.590521783
NA18623	133	CC	CC	0.9998	CC	0.53285985
NA18576	141	CT	CT	0.9992	CT	0.440563717
NA18633	142	CT	CT	0.9998	ct	0.527885332
NA18964	146	CT	CT	0.9996	CT	0.565155852
NA18994	150	CT	CT	0.9999	ct	0.350320284
NA18953	154	TT	TT	1	tt	0.053209294
NA18968	162	CC	CC	0.9999	cc	0.044542381
NA18992	166	TT	TT	1	tt	0.112022814
NA18959	170	TT	TT	1	tt	0.424738427
NA18995	174	CC	CC	0.9971	cc	0.179514824
NA18969	178	TT	TT	1	tt	0.211979697
NA18997	182	TT	TT	0.7498	tt	0
NA18960	186	CT	CT	0.9998	CT	0.574160781
NA18502	192	CC	CC	0.9956	cc	0.183843611
NA18863	199	CC	CC	1	cc	0.305472663
NA19145	200	CC	CC	0.9977	cc	0.348944074
NA18855	207	CT	CT	0.995	ct	0.158701427
NA18505	208	CC	CC	0.9392	cc	0.098093111
NA18862	215	CC	CC	0.9983	cc	0.085110529
NA18856	216	CT	CT	0.999	ct	0.397965995
NA18503	220	CC	CC	0.9837	CC	0.43518866
NA19152	224	CC	CC	0.9994	cc	0.618929691
NA19210	228	CT	CT	0.9999	CT	0.410318679
NA19139	232	CC	CC	0.9831	cc	0.526502582
NA19204	236	CT	CT	0.9998	ct	0.236926847
NA18507	244	CC	CC	1	cc	0.498292567
NA19159	252	CC	CC	1	cc	0.432221683
NA18859	260	TT	TT	1	tt	0.492617043

MACGT discrepancy
LDA discrepancy
LDA NN (0.65 threshold)

		12583473				
Coriell	Sample	HapMap Call	LDA Call	LDA quality score	MACGT Call	MACGT quality score
NA12753	1	GG	GG	1	gg	0.29235514
NA12707	8	GG	GG	1	gg	0.387894356
NA11839	16	GG	GG	1	gg	0.183806253
NA10859	24	GG	GG	1	gg	0.199237642
NA07034	32	GG	GG	1	GG	0.366862091
NA07055	40	GG	GG	1	gg	0.591143342
NA12814	48	GG	GG	1	gg	0.55341098
NA10839	55	GG	GG	1	gg	0.340188786
NA10847	56	GG	GG	1	gg	0.329339558
NA12717	63	GG	GG	1	gg	0.385553659
NA10861	64	GG	GG	1	gg	0.481489822
NA12740	71	GG	GG	1	gg	0.322584265
NA12005	72	GG	GG	1	gg	0.381142771
NA12752	79	GG	GG	1	gg	0.537595088
NA10851	80	GG	GG	1	GG	0.556297371
NA12043	87	GG	GG	1	gg	0.435804685
NA12264	95	GG	GG	1	gg	0.464126597
NA18621	101	CG	CG	1	cg	0.238371463
NA18594	109	CG	CG	1	CG	0.480984416
NA18622	117	CG	CG	0.75	cg	0
NA18573	125	GG	GG	1	GG	0.716791104
NA18623	133	CG	CG	1	CG	0.509732796
NA18576	141	CG	CG	1	cg	0.373473239
NA18633	142	CG	CG	1	CG	0.637600593
NA18964	146	CG	CG	1	CG	0.477007935
NA18994	150	GG	GG	1	gg	0.643872542
NA18953	154	GG	GG	1	gg	0.476949028
NA18968	162	CG	CG	1	cg	0.204164854
NA18992	166	GG	GG	1	gg	0.548067138
NA18959	170	CC	CC	1	CC	0.696018398
NA18995	174	GG	GG	1	gg	0.504080141
NA18969	178	CG	CG	1	cg	0.347715016
NA18997	182	CG	CG	1	cg	0.007602537
NA18960	186	GG	GG	1	GG	0.505120147
NA18502	192	GG	GG	1	gg	0.325184582
NA18863	199	GG	GG	1	gg	0.502282829
NA19145	200	CG	CG	1	cg	0.542279899
NA18855	207	GG	GG	1	gg	0.637892657
NA18505	208	CG	CG	1	cg	0.552870369
NA18862	215	GG	GG	1	gg	0.3492406
NA18856	216	CG	CG	1	cg	0.501484629
NA18503	220	CC	CC	1	CC	0.696018398
NA19152	224	CG	CG	1	cg	0.527738405
NA19210	228	CG	CG	1	cg	0.264678121
NA19139	232	GG	GG	1	gg	0.629361389
NA19204	236	GG	GG	1	gg	0.286796439
NA18507	244	GG	GG	1	gg	0.373097067
NA19159	252	GG	GG	1	gg	0.531840696
NA18859	260	GG	GG	1	gg	0.497287026
