# Supplementary material for: Robust SNP genotyping by multiplex PCR and arrayed primer extension
Source: BMC Med Genomics. 2008 Jan 31;1:5. doi: 10.1186/1755-8794-1-5 (PMC2266772; doi:10.1186/1755-8794-1-5)
Supplement: Additional file 9 — Performance analyses for the different data sets, addressing the redundant probe chemistry. To further determine the effect of probe redundancy in our APEX methodology, we used LDA to reanalyze both data sets (original and 50-plex) but using non-redundant and partially-redundant probe-specific data. Three tables are shown (8, 9 and 10). [file 1755-8794-1-5-S9.doc]

**Additional file 9: 50-plex HapMap samples on 50 SNPs using smaller training set including three negative control samples**

| **Genotyping Model**  **(Classifiers)** | **0 threshold** | | **0.65 threshold** | |
| --- | --- | --- | --- | --- |
|  | Call rate | Miss-classification rate | Call rate | Miss-classification rate |
| APEX.L; APEX.R; ASO.L and ASO.R | 100 | 0.12* | 99.26 | 0.12* |
| APEX {APEX.L, APEX.R} | 100 | 0.18 | 97.28 | 0.12* |
| ASO {ASO.L, ASO.R} | 99.50 | 1.42 | 96.33 | 0.72 |
| LEFT {APEX.L, ASO.L} | 99.51 | 0.96 | 95.92 | 0.30 |
| ***RIGHT {APEX.R, ASO.R}*** | ***99.67*** | ***0.24*** | ***97.93*** | ***0.12**** |
| APEX.L | 99.37 | 2.52 | 98.27 | 2.04 |
| APEX.R | 99.72 | 1.98 | 96.88 | 1.38 |
| ASO.L | 99.32 | 2.40 | 98.66 | 2.10 |
| ***ASO.R*** | ***99.28*** | ***1.38*** | ***97.50*** | ***0.96*** |

*The only two discrepancies occurred due to the presence of hidden SNPs within the PCR primer sites. Otherwise, manual inspection of the data corresponding to those two cases was completely agreeable with the predicted genotypes by the automated genotype calling algorithm.
