# Supplementary material for: Robust SNP genotyping by multiplex PCR and arrayed primer extension
Source: BMC Med Genomics. 2008 Jan 31;1:5. doi: 10.1186/1755-8794-1-5 (PMC2266772; doi:10.1186/1755-8794-1-5)
Supplement: Additional file 10 — Performance analyses for the different data sets, addressing the redundant probe chemistry. To further determine the effect of probe redundancy in our APEX methodology, we used LDA to reanalyze both data sets (original and 50-plex) but using non-redundant and partially-redundant probe-specific data. Three tables are shown (8, 9 and 10). [file 1755-8794-1-5-S10.doc]

**Additional file 10: 50-plex HapMap samples on 50 SNPs using minimal training set including three negative control samples**

| **Genotyping Model**  **(Classifiers)** | **0 threshold** | | **0.65 threshold** | |
| --- | --- | --- | --- | --- |
|  | Call rate | Miss-classification rate | Call rate | Miss-classification rate |
| APEX.L; APEX.R; ASO.L and ASO.R | 99.61 | 0.10* | 96.96 | 0.10 |
| APEX {APEX.L, APEX.R} | 99.02 | 0.70 | 93.87 | 0.10 |
| ASO {ASO.L, ASO.R} | 98.57 | 4.80 | 87.1 | 1.00 |
| LEFT {APEX.L, ASO.L} | 98.67 | 3.20 | 91.54 | 0.85 |
| ***RIGHT {APEX.R, ASO.R}*** | ***98.39*** | ***1.05*** | ***91.28*** | ***0.25*** |
| APEX.L | 98.93 | 3.60 | 98.18 | 3.35 |
| APEX.R | 98.77 | 1.95 | 96.88 | 1.35 |
| ASO.L | 98.92 | 5.85 | 95.84 | 5.35 |
| ***ASO.R*** | ***98.34*** | ***5.20*** | ***95.30*** | ***4.90*** |

Note: in the previous two tables (9 and 10), for the genotyping models (‘Right{APEX.R, ASO.R}’ and ‘ASO.R’), the results are given for 49 SNPs.

There was a certain decline in performance levels while using the minimal training set for all genotyping models using different combinations of classifiers. This phenomenon was justified, since we only allowed 1 or 2 prototypes per class for each SNP, which may not be sufficient to build the exact and accurate genotype model using any sets of classifiers.

Note: for the 50-plex PCR HapMap samples, the smaller training sample had in total 510 cases (plus 3 negative controls which contributed 250 cases, NN) and the test data had 1,941 cases with validated genotypes. For the same set, the minimal training sample had in total 282 cases (plus 250 NNs) and the test data had 2,168 cases with validated genotypes.
