# Supplementary material for: Robust SNP genotyping by multiplex PCR and arrayed primer extension
Source: BMC Med Genomics. 2008 Jan 31;1:5. doi: 10.1186/1755-8794-1-5 (PMC2266772; doi:10.1186/1755-8794-1-5)
Supplement: Additional file 11 — Simple scatter plots for all 50 SNPs from 50-plex data set. For each plot the x-axis represents signal values for X allele and the y-axis represents signal values for Y allele. All values are in log scale. Magenta, green, blue and black coloured symbols denote the classes YY, YX, XX and NN (negative control samples), respectively. Plot (1) combines the two ASO-APEX Left probes (one for each allele); plot (2) combines the two ASO-APEX Right probes (one for each allele); plot (3) is for the APEX Left probe; plot (4) is for the APEX Right probe. The plots for SNPs rs3776720, rs12472674 and rs4739199 include labeled data-points for the individual Coriell samples that gave rise to discrepancies in genotype calling. [file 1755-8794-1-5-S11.pdf]

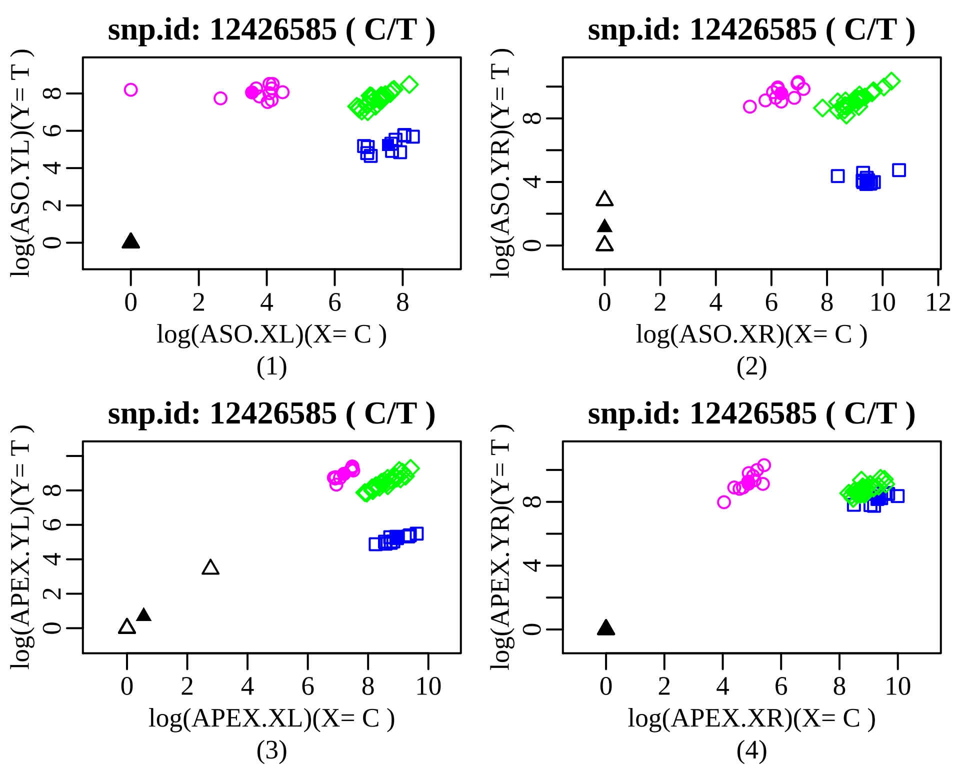
snp.id: 12426585 (C/T)
log(ASO.YL)(Y= T)
log(ASO.XL)(X= C)
(1)
snp.id: 12426585 (C/T)
log(ASO.YR)(Y= T)
log(ASO.XR)(X= C)
(2)
snp.id: 12426585 (C/T)
log(APEX.YL)(Y= T)
log(APEX.XL)(X= C)
(3)
snp.id: 12426585 (C/T)
log(APEX.YR)(Y= T)
log(APEX.XR)(X= C)
(4)

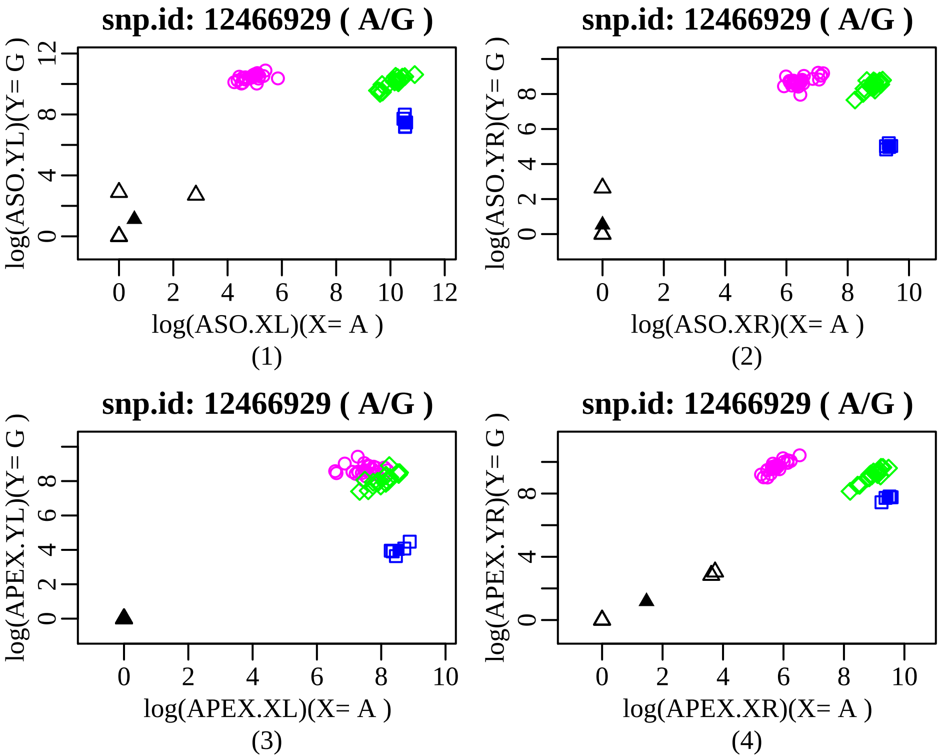
snp.id: 12466929 (A/G)
log(ASO.YL)(Y= G)
log(ASO.XL)(X= A)
(1)
snp.id: 12466929 (A/G)
log(ASO.YR)(Y= G)
log(ASO.XR)(X= A)
(2)
snp.id: 12466929 (A/G)
log(APEX.YL)(Y= G)
log(APEX.XL)(X= A)
(3)
snp.id: 12466929 (A/G)
log(APEX.YR)(Y= G)
log(APEX.XR)(X= A)
(4)

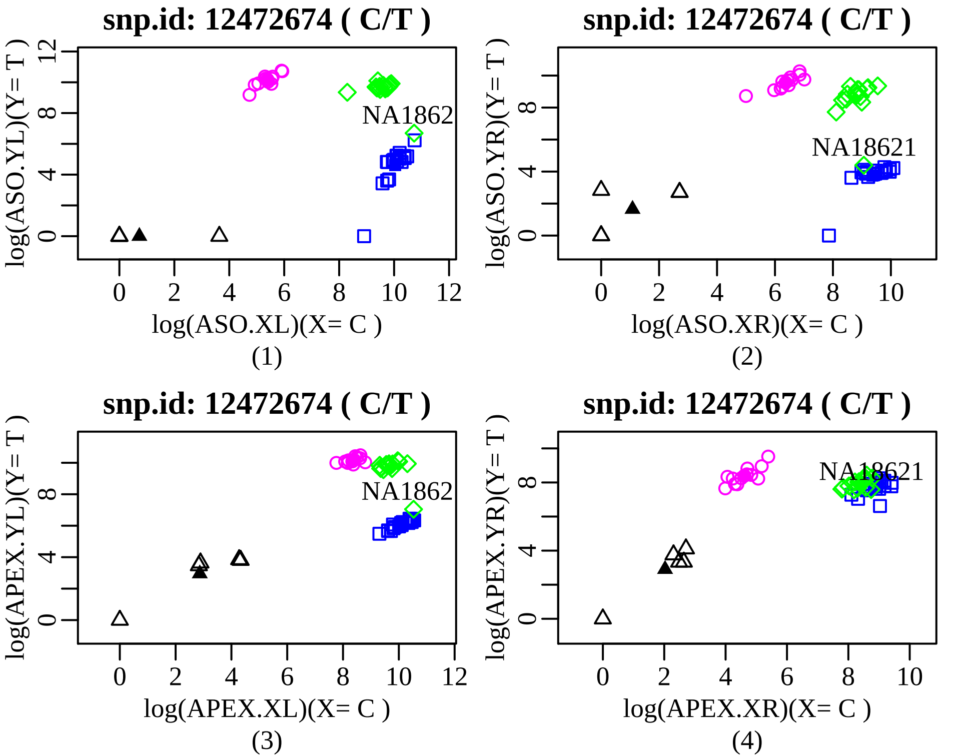
snp.id: 12472674 (C/T)
log(ASO.YL)(Y= T)
log(ASO.XL)(X= C)
NA1862
(1)
snp.id: 12472674 (C/T)
log(ASO.YR)(Y= T)
log(ASO.XR)(X= C)
NA18621
(2)
snp.id: 12472674 (C/T)
log(APEX.YL)(Y= T)
log(APEX.XL)(X= C)
NA1862
(3)
snp.id: 12472674 (C/T)
log(APEX.YR)(Y= T)
log(APEX.XR)(X= C)
NA18621
(4)

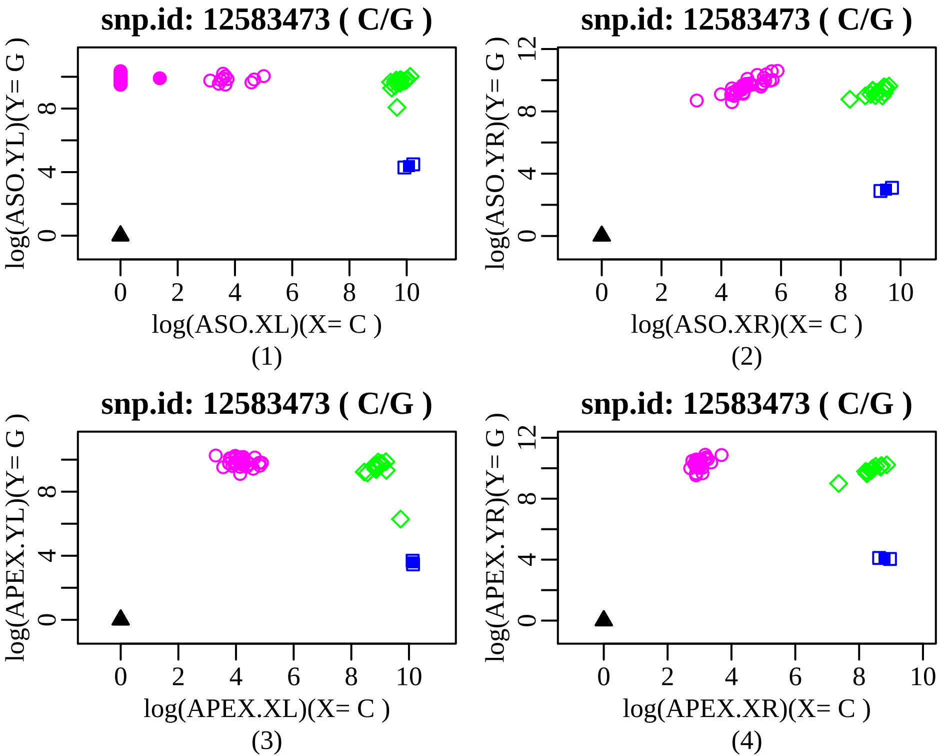
snp.id: 12583473 (C/G)
log(ASO.YL)(Y= G)
log(ASO.XL)(X= C)
(1)
snp.id: 12583473 (C/G)
log(ASO.YR)(Y= G)
log(ASO.XR)(X= C)
(2)
snp.id: 12583473 (C/G)
log(APEX.YL)(Y= G)
log(APEX.XL)(X= C)
(3)
snp.id: 12583473 (C/G)
log(APEX.YR)(Y= G)
log(APEX.XR)(X= C)
(4)

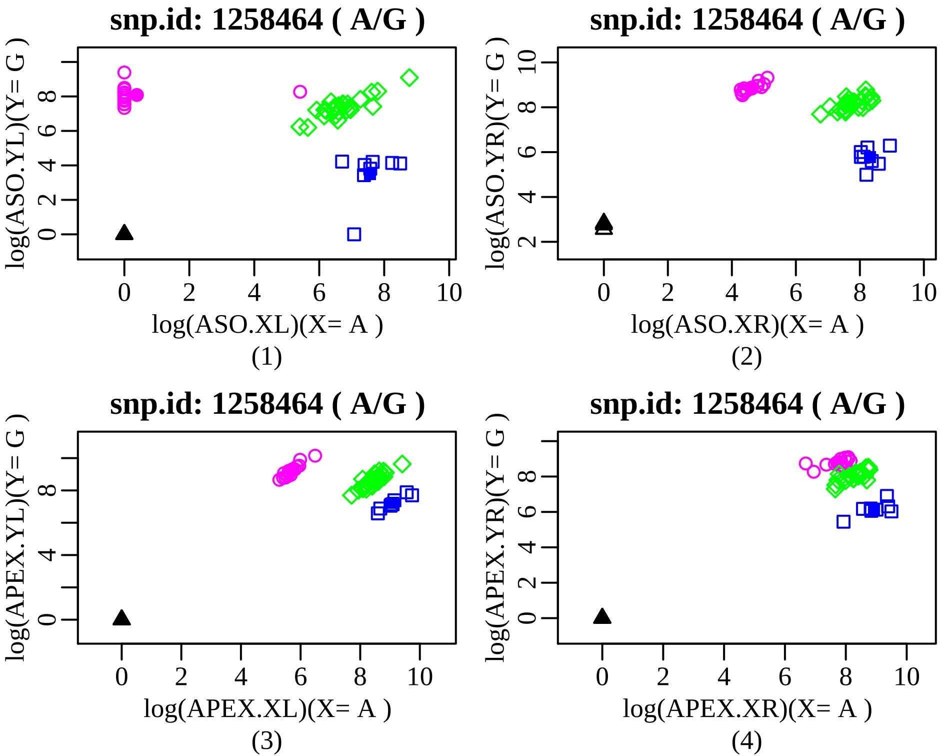
snp.id: 1258464 (A/G)
log(ASO.YL)(Y= G)
0 2 4 6 8
0 2 4 6 8 10
log(ASO.XL)(X= A)
(1)
snp.id: 1258464 (A/G)
log(ASO.YR)(Y= G)
2 4 6 8 10
0 2 4 6 8 10
log(ASO.XR)(X= A)
(2)
snp.id: 1258464 (A/G)
log(APEX.YL)(Y= G)
0 4 8
0 2 4 6 8 10
log(APEX.XL)(X= A)
(3)
snp.id: 1258464 (A/G)
log(APEX.YR)(Y= G)
0 2 4 6 8
0 2 4 6 8 10
log(APEX.XR)(X= A)
(4)

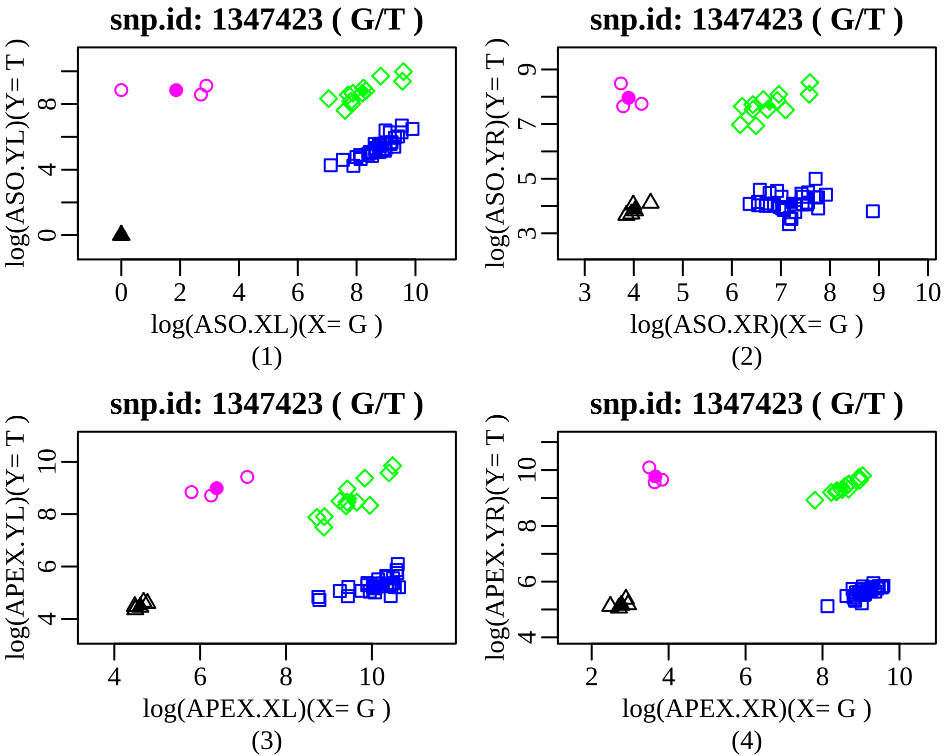
snp.id: 1347423 (G/T)
log(ASO.YL)(Y= T)
log(ASO.XL)(X= G)
(1)
snp.id: 1347423 (G/T)
log(ASO.YR)(Y= T)
log(ASO.XR)(X= G)
(2)
snp.id: 1347423 (G/T)
log(APEX.YL)(Y= T)
log(APEX.XL)(X= G)
(3)
snp.id: 1347423 (G/T)
log(APEX.YR)(Y= T)
log(APEX.XR)(X= G)
(4)

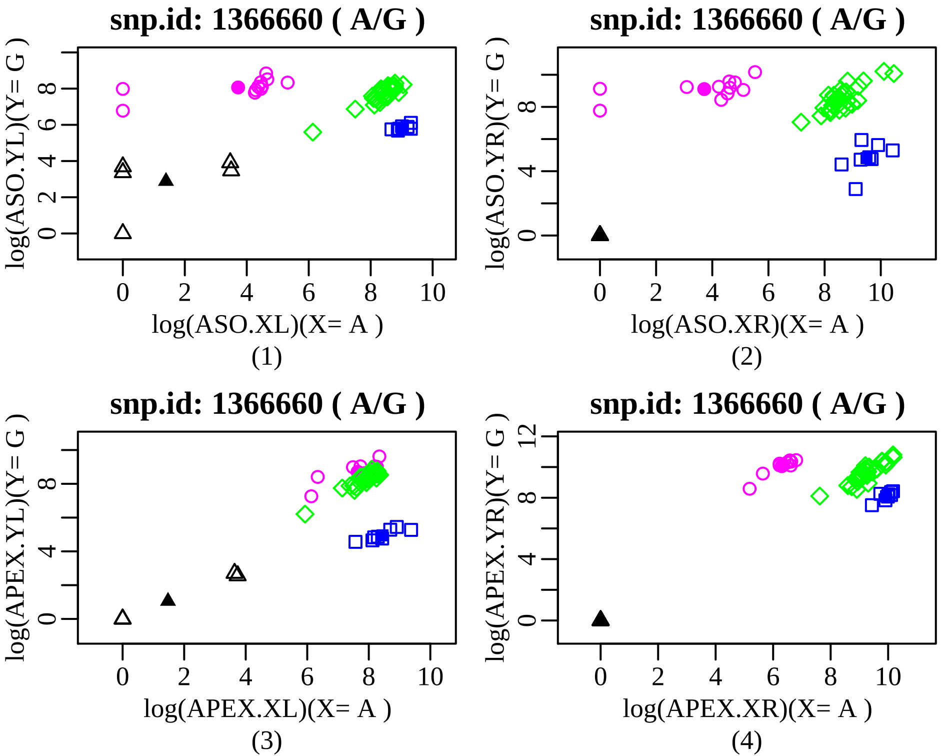
snp.id: 1366660 (A/G)
log(ASO.YL)(Y= G)
log(ASO.XL)(X= A)
0 2 4 6 8 10
0 2 4 6 8
(1)
snp.id: 1366660 (A/G)
log(ASO.YR)(Y= G)
log(ASO.XR)(X= A)
0 2 4 6 8 10
0 4 8
(2)
snp.id: 1366660 (A/G)
log(APEX.YL)(Y= G)
log(APEX.XL)(X= A)
0 2 4 6 8 10
0 4 8
(3)
snp.id: 1366660 (A/G)
log(APEX.YR)(Y= G)
log(APEX.XR)(X= A)
0 2 4 6 8 10
0 4 8 12
(4)

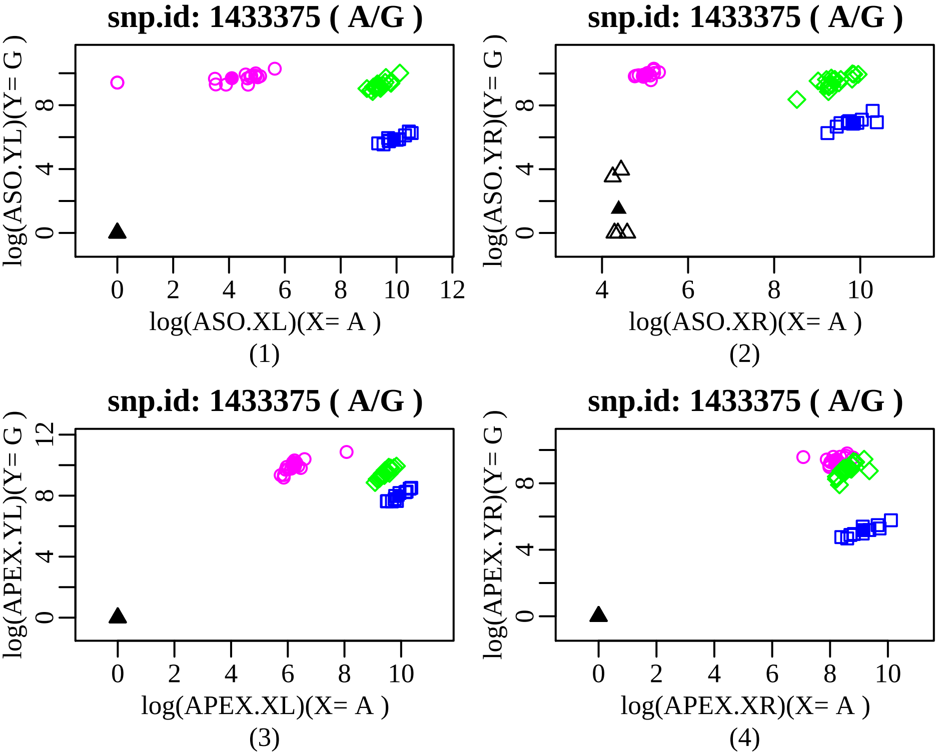
snp.id: 1433375 (A/G)
log(ASO.YL)(Y= G)
log(ASO.XL)(X= A)
(1)
snp.id: 1433375 (A/G)
log(ASO.YR)(Y= G)
log(ASO.XR)(X= A)
(2)
snp.id: 1433375 (A/G)
log(APEX.YL)(Y= G)
log(APEX.XL)(X= A)
(3)
snp.id: 1433375 (A/G)
log(APEX.YR)(Y= G)
log(APEX.XR)(X= A)
(4)

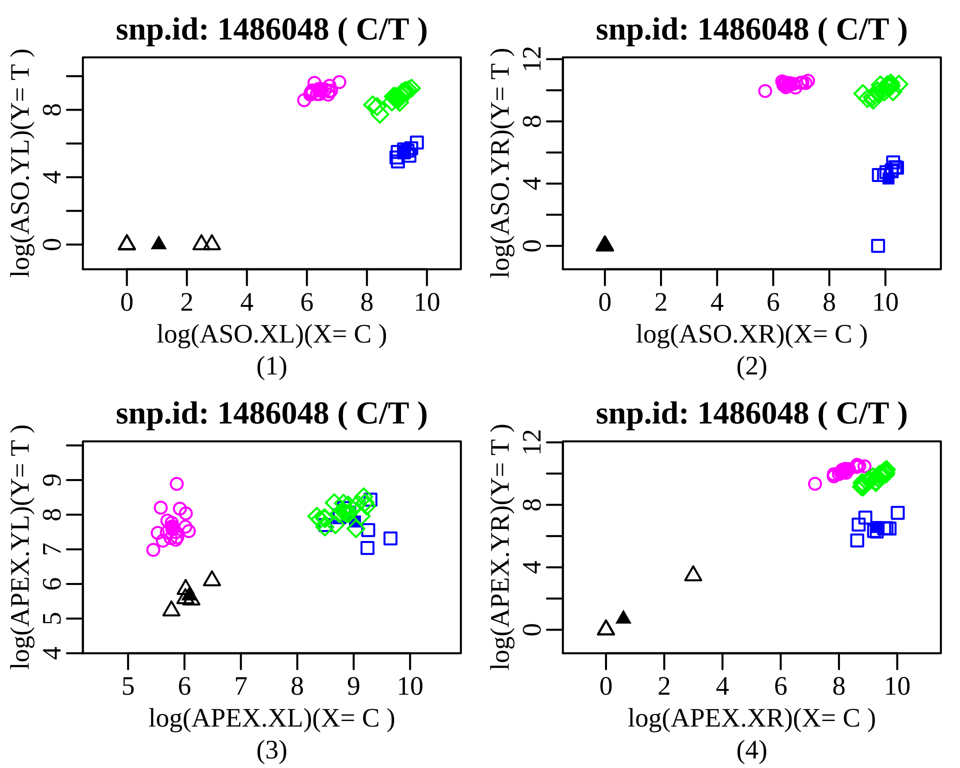
snp.id: 1486048 (C/T)
log(ASO.YL)(Y= T)
log(ASO.XL)(X= C)
(1)
snp.id: 1486048 (C/T)
log(ASO.YR)(Y= T)
log(ASO.XR)(X= C)
(2)
snp.id: 1486048 (C/T)
log(APEX.YL)(Y= T)
log(APEX.XL)(X= C)
(3)
snp.id: 1486048 (C/T)
log(APEX.YR)(Y= T)
log(APEX.XR)(X= C)
(4)

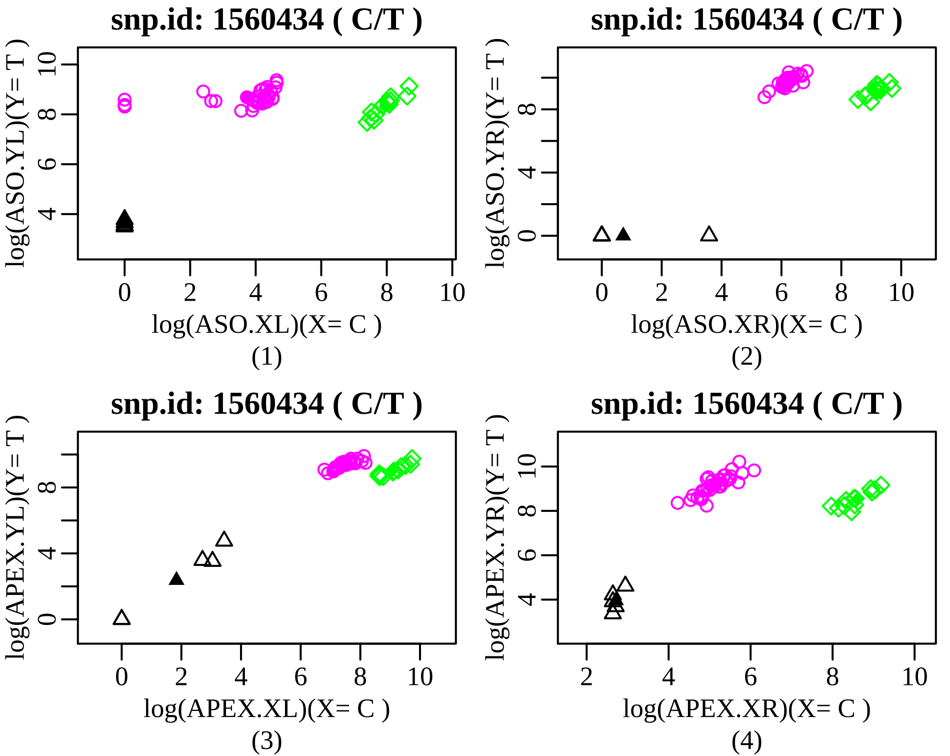
snp.id: 1560434 (C/T)
log(ASO.YL)(Y= T)
log(ASO.XL)(X= C)
(1)
snp.id: 1560434 (C/T)
log(ASO.YR)(Y= T)
log(ASO.XR)(X= C)
(2)
snp.id: 1560434 (C/T)
log(APEX.YL)(Y= T)
log(APEX.XL)(X= C)
(3)
snp.id: 1560434 (C/T)
log(APEX.YR)(Y= T)
log(APEX.XR)(X= C)
(4)

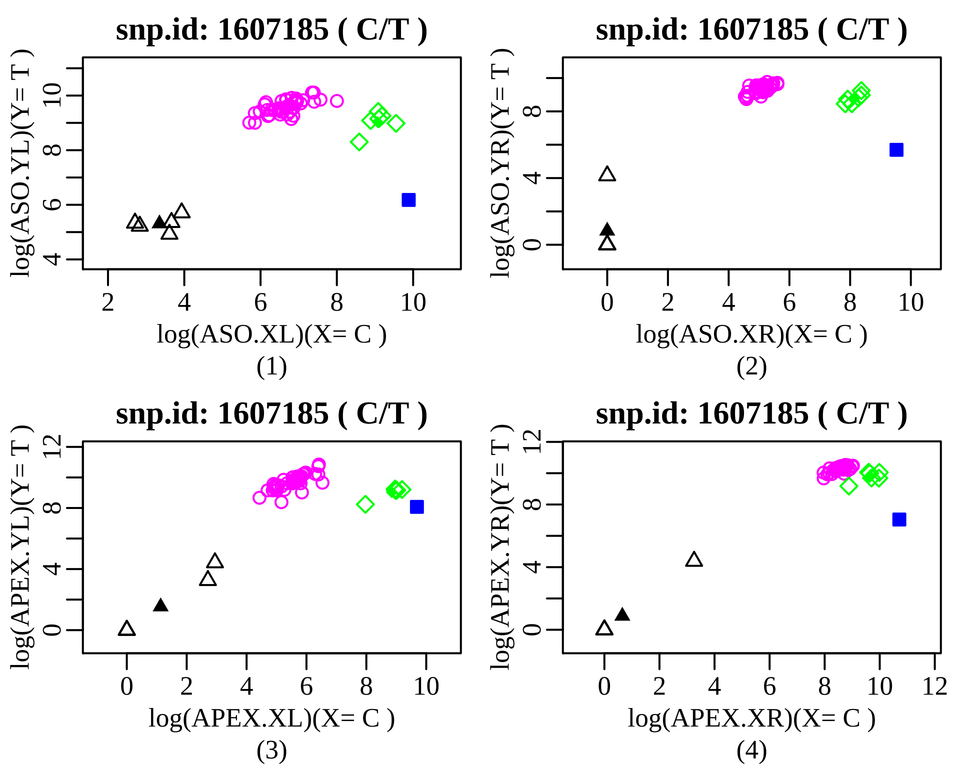
snp.id: 1607185 (C/T)
log(ASO.YL)(Y= T)
log(ASO.XL)(X= C)
(1)
snp.id: 1607185 (C/T)
log(ASO.YR)(Y= T)
log(ASO.XR)(X= C)
(2)
snp.id: 1607185 (C/T)
log(APEX.YL)(Y= T)
log(APEX.XL)(X= C)
(3)
snp.id: 1607185 (C/T)
log(APEX.YR)(Y= T)
log(APEX.XR)(X= C)
(4)

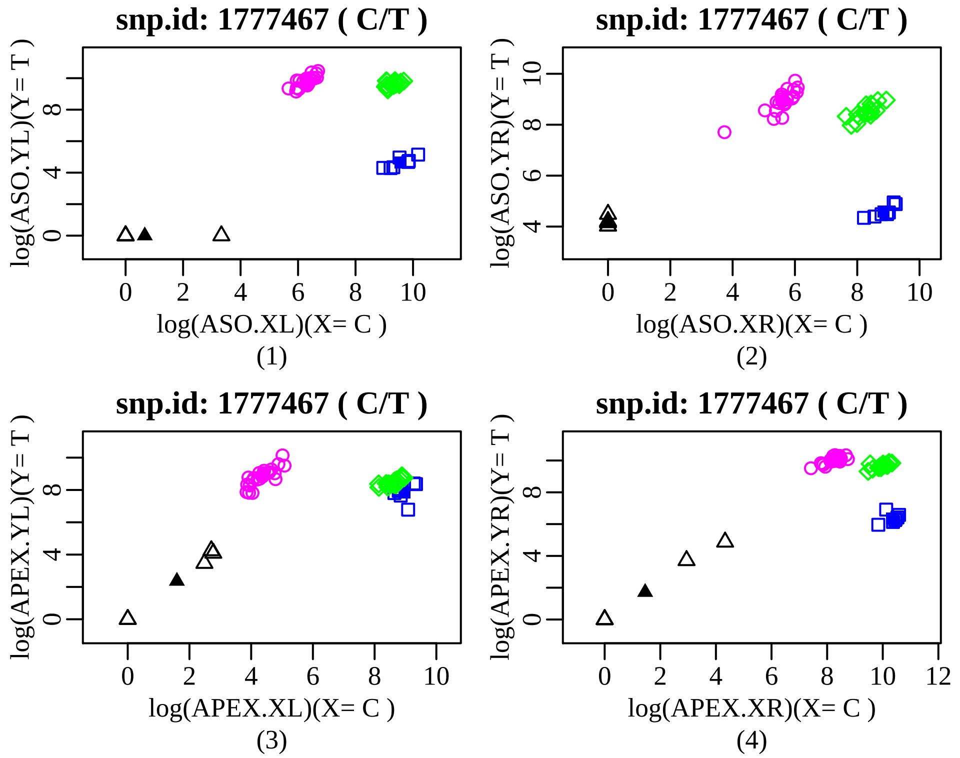
snp.id: 1777467 (C/T)
log(ASO.YL)(Y= T)
log(ASO.XL)(X= C)
(1)
snp.id: 1777467 (C/T)
log(ASO.YR)(Y= T)
log(ASO.XR)(X= C)
(2)
snp.id: 1777467 (C/T)
log(APEX.YL)(Y= T)
log(APEX.XL)(X= C)
(3)
snp.id: 1777467 (C/T)
log(APEX.YR)(Y= T)
log(APEX.XR)(X= C)
(4)

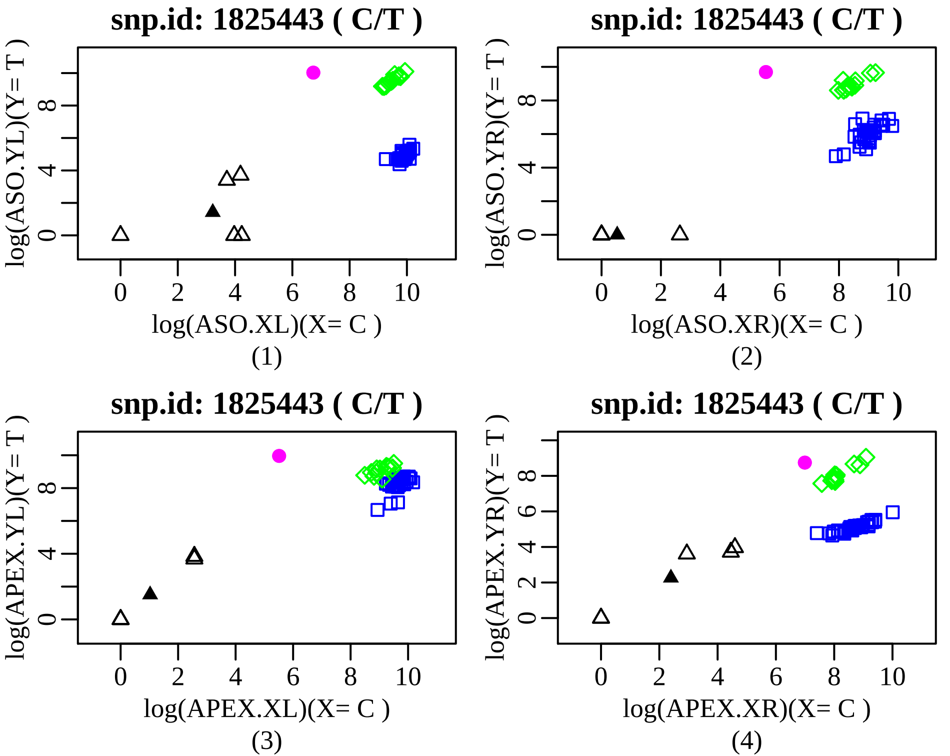
snp.id: 1825443 (C/T)
log(ASO.YL)(Y= T)
log(ASO.XL)(X= C)
(1)
snp.id: 1825443 (C/T)
log(ASO.YR)(Y= T)
log(ASO.XR)(X= C)
(2)
snp.id: 1825443 (C/T)
log(APEX.YL)(Y= T)
log(APEX.XL)(X= C)
(3)
snp.id: 1825443 (C/T)
log(APEX.YR)(Y= T)
log(APEX.XR)(X= C)
(4)

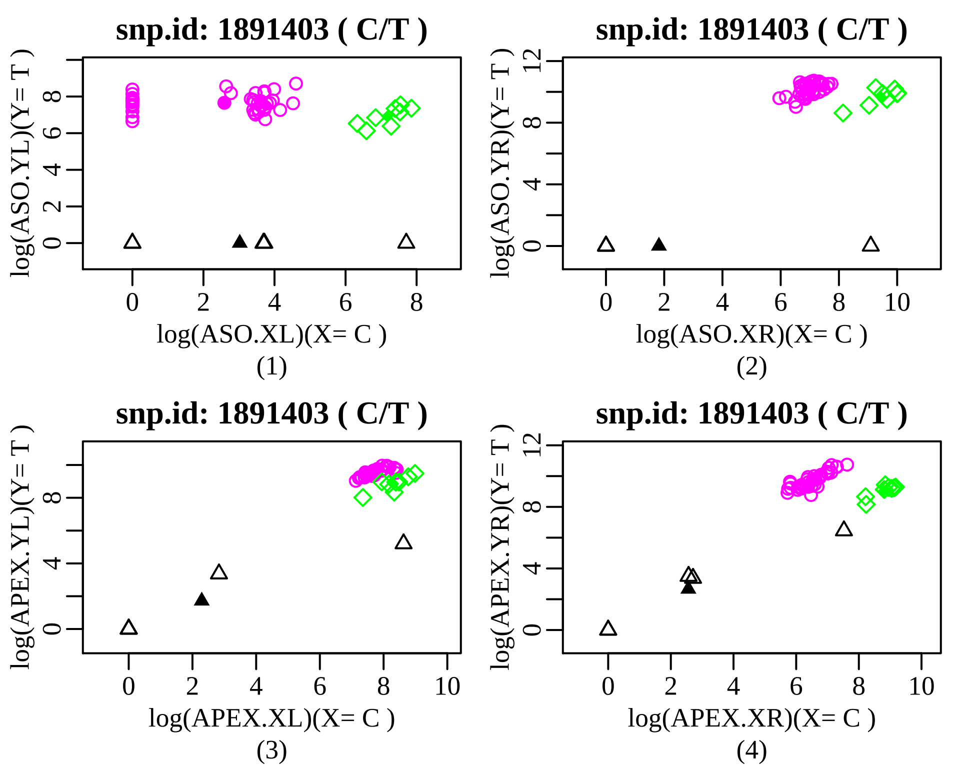
snp.id: 1891403 (C/T)
log(ASO.YL)(Y= T)
log(ASO.XL)(X= C)
(1)
snp.id: 1891403 (C/T)
log(ASO.YR)(Y= T)
log(ASO.XR)(X= C)
(2)
snp.id: 1891403 (C/T)
log(APEX.YL)(Y= T)
log(APEX.XL)(X= C)
(3)
snp.id: 1891403 (C/T)
log(APEX.YR)(Y= T)
log(APEX.XR)(X= C)
(4)

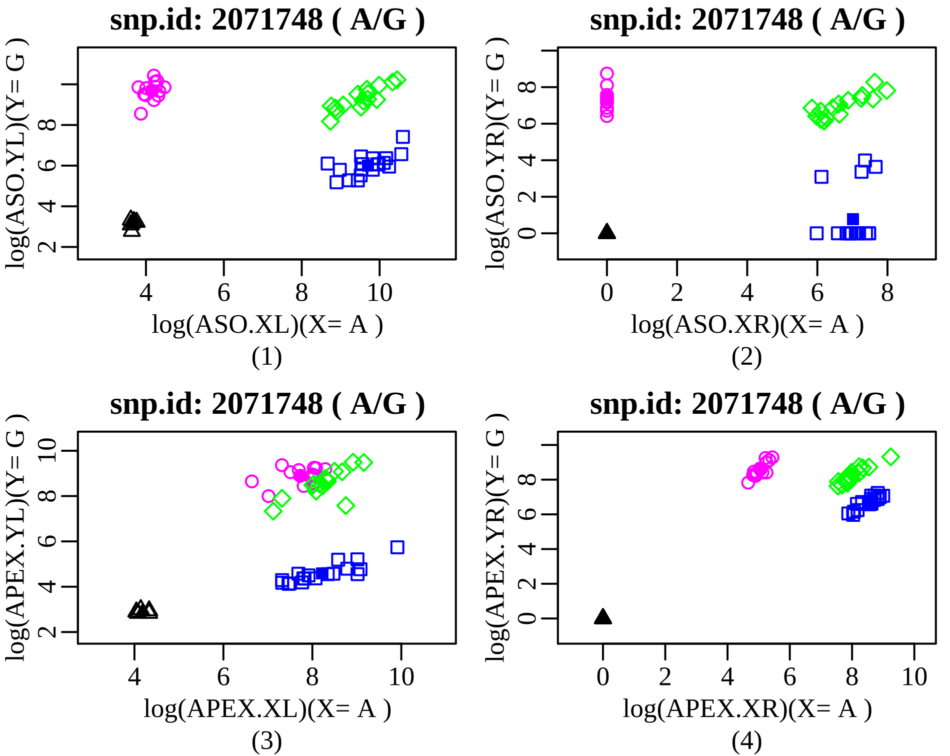
snp.id: 2071748 (A/G)
log(ASO.YL)(Y= G)
log(ASO.XL)(X= A)
(1)
snp.id: 2071748 (A/G)
log(ASO.YR)(Y= G)
log(ASO.XR)(X= A)
(2)
snp.id: 2071748 (A/G)
log(APEX.YL)(Y= G)
log(APEX.XL)(X= A)
(3)
snp.id: 2071748 (A/G)
log(APEX.YR)(Y= G)
log(APEX.XR)(X= A)
(4)

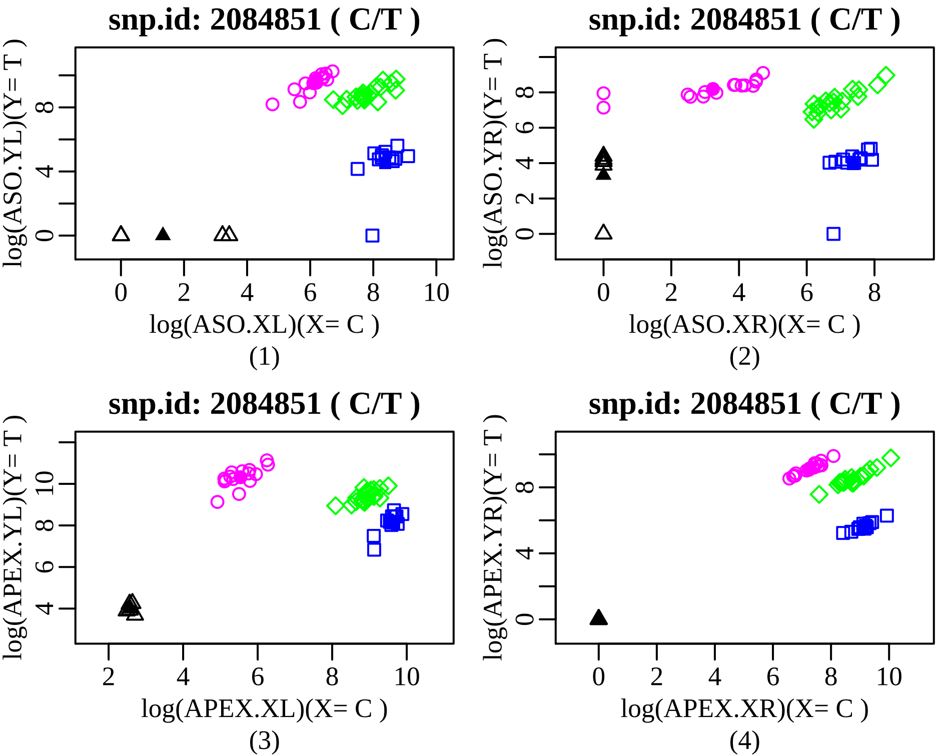
snp.id: 2084851 (C/T)
log(ASO.YL)(Y= T)
log(ASO.XL)(X= C)
(1)
snp.id: 2084851 (C/T)
log(ASO.YR)(Y= T)
log(ASO.XR)(X= C)
(2)
snp.id: 2084851 (C/T)
log(APEX.YL)(Y= T)
log(APEX.XL)(X= C)
(3)
snp.id: 2084851 (C/T)
log(APEX.YR)(Y= T)
log(APEX.XR)(X= C)
(4)

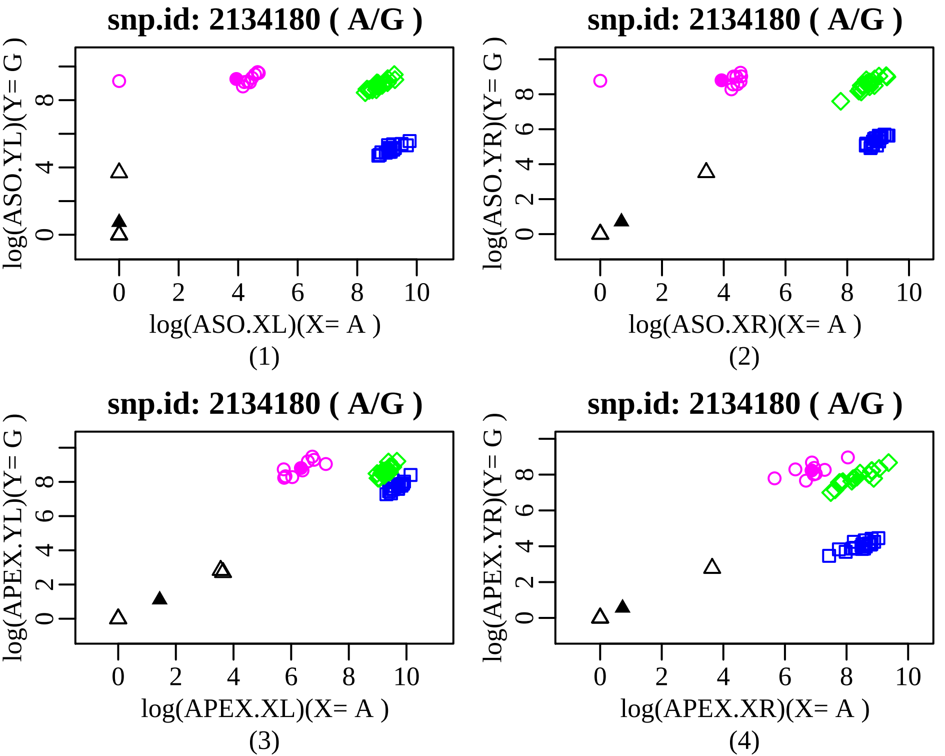
snp.id: 2134180 (A/G)
log(ASO.YL)(Y= G)
log(ASO.XL)(X= A)
(1)
snp.id: 2134180 (A/G)
log(ASO.YR)(Y= G)
log(ASO.XR)(X= A)
(2)
snp.id: 2134180 (A/G)
log(APEX.YL)(Y= G)
log(APEX.XL)(X= A)
(3)
snp.id: 2134180 (A/G)
log(APEX.YR)(Y= G)
log(APEX.XR)(X= A)
(4)

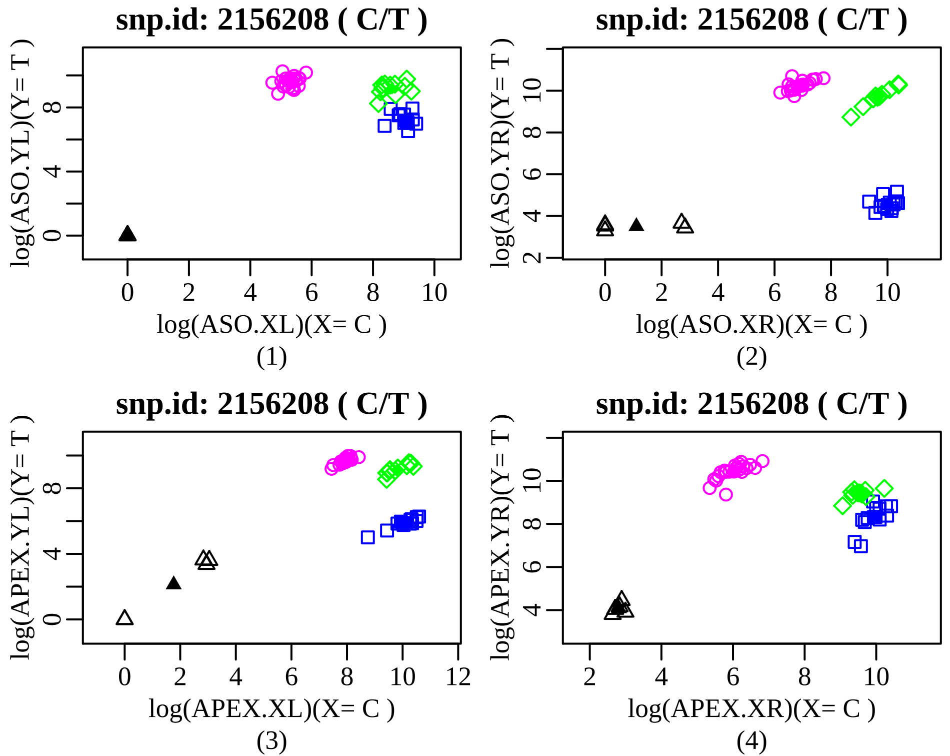
snp.id: 2156208 (C/T)
log(ASO.YL)(Y= T)
log(ASO.XL)(X= C)
0
4
8
0 2 4 6 8 10
(1)
snp.id: 2156208 (C/T)
log(ASO.YR)(Y= T)
log(ASO.XR)(X= C)
2 4 6 8 10
0 2 4 6 8 10
(2)
snp.id: 2156208 (C/T)
log(APEX.YL)(Y= T)
log(APEX.XL)(X= C)
0
4
8
0 2 4 6 8 10 12
(3)
snp.id: 2156208 (C/T)
log(APEX.YR)(Y= T)
log(APEX.XR)(X= C)
4 6 8 10
2 4 6 8 10
(4)

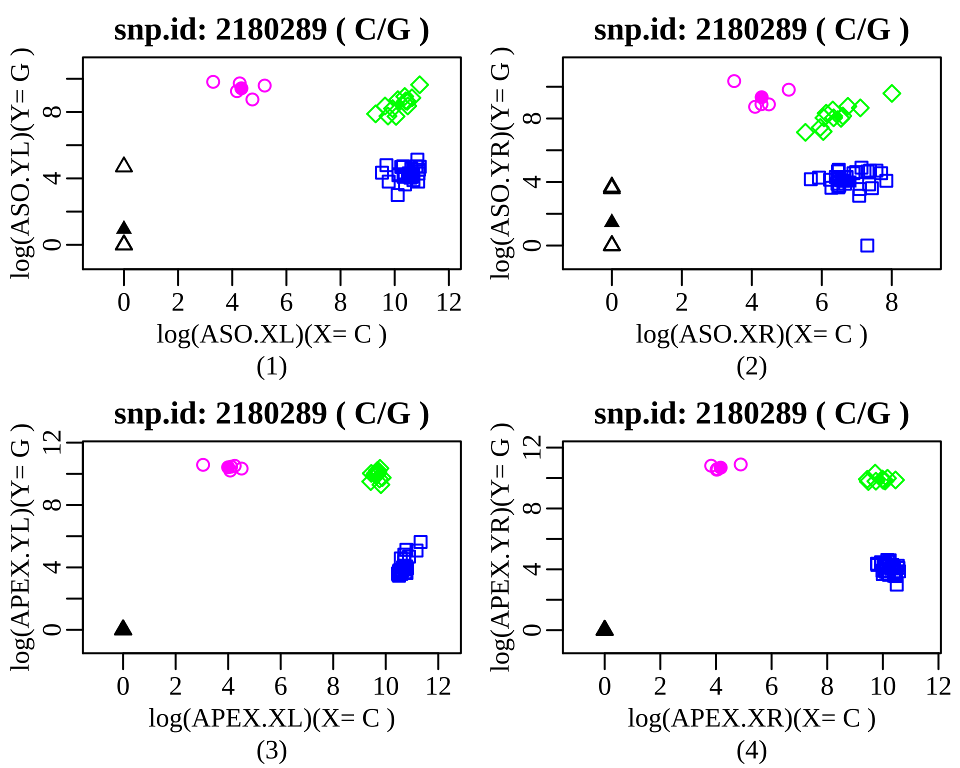
snp.id: 2180289 (C/G)
log(ASO.YL)(Y= G)
log(ASO.XL)(X= C)
(1)
snp.id: 2180289 (C/G)
log(ASO.YR)(Y= G)
log(ASO.XR)(X= C)
(2)
snp.id: 2180289 (C/G)
log(APEX.YL)(Y= G)
log(APEX.XL)(X= C)
(3)
snp.id: 2180289 (C/G)
log(APEX.YR)(Y= G)
log(APEX.XR)(X= C)
(4)

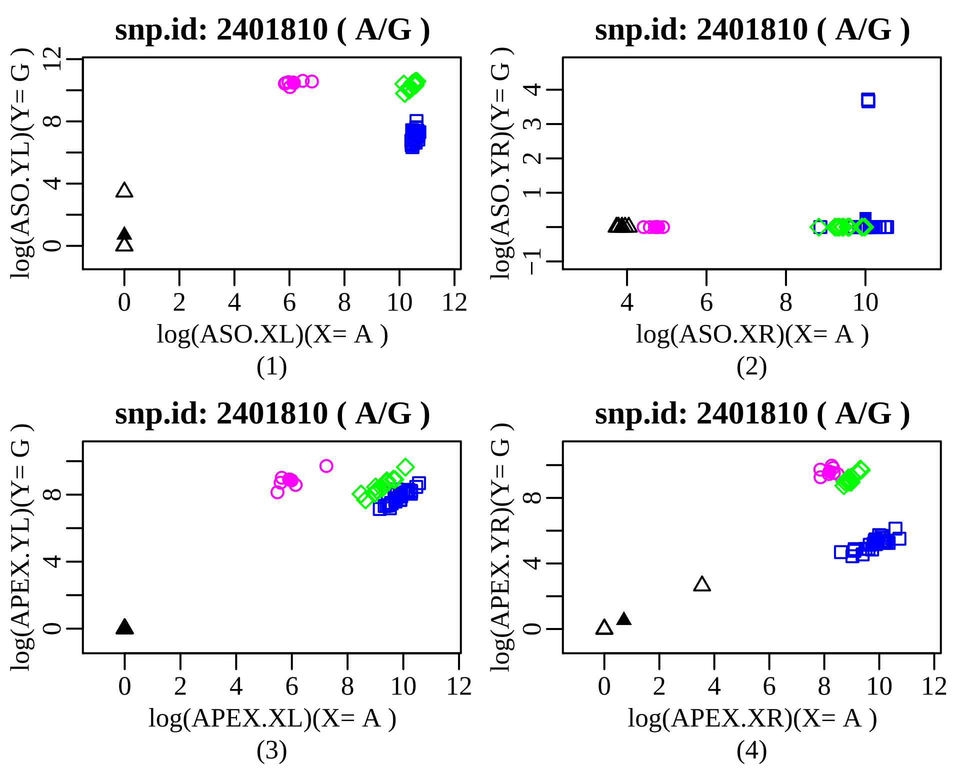
snp.id: 2401810 (A/G)
log(ASO.YL)(Y= G)
log(ASO.XL)(X= A)
(1)
snp.id: 2401810 (A/G)
log(ASO.YR)(Y= G)
log(ASO.XR)(X= A)
(2)
snp.id: 2401810 (A/G)
log(APEX.YL)(Y= G)
log(APEX.XL)(X= A)
(3)
snp.id: 2401810 (A/G)
log(APEX.YR)(Y= G)
log(APEX.XR)(X= A)
(4)

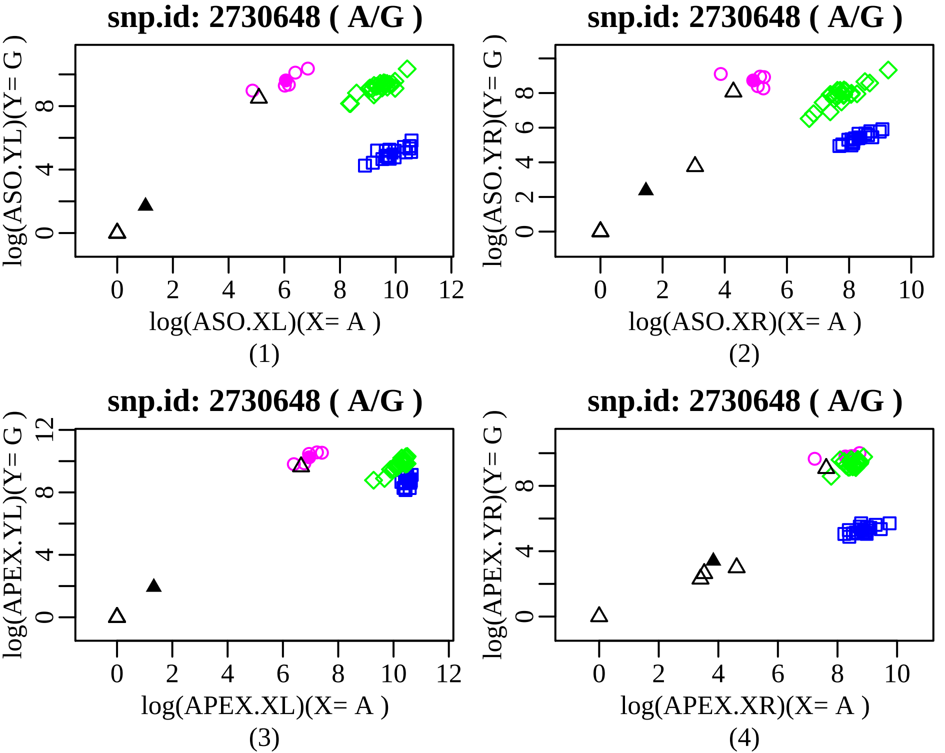
snp.id: 2730648 (A/G)
log(ASO.YL)(Y= G)
log(ASO.XL)(X= A)
(1)
snp.id: 2730648 (A/G)
log(ASO.YR)(Y= G)
log(ASO.XR)(X= A)
(2)
snp.id: 2730648 (A/G)
log(APEX.YL)(Y= G)
log(APEX.XL)(X= A)
(3)
snp.id: 2730648 (A/G)
log(APEX.YR)(Y= G)
log(APEX.XR)(X= A)
(4)

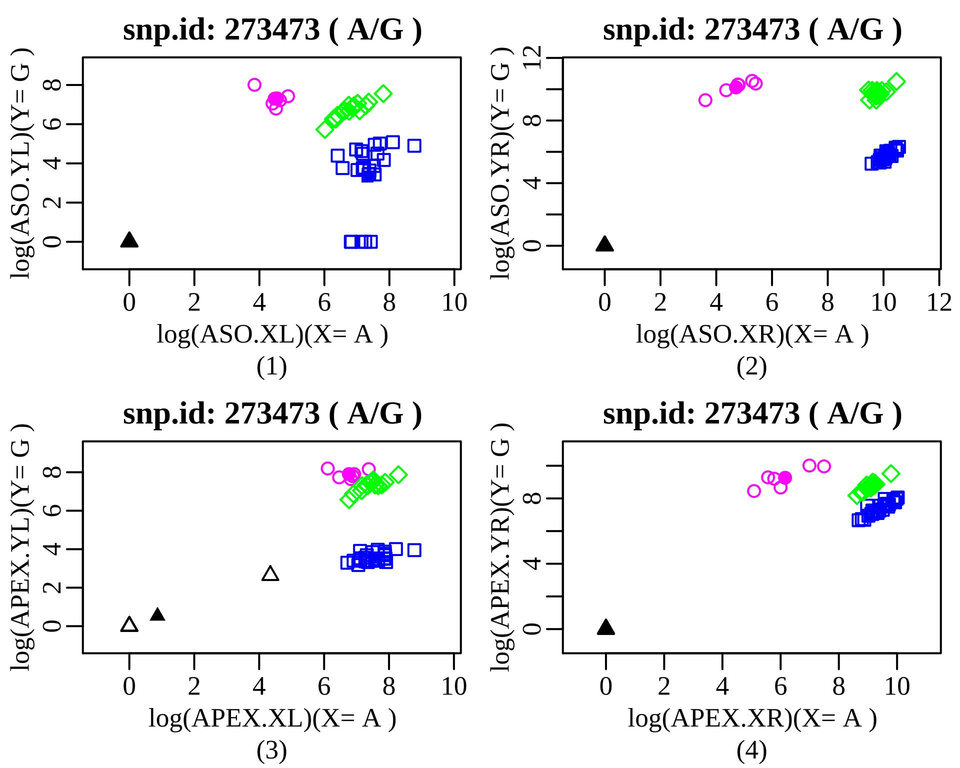
snp.id: 273473 (A/G)
log(ASO.YL)(Y= G)
log(ASO.XL)(X= A)
(1)
snp.id: 273473 (A/G)
log(ASO.YR)(Y= G)
log(ASO.XR)(X= A)
(2)
snp.id: 273473 (A/G)
log(APEX.YL)(Y= G)
log(APEX.XL)(X= A)
(3)
snp.id: 273473 (A/G)
log(APEX.YR)(Y= G)
log(APEX.XR)(X= A)
(4)

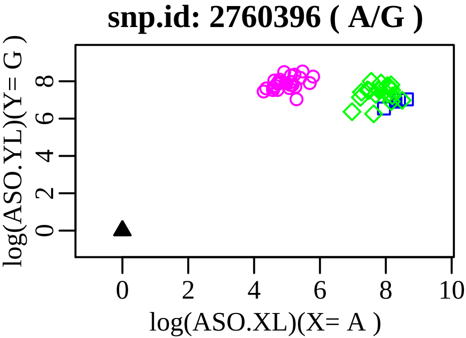
snp.id: 2760396 (A/G)
log(ASO.YL)(Y= G)
0 2 4 6 8
0 2 4 6 8 10
log(ASO.XL)(X= A)
(1)

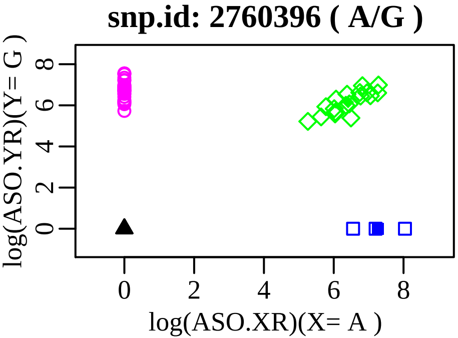
snp.id: 2760396 (A/G)
log(ASO.YR)(Y= G)
0 2 4 6 8
0 2 4 6 8
log(ASO.XR)(X= A)
(2)

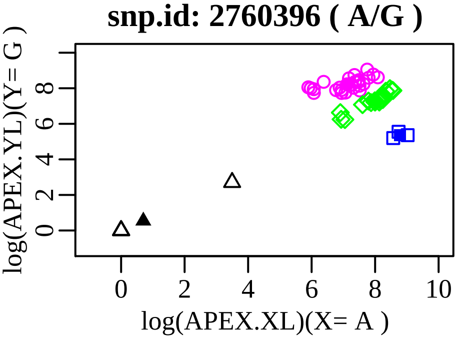
snp.id: 2760396 (A/G)
log(APEX.YL)(Y= G)
0 2 4 6 8
0 2 4 6 8 10
log(APEX.XL)(X= A)
(3)

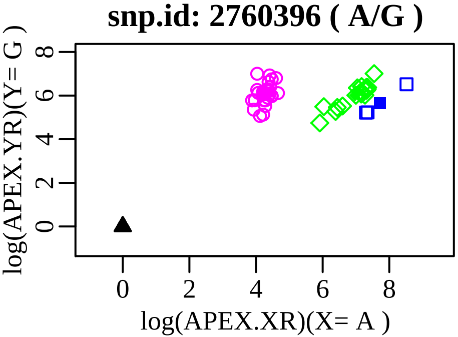
snp.id: 2760396 (A/G)
log(APEX.YR)(Y= G)
0 2 4 6 8
0 2 4 6 8
log(APEX.XR)(X= A)
(4)

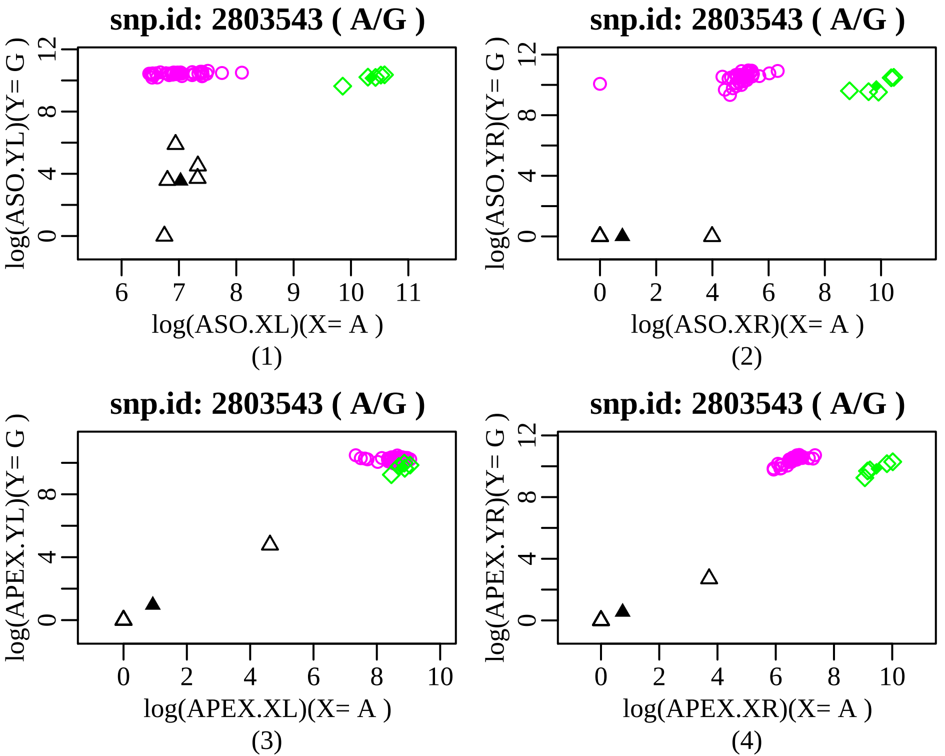
snp.id: 2803543 (A/G)
log(ASO.YL)(Y= G)
log(ASO.XL)(X= A)
(1)
snp.id: 2803543 (A/G)
log(ASO.YR)(Y= G)
log(ASO.XR)(X= A)
(2)
snp.id: 2803543 (A/G)
log(APEX.YL)(Y= G)
log(APEX.XL)(X= A)
(3)
snp.id: 2803543 (A/G)
log(APEX.YR)(Y= G)
log(APEX.XR)(X= A)
(4)

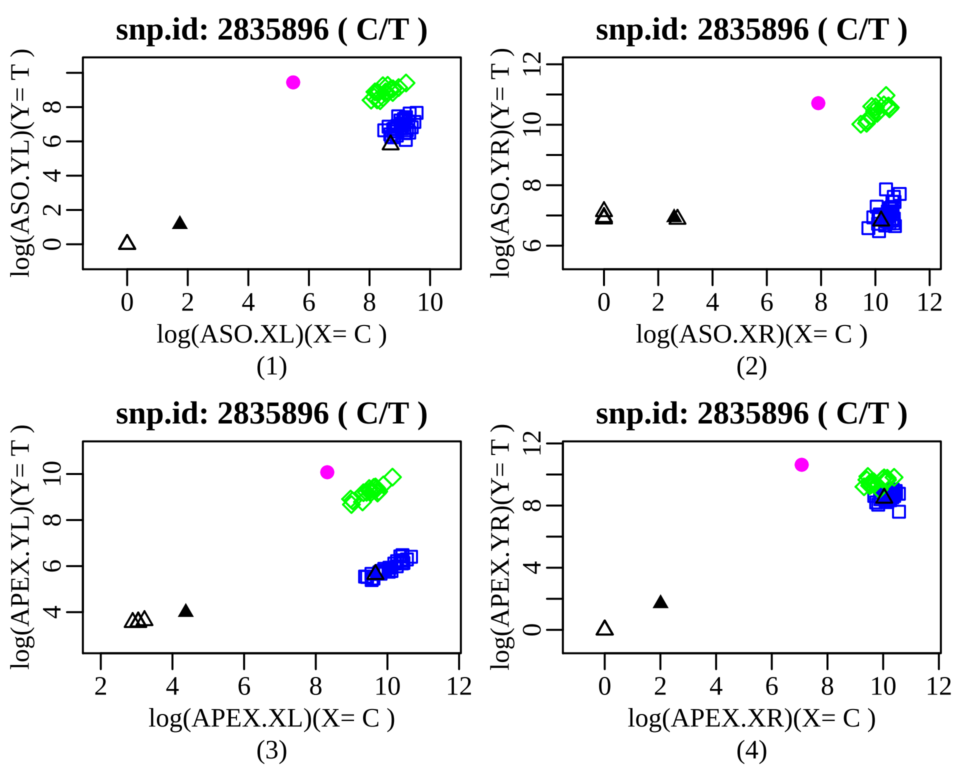
snp.id: 2835896 (C/T)
log(ASO.YL)(Y= T)
log(ASO.XL)(X= C)
(1)
snp.id: 2835896 (C/T)
log(ASO.YR)(Y= T)
log(ASO.XR)(X= C)
(2)
snp.id: 2835896 (C/T)
log(APEX.YL)(Y= T)
log(APEX.XL)(X= C)
(3)
snp.id: 2835896 (C/T)
log(APEX.YR)(Y= T)
log(APEX.XR)(X= C)
(4)

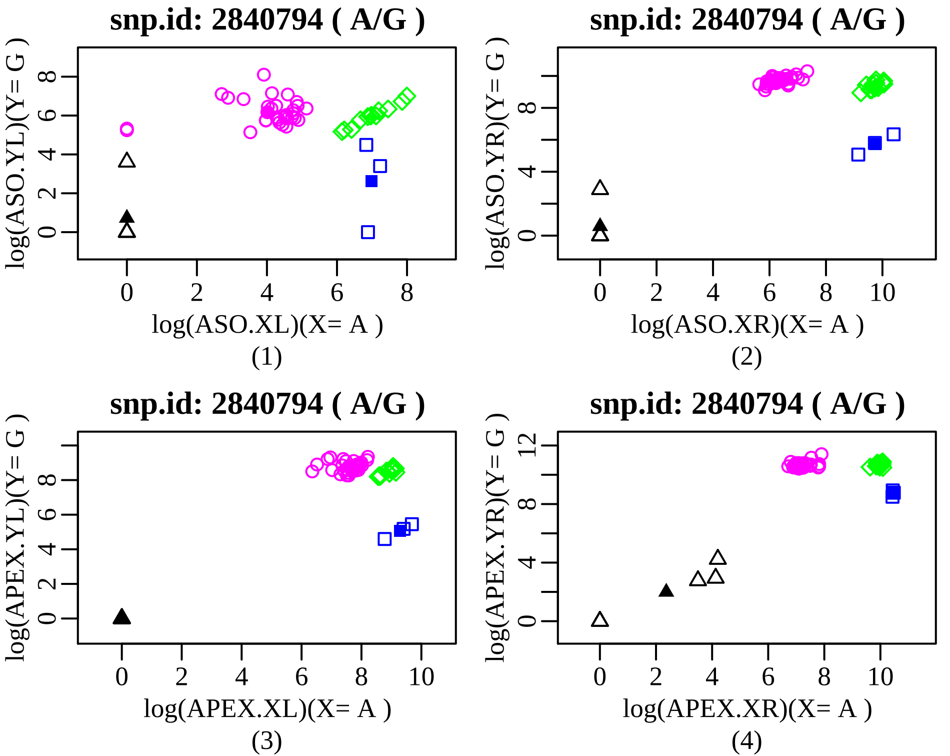

snp.id: 2840794 (A/G)
log(ASO.YL)(Y= G)
0
2
4
6
8
0
2
4
6
8
log(ASO.XL)(X= A)
(1)
snp.id: 2840794 (A/G)
log(ASO.YR)(Y= G)
0
4
8
0
2
4
6
8
10
log(ASO.XR)(X= A)
(2)
snp.id: 2840794 (A/G)
log(APEX.YL)(Y= G)
0
2
4
6
8
0
2
4
6
8
10
log(APEX.XL)(X= A)
(3)
snp.id: 2840794 (A/G)
log(APEX.YR)(Y= G)
0
4
8
12
0
2
4
6
8
10
log(APEX.XR)(X= A)
(4)

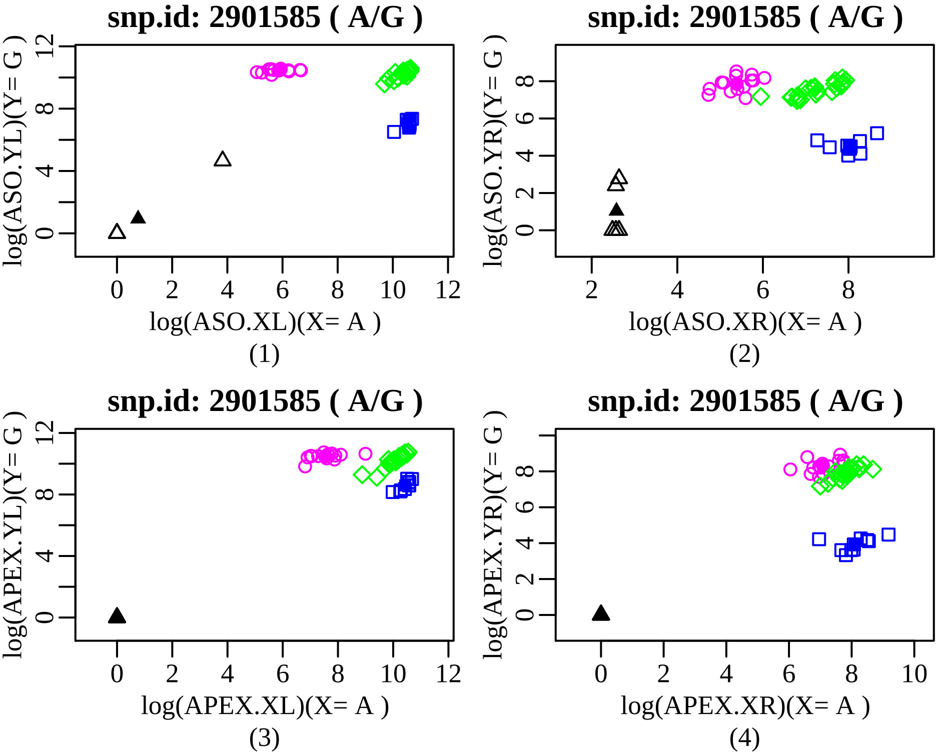

snp.id: 2901585 (A/G)
log(ASO.YL)(Y= G)
log(ASO.XL)(X= A)
0
2
4
6
8
10
12
0
4
8
12
(1)
snp.id: 2901585 (A/G)
log(ASO.YR)(Y= G)
log(ASO.XR)(X= A)
2
4
6
8
0
2
4
6
8
(2)
snp.id: 2901585 (A/G)
log(APEX.YL)(Y= G)
log(APEX.XL)(X= A)
0
2
4
6
8
10
12
0
4
8
12
(3)
snp.id: 2901585 (A/G)
log(APEX.YR)(Y= G)
log(APEX.XR)(X= A)
0
2
4
6
8
10
0
2
4
6
8
(4)

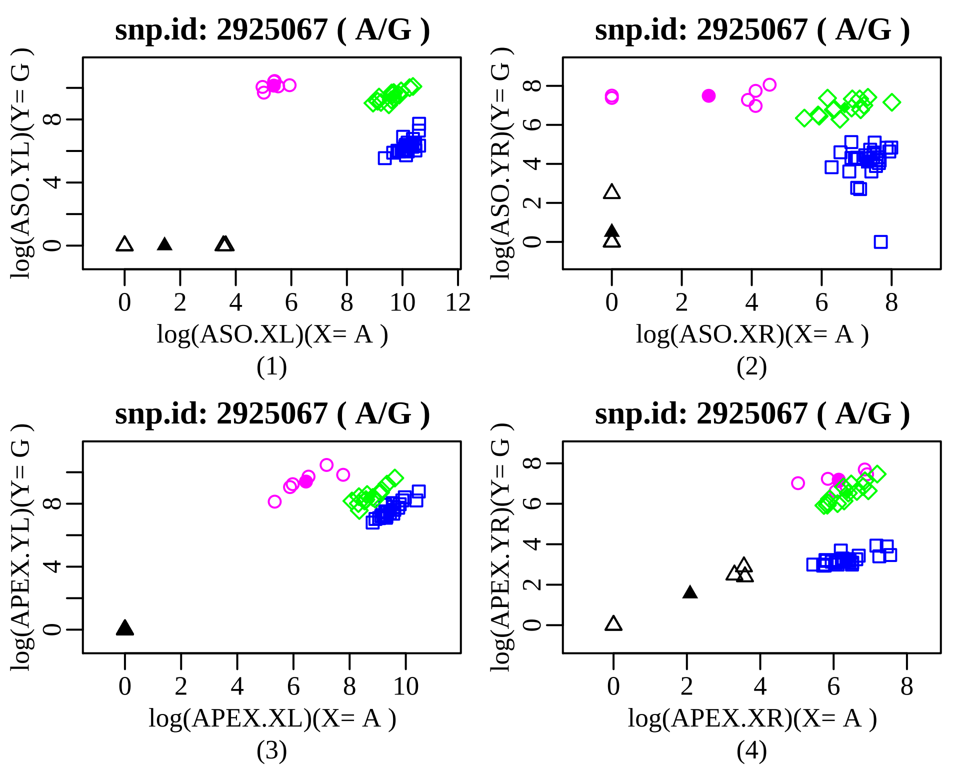
snp.id: 2925067 (A/G)
log(ASO.YL)(Y= G)
log(ASO.XL)(X= A)
(1)
snp.id: 2925067 (A/G)
log(ASO.YR)(Y= G)
log(ASO.XR)(X= A)
(2)
snp.id: 2925067 (A/G)
log(APEX.YL)(Y= G)
log(APEX.XL)(X= A)
(3)
snp.id: 2925067 (A/G)
log(APEX.YR)(Y= G)
log(APEX.XR)(X= A)
(4)

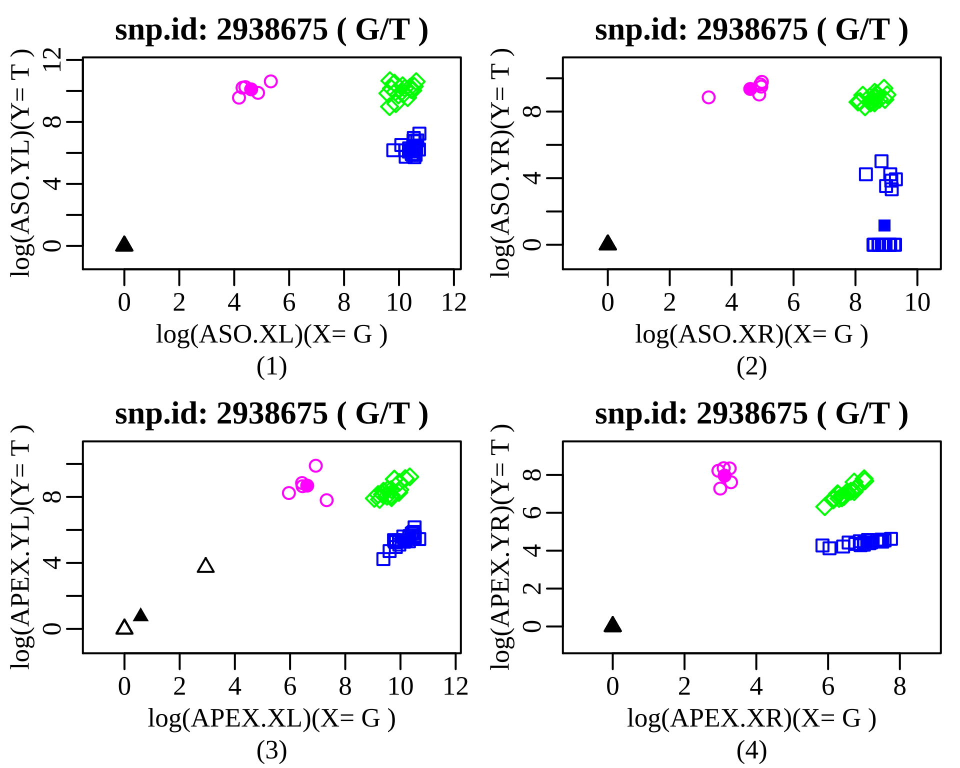
snp.id: 2938675 (G/T)
log(ASO.YL)(Y= T)
log(ASO.XL)(X= G)
(1)
snp.id: 2938675 (G/T)
log(ASO.YR)(Y= T)
log(ASO.XR)(X= G)
(2)
snp.id: 2938675 (G/T)
log(APEX.YL)(Y= T)
log(APEX.XL)(X= G)
(3)
snp.id: 2938675 (G/T)
log(APEX.YR)(Y= T)
log(APEX.XR)(X= G)
(4)

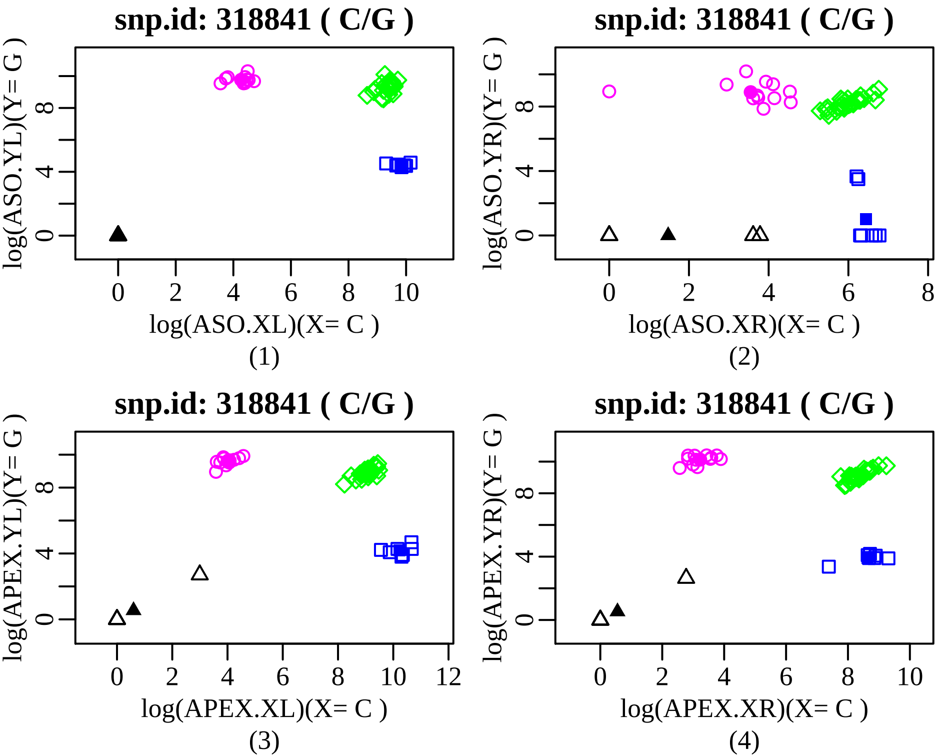
snp.id: 318841 (C/G)
log(ASO.YL)(Y= G)
0
4
8
0
2
4
6
8
10
log(ASO.XL)(X= C)
(1)
snp.id: 318841 (C/G)
log(ASO.YR)(Y= G)
0
4
8
0
2
4
6
8
log(ASO.XR)(X= C)
(2)
snp.id: 318841 (C/G)
log(APEX.YL)(Y= G)
0
4
8
0
2
4
6
8
10
12
log(APEX.XL)(X= C)
(3)
snp.id: 318841 (C/G)
log(APEX.YR)(Y= G)
0
4
8
0
2
4
6
8
10
log(APEX.XR)(X= C)
(4)

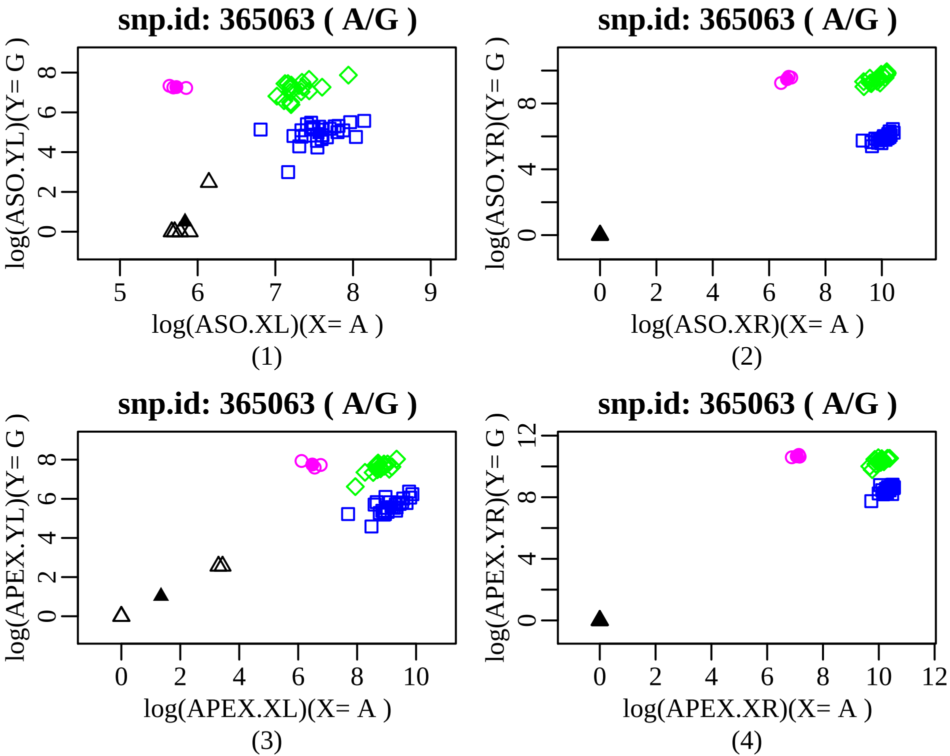
snp.id: 365063 (A/G)
log(ASO.YL)(Y= G)
log(ASO.XL)(X= A)
(1)
snp.id: 365063 (A/G)
log(ASO.YR)(Y= G)
log(ASO.XR)(X= A)
(2)
snp.id: 365063 (A/G)
log(APEX.YL)(Y= G)
log(APEX.XL)(X= A)
(3)
snp.id: 365063 (A/G)
log(APEX.YR)(Y= G)
log(APEX.XR)(X= A)
(4)

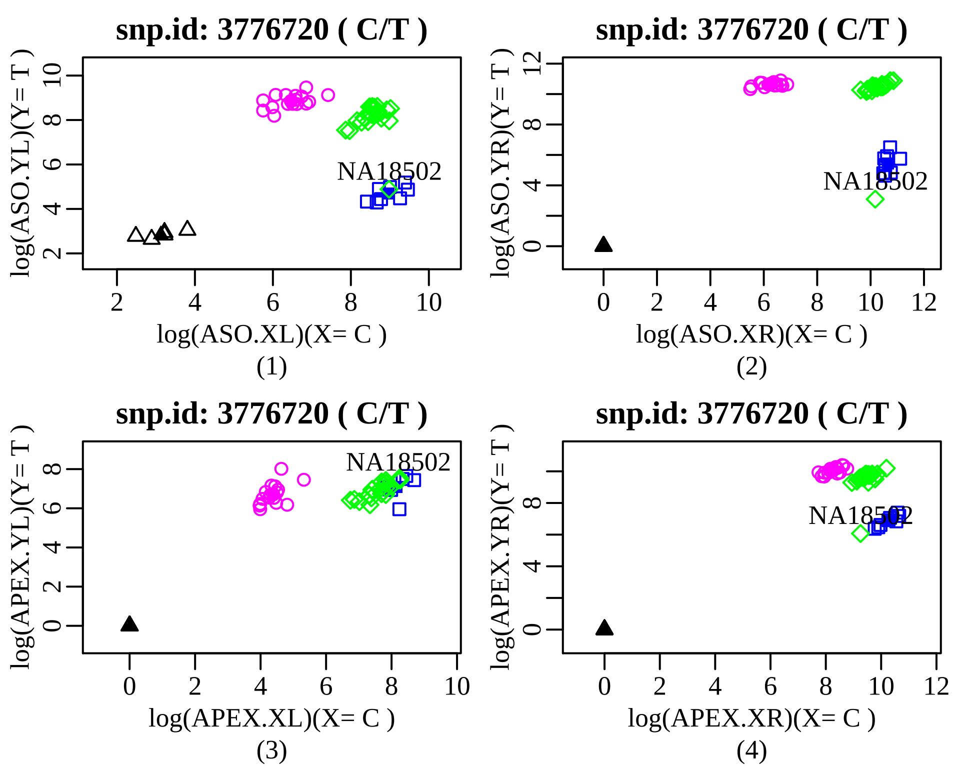
snp.id: 3776720 (C/T)
log(ASO.YL)(Y= T)
log(ASO.XL)(X= C)
NA18502
(1)
snp.id: 3776720 (C/T)
log(ASO.YR)(Y= T)
log(ASO.XR)(X= C)
NA18502
(2)
snp.id: 3776720 (C/T)
log(APEX.YL)(Y= T)
log(APEX.XL)(X= C)
NA18502
(3)
snp.id: 3776720 (C/T)
log(APEX.YR)(Y= T)
log(APEX.XR)(X= C)
NA18502
(4)

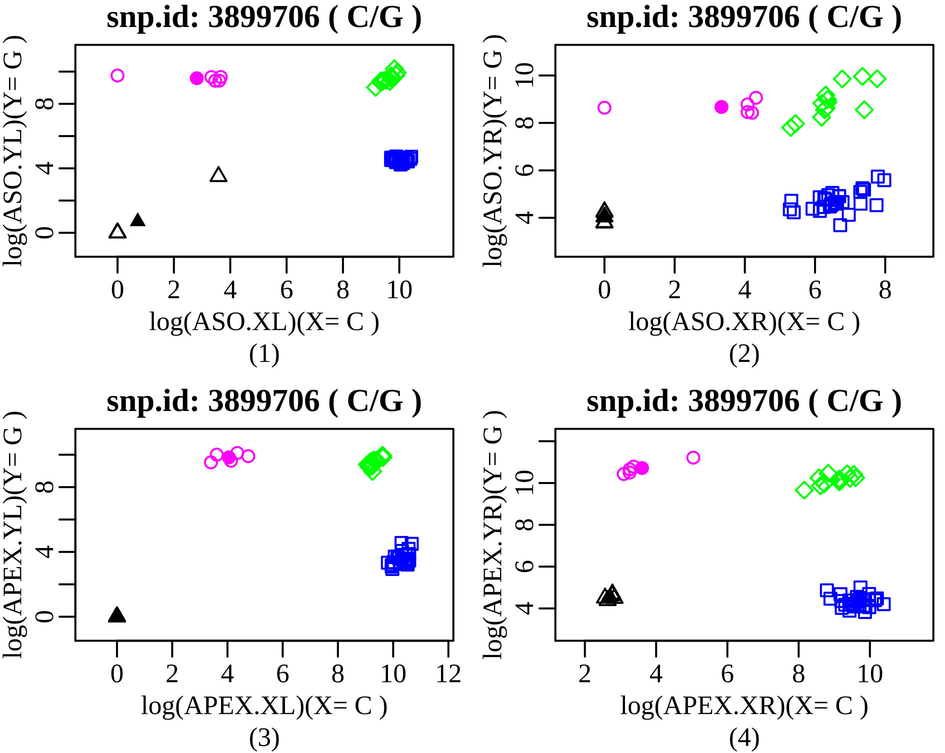
snp.id: 3899706 (C/G)
log(ASO.YL)(Y= G)
log(ASO.XL)(X= C)
(1)
snp.id: 3899706 (C/G)
log(ASO.YR)(Y= G)
log(ASO.XR)(X= C)
(2)
snp.id: 3899706 (C/G)
log(APEX.YL)(Y= G)
log(APEX.XL)(X= C)
(3)
snp.id: 3899706 (C/G)
log(APEX.YR)(Y= G)
log(APEX.XR)(X= C)
(4)

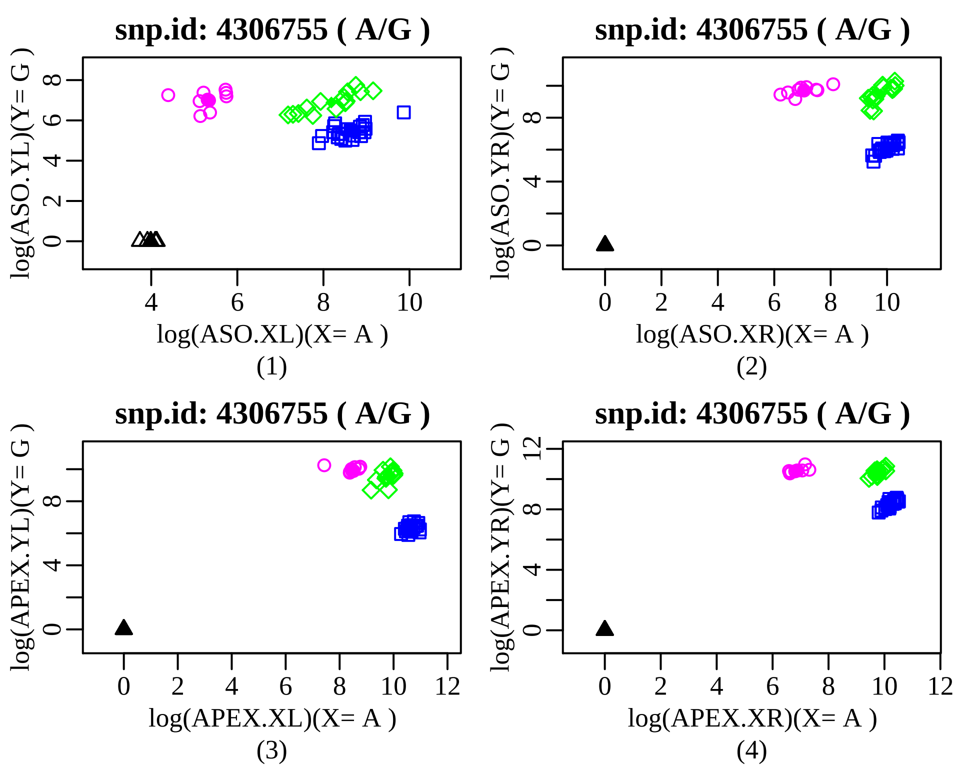
snp.id: 4306755 (A/G)
log(ASO.YL)(Y= G)
log(ASO.XL)(X= A)
(1)
snp.id: 4306755 (A/G)
log(ASO.YR)(Y= G)
log(ASO.XR)(X= A)
(2)
snp.id: 4306755 (A/G)
log(APEX.YL)(Y= G)
log(APEX.XL)(X= A)
(3)
snp.id: 4306755 (A/G)
log(APEX.YR)(Y= G)
log(APEX.XR)(X= A)
(4)

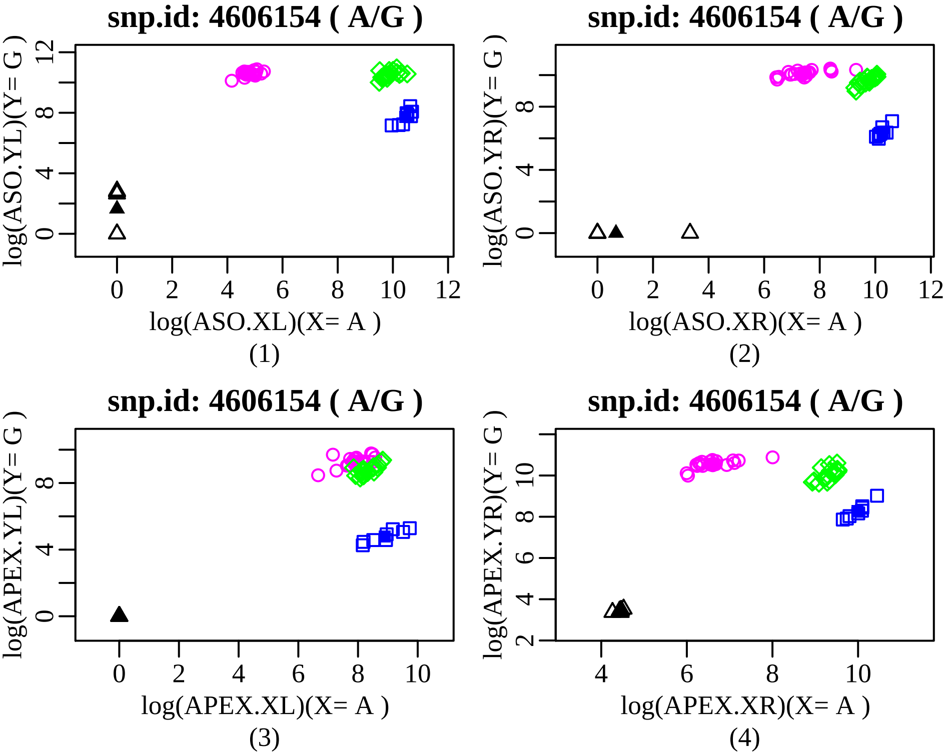
snp.id: 4606154 (A/G)
log(ASO.YL)(Y= G)
log(ASO.XL)(X= A)
(1)
snp.id: 4606154 (A/G)
log(ASO.YR)(Y= G)
log(ASO.XR)(X= A)
(2)
snp.id: 4606154 (A/G)
log(APEX.YL)(Y= G)
log(APEX.XL)(X= A)
(3)
snp.id: 4606154 (A/G)
log(APEX.YR)(Y= G)
log(APEX.XR)(X= A)
(4)

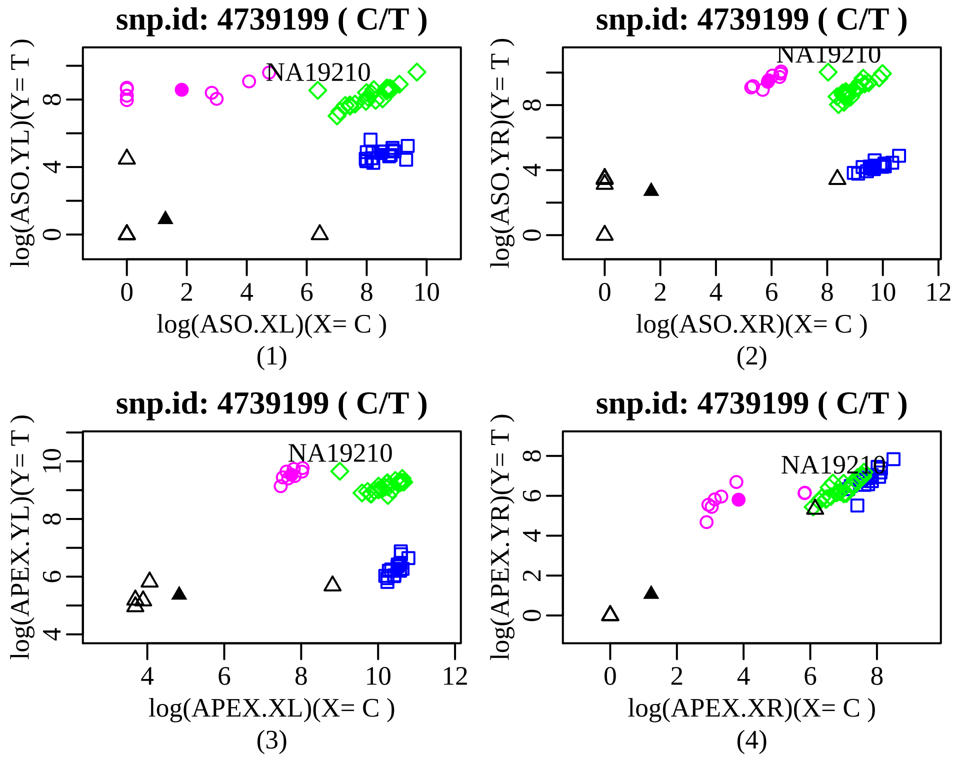
snp.id: 4739199 (C/T)
NA19210
log(ASO.YL)(Y= T)
log(ASO.XL)(X= C)
(1)
snp.id: 4739199 (C/T)
NA19210
log(ASO.YR)(Y= T)
log(ASO.XR)(X= C)
(2)
snp.id: 4739199 (C/T)
NA19210
log(APEX.YL)(Y= T)
log(APEX.XL)(X= C)
(3)
snp.id: 4739199 (C/T)
NA19210
log(APEX.YR)(Y= T)
log(APEX.XR)(X= C)
(4)

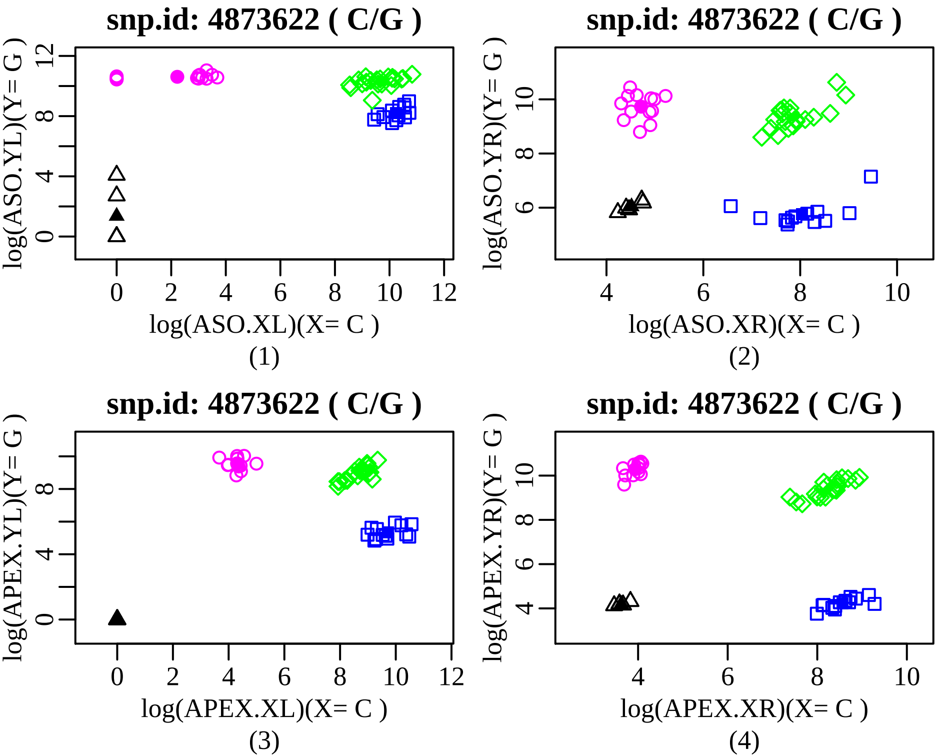
snp.id: 4873622 (C/G)
log(ASO.YL)(Y= G)
log(ASO.XL)(X= C)
(1)
snp.id: 4873622 (C/G)
log(ASO.YR)(Y= G)
log(ASO.XR)(X= C)
(2)
snp.id: 4873622 (C/G)
log(APEX.YL)(Y= G)
log(APEX.XL)(X= C)
(3)
snp.id: 4873622 (C/G)
log(APEX.YR)(Y= G)
log(APEX.XR)(X= C)
(4)

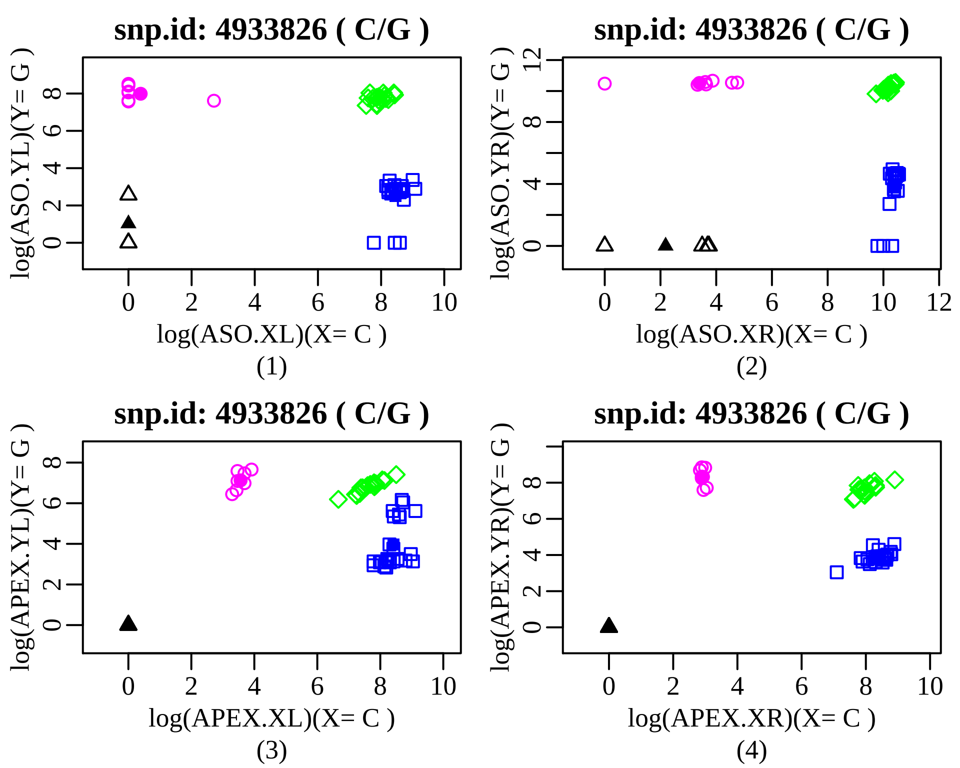

snp.id: 4933826 (C/G)
log(ASO.YL)(Y= G)
log(ASO.XL)(X= C)
0
2
4
6
8
10
(1)
snp.id: 4933826 (C/G)
log(ASO.YR)(Y= G)
log(ASO.XR)(X= C)
0
2
4
6
8
10
12
(2)
snp.id: 4933826 (C/G)
log(APEX.YL)(Y= G)
log(APEX.XL)(X= C)
0
2
4
6
8
10
(3)
snp.id: 4933826 (C/G)
log(APEX.YR)(Y= G)
log(APEX.XR)(X= C)
0
2
4
6
8
10
(4)

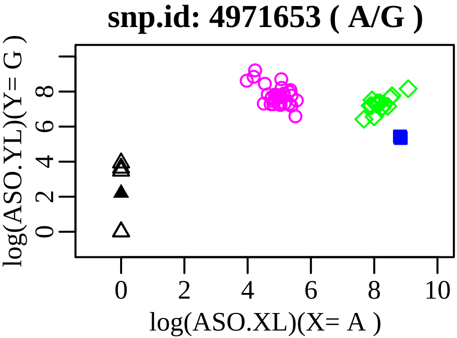
snp.id: 4971653 (A/G)
log(ASO.YL)(Y= G)
log(ASO.XL)(X= A)
(1)

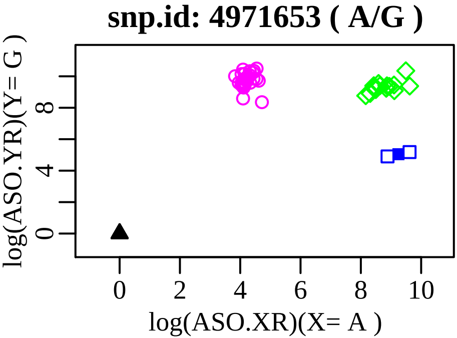
snp.id: 4971653 (A/G)
log(ASO.YR)(Y= G)
log(ASO.XR)(X= A)
(2)

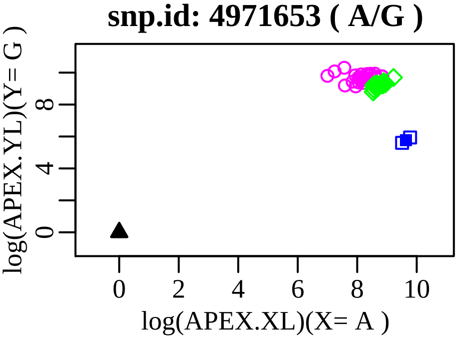
snp.id: 4971653 (A/G)
log(APEX.YL)(Y= G)
log(APEX.XL)(X= A)
(3)

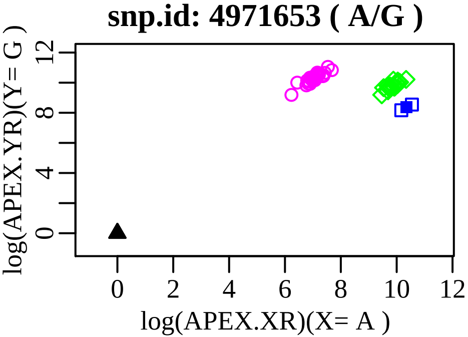
snp.id: 4971653 (A/G)
log(APEX.YR)(Y= G)
log(APEX.XR)(X= A)
(4)

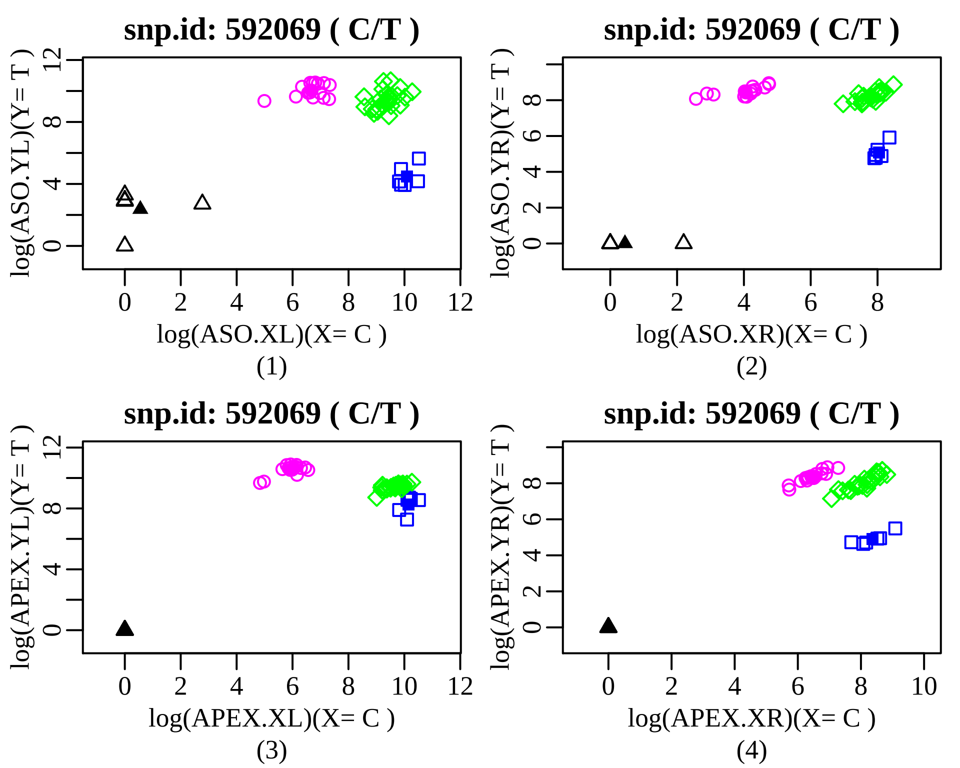
snp.id: 592069 (C/T)
log(ASO.YL)(Y= T)
log(ASO.XL)(X= C)
(1)
snp.id: 592069 (C/T)
log(ASO.YR)(Y= T)
log(ASO.XR)(X= C)
(2)
snp.id: 592069 (C/T)
log(APEX.YL)(Y= T)
log(APEX.XL)(X= C)
(3)
snp.id: 592069 (C/T)
log(APEX.YR)(Y= T)
log(APEX.XR)(X= C)
(4)

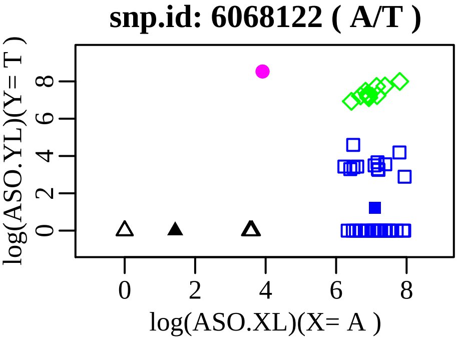
snp.id: 6068122 (A/T)
log(ASO.YL)(Y= T)
0
2
4
6
8
log(ASO.XL)(X= A)

(1)

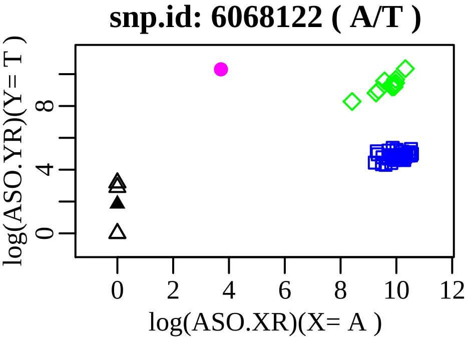
snp.id: 6068122 (A/T)
log(ASO.YR)(Y= T)
0
4
8
0
2
4
6
8
10
12
log(ASO.XR)(X= A)

(2)

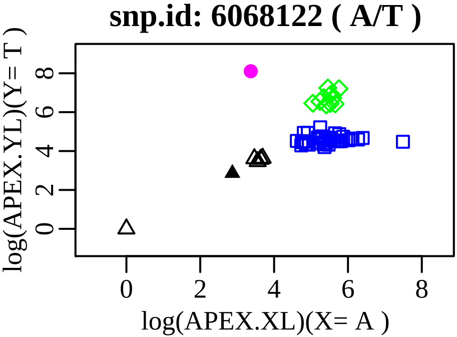
snp.id: 6068122 (A/T)
log(APEX.YL)(Y= T)
0
2
4
6
8
log(APEX.XL)(X= A)

(3)

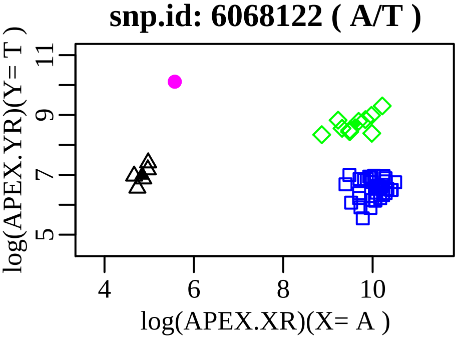
snp.id: 6068122 (A/T)
log(APEX.YR)(Y= T)
5
7
9
11
4
6
8
10
log(APEX.XR)(X= A)

(4)

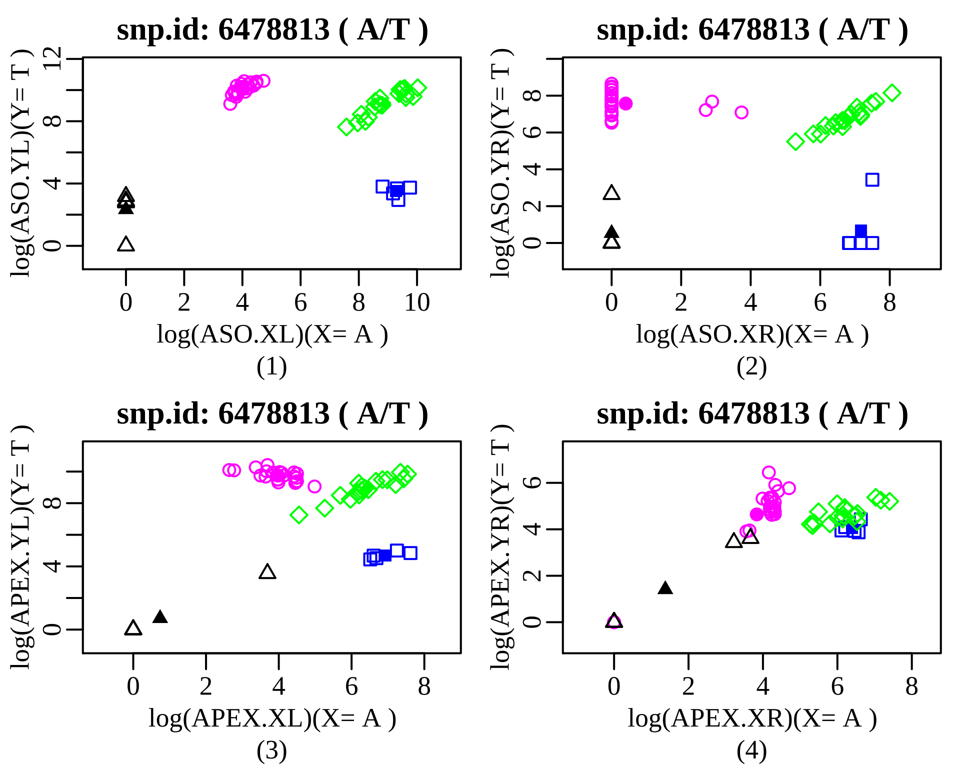

snp.id: 6478813 (A/T)
log(ASO.YL)(Y= T)
log(ASO.XL)(X= A)
(1)
snp.id: 6478813 (A/T)
log(ASO.YR)(Y= T)
log(ASO.XR)(X= A)
(2)
snp.id: 6478813 (A/T)
log(APEX.YL)(Y= T)
log(APEX.XL)(X= A)
(3)
snp.id: 6478813 (A/T)
log(APEX.YR)(Y= T)
log(APEX.XR)(X= A)
(4)

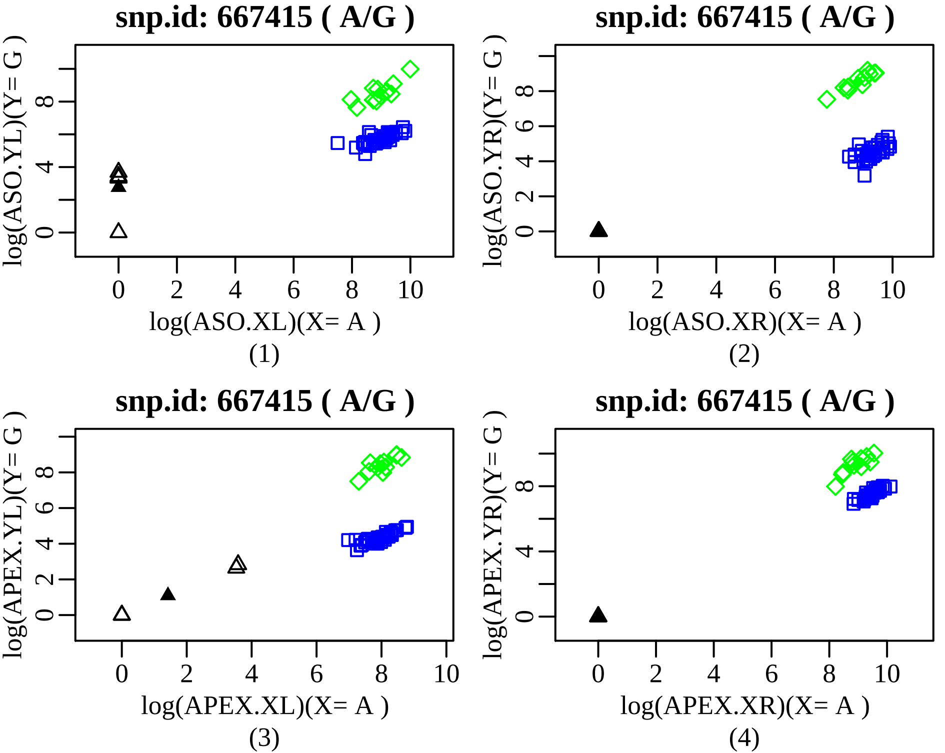
snp.id: 667415 (A/G)
log(ASO.YL)(Y= G)
0
4
8
0
2
4
6
8
10
log(ASO.XL)(X= A)
(1)
snp.id: 667415 (A/G)
log(ASO.YR)(Y= G)
0
2
4
6
8
0
2
4
6
8
10
log(ASO.XR)(X= A)
(2)
snp.id: 667415 (A/G)
log(APEX.YL)(Y= G)
0
2
4
6
8
0
2
4
6
8
10
log(APEX.XL)(X= A)
(3)
snp.id: 667415 (A/G)
log(APEX.YR)(Y= G)
0
4
8
0
2
4
6
8
10
log(APEX.XR)(X= A)
(4)

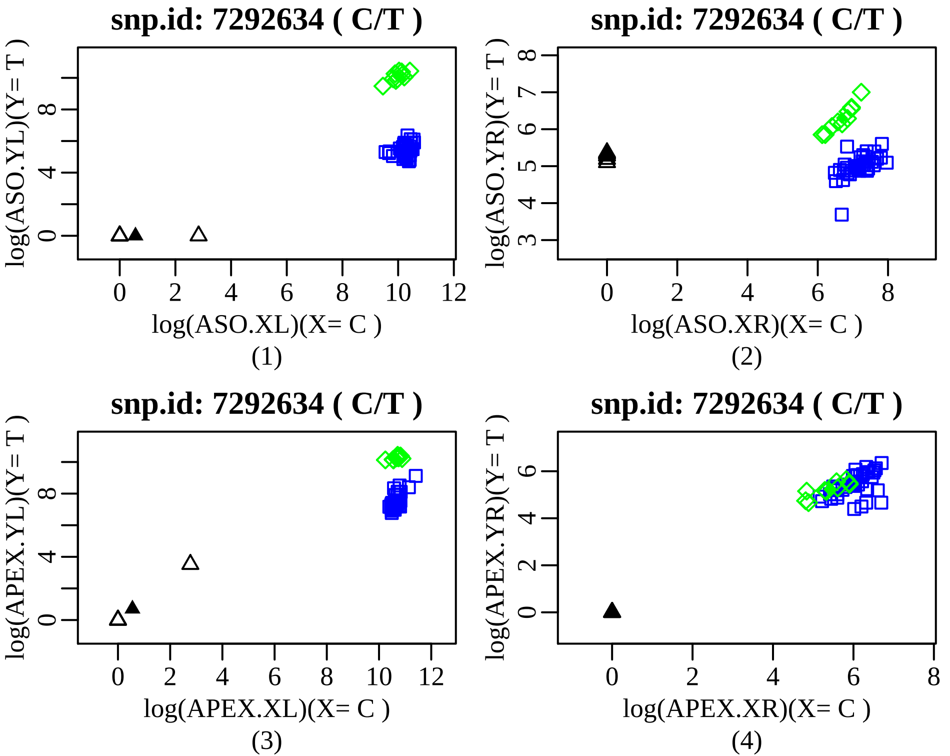
snp.id: 7292634 (C/T)
log(ASO.YL)(Y= T)
log(ASO.XL)(X= C)
(1)
snp.id: 7292634 (C/T)
log(ASO.YR)(Y= T)
log(ASO.XR)(X= C)
(2)
snp.id: 7292634 (C/T)
log(APEX.YL)(Y= T)
log(APEX.XL)(X= C)
(3)
snp.id: 7292634 (C/T)
log(APEX.YR)(Y= T)
log(APEX.XR)(X= C)
(4)

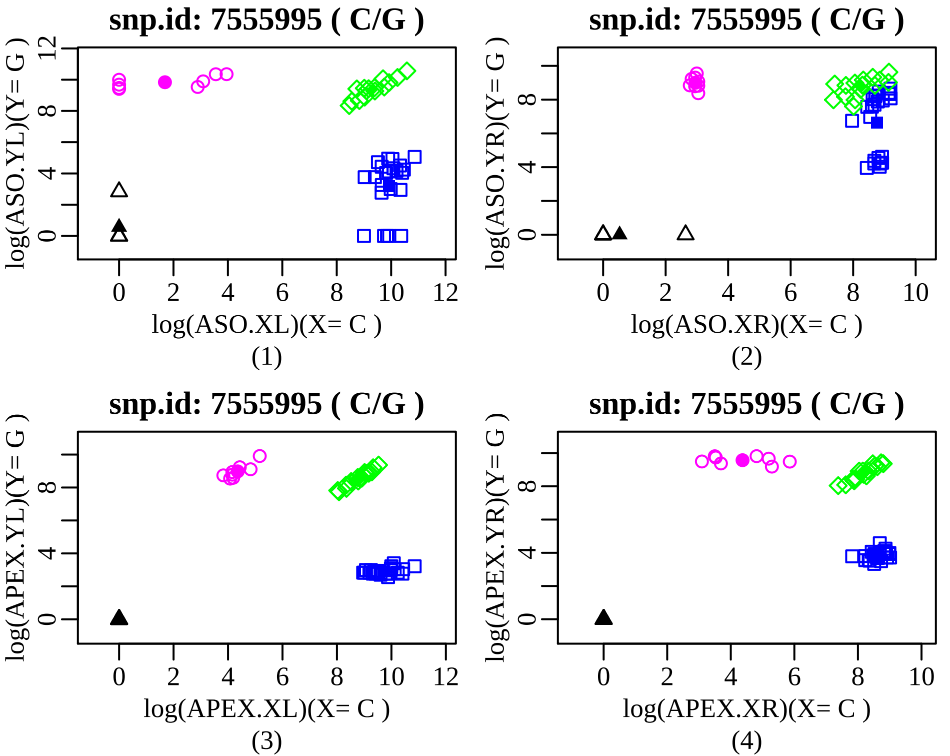
snp.id: 7555995 (C/G)
log(ASO.XL)(X= C)
log(ASO.YL)(Y= G)
(1)
snp.id: 7555995 (C/G)
log(ASO.XR)(X= C)
log(ASO.YR)(Y= G)
(2)
snp.id: 7555995 (C/G)
log(APEX.XL)(X= C)
log(APEX.YL)(Y= G)
(3)
snp.id: 7555995 (C/G)
log(APEX.XR)(X= C)
log(APEX.YR)(Y= G)
(4)

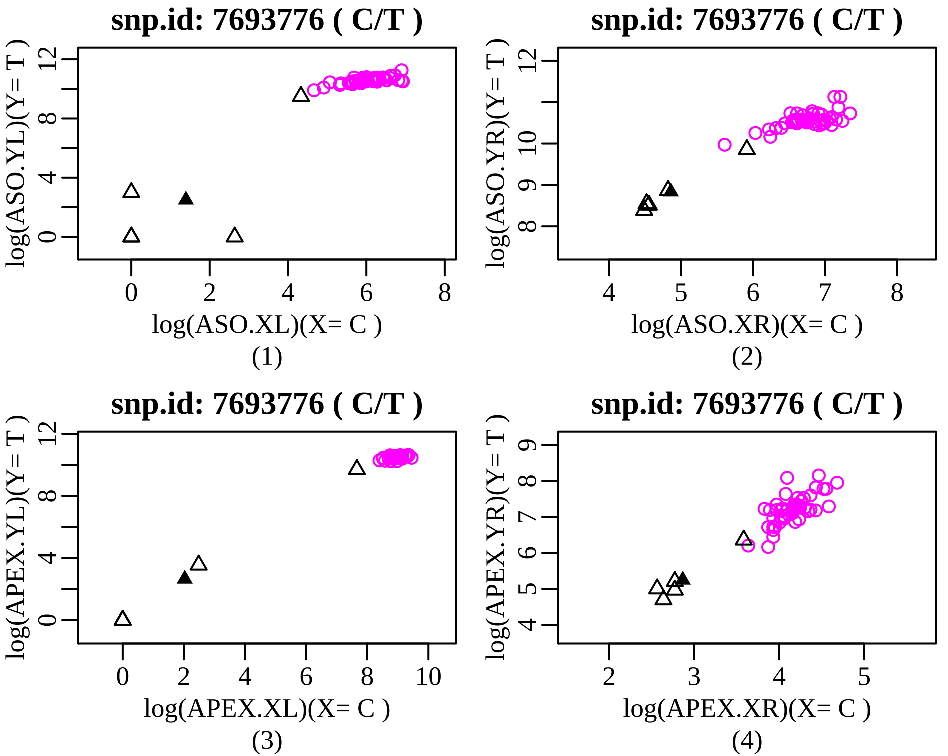
snp.id: 7693776 (C/T)
log(ASO.YL)(Y= T)
log(ASO.XL)(X= C)
(1)
snp.id: 7693776 (C/T)
log(ASO.YR)(Y= T)
log(ASO.XR)(X= C)
(2)
snp.id: 7693776 (C/T)
log(APEX.YL)(Y= T)
log(APEX.XL)(X= C)
(3)
snp.id: 7693776 (C/T)
log(APEX.YR)(Y= T)
log(APEX.XR)(X= C)
(4)

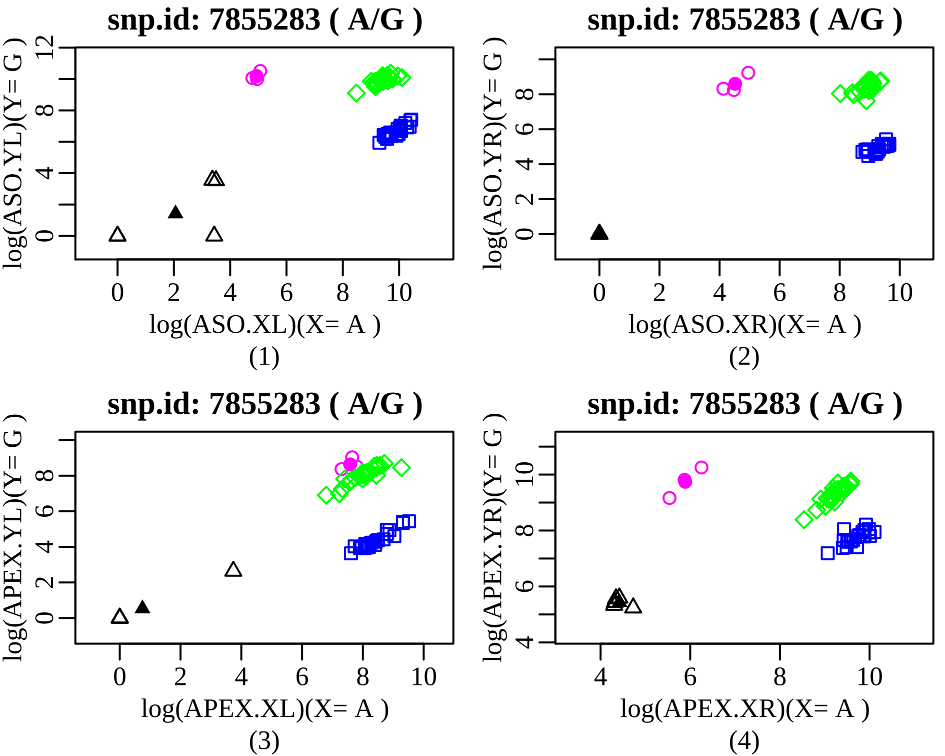
snp.id: 7855283 (A/G)
log(ASO.YL)(Y= G)
log(ASO.XL)(X= A)
(1)
snp.id: 7855283 (A/G)
log(ASO.YR)(Y= G)
log(ASO.XR)(X= A)
(2)
snp.id: 7855283 (A/G)
log(APEX.YL)(Y= G)
log(APEX.XL)(X= A)
(3)
snp.id: 7855283 (A/G)
log(APEX.YR)(Y= G)
log(APEX.XR)(X= A)
(4)

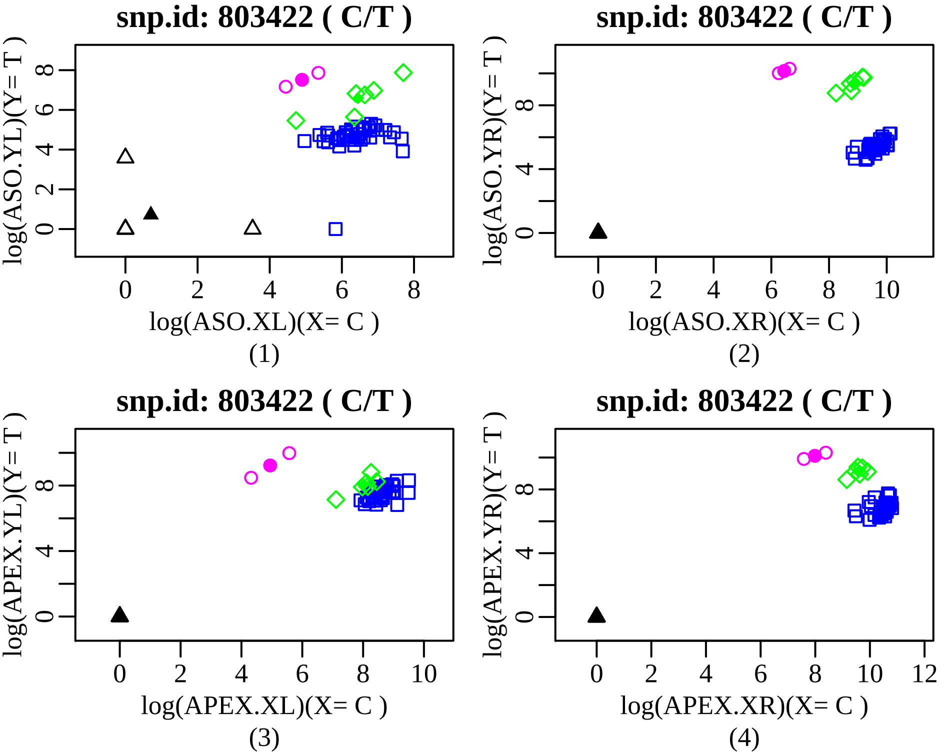
snp.id: 803422 (C/T)
log(ASO.YL)(Y= T)
log(ASO.XL)(X= C)
(1)
0
2
4
6
8
snp.id: 803422 (C/T)
log(ASO.YR)(Y= T)
log(ASO.XR)(X= C)
(2)
0
4
8
0
2
4
6
8
10
snp.id: 803422 (C/T)
log(APEX.YL)(Y= T)
log(APEX.XL)(X= C)
(3)
0
4
8
0
2
4
6
8
10
snp.id: 803422 (C/T)
log(APEX.YR)(Y= T)
log(APEX.XR)(X= C)
(4)
0
4
8
0
2
4
6
8
10
12

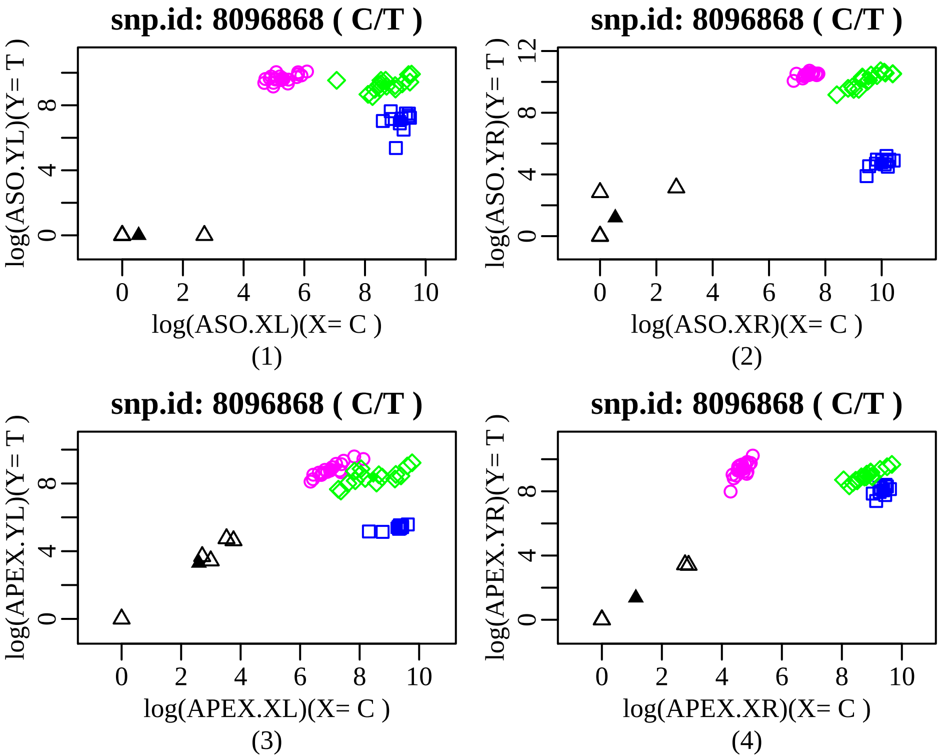

snp.id: 8096868 (C/T)
log(ASO.YL)(Y= T)
log(ASO.XL)(X= C)
(1)
snp.id: 8096868 (C/T)
log(ASO.YR)(Y= T)
log(ASO.XR)(X= C)
(2)
snp.id: 8096868 (C/T)
log(APEX.YL)(Y= T)
log(APEX.XL)(X= C)
(3)
snp.id: 8096868 (C/T)
log(APEX.YR)(Y= T)
log(APEX.XR)(X= C)
(4)

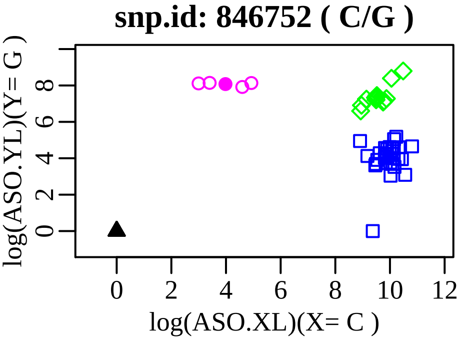
snp.id: 846752 (C/G)
log(ASO.YL)(Y= G)
0 2 4 6 8
0 2 4 6 8 10 12
log(ASO.XL)(X= C)
(1)

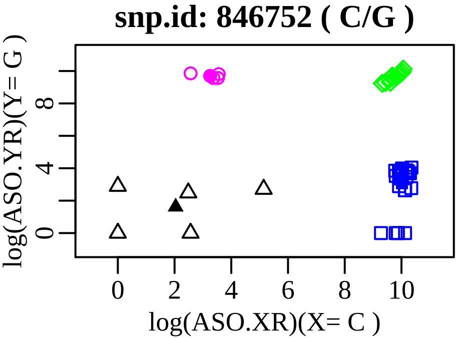
snp.id: 846752 (C/G)
log(ASO.YR)(Y= G)
0 4 8
0 2 4 6 8 10
log(ASO.XR)(X= C)
(2)

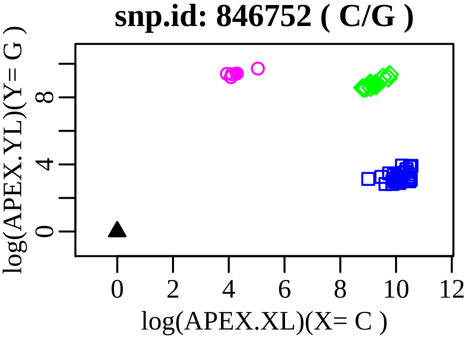
snp.id: 846752 (C/G)
log(APEX.YL)(Y= G)
0 4 8
0 2 4 6 8 10 12
log(APEX.XL)(X= C)
(3)

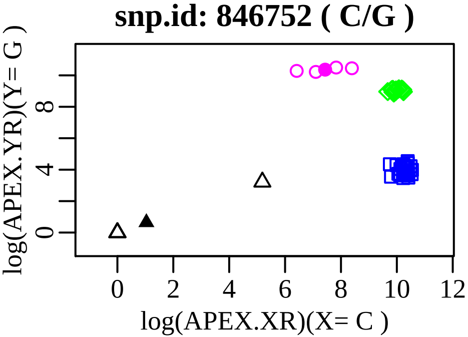
snp.id: 846752 (C/G)
log(APEX.YR)(Y= G)
0 4 8
0 2 4 6 8 10 12
log(APEX.XR)(X= C)
(4)
