# Supplementary material for: Robust SNP genotyping by multiplex PCR and arrayed primer extension
Source: BMC Med Genomics. 2008 Jan 31;1:5. doi: 10.1186/1755-8794-1-5 (PMC2266772; doi:10.1186/1755-8794-1-5)
Supplement: Additional file 12 — Data structure for SNP rs12466929 & DNA sample HapMap 101 (Coriell NA18621 – AA). Illustrative table of microarray four-channel intensity data from 30 spots corresponding to one SNP (rs12466929) and one DNA sample. [file 1755-8794-1-5-S12.pdf]

Additional file 12: Data structure for SNP rs12466929

& DNA sample HapMap 101 (Coriell NA18621 - AA)

Spot ID	Probe ID	Expected allele ID	A	C	G	T
Spot 1	APEX_LEFT	A and/or G	**1394**	314	**29**	82
Spot 2	APEX_LEFT	A and/or G	**1148**	302	**27**	83
Spot 3	APEX_LEFT	A and/or G	**597**	163	**43**	100
Spot 4	APEX_LEFT	A and/or G	**1106**	259	**27**	74
Spot 5	APEX_LEFT	A and/or G	**1504**	423	**32**	85
Spot 6	APEX_RIGHT	T and/or C	62	**943**	72	**4785**
Spot 7	APEX_RIGHT	T and/or C	82	**1300**	90	**5443**
Spot 8	APEX_RIGHT	T and/or C	63	**1672**	95	**6841**
Spot 9	APEX_RIGHT	T and/or C	66	**1086**	68	**4718**
Spot 10	APEX_RIGHT	T and/or C	122	**1220**	70	**4871**
Spot 11	ASO_1LEFT	C	354	**15528**	25	344
Spot 12	ASO_1LEFT	C	506	**22330**	28	446
Spot 13	ASO_1LEFT	C	529	**18018**	31	383
Spot 14	ASO_1LEFT	C	368	**19526**	26	362
Spot 15	ASO_1LEFT	C	403	**17804**	26	381
Spot 16	ASO_2LEFT	C	55	**669**	22	85
Spot 17	ASO_2LEFT	C	112	**345**	27	87
Spot 18	ASO_2LEFT	C	61	**368**	22	66
Spot 19	ASO_2LEFT	C	51	**849**	27	95
Spot 20	ASO_2LEFT	C	60	**683**	17	79
Spot 21	ASO_1RIGHT	G	503	51	**3518**	134
Spot 22	ASO_1RIGHT	G	660	70	**4430**	168
Spot 23	ASO_1RIGHT	G	943	83	**5266**	172
Spot 24	ASO_1RIGHT	G	1128	61	**4377**	144
Spot 25	ASO_1RIGHT	G	871	67	**4063**	156
Spot 26	ASO_2RIGHT	G	136	43	**60**	90
Spot 27	ASO_2RIGHT	G	0	0	**0**	0
Spot 28	ASO_2RIGHT	G	100	14	**26**	49
Spot 29	ASO_2RIGHT	G	35	18	**26**	36
Spot 30	ASO_2RIGHT	G	37	14	**22**	39

In our system, multiple probes associated with each SNP are designed; namely, classical APEX probes and allele-specific APEX (ASO) probes. There are six probes in total for each bi-allelic SNP and each probe has five replicates, giving 30 different spots randomly allocated across the chip for a single SNP. Each spot from the microarray generates four coloured signal intensities corresponding to four different channels A, C, G and T. This table is an example of a data source for a single HapMap sample and a single SNP (genotype AA in this case). The signals which are expected from each spot are highlighted accordingly.
