# Supplementary material for: Robust SNP genotyping by multiplex PCR and arrayed primer extension
Source: BMC Med Genomics. 2008 Jan 31;1:5. doi: 10.1186/1755-8794-1-5 (PMC2266772; doi:10.1186/1755-8794-1-5)
Supplement: Additional file 13 — List of explanatory variables listed by appropriate classifiers. Each of these classifiers consists of a pair of explanatory variables, generically denoted by X and Y, corresponding to two candidate alleles in the SNP position. Values are based on the data shown in Additional file 12. [file 1755-8794-1-5-S13.pdf]

Additional file 13: List of explanatory variables listed by appropriate classifiers

Classifier	Variables used by classifier		Values	
APEX.L	APEX.XL	APEX.YL	1148	29
APEX.R	APEX.XR	APEX.YR	4871	1220
ASO.L	ASO.XL	ASO.YL	18018	669
ASO.R	ASO.XR	ASO.YR	4377	26
